# Proceedings to the 61^st^ Annual Conference of the Particle Therapy Cooperative Group: 10 June – 16 June, 2023 Co-organized by the Quironsalud and the Clinica Universidad de Navarra, Madrid, Spain.

**DOI:** 10.14338/IJPT-23-PTCOG61-10.2

**Published:** 2023-11-03

**Table:** 

Table of Contents	
Program Description	pg. xx
2023 Committees	pg. xx
Oral Presentations	pg. xx
Poster Presentations	pg. xx

## Program Description

### Overview

The theme of PTCOG 61, **Integrative Particle Therapy and Complementary Care**, reflects the importance of integrating particle therapy into comprehensive cancer care. PTCOG 61 began with an educational session in Spanish language, followed by two days of educational programs in English language and three days of multi-session scientific reports, in addition to several subcommittee meetings.

***Research Topics for the 61^st^ annual conference were*:**

**Clinical** (11 Topics)

- Challenging clinical scenario: Why or why not particle therapy
- Comparative effectiveness and cost-effectives analysis
- General clinical results and patient reported outcome
- Particle therapy-based re-irradiation
- Particle therapy-based stereotactic radiotherapy and hypo-fractionated radiotherapy
- Combined particle therapy with novel systemic therapy: efficacy and toxicities
- Long-term complications and image changes after particle therapy
- Complementary therapy in particle therapy (rehabilitation)
- Novel treatment strategies using particle therapy
- Clinical implementation of adaptive particle therapy
- LET and variable RBE based particle therapy

**Physics:** (8 Topics):

- Beam delivery and nozzle design
- Quality Assurance
- Absolute and relative dosimetry
- Treatment planning
- Dose calculation and optimization
- Image guidance
- Adaptive and 4D therapy
- FLASH and mini-beams – Planning, delivery, and dosimetry

**Biology:** (8 Topics)

- iCPRT (immuno charged particle radiotherapy) – what progress has been made?
- Targeted agents with CPRT
- Biomarkers of CPRT response
- Biological advantages for CPRT vs XRT
- Mathematical Modeling and simulations of CPRT
- Integrating biology in treatment planning – RBE and beyond
- BNCT
- FLASH and mini-beams

### Target Audience

Healthcare professionals who treat cancer patients using radiation therapy/particle therapy and specifically:

- Radiation Oncologists
- Medical Physicists
- Dosimetrists
- Residents
- Radiation Therapists

### Particle Therapy Cooperative Group (PTCOG) 2023 Committees

***PTCOG Executive and Organizing Committees***

**Marco Durante, Ph.D., PTCOG President**

**Martin Jermann, MSc, PTCOG Secretary**

**Alejandro Mazal, MSc, Ph.D.**, Director técnico y Jefe de Servicio de Física Médica, Centro de Protonterapia Quirónsalud, Madrid

**Felipe Calvo Manuel, MD, Ph.D.**, Director científico de la Unidad de Protonterapia del Cancer Center Clínica Universidad de Navarra.

***PTCOG61 Scientific Program Subcommittee Members***

**Michael Story, Ph.D.**

*Co-Chairman of Scientific Program Subcommittee, Biology*

**Joe Y. Chang, M.D.**

*Co-Chairman of Scientific Program Subcommittee, Clinics*

**Antony J. Lomax, Ph.D.**

*Co-Chairman of Scientific Program Subcommittee, Physics*

***PTCOG61 Educational Subcommittee Members***

**Hesham Gayar, M.D.**

*Co-Chairman of Education Subcommittee, Clinics*

**Hakan Nyström, Ph.D.**

*Co-Chairman of Education Subcommittee, Physics & Technology*

## Oral Abstracts

### O 001 - Multi-slit prompt-gamma camera for accurate in vivo beam range verification in pencil beam scanning proton therapy

#### Youngmo Ku^1^, Sehoon Choi^1^, Jaeho Cho^1^, Sehyun Jang^1^, Jong Hwi Jeong^2^, Sung Hun Kim^2^, Sungkoo Cho^3^, Chan Hyeong Kim^1^

##### ^1^Hanyang University, Department of Nuclear Engineering, Seoul, Korea Republic of ^2^National Cancer Center, Proton Therapy Center, Goyang-Si, Korea Republic of ^3^Samsung Medical Center, Department of Radiation Oncology, Seoul, Korea Republic of

Proton beam’s sharp dose fall-off at its range enables highly conformal dose delivery in proton therapy. This high conformality, however, has not reached its physical potential, mainly owing to beam range uncertainty, which is approximately 4% in clinical practice. To reduce range uncertainty by prompt gamma imaging, in the present study, a multi-slit prompt-gamma camera was developed, as shown in Figure 1. The developed camera is for clinical applications, featuring (1) a wide field of view camera (= 216 mm) achieved by a multiple-parallel-slit tungsten collimator and 144 CsI(Tl) detectors, (2) a six-dimensional automatic camera positioning trolley for arrangement of the camera in various clinical situations with high speed and accuracy, and (3) a high-precision beam range determination algorithm which locates the centroid of fall-off in prompt gamma depth distribution. Figure 2 shows the range measurement precision of the camera evaluated for spot beams of six different energies (99.68–150.62 MeV) and eight different numbers of protons (2 × 10^7^–5 × 10^9^) to a PMMA phantom. The precision hardly depended on the beam energy, whereas it significantly improved with the number of protons. When the number of protons was 1 × 10^8^, which is the typical number of protons in a spot, the precision reached ∼3%. For 1 × 10^9^ protons, which can be readily achieved by statistical spot aggregation, the precision was even less than 1%. The results of the present study assure that the range uncertainty of the proton beam in clinical practice can be significantly reduced by using the developed multi-slit prompt-gamma camera.

### O 002 - Proton range verification from protoacoustic signals with neural networks

#### Clara Freijo^1^, Javier García-Muñoz^1^, Daniel Sanchez-Parcerisa^1^, Jose Manuel Udias^1^, Joaquin Lopez Herraiz^1^

##### ^1^Complutense University of Madrid, Nuclear Physics Group. EMFTEL and IPARCOS, Madrid, Spain

Protoacoustics is a promising method for range verification in proton therapy, as the generated thermoacoustic pressure waves provide information on the deposited dose distribution in tissues. A neural network (ProtoNN) was developed to estimate the Bragg peak (BP) location from the measured protoacoustic signals, for each beamspot of the treatment plan. The goal of ProtoNN is to estimate BP locations in less than 100ms with a precision better than 1mm, and produce an alert if it deviates from the treatment plan. The training dataset comprised simulated acoustic waveforms derived from each of the spots in a single-field proton plan for a prostate tumor (Fig. 1a,b). Dose calculation was performed with the open-source treatment planning system matRad, while k-Wave was used for acoustic wave propagation in 3D. ProtoNN consisted of three fully-connected layers with a total of 1.5M parameters. Trained in 20min using 430 cases, it was able to identify the BP position of 140 (different) validation proton beamlets (Fig. 1c) with an average accuracy of 0.9 mm, taking 77ms per beamlet. Furthermore, ProtoNN was also able to detect deviations in the patient with respect to the planning-CT. As an example, the reference CT was modified inserting an air region (1.75x2.75x2.0 cm3) within the patient in the beam direction, creating large deviations in the estimated BP location with respect to the expected one (Fig. 1d) that were detected by ProtoNN, which emerges as a fast, flexible and promising proton range verification tool.

### O 003 - Compton camera performance tests as a treatment monitoring system in a Proton therapy center

#### Rita Viegas Rego^1^, Jorge Roser^1^, Luis Barrientos^1^, Marina Borja-Lloret^1^, Jose Vicente Casaña^1^, Fernando Hueso-González^1^, Javier Pérez-Curbelo^1^, Ana Ros^1^, Gabriela Llosá^1^

##### ^1^IFIC, Experimental Physics, Paterna, Spain

Two Compton cameras (CCs) for hadron therapy treatment monitoring are under development at the IRIS group of IFIC-Valencia, Spain. The prototype’s detection layers are composed by monolithic LaBr_3_ crystals coupled to SiPM arrays. The systems differ on the employed readout electronics: whereas MACACOIII uses the VATA64HDR16 ASIC from IDEAS, MACACOp uses the TOFPET2 ASIC, for improved timing resolution. Both CCs were fully characterized in the laboratory. In-beam tests were performed at the proton therapy center CCB in Krakow, Poland. The CCs were placed in opposite directions, perpendicular to an RW3 target (fig.1). Measurements were performed by irradiating the target with mono-energetic proton beams with 88.38MeV, 90MeV and 91.62MeV at a current of 60pA and a length of 10 minutes. This way, displacements of the Bragg peak of 2 and 4mm were generated. The 80% distal depth position of the recovered gamma-ray distribution (R80) of the reconstructed images’ longitudinal profiles (fig.2) was used to determine the separation of the recovered spots, at the expected 4.44 MeV PG energy. The values obtained for both systems are within the expected value, 0.89±1.4mm and 4.4±1.4mm with MACACOIII and 2.5±1.4mm and 4.2±1.4mm with MACACOp. The obtained results show the ability of the systems to detect 2 and 4mm variations in the PG emission distribution. Further work is aimed to reduce the uncertainties associated with the reconstruction method.

### O 004 - Labeling the tumor with 31-P, 63-Cu, and 89-Y provides an in vivo prompt gamma-based range verification for therapeutic protons

#### Giorgio Cartechini^1^, Elena Fogazzi^1^, Luna Pellegri^2^, Marie Vanstalle^3^, Chiara La Tessa^4^

##### ^1^University of Trento, Physics, Trento, Italy ^2^iThemba LABS, Physics, Cape Town, South Africa ^3^Université de Strasbourg, Physics, Strasbourg, France ^4^University of Miami, Radiation Oncology, Miami, USA

Range uncertainty is a main limitation to fully exploiting the benefits of proton therapy. Its reduction will improve treatment effectiveness by increasing both dose conformality in the tumor and normal tissue sparing. The methodology is based on the detection of prompt gammas (PG), whose production is artificially enhanced with a non-radioactive element transported selectively to the tumor with a drug carrier. Nuclear interactions of this element with protons generate a signature PG spectrum, from which both the absolute proton range and the tumor position can be reconstructed by exploiting the existing PG spectroscopy methods. Combining experimental data and calculations obtained both with TALYS and TOPAS Monte Carlo code, we selected three stable elements: ^31^P, ^63^Cu and ^89^Y. We then measured the PG energy spectra emitted by solutions of water and the candidate materials (CuSO_4_+H_2_O, NaH_2_PO_4_+H_2_O and Y(NO_3_)_3_+H_2_O) when exposed to clinical proton beams (Figure 1). From measurements, we evaluated that, at a realistic element concentration in the tumor of 0.4 mM, 10^9^ protons for an iso-energy slice, and an advanced detection system, the produced PG signature is large enough to be distinguished from the normal tissue background. In addition, using the ^31^P label on a simplified patient geometry, we experimentally proved that the proposed methodology predicts the absolute proton range with a 2 mm accuracy. Following the PG spectroscopy approach by *F Hueso-González et al. Phys. Med. Biol. (2018)*, we applied our methodology to a real patient geometry, investigating the correlation between proton beam spatial shifts and PG production variations.

### O 005 - Range monitoring in proton therapy using the J-PET scanner

#### Karol Brzezinski^1^, Jakub Baran^2^, Damian Borys^3^, Jan Gajewski^4^, Grzegorz Korcyl^2^, Szymon Niedźwiecki^2^, Paweł Moskal^2^, Antoni Rucinski^4^

##### ^1^Instituto de Física Corpuscular, CSIC - University of Valencia, Paterna, Spain ^2^Faculty of Physics- Astronomy- and Applied Computer Science- Jagiellonian University, Center for Theranostics- Jagiellonian University, Krakow, Poland ^3^Silesian University of Technology, Department of Systems Biology and Engineering, Gliwice, Poland ^4^Institute of Nuclear Physics, Polish Academy of Sciences, Krakow, Poland

The Jagiellonian positron emission tomography (J-PET) technology, based on plastic scintillators, has been proposed as a cost-effective tool for in-vivo range monitoring during proton therapy (Moskal et al., DOI: 10.1126/sciadv.abh4394). Full-ring and dual-head J-PET geometries have been simulated in in-room and in-beam protocols. Experiments have been performed to validate simulations. Single pencil beam (SPB) and spread-out Bragg peak (SOBP) irradiations were simulated in a uniform PMMA phantom to assess the performance of different scanner geometries in terms of sensitivity and precision of range measurements in homogeneous targets (Baran, Brzezinski et al. PMB under review). Detailed simulations of treatment plans for a large cohort of 90 patients who underwent IMPT at Cyclotron Centre Bronowice (CCB) were performed, with errors in patient positioning and HU-proton stopping power calibration artificially introduced (Brzezinski et al. PMB under review). For these studies, a dedicated Monte Carlo framework, ProTheRaMon, was developed (Borys, Brzezinski et al DOI:10.1088/1361-6560/ac944c). Experiments were performed at CCB with a dual-head J-PET geometry. Simulations showed that double-layer full-ring and triple-layer dual-head geometries are the most cost-effective in terms of efficiency. The simulated geometries showed a precision <1 mm in range measurements of SPB and SOBP irradiations. From the patient simulations, a correlation was observed between the standard deviation of proton range shift maps and those of shifts in activity as reconstructed with J-PET data (see Figure1). Preliminary experimental results have demonstrated the ability of the J-PET prototype to detect range differences of SOBPs with precision of about 1 mm (See figure 2).

### O 006 - Multi-institutional experience of proton therapy for Ewing sarcoma and rhabdomyosarcoma in the Proton Collaborative Group (PCG) prospective registry

#### Katherine Von Werne^1^, Keitrina Mair^1^, John Chang^2^, Wolden Suzanne^3^, Tamara Vern-Gross^4^, Stephen Mihalcik^5^, Young Kwok^6^, Henry Tsai^7^, Jing Zeng^8^, Matthew Hall^1^

##### ^1^Miami Cancer Institute- Baptist Health South Florida, Radiation Oncology, Miami, USA ^2^Oklahoma Proton Center, Radiation Oncology, Oklahoma City, USA ^3^New York Proton Center & Memorial Sloan Kettering Cancer Center, Radiation Oncology, New York, USA ^4^Mayo Clinic- Arizona, Radiation Oncology, Phoenix, USA ^5^Northwestern University, Radiation Oncology, Warrenville, USA ^6^University of Maryland, Radiation Oncology, Baltimore, USA ^7^Procure NJ, Radiation Oncology, Somerset, USA ^8^University of Washington, Radiation Oncology, Seattle, USA

**Purpose**: To report on outcomes and acute toxicities following proton therapy (PT) for patients with rhabdomyosarcoma and Ewing sarcoma in a prospective multi-institutional registry (PCG).

**Methods**: Data on patients with primary rhabdomyosarcoma and Ewing sarcoma treated with definitive PT were queried from the PCG registry. Toxicities were scored using CTCAE v4.0.

**Results**: Three hundred and eleven (311) patients across 10 institutions (181 rhabdomyosarcoma, 130 Ewing sarcoma) met the eligibility criteria. Median age was 9 years (Interquartile Range: 5-15). Median dose was 50.4 GyRBE for rhabdomyosarcoma patients (Range: 45-66 GyRBE) and 55.8 GyRBE for Ewing sarcoma patients (Range: 45-66 GyRBE). Median follow-up was 2.2 years (Range 0.2-12.3 years). Two-year overall survival rates were 85.1% for Ewing sarcoma and 80.8% for rhabdomyosarcoma. The figure illustrates the prescription doses delivered by tumor histology; 19.2% of Ewing sarcoma and 27.1% of rhabdomyosarcoma patients, respectively, received dose-escalated radiotherapy (defined as >55.8 Gy for Ewing sarcoma and >50.4 Gy for rhabdomyosarcoma). Excluding alopecia and skin desquamation, 143 patients (46.0%) developed any acute grade 2+ non-hematologic toxicity, while 44 patients (14.1%) developed grade 3 toxicities, as shown in the table. Only one grade 4 toxicity (esophagitis) was reported.

**Conclusions**: In this multi-institutional prospective registry, 19.2% of Ewing sarcoma and 27.1% of rhabdomyosarcoma patients received dose-escalated PT, with 14.1% of patients experiencing any grade 3 toxicity. Long-term outcomes for disease control and late toxicity and anticipated cooperative group trial results are needed to fully assess the benefits and risks of dose-escalated radiotherapy for these tumors.

### O 007 - Investigating the potential relation between dose rate and phantosmia in pediatric patients receiving crania-spinal irradiation with PBS proton therapy

#### Arthur Lalonde^1^, Thomas Madden^2^, Myrsini Ioakeim-Ioannidou^2^, MacDonald Shannon^2^, Juliane Daartz^2^

##### ^1^CHUM, Radiation Oncology, Montreal, Canada ^2^Massachusetts General Hospital, Radiation Oncology, Boston, USA

**Purpose**: Reports of malodorous phantom smell (phantosmia) by pediatric patients receiving cranio-spinal irradiation (CSI) with proton beams have significantly increased at our institution after transitioning from passive scattering to pencil beam scanning (PBS) delivery. The purpose of this work is to evaluate if the dose rate (DR) to the olfactory region might be correlated with the incidence of phantosmia.

**Methods**: Dose and DR distributions from five pediatric patients who reported phantosmia during CSI with PBS were compared to those of five similar patients that did no report such symptoms. DR distributions were generated using an in-house developed DR calculation tool reporting the maximum time-averaged DR to each voxel for any time intervals of 10 ms, 100 ms and 1 s. The DR and total dose to the olfactory region was evaluated across eight dose-volume histograms (DVH) and DR volume histogram (DRVH) points.

**Results**: No statistically significant differences between symptomatic and asymptomatic patients were observed on the DVH points of interest. The DR to the nasal cavity at 10 ms and 100 ms time intervals was however substantially higher in the symptomatic cohort. Statistically significant differences were observed in the DR to 50% of the nasal cavity, with a mean value of 164.67 Gy/min (sd=41.39 Gy/min) for patients that reported phantosmia compared to 108.65 Gy/min (sd=47.98 Gy/min) for patients that did not report unusual olfactory symptoms (p=0.0411).

**Conclusion**: In this patient cohort, phantosmia experienced during treatment delivery was correlated with the peak DR to the nasal cavity during short time intervals.

### O 008 - Clinical outcome comparison between adolescents and young adults and adults with uveal melanoma by propensity score matching

#### Alessia Pica^1^, Damien Weber^1^, Claude Schweizer^2^, Aziz Chaouch^3^, Ann Schalenbourg^2^

##### ^1^Paul Scherrer Institut, Proton Therapy Center, Villigen, Switzerland ^2^Ophthalmic Hospital Jules Gonin, Adult Ocular Oncology, Lausanne, Switzerland ^3^Centre Universitaire de médecine générale et santé publique, Epidémiologie et systèmes de santé, Lausanne, Switzerland

**Purpose**: Adolescent and young adult (AYA) uveal melanoma (UM) occurs in patients below 40 years old. The purpose of this study is to compare the outcomes of AYA patients and older patients after proton therapy (PT).

**Methods**: A retrospective, comparative study was conducted in patients who underwent proton therapy for UM at at the Ocular Oncology Unit of the Jules-Gonin Eye Hospital and the Paul Scherrer Institute (PSI) Villigen, between January 1997 and December 2007 .They were divided into AYA group and older group based on age (<40, AYA group; ≥ 40, older group). Overall survival in the two groups was estimated using Kaplan-Meyer curves. The difference between these curves was tested using logrank test adapted to clustered data. Propensity score matching (PSM) was used to select adult patients (i.e. ≥ 40 years old) with similar characteristics as the AYAs.

**Results**: We identified 272 AYA and 1’979 adult patients. Before PSM, the AYAs have a higher incidence of primary iris melanoma (4.0% vs. 1.4%; p=0.003) than the older patients. The estimated 5-year year OS in AYA group and older group was not different (P=0.6). Cumulative incidence of metastasis for the AYA group was not different between the two groups (P=0.214). However, according to the relative survival (RS) estimation, AYA group has a worse RS than the older group (P=0.002).

**Conclusions**: UM might be more aggressive in AYAs, and the relative survival after PT is worse than in older patients.

### O 009 - Long-term pediatric outcomes from the world’s first single-vault compact proton beam accelerator

#### Sabrina Prime^1^, Douglas Caruthers^1^, Tianyu Zhao^1^, Stephanie Perkins^1^

##### ^1^Washington University School of Medicine, Department of Radiation Oncology, Saint Louis, USA

**Purpose**: To evaluate the experience and outcomes of pediatric patients (≤21 years) treated with proton beam therapy (PBT) on the world’s first single-vault synchrocyclotron proton machine.

**Patients and Methods**: Pediatric patients were treated with passive scatter technology (2014-mid-2020) or pencil beam scanning (mid-2020 to date). Patient demographics, diagnoses, disease-specific and overall outcomes were collected and summarized. Kaplan-Meier curves were generated to review site- and disease-specific outcomes.

**Results**: A total of 221 pediatric patients were treated with PBT, comprising approximately 20% of patient volume at our center. Median follow-up time was 3.5 years. CNS tumors comprised 70% of diagnoses, of which 46% received craniospinal irradiation. Three- and 5-year overall survival for all pediatric patients was 86% and 83% respectively. CNS patients fared better, with 3- and 5-year overall survivals of 88% and 87%, compared to patients treated at non-CNS sites (80% and 73%), largely driven by poorer outcomes in metastatic sarcomas. There were no cases of brainstem toxicity. Despite use of audio-visual technology and presence of child life therapy throughout treatment, 41% of children required sedation for treatment. No secondary malignancies had developed at time of analysis, though 3 benign in-field lesions were identified.

**Conclusions**: At a large academic medical center, a single-room proton therapy center experienced 20% of patient volume attributed to pediatric patients. Craniospinal irradiation and daily sedation were required for 46% and 41% of patients, respectively. Outcome data demonstrated excellent overall survival, especially for CNS tumors.

### O 010 - Optic survey 2022: Practices and trends in ocular proton and ion therapy

#### Linda Mortimer^1^, Roelf Slopsema^2^, Alejandro Mazal^3^

##### ^1^The Clatterbridge Cancer Centre, Medical Physics, Wirral, United Kingdom ^2^Emory University, Department of Radiation Oncology, Atlanta, USA ^3^Quironsalud Proton Therapy Centre, Medical Physics, Madrid, Spain

The purpose of this survey was to provide an updated overview for practices and technology in ocular proton therapy (OPT). The survey was completed by 13 operational centers and 4 with plans to become operational (17 in total). It comprised 127 questions on topics including simulation & planning, patient immobilization and alignment, therapy system hardware, dosimetry, quality assurance and future plans. Almost 38,000 patients were treated by the 13 operational centers by end of 2021. Diagnoses and prescriptions were consistent between centers. Similarities in practice were observed for immobilization, eyelid retraction and online monitoring methods, but differences were noted in chair, X-ray hardware, image acquisition and position verification systems. Just over half of centers (54%) used a common ocular TPS, while the remainder used one of five alternative systems. Nine (69%) operational centers used MRI imaging and eight (62%) used CT for treatment planning. A large variation was observed in therapy system hardware, reflected in differences in quality assurance programmes, including frequency of checks of individual parameters and time spent on daily QA. All centers followed the same dosimetry Code of Practice (IAEA TRS-398). A comparison of the current and predicted status of ocular facilities, indicated a shift towards high-energy degraded beams, the use of PBS systems with adapted nozzles. TPS development was ranked as being the most important to improve ocular particle therapy, followed by MRI imaging for target delineation, development of QA devices and methods specific to eye therapy, and increased access to OPT.

### O 011 - New image reconstruction approach for in-vivo range verification of multiple field carbon ion treatments by means of in-beam PET system

#### Veronica Ferrero^1^, Davide Bersani^2^, Pietro Carra^2^, Piergiorgio Cerello^1^, Martina Moglioni^3^, Matteo Morrocchi^3^, Francesco Pennazio^1^, Giacomo Traini^4^, Viviana Vitolo^5^, Elisa Fiorina^1^

##### ^1^INFN, sez. Torino, Torino, Italy ^2^INFN, sez. Pisa, Pisa, Italy ^3^University of Pisa and INFN, Physics Dep., Pisa, Italy ^4^INFN, sez. Roma1, Roma, Italy ^5^Fondazione CNAO, Clinical Dep., Pavia, Italy

Effective in-vivo range verification is still an open issue to pursue innovation in particle therapy. Positron Emission Tomography (PET), relying on beam-tissue interactions, has been investigated in clinics, mainly for protons. In-beam PET systems acquire data during field irradiations, without slowing down the clinical workflow. At the CNAO, the INSIDE bimodal system (fig.1a) is under clinical monitoring in total 40 patients (ClinicalTrials.gov NCT03662373). It features an in-beam PET and a Dose Profiler, a secondary particle tracker designed for carbon ions. For carbon ions, the positron emitter production is severely reduced (at least 10x with respect to protons) and mainly 11C is produced. Due to its long half-life (20 min), few annihilation signals can be collected during each field acquisition. On the other hand, since 11C isotopes are primarily produced by projectile fragmentation, most of the positron emitter decays are in the Clinical Target Volume. Therefore, we propose a new method exploiting this key element to increase in-vivo range verification reliability. It allows to consider multiple field irradiations in a single PET image gaining statistical significance. This method is based on the Maximum Likelihood Expectation Maximization (MLEM) approach (fig.1b). A virtual detector, covering all the possible positions of the real detectors, is introduced to identify in a single space all annihilation events. Moreover, a patient-tailored MLEM sensitivity is calculated to carefully consider each field contribution. The proposed approach was validated with Monte Carlo simulations and tested on the INSIDE in-vivo data (fig.2). Results will be compared with the Dose Profiler measurements.

### O 012 - Imaging of proton beams with a novel spherical in-beam PET scanner for range monitoring in small animal irradiation research

#### Francesco Evangelista^1^, Munetaka Nitta^1^, Giulio Lovatti^1^, Ze Huang^1^, Marco Pinto^1^, Jonathan Bortfeldt^1^, Taiga Yamaya^2^, Mateusz Sitarz^3^, Peter Thirolf^1^, Katia Parodi^1^

##### ^1^Ludwig-Maximilians-Universität LMU, Medical Physics, Garching, Germany ^2^National Institutes for Quantum Science and Technology QST, Institute for Quantum Medical Science, Chiba, Japan ^3^Danish Centre for Particle Therapy DCPT, Clinical Medicine, Aarhus, Denmark

The SIRMIO (Small animal proton Irradiator for Research in Molecular Image-guided radiation-Oncology) project aims at developing an image-guided platform to be used at existing clinical facilities for precision proton irradiation of small animals (Parodi et al 2019). In a thorough experimental campaign at the Danish Centre for Particle Therapy (DCPT) in Aarhus the functionality of the Positron Emission Tomography (PET) scanner in a realistic beam scenario was tested. The scanner is composed of 56 scintillation detectors arranged on a sphere with a 72 mm inner diameter. Each detector consists of 3 layers of LYSO crystals (0.9 × 0.9 × 6.67 mm^3^) which provide depth-of-interaction information. The detectors are developed at LMU in collaboration with QST (Nitta et al, NSS/MIC, 2021). For the experiment, a cylindrical PMMA target was placed inside the PET scanner and then exposed to a clinical proton beam of 70 MeV, which was modulated by the SIRMIO beamline [Fig.1]. Three sets of irradiation were carried out, each of which lasted ∼5 minutes and with energies of 30 MeV, 40 MeV and 50 MeV, respectively, at the target entrance. The spatial distribution of the imaged radioactivity in the first 10 minutes after beam-off was reconstructed with a Maximum-Likelihood-Expectation-Maximization iterative algorithm. The first results show the capability of the PET system to visualize the beam position [Fig.2]. Improved images including corrections, e.g., for sensitivity will be presented and compared to simulation predictions. This work is supported by ERC Grant 725539 (SIRMIO) and EU Grant 730983 (INSPIRE). We thank the entire SIRMIO team, the DCPT team and collaborators.

### O 013 - Intra and inter field PET imaging for treatment verification of proton therapy with the PETITION detector

#### Keegan McNamara^1,2^, Marina Beguin^2^, Günther Dissertori^2^, Judith Flock^2^, Cristian Fuentes^2^, Antony J. Lomax^1,2^, Shubhangi Makkar^1,2^, Christian Ritzer^2^, Damien C. Weber^1,3,4^, Carla Winterhalter^1^

##### ^1^Paul Scherrer Institut, Center for Proton Therapy, Villigen, Switzerland ^2^ETH Zürich, Department of Physics, Zürich, Switzerland ^3^Inselspital- Bern University Hospital- University of Bern, Department of Radiation Oncology, Bern, Switzerland ^4^University Hospital of Zürich, Department of Radiation Oncology, Zürich, Switzerland

Treatment verification of proton-therapy using PET detectors provides the unique possibility to gain both in-beam feedback of treatment by imaging short-lived isotopes during treatment (intra-field), potentially resolving the range of individual pencil beams, and post irradiation 3D PET images of patients following delivery of each field, allowing validation of the total field delivered within the patient anatomy (inter-field). We present the combination of these two regimes using the PETITION (PET for intensive care and innovative proton therapy) detector for a simulated patient case consisting of 3 treatment fields. The open-ring design allows for recording of coincidences between delivery of energy layers (100ms), giving 2D images of short-lived isotopes such as ^12^N (t_1/2_=11ms). Intra-field imaging shows the progression of the distal edge of the field through projection of coincidences along the beam axis (Fig. 1) with the 50% cumulative dose fall-off being correlated to the 50% activity fall-off (R^2^=0.947). Rotation of the scanner between delivery of each field to the new imaging position reduces the 3D distortion in the reconstructed images, and a new iterative reconstruction technique was implemented to combine multiple scanner positions for higher image quality. Inter-field 3D reconstruction is shown in Fig. 2. The new rotating reconstruction leads to a 54-70% reduction in the mean-absolute-error when compared to reconstruction using a fixed scanner position. The proposed combined intra/inter-field protocol provides the improved spatial resolution of existing in-room protocols using commercial full-ring scanners, with the prompt PET imaging capabilities of existing in-beam dual head scanners.

### O 014 - Road to intra-fractionation adaptive proton therapy with online PET image-based beam range guidance

#### Dongxu Yang^1^, Xiaorong Zhu^2^, Mingli Chen^1^, Lin Ma^3^, David Grosshans^4^, Lu Weiguo^1^, Yiping Shao^1^

##### ^1^UT Southwestern Medical Center, Radiation Oncology, Dallas, USA ^2^University of Texas MD Anderson Cancer Center, Radiation Physics, Houston, USA ^3^University of Pennsylvania medical school, Radiation Oncology, Philadephia, USA ^4^University of Texas MD Anderson Cancer Center, Radiation Oncology, Houston, USA

This study aims to measure and compensate the proton beam range shift (away from the planned value) within a treatment fractionation for achieving accurate proton therapy. A newly developed PET suited for acquisition during radiation consists of 20 detector panels in a polygon configuration with ∼3mm spatial resolution, ∼32cm diameter and 6.4cm axial field-of-view, and two removable detector panels for beam passing. A head phantom was placed inside PET. A fraction of planned therapeutic scanning beams (referring as probing beams) with selected doses and layers irradiated the phantom and immediately followed by only a 60-second PET acquisition for beam range measurement. Undershot or overshot range-shift was introduced by adding or removing a ∼6.8mm thick Lucite sheet between the phantom and beam nozzle. If measured range-shift exceeds the defined margin, delivery of the rest beams can be adjusted online to compensate the range-shift so that the fully delivered dose distribution will match to the planned one. Results show for probing beams with 3 or more adjacent layers (5.5 to 8.7MU), PET can measure sufficiently accurate range-shift with <1.0mm deviation from that measured by radiated Gafchromic films. With the corresponding range-shift compensation, the gamma index passing rate between fully delivered and planned dose distributions was ∼98%-99%, with 3%, 3mm criteria and calculated over a 5x5x5 cm^3^ region-of-interest, which is significantly better than ∼73%-81% when measured without range-shift compensation. Overall, this study demonstrated the feasibility of intra-fractionation adaptive proton therapy with ∼60-second PET acquisition and possible <60-second range-shift measurement and adjustment of remaining beam delivery.

### O 015 - On-the-fly reconstruction of activation by proton beams using in-beam PET

#### Andrea Espinosa Rodríguez^1,1^, Fernando Arias-Valcayo^1^, Victor V. Onecha^1^, Paula Ibañez^1^, Samuel España^1^, Daniel Sanchez-Parcerisa^1^, Juan Antonio Vera-Sanchez^2^, Alejandro Mazal^2^, Luis Mario Fraile^1^, Jose Manuel Udias^1^

##### ^1^Grupo de Física Nuclear- EMFTEL & IPARCOS, Complutense University of Madrid- CEI Moncloa, Madrid, Spain ^2^Centro de Protonterapia, Quironsalud, Madrid, Spain

**Introduction**: In-beam PET is attractive in terms of feedback-time of treatment quality in protontherapy. However, it is challenged by the high rates produced during the beam-on period. In this work, we report the first results using a novel in-beam portable PET system that can detect and process on-the-fly the β^+^ activity produced during and after irradiation.

**Methods**: The specific PET setup consisted of 6 phoswich detector blocks with 338 pixels each, with of 1.55×1.55 × LYSO (7mm)+GSO (8mm). The system was coupled to a fast data acquisition system able to sustain rates up to 10 Msingles/sec. Two different PMMA targets were irradiated with mono-energetic clinical proton beams at the Quirónsalud proton therapy center (Figure 1).

**Results**: The radionuclide-specific contribution (^11^C, ^15^O, ^10^C) was obtained from the time-activity curves corresponding to the irradiated region. 3D maps of the activity were reconstructed on-the-fly every 0.5 seconds and with a 0.5 mm spatial resolution (Figure 2). We also assessed the system response to changes in the position and direction of the beam during irradiation.

**Conclusion**: This validates the experimental setup to be used for in-beam on-the-fly reconstruction of the 3D activity when irradiating with proton beams and provides a gold standard to obtain the deposited dose distribution when combined with a fast dose reconstruction method.

### O 016 - Protons for primary spinal tumors: Consensus statement from the PTCOG Skull Base/CNS/Sarcoma subcommittee on simulation, treatment planning and delivery

#### Arpit Chhabra^1^, Markus Stock^2^, Mark McDonald^3^, Heng Li^4^, Janes W. Snider^5^, Adam Kole^6^, Robert Press^7^, Michael H. Soike^6^, Jun Zhou^3^, Rex Carden^6^, Mark Mishra^8^, Bree Eaton^3^, Pouya Sabouri^9^, Francesca Albertini^10^, Chengyu Shi^11^, Haibo Lin^12^, Sina Mossahebi^8^, Jake Wang^13^, Rovel Colaco^14^, Charles B. Simone II^12^

##### ^1^New York Proton Center, Department of Radiation Oncology, NY, USA ^2^EBG MedAustron, Department of Radiation Oncology, Austria, Austria ^3^Emory University, Department of Radiation Oncology, Atlanta, USA ^4^Johns Hopkins University, Department of Radiation Oncology, Baltimore, USA ^5^South Florida Proton Therapy Institute, Department of Radiation Oncology, Delray Beach, USA ^6^University of Alabama, Department of Radiation Oncology, Birmingham, USA ^7^Miami Cancer Institute - Baptist Health, Department of Radiation Oncology, Miami, USA ^8^University of Maryland, Department of Radiation Oncology, Baltimore, USA ^9^University of Arkansas, Department of Radiation Oncology, Little Rock, USA ^10^Paul Scherrer Institute, Department of Radiation Oncology, Villigen, Switzerland ^11^City of Hope, Department of Radiation Oncology, Irvine, USA ^12^New York Proton Center, Department of Radiation Oncology, New York, USA ^13^Willis-Knighton Cancer Center, Department of Radiation Oncology, Shreveport, USA ^14^The Christie NHS Foundation Trust, Department of Radiation Oncology, Manchester, USA

**Purpose/Objectives**: Proton beam therapy (PBT) plays an important role in the management of primary spine tumors. The purpose of this guideline is to summarize safe and optimal delivery of PBT for spinal tumors.

**Materials and Methods**: The Particle Therapy Co-Operative Group (PTCOG) Skull Base/CNS/Sarcoma Subcommittee consisting of radiation oncologists and medical physicists with specific expertise in spinal irradiation developed expert consensus recommendations discussing treatment planning considerations and current approaches in the treatment of primary spinal tumors.

**Conclusions**: The PTCOG Skull Base/CNS/Sarcoma Subcommittee has developed recommendations to enable centers to deliver PBT safely and effectively for the management of primary spinal tumors.

### O 017 - A blinded clinical study: Deep learning plan quality equals manual plan quality for oropharyngeal carcinoma patients

#### Ilse Van Bruggen^1^, Marije van Dijk^1^, Minke Brinkman-Akker^1^, Fredrik Löfman^2^, Stefan Both^1^, Johannes Langendijk^1^, Erik Korevaar^1^

##### ^1^UMCG, Radiotherapy, Groningen, Netherlands ^2^RaySearch Laboratories, Machine learning, Stockholm, Sweden

**Objective**: The aim of this study was to develop and implement deep learning based automated robust IMPT planning for oropharyngeal carcinoma (OPC) patients and to compare the results with manual planning.

**Method**: IMPT dose distributions were predicted using a deep learning optimization (DLO) model which was trained using contours and doses of 60 OPC patients. A robust mimicking optimization algorithm using voxel-based mimicking and 21 perturbed scenarios was then used to generate a machine deliverable plan from the predicted dose distributions. A radiation therapy technologist (RTT) was allowed to further post-process the plan for up to two hours, e.g., add objectives, change weights of objectives and/or continue optimization. DLO plans were compared with manual plans in two blinded studies: a retrospective (n=10) and a prospective (n=8). All plans were evaluated in a multidisciplinary meeting with at least one physician, one physicist and one RTT (table 1).

**Results**: The DLO plans were preferred over manual plans in 10/10 patients in the retrospective study and in 5/8 patients in the prospective study (table 1). Differences between manual and DLO plans are limited (figure 1). A deliverable DLO IMPT plan was generated within 2.5 hours, 0.5 offline time followed by 2 hours post-processing time. Manual plans were generated in 8 to 16 hours.

**Conclusion**: DLO planning with manual post-processing can replace manual planning for OPC patients and reduces planning time.

### O 018 - Dosiomics-based survival analysis to predict local recurrence in skull base chordomas treated with carbon ion radiotherapy

#### Giovanni Parrella^1^, Simone Annunziata^1^, Letizia Morelli^1^, Silvia Molinelli^2^, Giuseppe Magro^2^, Giulia Riva^3^, Alberto Iannalfi^3^, Chiara Paganelli^1^, Ester Orlandi^4^, Guido Baroni^1^

##### ^1^Politecnico di Milano, Department of Electronics- Information and Bioengineering, Milano, Italy ^2^Centro Nazionale di Adroterapia Oncologica, Medical physics unit, Pavia, Italy ^3^Centro Nazionale di Adroterapia Oncologica, Radiotherapy unit, Pavia, Italy ^4^Centro Nazionale di Adroterapia Oncologica, Clinical unit, Pavia, Italy

**Purpose**: To apply a dosiomics approach to physical, RBE-weighted dose (LEM-I model) and dose-averaged Linear Energy Transfer (LET_d_) maps, to identify possible prognostic factors for local recurrence (LR) in Skull base chordomas (SBC) treated with Carbon Ion Radiotherapy (CIRT).

**Methods**: Data from 54 SBC patients were retrospectively selected at the National Centre for Oncological Hadrontherapy (CNAO, Italy). After a median follow-up time of 61.7 months, LR was identified in 18 patients, against 36 recurrence-free control patients. Cox proportional hazard models regularized with an elastic-net penalty (r-Cox) and Survival Support Vector Machine (s-SVM) were fed with dosiomics features extracted from LET_d_, physical and RBE-weighted dose (D_PHYS_ and D_LEM_) maps and tuned through a repeated 5-fold cross validation (Figure 1). Patients were then stratified in low/high risk of showing a LR. Models’ performance was evaluated in terms of Uno’s censoring-adjusted C-statistic (UnoC), and Kaplan-Meier (KM) survival curves through log-rank tests (α=0.05).

**Results**: The best performance was achieved with s-SVM built with LET_d_ dosiomics features, reaching a UnoC of 0.84 and statistically significant KM curves (p-value=0.0215, Figure 2), with D_PHYS_ and D_LEM_ models showing weaker results (0.74, 0.62 respectively) and non-significantly separated KM curves. The same trend was obtained with the r-Cox model (i.e. 0.84, 0.74, 0.60 for LET_d_, D_PHYS_, D_LEM_ respectively), but no significant KM curve was highlighted in this scenario.

**Conclusion**: LET_d_ maps shows a great potential as a source of prognostic factors for SBC in CIRT, and dosiomics appears to be promising in supporting patients’ stratification and outcome prediction during treatment planning.

### O 019 - Prediction of plan adaptation in head and neck cancer proton therapy using clinical, radiomic, and dosimetric features

#### Duncan Bohannon^1^, James Janopaul-Naylor^1^, Sagar Patel^1^, Chaoqiong Ma^1^, Chih-Wei Chang^1^, Yinan Wang^1^, Xiaofeng Yang^1^, Mark Mcdonald^1^, Jun Zhou^1^

##### ^1^Emory University, Radiation Oncology, Atlanta, USA

**Introduction**: Head and neck (HN) proton therapy is sensitive to anatomy changes like weight loss. At our institution, this leads to a 35% re-plan rate. This project aims to use a support vector machine (SVM) trained with patients’ pre-treatment clinical, radiomic, and dosimetric features to predict need for re-plan.

**Materials and Methods**: Data were gathered from 173 HN patients. These include tumor stage, site, robustness, and beam dose heterogeneity (BHI), defined as the ratio of the maximum beam dose to the CTV to half of the prescription. Robustness features include the fraction of all scenarios where V100>95%, mean V100, and mean max dose change from nominal plan. Radiomic features were gathered from the CTV using pyradiomics. First, second, and third order features were calculated, and all available filters were applied. A SVM was trained with all these features and an 80/20% split for training/testing was used. Due to highly nonlinear data, a radial basis kernel was used. Hyperparameter optimization fine tuned the SVM. A project pipeline is shown in Figure 1.

**Results**: The SVM had a sensitivity and specificity of 98.7% and 81.4% respectively with an area under the curve (AUC) for its receiver operator characteristic curve of 0.92 on the training data. The testing data had sensitivity, specificity, and AUC of 90.0%, 65.0%, and 0.82 respectively. ROCs are shown in Figure 2.

**Conclusion**: Our HN re-plan prediction model has high predictive power, allowing planners to evaluate the risk of a re-plan before treatment. To improve our model, features from dosiomics will be included in the future.

### O 020 - Accounting for the inter-fractional positional variability of the femoral heads in prostate cancer proton therapy

#### Edgar Gelover^1^, Serdar Charyyev^2^, Alex Stanforth^1^, Mingyao Zhu^1^, Haijian Chen^1^, Roelf Slopsema^1^, Katja Langen^1^, Stella Flampouri^1^

##### ^1^Winship Cancer Institute of Emory University, Radiation Oncology, Atlanta, USA ^2^Stanford University, Radiation Oncology, Stanford, USA

**Purpose**: In this work, a novel treatment planning technique for prostate cancer using multi-CT optimization is compared to standard planning approaches.

**Materials and Methods**: 22 prostate cancer patients with surgically implanted fiducials were included in this study. Three different intensity modulated proton therapy plans were generated: a) a PTV based plan, b) a robustly optimized (RO) plan using 5 mm setup and 3.5% range uncertainties (3DRO) and, c) a plan that in combination with typical uncertainties, uses four additional CT data sets artificially generated through deformable registration that simulate the movement of the femoral heads (FH) (4DRO). The FH displacement magnitude was derived by extracting the relative distance of the prostate/FHs using the centroid of the implanted fiducials on kV orthogonal images from 38 previously treated patients, equivalent to 954 treatment fractions. Robustness parameters and plan quality metrics were obtained from the nominal and QACT datasets of each patient.

**Results**: The FH displacement analysis yielded 0.6 and 0.7 cm in the sup-inf and ant-post directions, respectively (95% percentile). The 3DRO and 4DRO plans showed improved conformity and reduced integral dose compared to the PTV based plans. 4DRO plans showed enhanced robustness in both nominal and QACT data sets. Moreover, PTV plans are more susceptible to the development of hotspots.

**Conclusions**: Our findings indicate that, compared to PTV-based plans, 4D robustly optimized dose distributions offer advantages regarding plan quality and robustness. Moderate, yet, statistically significant differences were found between the 3DRO and 4DRO plans.

### O 021 - Heart, breast, thyroid doses on high-risk pediatric Children’s Oncology Group Hodgkin lymphoma trial by RT modality

#### Bradford Hoppe^1^, Hitesh Dama^2^, Anne Marie Charpentier^3^, Raymond Mailhot^4^, Kenneth roberts^5^, Rahul parikh^6^, Frank Keller^7^, Kara Kelly^8^, Sharon Castellino^7^, David Hodgson^2^

##### ^1^Mayo Clinic, Radiation Oncology, Jacksonville, USA ^2^Princess Margaret Cancer Centre, Radiation Oncology, Toronto, Canada ^3^University of Montreal, Radiation Oncology, Montreal, Canada ^4^University of Florida, Radiation Oncology, Jacksonville, USA ^5^Yale University, Radiation Oncology, New Haven, USA ^6^Rutgers, Radiation Oncology, New Brunswick, USA ^7^Emory University, Pediatrics, Atlanta, USA ^8^University of Buffalo, Pediatrics, Buffalo, USA

**Purpose**: We investigated the impact on organ at risk (OAR) doses when delivering ISRT using 3D-conformal radiotherapy (3D), intensity modulated radiotherapy (IMRT), and proton therapy (PT) to thoracic sites on the Children’s Oncology Group trial AHOD1331 (NCT021664643) for high risk classic Hodgkin lymphoma (cHL).

**Methods**: This multicenter randomized, open-label phase 3 study enrolled patients 2-21 years evaluating the role of brentuximab vedotin in pediatric cHL. ISRT was given to bulky mediastinal adenopathy and slow responding lesions. RT plans were normalized to 21 Gy and doses to the thyroid, breast, and heart were evaluated for the modalities.

**Results**: Radiation plans were available with doses to the OARs in 82, 126, and 74 patients for 3D, IMRT, and PT, respectively. The average mean heart dose was 9.9Gy, 10.5Gy, and 7.4Gy with 3D, IMRT, and PT, where PT was significantly lower than IMRT and 3D (P<0.001). The average mean breast dose was 2.9Gy, 5.5Gy, and 2.2Gy, where PT was significantly lower than IMRT (p=0.00001) and 3D (p=0.032), while IMRT was significantly higher than 3D (p=0.00001). The average mean thyroid dose was 11.6Gy, 11.5Gy, and 13.8Gy with 3D, IMRT, and PT, where PT was statistically significantly higher than IMRT (p=0.017) and 3D (p=0.0377).

**Conclusions**: As observed on AHOD1331, patients treated with PT had significantly lower doses to the heart and breast, but higher doses to the thyroid compared with 3D and IMRT. Future work should investigate whether replanning IMRT patients with PT leads to similar reductions in the dose to the OARs.

### O 022 - Proton therapy reduces lymphopenia in CNS glioma patients compared to intensity modulated photon radiotherapy

#### Anindita Das^1^, Sylvia Jacinthlyn^1^, Preethi Subramanyam^1^, Ganapathy Krishnan^2^, Pankaj Kumar Panda^3^, Roopesh Kumar^4^, Adhithyan Rajendran^5^, Sushama Patil^6^, Dayananda Sharma^2^, Rakesh Jalali^1^

##### ^1^Apollo Proton Cancer Centre, Neuro Oncology Cancer Management Team- Department of Radiation Oncology, Chennai, India ^2^Apollo Proton Cancer Centre, Department of Medical Physics, Chennai, India ^3^Apollo Proton Cancer Centre, Department of Clinical Research, Chennai, India ^4^Apollo Proton Cancer Centre, Neuro Oncology Cancer Management Team- Department of Neurosurgery, Chennai, India ^5^Apollo Proton Cancer Centre, Neuro Oncology Cancer Management Team- Department of Diagnostic & Intervention Radiology, Chennai, India ^6^Apollo Proton Cancer Centre, Neuro Oncology Cancer Management Team- Department of Pathology, Chennai, India

**Background**: Maximal safe resection followed by adjuvant radiotherapy (RT) and chemotherapy is the standard of care in Grade 2-4 gliomas. Radiation-induced lymphopenia (RIL) is shown to possibly affect treatment outcomes adversely. Proton beam therapy (PBT) may reduce volume of normal brain receiving moderate radiation doses- an important predictor of RIL. We aimed to evaluate the incidence and severity of RIL during proton beam therapy (PBT), and compare it against photon-based RT (XRT).

**Methods**: We identified Grade 2-4 glioma patients treated with PBT and XRT at our centre between Jan 2019 to Dec 2021. We compared the incidence and severity of RIL from weekly complete blood count (CBC) data prospectively collected during RT, and whole brain volume receiving 20 Gy and 25 Gy between these modalities.

**Results**: The incidence of any degree of lymphopenia (48% in PBT, vs. 81.2% in XRT) and severe lymphopenia (8% in PBT, vs. 24.6% in XRT) were both significantly lesser in patients who received PBT. Severe RIL after receiving PBT was only seen in CNS WHO Gr- 4 tumours. Mean whole brain V20GyE and V25 GyE were significantly lower in the PBT patients than XRT patients. Whole brain V20GyE and V25GyE inversely correlated to the nadir ALC. Patients with lymphopenia showed a trend towards poorer progression-free survival (p=0.06).

**Conclusion**: Proton therapy seems to have a superior sparing of normal brain to moderate dose radiation than photon-based RT, and reduces incidence of lymphopenia. Glioma patients with lymphopenia possibly have worse outcomes than the ones with maintained lymphocyte counts.

### O 023 - Hypo-fractionated carbon-ion radiotherapy (CIRT) for high-risk prostate cancer: The MedAustron approach

#### Razvan Galalae^1^, Ankita Nachankar^2,3^, Eugen Hug^4^, Ulrike Mock^4^, Antonio Carlino^5^, Piero Fossati^4,6^

##### ^1^Klinik für Radio-Onkologie- Klinikum Bremerhaven- Germany, Department of Radiation Oncology, Bremerhaven, Germany ^2^MedAustron, Department of Radiation Oncology, Wiener Neustadt, Austria ^3^ACMIT- Austria, Medical Research and Development, Wiener Neustadt, Austria ^4^MedAustron Ion Therapy Center- Austria, Department of Radiation Oncology, Wiener Neustadt, Austria ^5^MedAustron Ion Therapy Center- Austria, Medical Physics, Wiener Neustadt, Austria ^6^Klinik für Radio-Onkologie- Klinikum Bremerhaven- Germany, Department of Radiation Oncology, Krems an der Donau, Austria

**Purpose**: Photon-based external beam radiotherapy(p-EBRT) is an established treatment option in all risk-strata of localized prostate cancer and reported results are excellent both in terms of outcome and toxicity. Carbon-ion radiotherapy(CIRT) could potentially have an advantage further reducing the risk of toxicity and improving the outcome in High risk prostate cancer (HR-PC) as suggested by the JCROS(Japanese working group for carbon-ion) reported data in 2016. Here we present our technique and preliminary results of CIRT in HR-PC. Dose and fractionation is derived from Japanese experience corrected to account for the different RBE systems, moreover we included elective node irradiation (ENI) for HR-PC.

**Material and Methods**: Twenty-six patients with HR-PC (T-stage>/=cT2c or PSA>=20ng/mL or Gleason-score>7) treated with neoadjuvant hormone-therapy from November 2020-November 2022 were prescribed CIRT to dosages of 57.6-68.8Gy RBE/12-16fractions (based on urinary-dysfunction risk) (4fractions/week) using the LEM-I RBE model. Institutional image-guidance (gold-markers) and rectal-sparing (SpaceOAR/SpaceOAR-vue) and bladder-protocol were applied during treatment-planning and implementation. Image-matching was based on pelvic bony-anatomy-matching followed by gold-markers-matching (optimal difference between 2 matches <5mm) (figure-1).

**Results**: The median follow up was 7 months (range, 2.3-20.4). The median IPSS-score, Gleason-score and serum PSA were 7(range, 2-18), 7(range, 7-10), 7ng/mL(range, 0.35-260) respectively (Figure-2). Only one patient had bio-chemical failure (Figure-2).Acute-toxicities (CTCAE-v.5) include grade-2 gastrointestinal 2(7.6%), genitourinary 3(11.5%), with no >/=grade-3 toxicities(CTCAE-v.5). There were no >/=grade-2 late-toxicities reported.

**Conclusion**: CIRT for HR-PCs can be safely performed with the European RBE model and including ENI. Early results confirm the excellent toxicity profile. Longer follow-up is required to assess oncological outcome.

### O 024 - Associated factors for patient selection in proton therapy

#### Djoya Hattu^1^, Lieke in ’t Ven^1^, Ruud Houben^1^, Judith van Loon^1^

##### ^1^Maastro Clinic, Radiation Oncology Department, Maastricht, Netherlands

**Introduction**: Patient selection for proton therapy is based on a predefined minimal difference in normal tissue complication probability (ΔNTCP) between the photon and proton treatment plan. A photon-proton plan comparison is indicated for almost all lung cancer patients treated with radical intent. It is not feasible for referring hospitals to refer all patients for a plan comparison. To select the correct patients, this study investigated parameters that are associated with a positive plan comparison outcome.

**Methods**: All lung cancer patients with a plan comparison in our institute were included in this study (N=193). The ΔNTCP between the photon and proton plan for two-year overall survival (driven by the mean heart dose) was calculated for these patients. Relevant patient and tumor characteristics were collected. Univariate analysis of these parameters was performed and p-values smaller than 0.05 were considered significant. ΔNTCP thresholds for a positive outcome of 2%, 4% and 5% were evaluated.

**Results**: The number of patients with a positive plan comparison were 134, 39, and 19 for a ΔNTCP of 2, 4, and 5% respectively. Results of the univariate analysis are shown in Table 1. Out of all the parameters tested, 11 and 16 parameters showed a significant positive and negative association with a positive outcome respectively.

**Conclusion**: The associated parameters for a positive plan comparison outcome could guide hospitals in selecting the optimal patients for referral for proton therapy. This could tackle the current bias in patient selection due to the geographic distance to the nearest proton facility.

### O 025 - An autoplan approach to generate the IMPT/VMAT plan comparison report for proton therapy financial clearance

#### Xiaodong Zhang^1^, Li Liao^1^, Mayankkumar Amin^1^, Cody Wages^2^, Narayan Sahoo^1^, Ronald Zhu^1^, Steven Frank^2^, Brandon Gunn^2^

##### ^1^University of Texas- MD Anderson Cancer Center, Radiaiton Physicis, Houston, USA ^2^University of Texas- MD Anderson Cancer Center, Radiation Oncololgy, Houston, USA

Increasingly, commercial payers are requiring comparative IMRT plans as part of the initial authorization process or as part of the appeal process for proton therapy insurance/financial clearance. At our center comparison plans have historically been developed by the clinical dosimetry teams using standard planning workflows, and it could take up to 1-2 weeks to provide comparison reports. This could lead to delays in reaching the financial clearance determination and patient care and provider/patient uncertainty and dissatisfaction. We developed an autoplan approach to generate the comparison plans which have the similar quality as the plans generated by our clinical dosimetry teams. Once the target contours are completed by the treating physician, the autoplan, implemented as a Raystation script, automatically generates OAR contours, designs beam angles, generates optimization objectives, performs optimization to achieve the best OAR sparing while meeting target prescription constrains and generates the plan comparison report. The system mainly generates the comparison plans for head neck cases (Figure1) but also generates the reports for brain, lung, esophagus, liver, pelvic and prostate cases (Figure2). By comparing the comparison plans generated by autoplan and clinical plan, we demonstrate that autoplan and clinical IMRT and VMAT plans are comparable: if a proton plan is better in one goal in clinical IMPT plan, proton plan is also better in the same goal in autoplan. The autoplan can provide the comparison report within 2 hours. Currently, the autoplan approach is routinely used in our center to provide the comparison report for proton therapy insurance approval.

### O 026 - Dirty Dose/Clean Dose: A novel concept to reduce uncertainty in proton relative biological effectiveness by optimizing not photon-like dose contributions

#### Lena Heuchel^1^, Christian Hahn^1,2,3^, Jakob Ödén^4^, Erik Traneus^4^, Jörg Wulff^5,6^, Beate Timmermann^5,6,7,8^, Christian Bäumer^1,5,6,7^, Armin Lühr^1^

##### ^1^TU Dortmund University, Department of Physics, Dortmund, Germany ^2^Faculty of Medicine and University Hospital Carl Gustav Carus- Technische Universität Dresden, Department of Radiatiotherapy and Radiation Oncology, Dresden, Germany ^3^Faculty of Medicine and University Hospital Carl Gustav Carus- Technische Universität Dresden- Helmholtz-Zentrum Dresden-Rossendorf, OncoRay – National Center of Radiation Research in Oncology, Dresden, Germany ^4^RaySearch Laboratories AB, RaySearch, Stockholm, Sweden ^5^WPE, West German Proton Therapy Center Essen, Essen, Germany ^6^University Hospital Essen, West German Cancer Center WTZ, Essen, Germany ^7^German Cancer Consortium DKTK, German Cancer Research Center DKFZ, Heidelberg, Germany ^8^University Hospital Essen, Department of Particle Therapy, Essen, Germany

**Purpose**: Applying photon dose threshold values for organs at risk (OAR) introduces uncertainties in proton therapy. To reduce uncertainties, a novel concept for creating photon-like “clean” dose plans optimizes “dirty” dose contributions from high linear energy transfer (LET) protons.

**Methods**: Clinically acceptable multi-field optimized planning target volume-based reference plans (REFplan) for cranial proton therapy patients were created using a constant relative biological effectiveness (RBE) of 1.1. Using the Wedenberg RBE model (α/β=2Gy), the LET leading to an RBE of 1.1 was calculated (LET_thres_) to divide the dose in two parts: photon-like clean dose (dose contribution of protons with LET<LET_thres_) and dirty dose (LET>LET_thres_). Max dirty dose objectives were added to penalize dirty dose contributions higher than a set dose level in critical OAR (DDopt). Research version of RayStation v11 was used for plan optimization and recalculation. Near-maximum values of dirty dose (DD_1_) and Wedenberg dose (D_wed,1_) and resulting normal tissue complication probability (NTCP_wed_) were calculated. For clarity, results for one representative chondrosarcoma patient are presented (LET_thres_=1.5keV/µm).

**Results**: For the OAR brainstem/chiasm/right/left optical nerve (all α/β=2Gy), DD_1_ reduction of 24.3/20.7/23.8/24.6Gy(RBE) was achieved leading to D_wed,1_ reduction of 2.0/3.5/5.9/4.8Gy(RBE) translating into NTCP_wed_ reduction of 9/14/14/5 percentage points (pp), respectively (Figure 1). Varying α/β from 2Gy to 3Gy led to less NTCP_wed_ changes for DDopt (average:1.0pp) than for REFplan (4.5pp).

**Conclusion**: The Dirty Dose concept allows for reducing RBE uncertainties by optimizing a physical dose parameter leading to more photon-like clean dose distributions in OAR with reduced dependence on biological parameters.

### O 027 - Development and benchmarking of a dose rate engine for raster scanned FLASH helium ions

#### Luisa Rank^1,2^, Ozan Dogan^1,3^, Benedikt Kopp^1^, Stewart Mein^1,4,5,6^, Gianluca Verona-Rinati^7^, Rafael Kranzer^8,9^, Marco Marinelli^7^, Andrea Mairani^1,4,5,6,10^, Thomas Tessonnier^1,4,6^

##### ^1^Heidelberg Ion-Beam Therapy Center HIT, Department of Radiation Oncology - Heidelberg University Hospital, Heidelberg, Germany ^2^Karlsruhe Institute of Technology KIT, Faculty of Physics, Karlsruhe, Germany ^3^Heidelberg University, Faculty of Physics and Astronomy, Heidelberg, Germany ^4^Heidelberg Institute of Radiation Oncology HIRO, National Center for Radiation Oncology NCRO and Heidelberg University Hospital UKHD and Heidelberg Faculty of Medicine MFHD and German Cancer Research Center DKFZ, Heidelberg, Germany ^5^Division of Molecular and Translational Radiation Oncology, Heidelberg Faculty of Medicine MFHD and Department of Radiation Oncology at Heidelberg University Hospital UKHD, Heidelberg, Germany ^6^Clinical Cooperation Unit Translational Radiation Oncology, German Cancer Consortium DKTK Core-Center Heidelberg and National Center for Tumor Diseases NCT and Heidelberg University Hospital UKHD and German Cancer Research Center DKFZ, Heidelberg, Germany ^7^University of Rome “Tor Vergata”, Department of Industrial Engineering, Rome, Italy ^8^University Clinic for Medical Radiation Physics, Medical Campus Pius Hospital - Carl von Ossietzky University, Oldenburg, Germany ^9^PTW-Freiburg, Research and Development, Freiburg, Germany ^10^Medical Physics, National Centre of Oncological Hadrontherapy CNAO, Pavia, Italy

Particle therapy at high dose and ultra-high dose rate (uHDR), “FLASH” therapy, seems to be a promising method to increase the efficacy of cancer treatments. The description of the so-called FLASH effect strongly depends on dose rate which is a complex quantity to assess for scanned particle therapy delivery. This work presents an analytical dose rate calculation engine for raster-scanned charged particle beams. By combining dose information and particle fluence, the dose rate is derived. Three calculation models are used to report dose rate: instantaneous, mean and threshold-based (ḋ5-95) as reported in recent works. The predictions are compared to instantaneous dose rate measurements with diamond detectors using different field sizes and depths in water. Raster-scanned uHDR helium-ion beam measurements (Figure 1a), performed at the Heidelberg Ion Beam Therapy Center (HIT), were used to validate the engine. Good agreement in instantaneous (inst.) dose rates was found between simulations and measurements (Figure 1b). Different dose-rate-calculation methods lead to sizeable differences in absolute dose rate values (Figure 1c). For varying depths in water, discrepancies between simulated and experimental ḋ5-95 values were below 10% (Figure 1d). Systematic modification of field sizes and scan paths yielded a mean dose rate prediction accuracy within 7% in the entrance channel. The calculation method, particle fluence and scanning path affect the dose rate, which underlines the need for standardized dose rate calculation. Through relatively accurate prediction of dose rate, the presented engine could help to further investigate the FLASH-effect with raster-scanned charged particles.

### O 028 - LETd optimization verification with an SOI microdosimeter

#### Vladimir Pan^1^, Nicolas Depauw^2^, Linh Tran^1^, Anatoly Rosenfeld^1^

##### ^1^University of Wollongong, Physics, Wollongong, Australia ^2^Massachusetts General Hospital, Radiation Oncology, Boston, USA

A first of its kind experimental verification of an LET_d_-optimized treatment plan for proton therapy has been carried out at the Massachusetts General Hospital (MGH), Boston, USA with a silicon-on-insulator (SOI) microdosimeter developed by the Centre for Medical and Radiation Physics (CMRP), Wollongong, Australia. A clinical treatment plan using two fields at 45- and 315- degree gantry angles was generated using single-field optimization (SFO) on a typical ependymoma structure set with Astroid, the proton treatment planning system (TPS) at MGH. A dose of 54 Gy(RBE = 1.1) was prescribed to the clinical treatment volume (CTV). The plan was then reoptimized in order to reduce the LET_d_ found within the brainstem. Both plans (original and reoptimized) were delivered with a scanning beam in a solid water phantom and the total experimental y_D_ was measured at various depths along the central axis through the isocentre, i.e. the bisector of the angles produced by the two beams. Following LET_d_ optimization, a clear reduction in the y_D_ was found within the brainstem by an average of ∼12% while maintaining adequate coverage of the tumour structure as shown in Figure 1.

### O 029 - Toward real-time adaptive proton therapy: A particle therapy custom-designed deep learning model

#### Ahmad Neishabouri^1^, Mein Stewart^2,3^, Kopp Benedikt^2^, Mairani Andrea^2^

##### ^1^German Cancer Research Center DKFZ, Clinical Cooperation Unit Radiation Oncology, Heidelberg, Germany ^2^Heidelberg University Hospital, Heidelberg Ion-Beam Therapy Center HIT - Department of Radiation Oncology, Heidelberg, Germany ^3^Perelman School of Medicine of the University of Pennsylvania, Department of Radiation Oncology, Philadelphia- Pennsylvania, USA

Adaptive proton therapy requires continuous tracking of patient movement and real-time adjustment of the proton beam. This necessitates an instantaneous dose calculation scheme capable of accurately simulating particle transport in complex geometries. Earlier, we introduced a novel deep learning (DL) approach capable of estimating Pencil Beam (PB) dose, fast and accurately enough to achieve this goal. However, although the introduced framework is capable of producing PB dose in millisecond time frames, upscaling the task to a beam consisting of multiple thousands PBs while maintaining instantaneous runtimes (subsecond) introduces multiple challenges, as the model design is highly impacted by the input size. In this context, the resolution in which the PBs are extracted from the original computed tomography (CT) and the ground truth Monte Carlo dose distributions are of major challenges. A coarse-resolution extract (3mm) will lead to a less-parametrized model therefore faster forward propagation, however, severely compromising accuracy, especially in longitudinal axes where any uncertainty in localizing the Bragg peak will yield a 3mm uncertainty. A fine-resolution extract (1mm) can alleviate this problem at the price of a lengthy inference time. Here, we present a particle therapy custom-designed DL model, based on the previously published Long Short-Term Memory (LSTM) model, which deftly profits from the characteristic of LSTM models being sequence-length independent, in favour of the particles’ characteristic energy release in the longitudinal axes. The presented model is 91% smaller and eliminates the inherent uncertainty of the previously published model, resulting in a better estimate of the Bragg peak position and magnitude.

### O 030 - Calculation of voxel-by-voxel dose rates in patients receiving proton pencil beam scanning radiotherapy

#### Rachael Hachadorian^1^, LaLonde Arthur^2^, Ethan Cascio^3^, Thomas Madden^1^, Jan Schuemann^4^, Daartz Juliane^5^

##### ^1^Massachusetts General Hospital - Harvard Medical School, Radiation Oncology, Boston, USA ^2^University of Montreal, Radiation Oncolocgy, Quebec, Canada ^3^Massachustts General Hospital, Francis Burr Proton Therapay Center, Boston, USA ^4^Massachusetted General Hospital, Deptartment of Radiation Oncology Research, Boston, USA ^5^Harvard Mass General Hospital, Radiation Oncology, Boston, USA

**Purpose and Objectives**: Treating patients with pencil beam scanning protons introduces the ability to treat using higher/more variable spot dose rates. Coupled with the recent surge in studies evaluating changes to biological response when using UHDR/FLASH) irradiations, this work presents an analytical means of discerning correlation between voxelwise dose rate and clinical outcomes This study uses analytical tools to both compute and measure maximum dose rates for clinical PBS treatment plans.

**Materials and Methods**: With the ability to extract a timing scheme and model an expected dose map, we define an array of dose rate quantities over a time interval of interest for each voxel. These quantities include the voxel-wise maximum instantaneous dose rate (VMIDR), voxel-wise effective dose rate (VEDR), Voxelwise dose at or above min dose rate (VDDR) to name a few. These values were analyzed using MiM.

**Results**: Dose rate distributions in patients are highly heterogeneous and are characterized by focal hot spots. Voxelwise maximum instantaneous dose rates for 10ms intervals in a typical cranial plan range from ∼50 Gy/min to ∼350Gy/min. Measured single point dose rates agree well with computed values.

**Conclusions**: The characteristics of voxelwise dose rate distribution are much different from previous established clinical practice. This computation presents a necessary tool for further investigation of correlation to clinical outcomes.

### O 031 - Evaluating the cost-effectiveness of hypofractionated consolidative radiotherapy for mediastinal Hodgkin lymphoma

#### Raymond Mailhot Vega^1^, Keaton Reiners^1^, Jonathan Fakhry^2^, Fantine Giap^2^, Adam Holtzman^1^, Eric Brooks^1^, Erin Mobley^3^, Susan Parsons^4^, Bradford Hoppe^5^, Nancy Mendenhall^1^

##### ^1^University of Florida Proton Therapy Institute, Radiation Oncology, Jacksonville, USA ^2^University of Florida, Radiation Oncology, Gainesville, USA ^3^University of Florida, Surgery, Jacksonville, USA ^4^Tufts University, Medicine, Boston, USA ^5^Mayo Clinic, Radiation Oncology, Jacksonville, USA

**Introduction**: Proton therapy (PT) has been shown to deliver lower cardiac doses for patients with mediastinal Hodgkin lymphoma (HL). While its cost has been claimed to be prohibitive, published cost-effectiveness guidances provide decision-makers with aids for resource allocation with respect to cardiac toxicity. During the COVID-19 pandemic, the International Lymphoma Radiation Oncology Group (ILROG) provided hypofractionated regimen recommendations for patients with HL. We sought to evaluate and compare the cost-acceptability of established conventional versus hypofractionated regimens.

**Methods**: We utilized population-based data to create a Markov model to compare cost-acceptability thresholds based on mean heart dose (MHD) for PT compared to intensity-modulated radiation therapy (IMRT) favorability for a given IMRT dose. The HD14 trial informed relapse risk. Coronary heart disease risk was informed by the Framingham risk calculator modified by the mean heart dose from radiotherapy. The hypofractionated and conventional regimens assumed 27Gy in 9 versus 30.6Gy in 17 fractions with approximately equivalent EQD2, as per ILROG. The base case patients were a 30-year-old man and woman. Effectiveness was measured in Quality-Adjusted Life Years (QALYs) and costs in 2018 USD.

**Results**: Model calibration corroborated relapse and cardiotoxicity estimates. The results of the MHD driven cost-acceptability thresholds with hypofractionation are displayed in Table 1 below. On average, a mean heart dose reduction of 2.2Gy and 3.2Gy for men and women, respectively, favored proton cost-acceptability at $100K/QALY.

**Conclusion**: Hypofractionated regimens are not the standard for consolidative radiotherapy for HL. If explored prospectively, PT hypofractionated regimens would have lower MHD cost-effective thresholds than conventional regimens.

### O 032 - Significant reduction of acute and late toxicity after IMPT in nasopharyngeal cancer

#### Hans Langendijk^1^, Tineke Meijer^1^, Anne van den Hoek^1^, Stefan Both^1^, Edwin Oldehinkel^1^, Hans Verbeek^1^, Dan Scandurra^1^, Jeffrey Free^1^, Roel Steenbakkers^1^

##### ^1^The University Medical Center Groningen UMCG, Radiation Oncology, Groningen, Netherlands

**Introduction**: The aim of study was to test the hypothesis that IMPT reduces acute and late radiation toxicity in nasopharyngeal cancer patients.

**Materials and Methods**: The study population was composed of 112 NPC patients treated with curative radiotherapy (RT) or chemoradiation. Between 2007 and Dec 2017, alle patients were treated with IMRT or VMAT. Since January 2018, 72 out of 74 patients (97%) qualified for IMPT according to model-based selection. All patients were included in a prospective data registration program in which acute and late toxicity was prospectively scored assessed before, during and after RT. To determine the overall effect on acute and late toxicity, the Weighted Overall Toxicity Score (WOTS) was calculated, defined as the sum of all toxicities weighted by toxicity grading. In addition, the WOTS Area Under the Curve (WOTS-AUC) was calculated representing the WOTS from the start of treatment until 24 months after completion of treatment.

**Results**: IMPT resulted in significant reductions of various acute and late toxicities (Figure 1), including xerostomia, loss of taste, dysphagia, tube feeding dependence, sore mouth, and mucosal reactions. Only acute dermatitis was significantly worse at the end of IMPT, but completely recovered at 5 weeks after treatment. From week 4 to 24 months, the WOTS was significantly lower after IMPT (Figure 2). In the IMPT group, the WOTS-AUC as measure for overall toxicity was 53% lower.

**Conclusion**: In this prospective cohort study, IMPT resulted in more than 50% reduction of various acute and late toxicities.

### O 033 - Financial toxicity in patients receiving proton therapy abroad: A prospective cross-sectional study

#### Barbara Bachtiary^1^, Léonie Grawehr^1^, Damien Charles Weber^1^

##### ^1^Paul Scherrer Institut, Center for Proton Therapy, Villigen, Switzerland

**Purpose**: As proton therapy is unavailable in some countries, cancer patients often must be treated abroad. By definition, this treatment is associated with higher costs, which patients often have to pay themselves partly. This observational study aimed to assess the financial burden (“financial toxicity”) and coping strategies in foreign patients who underwent proton therapy (PT) at the Paul Scherrer Institute.

**Methods**: Thirty-nine patients participated in the study, of which 30 (77%) were adults (median age, 51 years; IQR [37.0, 62.8]) and nine (23%) caregivers of children with cancer (median age of 12 years; (IQR [6.0, 14.0]). Financial toxicity was evaluated using the Comprehensive Score for financial Toxicity (COST) measure. The score ranges from 0 (financial ill-being/distress) to 44 (perfect financial well-being). Financial coping strategies were explored using polar questions (yes-no questions).

**Results**: Patients came from 13 different countries, but most were from Europe (n=35; 90%). The median score for financial toxicity was 25 (IQR [19.9, 30.0]) for all patients, 21.5 (IQR [17.0, 31.5]), and 25 (IQR [24.8, 29.0]) for adults and caregivers, respectively. The analysis of coping strategies revealed that 61.5% of the patients had to spend savings, 23.1% had to borrow money, 53.8% had to limit their leisure activities, and 20.5% had to reduce their expenditure on food and clothing due to the treatment.

**Conclusion**: Cancer patients from foreign countries where PT is not available and therefore have to travel abroad, treated at PSI/Switzerland, are substantially affected by financial toxicity.

### O 034 - Proton and carbon ion radiotherapy for operable early stage lung cancer: Three-year results of a prospective nationwide registry

#### Hideyuki Harada^1^, Hiroaki Suefuji^2^, Keita Mori^3^, Masaki Nakamura^4^, Nobuteru Kubo^5^, Haruhiko Nakayama^6^, MIyako Satouchi^7^, HIdefumi Aoyama^8^, HIroshi Tsuji^9^, Yoshiyuki Shioyama^10^

##### ^1^Shizuoka Cancer Center, Radiation and Proton Therapy Center, Nagaizumi, Japan ^2^SAGA-HIMAT Foundation, Ion Beam Therapy Center, Tosu, Japan ^3^Shizuoka Cancer Center, Department of Biostatistics- Clinical Research Center, Nagaizumi, Japan ^4^National Cancer Center Hospital East, Division of Radiation Oncology and Particle Therapy, Kashiwa, Japan ^5^Gunma University Graduate School of Medicine, Department of Radiation Oncology, Maebashi, Japan ^6^Kanagawa Prefectural Hospital Organization, Vice Chairman, Yokohama, Japan ^7^Hyogo Cancer Center, Department of Thoracic Oncology, Akashi, Japan ^8^Hokkaido University, Department of Radiation Oncology- Faculty of Medicine, Sapporo, Japan ^9^National Institutes for Quantum and Radiological Science and Technology, QST Hospital, Chiba, Japan ^10^SAGA HIMAT Foundation, Ion Beam Therapy Center, Tosu, Japan

**Purpose**: The purpose of this analysis was to clarify the clinical outcomes of particle-beam radiation therapy for operable early stage lung cancer.

**Materials and Methods**: Patients of early stage lung cancer (T1-T2bN0) who were eligible for radical surgery but did not wish to undergo surgery were treated by proton-ion (PT) or carbon-ion (CT) radiation therapy and registered in Japanese prospective registry. For PT, peripheral type and central type were treated 66-70 Gy(RBE) in 10 fractions and 72.6-80 Gy(RBE) in 22-25 fractions, respectively. For CT, peripheral type and central type were treated 54.0-64.0 Gy (RBE) in 4 fractions or 50 Gy (RBE) in single fraction, and 54-72 Gy (RBE) in 9-16 fractions, respectively.

**Results**: Two hundred seventy-four (274) patients were enrolled and included in efficacy and safety analyses. Most tumors were adenocarcinoma (44%), and 105 (38%) were not histologically confirmed and diagnosed clinically. 250 (91%) of 274 patients had tumors that were peripherally situated. 138 (50%) and 136 (50%) patients were treated by PT and CT, respectively. The median follow-up time for all censored patients was 42.8 months (IQR 36.7 – 49.0). Median overall survival had not been reached (95% CI not reached–not reached), 3-year progression free survival was 81% (95% CI;76–86) and overall survival was 93% (95% CI;89–96), respectively. 3-year cumulative local relapse ratio was 4% (95% CI; 1-6). No grade 3 or severe treatment-related toxicity was observed.

**Conclusion**: Particle therapy for operable early-stage lung cancer was a safe treatment with excellent 3-year overall survival and progression free survival.

### O 035 - Highly customized moderately hypofractionated proton therapy for large/extralarge uveal melanomas: A randomized phase II trial

#### Juliette Thariat^1^, joel herault^2^

##### ^1^Centre baclesse/ARCHADE, Radiation Oncology, caen, France ^2^Centre lacassagne, radiation physics, nice, France

**Background**: Patients with large uveal melanomas are at major risk of metastases. Enucleation is the standard treatment, but some patients are reluctant to immediate enucleation. Proton therapy yields five-year local control rates and eyeball retention of >85% and ≈20% in large uveal melanomas. Patients with T3/T4 uveal melanomas refusing enucleation were randomized between standard 4-fraction or moderately hypofractionated 8-fraction proton therapy. The main endpoint was the two-year local recurrence-free survival without enucleation.

**Material and Methods**: A single-masked 1:2 randomized phase II trial was conducted between 2015 and 2017; with planned endoresection and distance to the posterior pole as strata. Local events were defined as local relapse, or enucleation due to complications or relapse.

**Results**: The 32 patients mean 64 yo, had T3/4 (N=17/15), M1 (N=2) tumors, of mean diameter 16.5mm and thickness 9.1mm; posterior in 56.5%. PT customization in standard/experimental arms included contralateral eye fixation > 64/71%, 3 lid retractors 46/43%, instable eye 27/43% (mean Xrays 37/66), wedge 27/57% in addition to ophthalmologic customization (MR, transillumination, 5 clips). Median follow-up was 56.7 months. Two-year local recurrence-free survival rate without enucleation was 80%, in both arms. Of 9 enucleations, 3 were for relapse and 6 toxicities. Two year-overall survival was 72%. Mean difference was 1.44/1.01(logMAR) in visual acuity in standard/experimental arms (p=0.39).

**Conclusion**: More moderate hypofractionation is feasible without deteriorating local control and with similar toxicity rates in patients with large uveal melanomas. It required substantial customization to adapt to low vision and large tumor volume. Larger studies incorporating adjuvant treatments are warranted.

### O 036 - Is there a role for mitotic catastrophe in the formation of immunogenic cytosolic dsDNA after photon or carbon ion exposure?

#### Claudia Fournier^1^, Alexander Helm^1^, Nicole Averbeck^1^, Cristina Totis^1^, Hintze Dennis Fabian^1^, Burkhard Jakob^1^, Marco Durante^1^

##### ^1^GSI Helmholtz Center Darmstadt, Biophysics, Darmstadt, Germany

A combination of radiotherapy and immunotherapy can enhance the immunogenicity of tumor cells and elicit stable immune responses. Recent work showed that immunogenicity in breast cancer cells is enhanced by the occurrence of radiation-induced cytosolic dsDNA, followed by activation of the cGAS-STING pathway and release of IFN-β, prerequisites for the activation of immune cells. A fractionated dose of 3x8 Gy was found particularly efficient. Charged particles bear advantages for a combined therapy, because circulating immune cells are spared more efficiently. The higher biological effectiveness of carbon ions might imply more pronounced immunogenic effects. We quantified radiation-induced, cytosolic dsDNA of triple-negative, breast cancer cells (4T1, mouse) 24 h after exposure to doses of up to 24 Gy (single and fractionated doses), comparing carbon ions and photons. To trace back the origin of the dsDNA fragments, occurrence of cell death, formation of micronuclei and cell-cycle progression were assessed. We analysed c-GAMP induction (surrogate for cGAS-STING activation), and IFN-β release as a read-out for immunogenicity. For both carbon ions and photons, we detected dsDNA fragments in the cytoplasm and features of mitotic catastrophe. Carbon ions were found equally or slightly more effective. The magnitude of the effect was dependent on dose and G2-M cell-cycle arrest. The formation of micronuclei, which are discussed as possible origin of cytoplasmic dsDNA fragments, was of minor importance. This points to mitotic catastrophe as a possible source of dsDNA fragments.

### O 037 - Particle radiation may bear the potential to widen the dose window for abscopal effects in combination with immunotherapy

#### Thomas Friedrich^1^, Vladislav Sandul^1^, Michael Scholz^1^, Marco Durante^1^

##### ^1^GSI Helmholtzcenter for Heavy Ion Research, Biophysics, Darmstadt, Germany

The combined action of immunotherapy with immune checkpoint blockers and radiation therapy has proven to allow for abscopal response in some cases. However, not all patients respond to this combination and the reason for this selectivity is still unclear. We developed a dynamical model [1] to describe tumor growth inhibition and the onset of immune reactions following a schedule of checkpoint blocker delivery and radiation. The model has been validated with a broad set of preclinical data at low LET. It predicts the existence of a dose window for abscopal effects, in accordance to experimental observations. If the tumor dose is too small, it is not sufficient to trigger an immune response. If it is too large, it may inhibit immune response by immune cell inactivation. By considering the enhanced effectiveness of ion radiation as well as the inverted dose profile within the model, a widening of the dose window for abscopal response is expected for two reasons. First, cell inactivation is induced stronger and consequences are developing sooner as compared to photon exposure. This would allow a systemic response at lower doses. Second, because with particle radiation lower doses to volumes outside the target field are achieved, blood and lymphoid organs receive less dose, which spares immune cells and allows higher tolerable target doses. The model makes predictions for dedicated preclinical experiments testing for abscopal responses.

### O 038 - Glutaminase 1 inhibition sensitizes radioresistant KEAP1 mutant lung cancer cell lines to protons

#### Scott Bright^1^, Mandira Manandhar^1^, Rishab Kolachina^1^, David Martinus^1^, Mariam Ben Kacem^1^, Poliana C. Marinello^1^, Tingshi Su^1^, David B. Flint^1^, Simona F. Shaitelman^2^, Gabriel Sawakuchi^1^

##### ^1^MD Anderson, Radiation Physics, Houston, USA ^2^MD Anderson, Radiation Oncology, Houston, USA

Mutations in the protein Kelch-like ECH-associated protein 1 (KEAP1), which is observed in approximately 20% of lung cancers, rewires metabolism by constitutively activating nuclear factor-erythroid factor 2-related factor 2 (NRF2), which then drives production of the antioxidant glutathione (GSH) from glutamate. KEAP1 mutant cells are critically reliant on the conversion of glutamine to glutamate via the enzyme Glutaminase-1 (GLS1) to support GSH production. We hypothesized that KEAP1 mutant cells are radiosensitized by GLS1 inhibition (GLS1i) because they lack enough glutamate to sustain antioxidant production and radiation will therefore induce cytotoxic levels of oxidative stress. We performed clonogenic survival assays in NCI-H460 and NCI-H1299 cells with wild type or silenced KEAP1. Cells were exposed to 6 MV x-rays or 3.9 keV/µm protons and treated with IACS-6274, a novel GLS1i discovered at MD Anderson and currently in clinical trials. We determined radiosensitization metrics from the clonogenic survival curves, alongside assays investigating DNA damage, GSH consumption and lipid peroxidation. We observed that KEAP1 silenced cells were much more radioresistant compared to KEAP1 wild type cells. IACS-6274 radiosensitized both wildtype and silenced KEAP1 cells. NCI-H460 showed increased RBE with GLS1i while NCI-H1299 showed no difference. Radiosensitization could be rescued with an ROS scavenger. GLS1i reduced the oxidative health of cells and increased radiation induced lipid peroxidation. Our results suggest that IACS-6274 is a potent radiosensitizer for lung cancer including a radioresistant sub-population with mutations in KEAP1. GLS1i combined with protons may be a novel treatment strategy for unresectable KEAP1 mutant lung cancer.

### O 039 - Recombinant endostatin combined with proton therapy for non-small cell lung cancer: An attractive treatment strategy?

#### Charnay Cunningham^1,2^, Julie Bolcaen^2^, Alessandra Bisio^3^, Amanda Genis^1^, Hans Strijdom^1^, Charlot Vandevoorde^4^

##### ^1^Stellenbosch University, Centre for Cardio-metabolic research in Africa CARMA- Division of Medical Physiology, Stellenbosch, South Africa ^2^NRF iThemba LABS, SSC LABS- Radiation Biophysics Division, Cape Town, South Africa ^3^University of Trento, Department of Cellular- Computational and Integrative Biology – CIBIO, Trento, Italy ^4^GSI Helmholtz Centre for Heavy Ion Research, Biophysics Department, Darmstadt, Germany

Non-small cell lung cancer (NSCLC) is the most prevalent type of lung cancer, which remains the leading cause of cancer-related mortality worldwide. Anti-angiogenic drugs, such as recombinant endostatin (RE), have been intensely studied to inhibit tumour angiogenesis in NSCLC, leading to mixed and often disappointing results in (pre)clinical trials. However, there is an emerging interest in the potential of RE to create a vascular normalization window, which can convert the functionally abnormal tumour vasculature into a more ‘normal’ state. This effect can enhance the systemic delivery of cytotoxic drugs, immunotherapy, and improve tumour radiosensitivity by the reduction of tumour hypoxia. Despite the fact that previous studies revealed distinct differences in angiogenic effects between proton and photon irradiation, combination treatments with RE and proton therapy remain underexplored. Therefore, this in vitro study investigates the up- and down-regulation of angiogenic factors in three cells lines that are relevant to the NSCLC tumour microenvironment (human lung fibroblasts, HUVEC and A549 cells) after low and high doses of 230 MeV proton and photon irradiation, combined with RE. In addition, the impact of the combination treatment is evaluated on cell migration, tubulogenesis, invasion and cell proliferation. The results of the irradiation campaigns are currently under analysis and will be presented at PTCOG61. This will allow us to gain a better insight in the angiogenic mechanisms induced by protons and photons, which will need to be taken into consideration in order to maximize the potential of RE in combination treatments with proton therapy for NSCLC.

### O 040 - Sequence of combined chemo-radiotherapy with doxorubicin and proton LET affect cellular dose response of preclinical sarcoma and normal tissue models

#### Teresa Bernardo^1^, Diana Klein^2^, Lena Heuchel^3^, Carina Behrends^3,4,5^, Christian Möllers^1^, Beate Timmermann^1,4,5,6^, Armin Lühr^3^, Cläre Von Neubeck^1^

##### ^1^University Duisburg-Essen, Department of Particle Therapy, Essen, Germany ^2^University Hospital Essen, Institute of Cell Biology, Essen, Germany ^3^TU Dortmund University, Department of Physics, Dortmund, Germany ^4^University Hospital Essen, West German Proton Therapy Centre Essen WPE, Essen, Germany ^5^University Hospital Essen, West German Cancer Centre WTZ, Essen, Germany ^6^German Cancer Consortium DKTK, Partnersite Essen, Essen, Germany

Soft tissue sarcoma (STS) are clinically treated with surgery and doxorubicin (Dox, first line drug) in combination with conventional photon (X) or proton (H+) beam radiotherapy (RT) to spare normal tissue. The project aims at 1) identifying effects of sequence alterations of combined treatments (XRT, HRT, Dox) relative to 2) LET changes resulting in increased relative biological effectiveness (RBE) on a cellular level in preclinical STS models (fibrosarcoma, giant cell tumor, rhabdomyosarcoma) and human endothelial cells (HMEC), representing normal tissue. The cellular response was analyzed regarding clonogenic cell survival (colony formation), viability (WST-1 assay), proliferation (CV-assay), apoptosis (Nicoletti assay), and cell motility (scratch assay) at different time points (48 h, 96 h) and in three sequences (Dox only before, before and after, or only after irradiation). The depth dose profile of a Bragg peak was modelled with a range shifter block of 12 different layers. Corresponding doses and LETs were computed via the treatment planning system RayStation. Overall, the combination of Dox treatment before and after RT is most effective, with preferences of fibrosarcoma for HRT and rhabdomyosarcoma for XRT. The cellular viability and proliferation were dose and LET dependent affected, and mimicked the inverted depth dose profile of the Bragg peak. The biological endpoints showed a time dependency which seems related to a cell cycle arrest or the cell doubling time. Comparison with XRT and RBE calculations are ongoing.

### O 041 - Status of the NHA C400 system: A cyclotron for particle therapy

#### Philippe Velten^1^, Olivier Cosson^2^, Laurent Maunoury^1^, Jérôme Mandrillon^3^, Vincent Nuttens^3^, Willem Kleeven^3^, Michel Abs^3^, Philippe Cailliau^4^, Sébastien Deprez^5^, Laurent Koffel^5^

##### ^1^Normandy Hadrontherapy, Physics, Caen, France ^2^Normandy Hadrontherapy, System, Caen, France ^3^Ion Beam Applications, Physics, Louvain-la-Neuve, Belgium ^4^Ion Beam Applications, System, Louvain-la-Neuve, Belgium ^5^Ion Beam Applications, Hardware, Louvain-la-Neuve, Belgium

The NHa C400 is an isochronous cyclotron for cancer therapy delivering high dose rates of alpha to carbon ions at 400 MeV/amu and proton ions at 260 MeV. The NHa company, of which IBA is a major shareholder, designs, produces and markets the new multi-ions C400 based therapy system while IBA experts are deeply involved in all aspects of its conception. The first multi-ions system will be delivered to the CYCLHAD centre in Caen-Normandy-France which is already equipped with an IBA Proteus®ONE protontherapy system in operation since 2018. With the NHa system, multiparticle hadrontherapy inherits many proven technologies and latest innovations developed for the IBA Proton Therapy systems driven by cyclotrons. Specifically for this new system, an injection beamline is developed comprising three independent ion sources (H_2_^+^;^4^He^2+^;^12^C^6+^) mounted on a platform on top of the cyclotron vault. Protons are obtained via stripping of H_2_^+^ while ions are extracted via an electrostatic deflector followed by a magnetostatic channel. We will explain the main technical advantages of the C400 based solution, describe the most innovative concepts and technical solutions on the accelerator and show the construction progress.

### O 042 - Experimental demonstration of momentum cooling to halve the treatment delivery time in PSI’s OPTIS2 beamline

#### Vivek Maradia^1,2^, David Meer^1^, Rudolf Dölling^3^, Jan Hrbacek^1^, Damien Charles Weber^1,4,5^, Antony John Lomax^1,2^, Serena Psoroulas^1^

##### ^1^Paul Scherrer Institut, Center for Proton Therapy, Villigen, Switzerland ^2^ETH Zurich, Physics Department, Zurich, Switzerland ^3^Paul Scherrer Institut, Large Accelerator Facility, Villigen, Switzerland ^4^University of Bern, University Hospital Bern, Bern, Switzerland ^5^University Hospital Zurich, Radiation Oncology, Zurich, Switzerland

Over the last five decades, protons (70 MeV) are used to treat eye tumors. However, in cyclotron-based proton therapy facilities with gantries, the output energy from the cyclotron is 230/250 MeV beam. To achieve a 70 MeV beam, it is required to use the degrader, which increases the momentum spread (up-to ±4.5%) of the beam. Ocular treatments however require a low momentum spread (±0.25%) to achieve a sharper distal fall-off of the Bragg peak. Low momentum spread is achieved using the slits in the energy selection system (ESS) at the expense of low beam current/transmission. This currently limits the dose rate that can be delivered to the patient to 15 Gy/min with a total transmission of 0.27% from the cyclotron to the isocenter, corresponding to treatment times of about 1 minute. Long treatment time can be difficult for many patients. Here we propose the use of momentum cooling using a wedge in the ESS (instead of a slit) to reduce the momentum spread of the beam without introducing significant beam losses. For our experiment, we transport the maximum acceptable momentum spread of ±1.3% up to the wedge, to reduce the momentum spread to <±0.3% using a wedge made of polyethylene (Fig.1). By using a wedge, we obtain a factor 2 higher transmission, which could eventually halve the treatment delivery time and increase patient comfort. Due to these encouraging results, we are currently preparing to implement this solution for clinical use in the OPTIS2 beamline.

### O 043 - “StaticArc”: A new particle therapy delivery technique using current level of technology

#### Dalfsen Raymond^1^, Louis Genet^1^, Stuart Swerdloff^2^

##### ^1^Elekta Pty Ltd, SW Product Management, North Sydney, Australia ^2^Elekta NZ, OIS Particle Therapy Solutions, North Sydney, Australia

**Purpose**: *StaticArc*, a form of Step-and-Shoot multi-angle treatment, is a technique that can be performed using current technologies, gaining dosimetric benefits from utilizing multiple treatment fields, while improving treatment fraction efficiency. The basic principle is to concatenate a collection of static PBS beams into a single consolidated beam where the change in gantry angle occurs when the beam is off. This technique avoids the need for manual beam selection and repetitive verification of parameters that are unchanged between originating beams, resulting in better throughput. We elucidate the workflow involved, using commercial software solutions and a treatment machine Emulator.

**Methods and Materials**: Using Monaco 6.2, MOSAIQ 3.2.1, and a treatment machine Emulator (ELEKTA IFS1909 v1.54), a clinically representative, 7 Field PBS plan was created and exported as a DICOM RT Ion Plan to MOSAIQ using the Consolidated Field Sequence (CFS) feature in Monaco. This process was then repeated for the same plan, without employing CFS.

**Results**: Both plans were “delivered” on the machine (emulator) successfully. Time required to prepare the plan in the OIS was reduced by 35%, and treatment delivery time overhead (exclusive of beam delivery and gantry motion) from beginning of setup to end of (last) beam and recording was reduced by 70%.

**Conclusion**: Use of *StaticArc* provides significant efficiencies in department throughput and potential dosimetric advantages, without requiring beam on during gantry rotation, through the ability to utilize a higher number of beams than typical. It is hoped that this capability will serve as an immediate and intermediate step towards Dynamic PT Arc delivery.

### O 044 - Design of a large energy acceptance beamline demonstrator for the TURBO project

#### Adam Steinberg^1^, Hannah Norman^1^, Jacinta Yap^2^, Robert Appleby^1^, Suzie Sheehy^2^

##### ^1^University of Manchester, Department of Physics and Astronomy, Manchester, United Kingdom ^2^University of Melbourne, Department of Physics, Melbourne, Australia

Hadron therapy has a long beam delivery time, which limits the number of patients treated and leads to interplay effects. Promisingly, these issues may be mitigated by increasing beamline energy acceptance to over ±30%, but delivering such a wide range of energies is not possible with conventional accelerator methods. We investigate the use of Fixed Field Accelerator optics, which may enable simultaneous transport of all energies. A key challenge with Fixed Field optics is integration with other accelerator systems, as it is difficult to match all accelerator parameters simultaneously, and the trajectory of particles through the beamline varies with energy. To demonstrate the required concepts, the TURBO project at the University of Melbourne is investigating the design of a large energy acceptance beamline using a scaled-down prototype. This novel beam transport system will be able to deliver a continuous beam of protons between 0.5-3.5MeV, generated by a Pelletron with an energy degrader. The design is a closed-dispersion arc, which could easily be integrated into facilities where a beam is extracted from a cyclotron, synchrotron, or linac. We discuss the key features of the beamline in this work. In addition, we show that studies of beam and magnet errors indicate that this arc is robust under realistic operating conditions, as is required for clinical operation. The construction of this low energy demonstrator beamline would verify these results, paving the way for translation to new and existing facilities.

### O 045 - A comparative dosimetric phantom study for characterization of a novel dedicated ocular nozzle that uses Pencil Beam Scanning delivery

#### Mark Pankuch^1^, Mingcheng Gao^1^, Hazel Wang^1^, Amanda Duggal^1^, William Hartsell^2^

##### ^1^Northwestern Medicine Chicago Proton Center, Medical Physics, Warrenville, USA ^2^Northwestern Medicine Chicago Proton Center, Radiation Oncology, Warrenville, USA

**Purpose**: Small proton fields are required to treat ocular targets. Typical PBS fields require use of a range shifter(RS) creating a penumbra that is too large to maintain adequate plan quality. PBS beam characteristics can be improved with the implementation of a novel dedicated ocular nozzle.

**Methods**: A virtual PBS machine was constructed in RayStation with a 7.5cm RS positioned 50cm upstream from customizable apertures. Five idealized targets ∼1.5cm in size: a box, a circle, a triangle and two crescents (up and down) were centered 2cm deep in a uniform phantom. PBS plans using the ocular nozzle were optimized to deliver 10Gy(RBE). PBS dose distributions were compared to our institution’s standard ocular beams which use Uniform Scanning(US) with traditional apertures and compensators. PBS plans were optimized to achieve identical target coverage as the US plans.

**Results**: To quantify dose spillage around the targets, 5mm expansion structures were created distal, proximal and lateral to each shape. Mean doses in the lateral and proximal volumes are lower for all PBS plans. Distal mean doses are higher in the PBS plans due to inferior distal shaping. CI(100%) and CI(50%) were lower in the PBS plans except for CI(100%) of the triangle target where PBS distal conformity was poor. Lateral penumbra of the PBS beams were more than 1mm smaller for all targets. Distal penumbras were nearly identical.

**Conclusions**: PBS fields using the ocular nozzle demonstrate less dose spillage lateral and proximal to the targets with less conformity distally.

### O 046 - Independent risk model validation confirms variable proton RBE inducing late-occurring brain injuries

#### Julia Bauer^1,2,3^, Semi Harrabi^1,2,3,4,5^, Tanja Eichkorn^1,2,3^, Emanuel Bahn^1,2,3,4^, Lia Velkova^6,7^, Klaus Herfarth^1,2,3,5^, Jürgen Debus^1,2,3,4,5,8^, Markus Alber^1,2,3^

##### ^1^Heidelberg University Hospital, Radiation Oncology, Heidelberg, Germany ^2^NCT, National Center for Tumor Diseases, Heidelberg, Germany ^3^HIRO, Heidelberg Institute for Radiation Oncology, Heidelberg, Germany ^4^German Cancer Research Center, Clinical Cooperation Unit Radiation Oncology, Heidelberg, Germany ^5^HIT, Heidelberg Ion-Beam Therapy Center, Heidelberg, Germany ^6^Institute for diagnostic and interventional radiology, Municipal Clinical Center Karlsruhe, Karlsruhe, Germany ^7^Contributed during the employment at 1, now with 6, Heidelberg, Germany ^8^DKTK, German Cancer Consortium- partner site Heidelberg, Heidelberg, Germany

We report on the independent validation of a risk model predicting MRI-contrast enhancing brain lesions (CEBLs) in low-grade-glioma (LGG) patients observed after proton therapy. The model suggests a variable proton RBE and an enhanced radiosensitivity of the periventricular brain region (PVR). In contrast to conventional normal-tissue complication probability (NTCP) models, the validated model predicts statistically independent probabilities for the endpoint “origin of lesion” (POLO) in each brain tissue voxel. POLO distributions are calculated based on absorbed dose, dose-averaged linear energy transfer (LET_d_), and PVR information, thus providing a spatially-resolved risk map indicating critical regions (figures (a, b)). Assuming serial complication characteristics, the POLO map is condensed into an NTCP per subject and compared to clinically observed CEBL incidence. We identified 119 LGG patients for validation, whereof 23 developed at least one CEBL. The observed incidence of 19.3% matched the predicted mean NTCP of 18.9%. A good NTCP calibration was observed (figure (c)). Main differences between validation and model cohort (in brackets) relate to the target volume (median V_PTV_=245(208) cm³) and CEBL size (median V_CEBL_=110(235) mm³). Revision of model parameters based on ∼58 Mio. observations (voxels) yielded a less pronounced RBE effect (LET_d_ dependency) and enhanced PVR sensitivity compared to the original model. This emphasizes the impact of uncertainties in lesion origin (present in extended CEBLs) on risk prediction. For the first time, our results independently confirm previously reported clinical evidence for proton RBE larger than 1.1 and enhanced PVR radiosensitivity, calling for a paradigm shift in brain tumour proton treatment.

### O 047 - Sacral-nerve-sparing strategy in carbon-ion radiotherapy (CIRT) for treatment of pelvic sarcomas: The MedAustron approach of physical nerve-sparing and LET evaluation

#### Ankita Nachankar^1,2^, Eugen Hug^1^, Maciej Pelak^1^, Slavisa Tubin^1^, Marcus Stock^3,4^, Antonio Carlino^3^, Joanna Gora^3^, Giovanna Martino^3^, Mansure Schafasand^3,5^, Piero Fossati^1,6^

##### ^1^MedAustron Ion Therapy Center- Austria, Department of Radiation Oncology, Wiener Neustadt, Austria ^2^ACMIT- Austria, Medical Research and Development, Wiener Neustadt, Austria ^3^MedAustron Ion Therapy Center- Austria, Medical Physics, Wiener Neustadt, Austria ^4^Karl Landsteiner University of Health Sciences- Austria, Medical Physics, Krems an der Donau, Austria ^5^Medical University of Vienna- Department of Radiation Oncology- Vienna- Austria, Medical Physics, Wien, Austria ^6^Karl Landsteiner University of Health Sciences- Austria, Department of Radiation Oncology, Krems an der Donau, Austria

**Purpose**: Radiation-induced lumbosacral neuropathy (RILSN) is a potentially debilitating toxicity following high-dose radiotherapy for pelvic malignancies. We evaluated the feasibility of a sacral-nerve-sparing optimized (SNSo-CIRT) strategy.

**Material and Methods**: Individual sacral-nerve roots (L5–S3 levels) were routinely contoured (Figure 1a). SNSo-CIRT plans prescribed to dosages of 73.6 (64-76.8) Gy RBE/16 fractions (4 fractions/week) using the local effect model-I(LEM-I). We subsequently recomputed doses based on the modified microdosimetric kinetic model (mMKM). Doses to sacral-nerve-roots and sacral-nerves-to-spare (outside high-dose CTV) were limited to D2% <73 Gy RBE and D5% <69 Gy RBE (LEM-I) respectively, and hot spots were avoided (Figure 1b).

**Results**: Pelvic sarcomas/chordoma patients (n = 30) treated with SNSo-CIRT from August 2019 -February 2022 were analyzed. The median follow-up was 15 months (range, 11.9-18). Four patients (13.3%) developed grade-2/3 RILSN (CTCAE v5). The median time to develop RILSN was 13.6 months (range, 10.6-16.6). RILSN-free survival at 1 & 2-years is 90% (range, 79-100) and 82.5% (range, 65-100) respectively. LEM-I, mMKM doses were comparable in patients with/without RILSN. Patients with RILSN had high LETd on sacral-nerves, LETd to 1, 2 and 4 cm length of sacral-nerves were significant predictors for RILSN-free survival (p<0.05) (figure 2). Other grade-2/3 toxicities (CTCAE v5) were, tumor/treatment associated local pain (57%), paraesthesia (13.3%), wound complications (10%), sacral-insufficiency fractures (6.7%), urinary dysfunction (6.7%). One & 2-year local control was 90% (CI, 90-100) & 80% (CI, 83-100) respectively.

**Conclusion**: SNSo-CIRT is technically feasible. LETd evaluation for sacral-nerves appears clinically promising and relevant. A prospective clinical study will commence soon.

### O 048 - Predicting pulmonary toxicities from proton radiotherapy in locally advanced lung cancer patients enrolled on the clinical trial NCT01255748

#### Gilmer Valdes^1^, Tomi Nano^1^, Jessica Scholey^1^, Charles Simmone^2^

##### ^1^University of California- San Francisco, Radiation Oncology, San Francisco, USA ^2^New York Proton Center, Radiation Oncology, New York, USA

**Purpose**: To predict the probability of grade ≥2 pneumonitis or dyspnea within 12 months of receiving proton therapy (PBT).

**Methods**: Consecutive patients enrolled and treated with PBT from 17 institutions were evaluated for grade 2 pulmonary toxicities. Both demographic and treatment characteristics were studied. Balanced Accuracy (BA) and Area under the Curve (AUC) were used as the main metrics.

**Results**: From the 965 patients studied, 256 (28.2%) had grade ≥2 pulmonary toxicity (age 70 (8-97) years, 85% with grade 3 or higher, 46.4% received concurrent chemo, median prescription dose 60 Gy and median dose per fraction 2 Gy/fraction). The most important demographic and dosimetric variables associated to the toxicities (p <0.05) were weight, and age. The probability of pulmonary toxicity was 0.08 among the centers treating primarily with PBS and 0.34 from those treating with older techniques (p <0.01). Abdominal compression resulted to be highly significant as well (p <0.01). As expected, higher total radiation delivered dose and dose to the ipsilateral lung increased the likelihood of pulmonary toxicities. When we combined demographic with dosimetric features, an AUC 0.69 ± 0.024 was obtained. After analyzing performance versus the number of data points we observed that accuracy is still limited by the number of observations.

**Conclusion**: In this large analysis of prospectively enrolled patients with lung cancer, advanced machine learning methods have identified that PBS, abdominal compression, and reduction of the dose received by the normal lung can lead to significantly smaller probability of developing grade ≥2 pneumonitis or dyspnea.

### O 049 - Estimating the risk of developing hypothyroidism in comprehensive breast cancer patients with sensitivity analysis computed from patient-setup and proton-range uncertainties

#### Jen Yu^1^, Marcio Fagundes^1^, Andrew Wroe^1^, Minesh Mehta^1^, Alonso Gutierrez^1^

##### ^1^Miami Cancer Institute, Proton Therapy- Radiation Oncology, Miami, USA

**Purpose**: Utilizing a photon-based normal tissue complication probability (NTCP) model, we estimated the probability of comprehensive breast cancer patients developing hypothyroidism (HT) 5-years post intensity modulated proton therapy (IMPT) and performed a sensitivity analysis of the occurrence of this complication based on patient-setup and proton-range uncertainties.

**Methods**: The study included fifty breast cancer patients who received comprehensive IMPT to 50.4Gy. Two en-face beams, single-field-optimization and Monte Carlo algorithm were used for planning. Plan robustness was evaluated with 5mm setup errors and 3.5% range uncertainties. The calculated NTCP was correlated with the total thyroid volume. NTCP error bars were derived from the thyroid dose variations induced by the setup and range uncertainties. NTCP population distributions were also analyzed.

**Results**: NTCP was found to decrease with increasing total thyroid volume (Fig. 1). For larger thyroids (>8.5cc), the average NTCP was 16.7±2.2%. For smaller thyroids (≤8.5cc), the average NTCP was 39.5%, with a range of +8.1%, −8.9%. For all patients with smaller thyroids, the HT risk was more than 15%. For patients with larger thyroids, only 43% of them were estimated to have HT higher than 15%. Patient setup and range uncertainties introduce a variation in the risk estimation, which is about 8% and 2% for smaller and larger thyroids, respectively. Fig. 2(a)-(c) shows the NTCP distribution and the box plots. Fig. 2(d) summarizes the data.

**Conclusion**: This work indicates that the thyroid volume is a strong indicator for HT. The comprehensive breast cancer patients would benefit from proactive HT screening and planning techniques that reduce thyroid dose.

### O 050 - IMPT does not result in higher rates of radiation ulcers in oropharyngeal cancer

#### Anne van den Hoek^1^, Stefan Both^1^, Jeffrey Free^1^, Edwin Oldehinkel^1^, Hans Verbeek^1^, Dan Scandurra^1^, Roel Steenbakkers^1^, Tineke Meijer^1^, Hans Langendijk^1^

##### ^1^The University Medical Center Groningen UMCG, Radiation Oncology, Groningen, Netherlands

**Introduction**: A recent study found evidence of increased PET-detected mucosal reactions associated with variable RBE in head and neck cancer patient raising the question if this could lead to higher rates of mucosal radiation ulcers after IMPT compared to IMRT/VMAT. Therefore, the aim of this study was to investigate if the cumulative incidence of radiation ulcers after IMPT was higher than after IMRT/VMAT.

**Materials and Methods**: The study population was composed of 581 oropharyngeal cancer (OPC) patients treated with definitive radiotherapy, chemoradiation. In total, 443 patients were treated with IMRT or VMAT. Since January 2018, 138 out of 202 patients (68%) qualified for IMPT according to model-based selection. All patients were included in a prospective data registration program in which acute and late toxicity was prospectively assessed before, during and after RT. The primary endpoint was the cumulative incidence of radiation ulcers at 24 months. Univariate and multivariate analysis was performed using Kaplan-Meier method and the Cox proportional hazard model, respectively.

**Results**: After 24 months, the cumulative incidence of radiation ulcers was 11.5% after IMRT/VMAT versus 12.9% after IMPT (p=0.865) (Figure 1). No difference was noted in the distribution of toxicity grading (Figure 1). In the multivariate analysis, T-stage (UICCv7), smoking status and growth pattern were identified as independent prognostic factors (Table 1). IMPT was not associated with the cumulative incidence of radiation ulcers.

**Conclusion**: In this large prospective cohort study of OPC patients, IMPT was not a risk factor for the development of radiation ulcers.

### O 051 - Unraveling the radiobiological effects of CIRT on vaginal mucosal melanoma cells using 2D and 3D models

#### Alexandra Charalampopoulou^1,2^, Amelia Barcellini^3,4^, Giovanni Battista Ivaldi^5^, Giuseppe Magro^6^, Marco Liotta^7^, Ester Orlandi^3^, Marco Giuseppe Pullia^8^, Paola Tabarelli De Fatis^7^, Francesco Moccia^9^, Angelica Facoetti^1^

##### ^1^National Center for Oncological Hadrontherapy CNAO, Radiobiology, Pavia, Italy ^2^Istituto Universitario di Studi Superiori IUSS, Biology, Pavia, Italy ^3^National Center for Oncological Hadrontherapy CNAO, Clinical, Pavia, Italy ^4^University of Pavia, Internal Medicine and Medical Therapy, Pavia, Italy ^5^Istituti Clinici Scientifici Maugeri IRCCS, Radiation Oncology, Pavia, Italy ^6^National Center for Oncological Hadrontherapy CNAO, Medical Physics, Pavia, Italy ^7^Istituti Clinici Scientifici Maugeri IRCCS, Medical Physics, Pavia, Italy ^8^National Center for Oncological Hadrontherapy CNAO, Research and development, Pavia, Italy ^9^University of Pavia, Biology and Biotechnology, Pavia, Italy

**Background**: Primary vaginal mucosal melanomas are characterized by intrinsic radio-chemoresistance and neurotropism. C-ion radiotherapy (CIRT) has been proven to be effective in its treatment, but there is still a lack of data concerning its different radiobiological effects compared to photons (XRT). This study aims to comprehensively investigate the influence of C-ions on some key aspects of this tumour including survival, proliferation, melanin synthesis, migration through PNI and PD-L1 expression through 2D cultures and 3D biological scaffolds.

**Methods**: HMV-II cells were irradiated with different doses (range=1-10 Gy) of XRT and C-ions. Cell survival was assessed by the clonogenic survival assay, viability with the automated cell counter LUNA-II and proliferation with the Olympus Provi^TM^ CM20 incubation monitoring system in time intervals of 24h. The scratch and transwell migration assays evaluated cell migration and NT-3 was added to test PNI. Dendrite length was measured and melanin production was assessed also after the regulation of Ca^2+^ signalling by inhibiting the SOCE channel. ELISA assay was performed to measure PD-L1 expression.

**Results**: Compared to XRT, both in 2D and 3D, CIRT reduced more significantly cell survival (P<0.002), proliferation (P<0.005) and migration (P<0.001), while NT-3 increased this general trend (P<0.002). We observed an increase in dendritic length (P<0.001) and melanin production after the irradiation, but an inversion of this trend with the inhibition of SOCE channel (P<0.003). CIRT upregulated the PD-L1 expression (P<0.002).

**Conclusions**: CIRT showed to affect significantly the behaviour of mucosal melanoma cells, both in terms of magnitude and molecular pathways, representing a promising strategy in clinical oncological research.

### O 052 - Single cell analysis of microglia activation status to dose and LET in proton-irradiated mouse brains

#### Sindi Nexhipi^1,2^, Theresa Suckert^1,3,4^, Marc Boucsein^5,6^, Johannes Soltwedel^1,2,7^, Elke Beyreuther^1,8^, Emanuel Bahn^5,6,9,10^, Mechthild Krause^1,2,3,11,12^, Armin Lühr^1,2,3,13^, Antje Dietrich^1,3^

##### ^1^OncoRay – National Center for Radiation Research in Oncology, Faculty of Medicine and University Hospital Carl Gustav Carus- TU Dresden, Dresden, Germany ^2^Helmholtz-Zentrum Dresden-Rossendorf, Institute of Radiooncology – OncoRay, Dresden, Germany ^3^German Cancer Consortium DKTK- Partner Site Dresden, German Cancer Research Center DKFZ, Dresden, Germany ^4^Institute for Research in Biomedicine IRB Barcelona-, Barcelona Institute of Science and Technology BIST, Dresden, Germany ^5^Clinical Cooperation Unit Radiation Oncology, German Cancer Research Center DKFZ, Heidelberg, Germany ^6^Department of Radiation Oncology, Heidelberg University Hospital, Heidelberg, Germany ^7^DFG Cluster of Excellence Physics of Life, TU Dresden, Dresden, Germany ^8^Helmholtz-Zentrum Dresden - Rossendorf, Institute of Radiation Physics, Dresden, Germany ^9^National Center for Tumor Diseases NCT, Partner Site Heidelberg, Heidelberg, Germany ^10^Heidelberg Institute of Radiation Oncology HIRO, Heidelberg, Heidelberg, Germany ^11^Department of Radiotherapy and Radiation Oncology, Faculty of Medicine and University Hospital Carl Gustav Carus- TU Dresden, Dresden, Germany ^12^National Center for Tumor Diseases NCT, Partner Site Dresden, Dresden, Germany ^13^Medical Physics and Radiotherapy, Faculty of Physics- TU Dortmund University, Dortmund, Germany

Variable relative biological effectiveness (RBE) and ventricular proximity are shown to impact appearance of late side effects in patients treated with cranial proton therapy. To study these parameters, we quantified neuroinflammation based on the activation status of the brain immune cells, microglia. This endpoint was evaluated in a mouse model of proton irradiation of the right hippocampus with single doses between 40 and 85 Gy. Brains were excised within 6 months post-radiation, cut axially into ∼30 slices per brain (with 100 µm intervals) and stained against Iba1 and DAPI for microglia and nuclei detection, respectively. An in-house algorithm segmented microglia and retrieved information on their distance to ventricles, as well as morphological parameters used to calculate the activation status, M-Score. The immunofluorescence images were co-registered to the planning computed tomography (CT) and corresponding Monte-Carlo dose and linear energy transfer (LET) calculations (TOPAS). We then performed section-based histo-cytometry to investigate single-cell activation response in relation to spatial parameters. This framework allows the analysis of the activation status of all microglia in the sections and the correlation of the status with the cells’ ventricular distance, dose and LET. The population-based analyses in whole mouse brains revealed a clear dose-dependent increase of microglia activation. Cells with a high M-Score (i.e., high activation status) are more prevalent in the periventricular region, the dose maximum area, and the distal beam edge (Fig.1). The latter might point towards a variable proton RBE and our data supports microglia inflammation as potential biomarker for radiation-induced brain damage.

### O 053 - Cerebral organoid slices cultured at air-liquid interface model CNS effects of particle radiotherapy

#### Tamara Bender^1^, Esther Schickel^1^, Timo Smit^1^, Kim Knorr^1^, David Grosshans^2^, Marco Durante^1^, Insa S. Schroeder^1^

##### ^1^GSI Helmholtz Centre for Heavy Ion Research, Biophysics, Darmstadt, Germany ^2^The University of Texas MD Anderson Cancer Center, Radiation Oncology, Houston, USA

Ionizing radiation plays a central role in the management of glioblastoma (GBM) for which no effective therapy is available. Although photon therapy is standard care for GBM, less frequently applied carbon ions may have potential to offer improved disease control rates. However, radiation can induce brain necrosis and increased tumor aggressiveness in the recurrence. We studied the responses of cerebral organoid slices to different radiation qualities. Tumor organoids were generated by overexpression of oncogenes and deletion of tumor suppressor genes. Single GBM-like cells isolated from these organoids were co-cultured with sister organoid tissue slices maintained at air-liquid interface (ALI) and irradiated with 15 Gy X-rays or ^12^C ions in the SOBP. Changes in the cell marker expression were evaluated using qPCR and immunofluorescence staining. ALI-culture enabled long-term maintenance and showed no signs of necrosis in comparison to whole organoids. GBM-like cells displayed tumor-like properties, i.e., overexpression of oncogenes and downregulation of tumor suppressor genes, increased proliferation and invasive potential, which resulted in their overgrowing the organoid slices. Finally, radiation, depending on quality, led to changes in the cell composition, myelination status, proliferative and invasive potential. GBM-like cells, which survived radiation, showed high expression of markers of invasive potential (ADAM10 and MMP2) as commonly observed in GBM patients. This project is supported by the Federal Ministry of Education and Research (02 NUK 049A) and the NIH grant 1RO1CA256848-01. Irradiation was performed at the beam line/infrastructure HTA at the GSI Helmholtzzentrum fuer Schwerionenforschung, Darmstadt (Germany) in the frame of FAIR Phase-0 (SBio08_Schroeder).

### O 054 - Characterising dependencies on specific DSB repair pathways in response to different radiation qualities

#### Francisco Liberal^1^, Stephen McMahon^1^

##### ^1^Queen’s University Belfast, The Patrick G Johnston Centre for Cancer Research, Belfast, United Kingdom

Particle therapy offers significant benefits for the physical precision of radiotherapy. However, there remains a lack of biological optimization, particularly around how the RBE of different qualities of radiation varies between patients, which would ultimately enable better allocation of these expensive therapies. CRISPR-Cas9 genetically modified normal human retinal epithelial cell lines (RPE-1) with defects in different DNA repair genes were used to identify and characterize dependencies on specific DNA repair pathways in response to different qualities of ionizing radiation. The clonogenic assay was used to measure radiation efficacy, and 53BP1 immunofluorescence foci were used to quantify irradiation induced DSBs and their repair kinetics after exposure to photons, protons and alpha-particles. NHEJ-defective cells proved to be markedly hypersensitive across all tested radiation sources. Due this hypersensitivity, the impact of NHEJ knockout on survival decreases with increasing incident radiation LET, potentially due to overkilling effects. HR-defective cells had moderately increased sensitivity across all tested radiation sources. Notably, the contribution of HR pathway to survival appears independent of LET in this study (Fig.1). Corroborating our survival data, NHEJ-defective cells had the least DSB repair after low LET exposure (Fig.2A), and no visible repair after high LET exposure (Fig.2B). HR-defective cells also had slower DSB repair kinetics, but its impact is not as severe as NHEJ. These results suggest that the major DNA repair pathway is NHEJ, independent of LET, and in these genetically modified cell lines, no increased dependence on HR is seen with increasing LET.

### O 055 - Towards automatic treatment verification with prompt-gamma-imaging: CNN-based detection of anatomical changes in prostate-cancer proton therapy

#### Julian Pietsch^1,2^, Jonathan Berthold^1,2^, Julia Thiele^3^, Tobias Hölscher^3^, Erik Traneus^4^, Guillaume Janssens^5^, Julien Smeets^5^, Kristin Stützer^1,2^, Steffen Löck^1,3,6^, Christian Richter^1,2,3,6^

##### ^1^OncoRay – National Center for Radiation Research in Oncology, Faculty of Medicine and University Hospital Carl Gustav Carus- Technische Universität Dresden- Helmholtz-Zentrum Dresden - Rossendorf, Dresden, Germany ^2^Helmholtz-Zentrum Dresden - Rossendorf, Institute of Radiooncology - OncoRay, Dresden, Germany ^3^Department of Radiotherapy and Radiation Oncology, Faculty of Medicine and University Hospital Carl Gustav Carus- Technische Universität Dresden, Dresden, Germany ^4^RaySearch Laboratories AB, Research, Stockholm, Sweden ^5^Ion Beam Applications SA, Research, Louvain-la-Neuve, Belgium ^6^German Cancer Consortium DKTK- partner site Dresden, Germany and German Cancer Research Center DKFZ, Heidelberg, Germany

**Introduction**: We present the worldwide first results for the automatic evaluation of clinical prompt-gamma-imaging (PGI) data using convolutional neural networks (CNNs) to detect anatomical changes in prostate-cancer proton treatments.

**Materials and Methods**: Spot-wise range shifts were monitored with a PGI-slit-camera during 192 field deliveries of prostate-cancer treatments (15 patients, 1.5 Gy/field) with prior in-room control CT acquisition in treatment position. Corresponding spot-wise shifts of integrated depth-dose profiles between planning and control CTs were utilized for field-wise ground truth classifications on the dose level: Treatment fields were classified as being affected by anatomical changes of the patient (change) or not (no change). Two 16x16x16 input channels, containing the averaged PGI range shift and the respective summed proton number (Fig.1), were generated for training 3D-CNNs to detect anatomical changes using patient-wise leave-one-out cross-validation.

**Results**: Anatomical deviations in rectum filling, femur orientation, and/or lateral soft tissue distribution induced dose changes, mostly below current clinical intervention thresholds, in 96 fields (50%). The CNNs achieved training and validation accuracies of 0.85 (range: 0.75-0.89) and 0.85 (0.63-1.00), respectively (Fig.2). Using only PGI information, relevantly affected fields were detected with a sensitivity of 0.83 and a specificity of 0.88 in the validation data. A final evaluation on an already identified independent test cohort (10 patients) will be presented.

**Conclusion**: Our study shows that CNNs can reliably detect dosimetrically relevant anatomical changes from clinical PGI data of prostate-cancer patients and highlights the potential of a routine clinical application of PGI in an automated feedback loop for online adaptive proton therapy.

### O 056 - Feasibility of low dose CT in adaptive proton therapy

#### Sydney Shindler^1^, Tong Zhu^1^, Arash Darafsheh^1^, Stephanie Perkins^1^, Tianyu Zhao^1^, Tiezhi Zhang^1^, Yao Hao^1^

##### ^1^Washington University School of Medicine, Department of Radiation Oncology, St Louis, USA

**Purpose**: In adaptive proton therapy, imaging is imperative for daily patient setup, monitoring anatomical changes, dose prediction, and replanning. However, cone-beam computed tomography (CBCT) usage is limited by artifacts and inaccurate Hounsfield values (HU). In-room CT resolves these limitations but presents a large accumulated dose across multiple fractions. This work demonstrated the feasibility of using low dose CT images for online adaptation and daily alignment.

**Methods**: Two quality assurance and three anatomical phantoms were imaged using eighteen different scan techniques. Ten proton therapy patients were included in this study. Patients were imaged daily using different techniques: normal dose (conventional) CT scan and two levels of low dose CT scans. Regions of interest were contoured on conventional CT and rigidly copied into low dose CTs. Signal-to-noise ratios (SNR) were investigated. Treatment plans were generated in RayStation 11A to study dosimetric impact.

**Results**: With decreasing imaging dose, SNR was reduced for all the patients as shown in the table. Both target coverage and organs at risk sparing of phantoms and patients without obvious anatomy variation show unnoticeable changes between conventional, low dose, and extremely low dose CT scans (6% of conventional scan’s CT dose index). Dosimetric comparisons are shown in the figure. CTDIvol are about 42.5mGy, 7.1mGy, and 3.5mGy for 300mAs, 50mAs, and 25mAs scans.

**Conclusions**: This work supported the use of low dose CT scans for daily alignment as well as replanning in adaptive proton therapy. Optimized low imaging dose should be determined based on each institution’s practice and treatment sites.

### O 057 - Comparison of 4D beam delivery methods for pancreatic cancer based on time-resolved 3DCT-MRI images

#### Timo Steinsberger^1^, Anestis Nakas^2^, Maria Chiara Martire^1^, Lennart Volz^1^, Alessandro Vai^3^, Mario Ciocca^3^, Viviana Vitolo^4^, Guido Baroni^2^, Chiara Paganelli^2^, Christian Graeff^1^

##### ^1^GSI Helmholtzzentrum für Schwerionenforschung GmbH, Biophysics, Darmstadt, Germany ^2^Politecnico di Milano, Dipartimento di Elettronica- Informazione e Bioingegneria, Milano, Italy ^3^Fondazione CNAO, Department of Medical Physics, Pavia, Italy ^4^Fondazione CNAO, Radiotherapy department, Pavia, Italy

**Purpose**: To compare the performance of different motion mitigation techniques for realistic, non-periodic motion in carbon ion therapy (CIRT) relying on time-resolved tr3D CT-MRI data.

**Methods**: Planning 4DCT (8 respiratory phases reconstructed retrospectively) of a patient with pancreatic tumor (range of motion 4.2 mm) treated with CIRT were acquired at CNAO, Italy. 18 respiratory cycles of tr3D CT-MRI data with 8 phases each were generated from 2D cine-MRI images (3T, 1.15 minutes) acquired the same day as proposed by Meschini et al. 2022 (https://doi.org/10.1002/mp.15510). Treatment plans (two fields, 4.8 Gy(RBE)) were created in TRiP98 aiming for target D95>95% and V75<1% in the duodenum using simultaneous robust optimization on all 4DCT phases (4DITV), optional lateral beam tracking (4DtITV) and increased spot weights for 8x layered rescanning/retracking. Plan libraries covering all 4DCT phases were created using the single phase uniform dose (SPUD, figure 1) approach for multi-phase 4D delivery (MP4D) with optional residual tracking (MP4DRT). The rescanning and retracking plans were also delivered with MP4D and MP4DRT to enforce breath-sampling (BS). Planned (4DD) and delivered (4DDD) dose were calculated on the 4DCT and the tr3DCT-MRI series, respectively.

**Results**: Target D95, V75 in duodenum, and delivery time are listed in table 1.

**Conclusions**: Real-time adaptive MP4DRT and lateral beam retracking with sufficient number of rescans lead to conformal dose distributions with a slight trade-off between conformity (MP4DRT) versus robustness and treatment time (retracking), both out-performing ITV deliveries and MP4D alone. The study will be expanded to more patients and verified experimentally.

### O 058 - Comparison of respiratory motion characteristics between upright and horizontal positioning using an upright open MR: A preliminary study

#### Ye Zhang^1^, Alisha Duetschler^1,2^, Andrea Serra^3^, Marco Battiston^3^, Filippo Grillo Ruggieri^1^, Damien Weber^1,4,5^, Antony Lomax^1,2^

##### ^1^Paul Scherrer Institut, Center for Proton Therapy, Villigen-PSI, Switzerland ^2^ETH Zurich, Department of Physics, Zurich, Switzerland ^3^ASG Superconductors SpA, Paramed MRI Unit, Genova, Italy ^4^Inselspital- Bern University Hospital- University of Bern, Department of Radiation Oncology, Bern, Switzerland ^5^University Hospital of Zürich, Department of Radiation Oncology, Zurich, Switzerland

**Purpose**: Due to growing interest in upright patient positioning for proton therapy, we compare respiratory motion amplitudes, reproducibility and drift between upright and supine patient positioning.

**Methods**: An upright 0.5T MR (MROPEN_EVO, ASG, Italy) was used for acquiring time-resolved 2D cine-MR images (HASTE, 2.57 × 1.64 mm) of two volunteers in both upright and supine orientations (Fig1ad). A sagittal plane covering the thoracic and upper abdomen was selected by ensuring the diaphragm dome was consistently imaged for both orientations (Fig1be). Continuous cine-MRs (6min, 1.97Hz) were collected over 4 sessions with short pauses (4min) in between, for a total duration of >30min. The location of the apex of the diaphragm dome was extracted using edge detection (Fig1cf), and then used as a surrogate for comparing motion characteristics of the two orientations for the duration of acquisition.

**Results**: For both subjects, motion amplitude and variability are smaller (Fig2a) and more stable (Fig2b) for upright compared to supine positioning. Violin plots in Fig2c show normalised motion amplitudes (to the overall mean), in which more skewed distributions in the superior direction were observed for the supine scenario (yellow), which are more limited for upright (green), indicating that baseline drift may be less pronounced for upright positioning.

**Conclusion**: We have performed the first comparative imaging studies of respiratory motion variability between upright and supine positioning using an open MRI. Preliminary results show pronounced differences in motion between the two orientations. However, systematic investigations with a larger population group will be pursued to confirm these initial observations.

### O 059 - Toward adaptive proton radiotherapy: Automated pre-treatment dose verification of spot-scanned plans using sliding gantry CT localization images

#### Sharareh Fakhraei^1^, Daniel Mundy^1^, Erik Tryggestad^1^, Jon Kruse^1^, Jedediah Johnson^1^, Srinivas Seetamsetty^1^, Thomas Barrett^1^, Hok Seum Wan Chan Tseung^1^, Scott Lester^1^, Ryan Phillips^1^

##### ^1^Mayo Clinic, Radiation Oncology, Rochester, USA

**Background**: Proton radiotherapy dose distributions are sensitive to anatomical changes and setup deviations. Routine verification of CT scans and dose calculations ensure continuing plan fidelity throughout a treatment course; however, the process is time-intensive and may not reflect the actual anatomy and patient alignment during treatment.

**Method**: Two treatment rooms at our institution feature sliding gantry CT-on-Rails (CToR) scanners, used for either pre-treatment volumetric localization or post-treatment verification scans. CToR image acquisition triggers an automated verification process that sends the CToR image and spatial registration object (SRO), representing patient alignment at treatment time, to commercial image processing software. An automated workflow aligns the CToR image to the reference CT using the SRO, generates the registered verification image, propagates rigid and deformed contours to this new image, and exports the corresponding DICOM objects back to the planning system for automated dose calculation. Monte Carlo physical and biological doses are also calculated using an in-house system.

**Results**: For both a complex and simple head-and-neck plan, the times required for a dosimetrist to complete the verification workflows were 53 and 32 minutes, respectively. The automated workflow completed the same tasks in 15 and 11 minutes. Further optimization is in progress. A user interface has been developed to monitor the status of automated verifications.

**Conclusion**: We have clinically implemented a fully automated verification workflow for proton therapy that allows for efficient off-line plan verification as well as prospective pre-treatment evaluation of dose. This is the first step in an online adaptive proton therapy process.

### O 060 - Proton radiography with a multi-layer strip ionization chamber device

#### Shuang Zhou^1^, Jonathan Haefner^1^, Nathan Harrison^2^, Alexander Stanforth^2^, Liyong Lin^2^, Xinyuan Chen^1^, Tianyu Zhao^1^, Tiezhi Zhang^1^

##### ^1^Washington University in St. Louis, Radiation Oncology, St. Louis, USA ^2^Emory University, Radiation Oncology, Atlanta, USA

**Purpose**: Proton radiography is important for direct measurement of proton stopping power ratio. We developed a novel multi-layer strip ionization chamber (MLSIC) device that can be used to acquire proton radiographic images by scanning a proton pencil beam.

**Methods**: Originally designed for IMPT QA, the MLSIC device comprises 66 layers of strip ionization chambers in every 2.9 mm water equivalent thickness (WET). The spatial resolution is 2 mm and maximum measurable field size is 25 cm. A total of 768 channels of ionization currents were sampled by a data acquisition (DAC) device at frequencies up to 3.67kHz. A head phantom and a Gammex electron density phantom were imaged with a Varian ProBeam. Single energy layer plans with 20 by 20 cm^2^ field size and 2-mm spot spacing was delivered with 1 nA nozzle current. The proton spot dose profile and integral depth dose were derived from the raw data. Residual ranges of the exit proton beams were estimated using fits with an empirical model. Proton WET images were acquired from proton range data. Proton stopping power ratio readout from WET image was compared to IBA ZEBRA measurements.

**Results**: Proton radiographic images can be precisely measured by our MLSIC with a single proton beam delivery. The proton stopping power ratio measured from WET images was -0.76% less than the known value on average, with a standard deviation of 0.28%.

**Conclusion**: MLSIC can be used to measure proton radiographic image and for direct proton stopping power ratio measurement.

### O 061 - Real-time in vivo range verification for proton therapy based on N-12 imaging

#### Brian Zapien Campos^1^, Zahra Ahmadi Ganjeh^1^, Stefan Both^2^, Peter Dendooven^1^

##### ^1^University Medical Center Groningen- University of Groningen, Particle Therapy Research Center, Groningen, Netherlands ^2^University Medical Center Groningen, Groningen Proton Therapy Center, Groningen, Netherlands

A current problem in proton therapy is the absence of reliable and accurate methods to measure the proton range in vivo and in real-time during irradiation. Imaging of positron emitters produced in the patient by the proton beam can be used for this purpose. Next to long-lived positron emitters (mostly C-11, O-15, and C-10), the very short-lived positron emitter N-12 (half-life 11ms) is produced and has the potential to be used for real-time verification of the proton range. In this contribution, the latest results in the development of N-12 imaging are presented. At the Particle Therapy Research Center (PARTREC), a modified dual-head Siemens Biograph mCT PET scanner was used to image the beam-induced activity on homogeneous graphite and PMMA targets, and a 731-HN phantom. The irradiation plan consisted of pulsed pencil-beam (pulse width of 5 ms and periods of 100 ms) of 66.5 and 150 MeV (1E8 protons per pulse) and a PET data acquisition for 660 s. The N-12 images were calculated by subtracting the late image (time window from 50 to 94 ms) from the early image (from 4 to 49 ms). Our results proved to be satisfactory to measure the N-12 cross-section with uncertainty between 3-5%. Furthermore, N-12 images of the head phantom show promise for accurately measuring the proton range in real-time.

### O 062 - Investigating the feasibility of patient positioning based on a helium-beam radiograph (αRAD) acquired with thin silicon pixel detectors

#### Yanting Xu^1,2,3^, Margareta Metzner^1,2,3^, Daria Zhevachevska^1,2,4^, Annika Schlechter^1,2,3^, Stephen Schaumann^5^, Ralf Omar Floca^2,5,6^, Benjamin Ackermann^7^, Oliver Jäkel^1,2,6,7^, Mária Martišíková^1,2^, Tim Gehrke^1,2,6^

##### ^1^German Cancer Research Center DKFZ, Department of Medical Physics in Radiation Oncology, Heidelberg, Germany ^2^National Center for Radiation Research in Oncology NCRO, Heidelberg Institute for Radiation Oncology HIRO, Heidelberg, Germany ^3^Heidelberg University, Faculty of Physics and Astronomy, Heidelberg, Germany ^4^Heidelberg University, Medical Faculty Mannheim, Heidelberg, Germany ^5^German Cancer Research Center DKFZ, Medical Image Computing, Heidelberg, Germany ^6^Heidelberg University Hospital, Radiation Oncology, Heidelberg, Germany ^7^Heidelberg Ion-Beam Therapy Center HIT, Radiation Oncology- Heidelberg University Hospital, Heidelberg, Germany

Ion-beam radiography offers the potential to make ion-beam radiotherapy more precise. It is a promising technique to verify the water-equivalent thickness (WET) map based on the X-ray CT which is used to plan the treatment. Therefore, errors due to e.g. anatomical changes could be detected prior to each treatment fraction, which is why ion radiography is a promising alternative to the clinically used X-ray projections for positioning. In this contribution, the possibility of patient positioning by means of helium-beam radiography is assessed for an anthropomorphic head phantom. For the acquisition of helium-beam radiographs, an imaging system based on thin silicon pixel detectors, called Timepix, was used at the Heidelberg ion-beam therapy center to track ions at low fluence rates of helium ions. The setup consists of a front and rear tracker and a detector measuring the energy deposition of single ions. The energy deposition was connected to water-equivalent thickness information using newly established signal calibrations. Two radiographs of an anthropomorphic head phantom were acquired with identical parameters apart from an intentionally introduced rotation of the phantom by 1°. Then, the two radiographs were independently registered to an X-ray CT using a 2D-to-3D in-house image registration algorithm. Finally, the difference in the resulting translation and rotation vectors were compared to the ground truth rotation of 1°. In this way, an accuracy of 0.27° and precision of 0.32° for rotations and an accuracy and precision of both 0.06 mm for translations were found, revealing a real potential for clinical use.

### O 063 - Proton radiography using a novel integrating acquisition and fast imaging method for image guidance

#### Sam Beddar^1,2^, Mikaël Simard^3^, Ryan Fullarton^3^, Daniel Robertson^4^

##### ^1^The University of Texas MD Anderson Cancer Center, Radiation Physics, Houston- TX, USA ^2^The University of Texas, Graduate School of Biomedical Sciences at Houston, Houston- TX, USA ^3^University College London, Department of Medical Physics and Biomedical Engineering, London, United Kingdom ^4^Mayo Clinic Arizona, Division of Medical Physics, Phoenix- AZ, USA

The purpose of this project is to construct and characterize a practical, cost-effective proton radiography system suitable for clinical use and without needing to modify clinical beamlines. We are using a novel integrating detector acquisition method as opposed to single-particle tracking approaches that employs trackers placed in front/behind the patient. Our developed system consists of a large monolithic solid detector placed behind the object to be viewed by three high-speed camera systems to capture the light from multiple directions. The distal camera, provides a beam eye of the object. The two lateral cameras provide additional information of the proton beam penetration through the object which can be used to improve the image quality and performance of the final radiograph. The imaging system was tested for uniformity, stability, linearity, and accuracy in measuring water-equivalent thickness (WET). A custom Las Vegas phantom was radiographed for qualitative assessment of the system’s performance. Other characteristics of our imaging system preliminary results will be presented, including proton radiographs of adult and pediatric phantom head. Finally, potential use of this system for image guidance for proton therapy patients will be briefly discussed. In addition, by enabling the online verification of patients’ WETs, this system will facilitate the detection of patient anatomy changes that would/could trigger adaptive radiotherapy re-planning.

### O 064 - Novel high-density glass scintillators for proton radiography

#### Ethan Stolen^1^, Lincoln Houghton^2^, Charles-Antoine Collins Fekete^3^, Sam Beddar^4^, Ugur Akgun^5^, Daniel Robertson^6^

##### ^1^University of Florida, Department of Physics, Gainesville, USA ^2^Mayo Clinic, Radiation Oncology, Phoenix, USA ^3^University College London, Medical Physics & Biomedical Engineering, London, United Kingdom ^4^The University of Texas MD Anderson Cancer Center, Radiation Oncology, Houston, USA ^5^Coe College, Department of Physics, Cedar Rapids, USA ^6^Mayo Clinic Arizona, Radiation Oncology, Phoenix, USA

The development of proton radiography promises several advantages, including improved image guidance for adaptive radiation therapy and reduction in proton stopping power uncertainties. Proton radiography detectors require large scintillator volumes, which can be bulky and expensive. This study aims to develop a high-density scintillating glass that can be used in proton radiography detectors, resulting in a more affordable, compact detector. We synthesized five different activated glass scintillator samples by adding different ratios of europium (III) oxide or terbium (III) oxide to three base glasses, including various percentages of tungsten (III) oxide to increase the density. A flat panel scintillator detector was modified to house powdered samples of the glass scintillator materials and a plane-parallel ionization chamber. The glasses’ response to high-energy protons were characterized by measuring their light output compared to anthracene, a common reference scintillator. Their ionization quenching was determined by comparing the dose, linear energy transfer (LET), and light output as a function of depth. LET was calculated using the TOPAS, Monte Carlo system. The results demonstrate ionization quenching and its correction based on Birks’s model, as seen in Figure 2 (b)-(f), with the Birks factors listed in Figure 1 (a). The samples exhibit the small light yield expected from glass scintillators, which may be worsened by the samples having poor transparency in powder form. The scintillation efficiency increased with activation element concentration until a threshold, then decreased due to concentration quenching. These results will be used to select the optimal scintillator for a full-size proton radiography detector.

### O 065 - Preoperative stereotactic radiosurgery: A novel diagnostic therapy strategy to inform patient selection for particle therapy

#### Daniel Trifiletti^1^, Jennifer Peterson^1^, Beatriz Fernandez-Gil^2^, Steven Herchko^1^, Yan Asmann^3^, Erik Middlebrooks^4^, Wendy Sherman^5^, Alfredo Quinones-Hinojosa^2^

##### ^1^Mayo Clinic, Radiation Oncology, Jacksonville, USA ^2^Mayo Clinic, Neurosurgery, Jacksonville, USA ^3^Mayo Clinic, Health Sciences Research, Jacksonville, USA ^4^Mayo Clinic, Radiology, Jacksonville, USA ^5^Mayo Clinic, Neurology, Jacksonville, USA

**Objective**: Patient-specific treatment decision-making is critical, and a “one size fits all” approach is no longer appropriate. Specifically, while appropriate patient selection is paramount, strategies to inform photon/proton/carbon radiotherapy selection are lacking. We propose a novel strategy that incorporates preoperative stereotactic radiosurgery (SRS) as a “diagnostic therapy” in patients with radiation-resistant tumors.

**Methods**: We outline our proposed methodology for patients with radiation-resistant tumors using photon, proton, and carbon SRS with glioblastoma as a prototypic radioresistant tumor. Tumor response is assessed using advanced techniques to inform adjuvant therapy.

**Results**: In glioblastoma, our strong multidisciplinary collaboration has confirmed the early feasibility of a technique (Figure 1) in which patients with presumed glioblastoma undergo stereotactic biopsy then preoperative SRS then gross total resection within 14 days. Blood, tumor, peritumoral tissue, and images are compared to pre-SRS samples. Histopathologic, hypoxic, tumor/serum mRNA, and tumor/serum CYTOF analyses are done to determine early treatment response including repair pathways activated as well as local and systemic immune responses. These results then directly inform adjuvant therapy including radiotherapy modality, dose, fractionation, radiosensitization, and immunotherapeutics including tumor-specific vaccines. Direct extension of this work including preoperative proton and carbon ion SRS cohorts for comparative analyses will launch soon.

**Conclusion**: This framework reconsiders how particle therapy is used in cancer therapy. Through this ‘diagnostic therapy’ strategy, appropriate patient, tumor, and treatment techniques are aligned to create an individualized plans for patients. Prospective clinical studies are needed and are currently underway in patients with newly diagnosed glioblastoma with expansions planned shortly (NCT05030298).

### O 066 - Upright, fixed beam, proton therapy for breast cancer: A volunteer positioning study using a rotating chair and specialised bras

#### Tracy Underwood^1,2^, Joanna McNamara^3^, Jemma Nunn^1^, Niek Schreuder^4^, Heidi Probst^3^

##### ^1^Leo Cancer Care, Research, Horley, United Kingdom ^2^University College London, Department of Medical Physics and Biomedical Engineering, London, United Kingdom ^3^Sheffield Hallam University, Radiotherapy, Sheffield, United Kingdom ^4^Leo Cancer Care, Research, Middleton, USA

**Purpose**: Gantry-free, upright proton therapy using fixed beams is attracting interest as it promises more cost-effective and compact treatment rooms. We investigated the feasibility of upright patient positioning for proton therapy of the breast, using a rotating chair (‘Eve’ from Leo Cancer Care). To reduce inframammary skin folds (ISF) & associated toxicity we tested the Chabner XRT® Radiation Bra (CIVCO Radiotherapy™) & the Support4All (S4A) bra (under development at Sheffield Hallam University).

**Methods**: After ethics board review, we recruited 7 healthy women, with bra cup-sizes³C. Anatomical markers enabled measurement of repositioning errors using optical cameras. Volunteers trialled an ‘arms-up’ position (using a Monarch wingboard from CIVCO Radiotherapy™) and an ‘arms-down’ position. The volunteers spent ∼1 hour positioned on Eve. Comfort was assessed using questionnaires.

**Results**: All volunteers reported that upright treatment positions felt comfortable, especially ‘arms-down’. Bras were viewed favourably. With no bra, the mean ISF measurement was 4.8cm (range 2.5cm:9cm). The thicker S4A bra eliminated the ISF for 7/7 women. The thinner Chabner XRT bra eliminated the ISF for 4/7 women. Breast repositioning errors varied from ∼1:10mm.

**Conclusion**: Upright proton therapy of the breast appears feasible: even for larger breasted women, RT bras are effective in reducing/eliminating ISF. Upright, patients’ lowered arms can be moved behind the breasts and the body, away from the treatment fields. ‘Arms-down’ upright treatment positions are likely to improve patient comfort, especially post-surgery. Further research is now underway on (1) reducing set-up errors by further stabilizing the upper body, (2) internal anatomy and (3) patient perspectives.

### O 067 - Proton beam therapy using bioabsorbable spacer placement for pediatric patients with bone and soft tissue sarcoma: The Kobe Proton Center experience

#### Yusuke Demizu^1^, Hikaru Kubota^1^, Masayuki Mima^1^, Nobuyoshi Fukumitsu^1^, Takeshi Suzuki^2^, Takumi Fukumoto^3^, Ryohei Sasaki^4^, Toshinori Soejima^1^

##### ^1^Hyogo Ion Beam Medical Center Kobe Proton Center, Radiation Oncology, Kobe, Japan ^2^Hyogo Ion Beam Medical Center Kobe Proton Center, Anesthesiology, Kobe, Japan ^3^Kobe University Graduate School of Medicine, Hepato-Biliary-Pancreatic Surgery, Kobe, Japan ^4^Kobe University Graduate School of Medicine, Radiation Oncology, Kobe, Japan

**Background**: Bone and soft tissue sarcomas (BSTSs) are relatively common in pediatric patients. In cases with a tumor adjacent to organs at risk such as the digestive tract, proton beam therapy (PBT) using bioabsorbable spacer (BAS) placement is useful; however, only a few have been reported. A smooth clinical flow from the surgical spacer placement (SSP) to the finish of PBT is crucial because BAS is absorbed gradually. We report our experience mainly of its feasibility at Kobe Proton Center.

**Methods**: Five pediatric BSTS patients have been treated with PBT using BAS placement. Patient characteristics and factors related to BAS were retrospectively analyzed.

**Results**: The median age was 10 years (range, 1-15). Ewing sarcoma in 3, mesenchymal chondrosarcoma in 1, and rhabdomyosarcoma in 1. Pelvis in 3, lumber spine in 1, and retroperitoneum in 1. PBT of 59.4 Gy (RBE) in 33 fractions (4 patients) or 41.4 Gy (RBE) in 23 fractions (1 patient) was used. Concurrent chemotherapy was administered in all patients. The median period from SSP to the initiation of PBT was 22 days (range, 21-23). The median period from SSP to the finish of PBT was 69 days (range, 57-71). BAS was useful and the planned PBT was completed in all patients. The median period from SSP to the disappearance of BAS was 140 days (range, 122-154). No patients experienced BAS-related adverse events.

**Conclusions**: PBT using BAS placement for pediatric BSTS patients was feasible and safe. Larger multicenter studies with a longer follow-up are warranted.

### O 068 - Non-invasive CArdiac RAdioablation for Ventricular Tachycardia (CARA-VT): Photons versus protons dosimetric comparison in the perspective of ECG gated treatments

#### Alfredo Mirandola^1^, Eleonora Rossi^1^, Luca Maria Colombo Gomez^2^, Adele Valentini^3^, Laura Mantovani^2^, Elisabetta Bonzano^4^, Viviana Vitolo^5^, Amelia Barcellini^5^, Ester Orlandi^5^, Mario Ciocca^1^

##### ^1^Fondazione CNAO, Medical Physics Department, Pavia, Italy ^2^IRCCS Fondazione Policlinico San Matteo, Medical Physics Department, Pavia, Italy ^3^IRCCS Fondazione Policlinico San Matteo, Radiology Department, Pavia, Italy ^4^IRCCS Fondazione Policlinico San Matteo, Radiotherapy Department, Pavia, Italy ^5^Fondazione CNAO, Radiotherapy Department, Pavia, Italy

**Introduction**: Ventricular tachycardia is a major public health problem and may cause sudden cardiac death. StereoTactic Arrhythmia Radioablation (STAR) is an emerging, not invasive approach, based on the delivery of a high dose (25 Gy) of radiation in a single fraction. The aim of this study was to assess the feasibility of STAR for both photons and protons, combining deep inspiration breath-hold (DIBH) and ECG gating.

**Method**: Three patients received two DIBH ECG gated CT scans with/without contrast medium. In order to minimise the dose to patients, the field of view (FOV) was restricted to the heart except for treatment planning CT images. The latter were acquired with an extended FOV to include the whole thorax. An invasive non-fluoroscopic intracardiac mapping, performed during a previous catheter ablation procedure, was used to define the target (CTV) for each cardiac phase. The target for the photon treatment was defined as the ITV from the diastolic and systolic phase targets. The proton plan was optimized on the CTV of the diastolic phase only, assuming to carry out the treatment with ECG gating. Photon plans were simulated with the Elekta Monaco TPS, using VMAT with optimized arc aperture. Proton planning was performed using the RayStation TPS, with robust optimization (±2mm/3%).

**Results**: The use of proton beams granted a significant reduction in both the maximum and mean dose to cardiac OARs, also improving both target coverage (V_95%_ +1,9%) and dose homogeneity (HI +2%). The implantable cardioverter-defibrillators did not interfere with the planning.

### O 069 - Clinical implementation of intensity modulated neutron therapy (IMNT) at the University of Washington Medical Center

#### Robert Emery^1^, Robert Stewart^1^, Adrienne Lehnert^2^, Marissa Kranz^1^, Eric Dorman^1^, Jon Jacky^1^, Upendra Parvathaneni^1^, Jay Liao^1^

##### ^1^UW Medical Center, Radiation Oncology, Seattle, USA ^2^UW Medical Center, Radiology, Seattle, USA

High linear energy transfer (LET) neutron therapy is uniquely beneficial in the treatment of select cancers. Since 1984, we have treated over 3,400 patients with 3D conformal neutron therapy (3DNRT). In October of 2022, we completed work to implement intensity modulated neutron therapy (IMNT). IMNT commissioning required major upgrades to the Clinical Neutron Therapy System (CNTS) as well as new technologies developed in-house for physics quality assurance (QA) and treatment planning. Modifications to the CNTS included upgrades to the dosimetry and leaf collimator controllers and an updated therapy control system based on the Experimental Physics and Industrial Control System (EPICS) toolkit. We worked with a commercial vendor to integrate neutron-specific scattering kernels, as well as other refinements, into their treatment planning system (TPS) to enable IMNT with the CNTS. An in-house-developed DICOM server is used to transfer plans from the TPS to the therapy control system. Pre-treatment physics QA includes ionization chamber measurements and a 2D gamma-analysis using a custom portal imaging system and Positron Emission Tomography (PET) for ^12^C(n,2n)^11^C reactions. Measured PET images are compared to Monte Carlo simulated fluence (decay) maps to confirm accurate field delivery. Clinical Impact: Target coverage is improved, and organ-at-risk (OAR) doses are reduced for IMNT compared to 3DNRT. A retrospective study of early OAR toxicity for our first 15 IMNT head and neck patients has been initiated to confirm treatment safety. For those patients that have historically benefited from 3DNRT, IMNT is rapidly becoming our new standard of care.

### O 070 - Ultra-high dose rate SOBP carbon ion irradiation: In vitro and in vivo experiments

#### Walter Tinganelli^1^, Uli Weber^2^, Anggraeini Puspitasari^2^, Alexander Helm^2^, Sylvie Lerchl^2^, Olga Sokol^2^, Palma Simoniello^3^, Christoph Schuy^2^, Claudia Fournier^2^, Marco Durante^2^

##### ^1^GSI Helmholtzzentrum für Schwerionenforschung, Biopyhsics, Darmstadt, Germany ^2^GSI, Biophysics, Darmstadt, Germany ^3^University of Naples Parthenope, Science and Technology, Naples, Italy

Radiotherapy is crucial in cancer treatment since more than 50% of patients receive it throughout their treatment. The efficacy of radiation treatment has a limitation due to the toxicity in healthy tissue. High doses of radiation given in a short time, with ultra-high dose rates, are less harmful to healthy tissue but just as efficient as conventional dose rate radiation for tumor growth inhibition, the FLASH effect. Compared to radiotherapy delivered at conventional dose rates, the FLASH effect appears only when irradiation is performed with dose rates over 40 Gy/sec. Radiotherapy under FLASH conditions may become a new strategy to treat cancer as it can potentially widen the therapeutic window of radiotherapy. The effect has been demonstrated with electrons, photons, and protons. Our group recently published in vitro and in vivo FLASH radiation results with carbon ions. We demonstrate for the first time that the FLASH effect is possible even with ions heavier than protons, opening new perspectives in cancer treatment with heavy particles. We will present the results of a C3H/He osteosarcoma mouse model exposed to the entrance channel or the SOBP of an ultra-high dose rate of carbon ions. Compared to conventional exposure to carbon ions, the ultra-high dose rate of carbon ions spared the healthy tissues while providing comparable tumor control. Furthermore, the results point to fewer lung metastases in mice whose tumors were exposed under FLASH conditions than those treated with a conventional dose rate.

### O 071 - Biologic impact of multi-minutes beam pauses on ultra-high dose rate (FLASH) proton radiation in a mouse model

#### Danielle Johnson Erickson^1^, Marco Schwarz^1^, Eric Ford^1^, Robert Emery^1^, Marissa Kranz^1^, Juergen Meyer^1^, Peter Goff^1^, Ramesh Rengan^1^, Jing Zeng^1^, Ning Cao^1^

##### ^1^University of Washington, Radiation Oncology, Seattle, USA

**Background**: We previously demonstrated a survival benefit in mice radiated with pelvic FLASH proton radiation compared to conventional (CONV) dose-rate radiation. Since clinical radiation treatments often involve multi-minute pauses in delivery (i.e. multiple fields), we sought to evaluate if these pauses would negate the FLASH effect.

**Methods**: A Scanditronix MC50 compact cyclotron beamline produces a 48.7MeV proton beam between 0.1-150 Gy/s. The system produces a 6cm diameter scattered-proton beam (flat to +/- 3%) at target. We radiated 6-week-old female C57BL/6 mice using the proton beam entrance region, whole pelvis (1.5cm-tall field) to 19-20 Gy at CONV (0.6–1 Gy/s) and FLASH (80-100 Gy/s) dose rates. Mice were stratified into 5 groups: 1) control/sham radiation; 2) CONV dose rate 19Gy; 3) FLASH 19Gyx1 (one continuous beam delivery); 4) FLASH 9.5Gy x2 (2-minute pause in between two beams each delivering 9.5 Gy); and 5) FLASH 6.7Gy x3 (two 2-minute pauses between three beams each delivering 6.7Gy). Mice were observed for survival. Log-rank Mantel-Cox was used to compare between groups. All experiments repeated in duplicate.

**Results**: After whole pelvis radiation, survival was 100% in the control/sham radiation group, 13% in the CONV radiation group, and 33-43% in the three FLASH radiation group (p=0.05 between CONV and 19 Gyx1 FLASH arm). There was no significant difference in survival between the three FLASH groups (p>0.6 for all comparisons).

**Conclusions**: Survival benefit from FLASH radiation was not impacted by multiple 2-minute pauses in dose delivery in a mouse pelvic radiation model.

### O 072 - Modeling ultra-high dose rate electron and proton FLASH effect with the physicochemical approach

#### Hai Siong Tan^1^, Kevin- Boon Keng Teo^1^, Lei Dong^1^, Andrew Friberg^1^, Costas Koumenis^1^, Eric Diffenderfer^1^, Jennifer Wei Zou^1^

##### ^1^University of Pennsylvania, Perelman School of Medicine- Department of Radiation Oncology, Philadelphia, USA

In this work, we examine a physicochemical model that keeps track of the kinetics of formation and decay of reactive oxygen species following radiation in the FLASH regime based on a recent work by Labarbe et al. We study the numerical solutions of the system of coupled differential equations equipped with the dose and beam parameters adopted in different FLASH experiments published in recent literature. Overall, we find many suggestive correlations between the area-under-the-curve of radical concentrations and the FLASH biological effects reported in these works. For a recent work that studied the full dose response curves for acute skin damage in mouse legs, we use the model to determine isoeffect curves in the dose/dose-rate parameter space, from which dose modifying factors can be inferred. Our work demonstrates that radical reaction kinetics can potentially enact a crucial role in the FLASH effect.

### O 073 - A chemical track structure analysis of the experimental observation of the FLASH effect at high LET

#### Emanuele Scifoni^1^, Daria Boscolo^2^, Gianmarco Camazzola^2^, Michael Krämer^2^, Uli Weber^2^, Marco Durante^2^, Martina Fuss^3^

##### ^1^INFN, Trento Institute for Fundamental Physics and Applications TIFPA, Trento, Italy ^2^GSI, Biophysics, Darmstadt, Germany ^3^Med_AUSTRON, Medical Physics, Wiener Neustadt, Austria

While the entire mechanism of the differential FLASH effect is far from being understood, despite the steadily accumulation of preclinical data, the radiation quality dependence of the latter effect is yet a more puzzling issue. In fact, even though most of the initial estimates were predicting for high LET radiation a strong reduction of the conditions allowing FLASH sparing[1], recent observations reported indications of a possible effect in vitro for helium[2] and carbon ions[3] and even in vivo[4]. By means of TRAXCHEM chemical track structure code, we analysed the radiation chemical impact of the temporal structures of plans irradiated in the specific experiments where a positive effect was found. Proton, helium and carbon ion plans, in spread out Bragg peak irradiation configuration (realized through range modulators) were considered, at different energies and oxygenation levels, attempting to reproduce the impact on oxygen depletion and radical-radical recombination, correlating different “effective” voxel-based dose rate metrics definitions to relevant time scales for the chemical processes. While proton plans returned compatible conditions for radical-radical recombination in the extreme cases of low LET and more generous dose rate definition, helium and carbon plans return much larger intertrack distances which especially in the case of carbon are excluding any possible recombination[2] of the species generated around the tracks in the relevant time scales for chemical interaction. Ruling out of mechanisms related to intertrack recombinations (as well as to oxygen depletion) seems necessary if the pilot high LET FLASH experimental results will be confirmed.

### O 074 - Assessing the biomolecular mechanisms underlying proton FLASH-RT through infrared microspectroscopy

#### Immaculada Martínez-Rovira^1^, Roberto González-Vegas^1^, Marjorie Juchaux^2^, Annaïg Bertho^2^, Iturri Lorea^2^, Ludovic De Marzi^3^, Annalisa Patriarca^3^, Ibraheem Yousef^4^, Yolanda Prezado^2^

##### ^1^Universitat Autònoma de Barcelona UAB, Physics Department, Cerdanyola del Vallès, Spain ^2^Institut Curie, Translational Research, Orsay, France ^3^Institut Curie, Protontherapy Center, Orsay, France ^4^ALBA Synchrotron, MIRAS Beamline, Cerdanyola del Vallès, Spain

FLASH radiotherapy (FLASH-RT) is an emerging modality that uses high dose rates of radiation to enable curative doses to the tumour while preserving healthy tissue. The impressive biological studies showed the potential of FLASH-RT to revolutionize cancer treatments. However, the complex biological basis underlying FLASH-RT remains unknown. Within this context, the aim of this work was to assess the biomolecular mechanisms underlying proton FLASH-RT through FTIRM (Fourier Transform Infrared Microspectroscopy). For this purpose, male Fischer rat whole brains (excepting cerebellum and olfactory bulb) were irradiated with a dose of 15 Gy at the Orsay Proton therapy Center (France). Proton irradiations were performed using standard (4.00±0.02 Gy/s) versus FLASH (257±2 Gy/s) dose rates. Analysis was performed at 12 hours post-RT. FTIRM data was acquired at the MIRAS Beamline of ALBA Synchrotron. Raster scanning maps of the whole rat brain sections were collected for each irradiation condition with a spatial resolution of 100x100 μm2. Principal Component Analysis (PCA) was performed in different regions of the brain sections. Preliminary PCA scores showed a clear separation between conventional proton RT and proton FLASH-RT irradiations in the fingerprint region. PCA loading plots revealed that most of the variance accounting for the separation between groups was related to conformational changes in the proteins. Differences in the lipid spectral ratios related to lipid structure, chain length, oxidative stress and lipid peroxidation were also observed, providing new insights into the biomolecular effects involved in proton FLASH-RT through FTIRM.

### O 075 - A simple biological dose can explain proton FLASH outcome variations with changing doses, dose rates, repainting schemes and beam splitting

#### Per Poulsen^1^, Mateusz Sitarz^2^, Eleni Kanouta^2^, Jacob Johansen^2^, Line Kristensen^2^, Christina Ankjærgaard^3^, Claus Andersen^3^, Cai Grau^2^, Brita Sørensen^2^

##### ^1^Aarhus University Hospital, Department of Oncology and Danish Center for Particle Therapy, Aarhus N, Denmark ^2^Aarhus University Hospital, Danish Center for Particle Therapy, Aarhus, Denmark ^3^Technical University of Denmark, DTU Health Tech, Roskilde, Denmark

**Introduction**: In a range of independent proton FLASH studies, we determined the acute skin toxicity incidence in mice as function of dose, dose-rate, repainting scheme and number of identical deliveries that the treatment was split into. A model that explains the observed toxicity variations across all irradiations may facilitate understanding of the conditions needed for FLASH.

**Methods**: The right hind limb of 603 CDF1 mice were irradiated in the entrance plateau of a PBS proton beam. The incidence of acute skin toxicity (of level 1.5-2.0-2.5-3.0-3.5) was scored for 47 different irradiation groups that mapped toxicity as function of dose for conventional and FLASH dose rate, toxicity as function of dose-rate with and without repainting, and toxicity when splitting the treatment into 1-6 identical deliveries separated by 2 minutes. The ability of an oxygen enhancement ratio weighted biological dose (D_biol_) to explain the toxicity variations observed across all studies was investigated using a simple oxygen kinetics model [Petersson, IJROPB 2020, with O_env_=0.19%, g=0.19Gy^−1^, lambda=0.25s^−1^, K=0.3%, m=3].

**Results**: Figure 1 shows the model-predicted conversion from the variable tested in each study (x-axis) to D_biol_ (y-axis). After conversion to D_biol_, all studies agreed with similar MDD_biol_50 (D_biol_ giving 50% moist desquamation incidence) for all toxicity levels (Figure 2). D_biol_ could thus explain the observed toxicity variations across all studies.

**Conclusion**: D_biol_ quantitatively explained the varying FLASH effect observed for a wide range of conditions. Calculating D_biol_ for other combinations of dose, dose-rate, repainting scheme and beam splitting may further elucidate the conditions needed for FLASH in this mouse model.

### O 076 - Multi-institutional consensus report on machine QA for Ultra High Dose Rate (UHDR) proton beams (FLASH) in transmission mode

#### Kees Spruijt^1^, Sina Mossahebi^2^, Haibo Lin^3^, Eunsin Lee^4^, James Kraus^5^, Anees Dhabeen^6^, Per Poulsen^7^, Matthew Lowe^8^, Ahmet Ayan^9^, Jeremy Godart^10^, Mischa Hoogeman^10^

##### ^1^HollandPTC, HollandPTC, Delft, Netherlands ^2^University of Maryland School of Medicine, Department of Radiation Oncology, Baltimore, USA ^3^New York Proton Center, New York Proton Center, New York, USA ^4^University of Cincinnati Medical Center, Department of Radiation Oncology, Cincinnati- USA, USA ^5^Hazelrig-Salter Radiation Oncology Center, Hazelrig-Salter Radiation Oncology Center, Birmingham, USA ^6^Emory University of Medicine, Department of Radiation Oncology, Atlanta, USA ^7^Aarhus University Hospital, Department of Oncology, Aarhus, Denmark ^8^The Christie NHS Foundation Trust, Christie Medical Physics and Engineering, Manchester, United Kingdom ^9^Ohio State University Medical Center, Department of Radiation Oncology, Columbus, USA ^10^University Medical Center Rotterdam, Department of Radiotherapy, Rotterdam, Netherlands

**Introduction**: The first clinical trials to investigate UHDR deliveries for radiotherapy in human have started (FAST-01, FAST-02) and more trials to investigate the FLASH effect are foreseen. To increase comparability between trials it is paramount to ensure comprehensive dose delivery, which relies upon a strong machine quality assurance (QA) program. The AAPM TG-224 report is the current standard on machine QA, however, it was not intended for UHDR proton beams. The aim of this work was to define guidelines on machine QA for UHDR proton beams.

**Methods**: An international group of medical physicists from different sites across the US and Europe gathered on a monthly basis for a period of one year. The following tasks were performed: (i) perform a risk assessment on the current standard of machine QA to determine the missing information for UHDR proton beams; (ii) write a report on machine QA for UHDR proton beams including recommendations for commercially QA devices and limits.

**Results**: The risk assessment showed a clear need for an additional guidance and recommendations on temporal dosimetry, including dose rate (constancy), spillage and scanning speed. Based on the group discussion and consensus, a report was created (Table 1), giving guidance on QA procedures.

**Discussion**: Multi-institutional consensus was reached on machine QA recommendations for an UHDR proton beam in transmission mode. The discussions also showed that there is lack of commercial detectors with a temporal resolution of ≪1ms and a need for additional experimental data to set proper limits for dose rate (constancy) and scanning speed.

### O 077 - Multiple institution planning, delivery and dosimetry comparisons for proton pencil beam scanning: Development of standard PBS planning cases (TG350)

#### Charles D. Bloch^1^, Jatinder Saini^1^, Dominic Maes^1^, Samantha Hedrick^2^, Nicolas Depauw^3^, Aman Anand^4^, Li Zhao^5^, Chin-Cheng Chen^6^, Pingfang Tsai^6^, Tiezhi Zhang^7^, Charles Shang^8^, Yu Chen^9^, Rachel Ger^10^, Haibo Lin^6^, Kristen McConnell^11^, Peng Wang^12^, Xuanfeng Ding^13^, Brian Neal^14^, Xiaoying Liang^15^, Lingshu Yin^10^

##### ^1^University of Washington, Radiation Oncology, Seattle- WA, USA ^2^Thompson Proton Center, Medical Physics, Knoxville- TN, USA ^3^Massachusetts General Hospital, Radiation Oncology, Boston- MA, USA ^4^Mayo Clinic Arizona, Radiation Oncology, Phoenix- AX, USA ^5^St. Jude Children’s Research Hospital, Radiation Oncology, Memphis- TN, USA ^6^New York Proton Center, Radiation Oncology, New York- NY, USA ^7^Washington University School of Medicine, Radiation Oncology, St. Louis- MO, USA ^8^South Florida Proton Therapy Institute, Radiation Oncology, Delray Beach- FL, USA ^9^Georgetown University Hospital, Radiation Medicine, Washington DC, USA ^10^Johns Hopkins University, Radiation Oncology, Washington DC, USA ^11^Miami Cancer Institute, Radiation Oncology, Miami- FL, USA ^12^Inova Health System, Radiation Oncology, Annandale- VA, USA ^13^William Beaumont Hospital, Radiation Oncology, Royal Oak- MI, USA ^14^ProCure Treatment Center, Radiation Oncology, Hillsborough- NJ, USA ^15^Mayo Clinic, Radiation Ongology, Jacksonville- FL, USA

AAPM Task Group 350 (TG350) has created seven site-specific end-to-end tests for proton pencil beam scanning (PBS) systems. The seven sites are prostate, brain, breast, c-shaped target, craniospinal, head and neck, and prostate with pelvic nodes. Each test consists of a homogeneous phantom CT data set, a site-specific CT structure set, and a set of planning instructions and goals. Each test was validated by teams with access to various proton delivery systems (Hitachi, IBA, Mevion, Protom and Varian). The teams were comprised of two dozen physicists from 15 different institutions with clinical proton therapy systems. Each exercise was planned with current commercial treatment planning systems (Astroid, Eclipse, Raystation) including pencil-beam, Monte Carlo, and Boltzmann solver algorithms. All plans were delivered on each different treatment machine and dose distributions were measured in the variety of ways currently used in patient specific QA. This included point dose ion chamber measurements, composite dose film measurements and pre-field ion chamber array measurements. The measurements were compared to the dose calculations and the results for each combination of machine/TPS/measurement technique were compiled. These tests and the measurement results from the task group participants will be published and provided to the community as a reference for their own commissioning and quality assurance programs. An overview of the tests will be given.

### O 078 - Experimental microdosimetry as LET and RBE monitor for Quality Assurance in clinical proton beams

#### Anna Bianchi^1^, Anna Selva^1^, Valeria Conte^1^

##### ^1^Laboratori Nazionali di Legnaro - Istituto Nazionale di Fisica Nucleare, Fisica delle radiazioni, Legnaro, Italy

Proton therapy treatment planning systems (TPS) are based on a fixed Relative Biological Effectiveness (RBE) equal to 1.1 all along the depth-dose profile. It is recognized that RBE sharply increases in the last millimetres of the proton range reaching values 50-60% higher. New TPS optimized on radiation quality (RQ) are under investigation to improve the outcomes of treatments. These new TPS could be based on a variable RBE or Linear Energy Transfer (LET) that is a good physical estimator of the RQ. However, as the dose is routinely before treatments to confirm the TPS calculation, a new Quality Assurance (QA) procedure must be implemented for new TPS and both RBE and LET are difficult or impossible to measure on a daily basis. Microdosimetry is a valuable tool that performs the spectral characterization of the radiation field that is related to the RQ and can be correlated to LET by means of the average values of the microdosimetric distributions or to RBE through models. Miniaturized Tissue Equivalent Proportional Counters (mini-TEPCs) have been developed to cope with the high intensity beams used in proton therapy and to perform a characterization of the RQ with high spatial resolution. In this talk, data measured with mini-TEPCs in different clinical proton beams, 62 MeV Spread Out Bragg Peak (SOBP) and 148 MeV SOBP, will be compared to LET showing a good agreement (Figure 1 for the 62MeV SOBP, Figure 2 for the 148MeV), and with RBE when different models are applied to microdosimetric spectra.

### O 079 - Quick and accurate patient-specific quality assurance of carbon-ion radiation therapy using log file

#### Yongdo Yun^1^, Min Cheol Han^1^, Hikaru Souda^2^, Sung Hyun Lee^2^, Yuya Miyasaka^2^, Takeo Iwai^2^, Jin Sung Kim^1^

##### ^1^Yonsei University College of Medicine, Department of Radiation Oncology, Seoul, Korea Republic of ^2^Yamagata University, Department of Heavy Particle Medical Science, Yamagata, Japan

The objective of this study is to develop a patient-specific quality assurance (PSQA) system of carbon-ion radiation therapy using a log file. The system has comprised two modules, named quick check and accurate check; these modules can calculate delivered dose distributions using the treatment planning system (TPS) and TOPAS Monte Carlo (MC) code, respectively. The quick check module was coded by scripting programming included in commercial TPS. The log file can be implemented in the module, and the delivered dose could be quickly calculated by using TPS algorithm. The MC-based module could be used for more accurate dose calculation, such as inhomogeneity evaluation. The module can calculate relative biological effectiveness (RBE) dose as an updated version from the previous presented by PTCOG60. For the validation test of our system, a phantom-based treatment plan and its delivered log file were obtained from Yamagata University Hospital, and calculated the delivered dose by each module. Figures 1 and 2 show screenshots of the PSQA system implemented in TPS and TOPAS, respectively. It was confirmed that delivered dose distributions were successfully calculated by reflecting the log data. The developed system will be routinely used in our clinical practice. The demonstration of the developed system and its detailed results would be presented at PTCOG61.

### O 080 - Fred Monte Carlo for routine clinical application at Cyclotrone Center Bronowice IFJ PAN in Cracow

#### Dawid Krzempek^1^, Wiktor Komenda^1^, Marta Balamut^1^, Jan Gajewski^1^, Magdalena Garbacz^1^, Hubert Jablonski^1^, Marzena Rydygier^1^, Angelo Schiavi^2^, Renata Kopec^1^, Antoni Rucinski^1^

##### ^1^Institute of Nuclear Physics PAN, Cyclotrone Centre Bronowice, Cracow, Poland ^2^Sapienza University of Rome, Department of Basic and Applied Sciences for Engineering, Rome, Italy

Increasing number of proton therapy patients and limited patient QA beamtime enforce the need of alternative dose recalculation and verification methods, such as Monte Carlo (MC) simulations. In this work the status of clinical implementation of GPU-accelerated MC code FRED [1] at the Cyclotron Center Bronowice (CCB) IFJ PAN will be presented. Commissioning and validation of the FRED proton beam model prepared for two gantries (IBA) at CCB [2] is currently followed by development of a semi-automated workflow that includes the data export from TPS (Varian Eclipse 16.1), MC simulation in a patient CT and MC results import to the clinical TPS. Based on the comparison of 36 clinical treatment fields, it was shown that using GPU-Accelerated FRED MC (2xGPU NVIDIA Titan) may decrease the plan recalculation time by a factor of about 1000 compared to GATE MC (100xCPU). The simulation results will be used for validation of TPS calculations, especially for complex treatment plans. We will also present the results of validation of FRED MC as an alternative to patient QA measurements. This validation included the comparison of the two dimensional dose distributions acquired using MatriXX PT (IBA Dosimetry) with the measurement planes simulated in water with FRED. The decrease in beamtime needed for Patient QA by about 500 hours/year is expected when FRED MC will partially replace the measurements.

### O 081 - Prompt gamma imaging for range verification in carbon ion radiation therapy

#### Aicha Bourkadi Idrissi^1^, Andrea Missaglia^2^, Francesco Casamichiela^2^, Davide Mazzucconi^2^, Giacomo Borghi^1^, Marco Carminati^1^, Stefano Agosteo^2^, Carlo Fiorini^1^

##### ^1^Politecnico di Milano, Dipartimento di Elettronica- Informazione e Bioingegneria - DEIB, Milan, Italy ^2^Politecnico di Milano, Dipartimento di Energia - DiE, Milan, Italy

In the field of hadrontherapy, instruments aimed to verify the particle range in vivo with a precision of few millimeters are under study. First clinical applications to proton range verification based on Prompt Gamma Imaging (PGI) technique have been recently demonstrated. On the other hand, the use of PGI verification within Carbon Ion Radiation Therapy (CIRT) is a field still quite unexplored. In this work we explore the PG fall-off retrieval capability with a pixelated knife-edge slit camera with a beam of C-ions at clinical energies through FLUKA numerical simulations (a general purpose Monte Carlo (MC) code for particle transport). Specifically, we scored the response of a pixelated knife-edge slit camera to the secondary particles emitted in the 3-7 MeV energy range by a ICRP soft tissue phantom emulating the patient irradiated with a monoenergetic pencil beam of C-ions of 150MeV/u (generic setup in Picture 1). Our results suggest that range verification on a spot-by-spot basis is not feasible to achieve the 4 mm accuracy target, due to the small number of ions delivered in a single spot, in the order of 10^6. However, a layer-by-layer approach seems possible, where the average number of primary particles delivered in a spill to cover a given layer of the tumor volume is about 5 × 10^7 ions. The conclusions drawn in the present simulation work will be soon validated in an experimental campaign, whose results will be reported at the conference.

### O 082 - Implementation of a heart dose based adaptative protocol in proton therapy of esophageal cancer

#### Richard Canters^1^, Vicki Trier Taasti^1^, Kim Van der Klugt^1^, Vilches Freixas Gloria^1^, Maaike Berbee^1^

##### ^1^GROW School for Oncology and Developmental Biology- Maastricht University Medical Centre+, Department of Radiation Oncology MAASTRO, Maastricht, Netherlands

**Purpose**: In an evaluation of our first 60 esophageal cancer patients treated with intensity modulated proton therapy (IMPT), we observed a systematic increase in heart dose during the treatment. In this study, we revaluated the effectiveness of a mean heart dose (MHD) based adaptive protocol.

**Methods**: We included 83 esophageal cancer patients treated in our institute with preoperative or radical chemoradiotherapy. Patients were treated using 4, 3, or 2 beam IMPT (Figure 1) using a combination of 3 mm internal target volume (ITV) margin and 5 mm setup robustness. Each patient received weekly repeat CTs during treatment, on which re-delineation took place. 60 Patients were treated according to an adaptation protocol that only evaluated ITV coverage, while 23 patients were treated according to an adaptive protocol that required adaptation both if under-dosage of the ITV occurred, or if MHD increased >1.5Gy with respect to the planning.

**Results**: In the patient group without OAR monitoring, a clear trend in heart dose was observed during treatment course, with an average increase in MHD of 0.5 Gy/wk (confidence interval (CI): [0.3,0.7] Gy/wk), while the group with OAR monitoring experienced an average MHD increase of 0.1 (CI: [-0.1,0.3]) Gy/wk (p<0.05). The high dose volume within the heart (V40Gy) showed similar results: 1.1 (CI: [0.7,1.5]) p.p./wk vs 0.2 (CI: [0,0.4]) p.p./wk (p<0.05) (Figure 2).

**Conclusion**: An adaptation protocol with additional focus on MHD can prevent undesired increases in heart dose in esophagus IMPT treatment.

### O 083 - Adaptive planning in PBS proton Beam therapy: Experience in consecutive 300 patients

#### Srinivas Chilukuri^1^, Utpal Gaikwad^2^, Sham Sundar^1^, Sapna Nangia^1^, Noufal Mp^1^, Manikandan Arjunan^1^, Rajesh Thiyagarajan^1^, Kartikeswar Patro^1^, Dayananda Sharma^1^, Rakesh Jalali^1^

##### ^1^Apollo Proton Cancer Centre, Radiation Oncology, Chennai, India ^2^Krupamayi Hospitals, Radiation Oncology, Aurangabad, India

**Background**: Inherent physical properties of pencil beam scanning proton beam therapy (PBS-PBT) makes it highly prone to significant dose perturbations necessitating treatment adaptations.

**Methods**: Initial 300 consecutive patients treated with PBS-PBT with daily image-guidance at our centre were analysed retrospectively for parameters related to adaptive re-planning. Patients underwent re-planning(QA)-CT after every 5-10fractions or earlier based on clinical or CBCT trigger. Nominal plans were overlaid and recalculated on QA-CTs to assess dose perturbations and need for adaptive re-planning. Several changes in planning strategies (Figure-1) were implemented after approximately initial 100 patients to reduce need for adaptive re-planning.

**Results**: Most patients underwent at least 3QA-CTs. 80 patients(26.67%) underwent adaptive re-planning of which 10(12.5%) underwent more than once. Most common sites, frequency, timing and reasons for plan-adaptation for various site are described in Chart-1. 52% of plans were adapted due to overdose in OARs, 31% were due to under-coverage of target while 17% had both and average dose perturbation was 9.2%(7-15%) and 6.5%(3-9%) in OAR and target doses respectively. Trigger for adaptive replanning was clinical in 10%, CBCT in 72%, and QACT in 18%. Most patients(97%) did not need treatment interruptions due to delays in re-planning. Of the initial 100 patients, 37% underwent plan adaptations while in next 200, 21.5% required adaptive re-planning.

**Conclusion**: There is considerably high requirement for adaptive re-planning during PBS-PBT. Despite routine use of CBCTs, QA-CTs may be required to identify need for adaptive re-planning especially in vulnerable sites/patients. Change in planning approaches may reduce need for adaptive re-planning.

### O 084 - Use of failure mode and effects analysis to efficiently implement daily adaptive proton therapy workflow in the clinic

#### Katarzyna Czerska^1^, Ilija Andaca^1^, Evangelia Choulilitsa^1,2^, Filippo Grillo Ruggieri^1^, Jan Hrbacek^1^, Antony John Lomax^1,2^, Arturs Meijers^1^, Michelle Van Heerden^1^, Damien Charles Weber^1,3,4^, Francesca Albertini^1^

##### ^1^Paul Scherrer Institute, Center for Proton Therapy, Villigen PSI, Switzerland ^2^ETH Zürich, Department of Physics, Zürich, Switzerland ^3^Inselspital- Bern University Hospital- University of Bern, Department of Radiation Oncology, Bern, Switzerland ^4^University Hospital of Zürich, Department of Radiation Oncology, Zürich, Switzerland

The necessity of daily adaptation in radiotherapy results from the deteriorating effect of anatomical variations on dose distribution during the course of treatment. The daily-adapted workflows are not only technologically challenging, but also require customized design of safety and QA procedures. Therefore, the ongoing development of daily adaptive proton therapy (DAPT) workflow at PSI takes into account the failure mode and effects analysis (FMEA) as the preparation step for future clinical integration. To identify the risk associated with individual steps of DAPT, the workflow was divided into three phases: pre-treatment, online and offline. The multidisciplinary team (oncologists, physicists, therapists) assigned the severity, probability and detectability scores (1 to 10) to the predefined failure modes of each phase, and subsequently, calculated the resulting risk priority numbers (RPNs) for all scenarios (Table 1). Between 5 to 25 DAPT-related failure modes were identified in different phases of the workflow. All of them were associated with human or software errors. Based on the resulting RPNs and current design controls, over twenty recommended actions were recognized and included: update/expansion of current clinical documentation, users training and/or workflow standardization, which are currently under the ongoing preparation. For newly introduced radiotherapy techniques such as DAPT, the FMEA is an indispensable step on the clinical implementation, leading to an increased patient safety and staff resources optimization. Moreover, as achieved in this study, it allows identifying the workflow drawbacks and design recommended actions prior to the clinical integration and thus minimizing the risk in advance.

### O 085 - Optimal QACT frequency during intensity modulated adaptive proton therapy

#### Nrusingh Biswal^1^, Mark Zakhary^1^, Ruslan Mogilnay^1^, Elizabeth Nichols^1^, Matthew Witek^1^, Byongyong Yi^1^

##### ^1^University of Maryland School of Medicine, Radiation Oncology, Baltimore, USA

**Purpose**: Patients treated with proton beam therapy (PBT) undergo routine quality assurance CT (QACT) scans during their course of treatment, which determines whether the initial plan remains accurate for the whole course of treatment or needs to be adaptively planned based on their QACT findings. Benefits of optimizing QACT frequency include reducing imaging dose and optimizing use of patient time and staff resources.

**Methods**: We performed a retrospective IRB-approved single-institution review of 2456 patients, who were treated with PBT between 2017 and 2021. There were 6954 QACTs performed, out of which only 500 (7.2%) were used for adaptive planning (table 1). For each anatomic site, QACT and adaptive planning patterns were analyzed to identify the optimal frequency for QACTs for different anatomical sites, while maintaining the same quality of care as before.

**Results**: The recommended QACT schedules for different anatomic sites are presented in the table 2. In addition, no QACTs are required if ≤ 5 fractions for all the anatomic sites, except SBRT patients need a QACT at first fraction. No QACTs within the last 7-10 fractions unless planning a break. QACT on first day of treatment for those patients where there has been a gap ≥ 3 weeks between the simulation and the first fraction delivery or any clinical change indications or change of implants/devices.

**Conclusion**: This newly proposed QACT patterns for all anatomic sites are optimal and maintain treatment quality. Following this analysis, we have adopted a new clinical paradigm for adaptive proton therapy with decreased routine QACTs.

### O 086 - PRAGUE (Proton RAnGe measure Using silicon carbidE): The first detector to measure proton-beam range with conventional and flash beams online

#### Giada Petringa^1^, Pablo Cirrone^1^, Mariacristina Guarrera^1^, Daniele Margarone^2^, Alma Kurmanova^1^, Salvatore Tudisco^1^

##### ^1^Istituto Nazionale di Fisica Nucleare INFN, Laboratori Nazionali del Sud LNS, Catania, Italy ^2^ELI-Beamlines, Institute of Physics FZU- Czech Academy of Sciences, Dolni Brezany, Czech Republic

Measuring and verifying the reliability and stability of the dosimetric properties of a radiotherapeutic beam is the most important task of any external-beam radiotherapy quality assurance program. We will present the work done within the PRAGUE (Proton Range Measurement Using Silicon Carbide) project funded by the H2020 and Fyzikální ústav AV ČR, v.v.i in the framework of the MSCA-IF IV program and by the INFN in the framework of the program for young researcher grants. The main goal of PRAGUE was the design, simulation, realization, and characterization of a real-time depth-dose distribution detector system based on thin Silicon Carbide multilayers for conventional and flash proton beams in the energy range between 30 MeV to 150 MeV. The detector developed was designed to work at the regime of extremely high dose rate beams and it allows the retrieval of real-time depth dose distributions with a high spatial resolution thanks to the development and use of a 10 μm, fully depleted 15x15 mm^2^ square SiC detector. A detector prototype was already realized, simulated, and tested with 30 and 70MeV conventional proton beams. The obtained results indicate the SiC detector is a suitable detector for relative dosimetry with charged particles. It showed, in fact, a stable and reproducible response and extremely good behaviour in terms of linearity with respect to absorbed dose was found. The negligible dependence of its response against energy and dose-rate and the high radiation hardness, represent advantageous features with respect to other commercial solid-state detectors for ion beam dosimetry.

### O 087 - Mitigation of range uncertainty for proton beam therapy using a millimeter-scale diode-based in vivo dosimeter

#### Kyoungtae Lee^1^, Rahul Lall^2^, Jessica Scholey^1^, Kavita Mishra^1^, Michel Maharbiz^2^, Mekhail Anwar^1^

##### ^1^University of California- San Francisco, Radiation Oncology, San Francisco, USA ^2^University of California- Berkeley, Electrical Engineering and Computer Science, Berkeley, USA

**Objective**: Proton beam range uncertainty, day-to-day anatomical variations, and target motion can potentially lead to both inaccurate targeting and increased toxicity via inadvertent dose to neighboring OARs. Key to mitigating these challenges is real-time knowledge of dose deposition within the tumor and OARs. Given both the highly non-linear dose deposition (LET) and mapping to biological effect of heavily-charged particles, real-time measurements of single-particle LET are needed.

**Methods**: Here, we present a novel 0.96x0.94x0.3 mm3 single-particle sensitive, diode-based in vivo dosimeter silicon chip, capable of direct implantation within tumors and near OARs. 4,096 nearly minimum size (1 µm2) diodes, signal amplifiers, and memories are all integrated into a silicon chip. The sensor directly measures protons incident on the sensor, providing relative measurements of particle LET and count with single-particle sensitivity in real-time. Only minimal electrical power (∼0.5 µW) and digital communication are needed to operate the sensor, suitable for future wireless implementation. Dose data streamed from sensors can be used to adjust the beam settings (i.e. proton energy), enabling real-time quality assurance for accurate tumor and OAR dose deposition.

**Results and Conclusion**: The sensor is successfully verified under a clinical 67.5 MeV proton beam in a water tank setup, and the measurement data is compared with Monte-Carlo simulation (TOPAS). The proposed novel system is the first proof of principle that can be utilized to deliver real-time, spatially localized tumor and OAR dose data, to ultimately improve the treatment outcomes by addressing dose and range uncertainties in proton EBRT.

### O 088 - Design of a new prompt gamma detection system associated to proton pulsed beam

#### Pierre Everaere^1^, Denis Dauvergne^1^, Etienne Testa^2^, Létang Jean-Michel^3^, Marie-Laure Gallin-Martel^1^

##### ^1^Laboratoire de Physique Subatomique et de Cosmologie, Physique Nucléaire et Applications Médicales, Grenoble, France ^2^IP2I, Prisme, Lyon, France ^3^INSA-Lyon, Creatis, Lyon, France

The on-line monitoring of the ion path in the patient in hadrontherapy represents a challenge whose resolution will make it possible to reduce the margins around the Clinical Target Volume (CTV) and the number of irradiation fields required. This monitoring can be performed by means of the detection of secondary particles such as the prompt gamma rays (PG) generated during nuclear reactions undergone by a fraction of the incident ions [Krimmer NIM 2018: https://doi.org/10.1016/j.nima.2017.07.063]. The objective of this work is to study and compare two detection systems and determine their operating regimes at low and high pulse beam intensities of the synchro-cyclotron S2C2 developed by IBA. These detection systems consist of scintillator detectors located around the patient and they have their own acquisition modalities. The new detection system envisaged to accommodate the highest beam intensities during processing is based on the measurement of the photon energy deposited during a processing spot of a few microseconds (Prompt Gamma Energy Integral). The comparison is made with an event-by-event acquisition system using the Prompt Gamma Peak Integral technique [Krimmer APL 2017: https://doi.org/10.1063/1.4980103] which is based on photon-by-photon detection on each of the detection channels. The feasibility study of these PGEI and PGPI techniques performed with Monte Carlo simulations (Gate version 9) will be discussed and compared to experimental results obtained with various types of gamma detectors.

### O 089 - Stopping-Power-Ratio (SPR) acquisition using counting CT information for charge particle therapies

#### Bo Lu^1^, Park Chunjoo^1^, Sridhar Yaddanapudi^1^, Xiaoying Liang^1^, Chris Beltran^1^, Keith Furutani^1^

##### ^1^Mayo Clinic, Radiation Oncology, Jacksonville, USA

**Purpose**: Most of current treatment planning systems use single-energy CT (SECT) Hounsfield Units (HU) to estimate charge particle SPRs for dose computation. Several recent studies suggested that Dual Energy CT (DECT) can be employed to improve the accuracy of SPRs by extracting both electron density and effective Z number from scans. With Counting-CT (CCT) technology emerging, an opportunity to further improving the accuracy of SPR calculation was presented. In this work, we explore an approach of using CCT scan information to acquire SPR values.

**Methods and Materials**: Both CCT and DECT scans were acquired on a CIRS electron Density phantom using SOMATOM and NAEOTOM CT scanners (Siemens Healthieer Co.), respectively. Electron density and effective Z number were extracted from DECT whereas decomposed material component was extracted from CCT scan using Siemens image processing software. Effective Z number was subsequently calculated using material component data extracted from CCT data. The average SPR values of each material for both proton and carbon ions were then calculated using Hunemohr’s method for both scans. Both CCT results and DECT results were compared to the gold standard calculated using CIRS provided data.

**Results and Conclusions**: The comparison results suggested that SPR values calculated using CCT scan are closely in align with gold standards. The root mean square errors of SPRs from CCT scan are even smaller than its counterpart from DECT scan. We therefore conclude CCT scan can potentially be utilized to enhance the accuracy of SPR value even further compared to DECT scans.

### O 090 - Ex-vivo proton range uncertainty verification of dose calculation on CT and CBCT-based virtual CT

#### Juan Perez^1^, Isabel Lorenzo Villanueva^1^, Rasmus Nilsson^2^, Sebastian Andersson^2^, Juan Antonio Vera Sánchez^1^, Juan Castro Novais^1^, Fernando Cerron Campoo^1^, Martin Janson^2^, Erik Engwall^2^, Alejandro Mazal^1^

##### ^1^Centro de Protonterapia Quirónsalud, Radiation Oncology, Pozuelo de Alarcón - Madrid, Spain ^2^RaySearch Laboratories, Research and Development, Stockholm, Sweden

**Objective**: To estimate calculated range uncertainty in CT images and synthetic CT from CBCT of an in-house phantom.

**Method**: A single-beam PBS plan has been optimized in Raystation 11B with the aim to achieve a uniform dose distribution with constant range in water behind an in-house ex-vivo phantom. The resulting PBS plan was recalculated on two synthetic CTs (sCT) based on a CBCT acquired in a Proteus One. The sCTs were generated using the “Virtual CT” (VCT) and “Corrected CBCT” (CorrCBCT) algorithms of RayStation 12B. Using a 2D array of parallel plane ion chambers (MatriXX One, IBA Dosimetry) in a DigiPhant PT water phantom a series of planes at different depths were measured and 3D dose distribution were reconstructed by stacking planes. Depth dose profiles were extracted from TPS calculation and measurement matching coordinates of every MatriXX detector.

**Results**: Two hundred twenty-three (223) dose lines have been evaluated. Figure 1a shows the average over all detectors of measured depth dose line versus average lines calculated in TPS for plan or real CT, and 2b shows a heat map of error ranges in VCT calculation showing that errors larger than 2mm coincide with bone areas and air-tissue interfaces.

**Conclusions**: Actual delivered doses can be evaluated based on CBCTs with uncertainties comparable to robust planning range uncertainty.

### O 091 - SISS: A novel method for fast, fully-automated robust multi-criterial IMPT planning

#### Wens Kong^1^, Michelle Oud^1^, Steven Habraken^1,2^, Merle Huiskes^3^, Eleftheria Astreinidou^3^, Coen Rasch^2,3^, Ben Heijmen^1^, Sebastiaan Breedveld^1^

##### ^1^Erasmus MC Cancer Institute- Universtiy Medical Center Rotterdam, Department of Radiotherapy, Rotterdam, Netherlands ^2^HollandPTC, Department of medical physics and informatics, Delft, Netherlands ^3^Leiden University Medical Centre, Department of Radiation Oncology, Leiden, Netherlands

**Purpose**: A novel method for fast, automated robust MCO (multi-criterial optimisation) in IMPT planning is proposed using Sparsity Induced Spot Selection (SISS). For cervical-, prostate- and head and neck cancer it is compared to conventional automated robust MCO with iterative pencil-beam resampling (IPBR, van de Water 2013).

**Methods**: Both MCO methods use the same ‘wish-list’ to configure aims and trade-offs for high-quality automated planning. The proposed method has three phases: 1) 20.000 spot candidates are distributed uniformly in the targets and the L-BFGS-B solver is used to optimise spot weights using a weighted-sum cost function derived from the wish-list, 2) 20% spots with the highest MU are selected for wish-list based MCO, 3) spots falling below the minimum MU requirement are removed, followed by a re-optimisation with the reference point method (RPM, Van Haveren (2017)) to ensure Pareto-optimality and adherence to constraints.

**Results**: Both methods were compared for computation times (Figure 1). On average, SISS reduced the optimisation time by a factor of 6, i.e. from 287 min to 47 min, with a maximum reduction by a factor of 13 for cervical cancer (210 min. vs. 13 min). The number of applied spots and energy layers were similar for both methods. For all sites, SISS produced similar CTV coverage and slightly reduced OAR dose, see Figure 2 for head and neck cancer.

**Conclusion**: The novel SISS for fully-automated robust MCO was 6 times faster than conventional IPBR (47 vs. 287 min) with similar target coverage and reduced OAR dose.

### O 092 - A deep learning approach for dose prediction of head and neck proton radiotherapy treatments

#### Ivan Vazquez^1^, Mary Gronberg^1^, Skylar Gay^1^, Steven Frank^2^, Ronald Zhu^1^, Laurence Court^1^, Ming Yang^1^

##### ^1^The University of Texas MD Anderson Cancer Center, Radiation Physics, Houston, USA ^2^The University of Texas MD Anderson Cancer Center, Radiation Oncology, Houston, USA

**Objective**: We investigated the effectiveness of a deep learning (DL) technique for predicting an optimal proton dose distribution for head and neck (H&N) cancer patients.

**Approach**: We used a dense and dilated 3D U-Net model that previously demonstrated an exceptional performance for photon dose prediction. A cohort of 152 bilateral H&N cases were used to train and test the DL model, with 90 cases used for training and 30 for validation. We used the remaining 32 cases to assess the performance of the model. All cases involved a three-beam configuration and had up to five prescription levels. As inputs, the DL model received 23 channels including the organs at risk, clinical target volumes (CTVs), and the planning CT. The performance of the model was evaluated using the mean absolute error (MAE), homogeneity index (HI), the Van’t Riet conformation number (CN), and comparisons of relevant dosimetric quantities.

**Result**: The MAE for predictions using the test group was 2.8±0.42 Gy while the HI and CN yielded mean absolute differences between the predictions and the ground truth of -0.01±0.02 and 0.02±0.47, respectively. The mean difference for the D_1%_, D_95%_, and D_99%_ of the clinical target volumes were -0.26±1.29 Gy, 0.20±0.88 Gy, and 0.38±1.53 Gy, respectively.

**Conclusion**: We have successfully trained a deep learning model to predict an optimal proton dose distribution. The model achieved a performance on par with past photon dose prediction results.

### O 093 - Problem size reduction in particle therapy treatment planning through AI-based selection of optimization voxels

#### Anastasiia Quarz^1,2^, Lennart Volz^1^, Moritz Wolf^1^, Marco Durante^1,3^, Christian Graeff^1,2^

##### ^1^GSI Helmholtzzentrum für Schwerionenforschung, Biophysics Department, Darmstadt, Germany ^2^Technische Universität Darmstadt, Department of Electrical Engineering and Information Technology, Darmstadt, Germany ^3^Technische Universität Darmstadt, Department of Condensed Matter Physics, Darmstadt, Germany

Online adaptive particle therapy requires fast, high-accuracy plan optimization. This is challenging, especially when robust biological or 4D dose optimization is needed. To reduce computational load, we developed a deep-learning method that removes voxels with predicted low relevance from the optimization. Due to the defined beam geometry, ignoring a fraction of voxels randomly selected from the interior of the volumes of interest (VOIs) in the computation of the dose influence matrix, while maintaining a high voxel density on the VOI boundary, can still yield high plan quality. This random sampling selection (RSS) is available in the TRiP98 research TPS and can reduce computational demand proportionally to the removed fraction of voxels but needs patient-specific settings for best results. To generalize the method, a deep learning framework was developed and trained on a cohort of head and neck cancer patients (50 train; 6 test), using the target coverage (D95>95%) as the main loss criterion. The RSS with previously established parameters for the patient cohort reduced the amount of target voxels to 25.6% (median), at median D95=98.6%. The AI framework, without further input, achieved a median D95=97.5% while reducing the number of voxels to 11.1% (median), providing a ∼85% decrease in memory compared to optimizing on all voxels. The AI voxel selection framework greatly improves computational efficiency, without the need for patient-specific parameter tuning, while maintaining high plan quality. The method hence has great potential for increasing offline workflow efficiency and enabling online plan re-optimization.

### O 094 - Optimizing on spots positions for better FLASH intensity modulated proton therapy

#### Pierre Lansonneur^1^, Anthony Magliari^2^, Lesley Rosa^2^, Jessica Perez^3^, Michael Folkerts^2^

##### ^1^Varian Medical Systems, FLASH Program, Le Plessis-Robinson, France ^2^Varian Medical Systems, FLASH Program, Palo Alto, USA ^3^Varian Medical Systems, FLASH program, Geneva, Switzerland

**Background**: In Intensity Modulated Proton Therapy (IMPT), the weights of individual pencil-beams or spots are optimized to fulfill dosimetric constraints. Theses spots are usually located on a regular lattice and their positions are fixed during optimization. To achieve ultra-high dose rate (FLASH-RT), the range of spot weights may however be limited to certain values, leading sometimes to sub-optimal plan quality.

**Aims**: To improve further the quality of FLASH plans, we propose a Direct Machine Parameter Optimization (DMPO) algorithm to optimize simultaneously the spots weights and positions.

**Methods**: Single-energy-layer transmission IMPT plans were created for peripheral lung cases with varying PTV sizes. Guided by SBRT RTOG protocol [1], each plan was prescribed to deliver 15 Gy in three fractions to the PTV. Optimization of both MU and spot positions was performed while enforcing a minimum spot MU to ensure high dose-rate. Several dose metrics were characterized and compared to those reached for spot weight optimization only (conventional). The PBS dose rate [2] was then calculated and evaluated. Finally, the performance of DMPO and conventional optimization were compared.

**Results**: Unlike conventional optimization scheme, all plans optimized with the DMPO algorithm passed the RTOG protocol inspired metrics. The fraction of irradiated volume (Dose > 2 Gy) receiving at least 40 Gy/s was above 91% for lungs and 95% for esophagus. Overall, DMPO resulted in a significant plan quality improvement for all patients with similar execution time.

**Conclusion**: This new optimization method will support the creation of fractionated IMPT plans for FLASH-RT.

### O 095 - Beam-mask facilitated deep learning-based accurate and efficient dose prediction for pencil beam scanning proton therapy to treat prostate cancer

#### Lian Zhang^1^, Jason M. Holmes^1^, Carlos E. Vargas^1^, Nathan Y. Yu^1^, Sameer R. Keole^1^, Steven E. Schild^1^, Sujay A. Vora^1^, William W. Wong^1^, Jiajian Shen^1^, Wei Liu^1^

##### ^1^Mayo Clinic, Radiation Oncology, Phoenix, USA

**Purpose**: Prostate cancer patients comprise nearly 40% of all the patients treated at Mayo Clinic Proton Center in Arizona. Deep learning (DL) has shown promising dose prediction results in photon therapy. However, more DL improvement is still needed to handle more challenging dose prediction in pencil beam scanning proton therapy (PBSPT).

**Methods**: PBSPT plans of 103 previously treated prostate cancer patients (97 for training and 6 independent patients for testing) were included in the study, each with CT, structure set and plan dose calculated by the in-house developed Monte-Carlo dose engine. To improve the accuracy of the DL model for proton dose prediction, we used a novel beam mask generated by ray-tracing of proton beams. Fully connected 3D-Unet was adopted as the DL backbone. Dose volume histogram indices and 3D Gamma passing rates with a threshold of 3%/3mm/10% were used as evaluation metrices.

**Results**: The proposed model achieved dosimetric performance comparable to the manually-derived clinical plans with the differences of clinical-target-volume D95 <0.5Gy and most organs-at-risk (OARs) Dmean <0.4 Gy. The 3D Gamma passing rates between the model-predicted dose and the clinical plan dose within targets, OARs and BODY are 99.99%±0.02%, 95.23%±1.61% and 95.11%±1.52%. The average dose prediction time is 191± 25ms.

**Conclusions**: An accurate and efficient DL-based proton dose prediction framework has been developed for PBSPT, which can predict accurate dose distributions not only inside but also outside targets and OARs. The framework can potentially further reduce the planning and adaptive replanning workload for prostate cancer patients treated with PBSPT.

### O 096 - Proton LET re-optimization workflow in the clinical setting at MGH

#### Nicolas Depauw^1^, Thomas Madden^1^, Benjamin Clasie^1^, Hanne Kooy^1^

##### ^1^Massachusetts General Hospital, Radiation Oncology, Boston, USA

Bio-dose^1^ re-optimization is a required step of protocol 2018-0344 “Pilot trial of LET optimized IMPT for pediatric patients with ependymoma”. The intent of the protocol is to evaluate the safety of reducing high LET values in the brainstem and the potential outcome on end-of-range effects. This work describes the integration of this LET re-optimization process into the clinical workflow at MGH. A plan is generated in the Astroid treatment planning system following both the protocol guidelines and the MGH planning approach. The CTV is prescribed 54 GyRBE using two posterior-oblique fields and sometimes a posterior field. Aperture edges are used for maximum organs-at-risk sparing. A single-field optimization method is employed along with a max dose of 55 GyRBE to the patient to maximize treatment robustness. While being stricter than protocol requirements, this represents the MGH clinical practice. The plan is then reoptimized using Astroid’s optimizer NYMPH^2^ in PUMA (scripting environment). The computational steps are: L_ij_ (LET contributions) matrices generated for the approved plan; bio-dose generated for the approved plan based on the *Unkelbach*^1^ formalism: *PhysicalDose*×(1+*LET*×*c*) [*c*=0.03]; plan reoptimized such that the original set of constraints and objectives are met while reducing the five hottest percent in the brainstem bio-dose; plan fluences overridden/locked based on the re-optimization (seamless integration to the delivery system); and bio-dose generated for the reoptimized plan. The physician is then presented with both plans (Fig.1) and the preferred plan used for patient treatment. This process was experimentally validated.

### O 097 - Retrospective evaluation of LET distribution in carbon-ion radiotherapy for pelvic sarcomas and LET optimization by blocking method: The MedAustron approach

#### Ankita Nachankar^1,2^, Mansure Schafasand^3,4^, Eugen Hug^5^, Antonio Carlino^3^, Marcus Stock^3,6^, Joanna Góra^3^, Piero Fossati^5,7^

##### ^1^MedAustron Ion Therapy Center- Austria, Department of Radiation Oncology, Wiener Neustadt, Austria ^2^ACMIT, Medical Research & Development, Wiener Neustadt, Austria ^3^MedAustron Ion Therapy Center- Austria, Medical Physics, Wiener Neustadt, Austria ^4^Medical University of Vienna- Department of Radiation Oncology- Vienna- Austria, Medical Physics, Wien, Austria ^5^MedAustron Ion Therapy Center- Austria, Radiation Oncology, Wiener Neustadt, Austria ^6^Karl Landsteiner University of Health Sciences- Austria, Medical Physics, Krems an der Donau, Austria ^7^Karl Landsteiner University of Health Sciences- Austria, Radiation oncology, Krems an der Donau, Austria

**Purpose/Objective**: Carbon-ion radiotherapy (CIRT) is a potentially curative treatment for unresectable-pelvic sarcomas. Despite high RBE and LET, tumor control in large-sarcomas(>500cc) remains unsatisfactory– based potentially on lower intra-tumoral dose-averaged LET(LETd). The high-LETd region lies at the end of the beam-range. With increasing tumor size, the extent of tumor covered by high-LETd decreases. Hence, we evaluated LETd-optimization by using “blocking technique” in large sarcomas.

**Material and Methods**: CIRT-LETd distribution was evaluated in 20 patients treated for pelvic sarcomas (large>/=500cc, small<500cc, 10 each) to prescription of 73.6(64-76.8)Gy RBE/16fractions(4fractions/week) using the local effect model-I(LEM-I). Subsequently doses were recomputed using the modified-microdosimetric kinetic model(mMKM). LETd-optimization was applied for large-sarcomas only as small-sarcomas originally showed high-LETd. For LETd-optimization, blocking structures were created for bilateral and vertical beams, to stop beams 2cm beyond the midplane of GTV(Figures 1d-f), plans were reoptimized and LEM-I/mMKM doses and LETd were recomputed.

**Results**: Both blocked and non-blocked plans were dosimetrically comparable in terms of D50%, D98% for LEM-I/mMKM for the target, and OARs constraints with slight increase in D2%, for mMKM inside the target (Figure 1). Blocking in large tumors improved LETd to 2%, 50%, 98%, for high-dose-PTV by 12%, 21% 7%, for high-dose-CTV by 30%, 27%, 12% and for GTV by 37%, 31%, 15% respectively(Figure 2a-c). Favorable LETd profile was observed on OARs (rectum, bowel-loops, sacral-nerves) and 1cm-rim-outside-PTV. Additionally, blocking redistributed high-LETd region to the central region of GTV, presumably the hypoxic tumor region(Figure 2g-i).

**Conclusion**: LETd-optimization by “blocking method” is technically feasible, clinically-relevant. A prospective clinical study will commence soon.

### O 098 - Dosimetric and dose-averaged linear energy transfer(LETd) evaluation of Radiation Induced Contrast Enhancement (RICE) following spot scanning proton beam irradiation–initial report

#### Joanna Gora^1^, Mansure Schafasand^1,2^, Ankita Nachankar^1,3^, Piero Fossati^1,4^, Antonio Carlino^1^, Marta Mumot^1^, Markus Stock^1,4^, Eugen Hug^1^, Maciej Pelak^1^, Carola Lütgendorf-Caucig^1^

##### ^1^EBG MedAustron, Medical Department, Wiener Neustadt, Austria ^2^Medical University Of Vienna, Radiotherapy Department, Vienna, Austria ^3^ACMIT, Acmit, Wiener Neustadt, Austria ^4^Karl Landsteiner University of Health Sciences, Particle Therapy, Krems an der Donau, Austria

Four hundred twenty-one (421) patients have been treated between 2017-2021, with the median prescribed dose of 58.5GyRBE(range 40-78GyRBE) and the median D1% to brain of 54.3GyRBE(range 30-76GyRBE). 15% of the treated patients developed RICE following proton therapy. The purpose of this study was to investigate retrospectively the dose and LETd distributions in context of present RICE. The initial analysis included 10 clinical plans, 5 with detected RICE and 5 RICE free. Both groups had similar total dose(54-60GyRBE) and beam configuration (3-4 beams,spaced>30°). The RBE-weighted dose was computed with the constant 1.1 RBE, optimized with single field optimization technique(SFO). Detailed dose and LETd analysis were performed in the treated volume and selected brain subvolumes. Evaluated dose distributions were homogeneous throughout treated volume (per plan and per beam) That was in correspondence with the LETd distribution: target LETd was on average 2.5keV/µm(figure2), increased values were present only at the beam’s distal edge, outside of the treated volume. 2% of the Brain80%-PTV1 subvolume would increase to 7.5keV/µm, however this structure was far from the RICE region. RICE was located always in the beam’s entrance region, with average LETd=2keV/µm(similar to treated volume), that was considerably lower than LETd in the healthy brain subvolumes(fig1,2). Interestingly, RICE free patients followed similar dose and LETd values trends. Initial evaluation showed that not only LETd values do not increase in RICE region, but also overall LETd distributions for patients with and without RICE are comparable. Those results would suggest, that reported RICE was independent of the treatment technique and LET distribution.

### O 099 - Avoiding brain injuries after proton therapy: Variable proton RBE-based treatment planning to be tested in a prospective randomized trial

#### Habiba Sallem^1,2,3^, Semi Harrabi^1,4^, Erik Traneus^5^, Emanuel Bahn^1,2,3,4^, Klaus Herfarth^1,2^, Jürgen Debus^1,6^, Markus Alber^1,2,3^, Julia Bauer^1,2,3^

##### ^1^Heidelberg University Hospital, Department of Radiation Oncology, Heidelberg, Germany ^2^National Center for Tumor Diseases NCT, Department of Radiation Oncology, Heidelberg, Germany ^3^Heidelberg Institute for Radiation Oncology HIRO, Department of Radiation Oncology, Heidelberg, Germany ^4^German Cancer Research Center DKFZ, Clinical Cooperation Unit Radiation Oncology, Heidelberg, Germany ^5^RaySearch Laboratories AB, RaySearch Laboratories AB, Stockholm, Sweden ^6^German Cancer Consortium DKTK, Partner site Heidelberg, Heidelberg, Germany

Recently reported risk models for late-occurring MRI-contrast enhancing brain lesions (CEBLs) observed after proton irradiation in low-grade-glioma (LGG) patients suggest a distal proton RBE larger than 1.1 and an enhanced radiosensitivity of the periventricular brain region (PVR). These findings motivated a prospective, randomized phase-II trial to answer whether considering risk prediction in treatment planning (TP) would allow for toxicity reduction as compared to the outcome after conventional treatment. Addressing this issue calls for a robust TP concept minimizing normal tissue complication probability (NTCP) for the endpoint CEBL occurrence in the experimental arm. The concept was developed on the research version 11BIonPG of RayStation TPS with a built-in variable RBE based optimizer, with parameters tuned to reproduce risk model RBE predictions. 10 clinical LGG patients with different representative target volume localizations, thus different risk factor contributions, were replanned and complication probabilities were evaluated. The TP protocol comprised an active sparing of the PVR as well as measures to control the redistribution of high RBE spots. Resulting local underdosage of the clinical and planning target volume was regulated by posing clinically relevant EUD (equivalent uniform dose) conditions. With the proposed concept, we obtained stable optimization results while successfully eliminating risk hot spots and significantly reducing NTCP compared to conventional TP (see figures). Testing this innovative concept in the prospective randomized trial in over 100 LGG patients constitutes the world-wide first clinical assessment of variable proton-RBE based TP with an expected considerable benefit for brain tumor patients treated with protons.

### O 100 - Extremely hypofractionated proton radiotherapy in the treatment of low and intermediate risk prostate cancer: Long-term outcomes

#### Jiri Kubes^1^, Pavel Vítek^2^, Vladimír Vondráček^2^, Barbora Ondrová^2^, Alexandra Haas^2^, Radek Zapletal^2^, Silvia Sláviková^2^, Kateřina Dědečková^2^, Martin Domanský^2^, Veronika Schlencova^2^, Josef Kvech^2^, Simona Zapletalova^2^

##### ^1^PTC Prague, Proton therapy dept., Prague, Czech Republic ^2^Proton therapy center Czech, Proton therapy, Prague, Czech Republic

**Purpose**: To retrospectively analyze 5-years biochemical free survival (bDFS) and late toxicity profile in patients with prostate cancer treated with pencil beam scanning (PBS) proton radiotherapy.

**Material and Methods**: Between January 2013 and June 2018, 885 patients with prostate cancer were treated with IMPT (intensity modulated proton therapy), with extremely hypofractionated schedule (36.25 GyE/5 fractions). Median of follow up time is 60,9 months. Mean age was 64.7 (40.0-85.7) years, mean PSA value was 5.9 μg/l (0.67-19,71 μg/l), 331 (37.4%), 322 (36.4%) and 230 (26.0%) had low, favorable intermediate and unfavorable intermediate risk cancer, respectively. 204 (23.1%) pts had neoadjuvant hormonal therapy, 7 (0.8%) patients had adjuvant hormonal therapy. bDFS and late toxicity profile were evaluated.

**Results**: Median treatment time was 10 (7-38) days. Estimated 5-years bDFS is 96.5%, 93.7% and 91.2% for low, favorable and unfavorable intermediate risk group, respectively. Late toxicity (CTCAE-v.4) was: Gastrointestinal: G2 - 76 (8.6%), G3 - 4 (0.45%); Genitourinal: G2 - 35 (3.9%), no G3 toxicity was observed. PSA relapse was observed in 58 pts: 16 local, 22 lymph node, 4 bone recurrences and 10 combined sites of relapse were detected. 45 (5.1%) pts. died during FU period, none of them from prostate cancer.

**Conclusion**: Extremely hypofractionated proton beam radiotherapy of prostate cancer is effective with long term bDFS comparable with other techniques for low and favorable intermediate risk prostate cancer and promising for unfavorable intermediate risk prostate cancer, with acceptable long term GI and favorable GU toxicity.

### O 101 - Immune response generated in glioblastoma by proton minibeam radiation therapy (pMBRT) in a preclinical orthotopic rat model

#### Lorea Iturri^1^, Annaïg Bertho^1^, Sarah Potiron^1^, Cristèle Gilbert^1^, Julie Espenon^1^, Fréderic Pouzoulet^2^, Annalisa Patriarca^3^, Ludovic de Marzi^3^, Marjorie Juchaux^1^, Yolanda Prezado^1^

##### ^1^Institut Curie, UMR 3347/U1021 – Signaling- Radiobiology and Cancer, Orsay, France ^2^Institut Curie, Translational Research Department- Experimental Radiotherapy Platform-, Orsay, France ^3^Institut Curie, Centre de Protonthérapie d’Orsay- Radiation Oncology Department, Orsay, France

Proton minibeam radiation therapy (pMBRT) is an preclinical proton therapy technique relying on spatial fractionation. This innovative radiation technique offers to widen the therapeutic index by a heterogeneous dose distribution consisting in high doses (peaks) deposited in the paths of millimetric beams and low doses (valleys) in the rest of the tissue. As a result, pMBRT has shown to enhance tumor control in a glioblastoma rat model while lowering the neurological toxicities associated to high doses of radiation. Nevertheless, the underlying radiobiological mechanisms associated with spatial fractionation efficacy, and most particularly with pMBRT, remain currently unclear and several hypotheses have been proposed, including a more efficient immune attraction to the tumor. In order to fill this gap in knowledge, we performed an extensive characterization of the immune response generated by pMBRT in a syngeneic rat model of glioblastoma compared to conventional proton therapy. Using state-of-the-art technologies such as high-parameter flow cytometry, inflammatory cytokine quantification and single cell transcriptomic analysis of tumor infiltrating leukocytes (single cell RNA sequencing, 10x genomics), we observed that pMBRT elicited an acute inflammatory phenotype in the tumor and the surrounding healthy tissue, while attracting a vast number of lymphocytes to the tumor microenvironment. Proton therapy is a rapidly advancing field in cancer treatment due to the encouraging characteristics of protons, yet there is a need for better understanding the radiobiological effects generated in the tissue; specifically involving spatial fractionation. Importantly, evaluating immune responses to radiotherapy will give vital information for future studies combining radio-immunotherapies prior successful clinical trials.

### O 102 - Infrared microespectroscopy evaluation of the biochemical effects of proton minibeam radiation therapy in vitro and in vivo

#### Roberto González-Vegas^1^, Ibraheem Yousef^2^, Olivier Seksek^3^, Ramon Ortiz^4^, Annaïg Bertho^4^, Marjorie Juchaux^4^, Ludovic De Marzi^5^, Annalisa Patriarca^5^, Yolanda Prezado^4^, Immaculada Martínez-Rovira^1^

##### ^1^Autonomous University of Barcelona, Department of Physics, Cerdanyola del Vallès, Spain ^2^ALBA-CELLS Synchrotron, BL01 - MIRAS Beamline, Cerdanyola del Vallès, Spain ^3^Centre National de la Recherche Scientifique, IJCLab, Orsay, France ^4^Institut Curie, Signalisation Radiobiologie et Cancer, Orsay, France ^5^Institut Curie - Centre de Protonthérapie d’Orsay, Radiation Oncology, Orsay, France

**Background**: Proton Minibeam Radiation Therapy (pMBRT) employs arrays of submillimetric proton beamlets, allowing to use high doses while sparing healthy tissue and maintaining tumor control. This study aims to report on the biological processes underlying pMBRT using Fourier transform infrared microspectroscopy (FTIRM).

**Materials and Methods**: Radiotherapy irradiations (100 MeV proton beams) were performed at Institut Curie Proton Therapy Center. Both *in vitro* (CTX-TNA2 rat astrocytes and F98 glioma cell lines; 5 to 20 Gy) and *in vivo* (male Fischer rats; whole brain irradiations excluding the olfactory bulb; 25 Gy) studies were performed. FTIRM measurements were conducted at the MIRAS beamline of ALBA Synchrotron at 24 hours (cells) and 2 hours, 48 hours and 15 days (tissue) post-radiotherapy. Data analysis included principal component analysis (PCA) and the assessment of the probability density of several spectral ratios, as well as biochemical imaging on tissue sections.

**Results**: Preliminary PCA in the fingerprint region revealed a group separation in both astrocytes and glioma cells, mainly from changes in the protein region. Depending on the irradiation configuration, different trends were observed in the amide I and phosphates I and II to amide II ratios. In the lipids, distinct yields for the broad beam and pMBRT peak doses were observed in the evaluated spectral ratios. Results were influenced by the irradiation configuration. Biochemical maps of the tissue sections are under evaluation.

**Conclusions**: This is the first study reporting biochemical changes arising from pMBRT irradiations, both *in vitro* and *in vivo*, using FTIRM. Further analysis is still ongoing.

### O 103 - Planar proton minibeam irradiation elicits spatially confined DNA damage in a human epidermis model

#### Harry Scherthan^1^, Stephanie-Quinta Wagner^1^, Lisa Fees^1^, Matejka Nicole^2^, Jessica Neubauer^2^, Sarah Rudigkeit^2^, Matthias Port^1^, Judith Reindl^2^

##### ^1^Institut für Radiobiologie der Bundeswehr in Verb. mit der Universität Ulm, Radiobiology, München, Germany ^2^Universität der Bundeswehr München, Institute for applied physics and measurement technology, München, Germany

High doses of ionizing radiation in radiotherapy can elicit undesirable side effects to the skin. Proton minibeam radiotherapy may circumvent such limitations due to the dose-volume effect, describing tissue-sparing observed at the macro scale by the use of sub-millimeter sized beams. Here, we mapped DNA damage dynamics in a 3D tissue context at the sub-cellular level. Epidermis models were irradiated with planar proton minibeams of 66 µm, 408 µm and 920 µm widths (sigma) and inter-beam-distances of 2.5 mm at an average dose of 2 Gy using the microbeam platform at the ion-microprobe SNAKE in Garching, GER. γ-H2AX + 53BP1 and cleaved-caspase-3 immunostaining revealed double-strand DNA damage and cell death, respectively, in time courses from 0.5 to 72 h after irradiation. Focused 66 µm proton minibeams induced sharply localized severe DNA damage (pan-γ-H2AX) in cells at the dose peaks, while damage in the dose valleys was similar to sham control. 408 µm and 920 µm minibeams induced DSB foci in all cells. At 72 h after irradiation, DNA damage had reached sham levels, indicating successful DNA repair. Increased frequencies of active-caspase-3 and pan-γ-H2AX-positive cells revealed incipient cell death at late time points. The spatially confined distribution of DNA damage appears to underlie the tissue-sparing effect after focused proton minibeam radiotherapy. Thus, it may be the method of choice in radiotherapy to reduce side effects to the skin. Furthermore, the damage distribution visible in the skin seems to be promising to use minibeams to reduce side effects in other tissue, too.

### O 104 - On the influence of the irradiation parameters in proton minibeam radiation therapy (pMBRT) on the biological response

#### Sarah Potiron^1^, Juchaux Marjorie^1^, Bertho Annaïg^1^, Gilbert Cristèle^1^, Espenon Julie^1^, Patriarca Annalisa^2^, De Marzi Ludovic^2^, Iturri Lorea^1^, Prezado Yolanda^1^

##### ^1^Institut Curie, New Approaches in Radiotherapy Team, Orsay, France ^2^Institut Curie, Protontherapy Center of Orsay, Orsay, France

Proton minibeam radiation therapy (pMBRT) is a preclinical spatially fractionated radiotherapy which is characterized by heterogeneous dose distributions [1]. pMBRT has shown a remarkable normal tissue sparing compared to conventional proton therapy (CPT) [2] and leads to an equivalent or superior tumor control [3]. While the link between the pMBRT parameters (peak and valley doses, beam widths and spacing) and biological response is not completely elucidated, a retrospective evaluation revealed that the valley doses have the highest correlation with increased lifespan in tumor-bearing animals [4]. In addition, X-rays MBRT showed a dependence of T cells and a great infiltration in tumor microenvironment [5]. Therefore, we wanted to investigate whether the valley dose influence the immune priming. For that purpose, we performed multiparameter flow cytometry on tumor from an orthotopic and syngeneic rat glioblastoma model at 7 days post-irradiation. The center-to-center distance was modulated so that the different groups received equal valley dose. Among groups, survival curves were statistically equivalent, as the immune infiltration. This suggests that valley doses are likely governing the increase lifespan with the immune system playing a major role. The observation of a significant increase of long-term survivals tumor-free indicates the important function of peak dose (maintaining equal valley dose) and additional effects are contributing. Along this line, evaluations of the impact of pMBRT and CPT and different parameter on the vasculature are ongoing and be presented. This study contributes to our understanding of the biological mechanisms in response to different configurations of pMBRT.

### O 105 - Experimental magnetic benchmarking of a proton pencil beam scanning nozzle model for development of MR-integrated proton therapy

#### Ekaterina Semioshkina^1^, Aswin Hoffmann^1^, Brad M. Oborn^2^

##### ^1^Helmholtz-Zentrum Dresden-Rossendorf, Institute of Radiooncology – OncoRay, Dresden, Germany ^2^University of Wollongong, Centre for Medical Radiation Physics, Wollongong, Australia

**Introduction**: It is expected that integration of MR imaging (MRI) with proton therapy (PT) will improve the targeting accuracy of PT for moving tumours. This work presents a benchmarked magnetic model of a proton pencil beam scanning (PBS) beamline for assistance in predicting the magnetic interference between the PBS and an in-beam MRI system.

**Methods**: A COMSOL finite element model (FEM) of a horizontal proton PBS beamline was created based on vendor provided information. This included the beam focusing quadrupole magnet pair, X and Y beam scanning magnets and all nearby steel supporting structures. The model was solved for various beam scanning parameters (70-230 MeV, dose spot positions X=0-20 cm and Y=0-15 cm). A selection of these simulations was experimentally validated. The magnetic fringe field of the PBS assembly was measured using a 3D magnetometer at measurements locations where the MRI scanner will be positioned (see figure 1).

**Results**: The magnetic field within the focusing and scanning magnets yoke gaps predicted by the FEM matched the vendor notes to within +/− 1%. The experimental fringe field measurements showed the field produced around the beam isocenter (ISO) [AH1] ranges from 0-8 μT, which matches within 1 μT of COMSOL values (see figure 2).

**Conclusion**: A detailed magnetic model of a proton PBS beamline has been successfully created and experimentally verified. This model will be invaluable tool to assess magnetic interference and design magnetic shielding to decouple the PBS and MRI magnetic fields.

### O 106 - Development and characterization of a proton and carbon ions counter for particle therapy applications

#### Emanuele Maria Data^1,2^, Felix Mas Milian^1,2,3^, Simona Giordanengo^2^, Mohammed Abujami^1,2^, Marco Donetti^4^, Oscar Ariel Martì Villareal^1,2^, Anna Vignati^1,2^, Roberto Cirio^1,2^, Vincenzo Monaco^1,2^, Roberto Sacchi^1,2^

##### ^1^University of Torino, Department of Physics, Torino, Italy ^2^Istituto Nazionale di Fisica Nucleare, Sezione di Torino, Torino, Italy ^3^Universitade Estadual de Santa Cruz, Physics, Ilheus, Brazil ^4^Centro Nazionale di Adroterapia Oncologica, cnao, Pavia, Italy

An innovative beam monitor for particle therapy was developed to count single protons and carbon ions in the clinical energy range. The designed detector exploits thin silicon sensors, which show sensitivity to single particles and fast charge collection times allowing to reach large counting rates. A 60 μm thick diode sensor and a 50 μm thick Low Gain Avalanche Diode sensor are used for detecting respectively protons and carbon ions. The sensitive area of both the sensors is 2.6 × 2.6 cm^2^, enough to cover the cross section of a pencil beam, and it is segmented in 146 strips with 180 μm pitch. The readout is based on 6 custom 24-channel front-end ASICs able to discriminate particle signals in a wide charge range (4-150 fC) with a maximum dead time of about 10 ns. The digital pulses produced by the discriminator are sent to 3 Kintex7 FPGA boards implementing counters for each channel, while a LabVIEW program reads and saves the data for offline analysis. The measurements performed with protons and carbon ions in CNAO (Pavia) result in beam projections (fig.1) with a FWHM comparable with measurements performed with gafchromic films. The proton counting efficiency shows a dependence on the beam energy because of geometric and pile-up effects (fig.2), whereas an efficiency above 90% with lower energy dependence is found for carbon ions. A pile-up correction method implemented in the readout will be presented. The results indicate the feasibility and limitations of directly measuring particle rate and beam shape.

### O 107 - Experimental characterization of collimator produced carbon ion minibeams with high resolution CMOS sensors

#### Lennart Volz^1^, Claire-Anne Reidel^1^, Christoph Schuy^1^, Yolanda Prezado^2,3^, Marco Durante^1,4^, Uli Weber^1^, Christian Graeff^1,5^

##### ^1^GSI Helmholtz Center for Heavy Ion Research GmbH, Biophysics, Darmstadt, Germany ^2^Institut Curie-Université PSL, CNRS UMR3347- Inserm U1021- Signalisation Radiobiologie et Cancer, Orsay, France ^3^Université Paris-Saclay, CNRS UMR3347-Inserm U1021- Signalisation Radiobiologie et Cancer, Orsay, France ^4^TU Darmstadt, Institute for Condensed Matter Physics, Darmstadt, Germany ^5^TU Darmstadt, Department of Electrical Engineering and Information Technology, Darmstadt, Germany

Ongoing experiments on heavy ion minibeam therapy rely on collimators to produce beamlets with the desired width and center-to-center distance (ctc). Interpretation of these experiments requires a solid understanding of the minibeam characteristics, especially in terms of secondary fragments and LET. We explore high-resolution CMOS sensors for beam characterization and introduce a new concept for neutron dose reduction. Measurements were acquired at the Marburg Ion Beam Therapy Center with carbon ion beams (183MeV/n, σ=5.3mm) using a brass collimator (3.5mm ctc; 600μm slit width) and six MIMOSA-28 sensors. Three sensors were positioned in front, and three behind the collimator. Primary and secondary particles were identified from the measured charge clusters; the track-averaged LET is proportional to the cluster size distribution. An alignment robustness analysis was performed. The setup was replicated in Geant4, enabling an extended parameter analysis. Neutron shielding with a PE collimator attached to the brass collimator was tested in simulation. The CMOS sensors proved to enable high-resolution fluence profile characterization (Figure 1a/b). The cluster size analysis showed a high LET beam component in the center of the dose valleys (Figure 1c). Rotational misalignment decreased primary fluence and introduced shoulders in the dose profiles (Figure 1d/e). The neutron shield (Figure 2) achieved a 20% reduction in peak neutron dose in simulations. This work demonstrates the potential of CMOS sensors for in-depth characterization of minibeam collimators. The fully characterized minibeam collimator has been used in recent pre-clinical heavy ion minibeam experiments at GSI. An easy-to-manufacture PE collimator can reduce neutron dose to the patient.

### O 108 - Implementation of a beam shaping system designed for an accelerator-based BNCT system optimised for head and neck cancer

#### Naonori Hu^1,2^, Ryo Kakino^1^, Kazuhiko Akita^1^, Syuushi Yoshikawa^1^, Mamoru Miyao^3^, Satoshi Takeno^4^, Teruhito Aihara^1^, Keiji Nihei^4^, Hiroki Tanaka^2^, Koji Ono^1^

##### ^1^Osaka Medical and Pharmaceutical University, Kansai BNCT Medical Center, Takatsukishi, Japan ^2^Kyoto University Institute for Integrated Radiation and Nuclear Science, Particle Radiation Oncology Research Center, Sennan-gun, Japan ^3^Osaka Medical and Pharmaceutical University Hospital, Central department of Radiology, Takatsukishi, Japan ^4^Osaka Medical and Pharmaceutical University Hospital, Department of Radiation Oncology, Takatsukishi, Japan

BNCT is usually performed in a single fraction, with the neutron irradiation time being approximately 30-60 minutes. It is preferable to bring the patient as close to the beam port as possible to keep the irradiation time short. In this study, a novel neutron collimation system for an accelerator based neutron source was designed to allow for a more comfortable treatment, while also reducing the treatment time. Two types of collimators were designed, a 5 cm and 10 cm extended collimator. Monte Carlo simulation package PHITS was used to model the conical collimators and simulation was performed to optimise the collimator design. Experimental measurements confirmed the simulation results and showed the proposed collimator design can reduce the irradiation time by approximately 60%. The dose delivered to the surrounding healthy tissue was reduced with the new collimator, showing a 25% decrease in the D_50%_ of the mucosal membrane. The extended collimators significantly reduced the irradiation time, and the treatment would be performed in a much comfortable position.

### O 109 - A novel dynamic craniospinal irradiation (CSI) technique

#### Lewei Zhao^1^, An Qin^1^, Gang Liu^2^, Peilin Liu^1^, Xiaoqiang Li^1^, Xuanfeng Ding^1^

##### ^1^Corewell Health William Beaumont University Hospital, Radiation Oncology, Royal Oak, USA ^2^Huazhong University of Science and Technology, Cancer Center- Union Hospital- Tongji Medical College, Wuhan, China

**Purpose/Objectives**: To improve the CSI pediatric patient treatment efficiency, we introduce a new treatment modality via continuous gantry and couch movement (CSI_-dynamic_).

**Materials and Methods**: This novel treatment is designed to deliver spot-scanning proton arc (SPArc) for the brain irradiation, followed by a dynamic couch motion in the spine irradiation (Figure 1A). Machine delivery sequence model of the IBA system was used to resample the nominal plan into hundreds of control points mimicking a dynamic couch continuous treatment delivery. The dynamic SPArc for brain irradiation - hippocampus sparing is generated based on the background dose optimization of spine irradiation. Delivery time of CSI_-dynamic_ was simulated and compared to the clinical treatment time based on the Mosaiq record.

**Results**: The CSI_-dynamic_ treatment takes 11 mins compared to 29 mins using clinical IMPT. Significant efficiency improvement was found in three areas (1) the field-by-field loading and communication between OIS and proton system; (2) Couch shifting time for the multi-iso setup; (3) imaging verficiation time of each iso. Static irradiation time is comparable (633s vs 564s) between the CSI_-dynamic_ and clinical IMPT. Gantry and couch movement speed was ploted accordingly, achieving a smooth mechanical connection (Figure 1B and 1C). Additionally, this novel CSI_-dynamic_ can significantly improve dosimetric outcome for hippocampus sparing, which can potentially preserve cognitive function (Figure 2).

**Conclusion**: The CSI_-dynamic_ technique with continuous gantry and couch movement could improve the treatment efficiency and the dosimetric plan quality for the hippocampus sparing which could be important to the pediatric patients.

### O 110 - Proton FLASH-RT to the spinal cord in Yucatan minipigs: A framework and dosimetric characteristics of a vendor’s proton machine

#### Mingyao Zhu^1^, Katja Langen^1^, Anees Dhabaan^1^, Sibo Tian^1^, Soumon Rudra^1^, Michael Folkerts^2^, Jeffrey Bradley^1^

##### ^1^Emory University School of Medicine, Radiation Oncology, Atlanta, USA ^2^Varian, Flash R&D, Palo Alto, USA

**Purpose**: To report a framework of proton FLASH-RT research in irradiating the C5 spinal cord in Yucatan minipigs using a ProBeam® in service-mode, and to present the dosimetric characteristics at FLASH and conventional dose rates.

**Methods**: The animals were simulated head-first-prone, under anesthesia, with a Siemens CT scanner. All the animals were irradiated with a right-lateral PBS field consisting of 7x7 (5mm apart) 250MeV proton spots. The spot MU and nozzle current were adjusted to achieve desired dose and dose rate and were calibrated on the day of experiment. A dummy plan was created to allow kV and CBCT image guidance (Figure1). A parallel-plate ion-chamber was placed 20cm downstream from the isocenter; the delivered dose was derived from the measured exit-dose. Voxel-wise dose rates were calculated using the Varian® FLEX TPS.

**Results**: To date, 13 animals (8 FLASH, 5 conventional) have been irradiated at 17.5Gy (4), 20.0Gy (6) and 22.5Gy (3) prescribed dose. It took ∼2 hours per animal from induction to treatment, resulting in 3 minipig irradiations per day. The delivered C5 spinal cord mean doses were within -1.26% to 3.45% of the Rx dose. The mean dose rates were 64±2.5 Gy/s and 0.36±0.01 Gy/s for FLASH and conventional dose rates, respectively. Voxel-wise dose rates in the C5 spinal cord were at least 70Gy/s for all dose levels (Figure2).

**Conclusion**: The ProBeam® system can deliver efficient and reliable PBS FLASH-RT in service-mode; it is feasible to simulate, treatment plan, and irradiate the spinal cord in multiple minipigs in one day.

### O 111 - A real-time, large-area transparent FLASH beam monitor and dosimeter

#### P.S. Friedman^1^, Vladimir Bashkirov^2^, Claudio Ferretti^3^, Daniel Levin^3^, Dale Litzenberg^4^, Nicholas Ristow^3^, Reinhard Schulte^2^, Monica Tecchio^3^

##### ^1^Integrated Sensors- LLC, R&D, Palm Beach Gardens, USA ^2^Loma Linda University, Biomedical Engineering Sciences, Loma Linda, USA ^3^University of Michigan, Physics, Ann Arbor, USA ^4^University of Michigan, Radiation Oncology, Ann Arbor, USA

A novel large-area system for real-time beam monitoring and dosimetry for all FLASH-radiotherapy modalities is being developed. It is based on a recently patented 2D beam imaging detector, with a prototype already tested. The system utilizes a machine-vision camera, folded optics, and novel, proprietary low-mass (<1 mm water-equivalent) 30x30 cm^2^ scintillators providing excellent radiation hardness. The ultrafast camera acquires data at up to 20,000 frames/s and continuously streams and analyzes each image in ≤50 µs for beam position, profile, and dosimetry during treatment. The system provides a linear response to particle flux over the FLASH dose-rate range and can achieve <50 µm spatial resolution for beam position and profiles. It features a thin profile and a rapid internal calibration system. A prototype system has been tested at the University of Michigan Hospital and the University of Notre Dame, the latter with an 8 MeV electron beam delivering 1.9 Gy per 2 ns pulse (i.e., an instantaneous dose rate of >900 MGy/s). The system can also analyze sporadic dose-rate spikes of <50 µs from synchrotron accelerators for proton and carbon-ion therapy and can be adapted for mini-beams and BNCT. This FLASH-enabling beam monitor provides a large-area, high accuracy, ultrafast beam position and profile analysis, and dosimetry with real-time verification during delivery to ensure patient safety with an application-specific response time of 0.1 to 2.5 ms (i.e., tailored to beam pulse structure).

### O 112 - Dosimetry, normal tissue sparing, and tumor control with proton pencil beam scanning (PBS) FLASH radiotherapy

#### Michele Kim^1^, Ioannis Verginadis^1^, Seyyedeh Azar Oliaei Motlagh^1^, Beryl Bell^2^, Francois Vander Stappen^2^, Wei Zou^1^, Costas Koumenis^1^, Lei Dong^1^, Eric Diffenderfer^1^

##### ^1^University of Pennsylvania, Department of Radiation Oncology, Philadelphia, USA ^2^Ion Beam Applications S.A., Research and Development, Louvain-la-Neuve, Belgium

**Background and Aims**: The goal of this study was to determine the capabilities of a gantry room with pencil beam scanning (PBS) for FLASH proton radiotherapy (F-PRT) and compare normal tissue sparing and tumor control capabilities with double scattered (DS) F-PRT for various definitions of dose rate.

**Methods**: The FLASH PBS beam was characterized for a gantry room. A treatment plan was designed with 4x4 pencil beam spots with 5mm spot spacing. A 2x2cm^2^ collimator was placed after the range shifter to ensure a sharp field penumbra. Effective field dose rate (EFDR) (or mean dose rate) was determined using a NIST-traceable Advanced Markus Chamber. Dose delivery characteristics were analyzed using machine log files. Whole abdomen of female C57BL/6J mice were irradiated to a dose of 15 Gy with one of four groups: (1) PBS F-PRT 167 Gy/s EFDR, (2) PBS F-PRT 112 Gy/s EFDR, (3) PBS standard dose rate (S-PRT) 1.6 Gy/s EFDR, (4) DS S-PRT 1.0 Gy/s EFDR. Using methods previously developed, EdU-positive proliferating cells and regenerated intestinal crypts were quantified.

**Results**: A maximum EFDR of 167 Gy/s was achieved using a cyclotron current of 800nA with spot durations of 3ms. Log files show beam stability between irradiations with maximum variation in field delivery time of 2.75ms. Equivalent normal tissue sparing (a)and tumor control (b) was observed for PBS and DS F-PRT (Fig. 1).

**Conclusion**: PBS F-PRT was demonstrated in a clinical treatment room, providing key evidence needed for safe and effective clinical translation of FLASH radiotherapy.

### O 113 - Proton Bragg peak FLASH-RT can be realized using a superconducting gantry with large momentum acceptance and universal range shifters

#### Yiling Zeng^1^, Bo Pang^1^, Wei Wang^2^, Xu Liu^2^, Bin Qin^2^, Zhiyong Yang^1^

##### ^1^Huazhong University of Science and Technology, Union Hospital, Wuhan, China ^2^Huazhong University of Science and Technology, Electrical and Electronic Engineering, Wuhan, China

**Purpose**: By pulling back the ranges of high energy protons using a universal range shifter (URS), the feasibility of multiple-energy Bragg peak FLASH-RT with a superconducting gantry with large momentum acceptance (LMA-SC gantry) has been investigated in this study.

**Method and Materials**: A simultaneous dose and spot map optimization algorithm was developed for Bragg peak FLASH-RT treatment planning. Bragg peak plans with URS and transmission beam plans were optimized for 10 prostate cancer patients and 10 lung cancer patients. The plan delivery parameters, dose metrics and dose rate metrics were evaluated with the parameter of LMA-SC gantry.

**Results**: In comparison of the transmission plans, Bragg peak plans achieved significantly better target conformity for prostate and lung cases. Bragg peak plans had better normal sparing with a significant reduction of mean dose in normal tissues of 39.1% and 45.0% for prostate and lung cases compared to transmission plans, respectively. While the Bragg peak plans yield lower overall FLASH dose rate coverage (> 40 Gy/s) of 8.4% and 19.4% in normal tissues for prostate (P = .001) and lung (P = .001) cases, respectively. However, except for the low dose region (≤1Gy), the Bragg peak plans yielded comparable FLASH dose rate coverage to transmission plans.

**Conclusions**: Multiple-energy Bragg peak FLASH-RT can be realized using the URS and LMA-SC gantry. Moreover, Bragg peak plans can achieve better normal tissue sparing and target conformity compared with transmission plans.

### O 114 - Near-field measurements and simulations for proton beam FLASH with 3D-range-modulators: Could there be an additional benefit from a minibeam effect?

#### Warisara Charuchinda^1,2^, Claire-Anne Reidel^1^, Klemens Zink^3^, Christoph Schuy^1^, Yuri Simeonov^3^, Felix Horst^3^, Per Poulsen^4^, Simon Busold^5^, Marco Durante^1^, Uli Weber^1^

##### ^1^GSI Helmholtzzentrum für Schwerionenforschung, Biopyhsics, Darmstadt, Germany ^2^Chulalongkorn University, Department of Physics, Bangkok, Thailand ^3^THM, Institute for Medical Physics and Radiation Protection, Gießen, Germany ^4^Aarhus University Hospital and Proton Centre, Department of Oncology, Aarhus, Denmark ^5^Varian Medical Systems, Proton Therapy, Troisdorf, Germany

The 3D range-modulator (3DRM) is a device used in particle delivery systems that can create a highly conformal and homogeneous dose distribution in the target volume with mono-energetic beams, providing an option for FLASH therapy. Usually, the modulators are positioned at a typical distance of 30-50 cm in front of the target in order to avoid the fluence ripples resulting from their periodic structures (pins). We performed different measurements with proton beams to characterize the near-field behind the 3DRM with pins of ca. 50 mm height and 3mm period. First, we used a stack of radiochromic films. Second, we applied a stack of CMOS sensors (Mimosa-28) with extremely high resolution (20μm pixel size) and thus could precisely evaluate the 3D fluence distribution of the modulated beam in the range of interest (Figure 1-2). Additionally, FLUKA Monte Carlo simulations were performed to investigate the fluence distributions of protons penetrating through the 3D range modulators in air or water and to determine the minimum distance at which the fluence is deemed homogeneous enough for treatment (Figure 1). Systematic studies of different spacing and modulator strengths were carried out for this purpose. Prospectively, instead of conservatively keeping the dose on the skin homogeneous, a strong dose inhomogeneity (having a peak-to-valley dose ratio of typically 2-3) could be applied in the proximal part of the target if low modulator-to-target distances are used. This parasitic effect could introduce additionally a kind of “minibeam” normal-tissue sparing. The potential benefit also will be discussed in the presentation.

### O 115 - Proton therapy re-irradiation provides promising clinical results in recurrent brain meningioma

#### Daniele Scartoni^1^, Irene Giacomelli^1^, Riccardo Pertile^2^, Sabina Vennarini^3^, Paola Feraco^4^, Lorena Picori^5^, Luciano Annicchiarico^6^, Silvio Sarubbo^6^, Dante Amelio^1^

##### ^1^APSS - Trento, Proton Therapy Center, Trento, Italy ^2^APSS - Trento, Clinical and Evaluative Epidemiology, Trento, Italy ^3^Fondazione IRCCS Istituto Nazionale dei Tumori, Pediatric Radiotherapy Unit, Milano, Italy ^4^APSS - Trento, Neuroradiology Unit, Trento, Italy ^5^APSS - Trento, Nuclear Medicine, Trento, Italy ^6^APSS - Trento, Neurosurgery, Trento, Italy

**Aim**: To evaluate the clinical outcomes, toxicity and prognostic factors conditioning survival of reirradiation with Proton therapy for recurrent meningiomas.

**Methods**: Recurrent meningioma who failed after radiotherapy were re-irradiated with active scanning proton therapy with a median dose of 54 GyRBE (range 50.4-60 GyRBE). Progression-free survival (PFS) and overall survival (OS) were estimated using the Kaplan-Meier method. The log rank test was performed to compare the entire survival experience between groups (significant p-value ≤ 0.05). Associations between qualitative variables were analyzed with the Fisher exact test (significant p-value ≤ 0.05).

**Results**: Thirty-two patients were re-irradiated with Proton therapy (10 grade I, 22 grade II-III). Median tumor volume at the time of re-irradiation was 43 cc (range 1.2-225.5) and median time from prior RT to reirradiation was 66 months (range 4-288). At a median follow-up of 27 months, 1-yr and 2-yr PFS were 89.4% and 74.5% (1-yr and 2-yr PFS were 100% for grade I, 100% and 76% for grade II, 66% and 50% for grade III). 1-yr and 2-yr OS were 86.6% and 83.0% (2-yr OS were 87% for grade I, 86% for grade II, 66% for grade III). Local recurrence rate was 34% with a median time of recurrence of 13.4 months. Five patients (14%) developed radionecrosis with a median time of 3.4 months from the end of PT (range 3-8.8).

**Conclusions**: Re-irradiation with proton therapy seems to be a safe and effective treatment in patients with recurrent meningioma, also for large volume and aggressive lesions (grade II-III).

### O 116 - Repeated carbon ion radiotherapy for hepatocellular carcinoma

#### Hiroyuki Katoh^1^, Hiroaki Koge^1^, Kio Kano^1^, Satoshi Shima^1^, Keisuke Tsuchida^1^, Yosuke Takakusagi^1^, Nobutaka Mizoguchi^1^, Daisaku Yoshida^1^

##### ^1^Kanagawa Cancer Center, Department of Radiation Oncology, Yokohama, Japan

**Introduction**: Hepatocellular carcinoma (HCC) is characterized by multicentricity, and although carbon ion radiotherapy (CIRT) has been shown to be effective, it often requires repeat treatments. We analyzed cases of HCC treated with repeated CIRT at our facility.

**Materials and Methods**: Fifteen patients who had undergone repeated CIRT procedures for HCC at our facility between 2017 and 2022 and had been followed up for at least 3 months were included. The median age of the patients was 70.6 (51.8-83.4) year old, and there were 11 male and 4 female patients. The median tumor diameter of the first lesion was 35 (10-94) mm, and the median tumor diameter of the second and subsequent lesions was 25 (11-46) mm. A total of 2 CIRTs were performed in 12 patients, 3 in 2 patients, and 4 in 1 patient, and in all, 34 lesions were treated with CIRT.

**Results**: The median observation period from the first treatment was 50.9 months. The median interval between CIRTs was 28.7 (4.6-61.6) months. The 3-year overall survival rates from the first and second treatments were 84.8% and 55.6%, respectively (p=0.059). The pre-treatment Child-Pugh (CP) classification was A, B, and C in 30, 3, and 1 cases, respectively. Three months after treatment, CP scores increased in 4 cases, decreased in 4 cases, and the other 26 cases had no change.

**Conclusion**: Repeated CIRT for HCC did not cause obvious deterioration of liver function, suggesting that multiple CIRT to the liver is well tolerated.

### O 117 - Early outcomes of normo- and moderately hypofractionated proton re-irradiation in recurrent head and neck tumors

#### Maciej Pelak^1^, Piero Fossati^1^, Slavisa Tubin^1^, Petra Mozes^1^, Petra Georg^2^, Rastko Konstantinovic^3^, Christoph Fussl^4^, Joanna Gora^1^, Ana Perpar^5^, Ankita Nachankar^1^, Carola Lütgendorf-Caucig^1^, Eugen Hug^1^

##### ^1^MedAustron, Ion Therapy Center, Wiener Neustadt, Austria ^2^Universitätsklinikum Krems, Radiation Oncology, Krems, Austria ^3^Salzkammergut Klinikum Vöcklabruck, Radiation Oncology, Vöcklabrück, Austria ^4^Uniklinikum Salzburg, Radiation Oncology, Salzburg, Austria ^5^Institute of Oncology Ljubljana, Radiation Oncology, Ljubjana, Slovenia

**Purpose/Objective**: Proton beam therapy (PBT) has shown promising results in salvage re-irradiation of head and neck tumors. Hypofractionation can shorten treatment time and impose a stronger biological effect on tumor compared to normofractionated regime. We analyzed the outcome of our patients treated with both schemes.

**Material and Methods**: Thirty-eight patients treated at our institution with PBT with normofractionated (nFx, n = 17) and moderately hypofractionated proton therapy (hypoFx, n = 21) between 2017 and 2021 were included. Majority of patients (92.1%) had macroscopic disease present and the most common tumor type was squamous cell carcinoma (68.4%). Only 2 patients received concomitant systemic treatment. The patient characteristics are displayed in Table 1.

**Results**: The median follow-up period was 17 months (1.7 – 49.3). The actuarial 1- and 2-year local control was 85.6%/70.2%, regional control was 79.9%/64.1%, progression-free survival was 55.8%/34% and overall survival was 68.3%/51.9%, respectively. The dominating pattern of failure was progression in irradiated field (9/17 cases of tumor progression, 52.9%). A significant trend towards better local control in hypoFx patients could be observed (overall LC 58.2% for nFx vs 90.5% for hypoFx, HR = 0.15, 95%CI: 0.02-0.86, p = 0.034) and high-dose PTV volume > 60 cm3 was a significant risk factor for locoregional progression (Figure 1). 11 patients (28.9%) developed ≥ G3 serious late complications, mostly soft tissue necrosis and infections which eventually improved in 6 of them.

**Conclusion**: Re-irradiation with PBT is a valuable option for patients with locally recurrent head and neck tumors after previous radiotherapy.

### O 118 - Outcomes and technical aspects of proton re-irradiation for ocular malignancies

#### Sumi Sinha^1^, Carly Zako^1^, Jessica Scholey^1^, Tomi Nano^1^, Kruthi Battar^2^, Armin Afshar^3^, Susanna Park^4^, Isabella Phan^5^, Devron Char^2^, Kavita Mishra^1^

##### ^1^University of California San Francisco, Radiation Oncology, San Francisco, USA ^2^The Tumori Foundation, Ophthalmic Oncology and Orbital Surgery, San Francisco, USA ^3^University of California San Francisco, Department of Ophthalmology, San Francisco, USA ^4^University of Davis California, Department of Ophthalmology & Vision Science, Sacramento, USA ^5^Kaiser Permanente San Francisco Medical Center, Ophthalmology, San Francisco, USA

**Background**: Radiotherapy for uveal melanoma and other ocular malignancies has demonstrated excellent success in local control and ophthalmic outcomes over the past 50 years. Salvage for local failure is medically and technically challenging; proton therapy presents a unique treatment option.

**Methods**: A retrospective review of n=2,344 patients treated with proton therapy for ocular conditions at a single institution (5/1994-11/2022) were reviewed and patients receiving proton re-irradiation after prior proton (n=16) or plaque (n=12) treatment with a minimum 3 months follow up were selected. Local control (LC) and overall survival (OS) estimates were calculated using the Kaplan-Meier method.

**Results**: The cohort median age was 67 years (range 41-86 years) and included 36% females. Median time between treatments was 4.1 years (range 0.8-14.4 years). Median tumor height and largest tumor dimension were 4.65mm (interquartile range 3.1-5.7mm) and 12.0mm (9.8mm-16.4mm) respectively. The most common re-irradiation prescription was 56 GyE in 4 fractions (92.9% patients) and all patients were treated for recurrent melanoma of the eye. Median follow up time after re-irradiation course was 3.2 years. The 3-year local control and overall survival after re-irradiation were 86.2% (95% confidence interval 62.4-95.5%) and 80.5% (95% CI 59.2-91.5%) respectively (Figure 1). The 3-year enucleation rate was 7.7% (95% CI 72.6-98.0%).

**Conclusions**: Here we review the safety and efficacy of re-irradiation of the eye with proton therapy in a single institution cohort and, to our knowledge, the first-ever published review of protons following plaques. Overall, re-irradiation for salvage of ocular tumors should be considered in select patients.

### O 119 - Safety and efficacy in multiple courses of carbon-ion radiotherapy (CIRT) for intrahepatic recurrent hepatocellular carcinoma

#### Tatsuya Ohno^1^, Tomizawa Kento^1^, Shibuya Kei^1^, Shiba Shintaro^1^, Okazaki Shohei^1^, Miyasaka Yuhei^1^, Okamoto Masahiko^1^

##### ^1^Gunma University, Gunma University Heavy Ion Medical Center, Maebashi, Japan

**Purpose**: To evaluate the safety and efficacy in multiple courses of carbon-ion radiotherapy (CIRT) for intrahepatic recurrent hepatocellular carcinoma (HCC).

**Method and Materials**: We retrospectively reviewed HCC patients who received repeated CIRT for intrahepatic recurrent HCC between 2010 and 2020.

**Results**: Forty-one patients had multiple courses of CIRT for HCC. During the second course, 17 of 41 patients (41.5%) underwent CIRT for local recurrence following the first irradiation. The median age at the first course was 76 years, and the median tumor size in all courses was 25 mm. The prescribed dose was 52.8 or 60.0 Gy (relative biological effectiveness) delivered in 4 or 12 fractions. The median follow-up period after first and second CIRT was 40 and 21 months. Median overall survival (OS) after first and second CIRT were 80 and 27 months. The 2- and 5-year OS rates after first CIRT were 88 and 50%, and the 2-year OS rate after second CIRT was 56%. The 1- and 2-year local control after second CIRT were 93% and 83%. There were no significant differences of local control between local recurrence and out-of-field recurrence groups (84% vs 81%, p = 0.83). The albumin–bilirubin scores at 3 and 6 months after second CIRT were not significantly different from those before second CIRT. No patients developed Grade 4 or greater toxicities according to the Common Terminology Criteria for Adverse Events version 4.0.

**Conclusions**: Multiple courses of CIRT for intrahepatic recurrent HCC demonstrated favorable tumor control with acceptable toxicities.

### O 120 - Microdosimetry in BNCT: An experimental tool for beam quality monitoring

#### Anna Selva^1^, Anna Bianchi^1^, Luca Bellan^1^, Enrico Fagotti^1^, Andrea Pisent^1^, Conte Valeria^1^

##### ^1^Italian National Institute of Nuclear Physics INFN, Legnaro National Laboratories, Legnaro, Italy

A fast and accurate in-phantom monitoring of radiation quality is particularly challenging for BNCT, where the radiation field is composed by several components with widely different biological effectiveness. Microdosimetry is a valuable technique towards this aim, allowing to discriminate the photon, neutron and BNC dose components; moreover, it can provide also an estimation of the Relative Biological Effectiveness (RBE) of the radiation field, useful for intercomparisons between different sources. In order to study the potential of microdosimetry for beam quality monitoring in BNCT, microdosimetric measurements were performed at the small-scale accelerator-based thermal neutron source available at the Legnaro National Laboratories of INFN. The source is based on a 5-MeV, 3-µA proton beam hitting a beryllium target; the emerging fast neutron spectrum is moderated by means of a heavy-water and graphite beam shaping assembly. A Tissue-Equivalent Proportional Counter with interchangeable cathode walls was filled at different gas pressures in order to study the energy deposition pattern for different sensitive volume sizes, in the range 0.5 – 10 µm. Measurements were taken both with 100ppm boron doping of the cathode walls and without boron doping, and a proper subtraction procedure was applied on the measured spectra to determine the relative dose contributions due to photons, neutrons and BNC reaction products. The RBE-weighted biological dose due to each component was also calculated by means of a biological weighting function. Such a microdosimetric characterization could therefore be a valuable tool for beam quality monitoring in BNCT.

### O 121 - Towards an adequate description of the dose-response relationship in BNCT of brain tumours

#### Barbara Marcaccio^1^, Ian Postuma^2^, Setareh Fatemi^2^, Valerio Varcesi^1^, Cinzia Ferrari^2^, Laura Cansolino^2^, Marco Crepaldi^3^, Daniele Dondi^2^, Dhanalakshmi Vadivel^2^, Yi-Wei Chen^4^, Fong-In Chou^5^, Jinn-Jer Peir^5^, Chuan-Jen Wu^5^, Hui-Yu Tsai^6^, Jia-Cheng Lee^4^, Agustina Portu^7^, Sara González^7^, Silva Bortolussi^1^

##### ^1^University of Pavia, Physics, Pavia, Italy ^2^National Institute of Nuclear Physics INFN, Pavia Unit, Pavia, Italy ^3^University of Pavia, Department of Clinical and Surgical Sciences- Integrated unit of experimental surgery- advanced microsurgery and regenerative medicine, Pavia, Italy ^4^Taipei Veterans General Hospital, Department of Oncology, Taipei, Taiwan Province of China ^5^National Tsing Hua University, Nuclear Science and Technology Development Center, Hsinchu, Taiwan Province of China ^6^National Tsing Hua University, Institute of Nuclear Engineering and Science, Hsinchu, Taiwan Province of China ^7^National Atomic Energy Commission CNEA, Unit of Buenos Aires, Buenos Aires, Argentina

Boron Neutron Capture Therapy (BNCT) is a highly selective radiotherapy based on the capture reaction:. Boron is administered to patients via compounds able to enrich the tumor with higher concentrations compared to normal tissue. The neutron capture produces highly ionizing particles, releasing all their energy in a confined region (5-9 µm). Therefore, it is possible to kill the tumor cells while preserving the healthy tissues. The BNCT absorbed dose is due to a mixed field of radiation with different biological efficacy. The complexity of this field makes it difficult to predict the therapeutic effect. It is therefore necessary to translate the BNCT dose in units equivalent to conventional photon radiotherapy, for which the dose-effect relationship is known. Glioblastoma multiforme (GBM), a brain tumor, stands out for its malignancy, rapid progression, and resistance to conventional treatments. This work aim is to contribute to a better understanding of the relationship between the therapeutic effect and the dose delivered in BNCT to GBM, by optimizing existing models for calculating the photon isoeffective dose, a biophysical model which has proven suitable to express BNCT dose into photon-equivalent units. New in-vitro radiobiological data are presented and used as the input for in-patient photon isoeffective dose calculation. A treatment planning for a clinical case is described, using an accelerator-based neutron beam designed in Italy. Photon Isoeffective dose model is compared with the traditional RBE-weighted dose, typically used in BNCT, showing the impact of choosing a suitable model for the description of the dose distribution.

### O 122 - BNCT with the use of secondary neutrons? Monte Carlo Simulations and Cell Survival Experiments

#### Villads Lundsteen Jacobsen^1^, Johansen Jacob Graversen^1^, Brita Singers Sørensen^2^, Fynbo Hans Otto Uldall^3^, Mateusz Krzysztof Sitarz^1^, Anders Frederiksen^2^, Niels Bassler^4^

##### ^1^Aarhus University Hospital, Danish Centre for Particle Therapy, Aarhus, Denmark ^2^Aarhus University Hospital, Department of Experimental Clinical Oncology, Aarhus, Denmark ^3^Aarhus University, Physics and Astronomi, Aarhus, Denmark ^4^Aarhus University Hospital- Aarhus- Denmark, Danish Centre for Particle Therapy DCPT, Aarhus, Denmark

Proton boron capture and proton boron neutron capture therapy both assert that adding boron would generate additional high-LET particles and would lead to an increase RBe in proton beams. Here we show results from phantom simulations and in vitro clonogenic assay for a V79 cell line exposed to sodium borocaptate (BSH) with 10B and 11B contents, before irradiation. TOPAS MC simulations show the n+10B capture reaction produces a factor of 177±24 more high-LET particles than the proposed p+11B reaction for typical phantom and PTV size in radiotherapy. By varying the phantom size (fig.2) and proton field size it was found that the fluence of low energy neutrons needed for the 10B reaction can vary up to two orders of magnitude, also confirmed by SHIELD-HIT12A MC simulations. Previous studies might underreport the neutron capture reaction channel, due to non clinical relevant use of phantom and field size, and a problematic choice of physics packages for the MC simulation. Experimentally obtained cell survival curves show no indication of an increased RBE in the presence of BSH (fig.1). However, an apparent BSH toxicity affected the colony growth and plating efficiency. Chemical analysis showed that the procured BSH was contaminated with oxidation products despite being stored in a protective gas. A photon (6 MV linac) reference experiment also indicated BSH toxicity, but no radiosensitization. In conclusion, if boron increases RBE, it’s most likely to arrive from n+10B rather than p+11B reactions for clinically relevant phantom and field sizes, when using natural boron.

### O 123 - Enhanced cell killing effects by proton boron reaction: Experimental evidence and theoretical analyses

#### Dalong Pang^1^, William Parke^2^, Cara Frame^1^, Alfredo Venela^3^, Mira Jung^3^, Anatoly Dritshilo^1^

##### ^1^Georgetown University Hospital, Radiation Medicine, Washington DC, USA ^2^The George Washington University, Physics, Washington DC, USA ^3^Georgetown University Medical Center, Lombardi Comprehensive Cancer Center, Washington DC, USA

Proton radiotherapy (RT) delivers therapeutic radiation through charged proton particle interaction in the targeted tissues. The primary advantage of proton RT over photon RT is a heavy charged particle with well-defined, straight trajectory in human tissues with an energy loss characterized by the Bragg peak. Previous studies have shown the effectiveness of proton boron capture therapy, implicating potential dose enhancement due to proton Boron interaction along with some controversies. The controversies are mostly based on Monte Carlo simulation of the proton Boron reaction and the amount of energy generated in the process. Here, we report that pretreatment of cancer cells with Boron enhance cell killing and extend DNA damage and delay damage repair. Using two cancer cell lines (SQ-20B and MCF-7), we examined the efficacy of Boron in combination with proton by cell survival assays and the gH2AX foci formation. Approximately, a 15% increase in cell killing at the 10% survival level was observed for both cell lines, suggesting the mechanism of the high-LET alpha particle facilitated cell killing and complex DNA DSBs. We further performed theoretical analyses using a classical mechanistic model to support our hypothesis of alpha particle contribution in cell killing by way of proton-Boron interaction. Our data is consistent with a previously published report by other investigators, and we advance the thesis that Boron is a proton specific radiation sensitizer and can be used with little cytotoxicity in combination with proton therapy.

### O 124 - Neutron capture reactions to treat Alzheimer’s disease: Toxicity evaluation on nervous system cells

#### Valeria Pascali^1,2^, Saverio Altieri^1,2^, Carmen Villagrasa^3^, Yann Perrot^3^, Marine Herve^3^, Lovisa Lundholm^4^, Sergey Belikov^4^, Mostafa Karimi Roshan^4^, Nicoletta Protti^1,2^

##### ^1^University of Pavia, Physics Department, Pavia, Italy ^2^National Institute of Nuclear Physics - INFN, Pavia Unit, Pavia, Italy ^3^Institut De Radioprotection Et De Surete Nucleaire - IRSN, Ionizing Radiation Dosimetry Laboratory - LDRI, Paris, France ^4^The Wenner-Gren Institute - Stockholm University, Department of Molecular Biosciences, Stockholm, Sweden

One of the most widespread neurodegenerative disorder in the world is Alzheimer’s disease (AD). Although the numbers have reached 58 million by 2021, there is still no cure able to defeat it. NECTAR project proposes an innovative idea focused on modifying the AD progression: studying the effectiveness of neutron capture reactions on B-10 and Gd-157 in order to damage β-amyloid aggregates, one of the AD hallmarks. This should be made possible thanks to the combined action of the charged particles and the gamma produced by the named reactions. The former should be able to deposit their energy inside the Aβ aggregates, damaging them; the gamma, acting over a long range, should activate the glia cells, promoting an inflammatory responses that further cleanse the aggregates. NECTAR suggests an innovative treatment based on low dose and low dose-rates, involving a pan-irradiation of the brain, a highly radiosensitive organ. In this scenario, several studies must be carried out to ascertain the toxicity and biological effects induced by the treatment. In-vitro experiments are being performed on neurons and glia cells samples, evaluating the cellular and cytogenetic effects. Observed experimental results are correlated with dosimetric quantities estimated by Monte Carlo simulations. The talk aims to present the results of the Monte Carlo simulations performed with the Geant4 and Geant4-DNA code in the described scenario: they concern the estimation of micro- and macrodosimetric quantities in (i) a network of voxellized neurons and (ii) in glia cells whose voxellized geometry was obtained from confocal images.

### O 125 - Deep learning based uncertainty prediction of deformable image registration for contour propagation and dose accumulation in adaptive radiotherapy

#### Andreas Smolders^1,2^, Damien Charles Weber^1,3,4^, Tony Lomax^1,2^, Francesca Albertini^1^

##### ^1^Paul Scherrer Institute, Center for Proton Therapy, Villigen, Switzerland ^2^ETH Zurich, Department of Physics, Zurich, Switzerland ^3^Inselspital- Bern University Hospital, Department of Radiation Oncology, Bern, Switzerland ^4^University Hospital Zurich, Department of Radiation Oncology, Zurich, Switzerland

**Aim**: Deformable image registration (DIR) enables contour propagation and dose accumulation in adaptive therapy. However, clinical use of DIR is limited, as different algorithms generally yield different solutions. This work presents a deep-learning method predicting deformable vector field (DVF) uncertainty and its propagation into contour and dose accumulation uncertainty.

**Methods**: A 3D UNet was trained on 52 CT pairs to predict the variance of a given DVF, using the fixed and moving image and DVF as input. The training combined an unsupervised loss (https://doi.org/10.1007/978-3-031-11203-4_7), yielding high uncertainty where contrast is low and inversely, with a supervised loss (https://openreview.net/forum?id=6b60oHnnST4) yielding high uncertainty in regions with non-deformable anatomical transformations, e.g. nasal cavity filling. The resulting probabilistic DVF is sampled to warp contours and dose distributions, yielding contour and dose samples. The method was evaluated on the DIRLAB dataset, containing 10 patients with in- and exhale CTs with 300 landmarks. Additionally, for 5 lung cancer patients with 9 repeated manually contoured CTs, the planning contours were propagated with 50 DVF samples to each repeated CT, yielding voxel-wise probabilities to be inside a contour. This allows calculation of the expected calibration error (ECE, https://doi.org/10.48550/arXiv.1706.04599).

**Results**: The model can predict the average DIRLAB landmark uncertainty relatively accurately and predicts contour uncertainty with ECE=2.5%, i.e. the predicted voxel-wise probabilities differ on average 2.5% from the actual proportion inside the contour (Fig.1). The DVF uncertainty can be translated in contour and dose uncertainty in 20 seconds (Fig.2).

**Conclusion**: The method can predict DIR uncertainty and is efficiently usable in adaptive therapy.

### O 126 - Real-time expected DVH for carbon ion therapy

#### Cosimo Galeone^1,2,3^, Timo Steinsberger^1^, Lennart Volz^1^, Marco Donetti^4^, Felix Mas Milian^2,5,6^, Roberto Sacchi^2,6^, Anna Vignati^2,6^, Marco Durante^1,7^, Simona Giordanengo^6^, Christian Graeff^1,3^

##### ^1^GSI Helmholtzzentrum für Schwerionenforschung GmbH, Biophysics, Darmstadt, Germany ^2^University of Torino, Physics, Torino, Italy ^3^Technical University of Darmstadt, Department of Electrical Engineering and Information Technology, Darmstadt, Germany ^4^Fondazione CNAO, Medical physics, Pavia, Italy ^5^Universidade Estadual de Santa Cruz, Department of Exact and Technological Sciences, Ilheus, Brazil ^6^National Institute of Nuclear Physics, Division of Torino, Torino, Italy ^7^Technical University of Darmstadt, Condensed Matter Physics, Darmstadt, Germany

The estimation of dose errors is crucial for online adaptive particle therapy. Fast forward 4D dose calculation (4DFDC) can assess motion mitigation strategies during treatment. We propose a system to predict the final dosimetric outcome during delivery. The framework, based on INFN-RIDOS, calculates the planned and delivered 4D-doses parallel to delivery and updates the expected delivered dose, as: ExpectedFinalDose = FullPlanDose − ∑ PlanDose + ∑ DeliveredDose, summing over all spots delivered up to that point. PlanDose per spot uses planned particle numbers and beam positions, DeliveredDose those measured by nozzle detectors. The system connected to the research version of the CNAO Dose Delivery System at GSI via TCP/IP, receives measured spot properties (MU, beam position and motion phase) during delivery. After each motion phase, the dose distributions and projected DVH are updated on a GUI (Fig.1). After each slice, a γ-analysis shows differences between planned and expected dose. The algorithm was tested on a virtual XCAT 4DCT using previously recorded delivery data. To simulate a delivery error, the fluence of 5000 random spots was increased by 5%. The framework successfully achieved the 4DFDC of each spill faster than the spill pause, realizing online calculation. The planned dose was validated against a research TPS (TRiP98) (Fig.2), showing good agreement with γ-analysis passing rate (3mm/3%)>98%. We demonstrated the feasibility of a real-time dose calculation with clinically acceptable precision. Projected DVHs could be used to identify treatment failures during delivery and to trigger intervention if necessary.

### O 127 - First demonstration of robust online dose restoration evaluated with extensive robustness analysis for H&N IMPT

#### Michelle Oud^1,2^, Sebastiaan Breedveld^1^, Marta Giżyńska^2^, Steven Habraken^1,2^, Zoltán Perkó^3^, Ben Heijmen^1^, Mischa Hoogeman^1,2^

##### ^1^Erasmus MC Cancer Institute- University Medical Center Rotterdam, Department of Radiotherapy, Rotterdam, Netherlands ^2^HollandPTC, Department of Medical Physics & Informatics, Delft, Netherlands ^3^Delft University of Technology- Faculity of Applied Sciences, Department of Radiation Science and Technology, Delft, Netherlands

**Purpose/Objective**: While online adaptive IMPT accounts for inter-fraction anatomical changes, residual uncertainties remain. The impact of these uncertainties may be mitigated with robust online plans, but at the expense of calculation time and OAR sparing. We studied the dosimetric benefit of robust online adaptive dose restoration (OnA) compared to our clinical offline plan adaptation strategy (OffA) for H&N cancer, including an extensive robustness analysis.

**Material and Methods**: For 15 patients with 67 contoured repeat-CTs, doses on repeat-CTs for the OnA and OffA strategy were compared. For OnA, an initial treatment plan was generated using the planning CT (pCT) with 1 mm/3% setup/range robustness settings. Online adaptation on the repeat-CTs consisted of 1) restoring pencil-beam energies to planned water-equivalent-path-lengths, 2) adding pencil-beams, 3) robust reoptimization of pencil-beam intensities (1 mm/3%, Intel® Xeon® Gold 6248), 4) removal low-weighted pencil-beams. For OffA, 3 mm/3% robustness settings were used and offline replanning was applied when clinically requested. CTV dose was evaluated on every repeat-CT in the voxel-wise minimum and maximum scenario (1 mm/3%, 29 scenarios). Normal tissue complication probabilities (NTCPs) were calculated using nominal dose.

**Results**: CTV coverage improved with OnA compared to OffA, avoiding strong coverage losses (Fig.1). OnA significantly reduced the risk of xerostomia and dysphagia ≥ grade II with 3.7±1.8 and 3.2±3.2 percentage points (mean±SD), respectively (Fig.2). OnA intensity reoptimization times were on average 3.0 minutes.

**Conclusion**: Our robust online dose restoration approach results in significantly improved robust CTV coverage and reduced NTCPs compared to our current protocol. Reoptimization times may be feasible in clinical practice.

### O 128 - Online adaptive planning for proton therapy using the reference point method

#### Zihang Qiu^1,2^, Nicolas Depauw^2^, Bram Gorissen^3^, Thomas Madden^2^, Ali Ajdari^2^, Dick den Hertog^1^, Thomas Bortfeld^2^

##### ^1^University of Amsterdam, Department of Business Analytics, Amsterdam, Netherlands ^2^Massachusetts General Hospital, Department of Radiation Oncology, Boston, USA ^3^Broad Institute, Stanley Center for Psychiatric Research, Cambridge, USA

Online adaptive radiation therapy requires generating a quality daily treatment plan within minutes. A patient’s initial treatment plan can be used as a handy starting point to facilitate the planning process. In this study, we investigated the reference point method, a mathematical optimization model for online adaptive proton planning. The reference point method seeks a daily plan dosimetrically similar to the patient’s initial plan by mimicking the achieved objective values in the initial plan. Planning parameters of the initial plan, such as beam arrangement, planning objective, and constraint functions, are reused to establish the optimization problem for daily plan generation so that the need for human intervention is also minimized. The method was tested on a diverse cohort of 17 proton patients with various cancer sites, including breast, CNS, head and neck, prostate, and sarcoma cancer. Benchmarked against the offline adapted clinical replan of the patients, the reference point method-generated replans were scored ‘clearly better’ for 4 cases, ‘slightly better’ for 3 cases, ‘comparable’ for 6 cases, and ‘slightly worse’ for 4 cases by an experienced medical physicist in charge of clinical proton planning at our institution. Among the 4 plans ‘slightly worse’ than the clinical replan, 3 were deemed ‘clinically acceptable.’ The minimum, mean, and maximum time cost of plan generation were 29s, 216s, and 679s, using a linear optimizer on a high-performance computer from the Google Cloud platform.

### O 129 - Estimating segmentation accuracy in poorly segmented organs using deep-learning contour propagation uncertainty

#### Luciano Agustin Rivetti^1^, Andrej Studen^1,2^, Manju Sharma^3^, Jason Chan^3^, Robert Jeraj^1,4,5^

##### ^1^University of Ljubljana, Faculty of Mathematics and Physics, Ljubljana, Slovenia ^2^Jožef Stefan Institute, Experimental Particle Physics Department, Ljubljana, Slovenia ^3^University of California, Department of Radiation Oncology, San Francisco, USA ^4^University of Wisconsin, Department of Medical Phyisics, Madison, USA ^5^Jožef Stefan Institute, Reactor Physics, Ljubljana, Slovenia

In daily adaptive radiotherapy, fast and accurate acquisition of the daily structures is needed. Deformable image registration (DIR) speeds up this process by propagating the planning structures to the new daily anatomy. However, classical DIR doesn’t estimate the accuracy of the propagated structures leading to the time-consuming task of identifying and reviewing the structures which were not accurately segmented. In this work, deep learning was used to develop a fast and accurate DIR method for contour propagation that predicts the structure uncertainty and allows the estimation of the structure segmentation accuracy in poorly segmented organs in head and neck radiotherapy. An uncertainty score (UCS) obtained from the structure uncertainty was defined and validated to identify propagated structures that were prone to errors. The method’s performance was assessed by calculating the surface dice similarity coefficient (SDSC) between the propagated structures and their ground truth. In addition, the results were compared using a reference DIR method based on b-splines (Elastix). The results showed that the presented method has a median SDSC higher than Elastix for six of the ten structures analyzed (p>0.05). The UCS showed a strong Spearman anticorrelation (<-0.7) with the SDSC for poorly segmented structures, i.e., structures with a low mean and high standard deviation of SDSC. In conclusion, a fast and predictive Deep Learning method to propagate contours with competent accuracy with physician-delineated contours was developed. The method allows the estimation of the structure segmentation accuracy in poorly segmented organs without compromising the performance of the contour propagation.

### O 130 - Modelling small block aperture in an in-house developed GPU-accelerated Monte Carlo-based dose engine for pencil beam scanning proton therapy

#### Hongying Feng^1^, Jason Holmes^1^, Sujay Vora^1^, Martin Bues^1^, Joshua Stoker^1^, William Wong^1^, Terence Sio^1^, Jiajian Shen^1^, Wei Liu^1^

##### ^1^Mayo Clinic Arizona, Department of Radiation Oncology, Phoenix, USA

**Introduction**: Recently pencil-beam-scanning proton therapy (PBSPT)-based stereotactic radiosurgery has been proposed to treat brain cancer patients with shallow small targets, which requires a fast and accurate Monte Carlo (MC)-based proton dose engine for treatment planning. However, the conventional fast MC codes either do not support block aperture or are not fast enough for clinical use.

**Methods**: We modeled small block apertures in our in-house developed GPU-accelerated MC-based proton dose engine, VPMC. To validate the block aperture module, VPMC was first validated by MCsquare in nine water phantom simulations with 3cm thick brass apertures: four were with apertures of 1, 2, 3, and 4cm openings without range shifter, while the other five were with apertures of 1, 2, 3, 4, and 5cm openings with a range shifter of 45mm water equivalent thickness. Then, VPMC was benchmarked with MCsquare and RayStation MC for 10 patients with small targets (average volume 8.4 cc). Finally, 4 typical patients were selected for robust optimization with block apertures using VPMC.

**Results**: In the water phantom, 3D gamma passing rate (2%/2mm/10%) between VPMC and MCsquare was 99.690.22% (Table 1). In the patient geometry, 3D gamma passing rates (3%/2mm/10%) between VPMC/MCsquare and RayStation MC were 97.792.21%/97.781.97%, respectively. Meanwhile, the calculation time was drastically decreased from 112.45114.08 seconds (MCsquare) to 8.206.42 seconds (VPMC) (Table 2) with the same statistical uncertainty. The mean calculation time for 13 influence matrices in robust optimization by VPMC was 77.9 seconds.

**Conclusions**: VPMC has been successfully enhanced to model block apertures for PBSPT.

### O 131 - MonteRay: A fast Monte Carlo dose calculation engine for carbon ions and more

#### Peter Lysakovski^1,2^, Benedikt Kopp^1^, Stewart Mein^1,3^, Thomas Tessonnier^1^, Alfredo Ferrari^1^, Thomas Haberer^1^, Jürgen Debus^1,4,5^, Andrea Mairani^1,6^

##### ^1^Heidelberg University Hospital, Heidelberg Ion-Beam Therapy Center- Department of Radiation Oncology, Heidelberg, Germany ^2^Heidelberg University, Faculty of Physics and Astronomy, Heidelberg, Germany ^3^Perelman School of Medicine of the University of Pennsylvania, Department of Radiation Oncology, Philadelphia, USA ^4^National Centre for Radiation Oncology NCRO, Division of Molecular and Translational Radiation Oncology- Heidelberg Institute of Radiation Oncology HIRO, Heidelberg, Germany ^5^German Cancer Research Center DKFZ, Translational Radiation Oncology- German Cancer Consortiuum DKTK- National Center for Tumor Diseases NCT, Heidelberg, Germany ^6^National Centre of Oncological Hadrontherapy CNAO, Medical Physics, Pavia, Italy

At the Heidelberg Ion-Beam Therapy Center (HIT), protons, helium ions and carbon ions are used for patient treatment. Currently, treatment planning for carbon and helium ions is performed using analytical dose calculation algorithms. However, the use of Monte Carlo (MC) engines for independent dose calculation or as new engine standard is envisioned to improve dose accuracy prediction in challenging scenarios. Classical MC engines such as FLUKA are too slow for clinical practice. Instead, fast algorithms must be developed to accelerate simulations without sacrificing accuracy. Recently, the fast MC engine MonteRay was presented for protons and helium ions, achieving good accuracy when compared against measurements (e.g. SOBPs) and FLUKA simulations. In this work, the extension of MonteRay to carbon ions is presented. Figure 1 shows measured and MonteRay simulated carbon SOBPs at different depths in water. Excellent agreement of depth-dose curves was found with average relative errors in the high dose region of 1.0%, 0.6% and 0.8%, for the three depths respectively. To benchmark heterogenous scenarios, comparison against measured 2D dose-distributions behind an anthropomorphic phantom (Figure 2 a, b) were carried out. Agreements of 99.1% in terms of 3%/3mm local gamma passing rate were found (Figure 2 c). Comparing runtimes against those of FLUKA, speedups of 20-40 were observed. With the inclusion of carbon ions, MonteRay is now able to simulate all clinically used particles at HIT. In the future, introduction of MonteRay into the clinical workflow is planned as a secondary dose engine to verify TPS computed doses.

### O 132 - Minimization of number of energy jumps for proton arc therapy

#### Gezhi Zhang^1^, Yong Long^1^, Yuting Lin^2^, Ronald Chen^2^, Hao Gao^2^

##### ^1^Shanghai Jiao Tong University, University of Michigan-Shanghai Jiao Tong University Joint Institute, Shanghai, China ^2^University of Kansas Medical Center, Radiation Oncology, Kansas City, USA

**Purpose**: The optimization of energy layer distributions is crucial to proton ARC therapy: on one hand, a sufficient number of energy layers is needed to ensure the plan quality; on the other hand, an excess number of energy jumps can substantially slow down the treatment delivery. This work will develop a new treatment plan optimization method with direct minimization of number of energy jumps (NEJ), which will be shown to outperform state-of-the-art methods in both plan quality and delivery efficiency.

**Methods**: The proposed method jointly optimizes the plan quality and minimizes the NEJ. To minimize NEJ, (1) the proton spots *x* is summed per energy layer to form the energy vector *y*; (2) *y* is binarized via sigmoid transform into *y*_1_; (3) *y*_1_ is multiplied with a predefined energy order vector via dot product into *y*_2_; (4) *y*_2_ is filtered through the finite-differencing kernel into *y*_3_ in order to identify NEJ; (5) only the NEJ of *y*_3_ is penalized, while *x* is optimized for plan quality. The solution algorithm to this new method is based on iterative convex relaxation.

**Results**: The new method is validated in comparison with state-of-the-art methods called energy sequencing (ES) method and energy matrix (EM) method. In terms of delivery efficiency, the new method had fewer NEJ, less energy switching time, and generally less total delivery time. In terms of plan quality, the new method had smaller optimization objective values, lower normal tissue dose, and generally better target coverage.

### O 133 - A fast analytical dose calculation approach for MRI-guided proton therapy

#### Alisha Duetschler^1,2^, Carla Winterhalter^1^, Sairos Safai^1^, Damien C. Weber^1,3,4^, Antony J. Lomax^1,2^, Ye Zhang^1^

##### ^1^Paul Scherrer Institute, Center for Proton Therapy, Villigen PSI, Switzerland ^2^ETH Zurich, Department of Physics, Zurich, Switzerland ^3^University Hospital of Zurich, Department of Radiation Oncology, Zurich, Switzerland ^4^Inselspital - Bern University Hospital - University of Bern, Department of Radiation Oncology, Bern, Switzerland

**Purpose**: MRI-guided proton therapy, which could help exploit the potential of proton therapy, requires a fast dose calculation approach, especially for online plan adaptations. Monte-Carlo (MC), the gold standard for calculating dose in magnetic fields, is however time-consuming. We have therefore developed a fast GPU-based modification of the raycasting analytical calculation which accounts for beam deflections due to the magnetic field of a MR scanner.

**Methods**: From TOPAS-MC simulations of proton beams (70-229 MeV) in water with orthogonal magnetic fields (0.5/1.5 T) central beam trajectories were extracted. From these, lookup tables (LUT) of incremental rotation angles as a function of water-equivalent pathlength are generated. The LUTs are then used to reconstruct the beam trajectory in heterogeneous media using a modified raycasting approach (fig1). This was benchmarked in water and for different materials and calculations showing the effect of magnetic fields on the dose for an example clinical case are presented.

**Results**: Good agreement between MC and analytical dose distributions was achieved with differences in range R_80%_ < 0.7 mm and lateral shifts < 0.9 mm for all energies and magnetic field strengths in water (fig2a). Dose distributions and DVHs, calculated analytically and with MC in patient geometry, are shown in fig2b. 3%/3mm-gamma agreements above 99.6% were achieved for 0-1.5 T with calculation times reduced from hours/days using MC to < 1 minute.

**Conclusion**: The analytical calculation approach can simulate proton dose distributions in magnetic fields with high speed and accuracy. Next, we will incorporate this into an appropriate optimization strategy.

### O 134 - A novel dose calculation patient-specific method in the blood of major vessels in pencil beam scanning (PBS) proton therapy systems

#### Marina García Cardosa^1^, Pedro Borja Aguilar^2^, Rosa Meiriño^3^, Juan Diego Azcona^2^, Felipe Ángel Calvo^3^, Javier Burguete^1^

##### ^1^University of Navarra, Physics and Applied Mathematics, Pamplona, Spain ^2^Clínica Universidad de Navarra, Hospital Radiophysics, Madrid, Spain ^3^Clínica Universidad de Navarra, Radiotherapy Oncology, Madrid, Spain

**Purpose**: To develop a novel patient-specific method to quantify the dose delivered to circulating blood in major vessels.

**Materials and Method**: Using patient data including vasculature and temporal structure of the beam delivery, we quantify the dose received by the circulating blood. An expert determines the vessel contours using the amplitude signal from a Phase Contrast MRI sequence. The temporal structure is obtained using Monte Carlo in RayStation, a dose map is calculated for every energy level and the start and end time for this field is determined from the patient treatment taken from the PBS console. The blood volume is divided into 5.5·10^6 particles of 1mm^3 that are tracked in the vasculature and are propagated with a measured velocity using a Phase Contrast MRI sequence. The acquired velocity field is a temporal average of the pulsating flow rate and the displacement of the vessels. A divergence cleaning method is applied since averaged flows are not divergence free.

**Results**: From the dose received by each one of the fluid particles, a statistic analysis can be performed: average, dose, Dose-Volume Histogram (DVH). Preliminary results show that in a patient 0.08% of the blood volume received more than 0.1Gy, being the highest dose 0.16Gy for 0.0006% of the blood volume. We can accomplish a robustness study analysing displacement of the vessels, inaccurate vessel diameter determination, and cardiac output uncertainties.

**Conclusions**: A personalised patient-specific approach to determine the dose received in vessels and blood during proton therapy PBS in clinical practice is feasible.

### O 135 - Proton and carbon ion therapy for intracranial meningioma after previous brain radiotherapy

#### Giulia Riva^1^, Lucia Pia Ciccone^1^, Stefania Russo^1^, Alberto Iannalfi^1^, Ester Orlandi^1^

##### ^1^National Center of Oncological Hadrontherapy CNAO, Clinical Department, Pavia, Italy

**Purpose**: Meningiomas show high rates of control after radiotherapy(RT). Progression after RT can occur with few options for salvage treatment. Re-RT is often difficult due to the limited radiation tolerance of the surrounding organs at risk(OARs). The aim is to evaluate the feasibility and safety of re-RT with proton(PT) and carbon ion(CIRT) for meningiomas.

**Materials and Methods**: We reviewed data from patients who received re-RT with PT or CIRT for meningioma from 2013 to 2021. Local control(LC), Disease-free-survival(DFS) and Progression-free-survival(PFS) were estimated using the Kaplan-Meier; p values were calculated using Fisher’s test. Toxicity was reported according to CTCAEv5.0.

**Results**: Sixty-nine patients treated with PT (n=48, 70%) or CIRT (n=21, 30%) for recurrent/RT-induced meningioma were evaluated. Table1 shows patients’ characteristics while, in Table2, data of particle-therapy are reported. Median follow-up was 20 months(range, 3-99). 2y-LC and 4y-LC were 78.8% and 55.0%, There was no significant difference between recurrent and RT-induced meningiomas in terms of LC(p=0.71), DFS(p=0.71) and PFS(p=0.49). Acute grade(G)3 toxicity was reported in 2(3%) cases and late G3 toxicity in 2(3%) patients(optic-nerve disorder and new-onset of seizure without radionecrosis). Brain necrosis occurred in 19(28%) cases: 7, 11 and 1 patients developed G1, G2 and G3 radionecrosis. No acute/late G4-G5 toxicities were observed.

**Conclusions**: PT/CIRT re-RT is a feasible and safe treatment in recurrent/RT-induced meningiomas. PT/CIRT can improve dose-conformity and reduce radiation-dose to OARs potentially leading to clinical benefits in terms of toxicity. Moreover, CIRT provides an increased RBE, which could be beneficial on LC considering radiation-resistance of recurrent/RT-induced meningiomas.

### O 136 - Clinical and dosimetric outcomes of a phase II study of proton radiation therapy for lower grade gliomas

#### Jason Slater^1,2^, Nora Horick^3^, Lisa Nachtigall^4^, Michael Parsons^5^, Giuliana Zarrella^6^, Nicholas Tritos^4^, Jorg Dietrich^5^, Jennifer Pursley^2^, Barbara Fullerton^2^, Irene Wang^2^, Kevin Oh^2^, Jay Loeffler^2^, Beow Yeap^3^, Helen Shih^2^

##### ^1^Loma Linda University School of Medicine, Radiation Medicine, Loma Linda, USA ^2^Massachusetts General Hospital, Radiation Oncology, Boston, USA ^3^Massachusetts General Hospital, Biostatistics, Boston, USA ^4^Massachusetts General Hospital, Neuroendocrine & Pituitary Tumor Clinical Center, Boston, USA ^5^Massachusetts General Hospital, Stephen E. & Catherine Pappas Center for Neuro-Oncology, Boston, USA ^6^Massachusetts General Hospital, Neuropsychology, Boston, USA

**Purpose**: To report preliminary outcomes and dosimetry of patients with lower grade gliomas (LGG) treated with proton therapy.

**Methods**: Patients with grade 2 or IDH mutant grade 3 glioma and indications for radiation therapy were enrolled in a prospective single arm trial of proton therapy (PRT), receiving a dose of 54 Gy(RBE) or 59.4 Gy(RBE), respectively. Summary of dosimetry and of baseline and regular post-treatment evaluations of neuroendocrine function, neurocognitive functions (NCF), quality of life (QOL), and progression-free survival (PFS) were performed.

**Results**: Sixty patients received PRT with a median follow up of 4.1 years. Table 1 shows patient demographics. The mean whole brain D50 was 1.64 Gy(RBE) [range: 0.0 – 21.4 Gy(RBE)], and the mean pituitary D50 was 0 Gy(RBE) [range: 0 – 49.3 Gy(RBE)]. The mean D50 to ipsilateral temporal lobe was 21.7 Gy(RBE) [range: 0 – 61.3 Gy(RBE)], and the mean D50 to the contralateral temporal lobe was 0.9 Gy(RBE) [range: 0-52.9 Gy(RBE)]. New endocrine dysfunction was detected in 3 patients (5%), details in Table 2. There was one grade 4 case of brain necrosis (2%) but no grade 3 toxicities. Three-year PFS, Neurocognitive function and QOL data will be presented.

**Conclusions**: Lower grade glioma patients receiving PRT have a similarly high 3-year PFS as previous reports on photon therapy. PRT shows significant dosimetric sparing of whole brain, pituitary and non-involved temporal lobes. These lower doses may explain the positive neuroendocrine, neurocognitive and QOL results.

### O 137 - Effectiveness and safety of proton therapy for brain meningiomas: A monocentric experience

#### Lucia Pia Ciccone^1^, Giulia Riva^1^, Marco Rotondi^1^, Stefania Russo^1^, Alberto Iannalfi^1^, Ester Orlandi^1^

##### ^1^National Center for Oncological Hadrontherapy CNAO, Clinical Department, Pavia, Italy

**Purpose**: Brain meningiomas (BM) are typically located near critical organs, BM should be treated with proton therapy (PT, Fig.1). Here we present a monocentric report of BM treated with PT.

**Materials and Methods**: We reviewed data from 168 BM patients (pts) treated with PT between 2014 and 2021.LC and PFS are evaluated using Kaplan-Meier. Radiation-induced AEs (RIAEs) were reported according to CTCAE v5.0. Late RIAE>G2 were analysed using Fisher’s test.

**Results**: Median pts age was 55 years (15-86). One hundred-eight received PT in adjuvant setting or for relapse: 74 WHO I and 29 WHO II-III. Surgery/biopsy was performed in 60 pts (36%). PT median total-dose was 55.8GyRBE (50,4–66) and median dose/fraction was 1.8GyRBE (1.8–3.6). Median-ccGTV and Median-ccCTV were 15.6cc (0–324) and 30.5cc (3.1–354). Median follow-up was 40 months (6-99). Ten pts had local-relapse after a median time of 40 months (13-50), one local-recurrence occurred in WHO I case. 5y-Local Control (LC) and 5y-Progression-free-survival (PFS) were 98.1% and 90.7% in WHO I and no-surgery group. WHO II-III group 5y-LC and 5y-PFS were 53.0% and 42.3%, respectively. Two acute RIAEs=G3 were reported (radiodermatitis, edema cerebral). Late RIAEs were reported in Table1. All-grade brain radionecrosis was reported in 25 pts (15%); 2 and 3 of them experienced G2 and G3 brain toxicity, respectively. No G4-G5 RIAEs were highlighted.

**Conclusion**: PT can be considered a valuable treatment for BM due to the high rate of LC with minimal side effects. PT seems to be an effective and safe treatment also when it is given in progressive and pre-treated diseases.

### O 138 - The initial clinical experience with an interactive, multi-language, app-based neurocognitive evaluation program for proton therapy on CNS malignancies

#### Tugce Kutuk^1^, Natalie Mesa^1^, Yazmin Odia^2^, Matthew D. Hall^1^, Haley Appel^1^, Miguel Ramirez^1^, Alonso La Rosa^1^, Manmeet Ahluwalia^3^, Minesh P. Mehta^1^, Rupesh Kotecha^1^

##### ^1^Miami Cancer Institute- Baptist Health South Florida, Radiation Oncology, Miami, USA ^2^Miami Cancer Institute- Baptist Health South Florida, Neuro-oncology, Miami, USA ^3^Miami Cancer Institute- Baptist Health South Florida, Medical Oncology, Miami, USA

**Purpose**: Proton therapy (PT) for patients with CNS malignancies is often recommended to reduce integral doses to key substructures associated with neurocognitive function (NCF). However, NCF is often not evaluated in clinical practice and key proton-specific dosimetric parameters associated with NCF have yet to be adequately demonstrated. The objective of this prospective observational study was to assess NCF in patients treated with PT using a comprehensive app-based neurocognitive assessment.

**Methods**: Patients undergoing PT from December 2021 to December 2022 were enrolled into a prospective observational study (NCT05504681) and completed feedback surveys. The neurocognitive assessment consisted of learning and memory (Hopkins verbal learning test-revised [HVLT-R]), attention and processing speed (Digit symbols modalities test [SDMT]), verbal fluency (Controlled oral word association test [COWAT]) and executive function (Trail making test [TMT]).

**Results**: 21 patients were enrolled during the study period; 57.1% had a high school level of education, and 33.3% performed testing in Spanish. At 3 months, the mean relative decline was 30% for HVLT-R-immediate recall, 34.4% for HVLT-R-delayed recall, 3.4% for HVLT-R-recognition, and 23.1% for TMT-A. However, we observed a mean relative improvement of 16.5% for COWAT, 15.3% for SDMT, and 19.7% for TMT-B. The majority of the patients found the system easy to use (90%), relevant to their care (60%) and would recommend it to other patients (90%).

**Conclusion**: The initial clinical experience with this interactive, multi-language, app-based neurocognitive evaluation program shows broad patient acceptance and a high level of compliance; further dosimetric correlatives to outcomes will be evaluated with future analyses.

### O 139 - Proton therapy for craniospinal irradiation: A consensus statement from the PTCOG Skull Base/CNS/Sarcoma subcommittee on simulation, treatment planning and delivery

#### Arpit Chhabra^1^, Michael Soike^2^, Rex Carden^2^, Markus Stock^3^, Mark McDonald^4^, James W. Snider^5^, Adam Kole^2^, Robert Press^6^, Jun Zhou^4^, Mark Mishra^7^, Bree Eaton^4^, Pouya Sabouri^8^, Francesca Albertini^9^, Chengyu Shi^10^, Haibo Lin^1^, Sina Mossahebi^7^, Jake Wang^11^, Rovel Colaco^12^, Heng Li^13^, Charles B. Simone^1^

##### ^1^New York Proton Center, Department of Radiation Oncology, New York, USA ^2^University of Alabama, Department of Radiation Oncology, Birmingham, USA ^3^EBG MedAustron, Department of Radiation Oncology, Wiener Neustadt, Austria ^4^Emory University, Department of Radiation Oncology, Atlanta, USA ^5^South Florida Proton Therapy Institute, Department of Radiation Oncology, Delray Beach, USA ^6^Miami Cancer Institute - Baptist Health, Department of Radiation Oncology, Miami, USA ^7^University of Maryland, Department of Radiation Oncology, Baltimore, USA ^8^University of Arkansas, Department of Radiation Oncology, Little Rock, USA ^9^Paul Scherrer Institute, Department of Radiation Oncology, Villigen, Switzerland ^10^City of Hope, Department of Radiation Oncology, Irvine, USA ^11^Willis-Knighton Health System, Department of Radiation Oncology, Shreveport, USA ^12^The Christie NHS Foundation Trust, Department of Radiation Oncology, Manchester, United Kingdom ^13^Johns Hopkins University, Department of Radiation Oncology, Baltimore, USA

**Purpose/Objectives**: Proton craniospinal irradiation (CSI) offers established dosimetric and clinical benefits compared to photon therapy. These guidelines summarize several elements for optimal deliver of Proton CSI.

**Materials and Methods**: The PTCOG Skull Base/CNS/Sarcoma Subcommittee consisting of radiation oncologists and medical physicists with specific expertise in CSI developed recommendations around delivery of Proton CSI.

**Results**:

- ***Simulation***: Simulation should be performed supine with a long or short mask around a thermoplastic pillow on a base of skull board. Arms should be at the side or across the abdomen. A custom device should be employed to stabilize the lumbar spine and pelvis to prevent rotational errors. Institutional variability exists on the extent of the custom mold, either throughout pelvis or from knees down with the patient flat on the table.
- ***Target Volume Delineation***: The entire CSI axis should be contoured with attention to covering hypoglossal canal, jugular foramen, internal auditory canal, foramen ovale, rotundum, superior orbital fissure, and optic canal. Spinal and sacral nerve roots should be contoured as they exit the spinal canal and sacrum.
- ***Treatment Planning Methodology***: Field arrangement should employ two posterior oblique fields for cranial portion and single or multiple posterior field(s) for spinal portion. Fields are created using the gradient-optimized methods by producing complementary dose gradients at the beam edges in the overlapping region (Figure 1). Robust optimization should be used to create robust gradient match to ensure target coverage and OAR sparing under specified setup and range uncertainties.

**Conclusions**: These recommendations are intended to enable centers to deliver proton CSI safely and effectively.

### O 140 - Adaptation of stochastic microdosimetric kinetic model to hypoxia for hypo-fractionated multi-ion therapy treatment planning

#### Taku Inaniwa^1^, Nobuyuki Kanematsu^2^, Makoto Shinoto^3^, Masashi Koto^3^, Shigeru Yamada^3^

##### ^1^Institute for Quantum Medical Science- QST, Department of Accelerator and Medical Physics, Chiba, Japan ^2^Institute for Quantum Medical Science- QST, Department of Accelerator and Medical Physics, Chib, Japan ^3^QST, QST Hospital, Chiba, Japan

**Purpose**: For hypo-fractionated multi-ion therapy (HFMIT), the stochastic microdosimetric kinetic (SMK) model had been developed to estimate the biological effectiveness of radiation beams with wide linear energy transfer (LET) and dose ranges. The HFMIT will be applied to radioresistant tumors with oxygen-deficient regions. The response of cells to radiation is strongly dependent on the oxygen condition in addition to radiation type, LET and absorbed dose. This study presents an adaptation of the SMK model to account for oxygen-pressure dependent cell responses, and develops the oxygen-effect-incorporated stochastic microdosimetric kinetic (OSMK) model.

**Material and Methods**: In the model, following assumptions were made: the numbers of radiation-induced sublethal lesions (double-strand breaks) are reduced due to lack of oxygen, and the numbers of oxygen-mediated lesions are reduced for radiation with high LET. The model parameters were determined by fitting survival data under aerobic and anoxic conditions for human salivary gland tumor cells and V79 cells exposed to helium-, carbon-, and neon-ion beams over the LET range of 18.5-654.0 keV/μm.

**Results and Discussion**: The OSMK model provided good agreement with the experimental survival data of the cells with determination coefficients > 0.9. In terms of oxygen enhancement ratio, the OSMK model reproduced the experimental data behavior, including slight dependence on particle type at the same LET. The OSMK model was then implemented into the in-house treatment planning software for the HFMIT to validate its applicability in clinical practice.

**Conclusion**: The OSMK model offered the accuracy and usability required for hypoxia-based biological optimization in HFMIT treatment planning.

### O 141 - RBE modeling in view of the first carbon-proton therapy center in America: The MCF MKM and the AMDM

#### Alessio Parisi^1^, Chris J. Beltran^1^, Hermann Fuchs^2^, Keith M. Furutani^1^

##### ^1^Mayo Clinic, Radiation Oncology, Jacksonville, USA ^2^Medical University Vienna, Radiation Oncology, Vienna, Austria

Mayo Clinic Florida (MCF) is building the first-in-America carbon-proton therapy center in Jacksonville. Currently, two approaches are mostly used for RBE modeling in clinic: the local effect model (LEM) and the microdosimetric kinetic model (MKM). Several versions of both models exist, with differences in their formalism and results. Firstly, our novel implementation of the MKM (MCF MKM) is presented in detail. This includes a systematic benchmark of the MCF MKM predictions against corresponding *in vitro* clonogenic survival data for several human and rodent cell lines (ions from ^1^H to ^238^U) and *in silico* results of other models (different MKM versions, LEM IV, MEDRAS, and LET-based proton RBE models) (Figure 1). The novel MCF MKM strategies to a *priori* determine the cell-specific model parameters (i.e., the size of the subnuclear domains) are also discussed. Secondly, it is currently unpractical to compute and store microdosimetric distributions in real clinical scenarios (large amount of data). This limits retrospective RBE recalculations with different models and imposes the use of precalculated cell- and model- specific RBE tables. We present a new methodology (abridged microdosimetric distribution methodology, AMDM) to compute and store a limited amount of data in each voxel. These cell- and model- independent quantities are used in a second step to compute the RBE for any arbitrary cell line with any microdosimetric model. When compared to full spectral calculations, the AMDM-based RBE showed a max deviation < ∼0.6% (Figure 2). The approach was successfully implemented in PHITS and GEANT4 and the results compared.

### O 142 - Proposing a clinical model for RBE based on proton track-end counts

#### Nicholas Henthorn^1^, Gardner Lydia^2^, Aitkenhead Adam^3^, Edward Smith^1^, Michael Merchant^1^, Ranald Mackay^3^, Karen Kirkby^1^, Pankaj Chaudhary^2^, Stephen McMahon^2^, Tracy Underwood^1^

##### ^1^University of Manchester, Division of Cancer Sciences, Manchester, United Kingdom ^2^Queen’s University Belfast, Patrick G Johnston Centre for Cancer Research, Belfast, United Kingdom ^3^The Christie NHS Foundation Trust, Christie Medical Physics and Engineering, Manchester, United Kingdom

Clinical application of LET optimisation remains contentious for proton therapy, in part due to challenges associated with the definition and calculation of LET and its exact relationship with RBE. This has raised interest in other metrics with favourable properties for biological optimisation, such as the number of proton track-ends (TE) in a voxel. We develop an ‘effective dose concept’ to translate between the total TE count per unit mass in a voxel and an RBE (Equation 1). E_TE_ represents an additional pseudo energy deposition per TE in a voxel (TE_i_) relative to the physical energy deposition (E_Phys,i_). RBE = 1.1 + (E_TE_ × TE_i_)/E_Phys,i_ [1] Dose, TE and LET_d_ distributions were simulated using MC models for a series of water phantoms, *in vitro* studies (Figure 1), and patient plans. We evaluated the correlation between TE and regions of elevated biological effectiveness in comparison to LET_d_-based models for an example paediatric ependymoma case (Figure 2). TEs were found to correlate with biological effects in *in vitro* experiments with an accuracy comparable to LET_d_. In patient simulations, our TE model identified the same biological hotspots predicted by LET_d_ based models of RBE. These results suggest that an RBE model based on proton TE counts may match information provided by LET_d_-based models, whilst offering superior statistical properties.

### O 143 - Effect of dose nonuniformity on cell killing effect in carbon ion radiotherapy

#### Misato Umemura^1^, Toshiro Tsubouchi^2^, Kazumasa Minami^1^, Naoto Saruwatari^1^, Masashi Yagi^3^, Noriaki Hamatani^2^, Masaaki Takashina^2^, Kazuhiko Ogawa^4^, Tatsuaki Kanai^2^, Masahiko Koizumi^1^

##### ^1^Osaka University Graduate School of Medicine, Medical Physics and Engineering, Osaka, Japan ^2^Department of Medical Physics, Osaka Heavy Ion Therapy Center, Osaka, Japan ^3^Osaka University Graduate School of M edicine, Department of Carbon Ion Radiotherapy, Osaka, Japan ^4^Osaka University Graduate School of Medici ne, Department of Radiation Oncology, Osaka, Japan

**Introduction**: In radiation therapy, uniform dose distributions in planning target volume are recommended. However, in photon beam therapy, spatially nonuniform dose distributions are reported to induce anti-tumor effect while sparing organs at risks. In carbon ion therapy, the effect of nonuniform dose distributions is little known. In the present research, we focused on the investigation of the biological effect caused by nonuniform dose distributions using carbon ions.

**Material and Methods**: The biological effect of nonuniform irradiation fields were investigated with HSGc-C5 and Nuli-1. First, we employed two patterns of nonuniform irradiation fields and conducted colony formation assay to obtain the survival fractions (S.F.). Based on alfa and beta parameters in the LQ model derived from the S.F. obtained by uniform dose exposure, we simulated the S.F. of nonuniform irradiation and compared to experimental ones. Second, we conducted immunofluorescent staining after nonuniform dose exposure to evaluate DNA damages and the ratio of cell apoptosis.

**Results and Conclusion**: The S.F. of Nuli-1 are higher than HSGc-C5 in the nonuniform dose distribution, although opposite results were confirmed in the uniform one from Fig1. These results suggest that nonuniform dose distributions with carbon ions support to spare normal tissues. We are analyzing results of immunofluorescent staining to verify this mechanism and will give our presentation at the meeting.

### O 144 - Dosimetric variability in the Bragg peak for radiobiological study

#### Mohammad Rezaee^1^, Lingshu Yin^2^, John Wong^1^

##### ^1^Johns Hopkins University, Radiation Oncology and Molecular Radiation Sciences, Baltimore- MD, USA ^2^Johns Hopkins University- Proton Therapy Center, Radiation Oncology ad Molecular Radiation Sciences, Washington- DC, USA

In this study, we investigate the feasibility of expanding the pristine Bragg peak in a low-density medium to alleviate the uncertainty of RBE measurements associated with the steep dosimetric gradient. A water medium was irradiated with a 1cm × 1cm, 142.4 MeV proton beam in a TOPAS Monte-Carlo simulation. In the Bragg peak region, 14 cm downstream, energy deposition events in 1 mm thick water plates to mimic cell culture samples were scored every 2 mm in water and every 10 mm in a hypothetical medium of 5% water density, denoted as LD-medium. In water, the peak dose was 59 Gy; the span of 80% – 80% dose maximum was 4 mm; and a shift of 0.2 mm in the range resulted in a maximum difference of 6 Gy. Correspondingly in the LD-medium, these values were 49 Gy; 76 mm; and < 3 Gy, respectively. In the descending region of the Bragg peak, dose-weighted average LET increased to 14 keV/mm in water, larger than 10 keV/mm at the same water equivalent depth in the LD-medium with similar quantile variation of ±10%. Beyond this depth, the LET distribution was highly variable in water, but changed modestly for an additional water equivalent 8 mm in the LD-medium. Our results indicate that a LD-medium can alleviate the positional uncertainty for physical and biological measurements in the Bragg peak region of a particle beam. More importantly, the results highlight the confounding complexity of LET variations whose impact on radiobiological response needs to be addressed.

### O 145 - Utilizing piecewise linear DVH based score functions to fully automate FLASH/IMPT dose optimization for objective treatment modality comparison

#### Anthony Magliari^1^, Pierre Lansonneur^2^, Esa Kuusela^3^, Jessica Perez^4^, Miriam Krieger^5^, Lesley Rosa^1^, Wendy Smith^1^, Adam Harrington^6^, Karl Bush^7^, Michael Folkerts^7^

##### ^1^Varian, Medical Affairs, Palo Alto, USA ^2^Varian, Research, Lyon, France ^3^Varian, Research, Helsinki, Finland ^4^Varian, Flash, Baden, Switzerland ^5^Varian, Research, Halle, Germany ^6^Varian, Flash, Palo Alto, USA ^7^Varian, Research, Palo Alto, USA

**Background**. Common dose optimizers use several individual DVH points organized by the user interactively (or by other automated means) as input. Second order derivatives convert input into [KE1] [MA2] quadratic cost functions which the system iterates to reduce. Since 2011, dosimetric scorecards have been created to precisely capture a specific clinical intent^1^. Scorecard uses include: scoring treatment planning competitions; quantifying dosimetric quality improvements of various techniques, energies, collimation devices, modalities and; tuning the inputs to common automated [FM3] [MA4] [MA5] quadratic DVH point optimizers (ie: RapidPlan and Ethos)^2^.

**Aim**. A novel fully-automated dosimetric treatment plan optimization process which creates plans directly from the precise expressed clinical intent (in scorecard form) thereby eliminating any reliance on historical treatment plans or variability in quality introduced by subjective intermediate-user generated priorities. [FM6] [MA7]

**Methods**. A single-fraction 24Gy SBRT Spine scorecard was the only input to the proton spot-weight optimizer for all plans on a single case. Single-energy-layer (250MeV) beam plans with five and ten fields were compared to traditional multi-energy layer (ML-IMPT) beam plans with two and three fields. Additional optimization(s) increasing priority on a minimum MU constraint enabled ultra-high dose rates, verified by PBS dose rate calculation^3^.

**Results**. All 250MeV plans outperformed ML-IMPT. Two field ML-IMPT and five field 250MeV plans could not achieve a positive score for all metrics (failing). Higher minimum MU priority led to decreasing plan quality (score).

**Conclusion**. With novel automated scorecard-based optimization, we objectively compared two different treatment modalities and discovered that a FLASH-RT plan out-scored a traditional IMPT plan.

### O 146 - A novel proton CT biologic phantom is proposed to verify x-ray CT calibration in proton treatment planning

#### Elena Fogazzi^1,2^, Mara Bruzzi^3,4^, Carlo Civinini^4^, Simon Rit^5^, Monica Scaringella^4^, Marina Scarpa^1,2^, Francesco Tommasino^1,2^, Diego Trevisan^6^, Paolo Farace^2,6^

##### ^1^University of Trento, Physics department, Trento, Italy ^2^Italian National Institute of Nuclear Physics INFN, Trento Institute for Fundamental Physics and Applications TIFPA, Trento, Italy ^3^University of Florence, Physics and Astronomy, Sesto Fiorentino Florence, Italy ^4^Italian National Institute of Nuclear Physics INFN, Florence division, Sesto Fiorentino Florence, Italy ^5^University of Lyon, INSA Lyon- Université Claude Bernard Lyon- UJM Saint Etienne- CNRS Inserm, Lyon, France ^6^Hospital of Trento APSS, Medical Physics Unit, Trento, Italy

**Purpose**: A novel biological phantom has been developed at the Trento Proton Therapy Center, in collaboration with the INFN and the University of Trento. Based on that, we designed the first clinical application for the INFN proton computed tomography (pCT) system, aiming at introducing an advanced method for the verification of x-ray CT (xCT) calibration in proton treatment planning.

**Methods**: The bio-phantom is made of biologic inserts of a bovine specimen, stabilized with a formalin solution and embedded in agar-agar gel in a plastic housing (Fig.1). The bio-phantom 3D relative stopping power (RSP) map is obtained by the INFN pCT system. The RSP map is then compared with that one obtained from the bio-phantom’s HU map by conventional xCT calibration implemented in the Treatment Planning System (TPS).

**Results**: A standard xCT calibration obtained by an electron-density phantom was exemplarily tested in head configuration and compared with the pCT reconstruction (Fig.2). The RSP difference between pCT and xCT was within 3.5% of the RSP values for most of the voxels, except for bone tissue and for partial volume effects observed at tissues’ boundaries.

**Conclusions**: By scanning the phantom with both pCT and any xCT system, it is possible to verify the corresponding xCT calibration for proton treatment planning, using the pCT bio-phantom as a reference standard and overcoming the limitation of standard synthetic phantoms. The bio-phantom might be temporarily shipped for xCT measurements to any proton therapy center not equipped with a pCT system for a multi-center survey.

### O 147 - Evaluation of single energy and dual energy computed tomography simulation methods for pencil Beam scanning proton therapy planning

#### Sina Mossahebi^1^, Pouya Sabouri^2^, Antony Koroulakis^3^, Dina Cusatis^1^, Lehman Kara^1^, Patrick Wohlfahrt^4^, Jainil Shah^4^, Jason Molitoris^1^

##### ^1^University of Maryland School of Medicine, Radiation Oncology, Baltimore, USA ^2^University of Arkansas for Medical Sciences, Radiation Oncology, Little Rock, USA ^3^Anne Arundel Medical Center, Radiation Oncology, Annapolis, USA ^4^Siemens Healthineers, Medical Imaging, Forchheim, Germany

**Purpose**: To mitigate proton range uncertainties (RU), the irradiation volume is generally expanded beyond the target. A byproduct of this expansion is elevated doses to surrounding organs at risk (OARs). Prior phantom and animal tissue studies have demonstrated that dual-energy CT (DECT) can lower RU. We quantify the expected differences that arise from using DECT or single-energy CT (SECT) images for intensity modulated proton therapy (IMPT) planning.

**Methods**: SECT and DECT scans of 60 IMPT patients (21 HN, 20 pelvis, 11 breast, 8 brain) were acquired under IRB approval. Clinical plans generated on the SECT images were recomputed on the corresponding DECT image. For each patient group, target coverage, mean and maximum OAR doses were compared.

**Results**: Systematic differences in target minimum dose, and V100% were observed. On average, V100% was overestimated by 1.3%(HN), 2.4%(pelvis), 3.3%(breast), 1.2%(brain) when SECT scan was used for planning relative to DECT scan. In target minimum dose, variations as high as 7.7%(HN), 3.1%(pelvis), 7.8%(breast), 2.6%(brain) were seen with DECT indicating target under-dosage. Since all clinical plans were optimized robustly to meet V95% coverage, V95% (HN:99.8%±0.5%, pelvis:99.9±0.2%, breast:98.5±2.8%, brain:100.0±0.1%) and D95 (HN:99.6±1.1%, pelvis:99.6±0.3%, breast:99.1±0.7%, brain:99.9±0.8%) were in higher agreement (<1.5%). For the majority of patients (39/60), DECT indicated higher OAR maximum doses for serial organs.

**Conclusion**: The reduction in RU associated with DECT implies the potential for more conformal proton plans. Moreover, utilization of DECT images in treatment planning improves accuracy of computed target coverage and OAR dose, thereby improving overall treatment quality.

### O 148 - On the feasibility of high-dimensional multivariate machine learning model-based outcome optimization for intensity-modulated treatment planning in proton therapy

#### Tim Ortkamp^1,2,3^, Oliver Jäkel^2,3,4,5^, Martin Frank^1,2^, Niklas Wahl^3,5^

##### ^1^Karlsruhe Institute of Technology, Steinbuch Centre for Computing, Karlsruhe, Germany ^2^Helmholtz Information and Data Science Academy, Hemholtz Information and Data Science School for Health, Karlsruhe/Heidelberg, Germany ^3^German Cancer Research Center, Medical Physics in Radiation Oncology, Heidelberg, Germany ^4^Heidelberg Ion Beam Therapy Center, Medical Physics in Radiation Oncology, Heidelberg, Germany ^5^Heidelberg Institute for Radiation Oncology, Medical Physics in Radiation Oncology, Heidelberg, Germany

Machine Learning (ML) models on normal tissue complication or tumor control probability ((N)TCP) exploiting e.g. dosiomics and radiomics features are advancing radiotherapy treatment outcome prediction. Due to the model complexity and the potentially high dimensionality of the feature space, their integration into explicit (N)TCP model-based IMRT plan optimization may be non-intuitive. In this study, we intend to show that integrating high-dimensional multivariate ML models is feasible, and can lead to acceptable dose distributions which improve upon (N)TCP. We established a new Python module (*pyanno4rt*) to optimize treatment plans for intensity-modulated photon and proton therapy, including data preprocessing, outcome model training via sequential model-based optimization, and input feature (re-)calculation. On a set of 24 common dosiomics and radiomics features, plan optimization can be conducted with automatic differentiation of feature and model functions using the Python library *JAX*, followed by nonlinear quasi-Newton methods using the *IPOPT* solver. As a proof of concept, we fitted a multivariate logistic regression model for grade 2+ long-term xerostomia risk assessment based on 48 dosiomics and radiomics features of the parotid glands over a cohort of 153 head-and-neck cancer patients, and utilized model-based objective functions to optimize treatment plans. Results (see figure 1) indicate that applying the model indeed lowers the estimated NTCP, without compromising plan quality. This improvement in NTCP can be attributed in part to changes in the values of high-impact features, as can be seen in table 1. In summary, our results show the feasibility of high-dimensional multivariate outcome model-based plan optimization in photon and proton therapy.

### O 149 - An orthogonal matching pursuit optimization method for proton IMPT, ARC and FLASH with large-minimum-MU constraints

#### Ya-Nan Zhu^1^, Xiaoqun Zhang^1^, Yuting Lin^2^, Chris Lominska^2^, Hao Gao^2^

##### ^1^Shanghai Jiao Tong University, Institute of Natural Sciences and School of Mathematics, Shanghai, China ^2^University of Kansas Medical Center, Radiation Oncology, Kansas City, USA

**Purpose**: The intensities of proton spots need to meet the minimum-MU (MMU) threshold in order for the spots to be deliverable. Since the dose rate is proportionally associated with the MMU threshold, higher-dose-rate proton RT (e.g., for efficient IMPT and proton ARC, and FLASH) needs to solve the MMU problem with large MMU threshold, which however makes nonconvex problem difficult to solve. The conventional optimization methods usually cannot satisfactorily handle large MMU threshold, for which this work will develop an effective optimization method based on orthogonal matching pursuit (OMP).

**Methods**: The new method consists of two essential components: (1) iterative convex relaxation method is used to determine the active sets for dose-volume planning constraints and decouple the MMU constraint from the rest.; and (2) a modified OMP optimization algorithm is used to handle the MMU constraint: the non-zero spots are greedily selected via OMP to form the solution set to be optimized; and then a convex constrained subproblem is formed and can be conveniently solved to optimize the spot weights restricted to this solution set via OMP. During this iterative process, the new non-zero spots localized via OMP will be adaptively added to or removed from the optimization objective.

**Results**: The new method via OMP is compared with ADMM and a state-of-the-art method called stochastic coordinate descent (SCD) for high-dose-rate IMPT, ARC, and FLASH problems of large MMU thresholds, and the results suggest that OMP substantially improved the plan quality from ADMM and SCD.

### O 150 - Reduced radiation exposure to circulating-blood-cells in proton-therapy compared with X-ray therapy in locally-advanced lung cancer: Computational simulation based on circulating-blood-cells

#### Hyunki Park^1^, Nalee Kim^1^, Jungwook Shin^2^, Sung Hwan Ahn^1^, Hongryull Pyo^1^, Jae Myoung Noh^1^, Kyungmi Yang^1^, Woojin Lee^1^, Byoungsuk Park^1^

##### ^1^Samsung Medical Center- Sungkyunkwan University School of Medicine, Department of Radiation Oncology, Seoul, Korea Republic of ^2^National Cancer Institute- National Institutes of Health, Division of Cancer Epidemiology and Genetics, Rockville- MD 20850, USA

**Background**: We estimated the dose of circulating blood cells (CBCs) in patients with locally advanced non-small cell lung cancer for predicting severe radiation-induced lymphopenia (SRIL) and compared pencil-beam scanning proton therapy (PBSPT) and intensity-modulated (photon) radiotherapy (IMRT).

**Materials and Methods**: After reviewing 325 patients who received definitive chemoradiotherapy with PBSPT (n = 37) or IMRT (n = 164). SRIL was diagnosed when two or more events of an absolute lymphocyte count < 200 µL occurred during the treatment course. Dose information for the heart and lungs was utilized for the time-dependent computational dose calculation of CBCs.

**Results**: The dose distribution of CBCs was significantly lesser in the PBSPT group than that in the IMRT group. Overall, 75 (37.3%) patients experienced SRIL during the treatment course; 72 and 3 patients were treated with IMRT and PBSPT, respectively. SRIL was associated with poor progression-free and overall survival outcomes. Upon incorporating the dose information of CBCs for predicting SRIL, CBC D90% > 2.6 GyE was associated with the development of SRIL with the baseline lymphocyte count and target volume. Furthermore, PBSPT significantly reduced the dose of CBC D90% (odds ratio = 0.11; *p* = 0.004) compared with IMRT.

**Conclusion**: The results of this study demonstrate the significance of the dose distribution of CBCs in predicting SRIL. Furthermore, reducing the dose of CBCs after PBSPT minimized the risk of SRIL. Lymphocyte-sparing radiotherapy in PBSPT could improve outcomes, particularly in the setting of maintenance immunotherapy.

### O 151 - The model-based selection of lung cancer patients for intensity modulated proton radiotherapy

#### Robin Wijsman^1^, Olga Chouvalova^1^, Pieter Deseyne^1^, Annija Van der Leest^1^, Marleen Woltman-van Iersel^1^, Fred Ubbels^1^, Erik Korevaar^1^, Stefan Both^1^, Johannes Langendijk^1^

##### ^1^University Medical Center Groningen, Radiation oncology, Groningen, Netherlands

**Purpose**: Since October 2019, 198 lung cancer patients have been treated with intensity modulated proton radiotherapy (IMPT) after model-based selection (MBS). The objective of this study was to report on MBS.

**Material and Methods**: Lung cancer patients referred for definitive (chemo-)radiotherapy (n=466) were selected for IMPT using MBS. MBS is based on the difference in NTCP (ΔNTCP) for Grade≥2 radiation pneumonitis (RP, threshold 10%), Grade≥2 acute esophageal toxicity (AET, threshold 10%) and/or 2-year mortality (2yM, threshold 2%) based on a plan comparison (IMPT vs. VMAT). The dosimetric parameters in these NTCP models are mean lung dose (MLD), mean esophagus dose (MED) and mean heart dose (MHD). IMPT plans were created using a 3D robustly optimized planning technique (3 beams, 5 times layered rescanning).

**Results**: After MBS, 42% of patients qualified for IMPT. The plan comparisons (IMPT vs VMAT) showed significantly lower MHD (3.7 Gy vs. 8.8 Gy; p<0.001), MLD (9.4 Gy vs. 13.2 Gy; p<0.001) and MED (15.9 Gy vs. 18.7 Gy; p<0.001) for IMPT. This resulted in the NTCPs shown in Figure 1. Most patients qualified for IMPT based on an improvement in 2yM only (n=133, 67%). Improvement of 2yM together with lower risk of AET or RP was seen in 26 (13%) and 24 (12%) patients, respectively (Table 1).

**Conclusion**: Model-based selection of lung cancer patients for IMPT shows that (compared to VMAT) IMPT may be beneficial for a substantial number of patients in terms of lower risk of toxicity.

### O 152 - Carbon ion radiotherapy for patients with stage III non-small-cell lung cancer

#### Ningyi Ma^1^, Jian Chen^1^, Xue Ming^2^, Jingfang Mao^1^, Guo-Liang Jiang^3^, Kailiang Wu^3^

##### ^1^Shanghai Proton and Heavy Ion Center- Shanghai Key Laboratory of radiation oncology 20dz2261000- Shanghai Engineering Research Center of Proton and Heavy Ion Radiation Therapy, Department of Radiation Oncology, Shanghai, China ^2^Shanghai Proton and Heavy Ion Center- Shanghai Key Laboratory of radiation oncology 20dz2261000- Shanghai Engineering Research Center of Proton and Heavy Ion Radiation Therapy, Department of Medical Physics, Shanghai, China ^3^Shanghai Proton and Heavy Ion Center- Shanghai Key Laboratory of radiation oncology 20dz2261000- Shanghai Engineering Research Center of Proton and Heavy Ion Radiation Therapy- Fudan University Cancer Hospital- Fudan University, Department of Radiation Oncology, Shanghai, China

**Purpose**: To evaluate the survival and toxicities after carbon-ion radiotherapy (CIRT) using the pencil beam scanning technique in patients with stage III non-small cell lung cancer (NSCLC).

**Methods**: This study evaluated patients with stage III NSCLC who received CIRT between December 2016 and August 2021 in Shanghai Proton and Heavy Ion Center. CIRT was delivered with a median relative biological effectiveness-weighted dose of 79.2 (69–83.6) Gy/22 (19–24) fractions with a fraction size of 3.0–4.0 Gy. Toxicities were evaluated according to the CTCAE version 4.0. Kaplan-Meier methods was used for survival analysis.

**Results**: One hundred and three patients were included. There were 89 males and 14 females with a median age of 65 years (44-81). 34 were adenocarcinoma, 52 was squamous cell carcinoma, and 17 were non-specified or other NSCLC. Among them, 43 had AJCC stage IIIa, 50 stage IIIb and 10 stage IIIc tumors. The median follow-up time was 20.3 months, the median overall survival (OS) and progression-free survival (PFS) was 32.3 months and 16 months, respectively. The 2-year OS, local progression-free survival (LPFS), and distant metastasis-free survival rates were 63.3%, 71.4%, and 64.4%, respectively. Grade 3 CIRT-related acute and late toxicities occurred in 5.8% and 8.7% of patients, respectively, and no grade 4–5 side effects were observed.

**Conclusions**: CIRT resulted in mild toxicity with comparable OS and LPFS for stage III NSCLC. Prospective studies of CIRT for stage III NSCLC are warranted to better identify the optimal fractionation.

### O 153 - Hypoxia predicts favorable response to carbon ion radiotherapy in NSCLC defined by 18F-FMISO PET/CT Imaging

#### Jingyi Cheng^1^, Mao Jingfang^2^

##### ^1^Shanghai Proton and Heavy Ion Center, Nuclear Medicine, Shanghai, China ^2^Shanghai Proton and Heavy Ion Center, Radiation oncology, Shanghai, China

**Objective**: This study aimed to evaluate the changes of hypoxia before and after carbon ion radiotherapy (CIRT) in patients with non-small cell lung cancer (NSCLC) and whether ^18^F-fluoronitroimidazole (FMISO) PET/CT imaging could predict the response to CIRT in NSCLC patients.

**Methods**: Twenty-nine patients with NSCLC who received CIRT were retrospectively included. ^18^F-FMISO PET/CT imaging was performed before and after treatment, and chest CT was performed after radiotherapy. The evaluation of efficacy within one week after radiotherapy was defined as the immediate response (IR). And the efficacy evaluation at the first follow-up after radiotherapy was defined as early response (ER). The tumor to muscle ratio (TMR), hypoxia volume (HV), and the ΔTMR and ΔHV value of the corresponding changes before and after treatment of ^18^F-FMISO uptake were recorded.

**Results**: ①Baseline TMR could predict the immediate response with a cut-off value of 2.35, an AUC of 0.85 (0.62-1.00), a sensitivity of 80.0%, a specificity of 87.5% and an accuracy of 85.7%. Taking the baseline TMR=2.35 as the cut-off value, the high-hypoxia group (6 cases) and the low-hypoxia group (15 cases) were divided. The response rate of the high-hypoxia group was 66.7% (4/6), while in low-hypoxia group was 6.7% (1/15), (*P*=0.011). ②ΔTMR could predict early treatment response after CIRT at initial follow-up, with cut-off value of 36.6%, AUC of 0.80 (0.61-1.00), sensitivity of 72.7%, specificity of 90.0% and accuracy of 71.4%.

**Conclusion**: The higher the degree of tumor hypoxia, the better the response to CIRT, c ΔTMR could predict early treatment response after CIRT.

### O 154 - Inter and intra scanner reproducibility of DECT calculated SPR for proton dose calculation

#### Shannon O’reilly^1^, Taoran Li^1^, Wei Zou^1^, Alexander Lin^1^, Michelle Alonso-Basanta^1^, J. Nicholas Lukens^1^, Michelle Gentile^1^, Goldie Kurtz^1^, Lei Dong^1^, Kevin Teo^1^

##### ^1^University of Pennsylvania, Radiation Oncology, Philadelphia, USA

**Purpose**: A method to utilize dual energy computed tomography (DECT) derived proton stopping power ratio (SPR) CT images for optimization with reduced range uncertainty of 2% was developed and clinically implemented for over 700 patients (head and neck and brain) at our institution since 2020. Two models of DECT scanners (single- and dual-source) are used clinically. Repeat DECTs of patients performed on the same scanner versus different model scanners were analyzed.

**Methods**: Thirty-five patients (n=21 same scanner, n=14 different scanner) who had multiple DECT-based SPR images generated during their treatment course were analyzed to determine SPR reproducibility for adipose, brain, muscle, and dense bone. The modified CT images used in the treatment planning system were created using SPR-derived from electron density (ρ_e_) and effective atomic number (Z_eff_) images that were converted into an importable CT using an inverted HU-SPR calibration curve. The single-source scanner utilized vendor calculated ρ_e_ and Z_eff_ while the dual-source used an in-house ρ_e_ and Z_eff_ calculation.

**Results**: For patients imaged on the same scanner, SPR-derived CT scans were found to be reproducible with average SPR differences of <0.1% for adipose, brain and muscle and <0.2% for dense bone. For those imaged on different scanners, greater variability was observed; however, scans were found to be reproducible with average SPR differences of <0.1% for adipose, <0.2% for muscle, and <0.3% for brain and dense bone.

**Conclusion**: DECT**-**calculated SPR are reproducible across two different DECT scanners utilizing different calibration methodologies, allowing scanners to be used interchangeably for treatment planning and treatment.

### O 155 - Assessment of set-up accuracy in breast patients treated with proton therapy when using surface imaging as compared to kV-based techniques

#### Daniel Cho^1,2^, Alonso Gutierrez^1,2^, Andrew Wroe^1,2^

##### ^1^Miami Cancer Institute, Radiation Oncology, Miami, USA ^2^Florida International University, Radiation Oncology, Miami, USA

**Purpose**: We evaluated the accuracy of a three camera CatalystPT Surface Imaging System (C-RAD) for daily breast set-up alignment in Proton Therapy when compared to kV-based techniques, namely orthogonal planar and CBCT imaging.

**Methods**: Initial positioning of breast patients for proton therapy is accomplished using C-RAD followed by orthogonal planar x-rays and/or CBCT to finalize pre-treatment localization. Immediately after patient positioning, couch coordinates and residual shifts on C-RAD are captured and saved to the treatment record. In this study, the difference between final x-ray aligned position and residual C-RAD differences were analyzed in ninety-seven patients (n=97) treated between 2019 and 2022. Data was correlated with age and BMI to determine if either of these factors impacted set-up accuracy.

**Results**: For each patient, we calculated the mean displacement difference over the entire course of treatment reported by C-RAD and x-ray alignment. A mean of all the patient means was then calculated. The mean displacement difference between x-ray based alignment and C-RAD was found to be 2.5±1.1 mm (lateral), 2.8±1.2 mm (longitudinal), 2.5±1.0 mm (vertical), 1.2±0.5° (Yaw), 1.0±0.5°(Pitch) and 1.0±0.5°(Roll). Pearson Correlation Coefficients (PCC) between patient’s Age & BMI and mean displacement difference was calculated. BMI showed some correlation but was not statistically significant (PCC=0.18, p-value = 0.075). No correlation between Age and mean displacement was noted. (PCC=0.004, p-value = 0.80705).

**Conclusion**: Across a large number of patients & fractions, C-RAD was able to position patients to within 3mm of isocenter as compared to x-ray based imaging techniques.

### O 156 - Patterns of practice of image-guided particle therapy for cranio-cpinal axis irradiation: A site-specific multi-institutional survey of European Particle Therapy Network

#### Petra Trnkova^1^, Alessandra Bolsi^2^, Aswin Hoffmann^3^

##### ^1^Medical University of Vienna, Department of Radiation Oncology, Wien, Austria ^2^Paul Scherrer Institute, Center for Proton Therapy, Villigen, Switzerland ^3^University Hospital Carl Gustav Carus, Proton Therapy Center, Dresden, Germany

Image guidance is one of the key components of precise particle therapy (PT). Clinical workflows, integrated devices and quality assurance procedures for image-guided particle therapy (IGPT) may vary across the institutes. Therefore, a survey to evaluate the status of site-specific IGPT across the PT institutes in Europe was conducted. Here, we report the results for cranio-spinal axis (CSA) irradiation. Eleven PT centres treating CSA, especially in paediatric patients, participated in the survey. Five centres treated routinely in prone position. Depending on age, treatment time and level of co-operation, patients were treated under anaesthesia using specific paediatric moldcare for immobilization to shell the whole patient. For planning imaging, 10 centres used a single-energy, one dual-energy CT. Six centres used additional MR images for delineation. Three centres used routinely surface imaging for initial positioning or surveillance. Two or six centres performed routinely or on-demand, respectively, imaging for evaluation of interfractional changes, using the same imaging modalities as for initial treatment planning. As CSA treatment fields are often long using several isocentres and merged fields, a daily setup procedure took up to 45 minutes. All centres perform verification imaging before every fraction, eight before each isocentre. Ten centres performed 2D IGPT, one 3D IGPT. The centres had indicated that faster and more streamlined workflows are needed to improve IGPT in CSA. The vendors should improve imaging, especially cone-beam CT and provide better solutions for multi-isocentre treatment. Standardization and guidelines for CSA irradiation are strongly needed to harmonize the clinical practice across the institutes.

### O 157 - Towards using the attenuation reconstruction from ToF-PET scans for dose calculation in proton therapy

#### Christian Bäumer^1^, Claus Maximilian Bäcker^1^, Maurizio Conti^2^, Walter Jentzen^3^, Simon Schmidt^1^, Mohammadreza Teimoorisichani^2^, Jörg Wulff^1^, Beate Timmermann^4^

##### ^1^West German Proton Therapy Centre, Medical Physics, Essen, Germany ^2^Siemens Medical Solutions, PET Physics and Reconstruction, Knoxville, Germany ^3^University Hospital Essen, Clinic for Nuclear Medicine, Essen, Germany ^4^West German Proton Therapy Centre, Department of Particle Therapy, Essen, Germany

PET-scanners with time-of-flight (ToF) option allow for the reconstruction of gamma-ray attenuation CTs (γCTs) [1]. These use 511 keV photons from β^+^ activity distributed in the patient as the radiation source for anatomical imaging. Activity and γ-attenuation are jointly reconstructed using the Maximum Likelihood Estimation of Attenuation and Activity. γCTs are suitable for dose calculation in proton therapy, because both the γ-attenuation at 511 keV and the slowing-down of protons scale with electron density n_e_ [2]. A phantom was equipped with cylinders of tissue substitutes and filled with ^18^F fluorodeoxygluocose solution. A PET scan with a time resolution of 210 ps was performed. The CT numbers of the cylinders, the noise, and the spatial resolution of the γCT were determined. Dose was computed on the γCT and compared with one based on X-ray CTs (xCTs). A γCT was successfully reconstructed (Fig. 1). The CT numbers of the tissue substitutes scaled linearly with n_e_. The standard deviation was 0.018 (in units relative to water). Despite the poorer resolution (6 mm FWHM) and higher noise (2.8% standard deviation) compared with xCTs, the same dose distribution resulted, as demonstrated by a gamma- index pass rate of 99.8% (2 mm/2%). Summarizing, radiation planning in proton therapy using gamma-beam attenuation CTs from ToF-PET scanners was demonstrated. Given the linear dependence on n_e_, the yCTs offer a potentially better assignment of stopping powers in a patient.

### O 158 - Analysis of radiographic images for setup verification in proton therapy treatment of ocular melanoma

#### Giulia Sellaro^1^, Andrea Pella^1^, Matteo Pepa^1^, Mario Ciocca^2^, Maria Rosaria Fiore^3^, Alessandro Vai^2^, Agnieszka Chalaszczyk^3^, Marco Rotondi^3^, Ester Orlandi^4^, Guido Baroni^1,5^

##### ^1^National Center of Oncological Hadrontherapy CNAO, Clinical Department - Bioengineering Unit, Pavia, Italy ^2^National Center of Oncological Hadrontherapy CNAO, Clinical Department - Medical Physics Unit, Pavia, Italy ^3^National Center of Oncological Hadrontherapy CNAO, Clinical Department - Radiotherapy Unit, Pavia, Italy ^4^National Center of Oncological Hadrontherapy CNAO, Clinical Department, Pavia, Italy ^5^Politecnico di Milano University, Department of Electronics Information and Bioengineering, Milan, Italy

In ocular proton therapy (OPT), image guidance and setup verification rely on X-ray images of tantalum clips sutured on the sclera of the disease eye prior to radiation therapy. In CNAO (Centro nazionale di Adroterapia Oncologica,Pavia,Italy) set-up corrections are defined through point-based rigid registration between clips three dimensional position and corresponding nominal configuration. A 3-Dof correction vector is consequently applied to the treatment chair aiming at minimizing set-up deviations. During irradiation, radiographic imaging is also used for a qualitative evaluation of eye stability. In the clinical routine a manual clip detection is done and clip registration is performed by a commercial software (VeriSuite,Medcom GmbH,2012). Goal of this study is to make available first results of an offline clip registration tool with semi-automatic segmentation. A dataset consisting of 40 patients who received OPT at CNAO in 2022 was considered. We collected all the in-room X-ray images acquired along the course of the treatment (mean Xray acquisitions per patient=34; 294 applied correction vectors). Results are presented as the residuals between the performance of the presented novel semi-automatic method against correction vectors returned by the commercial software. Overall, we found a median difference between the two registration tool (IQR)[mm] of -0.03(0.22), 0.0(0.12),-0.01(0.18) in medio-lateral, cranio-caudal and antero-posterior, respectively (Figure1,b). Efforts will be devoted to make a more in-depth study of intra-fractional stability as a way to possibly reduce the number of X-rays required for setup verification. Further investigation will be also dedicated to the optimization of imaging parameters to enhance metal detection.

### O 159 - Proton radiotherapy reduces the risk of pneumonitis in esophageal cancer patients

#### Christina Muijs^1^, Margriet Dieters^1^, Erik Korevaar^1^, Boudewijn van Etten^2^, Jan Willem Haveman^2^, Charlotte IJsbrandy^1^, Jannet Beukema^1^, Johannes Langendijk^1^

##### ^1^University Medical Center Groningen, Radiation Oncology, Groningen, Netherlands ^2^University Medical Center Groningen, Surgical Oncology, Groningen, Netherlands

**Background**: Neo-adjuvant chemoradiotherapy (nCRT) followed by surgical resection is the treatment of choice for patients with locally advanced esophageal cancer. This treatment approach is associated with various (postoperative) complications, such as pneumonitis. The aim of this study was to evaluate the impact of radiotherapy technique (photon vs proton RT) on the risk of pneumonitis.

**Material and Methods**: The study population was composed of 426 patients treated with nCRT (41.4 Gy) +/− surgical resection. All patients were entered in a prospective data registry. Intensity Modulated Proton Therapy (IMPT) was applied since April 2020 to patients who qualified for IMPT based on an estimated risk reduction of the 2-year mortality, according to model-based selection. The primary endpoint was pneumonitis (CTCAE v5.0) within 6 months after treatment and all types of pneumonia were considered. Photon RT consisted of Intensity Modulated RadioTherapy (IMRT) or Volumatric Modulated Arc Therapy (VMAT).

**Results**: Patient characteristics are listed in Table 1. Within 6 months after nCRT, 100 patients (23.5%) developed pneumonitis grade II or higher. After proton RT, 7 out of 95 (7.4%) patients developed pneumonitis compared to 28.3% (93/329) after photon RT (p<0.01). In multivariate analysis, pneumonitis was significantly associated with age, resection (no vs yes) and RT technique (photons vs. protons) (OR: 0.27, 0.12-0.62) (see Table 2).

**Conclusion**: The rate of pneumonitis was significantly lower after proton RT compared to photon RT.

### O 160 - Proton- vs. photon-based radiotherapy for esophageal cancer: Electrocardiogram-based assessment of cardiac toxicity

#### Roman Kowalchuk^1^, William Breen^1^, William Harmsen^2^, Taylor Weiskittel^3^, Zachi Attia^4^, Joerg Herrmann^4^, Krishan Jethwa^1^, Michael Haddock^1^, David Routman^1^, Christopher Hallemeier^1^

##### ^1^Mayo Clinic, Radiation Oncology, Rochester, USA ^2^Mayo Clinic, Quantitative Health Sciences, Rochester, USA ^3^Mayo Clinic, School of Medicine, Rochester, USA ^4^Mayo Clinic, Cardiology, Rochester, USA

**Objectives**: Though radiotherapy is an integral part of the treatment paradigm for esophageal cancer, it can cause a range of cardiac toxicities. We sought to explore whether electrocardiograms (ECGs) could predict clinical outcomes for patients and whether proton therapy could reduce toxicity.

**Methods**: All patients who received at least 41.4 Gy for esophageal cancer from 2015 through July 2022 were included. ECGs were analyzed with a previously validated artificial-intelligence assessment for atrial fibrillation (AF) (Noseworthy et al. Lancet 2022). This algorithm also demonstrated propensity for reduced ejection fraction (rEF). In addition to calculated propensity, cardiac events were extracted from the medical record and recorded.

**Results**: A total of 491 patients were analyzed: 235 (48%) had esophagectomy, and most had lower third esophageal cancer (84%). Median follow-up was 2.9 years. Of these, 233 (47%) were treated with photons and 258 (53%) with protons. For the entire cohort, after radiotherapy, patient ECGs had a higher propensity for AF (Figures 1-2, p<0.0001) and rEF (p<0.0001). No differences were identified between proton and photon therapy regarding propensity for AF or rEF (p=0.16 and p=0.71, respectively). Dosimetric parameters for patients treated with proton therapy were not predictive of propensity for cardiac toxicity.

**Conclusions**: Radiation therapy for esophageal cancer was associated with increased risk for multiple cardiac events. ECG may help stratify patients by risk for subsequent cardiac events. In this dataset, proton therapy did not clearly demonstrate a clinically meaningful reduction in cardiac toxicity, so we recommend assessment of cardiac substructures to determine dosimetric relationships.

### O 161 - Pencil beam scanning (PBS) intensity modulated proton radiotherapy (IMPT) – dose escalation for advanced cervical oesophagus cancer (CEC)

#### Pavel Vitek^1^, Jiri Kubes^1^, Vladimir Vondracek^1^, Katerina Jirankova^1^, Radek Zapletal^1^

##### ^1^Proton Therapy Center Czech, Proton radiotherapy, Praha 8, Czech Republic

Definitive chemoradiation (dCRT) is a standard therapy for CEC. Higher doses up to 60-66 Gy may be appropriate, but still have not been established as standard despite CEC may behave very aggressively. Favorable dosimetry of PBS IMPT may allow safe administration of doses up to 70 GyE, however a positive clinical outcome remains questionable. Single institution results are presented. PBS proton radiotherapy was administered in patients with advanced CEC (T3-4 or N+) in 2 phases: 50 GyE/25 fractions to cervical esophagus + mediastinal, cervical lymphnodes, next 20 GyE/10 fractions to the involved part of esophagus + involved lymphnodes. Concurrent chemotherapy carboplatinum + paclitaxel. Twenty-nine (29) pts. (27 SCC, 2 adenocarcinoma) were treated, 26 were evaluable. Median dose was 70 GyE (56-70). Response: 15 pts. achieved a complete regression (57,7%), 8 pts. partial regression (30,8%), 3 pts. stable and/or progressive disease (11,5%). 11 pts. (42,3%) are alive after a median follow up 10,2 months (1,6-51,7). Median overall survival 14 months (8,6-30,2). 3 pts. remain in complete regression past 3 years. 12 pts. (46%) developed a relapse (locoregional/distant) after a median time 29,5 months. Acute toxicity was modest (≤ gr 2 only), severe late toxicity (grade 3-4) included stenosis (2), fistulation (1), ulceration(1). PBS IMPT dose escalation for advanced CEC showed a good efficacy with acceptable acute toxicity. The relapse rate remains high and survival poor despite a small patient group is cured and achieves a durable response. The relationship between the late toxicity (probably unravelled) and survival data warrants further analysis.

### O 162 - Multi-institutional outcomes of proton beam therapy for hepatocellular carcinoma from the Proton Collaborative Group REG001-09 study

#### Michael Chuong^1^, Amanda Zakka^2^, Arpit Chhabra^3^, Jason Molitoris^4^, Jonathan Ashman^5^, Nasir Mohammed^6^, John Chang^7^, James Urbanic^8^, Michael Eblan^9^, Smith Apisarnthanarax^10^

##### ^1^Miami Cancer Institute, Radiation Oncology, Miami, USA ^2^Florida International University, Herbert Wertheim College of Medicine, Miami, USA ^3^New York Proton Center, Radiation Oncology, New York, USA ^4^Maryland Proton Treatment Center, Radiation Oncology, Baltimore, USA ^5^Mayo Clinic, Radiation Oncology, Scottsdale, USA ^6^Northwestern Medicine, Radiation Oncology, Chicago, USA ^7^Oklahoma Proton Center, Radiation Oncology, Oklahoma City, USA ^8^California Protons Treatment Center, Radiation Oncology, San Diego, USA ^9^Inova Schar Cancer Institute, Radiation Oncology, Fairfax, USA ^10^University of Washington, Radiation Oncology, Seattle, USA

**Background**: Proton beam therapy (PBT) is associated with favorable long-term freedom from local progression (FFLP) and overall survival (OS). Most PBT data for HCC are from Asia and thus there is a lack of prospectively reported outcomes from Western countries.

**Methods**: We evaluated 82 HCC patients on the Proton Collaborative Group REG001-09 prospective registry study (NCT01255748) treated with PBT in the U.S. Primary objective was describing FFLP, freedom from intrahepatic progression (FFIP), freedom from distant progression (FFDP), progression-free survival (PFS), OS, and acute/late toxicity.

**Results**: Median tumor size was 5.0 cm (range, 1.2-19 cm). Baseline Child-Pugh (CP) class was A, B, C, or unknown in 40.2%, 15.9%, 2.4%, and 41.5%, respectively. Median PBT dose was 59.3 Gy in a median 15 fx (range, 5-36 fx); median BED_10_ was 85.2 Gy. Most had pencil beam scanning (PBS) (56.1%) versus uniform scanning (32.9%). Median follow-up from diagnosis and PBT was 26 months and 13.5 months, respectively. Median FFLP, FFIP, FFDP, PFS, and OS from PBT start were not reached, 37 months, not reached, 14 months, and 26 months, respectively. On MVA, worse OS was significantly associated with age ≥median while improved OS was associated with BED_10_ ≥median. No acute/late grade 3+ toxicity was observed.

**Conclusions**: Dose-escalated PBT for HCC achieves excellent long-term FFLP with no grade 3+ toxicity in a Western population. These outcomes are noteworthy given the large tumor sizes ranging up to 19 cm. PBT should be strongly considered over photon therapy for larger HCC to safely deliver the highest possible BED_10._

### O 163 - Clinical benefit of carbon ion radiotherapy for recurrent/refractory ovarian/salpinx cancer

#### Amelia Barcellini^1,2^, Noriyuki Okonogi^3^, Giulia Fontana^4^, Alessandro Vai^5^, Silvia Molinelli^5^, Chiara Cassani^6,7^, Simona Secondino^8^, Kazutoshi Murata^3^, Shigeru Yamada^3^, Ester Orlandi^1^

##### ^1^National Center for Oncological Hadrontherapy CNAO, Clinical Department- Radiation Oncology Unit, Pavia, Italy ^2^University of Pavia, Department of Internal Medicine and Medical Therapy, Pavia, Italy ^3^QST Hospital- National Institutes for Quantum Science and Technology, Department of Radiation Oncology, Chiba, Japan ^4^National Center for Oncological Hadrontherapy CNAO, Clinical Department, Pavia, Italy ^5^National Center for Oncological Hadrontherapy CNAO, Clinical Department- Medical Physics Unit, Pavia, Italy ^6^IRCCS Fondazione Policlinico San Matteo University of Pavia, Unit of Obstetrics and Gynecology, Pavia, Italy ^7^University of Pavia, Department of Clinical- Surgical- Diagnostic and Pediatric Sciences, Pavia, Italy ^8^IRCCS Fondazione Policlinico San Matteo, Unit of Medical Oncology-, Pavia, Italy

**Aim**: To analyze the real‐world data set of the recurrent/refractory ovarian/salpinx cancer (RR‐OSC) treated with carbon ion radiotherapy (CIRT) at QST (Japan) and CNAO (Italy).

**Materials and Methods**: The endpoints of this study were the rate of objective response (OR), 1 and 2-year local control (LC) and toxicities. OR was defined as the sum of complete response (CR) and partial response (PR). LC was calculated with the Kaplan-Meier method. RTOG/EORTC and CTCAE scales were used to score toxicities according to institute policy.

**Results**: The data of 26 women (58% Asian and 42% Caucasian) consecutively treated with CIRT (range:39-64 Gy[RBE]) for RR-OSC were retrospectively reviewed. Lymph node lesions accounted for 54% followed by parenchymal ones (46%). The median age at CIRT was 59.5 years, and 42% of cases were high-grade serous carcinomas. Patients underwent at least 1 cytoreductive surgery and at least 1 previous line of chemotherapy. Four patients received PARP-i and 6 anti-VEGF before CIRT. No concomitant systemic therapies were administered. CR was achieved in eleven (42%) patients within 6 months after CIRT. With a median follow-up of 16 months, the OR was 85% and 15% of the cases experienced an in-field local recurrence. The 1- and 2-year LC rates were 89% and 74% respectively. Total dose, age, CIRT site and ethnicity did not impact LC or OR. Only one patient experienced G3 acute/late EORTC/RTOG enterocolitis. Toxicity was not related to the use of PARP-i/anti-VEGF.

**Conclusion**: CIRT seems a safe and feasible therapeutic option for RR-OSC. Prospective and randomized studies are warranted.

### O 164 - Development of novel targeted boronated small molecule drugs for BNCT and comparison with BPA in neutron irradiation experiments

#### Kendall Morrison ^1^

##### ^1^TAE Life Sciences, Drug Development, Santa Monica, USA

BNCT is a promising cancer treatment modality for certain indications including head and neck, melanoma, GBM, lung and breast cancer. At TAE Life Sciences we have developed a compact tandem accelerator-based neutron source with a low-energy neutron spectrum (2.5 MeV proton energy) operating at 10mA. The neutron source was installed in a hospital in Xiamen, China, and commissioned to treat cancer patients. In order to increase the efficacy of BNCT, certain shortcomings of BPA (4-boronophenylalanine) must be overcome. Chief among them is the improvement in boron concentration achieved in the tumor. We have previously reported on novel boronated tyrosine small molecules as alternatives to BPA. These exhibited good solubility, cellular uptake and retention. Furthermore, in addition to their easier formulation, they can deliver 2-3 times more boron across multiple cell lines and tumor models than can be achieved with BPA. Additionally, we demonstrated that the boron concentrations in the tumors correlated with the expression of LAT-1 transporter. Currently, these compounds are being evaluated in toxicology studies and in efficacy studies with neutron irradiation using the syngeneic murine CT26 tumor model. The outcomes of the in vivo studies, including BNCT, using these compounds will be presented.

### O 165 - Integrin αvβ3 targeted long retention type boron carrier for Boron Neutron Capture Therapy in F98 rat glioma models

#### Kohei Tsujino^1^, Hideki Kashiwagi^1^, Ryo Hiramatsu^1^, Shin-Ichi Miyatake^2^, Naonori Hu^3^, Hiroki Tanaka^4^, Minoru Suzuki^5^, Hiroyuki Nakamura^6^, Shinji Kawabata^1^, Masahiko Wanibuchi^1^

##### ^1^Osaka Medical and Pharmaceutical University, Department of Neurosurgery, Takatsukishi- Osaka, Japan ^2^Kansai BNCT Medical Center- Osaka Medical and Pharmaceutical University, Department of Neurosurgery, Takatsukishi- Osaka, Japan ^3^Kansai BNCT Medical Center- Osaka Medical and Pharmaceutical University, Department of medical physics, Takatsukishi- Osaka, Japan ^4^Institute for Integrated Radiation and Nuclear Science- Kyoto University, Department of Radiology, Asashiro-Nishi- Kumatori-cho- Sennan-gun- Osaka, Japan ^5^Institute for Integrated Radiation and Nuclear Science- Kyoto University, Department of Radiation and Nuclear Science, Asashiro-Nishi- Kumatori-cho- Sennan-gun, Japan ^6^Laboratory for Chemistry and Life Science- Institute of Innovative Research- Tokyo Institute of Technology, Department of Chemistry, Nagatsuta-cho- Midori-ku- Yokohama, Japan

Boron neutron capture therapy (BNCT) is a particle radiation modality capable of selectively destroying tumor cells. The most commonly used boron carrier is boronphenylalanine (BPA). BPA is taken up into the tumor cell via the L-type aminoacid transporter (LAT-1). However, there are some BPA-refractory situations. Therefore, a novel boron carrier is expected to improve the therapeutic performance of BNCT. In this study, a novel boron carrier which contained maleimide-functionalized closo-dodecaborate (MID), serum albumin as a drug delivery system, and cyclic RGD (cRGD) which can target integrin α_v_β_3_ was developed. We evaluated the efficacy of BNCT using cyclic RGD-functionalized MID-albumin conjugate (cRGD-MID-AC) in F98 rat glioma models. F98 glioma cells exposed to BPA, cRGD-MID-AC, and cRGD+MID were used for cellular uptake and neutron irradiation experiment. F98 rat glioma models were used for the biodistribution and neutron irradiation experiment after BPA or cRGD-MID-AC administration. In Vitro experiments suggested that BNCT using cRGD-MID-AC has the sufficient cell-killing effect the same as BNCT using BPA. In the biodistribution experiment, cRGD-MID-AC accumulated in the brain tumor, with the highest boron concentration observed 8 hours after administration. In Vivo neutron irradiation experiment suggested that statistically significant differences were observed between Untreated group and BNCT using cRGD-MID-AC groups by the log-rank test. Moreover, the long-term survivors, which were not observed in BNCT using BPA groups, were observed only in BNCT using cRGD-MID-AC after 8 hours after intravenous administration. These fundings suggest that cRGD-MID-AC provides highly selective BNCT against gliomas through a mechanism different from that of BPA.

### O 166 - Systems Medicine on BNCT: New perspectives for biomarker discovery and molecular mechanisms through clinical proteomics approach

#### Pierluigi Mauri^1^, Perico Davide^1^, Ying Tong^2^, Lichao Chen^2^, Dario Di Silvestre^1^, Shoji Imamichi^3^, Yu Sanada^4^, Antonella De Palma^1^, Wolfgang Sauerwein^5^, Mitsuko Masutani^6^

##### ^1^Institute of Technologies for Biomedical, Proteomics and Metabolomics, Segrate - Milan, Italy ^2^Nagasaki University Graduate School, Department of Molecular and Genomic Biomedicine- CBMM, Nagasaki, Japan ^3^National Cancer Center- Tokyo, Central Radioisotope Division- Research Institute- & Division Boron Neutron Capture Therapy- EPOC, Tokyo, Japan ^4^Kyoto University, Institute for Integrated Radiation and Nuclear Science, Kumatori, Japan ^5^DGBNCT, Deutsche Gesellschaft für Bor-Neutroneneinfangtherapie, Essen, Germany ^6^University of Nagasaki, Department of Molecular and Genomic Biomedicine- CBMM, Nagasaki, Japan

Boron neutron capture therapy (BNCT) is a non-invasive and selective radiotherapeutic technique using compounds containing the stable boron isotope ^10^B, typically ^10^B-p-boronophenylalanine (BPA). BNCT is based on nuclear reaction between neutrons and boron-10 atoms that are preferentially distributed in cancer cells resulting in the release of alpha-particles and recoiling lithium nuclei with short path lengths that induce DNA damage, leading to cell death. Despite the growing importance of BNCT for the treatment of different tumors such as head and neck cancer and gliomas, few studies are focused on the identification of biomarkers for cellular response to BNCT. In this context, systems biology approach and particularly proteomics, performed with liquid chromatography coupled with mass spectrometry (LC-MS), provide a powerful tool to investigate BNCT effect at molecular level. To investigate cellular responses to both the boron-containing compound and neutron irradiation, we performed proteomic analysis of the extracellular vesicles (EVs) of oral squamous carcinoma SAS cells treated with or without BPA and at different time points and irradiation doses. The data obtained allowed the stratification between BPA- and BPA+ conditions and different radiation doses. Identification of EV-derived differentially expressed proteins and related signaling pathways, such as apoptosis, DNA repair, and inflammatory response, was possible and in good agreement with publications on cellular profile analysis (Sato et al., 2015; Imamichi et al., 2021). Our results confirm the usefulness of proteomics investigation in BNCT and the potential of systems medicine approach, particularly using proteomics, to improve the identification of biomarkers and mechanisms associated with BNCT.

### O 167 - Tumor response to BNCT and survival outcomes with two boronophenylalanine (BPA) infusion schedules in recurrent head-and-neck squamous cell carcinoma

#### Hanna Koivunoro^1,2^, Ling-Wei Wang^3^, Heikki Joensuu^2^

##### ^1^Neutron Therapeutics Finland Oy, Medical Physics, Helsinki, Finland ^2^Helsinki University Hospital and University of Helsinki, Department of Oncology, Helsinki, Finland ^3^Taipei Veterans General Hospital, Department of Oncology, Taipei, Taiwan Province of China

In boron neutron capture therapy (BNCT) high-LET radiotherapy is achieved by combining external irradiation with low energetic neutrons and a carrier drug that transports ^10^B selectively into the cancer cells. Single-agent L-boronophenylalanine (L-BPA) has been the most frequently used boron carrier compound. L-BPA has been administrated as intravenous infusion using two different infusion schemes: the L-BPA infusion is either stopped 1 to 2 hours prior to starting neutron irradiation or L-BPA is infused until neutron irradiation and continued during neutron irradiation, but at half of the pre-irradiation infusion rate. In this retrospective study we evaluate patient outcomes after BNCT given with either “the stop scheme” or “the continued L-BPA infusion scheme” in a patient population with inoperable recurrent squamous cell carcinoma of head and neck. Sixty-nine of the patients received neutron irradiation at the FiR 1 research reactor site (Finland), where neutron irradiation was started about 90 minutes after the end of the L-BPA infusion. The L-BPA dose was 400 mg/kg given in 2 hours (rate 200 mg/kg/h). Thirty patients were treated at the THOR research reactor (Taiwan), where the cumulative L-BPA dose was the same (400 mg/kg), but the L-BPA infusion rate was slightly slower (180 mg/kg/h), administered over 2 hours before neutron irradiation and continued at the rate of 1.5 mg/kg/min concurrently with neutron irradiation. Results of tumor response to BNCT, treatment safety, and patient survival will be presented.

### O 168 - Boron Neutron Capture Therapy (BNCT) combined with the prebiotic Oligo-Fucoidan: Effect on microbiota in an experimental oral cancer model

#### Mónica A. Palmieri^1^, Sergio I. Nemirovsky^2^, Ignacio E. Czornenki^3^, Jessica A. Goldfinger^3^, Paula S. Ramos^3^, Emiliano C.C. Pozzi^4^, Silvia Thorp^4^, Paula Curotto^4^, Verónica A. Trivillin^3^, Marcela A. Garabalino^3^, Vanina A. Medina^5^, Cristina Costa^3^, Amanda E. Schwint^6^, Magdalena Pezzoni^6^, Andrea Monti Hughes^6^

##### ^1^Facultad de Ciencias Exactas y Naturales- Universidad de Buenos Aires, Departamento de Biodiversidad y Biología Experimental, Ciudad Autónoma de Buenos Aires, Argentina ^2^Consejo Nacional de Investigaciones Científicas y Técnicas CONICET- Facultad de Ciencias Exactas y Naturales- Universidad de Buenos Aires IQUIBICEN- CONICET-UBA, Instituto de Química Biológica, Ciudad Autónoma de Buenos Aires, Argentina ^3^Comisión Nacional de Energía Atómica CNEA, Departamento de Radiobiología, San Martín- Buenos Aires, Argentina ^4^Comisión Nacional de Energía Atómica CNEA, Departamento de Reactores de Investigación y Producción, Ezeiza- Buenos Aires, Argentina ^5^Consejo Nacional de Investigaciones Científicas y Técnicas CONICET- Universidad Católica Argentina BIOMED- UCA-CONICET, Instituto de Investigaciones Biomédicas, Ciudad Autónoma de Buenos Aires, Argentina ^6^Consejo Nacional de Investigaciones Científicas y Técnicas CONICET- Comisión Nacional de Energía Atómica CNEA, Departamento de Radiobiología, San Martín- Buenos Aires, Argentina

Oral microbiota is a heterogeneous group of microbial species colonizing the surfaces of the oral cavity. Microbiota could affect cancer therapy outcomes, including toxicity. Boron Neutron Capture Therapy (BNCT) is a particle radiotherapy, based on the administration of boron carriers incorporated preferentially by tumour cells, followed by neutron irradiation. BNCT clinical results for Head and Neck cancer have shown significant therapeutic efficacy. However, mucositis is a side effect that limits the dose administered to tumor. Dysbiosis is characterized by loss of beneficial microbes and expansion of pathogenic microbes, and might be linked to mucositis and tumorigenesis. It can be mitigated by the use of prebiotics, biological nutrients that are degraded by microbiota. Oligo-Fucoidan, a sulfated polysaccharide isolated from *Laminaria* japonica brown seaweed, stimulates beneficial bacteria in the gut. Our group showed, in the hamster cheek pouch oral cancer model, an enhancement in tumor control from 67% for BNCT to 94% for BNCT combined with Oligo-Fucoidan. In the present study, taxonomy-based analysis at phyla level showed that microbiota composition and proportion changed during cancerization in the hamster cheek pouch. Particularly, at 12 weeks after cancerization, we detected the Fusobacteria class in tumors and in the precancerous tissue surrounding tumors, the dose-limiting tissue for BNCT. Fusobacteria might be linked to oral squamous cell carcinoma progression. Ongoing studies are focused on the effect of Oligo-Fucoidan, BNCT and BNCT+Oligo-Fucoidan on microbiota in this experimental model. These results would explain the enhancement of BNCT tumor control when combined with Oligo-Fucoidan. Acknowledgments Hi-Q Marine Biotech (Taiwan).

### O 169 - Prospects of dose and LET measurements in heavy ion beams with point-like OSL detectors

#### Jeppe Brage Christensen^1^, Iván Domingo Muñoz^2,3,4^, Christina Stengl^3,4,5^, Michele Togno^6^, Sairos Safai^6^, Lily Bossin^1^, Oliver Jäkel^3,4,7^, Eduardo Gardenali Yukihara^1^

##### ^1^Paul Scherrer Institute, Department of Radiation Safety and Security, Villligen PSI, Switzerland ^2^University of Heidelberg, Department of Physics and Astronomy, Heidelberg, Germany ^3^National Center for Radiation Research in Oncology, Heidelberg Institute for Radiation Oncology HIRO, Heidelberg, Germany ^4^German Cancer Research Center DKFZ, Division of Medical Physics in Radiation Oncology, Heidelberg, Germany ^5^University of Heidelberg, Faculty of Medicine, Heidelberg, Germany ^6^Paul Scherrer Institute, Center for Proton Therapy, Villligen PSI, Switzerland ^7^Heidelberg Ion-Beam Therapy Center HIT, Department of Radiation Oncology, Heidelberg, Germany

The objective of this study is to investigate the applicability of Al2O3:C optically stimulated luminescence detectors (OSLDs) to simultaneously measure the dose and LET in H-1, He-4, C-12, and O-16 beams for clinically relevant fields. This information is useful for both treatment planning verification and to correct the OSLD response for ionization quenching. New experimental data on Al2O3:C OSLD efficiency was obtained at HIT and PSI for doses where the track overlap cannot be neglected. The detector efficiency is affected by the track overlaps, and this effect is for the first time modeled using compound Poisson processes (CPP) applied to target theory to introduce detector response corrections. For doses, where the track overlap can be neglected, the results demonstrate that the OSLDs are suitable for dose and LET measurements in H-1, He-4, and C-12 beams without the CPP corrections, except for O-16.The LET measurements are in agreement with the CPP model for H-1, He-4, and C-12 ions up to 25 keV/μm. Using the CPP model, characteristic dose thresholds are calculated for each ion as a function of energy, above which the response corrections are required. The OSL detectors can readily be applied to simultaneously measure dose and LET for doses below ∼1 Gy. For higher doses, or mixed particle fields, corrections derived from the CPP model must be applied. The OSL detectors are suitable to map the LET and dose in points of interest in experimental setups, involving H-1, He-4, and C-12 ions, with energies suitable for the treatment of deep seated tumors.

### O 170 - Experimental determination of kQ factors in scanned proton beams using a primary-standard level graphite calorimeter

#### Ana Lourenco^1,2^, Nigel Lee^1^, Michele Togno^3^, David Shipley^1^, Sam Flynn^1^, John Cotterill^1^, Sairos Safai^3^, Russell Thomas^1,4^, Hugo Palmans^1,5^

##### ^1^National Physical Laboratory, Medical Radiation Science, Teddington, United Kingdom ^2^University College London, Department of Medical Physics and Biomedical Engineering, London, United Kingdom ^3^Paul Scherrer Institute, Center for Proton Therapy, Villigen, Switzerland ^4^University of Surrey, Faculty of Engineering and Physical Science, Guildford, United Kingdom ^5^MedAustron Ion Therapy Center, Medical Physics Group, Wiener Neustadt, Austria

Beam quality factors, *k_Q_*, were experimentally determined in scanned proton beams for Roos-type ionisation chambers using a primary-standard level graphite calorimeter. Measurements were performed at the Paul Scherrer Institute, Switzerland, using single-energy layers with field sizes of 10x10 cm^2^, and equal number of MUs delivered at each spot, for 5 representative energies (ranging from 70 to 230 MeV) at a water-equivalent depth of 2 gcm^−2^. Calorimeter data were analysed using a range of drift curve time windows because, due to the delivery nature of scanned layers, the beam on/beam off points did not coincide with the visible signal in the calorimeter. The provisional *k_Q_* values determined here were compared with those from IAEA TRS-398 and with values from the literature obtained with Monte Carlo simulations as well as determined from previous graphite calorimetric measurements performed in the middle of the Spread-Out Bragg Peak (SOBP) at different proton facilities. Although the selected drift curve time window has a non-negligible impact, all results agreed within uncertainties. An analytical model of the scanning pattern is being developed to inform on the optimal drift curve time window.

### O 171 - Opportunity of MOSkin for dosimetry of proton therapy on breast cancer

#### Fang-Yi Su^1^, Vladimir Pan^1^, Nicolas Depauw^2^, Dean Cutajar^1^, Linh T. Tran^1^, Marco Petasecca^1^, Michael L.F. Lerch^1^, Anatoly Rosenfeld^1^

##### ^1^University of Wollongong, Centre for Medical Radiation Physics, Wollongong, Australia ^2^Francis H. Burr Proton Therapy Centre- Massachusetts General Hospital, Department of Radiation Oncology, Boston- Massachusetts, USA

Postmastectomy radiation therapy, using proton pencil beam scanning (PBS) with intensity modulation, irradiates the target on the chest wall and nodal regions yet reduces dose to the cardiac structures. As the treatment planning system does not necessary predict the dose built-up on the skin, a reliable and fast *in vivo* dosimetry is required. The aim of this study is to investigate the application of MOSkin dosimeters for skin dosimetry in PBS. We studied the linearity of MOSkin response in the middle of 6 cm SOBP with a 2 GyRBE delivered to the solid water phantom. The skin dose was measured in the similar field with MOSkin and Markus parallel ionization chamber. The PBS (energies 222-189 MeV) delivered 2 GyRBE to a half-cylindrical phantom with a radius of 12 cm, placed on the MatriXX 2D IC for dose verification. We also investigated the MOSkin LET dependence at different depths along the 223.58 MeV pristine BP. MOSkin had a linear response to proton dose up to 24 Gy, demonstrated a sensitivity of 2.15 mV/cGy. Using this calibration, the skin dose was within 2.75% up to the depth of 5 mm. The ratio of the MOSkin and MatriXX IC response was up to 38% in the BP of 31.5cm depth due to higher recombination of e-h pairs in a proton track with proton LET increasing. MOSkin is suitable for in vivo skin dosimetry during the breast treatment. An optimal procedure for calibration of the MOSkin for the skin dosimetry needs to be studied further.

### O 172 - Spatial mapping of the thermal neutron flux in proton therapy using the miniaturized Timepix3 detector with neutron converter

#### Cristina Oancea^1^, Michal Andrlik^2^, Carlos Granja^1^, Jan Jakubek^1^, Lukas Marek^1^, Jiri Pivec^1^, Jaroslav Solc^3^, Zdenek Vykydal^3^, Vladimir Vondracek^2^, Reinhard Schulte^4^

##### ^1^ADVACAM, Research and Development, Prague, Czech Republic ^2^Proton Therapy Center Czech, Medical Physics, Prague, Czech Republic ^3^Czech Metrology Institute, Metrology Department, Brno, Czech Republic ^4^Loma Linda University, Division of Bioengineering Sciences, California, USA

We present a method for selective detection, imaging, and measurement of the thermal neutron particle component generated during clinical proton pencil beam scanning (PBS). Such characterization is of interest for out-of-field dosimetry and neutron capture-enhanced proton therapy (NCEPT) research [1]. The semiconductor pixel detector Timepix3 was used to measure the flux and spatial distribution of the thermal neutron field produced in a water phantom (Fig. 1a). The detector, equipped with a ^6^LiF neutron converter (Fig. 1b), was prior calibrated in a thermal neutron reference field. Characteristic interactions in the pixel detector by thermal neutrons are discriminated from other scattered radiation by correlated spatial regions and high-resolution pattern recognition analysis of single particle tracks (Fig. 1d, e). Figure 2 displays the thermal neutron flux for a low-dose single spot PBS protons (0.02 MU/spot) at 200 MeV energy, measured at a depth of 15 cm for various lateral positions. The flux initially reduces exponentially but levels off at larger distances. The thermal neutron field in the mixed-radiation stray fields of PBS proton therapy can be measured with high discrimination by the Timepix3 detector. Such detailed spatial mapping of thermal neutrons serves out-of-field dosimetry for research in NCEPT.

### O 173 - FLASH range QA measurements with the Quality Assurance Range Calorimeter (QuARC)

#### Saad Shaikh^1^, Sonia Escribano-Rodriguez^1^, Raffaella Radogna^2^, Connor Godden^1^, Derek Attree^1^, Samuel Manger^3,4^, Karen Kirkby^3,4^, Marc-Jan Van Goethem^5^, Alexander Gerbershagen^5^, Simon Jolly^1^

##### ^1^University College London, Physics & Astronomy, London, United Kingdom ^2^University of Bari, Physics, Bari, Italy ^3^The University of Manchester, Division of Cancer Sciences- School of Medical Sciences- Faculty of Biology- Medicine and Health, Manchester, United Kingdom ^4^The Christie NHS Foundation Trust, Manchester Academic Health Science Centre, Manchester, United Kingdom ^5^Particle Therapy Research Center PARTREC, Department of Radiation Oncology- University Medical Center Groningen, Groningen, Netherlands

To ensure optimal patient safety with Proton Beam Therapy (PBT), several beam properties are measured as part of Quality Assurance (QA), with the proton range in water being key a parameter. Due to time restrictions in daily QA, comprehensive measurements are not made. Among many other technical challenges in realising FLASH PBT, current methods for range QA (namely ionisation chamber-based dosimetry) become unusable at the required high dose rates. The QuARC is a compact detector for proton range measurements under development at UCL. The detector utilises a series of optically isolated scintillator sheets where each is coupled individually to a photodiode in order to sample the proton depth-light distribution. Fitting to an analytical depth-light model, the original depth-dose curve is reconstructed and the proton range is measured in real-time to sub-mm precision, without any optical artefacts. Due to the nanosecond decay time of the plastic scintillator and the large dynamic range of the detector, range measurements are also possible at FLASH dose rates. Presented are FLASH range QA measurements made with the QuARC at The Christie in Manchester, UK and UMCG PARTREC in Groningen, Netherlands at beam intensities up to 50 nA. These show excellent agreement with clinical current depth-light measurements made between 1–10 nA, all while providing real-time water-equivalent ranges accurate to 0.5 mm from 70–245 MeV. The measurements demonstrate linear scaling of scintillator light output with beam current, thus providing promising results for an integrated QA solution for both existing clinical and FLASH PBT.

### O 174 - First patient measurements of carbon-ion therapy monitoring with charged nuclear fragments at the Heidelberg Ion Beam Therapy Center

#### Laurent Kelleter^1,2,3^, Pamela Ochoa Parra^1,3,4^, Luisa Schweins^1,3,4^, Rebekka Kirchgaessner^1,3,5^, Stefan Schmidt^1,3,6^, Renato Felix Bautista^1,3^, Semi Harrabi^7,8^, Oliver Jaekel^1,2,3,7,8^, Juergen Debus^1,2,3,4,7,8^, Maria Martisikova^1,2,3^

##### ^1^German Cancer Research Centre DKFZ, Medical Physics in Radiation Oncology, Heidelberg, Germany ^2^NCT, National Center for Tumor diseases, Heidelberg, Germany ^3^National Centre for Research in Radiation Oncology NCRO, Heidelberg Institute for Radiation Oncology HIRO, Heidelberg, Germany ^4^Heidelberg University, Department of Physics and Astronomy, Heidelberg, Germany ^5^Karlsruhe Institute of Technology KIT, Department of Physics, Heidelberg, Germany ^6^Tübingen University, Medical faculty, Tübingen, Germany ^7^Heidelberg University Hospital, Heidelberg Ion Beam Therapy Center, Heidelberg, Germany ^8^Heidelberg University Hospital, Department of Radiation Oncology, Heidelberg, Germany

Interfractional morphological changes in the patient anatomy pose a challenge to the dose conformity in carbon-ion radiotherapy. Tracking of charged nuclear fragments that are emitted from the patient could allow in-vivo treatment monitoring. A detection system based on 28 hybrid silicon pixel sensors (Timepix3 technology developed at CERN) was developed at the German Cancer Research Center (DKFZ). Details of the design, readout and the data analysis strategy are discussed in this contribution. The detection system was initially characterised in realistic irradiations of anthropomorphic head phantoms. Moreover, the results of the first patient measurements within the ongoing In-Vivo Monitoring (InViMo) clinical trial at the Heidelberg Ion Beam Therapy Center (HIT) are presented. The trial focuses on tumours in the head-and-neck region. The treatment monitoring performance is evaluated using comparisons to CT images, which represent the ground truth of the patient anatomy.

### O 175 - Validation of the Quantum physics processes underlying the integrated optimization of proton FLASH radiotherapy

#### Nathan Harrison^1^, Serdar Charyyev^2^, Cristina Oancea^3^, Alexander Stanforth^1^, Shuang Zhou^4^, William Dynan^1^, Tiezhi Zhang^4^, Steven Biegalski^5^, Liyong Lin^1^

##### ^1^Emory University, Radiation Oncology, Atlanta, USA ^2^Stanford University, Radiation Oncology, Stanford, USA ^3^Advacam, Research, Prague, Czech Republic ^4^Washington University, Radiation Oncology, St Louis, USA ^5^Georgia Tech, Mechanical Engineering, Atlanta, USA

**Introduction**: FLASH is a new treatment modality that requires integrated optimization of dose, instantaneous dose rate (IDR), and linear energy transfer (LET). As FLASH comes into clinical use, methods for ultra-fast measurement of these quantities will be indispensable.

**Methods**: Dose, IDR, and LET were measured using a FLASH proton beam with a nominal energy of 250 MeV, modulated by a 3D-printed ridge filter assembly (Fig.1A). Absolute dose was measured using a commercial array detector and a novel 4D multi-layer strip ionization chamber (MLSIC), which simultaneously measures IDR (Fig.1B). Further timing data was measured beyond the Bragg peak by detecting prompt gamma rays using a semiconductor pixelated detector, Minipix Timepix3. LET measurements were carried out in-beam using an Advapix Timepix3 detector that has 256x256 pixels of 55x55 μm resolution (Fig.1C). To characterize the quantum uncertainty of modulated proton beam energy, we developed an “under-sample-and-recover” technique for detecting individual protons within a high flux primary beam, which facilitated accurate measurement of LET spectra (Fig.2A).

**Results**: Simulations agreed with measurement, with a typical gamma passing rate for absolute dose of > 95% (3 mm/3% criteria) (Fig.2B). Likewise, simulations agreed with measurements of IDR (Fig.2C) and LET (Fig.2D), with averaged IDR values agreeing within 0.3% with instantaneous fluctuations of ∼10%, and LET distributions overlapping by over 90% and showing an increase in high LET components (> 4 keV/um) with increasing depth.

**Conclusion**: Novel QA methods of measuring quantum physics uncertainties in time (IDR) and energy (LET) underlying the FLASH and LET optimizations are validated.

### O 176 - Performance assessment of a deterministic dose algorithm for quality assurance in online adaptive proton therapy in heterogeneous patient geometries

#### Tiberiu Burlacu^1^, Danny Lathouwers^1^, Zoltan Perko^1^

##### ^1^Applied Science/Technische Universiteit Delft, Radiation Science & Technology, Delft, Netherlands

**Purpose/objective**: Showcasing a semi-analytic, deterministic, physics-based and adjoint capable proton dose algorithm in heterogeneous patient geometries used for head & neck (HN) patient-specific quality assurance (PSQA) purposes in online adaptive proton therapy.

**Methods**: We build on our previous physics-based dose calculation work and approximate the Linear Boltzmann Equation into two partial differential equations (PDEs): the one-dimensional Fokker-Planck (1DFP) and the Fermi-Eyges (FE) equations. The 1DFP is numerically solved via discontinuous Galerkin and Runge-Kutta methods. Using the 1DFP solution, the analytical Gaussian FE solution’s depth-dependent coefficients are computed, yielding 3D dose distributions from individual pencil beams. Lateral heterogeneities are included by an optimized beam-splitting scheme, where beamlets of increasing numbers are placed on concentric rings, with constant weight and spread per ring. The CT resolution bounds the ring radii and beamlet spreads. Our PDE-based approach enables applying adjoint theory to cheaply compute dosimetric changes due to perturbations in both anatomy and treatment plan – without performing costly re-computations – thereby being promising for PSQA.

**Results**: Sub-second beamlet execution times (0.3 s) were achieved. The energy deposited without nuclear interactions in a HN, prostate and lung CT versus TOPAS Monte Carlo reference is shown in Figure 1. Table 1 presents gamma index passing rates. The chosen energies and angles result in high heterogeneity and do not represent clinical choices.

**Conclusion**: Our model provided TOPAS-like performance for the HN (99%, 1mm/1%/10% dose-cutoff) and prostate cases (99.7%). Coupling the short beamlet execution time with the application of adjoint theory is likely to provide a fast and independent PSQA tool.

### O 177 - Clinical outcomes of major salivary gland tumors treated with proton and carbon‐ion radiation therapy

#### Qingting Huang^1^, Weixu Hu^1^, Jiyi Hu^1^, Jing Gao^1^, Jing Yang^1^, Xianxin Qiu^1^, Haojiong Zhang^1^, Jiade Lu^2^, Lin Kong^1,3^

##### ^1^Shanghai Proton and Heavy Ion Center SPHIC, Radiotherapy and oncology, Shanghai, China ^2^Heyou International Hospital, Department of radiotherapy, Foshan, China ^3^Fudan Universiy Shanghai Cancer Center, Radiotherapy and oncology, Shanghai, China

**Background**: Primary major salivary gland carcinomas (SGCs) present with diverse histological types that are known to be largely radioresistant. In view of the physical and biological advantages of proton and/or carbon-ion radiation therapy, we aimed to evaluate the short-term therapeutic effect and toxicities in patients with major SGCs treated with this form of radiation therapy.

**Methods**: Between August 2015 and March 2022, a total of 103 consecutive and non-selected major SGC patients who received particle RT at the Shanghai Proton and Heavy Ion Center (SPHIC) were retrospectively analyzed. The 3-year overall survival (OS), progression-free survival (PFS), local-regional control (LC), and distant metastasis-free survival (DMFS) rates, as well as prognostic factors were analyzed. Additionally, acute and late toxicities were also analyzed.

**Results**: With a median follow-up time of 32 (range, 3–73) months, the 3-year OS, PFS, LC, and DMFS rates were 89.5%, 77.5%, 91.8%, and 82.7%, respectively. At the time of analysis, six patients had developed local or regional recurrence, and fourteen additional patients had developed DM. Six patients had died due to disease progression, one patient with recurrence experienced a late Grade 5 hemorrhage at 9 months after re-irradiation with carbon ion and subsequently died, and one patient died of myocardial infarction. Otherwise, none of the patients had grade 3 or higher treatment-induced acute or late adverse effects except one who developed grade 3 acute mucositis.

**Conclusions**: Overall, intensity-modulated proton and/or carbon-ion radiation therapy provided satisfactory therapeutic effectiveness in our major SGCs patients with a low incidence of acute and late toxicities.

### O 178 - Proton radiation therapy with carbon ion boost in patients with nasopharyngeal carcinoma

#### Lin Kong^1,2^, Jiyi Hu^1^, Qingting Huang^1^, Weixu Hu^1^, Jing Gao^1^, Jing Yang^1^, Xianxin Qiu^1^, Haojiong Zhang^1^, Jiade Lu^3^

##### ^1^Shanghai Proton and Heavy Ion Center, Radiation Oncology, Shanghai, China ^2^Fudan Universiy Shanghai Cancer Center, Radiotherapy and oncology, Shanghai, China ^3^Heyou International Hospital, Radiotherapy and oncology, Foshan, China

**Background**: The outcome of nasopharyngeal carcinoma (NPC) is significantly improved after the prevailing use of chemoradiotherapy. Because of its physical advantage, proton radiation therapy [PRT] and carbon ion radiation therapy [CIRT]) could yield more conformal dose distribution, thus reducing the treatment-related toxicities, while providing comparative disease control. The current study is to evaluate the disease control and toxicity profile of PRT plus CIRT boost in NPC.

**Methods**: Consecutive patients with non-metastatic NPC treated with PRT plus CIRT boost between December 2016 and October 2021 were included. Overall survival (OS), cumulative incidences of local, regional, and distant failure were calculated. Toxicities were graded using the CTCAE (version 4.03).

**Results**: A total of 140 patients were included in the analysis. Among those, 112 patients had locoregionally advanced (III/IV) stages. All patients received 56 Gy (RBE) in 28 fractions, with a median CIRT boost dose of 17.5 (15-17.5) Gy (RBE). After a median follow-up time of 26.1 (2.8-70.3) months, 3 patients died, and 6, 5, and 6 patients developed local, regional and distant failure, respectively. The 3-year OS rate was 96.7% (Fig.1). The cumulative incidences of local, regional, and distant failure were 9.8%, 3.7%, and 7.6%, respectively (Fig.2). Acute toxicity was mild, and severe non-hematological toxicities were observed in only 2 patients (grade 3 oral mucositis and dermatitis). No grade ≥3 late toxicities were observed.

**Conclusion**: Proton radiation therapy with carbon ion boost could provide satisfactory disease control and mild toxicity profile for patients with NPC. Further follow up is necessary to assess the long-term outcome.

### O 179 - Head and neck adenoid cystic carcinoma of the minor salivary glands treated with carbon ion therapy at CNAO

#### Maria Bonora^1^, Barbara Vischioni^1^, Sara Ronchi^1^, Rossana Ingargiola^1^, Anna Maria Camarda^1^, Silvia Molinelli^1^, Sara Imparato^1^, Tiziana Rancati^2^, Francesco Fiorino^3^, Ester Orlandi^1^

##### ^1^Centro Nazionale Adroterapia Oncologica, Area Clinica, Pavia, Italy ^2^Fondazione IRCCS Istituto Nazionale dei Tumori, Prostate Cancer Program, Milano, Italy ^3^Universita’ Milano Bicocca, Universita’ Milano Bicocca, Milano, Italy

**Aim**: To report outcome of minor salivary glands adenoid cystic carcinoma (ACC) patients (pts) treated with curative carbon ion therapy (CIRT) at the National Center for Oncological Hadrontherapy (CNAO).

**Methods**: Between March 2013 and July 2020, 112 ACC patients (M/F = 55/57), mainly located at the paranasal sinuses, were treated with CIRT. Fifty-two pts received definitive treatment, 60 postoperative CIRT; of these 60% had positive margins (R1), and 40% R2. Ninety-two% of the pts had macroscopic GTV detected at pre-CIRT MRI. Prescribed dose was 65.6-68.8 Gy(RBE) in 16 fractions, 4 fr/week. The Kaplan–Meier method was used for local relapse free survival (LRFS), progression free survival (PFS), overall survival (OS), and distant metastasis free survival (DMFS) curves, compared with the Log-rank test. The Chi-square test assessed the association between variables.

**Results**: With a median follow-up of 45 months (8-90), 2 years- LRFS, OS, PFS and DMFS were 82.4%, 92.4%, 70.3% and 85.6%, respectively. At univariate analysis, prognostic factors for both LPFS and OS were tumor site (p=0.006; 0.023) and stage (p=0.012 0.003). For OS, age (p=0.029), GTV (p=0.002) and higher toxicity during follow-up (p=0.002) were additional prognostic factors, although not significant at multivariate analysis. OS decreased in pts after de-bulking (38.3%, p=0.014) compared to macroscopically resected patients (85.2%, p=0.014). Toxicity, evaluated according to CTCAE-v.4.0, is reported in table 1.

**Conclusions**: CNAO data for ACC are in line with CIRT facilities. CIRT might be offered as an alternative curative option to surgery in locally advanced cases, deemed to be R2.

### O 180 - Eye-sparing treatment approach for orbital malignant tumors by using particle radiotherapy

#### Weixu Hu^1^, Lin Kong^1^, Jiyi Hu^1^, Qingting Huang^1^, Jing Gao^1^, Jing Yang^1^, Xianxin Qiu^1^, Haojiong Zhang^1^

##### ^1^Shanghai Proton and Heavy Ion Center, Radiation Oncology, Shanghai, China

**Background**: Eye-sparing treatment is becoming increasingly favored for orbital tumors. The purpose of this study is to evaluate clinical outcomes of proton and/or carbon ion radiotherapy for orbital malignant tumors after eye sparing treatment.

**Methods and Materials**: Between May 2015 and Augest 2021, 61 patients underwent particle radiotherapy were analyzed. The LRFS, DMFS, PFS and DSS rates were calculated using Kaplan-Meier method. Toxicities were scored using the CTCAE 5.0.

**Results**: Fifty-six patients (91.8%) received carbon ion radiotherapy (CIRT) only, 4 patients treated with mixed proton and CIRT, 1 patient with proton. 32.6% of the patients had locally advanced (T3/4) disease. Most patients (59%) presented with lacrimal gland tumor. 39 patients (63.9%) received R2 resection or biopsy. With median follow-up of 32.7 months. The 4-year DSS, LRFS, DMFS and PFS rates were 87.6%, 69.8%, 73.7%, and 53.4%. In lacrimal gland tumors, univariate analyses using Cox regression revealed that patients with large tumor (diameter≥ 3cm) or orbital bone invasion had significantly higher local and distant failure rates. Moreover, locally advanced disease or extraorbital invasion not only had a higher DM rate, also had a poorer DSS. A total of 12 patients (19.7%) experienced visual decrease. Five of them developed grade 1-2 and 7 patient had grade 3-4 visual decrease. None of the patients experienced ophthalmectomy due to ocular complications. Two patients developed grade 1 brain injury.

**Conclusion**: Particle radiotherapy appeared to be a promising eye-sparing treatment approach and produced favorable outcomes. Further follow-up is necessary to evaluate long-term survivals and late toxicity profile.

### O 181 - Secondary cancer risk estimation in patients receiving proton therapy with a new radiobiological model based on the MKM

#### Andrea Attili^1^, Emanuele Scifoni^2^, Carlo Algranati^3^, Marco Cianchetti^3^, Paolo Farace^3^, Francesco Fracchiolla^3^, Stefano Lorentini^3^, Barbara Rombi^3^, Daniele Scartoni^3^, Francesco Tommasino^4^

##### ^1^Italian National Institute for Nuclear Physics INFN, Roma 3, Rome, Italy ^2^Italian National Institute for Nuclear Physics INFN, Trento Institute for Fundamental Physics and Applications TIFPA, Trento, Italy ^3^Azienda Provinciale per i Servizi Sanitari APSS, Protontherapy, Trento, Italy ^4^University of Trento, Department of Physics, Povo- Trento, Italy

**Introduction**: To quantify secondary cancer risk (SCR) in proton therapy (PT) beyond a pure dosimetric assessment, we present an analysis of selected patient cases applying a dedicated adaptation [1] of the microdosimetric kinetic model (MKM), accounting for protons’ variable relative biological effectiveness for such endpoint (SCR-RBE).

**Methods**: The MKM is adapted to evaluate both cell killing and mutation induction, to be intended here as the microscopic manifestation of a macroscopic tumor, after irradiation with a specific radiation quality. Treatment plans are recalculated with a validated Monte Carlo code (TOPAS), including the protons’ energy spectra for each voxel. Combining this information with the proposed MKM formalism, we evaluated the excess absolute risk (EAR) of cancer induction in organs of interest, assuming both constant and variable RBE.

**Results**: We present results obtained from the analysis of mediastinal lymphoma patients. Figure 1 refers to a representative female mediastinal lymphoma patient; the EAR was evaluated for breast, lung, esophagus and thyroid, as a function of the CTV dose. Different trends and absolute EAR values are observed for the different organs. The dependence on variable RBE was investigated assuming different radiosensitivities by modulating the alpha/beta ratio for mutation parameters. As a reference, the EAR obtained neglecting the dependence on LET is reported.

**Conclusions**: Our results indicate that SCR-RBE might play a not negligible role in the estimation of SCR in PT. The present analysis is currently being extended to multiple treatment sites.

### O 182 - DNA damage models of different complexity do not predict fundamental differences in damage yields

#### Shannon Thompson^1^, Kevin Prise^1^, Stephen McMahon^1^

##### ^1^Queen’s University Belfast, The Patrick G Johnston Centre for Cancer Research, Belfast, United Kingdom

Computational radiation response models offer a useful tool to explore mechanisms of DNA damage and repair, enabling comparisons of the biological effectiveness of ion exposures. However, there is large design variation amongst Monte Carlo DNA damage models, with increasing levels of biological detail increasing the required computational power. Despite extensive research, the level of model detail needed to accurately reproduce key experimental endpoints remains unclear. This work explores the influence of different damage model assumptions on double strand break (DSB) yield, distribution and complexity for a range of proton exposures. Using TOPAS-nBio, damage models were designed with increasing biological complexity, comparing the inclusion of realistic nuclear geometries and chemistry (Table 1). Despite different underlying assumptions similar trends of DSB yield with linear energy transfer (LET) were found across all models based on published damage parameter estimates (Figure 1). Notably, all models overlapped if a conservative 5% parameter uncertainty is included. These results indicate many differences in DNA damage models do not fundamentally impact the predicted DSB yield in a way which cannot be corrected with appropriate parameter selection. This suggests more complex models are not distinguishable from simpler approaches at the level of DSB yield. However, for a given yield, model differences do impact DSB distribution and complexity, which could affect predicted biological responses. A complete analysis, simulating repair and response for different ion exposures will be conducted to help determine the level of model detail required to reproduce these downstream experimental observations, and identify key data requirements for validation.

### O 183 - Effects of cell-specific radiosensitivity on biological effectiveness for therapeutic helium-, carbon-, oxygen-, and neon-ion beams

#### Takamitsu Masuda^1^, Taku Inaniwa^1^

##### ^1^National Institutes for Quantum Science and Technology, Department of Accelerator and Medical Physics, Chiba, Japan

**Purpose**: Our facility forwards a research project on hypofractionated multi-ion therapy. For starting to utilize helium-, oxygen-, and neon-ion beams based on the clinical experience in carbon-ion therapy, we examined how tumors and normal tissues with different radiosensitivity would respond to each ion.

**Methods**: Using a stochastic microdosimetric kinetic model, a one-field SOBP beam was created with helium, carbon, oxygen, and neon ions to achieve uniform 10% survival of HSG cells, the reference cell line for the Japanese biological dose model, from 90 mm to 150 mm depth. Then, recalculations were conducted by setting different radioresponse cell lines (NB1RGB, MIA Paca-2, SQ20B, and V79) at depths from 90 mm to 150 mm (target region) and from 0 mm to 10 mm (entrance region). For each cell line, the biological dose of each ion beam relative to the carbon-ion beam was evaluated.

**Results**: The helium-ion beam tended to deposit a larger biological dose for radiosensitive cell lines and a smaller biological dose for radioresistant ones in both regions (up to 17.4%). In contrast, the oxygen- and neon-ion beams showed the opposite trend in the entrance region (up to 11.3% for oxygen and 30.6% for neon). However, in the target region, the biological doses were almost the same as the those of carbon-ion beam (within 3.5%) regardless of radiosensitivity.

**Conclusions**: Regarding tumor control, this study suggests that the dose-fractionation protocols established for carbon-ion therapy can be reasonably applied to oxygen- and neon-ion beams, while there may be room for consideration when applied to helium-ion beam.

### O 184 - The invalidity of and alternative to the linear quadratic model as a predictive cell survival model in particle Beam radiotherapy

#### Heng Li ^1^

##### ^1^Johns Hopkins University, Radiation Oncology and Molecular Sciences, Washington DC, USA

**Purpose**: The linear-quadratic (LQ) model has been the dominant tool in modeling of cell survival as a function of dose, and used extensively in RBE modelling of particle beams. However, the applicability of LQ model in particle beam irradiation is questionable. As a second-order polynomial approximation, it suffers from two well-known pitfalls: non-monotonic behavior and poor extrapolation, both were examined in this study.

**Method**: This study examined raw data of 253 sets of photon and 943 sets of the ion beam from cell irradiation to understand how often the LQ model could result in a negative β, which would give unrealistic predictions. Additionally, the predictive performance of the LQ model, the power model, and the linear model was studied using leave one out cross-validation (LOOCV) and 2-fold cross-validation.

**Results**: When fitted to the LQ model, 7.5% of the photon and 29.8% of the ion beam dose response data would result in negative β, compared to 0.77% and 2.0% reported in the literature. LQ model performed poorly in LOOCV compared to the alternative power model, and performed the worst among the three models in 2-fold cross-validation.

**Conclusion**: LQ model leads to unrealistic parameters, which are vastly under-reported in the literature, and performs poorly in standard cross-validation tests. Therefore, the LQ model is not a valid predicative dose-response model for cell survival in particle beam irradiation. Alternative models need to be investigated.

### O 185 - A multi-stage Generalized Stochastic Microdosimetric Model (GSM2) including radiation chemistry as an insight in UHDR biological mechanism

#### Marco Battestini^1,2^, Francesco Giuseppe Cordoni^2,3^, Marta Missiaggia^4^, Andrea Attili^5^, Francesco Tommasino^1,2^, Chiara La Tessa^4^, Martina Fuss^6^, Michael Krämer^6^, Gianmarco Camazzola^6^, Daria Boscolo^6^, Emanuele Scifoni^2^

##### ^1^University of Trento, Department of Physics, Trento, Italy ^2^National Institute for Nuclear Physics, Trento Institute for Fundamental Physics and Applications, Trento, Italy ^3^University of Trento, Department of Civil- Environmental and Mechanical Engineering, Trento, Italy ^4^University of Miami, Radiation Oncology Department, Miami, USA ^5^National Institute for Nuclear Physics, Roma Tre Section, Roma, Italy ^6^GSI Helmholtz Center for Heavy Ions Research, Biophysics Department, Darmstadt, Germany

Ultra-High Dose Rate (UHDR) irradiation allows a larger sparing of normal tissue and unchanged tumor control with respect to conventional delivery. In recent years, a large amount of experimental evidence [1] confirmed this FLASH effect; however, the mechanism remains to date largely unexplained. The Generalized Stochastic Microdosimetric Model (GSM2) [2,3] is a probabilistic model that describes the time-evolution of the DNA damages in a cell nucleus from microdosimetric principles, with the possibility of describing different levels of spatio-temporal stochasticity [4] in the case of protracted irradiation, without considering the Poissonian assumption to treat the number of radiation-induced DNA damage. In order to describe the coupled evolution of DNA damages and fast chemical reaction kinetics [5], we develop a multiscale GSM2 (Figure 1), which takes into account radical recombination, oxygen consumption and re-oxygenation and intertrack effects. We simulate energy deposition by particles in a microscopic volume, in order to examine the combined effects of several chemical species and the time evolution of DNA damages (Figure 2), taking into account several possible effects on DNA damage at UHDR regime (reduction of DNA damage yield, damage fixation due to oxygenation and intertrack effects). We study the impact of any dose delivery time structure and different LET regimes. We show that the multiscale GSM2 can describe the empirical trend of dose and dose rate dependent cell sensitivity over a broad range.

### O 186 - Microdosimetry for treatment planning in proton therapy

#### Pietro Pisciotta^1^, Jacopo Magini^2^, Erik Traneus^3^, Mohammad Hussein^4^, Stefan Both^1^, Alexander Gerbershagen^1^, Giuseppe Schettino^4^, Francesco Romano^1,5^

##### ^1^University Medical Center Groningen‚ Particle Therapy Research Center PARTREC, Department of Radiation Oncology, Groningen, Netherlands ^2^University of Surrey, Department of Physics, Guildford, United Kingdom ^3^RaySearch Laboratories, RaySearch, Stockholm, Sweden ^4^National Physical Laboratory, Medical Radiation Science, Teddington, United Kingdom ^5^Istituto Nazionale di Fisica Nucleare, Catania Division, Catania, Italy

**Purpose**: The use of stochastic measurable microdosimetric quantities can lead to a better prediction of the biological radiation response for densely ionizing radiation. In this work a novel approach is investigated to implement microdosimetric quantities for proton treatment planning by means of look-up-tables (LUTs).

**Materials and Methods**: The LUTs include the microdosimetric means (ȳ_D_, ȳ_F_ and y*) obtained for each sampled energy by simulating with the Geant4 Monte Carlo code a monoenergetic beam shot perpendicularly at a water volume 1um thick and 1mm wide. In RayStation 11B-IonPG a simple setup was made, consisting of a rectangular water phantom into which a 150MeV proton beam was shot. The produced LUTs were used as weights to average the kinetic energy(KE) spectra generated by RayStation to calculate the microdosimetric means for each depth in water. To test the consistency of the adopted approach, these means were then compared to the ones obtained in a full top-to-bottom Geant4 simulation considered as reference, using the same test setup.

**Results**: LUTs have been built for protons for a wide range of KEs (Fig.1). Except for discrepancies in the entrance associated with larger statistical oscillation for high energy protons, the overall agreement in the peak region between the reference Geant4 simulations and RayStation was good, in particular within 10% for y* which is currently used in established radiobiological models (Fig.2).

**Conclusion**: This study showed the feasibility of using LUTs to calculate microdosimetric quantities and represents the first step towards the implementation of biological models based on microdosimetric, experimentally measurable, quantities within TPSs.

### O 187 - CT-based stopping-power ratio prediction using a Hounsfield look-up table: A consensus guide

#### Nils Peters^1,2,3^, Vicki Trier Taasti^3,4^, ESTRO Physics Workshop 2021 subgroup^5^, Patrick Wohlfahrt^2,6^

##### ^1^OncoRay – National Center for Radiation Research in Oncology- Faculty of Medicine and University Hospital Carl Gustav Carus- Technische Universität Dresden, Helmholtz-Zentrum Dresden - Rossendorf, Dresden, Germany ^2^Massachusetts General Hospital and Harvard Medical School, Department of Radiation Oncology, Boston, USA ^3^Shared, first authorship, listed alphabetically, Germany ^4^GROW School for Oncology and Reproduction- Maastricht University Medical Centre+, Department of Radiation Oncology Maastro, Maastricht, Netherlands ^5^On behalf of all workshop subgroup participants and EPTN WP5 members who contributed to the document, created within the ESTRO Physics Workshop 2021 on CT innovations in radiation oncology, ESTRO Physics Workshop 2021, Hungary ^6^now, with Siemens Healthineers, Forchheim, Germany

**Motivation**: Large variations in stopping-power ratio (SPR) prediction from computed tomography (CT) across European proton centres were observed in recent studies. To standardise CT-based SPR prediction using a Hounsfield look-up table (HLUT), a step-by-step consensus guide, created within the ESTRO Physics Workshop 2021 in a joint effort with EPTN-WP5, is presented.

**Methods**: The HLUT specification process includes six steps: Phantom setup, CT acquisition, CT number extraction, SPR determination, HLUT specification, and HLUT validation. Appropriate phantom inserts are tissue-equivalent for both X-ray and proton interactions and are scanned in head- and body-sized phantoms to mimic different beam hardening conditions. Soft tissue inserts can be scanned together, while scanning bone inserts individually reduces imaging artefacts. For optimal HLUT specification, the SPR of phantom inserts is measured and the SPR of tabulated human tissues is computed stoichiometrically. The HLUT stability is increased by including both phantom inserts and tabulated human tissues. Piecewise linear regressions of CT numbers and SPRs are performed for lung, adipose, soft tissue, and bone and then connected. Finally, a thorough validation is performed.

**Results**: The best practices and individual challenges are explained comprehensively for each step. A well-defined strategy for specifying the connection points between the individual line segments of the HLUT is presented. The guide was tested exemplarily on three CT scanners from different vendors, proving its feasibility on both single-energy CT and virtual monoenergetic images from dual-energy CT.

**Conclusion**: The presented step-by-step guide for CT-based HLUT specification with recommendations and examples can increase the clinical range prediction accuracy and reduce its inter-centre variation.

### O 188 - Development of an easy-to-use interplay simulator and its applications

#### Alexander Stanforth^1^, William LePain^1^, Qianxia Wang^1^, Roelf Slopsema^1^, Mingyao Zhu^1^, Haijian Chen^1^, Katja Langen^1^, Stella Flampouri^1^

##### ^1^Emory University, Radiation Oncology, Atlanta, USA

**Introduction**: Interplay in PBS treatments of moving targets affects the received dose distribution. A clinical tool was developed, validated, and deployed to include interplay in plan robustness evaluation. The tool provides both an assessment of individual patient dose degradation, and evaluation of mitigation used.

**Materials and Methods**: We developed a tool within the RayStation scripting module to simulate patient motion based on 4DCTs and incorporate beam delivery properties. 4D and 4D dynamic doses (AAPM-TG290) are calculated based on measured parameters and their statistical fluctuations. Measurements were performed to fine-tune parameters and validate accuracy. Additionally, we used a cohort of previously treated liver and lung patients with significant motion to determine the effects of spot spacing, energy layer spacing, minimum MU/spot and range shifter on plan quality.

**Results**: The validated tool is easy-to-use and provides multiple metrics on plan quality and robustness. In addition to direct or indirect repainting, energy layer spacing was shown to be the most critical variable to ensure adequate target coverage with interplay effects. The other factors had limited impact on plan quality, with their relative importance depending on the properties of the active scanning system and the individual patient characteristics.

**Discussion**: We developed a tool able to simulate interplay effects within a clinical treatment planning system and simplify 4D robustness evaluation. Additionally, this tool facilitated investigation of interplay sensitivity to planning techniques and lead to improved treatment plan resilience.

### O 189 - Partitioning of proton arc plans over fractions can improve delivery time and plan quality

#### Erik Engwall^1^, Otte Marthin^1^, Viktor Wase^1^, Johan Sundström^1^, Hans Langendijk^2^, Bas de Jong^2^, Erik Korevaar^2^, Stefan Both^2^

##### ^1^RaySearch Laboratories AB, Research and Development, Stockholm, Sweden ^2^University Medical Center Groningen, Department of Radiation Oncology, Groningen, Netherlands

**Purpose**: Proton arcs can be grouped into *dynamic arcs*, in which the delivery occurs during rotation, and *discrete arcs*, which can be seen as many IMPT beams delivered in step-and-shoot mode. We present a new method where the plans are partitioned into subplans delivered over alternating fractions, aiming to reduce delivery time and improve interfractional robustness.

**Methods**: We have created full-revolution discrete and dynamic arc plans for an oropharyngeal cancer patient previously treated with five IMPT beams. The discrete arc plans have 360 energy layers over 30 directions: one plan delivers all directions in every fraction (1x30), while two plans are partitioned into subplans with 10 (3x10) and 6 (5x6) directions. The dynamic arcs are planned with 180, 240 or 360 energy layers per fraction. Objectives are used to ensure that the target dose is uniform in every fraction. Target coverage is assessed over the treatment course using accumulated scenario doses on weekly CTs. NTCP values and doses to OARs, as well as delivery times, are also reported.

**Results**: Delivery times can be substantially reduced by an arc partitioning approach, especially for discrete arcs (Figure 1b). Target robustness is improved for the partitioned plans (Figure 2a), while NTCP values and OAR doses are compromised if the total number of directions is kept constant (Figure 2b). If the number of directions is instead increased (cf. 7x180, 7x360), OARs could be spared to a larger extent.

**Conclusions**: Partitioning of proton arcs could play an important role for delivery efficiency and plan quality.

### O 190 - A framework for single-energy proton FLASH planning using pin ridge filter

#### Chaoqiong Ma^1^, Xiaofeng Yang^1^, Chih-Wei Chang^1^, Mark McDonald^1^, Sibo Tian^1^, Jun Zhou^1^

##### ^1^Emory University, Radiation Oncology, Atlanta, USA

**Background**: Proton FLASH radiotherapy using single-energy transmission beams has limitations in normal tissue sparing. We proposed an inverse planning framework to design pin ridge filter (RF) for Bragg peak modulation in single-energy FLASH intensity-modulated proton therapy (IMPT).

**Methods**: A beam model of multiple energies for IMPT planning were commissioned in RayStation by modulating a 250MeV beam with range shifter (RS) of various thicknesses. An in-house inverse planning framework integrated within RayStation was developed to design pin RFs. An IMPT plan (IMPT-RS) was firstly generated using the commissioned beam model, followed by iterative energy layer reduction to push up minimum MU. Then, the energies and corresponding weights at each spot position of the IMPT-RS plan were translated to step thicknesses and widths of each ridge pin, respectively, to design RFs. Finally, an IMPT plan (IMPT-RF) was generated using the 250MeV beams with the designed RFs. This framework was validated for dose and dose rate distributions on a lung case.

**Results**: Comparing to the IMPT-RS plan, the lungs V_7Gy_, heart D_mean_, esophagus D_5cc_ and spinal cord D_0.35cc_ slightly increased by 2%, 0.05Gy, 2.5Gy and 2Gy, respectively, in the IMPT-RF plan. A moderately lower conformity was obtained in the IMPT-RF plan (CI=1.44, vs 1.20 from IMPT-RS plan). The FLASH effect was achieved in the whole CTV and in > 73% of the esophagus/lungs received >10% of the prescription dose.

**Conclusions**: The preliminary results demonstrated that the designed RFs using the proposed method has potential for FLASH IMPT planning. More clinical cases will be needed for further validation.

### O 191 - Carbon ions radiotherapy for unresectable sacral chordoma

#### Agnieszka Chalaszczyk^1^, Maria Rosaria Fiore^1^, Angelica Ghirelli^1^, Marco Rotondi^1^, Silvia Molinelli^1^, Alessandro Vai^1^, Stefania Russo^1^, Matteo Pepa^1^, Andrea Pella^1^, Mario Ciocca^1^, Ester Orlandi^1^

##### ^1^National center of hadrontherapy CNAO, radiotherapy, Pavia, Italy

**Aims**: Preliminary outcome and late toxicity of patients with primary sacral chordoma treated with definitive high-dose carbon ion radiotherapy (CIRT).

**Methods**: Between March 2013 and December 2021, 108 patients were treated after biopsy with definitive CIRT with a total dose of 70.4 - 76.8 Gy (RBE) in 16 fractions. Forty-three patients were enrolled in the international phase III protocol from March 2018. We retrospectively analyzed the outcome of 65 patients. Local control (LC), overall survival (OS) and local progression-free survival (LPFS) has been calculated with Kaplan Meyer method. Secondary endpoint was late toxicity, assessed according to Common Terminology Criteria for Adverse Events (CTCAE) version 5.0. Clinical-radiological evaluation has been performed every four months for two years, afterwards annually.

**Results**: Median follow-up was 47.7 months (range 12 – 115). Twenty-five local progression (38%) were observed, with a median time to local recurrence of 30 months (range, 7-60). Nineteen patients were lost to follow-up after median time of 56 moths (range 12-62). LPFS at 3 and 5 years were 69% and 51%, respectively (Fig.1). OS at 3 and 5 years were 90% and 76%, respectively (Fig.2). Late scored toxicities were G1-G2 neuropathy/skin toxicity in 44 (67%) patients. Grade 3 late toxicity was observed in 7 (10%) patients: 2 peripheral motor neuropathy, 2 neurological bladder, 1 bone fractures and 2 skin ulceration. The remaining 14 patients do not complain symptoms.

**Conclusions**: Definitive high dose CIRT could be a favorable strategy with acceptable toxicity for sacral chordoma where surgery is expected to be disabling.

### O 192 - Outcomes for base of skull chordoma and chondrosarcoma treated using simultaneous integrated boost proton beam therapy

#### Teresa Dinizulu^1^, Anna France^2^, Peter Sitch^3^, Frances Charlwood^3^, Matthew Lowe^3^, Neil Burnet^1^, Matthew Clarke^3^, Rovel Colaco^1^, Shermaine Pan^1^, Gillian Whitfield^1^

##### ^1^The Christie NHS Foundation Trust, Clinical Oncology, Manchester, United Kingdom ^2^The Christie NHS Foundation Trust, Proton Clinical Outcome Unit, Manchester, United Kingdom ^3^The Christie NHS Foundation Trust, Physics, Manchester, United Kingdom

**Aim**: Adjuvant proton therapy for skull base chordoma and chondrosarcoma is conventionally a two-phase approach. We report a single centre experience using a simultaneous integrated boost(SIB) approach.

**Method**: We retrospectively reviewed data of all patients completing treatment January 2019 to February 2022 with SIB plans. SIB plans prescribed 73.8Gy to CTV_High concurrent with 59.45Gy to CTV_Low in 41# for chordoma and 70.2Gy to CTV_High with 58.5Gy to CTV_Low in 39# for chondrosarcoma.

**Results**: Forty-one patients were included, 24 female, 26 chordoma. Median age was 49 years (4-74). Median follow-up was 14.8 months (6-27.4). Progression free survival(PFS) at 1 and 2 years for chondrosarcoma was 92.3% (78-100), and for chordoma, 96.2% (89-100) and 91.4% (80-100). (Figure 1) There was one death, due to pneumonia, in a patient with chondrosarcoma. One patient failed distantly, a chordoma, along the surgical access tract. Three patients progressed locally, all had residual disease. The proportion of GTV_residual receiving ≥66Gy was 100% in two and 98.1% in one. There was no statistically significant difference in mean CTV_High doses and percentage of GTV_residual ≥66Gy based on local progression status. One patient had radionecrosis (Grade 1), in the right medial temporal lobe and superficial right lateral brainstem. Dose to 2% (D2%) of brainstem was 62.6Gy and for right temporal lobe, D2% was 71.9Gy and D2cc 70.5Gy.

**Conclusion**: Follow-up in this cohort is limited. Rates of radionecrosis so far are low. The PFS falls within the expected range, but longer follow-up is necessary to fully interpret the data.

### O 193 - Ten-year outcomes following proton therapy for localized prostate cancer

#### Curtis Bryant^1^, Randall Henderson^1^, Romaine Nichols^1^, William Mendenhall^1^, Perry Johnson^1^, Kethandapatti Balaji^1^, Joseph Costa^1^, Nancy Mendenhall^1^

##### ^1^University of Florida Health, Proton Therapy Institute, Jacksonville, USA

**Purpose/Objective(s)**: To report 10-year biochemical outcomes, physician-reported toxicity, and patient-reported quality of life for patients treated for localized prostate cancer with proton therapy.

**Methods and Materials**: Ten-year outcomes from a prospective outcome tracking protocol were assessed and reported for 1272 men with localized prostate cancer. The median proton therapy dose was 78 CGE (72 to 82 CGE) delivered at 1.8 to 2 CGE per fraction. ADT was received by 193 men. Biochemical control was defined using the Phoenix definition. CTCAE, v5, was used for toxicity scoring and IPSS and EPIC for patient-reported outcomes.

**Results**: The median follow-up was 10.2 years. The 10-year biochemical control for patients with very low risk, low risk, favorable intermediate risk, unfavorable intermediate risk, high risk, and very high-risk prostate cancer were 97%, 96%, 90%, 84%, 71%, and 51%, respectively. On multivariate analysis, PSA, perineural invasion, and irradiation of the seminal vesicles predicted 10-year biochemical control. The 7- and 10-year grade 3+ gastrointestinal and urologic toxicity rates were 1.0% and 1.3%, and 4.0% and 5.9%, respectively. The 7- and 10-year grade 2+ GI toxicity rates were 12.1% and 13.2%, respectively. A clinically significant decline in EPIC sexual summary scores occurred as mean scores were 67 at baseline and fell to 41 at 10 years.

**Conclusion**: These long-term results show that proton therapy can provide favorable biochemical control outcomes for patients with localized prostate cancer. Late grade 3+ toxicity rates are low. There was an expected decline in overall sexual function as the patient cohort aged following proton therapy.

### O 194 - Proton therapy for breast cancer: The role of verification CT during radiotherapy

#### Christina Klassen^1^, Eric Brooks^2^, Raymond Mailhot Vega^2^, Nancy Mendenhall^2^, Julie Bradley^3^

##### ^1^University of Florida, University of Florida, Gainesville- FL, USA ^2^University of Florida, Radiation Oncology, Jacksonville- FL, USA ^3^University of Florida Health Proton Therapy Institute, Radiation Oncology, Jacksonville, USA

**Purpose**: The purpose of this study is to assess the frequency of replanning during a course of proton therapy for breast cancer.

**Methods**: Patients treated with proton therapy for breast cancer between 1/1/2017 and 7/1/2022 were identified from a prospective registry. Patient selection included whole breast or chest wall irradiation +/− regional nodal irradiation and ≥ 1 verification CT (VFCT) during radiotherapy. Clinical and treatment characteristics were extracted from the medical record.

**Results**: One hundred sixty-five patients were identified. 58% patients underwent lumpectomy (n=96) and 42% mastectomy (n=69; Reconstruction with immediate expander in 21, implant in 5 and no reconstruction in 43). Median total radiation dose was 60 (range, 42.4 – 70 GyRBE). Nine patients were treated with DS, 154 with PBS and two treated with a combination of DS and PBS. The use of VFCT was planned for most patients (79%) and obtained prn based on clinical or daily imaging concerns for the remainder (21%). Most patients underwent two VFCTs (range, 1-6). Re-planning was performed 58 times in 52 patients (31.7%). The modified plan started at a median fraction of 14.5 (range, 2-27) and there was a median of 3 business days between the VFCT and start of the new plan. Three patients missed treatment day(s) due to the need for a re-plan.

**Conclusion**: In a cohort of patients undergoing proton therapy for breast cancer, an adaptive plan was utilized in approximately one-third of patients. Attention to changes in breast and chest wall tissue is important for optimal proton therapy delivery.

### O 195 - Partial breast irradiation with proton beam: Ten-year results

#### Jessica Jutzy^1^, Carlos Garberoglio^2^, Sharon Lum^2^, Jerry Slater^1^, David Bush^1^

##### ^1^Loma Linda University, Department of Radiation Medicine, Loma Linda, USA ^2^Loma Linda University, Department of Surgery, Loma Linda, USA

**Purpose**: An updated analysis of a phase 2 trial investigating partial breast irradiation (PBI) using proton beam radiation therapy in patients with early-stage breast cancer.

**Methods and Materials**: Patients with invasive non-lobular breast carcinoma who underwent lumpectomy with negative margins and a maximum tumor dimension of 3 cm were eligible. All patients underwent axillary nodal assessment and were pathologically node negative. Patients received adjuvant PBI using proton beam radiation. Treatment was performed in the prone position using a full body pod with breast cup insert to immobilize the breast. Alignment to surgical clips using kV imaging was performed prior to each treatment. A dose of 40 Gy (RBE) in 10 fractions was delivered once daily over 2 weeks using multiple fields with aperture edits to spare skin. Clinical assessments and mammography were utilized to monitor for recurrence, toxicity, and cosmetic outcome.

**Results**: One hundred patients were enrolled and received protocol specified treatment. At a median follow-up of 153 months, ipsilateral breast tumor recurrence was observed in 11%; contralateral disease, 5%; axillary failure, 2%; and distant failure, 5%. Median disease-free survival was 138.5 months. Ten-year skin toxicity data was available for 22 patients. Two patients developed new late toxicity (grade 1 telangiectasia and grade 1 breast tenderness). No grade 3 or higher acute or late toxicities were present at any time point.

**Conclusions**: Proton partial breast radiotherapy provides good ipsilateral breast tumor control. Utilization of pod immobilization and skin aperture edits results in minimal skin toxicity.

### O 196 - Impact of respiratory motion on proton pencil beam scanning FLASH radiotherapy: An in silico and phantom measurement study

#### Yunjie Yang^1^, Minglei Kang^1^, Sheng Huang^2^, Chin-Cheng Chen^1^, Pingfang Tsai^1^, Francis Yu^1^, Carla Hajj^3^, Wolfgang Tome^4^, Charles Simone^3,5^, Haibo Lin^1,6^

##### ^1^New York Proton Center, Medical Physics, New York, USA ^2^Tianjin Medical University Cancer Institute and Hospital, Department of Radiation Oncology, Tianjin, China ^3^Memorial Sloan Kettering Cancer Center, Department of Radiation Oncology, New York, USA ^4^Montefiore Medical Center and Albert Einstein College of Medicine, Department of Radiation Oncology, Bronx, USA ^5^New York Proton Center, Radiation Oncology, New York, USA ^6^Memorial Sloan Kettering Cancer Center, Department of Medical Physics, New York, USA

**Purpose**: To investigate the effects of respiratory motion on the delivered dose in the context of proton pencil beam scanning (PBS) transmission FLASH-RT by simulation and phantom measurements.

**Methods**: An in-house simulation code was employed to perform *in silico* simulation of 2D dose distributions to investigate the impacts of various respiratory motion and treatment delivery parameters on the dynamic proton PBS transmission FLASH-RT dose delivery. A strip-ionization chamber array detector and an IROC motion platform were employed to perform phantom measurements of the 2D dose distribution for treatment fields similar to those used for simulation.

**Results**: Clinically relevant respiratory motion and treatment delivery parameters resulted in degradation of the delivered dose compared to the static delivery. Simulation showed that the gamma passing rates (2 mm/2%) and target coverage (V100%) could drop below 50% and 80%, respectively, for certain scenarios if no mitigation strategy was used. The gamma passing rates and target coverage could be restored to more than 95% and 98%, respectively, for short beams delivered at the maximal inhalation or exhalation phase. The simulation results were qualitatively confirmed in phantom measurements with the motion platform.

**Conclusions**: Respiratory motion could cause dose quality degradation in proton PBS transmission FLASH-RT treatments if no mitigation strategy is employed. Besides breath-hold, gated delivery can be an alternative motion management strategy to ensure high consistency of the delivered dose. To the best of our knowledge, this is the first study on motion impacts in the context of proton transmission FLASH radiotherapy.

### O 197 - A novel planning and delivery technology: Dose, dose rate and linear energy transfer (LET) optimization based on spot-scanning proton arc therapy FLASH (SPLASH-_LET_)

#### Gang Liu^1^, Fan Qingkun^2^, zhao Lewei^3^, Li Xiaoqiang^3^, Lu Xiliang^2^, Dai Shuyang^2^, Zhang Sheng^1^, Yang Kunyu^1^, Ding Xuanfeng^3^

##### ^1^Huazhong University of Science and Technology, Cancer Center-Union Hospital-Tongji Medical College, Wuhan, China ^2^Wuhan University, School of Mathematics and Statistics, Wuhan, China ^3^Corewell Health William Beaumont University Hospital, Radiation Oncology, Royal Oak, USA

**Purpose**: To achieve a high conformal dose with Linear Energy Transfer (LET) optimized FLASH proton therapy, we introduced a new planning and delivery technique concept, the voxel-wised optimization of LET distribution and dose rate based on scanning arc therapy (SPLASH_-LET_).

**Method and Materials**: The algorithm optimizes (1) the clinical dose-volume constraint based on dose distribution and (2) the clinical LET-volume constraint based on LET distribution using Alternating Direction Method of Multipliers(ADMM) with Limited-memory BFGS solver by minimizing the monitor unit (MU) constraint on spot weight and (3) the effective dose-average dose rate by minimizing the accelerator’s beam current sequentially. Such optimization framework enables the high dose conformal dynamic arc therapy with the capability of LET painting with voxel-based FLASH dose rate. It aims to minimize the overall cost function value combined with plan quality and voxel-based LET and dose rate constraints. Three representative cases(brain,liver and prostate cancer) were used for testing purposes. Dose-volume histogram (DVH), LET-volume histogram (LVH) dose rate volume histogram (DRVH) and dose rate map were assessed.

**Result**: SPLASH_LET_ could offer comparable plan quality compared to SPArc_original_ (Fig1). The DRVH results indicated that SPArc_original_ could not achieve FLASH using the clinic beam current configuration, while SPLASH_LET_ could significantly not only improve V_40Gy/s_ in target and region of interest(ROI) but also improve the mean LET in the target and reduce the high LET in organ at risk(OAR) in comparison with SPArc_original_ (Table 1,Fig1).

**Conclusion**: SPLASH_LET_ offers the first LET painting with voxel-based ultra-dose-rate and high-dose conformity treatment using proton beam therapy. Such technique has the potential to take full vantage of LET painting, FLASH and SPArc.

### O 198 - Quantitative real-time imaging of ultra-high dose rate proton pencil beams

#### Megan Clark^1^, Petr Bruza^1^, Rongxiao Zhang^2^, James Kraus^3^, Rex Cardan^3^, Brian Pogue^4^, Joseph Harms^3^

##### ^1^Dartmouth College, Thayer School of Engineering, Hanover, USA ^2^Dartmouth-Hitchcock Medical Center, Radiation Oncology, Lebanon, USA ^3^University of Alabama at Birmingham, Radiation Oncology, Birmingham, USA ^4^University of Wisconsin-Madison, Medical Physics, Madison, USA

**Purpose**: Radiation therapy (RT) delivered at ultra-high dose rates (FLASH) can increase the therapeutic ratio. However, RT delivered at high dose rates below the FLASH threshold are more toxic than RT at conventional dose rates. For clinical translation of FLASH, it is paramount that dose and dose rate are measured during patient-specific quality assurance (PSQA). Here, we report the performance of a real-time imaging system for characterization of proton FLASH beams.

**Methods**: An ultra-fast (>1 kHz), CMOS camera was placed 2 meters away from isocenter during delivery of a series of 5x5cm^2^ PBS fields ranging from 1.7-23.3 Gy and 8-100 Gy/s. A scintillation screen was placed in the beamline for spatial-temporal characterization. EBT3 film was used to produce a scintillation intensity-to-dose calibration curve, and dose rate calculations were made considering effective radiation time, and spatial and temporal dose profiles and dose rate information were compared to measurement with an ADCL-calibrated PPCO5 ion chamber.

**Results**: As compared to the PPC05, the scintillator intensity response scaled linearly with dose (R^2^=0.996) and mean dose rate (isocenter dose/irradiation time, R^2^=0.994). Gamma analysis at 3%/2mm showed strong agreement between film and scintillator, with a passing rate of 100%. Finally, the scintillator showed that planned and delivered spot positions agreed within 1.0±0.9mm.

**Conclusions**: We have shown that the CMOS camera is capable of measuring proton FLASH beams as each spot is scanned. This study is an important step towards verification of dose rate distributions for PSQA.

### O 199 - Aperture position optimization for LATTICE therapy

#### Weijie Zhang^1^, Yuting Lin^1^, Fen Wang^1^, Rajeev Badkul^1^, Ronald Chen^1^, Hao Gao^1^

##### ^1^University of Kansas Medical Center, Radiation Oncology, Kansas City, USA

**Purpose**: LATTICE radiation therapy (RT) aims to deliver 3D heterogenous dose of high peak-to-valley dose ratio (PVDR) to the tumor target, with peak dose at lattice apertures inside the target and valley dose for the rest of the target. In current clinical practice the lattice aperture positions are constant during treatment planning. This work proposes a new LATTICE plan optimization method that can optimize lattice aperture positions as plan variables, which is the first lattice aperture position optimization (APO) study to the best of our knowledge.

**Methods**: The new LATTICE treatment planning method optimizes lattice aperture positions as well as other plan variables (e.g., photon fluences or proton spot weights), with optimization objectives for target PVDR and organs-at-risk (OAR) sparing. To satisfy mathematical differentiability, the lattice apertures are approximated in sigmoid functions. For geometric feasibility, proper geometry constraints are enforced onto the lattice aperture positions. The lattice APO problem is solved by iterative convex relaxation method, where lattice aperture positions and photon/proton plan variables are jointly updated via the Quasi-Newton method.

**Results**: Both photon and proton LATTICE RT were considered, and the optimal lattice aperture positions in terms of plan objectives were found by solving all possible combinations on given discrete positions via heuristic searching based on standard IMRT/IMPT, which served as the ground truth for validating the new LATTICE method. The results show that the new method indeed provided the optimal lattice aperture positions with the smallest optimization objective, the largest target PVDR, and the best OAR sparing.

### O 200 - Radiation dose to circulating lymphocytes: Impact of proton FLASH radiotherapy and spot-scanning proton arc therapy for glioblastoma patients

#### Abdelkhalek Hammi ^1^

##### ^1^TU Dortmund University, Physics, Dortmund, Germany

**Purpose**: To investigate the potential protective effect of ultrahigh dose rate proton radiotherapy (FLASH-PT) on circulating lymphocytes (CL) against intensity-modulated proton therapy (IMPT) and spot-scanning proton arc therapy (SPArc).

**Materials and Methods**: A framework computing instantaneous dose to CL was implemented, encompassing two independent dynamic models: first, a beam delivery model simulating spatially varying instantaneous dose-rates inside patients based on parameters of a commercially available proton cyclotron. Second, a dosimetric-blood flow model (d-BFM) continuously simulating the spatiotemporal distribution of blood particles (BPs). The whole-body blood counts 78*10^6^ BPs. For brain patients, arterial branches were segmented from MR-angiography images. The probability of BPs to traverse bifurcations was simulated with a discrete-time Markov chain. We applied the d-BFM to seven Glioblastoma patients and compared FLASH-PT (60Gy) using currents of 300nA and minimum spot-weights of 1.2*10^7^ protons, and fractionated- IMPT and SPArc (2Gy) assuming clinical currents of 2nA. A FLASH effectiveness model was applied to assess the biologically effective dose during FLASH-PT .

**Results**: The irradiated blood volume (V>_0Gy_) after treatment increased from 1.6% ±0.3% for FLASH-PT to 2.1% ±0.2% and 11.4% ±4.1% for IMPT and SPArc, respectively. The CL depletion was 0.2% during FLASH-PT and increases to 3.1% and 1.4% for IMPT and SPArc, respectively.

**Conclusion**: Our dosimetric-blood flow model provides quantitative measures of the FLASH effect for different Glioblastoma patient scenarios, supporting assumptions about reducing risks of lymphopenia. Compared to IMPT, SPArc reduces the CL depletion of CL at the cost of increased irradiated blood volume.

## Poster Abstracts

### P 001 - Radiosensitization by PARP inhibition to carbon ion radiation therapy in nasopharyngeal carcinoma cell line

#### Haojiong Zhang^1^, Ziyu Le^1^, Kong Lin^2^, Jiade Lu^3^

##### ^1^Shanghai proton and heavy ion center, Radiation oncology, Shanghai, China ^2^Shanghai proton and heavy ion center- Fudan University Cancer Hospital, Radiation oncology, Shanghai, China ^3^Heyou international hospital, proton and heavy ion center, Foshan, China

**Background**: The poly (ADP-ribose) polymerase (PARP) family is related to DNA damage response, and PARP inhibitors (PARPi) have been demonstrated as a radiosensitizer in photon radiation and achieved inspiring results in some tumor treatments. Thus, we hypothesize that PARPi also has a synergistic effect in Carbon ion radiation therapy (CRT).

**Methods**: Olaparib and the nasopharyngeal carcinoma (NPC) cell line HK1 cells was chosen for our study. Four groups were set, control (N), Olaparib alone (O), carbon ion radiation alone (C) and Olaparib+Carbon ion combination group (CO). Cell survival was tested by CCK8. Colony formation and FACS were used to test apoptosis and cell cycles.

**Results**:

1. Olaparib and Carbon ion radiation combination group exhibited enhancement of radiosensitivity (fig1,2). Cell colony formation assay was performed in Carbon ion radiation and combination group, at 10% cell survival, the enhancement ratio of radiosensitivity was 1.98.
2. Cell apoptosis was increased in the combination group (fig3). we examined the cell apoptosis in all groups, we found that Olaparib alone did not change cell apoptosis a lot, while when Olaparib was combined with CRT, the apoptosis percentage increased significantly.
3. G2/M cell cycle arrest was noticed in the combination group (fig3). we tested the cell cycle distribution of four groups and observed that Olaparib with CRT can enhance G2/M cell cycle arrest.

**Conclusion**: Olaparib can significantly increase the radiosensitivity of NPC cells to carbon ion irradiation, which is expected to be an alternative option for CRT combination therapy.

### P 002 - Possibility of tumor diagnosis by beam irradiation: Comparison of biological washout rates in rats between in-beam PET and DEC-MRI

#### Chie Toramatsu^1^, Akram Mohammadi^1^, Nobuhiro Nitta^1^, Hidekatsu Wakizaka^1^, Yoko Ikoma^1^, Chie Seki^1^, Iwao Kanno^1^, Taiga Yamaya^1^

##### ^1^National Institutes for Quantum Science and Technology QST, Department of Advanced Nuclear Medicine Sciences, Chiba, Japan

Positron emission tomography (PET) has been used for treatment verification in charged particle therapy. This is based on the detection of positron emitters produced through fragmentation reactions in a patient. However, correction of the biological washout effect of the produced positron-emitters has been an issue. Modelling the washout effect is complicated, but we think that it may indicate the hemodynamics of tumour. At present, dynamic contrast-enhanced magnetic resonance imaging (DCE-MRI) is clinically used to evaluate the hemodynamics and radiation response of a tumour. In this work, we compared the washout rate of the positron-emitters produced during ^12^C beam irradiation and that of the MRI contrast agent, and explore the possibility of tumor diagnosis just by the beam irradiation. Different vascular types of tumour models were prepared using three rats. The tumour in each rat was irradiated by a ^12^C beam and scanned by in-beam PET (Fig.1-a). Then, the DCE-MRI experiment was performed for the same rats (Fig.1-b). The washout rate of the produced positron-emitters (*k_2,1st_*) and the MRI contrast agent (*k_2a_*) were derived using the single tissue compartment model (Fig.2-a, b). Obtained *k_2,1st_* and *k_2a_*, showed a linear correlation and they were inversely related to fractional necrotic volume. This is the first comparison study on the washout rate of the positron-emitters produced via a nuclear fragmentation reaction in tumour tissue and that of the MRI contrast agent administrated intravenously. This study suggested that the produced positron-emitters may function as a predictor of tumor response in radiation therapy.

### P 003 - DNA repair pathway choice after proton irradiation

#### Amaya Krusch^1^, David Eilenstein^1^, Henrieke Förster^1^, Alexander Loewer^2^, Marco Durante^1^, Burkhard Jakob^1^

##### ^1^GSI Darmstadt, Biophysic, Darmstadt, Germany ^2^TU Darmstadt, Biology, Darmstadt, Germany

**Introduction**: DNA double strand breaks (DSB) are the most relevant DNA lesion leading to cell killing after radiation. Non-homologous end joining (NHEJ) is active throughout the cell-cycle and is the most frequently used repair mechanism in human cells. NHEJ is a fast but error-prone repair pathway. Homologous recombination (HR) repair is active only during S and G2 phase, but is more accurate due to the utilization of the sister chromatid as a template. Recent findings suggest a higher involvement of HR in the repair of proton-induced DNA-DSBs, which could have a large impact on patient selection and combined treatments in proton therapy.

**Methods**: We used live cell imaging of human osteosarcoma cells (U2OS) stably expressing 53BP1-YFP and Rad52-mCherry to address the repair pathway utilization in a cell-cycle dependent manner after irradiation with 2 Gy of protons (mid-SOBP) or x-rays. To determine time of mitosis, time lapse series of cells were acquired in a large area using a confocal spinning disk microscope equipped with a climate chamber starting 24 h before irradiation. After irradiation, imaging was continued for an additional 24 h post-irradiation. Live-cell imaging results were correlated to immuno-cytochemistry and clonogenic survival data in combination with HR inhibition.

**Results**: Live cell experiments demonstrate a minor contribution of HR for both radiation qualities in repair proficient cells. Immuno-cytochemical data shows similar maximum RAD51 foci counts after photons or proton irradiation. However, delayed Rad51 removal in proton irradiated cells indicate a differential processing of a small subset of DNA-DSBs.

### P 004 - Olaparib elicits different biological effects in normal mouse tissues when combined with photon or proton beams

#### Dinu Stefan^1^, Mélody Mahier^2^, Elena Lequesne^2^, Frédéric Pouzoulet^3^, Ludovic De Marzi^4^, Frédérique Megnin-Chanet^5^, Jean-Louis Habrand^1^, Stéphanie Gente^2^, François Sichel^6^, Carine Laurent^7^

##### ^1^CLCC Baclesse- Université de Caen-Normandie, Radiotherapy Department- ABTE-ToxEMAC, Caen, France ^2^Université de Caen-Normandie, ABTE-ToxEMAC, Caen, France ^3^Institut Curie- PSL Research University, Translational Research Department, Orsay, France ^4^Institut Curie - Proton Therapy Center, Medical Physics Department, Orsay, France ^5^INSERM- Institut Curie- CNRS, Unit 1196- UMR9187, Orsay, France ^6^Université de Caen-Normandie- CLCC Baclesse, ABTE-ToxEMAC, Caen, France ^7^Université de Caen-Normandie- SAPHYN- CLCC Baclesse, ABTE-ToxEMAC, Caen, France

PARP inhibitors (PARPi) have a recognized radio-sensitizing effect by causing an increase in unrepaired DNA breaks after irradiation. However, their toxicity in association with irradiation has not been adequately studied *in vivo*. For this purpose, C57Bl6 mice were whole-body irradiated with photons or proton beams +/− olaparib. Olaparib did not modify the survival (Fig.1) or the weight of unirradiated mice but strongly decreased them when associated to irradiation. Blood and various organs were collected after the onset of acute toxicities. Skin, brain, lung, heart, small intestine and liver were cryomilled and biomarkers of genotoxicity, oxidative stress and inflammation were measured. PARP1 activity was increased only after photons and the addition of olaparib decreased it. Moreover, olaparib caused oxidative damage to lipids and proteins varying according to the tissues with an inverse effect when combined with photons or protons. The level of plasma inflammatory cytokines (Fig.2) was increased after photons or protons. Olaparib decreased TNF-α, IFN-γ, but also IL-10, and increased IL-6 and IL-12p70. In conclusion, protons leaded to: an increase in acute toxicity compared to photons (mouse survival and weight, PARP1 activity) but a decrease in oxidative damage to lipids and proteins in the majority of tissues. The combination with olaparib leaded to an increase in acute toxicity (survival, weight, PARP1 activity, pro- and anti-inflammatory cytokines) but also to a decrease in other pro-inflammatory cytokines and in oxidative damage to lipids (after photons only). This study will enable the clinical use of olaparib associated with photon or proton beam radiotherapy.

### P 005 - Prostate cancer cells more sensitive to protons with relative biological effectiveness over 1.5

#### Li Wang^1^, Xiaochun Wang^2^, Yuting Li^2^, Yinhan Zhang^1^, Jennifer Heldring^1^, Yupeng Li^2^, Jiafu Tang^2^, Narayan Sahoo^2^, Xiaorong Ronald Zhu^2^, Steven J. Frank^3^

##### ^1^The University of Texas MD Anderson Cancer Center, Experimental Radiation Oncology, Houston, USA ^2^The University of Texas MD Anderson Cancer Center, Radiation Physics, Houston, USA ^3^The University of Texas MD Anderson Cancer Center, Radiation Oncology, Houston, USA

**Purpose**: Proton radiotherapy (PRT) is effective in prostate cancer (PC), with favorable disease control and toxicity outcomes. However, radiobiologic evidence of advantages of PRT over photon radiotherapy (XRT) for PC is limited, and the molecular responses of PC cells to protons versus photons are unclear. We studied the molecular effects of PRT versus XRT in human PC cell lines.

**Methods**: We analyzed Du145, LNCap, and PC-3 cells as follows. Proton relative biological effectiveness (RBE) was determined by clonogenic survival assays after a dose of 2-, 4-, or 6-Gy RT. After a 4-Gy dose of PRT or XRT, radiation-induced DNA double-strand breaks (DSBs) were assessed by 53BP1 foci; cell cycle distribution (Propidium Iodide fluorescent dye staining), apoptosis, and necrosis (with Annexin V-FITC-conjugated stain) were analyzed by flow cytometry. Effects induced by PRT versus XRT were compared with Student’s t-tests.

**Results**: PRT killed more cells than XRT at all tested fraction sizes (all RBEs ≥1.09); RBE was influenced by cell type and radiation fraction size. PRT led to more persistent unrepaired DSBs at 24 hours (Figure 1, P<0.01). PRT and XRT seemed to cause similar trends (non-significant) of increased cell necrosis (at 48 hours) and apoptosis (at both 24 and 48 hours). PRT led to trends of greater cell cycle arrest at G2/M than XRT at 24 hours (Figure 2).

**Conclusion**: Prostate cancer cells were more sensitive to PRT than XRT. These effects may be partially related to PRT delaying DNA double-strand-break repair and increasing cell cycle arrest at G2/M.

### P 006 - High LET protons amplify radiation induced cell cycle delay compared to photons and low LET protons

#### Charlotte Heaven^1,2^, John-William Warmenhoven^1,2^, Nicholas T. Henthorn^1,2^, Amy L. Chadwick^1,2^, Elham Santina^1,2^, Jamie Honeychurch^1^, Christine K. Schmidt^1^, Norman F. Kirkby^2^, Karen J. Kirkby^1,2^, Michael J. Merchant^1,2^

##### ^1^University of Manchester, Division of Cancer Sciences, Manchester, United Kingdom ^2^The Christie NHS FT, Manchester Academic Health Science Centre, Manchester, United Kingdom

Proton therapy may result in differences in the extent and complexity of DNA damage compared to photon irradiation. The higher linear energy transfer (LET) property of Bragg peak protons creates more complex and clustered DNA damage. In this study we investigated the effect of different radiation induced damage on the cell cycle, using 300 kVp photons, low LET protons (0.4 keV/μm) and higher LET protons (average 6.5 keV/μm). RPE1 and H358 cells were irradiated at doses of 2 Gy and 4 Gy. Their cell cycle distributions were then analysed using flow cytometry 8, 16, 32 and 56 hours later and compared to sham irradiation. Analysis revealed that RPE1 cells, wild type for TP53, produce a stronger G2/M checkpoint block with low LET protons than photons which was dose dependent. A larger proportion of cells were held in G2/M following irradiation with the higher LET protons, suggesting an LET dependence in this response (figure 1). A second cell line (H358) that is TP53 null revealed a different cell cycle response, with a greater G2/M block at lower doses, as has previously been demonstrated in literature. Interestingly, the G2/M block in this second cell line was not dependent on dose or radiation modality, suggesting a different cell cycle response following DNA damage which may be related to TP53 status. These data demonstrate differences in downstream events following proton and photon irradiation. The results may further our understanding of radiation type and LET effects on both cancerous and normal tissue.

### P 007 - Evaluation of LET-dependent biological effects on anti-tumor effect and acute radiation syndrome

#### Takashi Shimokawa^1^, Toshiaki Kokubo^2^, Akiko Uzawa^3^, Tsuguhide Takeshima^3^, Taku Inaniwa^1^, Sumitaka Hasegawa^3^, Toshiyuki Shirai^1^, Shigeru Yamada^3^

##### ^1^Institute for Quantum Medical Science- QST, Department of Accelerator and Medical Physics, Chiba, Japan ^2^Quantum Life and Medical Science Directorate- QST, Laboratory Animal and Genome Sciences Section, Chiba, Japan ^3^Institute for Quantum Medical Science- QST, Department of Charged Particle Therapy Research, Chiba, Japan

Heavy-ion radiation is known to have advantages for cancer therapy and has shown good therapeutic outcomes. However, the current irradiation methods have been planned based on the irradiation dose mainly, and it is not enough to use the full potential of high LET radiation. In particular, the unique biological effects of heavy ion beams, such as RBE and OER, are changed in a LET-dependent manner. In order to enhance these biological effects, it is necessary to increase the LET used for irradiation from the current level. Therefore, it is expected that it will develop into a treatment that takes into consideration the biological effectiveness relative with LET. Although a lot of basic data is needed to provide effective and safe treatment, data on the biological effects of high-LET radiation, especially in animal models, are still limited. In this study, to obtain basic biological data required for future clinical treatment with the LET painting, especially acute radiation syndromes and antitumor effects, we evaluate these biological effects by using various ion beams with different LETs (13 ∼185 keV/µm) and mouse models with different radiosensitivity (C57BL/6J and C3H/He).

### P 008 - The photon isoeffective dose model: A new formalism in hadron therapy for dose calculations in photon equivalent units

#### Lucrecia Mariel Valeriano^1^, Sara J. González^1^, Gustavo A. Santa Cruz^2^

##### ^1^National Atomic Energy Commission, Computational Physics and Radiation Biophysics Division-Coordination Department BNCT, Buenos Aires, Argentina ^2^National Atomic Energy Commission, Nuclear Medicine and Radiotherapy Management, Buenos Aires, Argentina

As part of the research and development activities carried out within the framework of the Argentine Proton Therapy Center Project executed by the National Atomic Energy Commission (CNEA), this work presents a dose calculation formalism to compute photon isoeffective doses [1] for proton therapy avoiding the use of fixed RBE factors. Compared with the existing models that describe the dependence of RBE with LET, the introduced model takes into account all the primary and secondary particle radiation fields, allowing for synergistic effects between different components and sublethal damage repair. Photon isoeffective doses were studied along a Spread-Out Bragg Peak (SOBP) derived by Monte Carlo simulations of the mixed radiation field with PHITS. The radiobiological parameters of the model were obtained from the PIDE database [2] considering Co-60/6 MV reference photon and ion survival data of the V-79 cell line. Compared to the fixed RBE dose profile, isoeffective doses show a gradual increase with depth reaching a maximum deviation at the distal fall edge. This behavior is consistent with that observed with LET dependent RBE models. The fact that this model proved suitable to explain the observed clinical outcome in another hadron therapy, i.e., Boron Neutron Capture Therapy, makes it attractive for proton and carbon ion therapies.

### P 009 - Radiolysis modeling of FLASH during a continuous beam irradiation

#### Miguel Molina-Hernández^1,2,3^, Chi Yujie^4^, Tengda Zhang^1,5^, Patricia Gonçalves^2,3^, João Seco^1,6^

##### ^1^German Research Cancer Center, Division of Biomedical Physics in Radiation Oncology, Heidelberg, Germany ^2^Laboratory of Instrumentation and Experimental Particles Physics, Dosimetria, Lisbon, Portugal ^3^University Técnico Lisboa, Physics Department & Nuclear Sciences and Engineering, Lisbon, Portugal ^4^University of Texas at Arlington, Physics Research & Techniques in Radiation Medicine, Arlington, USA ^5^University of Heidelberg, Medical Faculty Mannheim, Mannheim, Germany ^6^University of Heidelberg, Physics and Astronomy, Heidelberg, Germany

**Introduction**: We have implemented a step-by-step Monte Carlo (MC) continuous irradiation model with gMicroMC that accounts for the temporal structure of the beam, its dose rate, and the water’s oxygen level. Pulse radiolysis has been modeled with multiple step-by-step MCs, meanwhile, continuous irradiation modeling is more computationally demanding, and the codes are still under development.

**Methods**: The model’s input parameters are the particle type and their kinetic energy, the dose rate, and the beam’s pulse micro-structure (frequency and width). To emulate the walls of a water tube, we set a periodic boundary condition (PBC) so that when the radicals hit the walls, they rebound (Figure 1).

**Results**: We simulated a proton beam with the pulse structure produced by an isochronous cyclotron (bunches interspaced by 32.84 ns). The LET was 0.78 keV/um, the dose rate was 30 kGy/s, and the total dose deposition was 5Gy. The oxygen partial pressure was 4%. Figure 2 shows the G values time-dose evolution during the irradiation and the radiochemical oxygen depletion (ROD). The G0 matched the experimental data. The yields of the hydrated electron, hydroxyl, and superoxide decreased through the irradiation, while hydrogen peroxide increased. The ROD was 0.4%/5Gy, which is consistent with experimental data published by our group.

**Conclusions**: The model is innovative due to its capacity to simulate the yield G of continuous irradiations. The PBC replicates the walls of a water tube, and it is key for ROD calculations, in terms of computational efficacy. The model is currently being calibrated with experimental G-values.

### P 010 - Impact of physical parameters on GBM survival curves in enhanced radiotherapy with gold nanoparticles

#### Joana Sequeira Antunes^1,2^, Jorge Miguel Sampaio^1,2^, Filipa Mendes^3^, António Paulo^3^

##### ^1^LIP - Laboratório de Instrumentação e Física Experimental de Partículas, Dosimetry, Lisbon, Portugal ^2^FCUL - Faculdade de Ciências da Universidade de Lisboa, Physics, Lisbon, Portugal ^3^C2TN - Centro de Ciências e Tecnologias Nucleares, DECN - Departamento de Engenharia e Ciências Nucleares- Instituto Superior Técnico, Lisbon, Portugal

**Purpose**: The combination of high-Z nanoparticles and radiotherapy leads to an increased radiation effect in tumoral cells without increasing the patient dose. In this work, we review the impact of physical parameters in the cell survival curves by applying a version of the Local Effect Model (LEM) in the presence of gold nanoparticles (AuNPs). Dose Enhancement Factor (DEF) and the Sensitive Enhancement Factor (SEF) are determined as a function of concentration, size, and distribution of AuNPs inside the cell.

**Material and Methods**: A method to reconstruct confocal microscopy images that allows using voxelized realistic cell geometries in MC simulations using TOPAS is presented. This method is applied to image reconstruction of GBM U87 and U373 cell lines. The simulations were benchmarked against Co-60 irradiations (figure 1).

**Results and Conclusions**: Keeping the number AuNP radius constant (5 nm) it is not observed a clear trend in the dose effect as a function of the mass of gold in the cytoplasm. The same happens when the gold mass inside is kept constant (0.008 ng) but the NP radius varies (5 - 50 nm). For a constant number of NPs (7.9 × 105) there is a critical radius (figure 2) above which the dose effect increases significantly. This effect is more significant when the NPs are close to the nucleus. These results suggest there is a balance between the increase in radiation and the shielding produced by the NPs.

### P 011 - Optimization of cell survival quantification method to study RBE with different LETs in a proton therapy irradiation facility

#### Marta Ibañez^1^, Natalia Chamorro^1^, Juan Ignacio Lagares^1^, Marta Oteo^1^, Víctor M. Luján-Rodríguez^1^, Juan Diego Azcona^2^, Miguel Ángel Martín-Rey^3^, Edilia I. De Almeida^4^, Pedro Arce Dubois^1^, Miguel Ángel Morcillo^1^

##### ^1^Centro de Investigaciones Energéticas- Medioambientales y Tecnológicas CIEMAT, Unidad de Aplicaciones Médicas de las Radiaciones Ionizantes, Madrid, Spain ^2^Clínica Universidad de Navarra, Radiophysics and Radiological Protection, Madrid, Spain ^3^Centro de Investigaciones Energéticas- Medioambientales y Tecnológicas CIEMAT, Hematopoietic Innovative Therapies, Madrid, Spain ^4^Centro de Investigaciones Energéticas- Medioambientales y Tecnológicas CIEMAT, Animal Facility, Madrid, Spain

A large amount of noise exists in the biological datasets for RBE models, which prevents their translation to the clinic. One of the reasons for this could be the use of clonogenic assays as the gold standard in radiobiology, which is time-consuming and subject-dependent, increasing the uncertainties in the results. Moreover, this method poses challenges when facilities are limited and many samples are required. Herein, we propose crystal violet absorbance instead of colony counting as a new survival quantification method for radiobiology. Absorbance measurement is objective and does not depend on colony formation, allowing the use of 96-well plates to minimize the irradiated area. Therefore, high doses/LETs per plate could be studied in proton therapy facilities. Absorbance was measured as a function of seeded cells, values were fitted to a sigmoidal curve (Figure.1a), and the range of seeded cells required to observe exponential growth (linear range) was obtained for each dose. We verified this method using different cell lines (U251, RenCa, 4T1.2, and V79) irradiated with an X-ray source before proceeding with protons. The cells were then stained and diluted with methanol. EC50 values (number of cells that gave a half-maximal response) were used to calculate the Survival Fractions. The results showed negligible differences from those obtained using 6-well plates and colony counting (Figure.1b). In summary, this new method allows a reproducible, easy, economical, objective, and rapid procedure to study the effect of irradiation on cell survival, allowing the use of many samples and cell lines in fewer irradiation sessions.

### P 012 - Calculating domain radius in the framework of the Microdosimetric Kinetic Model for different cell lines based on in vitro experiments

#### Daniel Suarez-Garcia^1^, Miguel Antonio Cortes-Giraldo^1^, Alejandro Bertolet^2^

##### ^1^Universidad de Sevilla, Department of Atomic- Molecular and Nuclear Physics, Sevilla, Spain ^2^Massachusetts General Hospital and Harvard Medical School, Department of Radiation Oncology, Boston- MA, USA

The Microdosimetric Kinetic Model (MKM) proposes the concept of a domain as the maximum distance that two sublethal lesions may travel to interact and lead to cell death. In proton radiotherapy, it is commonly assumed that the relative biological effectiveness (RBE) is constant at 1.1. However, heavier ions show larger RBE values which challenge their safe clinical application, so it is necessary to use more complex models, including radiobiological processes. In this work, we analyzed the Particle Irradiation Database Ensemble (PIDE) from the GSI, which provides data on ion type, irradiation conditions, cell line information, and the α and β parameters in the context of the Linear-Quadratic (LQ) model for various clonogenic experiments. By analyzing experiments involving protons and alpha particles, we obtained statistical distributions of domain sizes (rd) for different cell lines using the MKM. These results illustrate the differences in radiosensitivity across biological systems and supports the need for a variable parameter in the MKM that can be adapted to each system. We also provide a dataset of values for various cell lines.

### P 013 - Multi-criteria optimization for decision making in LET-based PBS treatment planning of brain tumors

#### Anne Vestergaard^1^, Erik Traneus^2^, Aida Muhic^1,3^, Yasmin Lassen-Ramshad^1^, Rikke Dahlrot^1,4,5^, Jesper Kallehauge^1^, Ole Nørrevang^1^, Bob Smulders^1,3^, Morten Høyer^1^

##### ^1^Aarhus University Hospital, Danish Centre for Particle Therapy, Aarhus N, Denmark ^2^RaySearch Laboratories AB, Physics Research, Stockholm, Sweden ^3^Rigshospitalet, Department of Oncology, Copenhagen, Denmark ^4^University of Odense, Department of Oncology, Odense, Denmark ^5^University of Southern Denmark, Departement of clinical research, Odense, Denmark

Treatment planning of proton therapy for brain tumors is based on RBE=1.1. Using LET in the optimization is currently investigated and addition of LET-based objectives to the optimization demands for evaluation of its costs and benefits. The aim of this study was to investigate the use of a newly developed Multi Criteria Optimization tool (MCO) for selecting the optimal plan for treatment of tumors close to the brainstem. Treatment plans for five patients with meningioma (prescribed dose 54 GyRBE) were optimized in a research version of Raystation (11B-IonPG, Raysearch Laboratories) using beam angles from the clinically treated plans. Pareto plans were generated with an objective on the dose component in the brainstem with LET>3 keV/µm (dirty dose (DD)). Multiple plan candidates were compared spanning over noDD to maxDD weight on the DD component in the brainstem. Oncologists selected the most suitable plan comparing variable RBE (McNamara, brainstem α/β=2) and the DD dose distributions. For all patients the maxDD decreased the brainstem D2cc (Table 1). In three out of five patients this was achieved without increase in other high-risk organs. For patient 1 and 2, the mean dose to the cochlea increased, but this increase was not judged to be of clinical importance. Two patients had an increase in chiasm D2%. For all patients the maxDD plan was considered superior by the oncologist. The newly developed MCO-tool is useful for evaluating the cost and benefit of LET-based dose constraints and for selecting the optimal plan for the patient.

### P 014 - Novel cell survival assay design to score end-of-range RBE at voxel size resolution for application in proton treatment planning

#### Erik Traneus^1^, Anders Ahnesjö^2^

##### ^1^RaySearch Laboratories AB, Research Department, Stockholm, Sweden ^2^Uppsala University, Cancer Precision Medicine IGP, Uppsala, Sweden

**Purpose**: Hypothesizing that end of range dose is predictive for proton RBE (Figure 1), we propose a novel assay design and present design parameters to measure survival for end of range doses delivered at voxel dimensions relevant for treatment planning. The overall aim is to facilitate data collection for RBE modelling suited for treatment planning applications.

**Methods**: It has been demonstrated that physical dose from energy depositions at LET>LET_thres_ (“Dirty Dose”) can be used as an effective driver for re-distributing high LET in proton treatment planning optimization (Figure 2). To assess the Dirty Dose associated RBE_DD_ we propose an assay where a cell suspension flows through a 3x3 mm^2^ channel irradiated by a static quasi-monoenergetic beam with range 2.85 mm (1 nA, 6 mm FWHM, 16 MeV) selected to match application of LET_thres_ = 3.1 keV/mm at plan optimization. For survival fraction scoring, the irradiated suspension is supposed to be deposited on a growth medium with a parallel non-irradiated suspension deposited simultaneously on an identical plate as reference.

**Results**: For the exemplary numbers above, the flow speed for 1 Gy irradiation should be 0.9 mm/s or 0.5 cm^3^/min corresponding to 10 cm^2^ plating surface at 0.5 mm thickness prior optional dilution.

**Conclusions**: We conclude that it should be feasible to collect data to determine cell survival and RBE for protons with LET>LET_thres_ direct in voxel geometry resolution. In the proposed method, a single data point is measured in contrast to traditional LQ-model based assays requiring extensive sets of dose and LET survival data points.

### P 015 - Lymphopenia patterns and radiation dose in circulating blood estimated by magnetic resonance imaging (MRI) flow

#### Rosa Meirino^1^, Javier Aristu^1^, Javier Serrano^1^, Borja Aguilar^2^, Javier Burguete^3^, María García Cardosa^3^, Jacobo Palma^1^, Carlota Guibert^1^, Marta Vidorreta^4^, Felipe Calvo^1^

##### ^1^Clinica Universidad de Navarra, Radiation Oncology, Madrid, Spain ^2^Clinica Universidad de Navarra, Radiophysics, Madrid, Spain ^3^Universidad de Navarra, Physics and applied mathematics department, Madrid, Spain ^4^Siemens, Siemens-healthineers, Madrid, Solomon Islands

**Background**: Circulating blood exposure to irradiation can be estimated in clinical practice introducing MRI flow into imaging for dosimetry treatment planning. Lymphocytes (L) counts are a biomarker of dose delivered to this biological target.

**Methods**: A prospective registration study (March 2021-May 2022) on 37 patients treated with a radiotherapy component (31 with proton and 6 with photon therapy), MRI-4D flow were performed. The sequences were exported to RayStation-TPS for vessels segmentation. Dose-volume-histograms were generated to estimate correlations between dosimetric distributions and lymphocytes count. EDIC (was calculated according to the formula (Figure 1)The integral dose in vessels (IDV) were calculated defining it as a ID [Gy·L]=D [Gy]·V [L], D=mean dose, V=Volume. Lymphocytes values were characterized as: total L and lymphopenia grade 3-4 (27 patients).

**Results**: The median EDIC was: 1.2 Gy. A comparison of means between patients treated with protons vs. photons (1.25 vs. 4.11) showed significance (p< 0.05). Similarly, the mean IDV was 0.24 Gy*L: protons vs. photons (0.38 vs. 4.9, p<0.05). With regards to the development of lymphopenia the mean EDIC value was statistically different: 4.11 VS 1.2 Gy (p <0.05). The IDV value correlated with the event lymphopenia: 4.67 VS 0.39 Gy*L (p <0.05). Six patients developed grade III or IV lymphopenia: 3 in the photontherapy group (50%) and 3 in the protontherapy group (14%).

**Conclusions**: Strategies to quantify the irradiation exposure to circulating blood and its impact on radiation-associated lymphopenia requires high quality imaging of vascular structures to generate specific DVHs Preliminary correlations with lymphopenia patterns are observed after proton and photon irradiation.

### P 016 - Proton irradiation of vascular structures and presence of biomarkers in standard clinical practice

#### Rosa Meirino^1^, Javier Aristu^1^, Javier Serrano^1^, Josune Orbe^2^, Diego Azcona^3^, Javier Burguete^4^, Marina Garcia Cardoso^4^, Jacobo Palma^1^, Felipe Calvo Manuel^1^

##### ^1^Clinica Universidad de Navarra, Radiation Oncology, Madrid, Spain ^2^Universidad de Navarra CIMA, Centro Investigación Medicina Aplicada, Madrid, Spain ^3^Clinica Universidad de Navarra, Radiophysics, Madrid, Spain ^4^Universidad de Navarra, Physics and applied mathematics, Pamplona, Spain

**Background**: The exposure of vascular structures to irradiation has been prospectively investigated in patients undergoing standard proton-therapy.

**Methods**: Between March 2021 and May 2022 in 20 patients treated with a radiotherapy component with proton therapy, series of MRI-4D flow images were performed to visualize the entire vascular tree. Reconstruction of the sequences were exported to RayStation TPS for vessels semi- automatic segmentation. Dose-volume-histograms (DVHs) were generated to estimate a potential correlation between dosimetric value and biomarkers values. EDIC (the effective dose to the circulating immune cells) was calculated according to the formula (Figure). The integral dose in vessels (IDV**)** were calculated defining it as a ID [Gy·L]=D [Gy]·V [L], where D is the mean dose delivered to volume V. Biomarkers of vascular damage studied were: p selectin, matrix metalloproteases (MMP) and citrullinated-H3. Pre and post proton irradiation determinations were analyzed.

**Results**: The median EDIC was: 1.079 Gy and the mean IDV was 0.13 Gy*L. Among the biomarkers studied, the variation in MMP9 is remarkable. The median was 3.96 ng/ml. 15 (75%) patients presented an increase at the end of treatment. The relationship of the increase in MMP9 with the values of EDIC (greater or less than 1 Gy) and IDV (greater or less than 0.1 Gy *L) showed a significant increase in the subgroups with a higher EDIC and IDV values (p< 0.05).

**Conclusion**: Exposure to proton irradiation of vascular structures induces presence of biomarkers related to endothelial damage. Preliminarily, MMP9 has been shown to be a determinant marker of the vessel proton irradiation.

### P 018 - Current status of BNCT and perspectives

#### Ignacio Porras ^1^

##### ^1^Universidad de Granada, Física Atómica- Molecular y Nuclear, Granada, Spain

Boron Neutron Capture Therapy (BNCT) has recently shown very promising results for the treatment of cancers of bad prognosis such as Glioblastoma Multiforme or recurrent head and neck cancers. This therapy is now facing a renaissance with the introduction of a new generation of in-hospital accelerator based BNCT centers, and some of them had started clinical trials. In this talk, a summary of previous results from the most recent clinical trials performed at research reactors, the first results from accelerator-based ones as well as the projected new facitilies around the world would be presented. In addition to this, some open research problems will be discussed, as it is the search for new boron compounds, the potential synergies with immunotherapies and the reduction of uncertainties with improved nuclear and radiobiological data and boron distribution measuments.

### P 019 - Preliminary results of the automatic segmentation of head-neck tumours in CT images using an nnUNet to enhance BNCT TPS

#### Francesco Morosato^1,2^, Ian Postuma^2^, Silva Bortolussi^1,2^, Francesca Brero^2^, Francesca Lizzi^3^, Barbara Marcaccio^1,2^, Valerio Vercesi^2^, Setareh Fatemi^2^

##### ^1^University of Pavia, Physics, Pavia, Italy ^2^Italian National Institute of Nuclear Physics - INFN, Unit of Pavia, Pavia, Italy ^3^Italian National Institute of Nuclear Physics - INFN, Unit of Pisa, Pisa, Italy

Clinical BNCT improvement and personalisation are main objectives in the ISNCT community. In this frame Artificial Intelligence (AI) can aid in the efforts to obtain precise and individualised treatments. Deep Learning (DL) is a powerful tool that can be used to automatically segment the ROI’s of interest for the therapy plan. The application of DL shows promise to segment large datasets of medical images in short time and without human induced variability. This could be helpful both for radiologists to quicken their tasks of contouring and for researchers since it would provide access to big datasets of segmented images useful to test their TPS. The AI_MIGHT project aims to apply DL to segment and study two tumour types commonly treated with BNCT, in particular Head and Neck tumours and Glioblastomas. AI_MIGHT will also take into account two imaging modalities: CT images as the gold standard in clinical BNCT and MRI as a future possible method of boron imaging in BNCT. This preliminary work has focused on Head and Neck tumours and CT images. The first step was to create a standardised dataset of those images collected from the Cancer Imaging Archive, a public database. Subsequently a neural network was trained and tested for the automatic segmentation of tumour ROIs in CT images of Head and Neck cancer patients. The segmented images will be the basis on which the TPS and dosimetry will be calculated. Results of this preliminary computational study are presented in this work.

### P 020 - Simulation of normal brain dosimetry of BNCT with fractionated X-ray therapy for application to newly diagnosed glioblastoma

#### Kei Nakai^1^, Masashi Mizumoto^1^, Kenta Takada^2^, Hiroaki Kumada^1^, Yoshitaka Matsumoto^1^, Toshimitsu Hayashi^3^, Eiichi Ishikawa^4^, Akira Matsumura^5^, Koichi Hashimoto^6^, Hideyuki Sakurai^1^

##### ^1^University of Tsukuba, Department of Radiation Oncology- Proton Medical Research Center, Tsukuba- Ibaraki, Japan ^2^Graduate School of Radiological Technology, Gunma Prefectural College of Health Sciences, Maebashi-Gunma, Japan ^3^Stella Pharma Corporation, pharmaceutical department, Osaka, Japan ^4^University of Tsukuba, Department of Neurosurgery- Faculty of Medicine, Tsukuba- Ibaraki, Japan ^5^Ibaraki Prefectural university of Health Sciences, President, Ami- Ibaraki, Japan ^6^University of Tsukuba, Tsukuba Clinical Research & Development Organization, Tsukuba- Ibaraki, Japan

We have been conducting a clinical study of reactor based BNCT for newly diagnosed glioblastoma. The final protocol was BPA-BNCT followed by 30 Gy. or 40 Gy fractionated X-irradiation. No grade 3 or higher adverse events were observed, suggesting improved survival, but the clinical trial was terminated due to restrictions on the use of nuclear reactors. Therefore, we decided to start up a newly developed accelerator neutron source and investigate the indication of BNCT with X-rays for newly diagnosed glioblastoma. In normal brain tissue, the boron concentration was the same as the blood concentration. The RBE values for thermal and epithermal neutrons were assumed to be those used in conventional reactors, since theoretically there is no difference between neutron sources (Table 1). CBE (compound biological effect) was assumed to be similar. Experimental RBE values for fast neutrons were used because they vary from instrument to instrument. Monte Carlo simulations were performed using a normal brain phantom with a virtual tumor lesion (Figure 1). In this case, the BNCT dose rule is the maximum dose to normal brain tissue. In the present study, the LQ model was applied, and when α/β were set to 2.0 to 3.0, the BNCT dose was calculated to be 8-8.6 Gy equivalent, which is the dose that can be delivered without exceeding the standard treatment dose of 60 Gy. The optimal calculation method and parameters require further study.

### P 021 - Tumor microenvironment influence in BNCT treated HT29 colon adenocarcinoma cell line

#### Carla Rodriguez^1^, Yanina Ferreyra^1^, Romina Oglio^1^, Marina Perona^2^, Erika Saroka^1^, Maria Alejandra Dagrosa^2^, Gustavo Santa Cruz^3^, Lisa Thomasz^2^

##### ^1^National Commission of Atomic Energy, Radiobiology, Buenos Aires, Argentina ^2^National Commission of Atomic Energy - National Council of Scientific and Technical Research, Radiobiology, Buenos Aires, Argentina ^3^National Commission of Atomic Energy - Nuclear Medicine and Radiotherapy Management, Radiobiology, Buenos Aires, Argentina

**Aims**: Colorectal cancer (CRC) represents the third worst diagnosed cancer in the world. In Argentina, CRC is the second most frequent cancer and also the second in mortality and treatments include radiotherapy and immunotherapy. Tumor microenvironment plays a key role in cancer development and progression, for example, sending signals that affect non irradiated cells function. As not much is known about how BNCT contributes to therapeutic outcomes from the tumor microenvironment the aim of this work was to study the up or down regulation of genes related to immunogenicity and metastases in irradiated HT29 colon adenocarcinoma cells and also the bystander effect of BNCT in non-irradiated cells.

**Results**: Until now we observed that radiation with neutrons increases Galectin 1 expression and that BPA attenuates this effect. IFNb increases when cells are irradiated with 8 Gy with and without BPA. An increase of dsDNA was found in the cytosol of irradiated cells. Bystander effect made no difference in proliferation of non irradiated cells but a reduction of cell migration in the BNCT group was observed.

**Conclusion**: The decrease of Galectin 1 induced at high doses in BNCT group and the increased expression of INFb could be related to the antiproliferative effect of BNCT. The increase of dsDNA would contribute to tumor immunogenicity.

### P 022 - Biological models for a preclinical proton minibeam radiotherapy facility

#### Jessica Neubauer^1^, Hendrik Niemeyer^1^, Anne Krull^1^, Nicole Matejka^1^, Aikaterini Rousseti^1^, Sarah Rudigkeit^1^, Günther Dollinger^1^, Judith Reindl^1^

##### ^1^Universtät der Bundeswehr München, applied physics and measurement technology, Neubiberg, Germany

Spatially fractionated radiotherapy using protons, so-called proton minibeam radiotherapy (pMBT) was developed for better sparing of normal tissue in the entrance channel of radiation. Preclinical in-vivo experiments conducted with pMBT in mouse ear models or rat brains support these prospects. However, the research on radiobiological mechanisms and the search for adequate application parameters delivering the most beneficial minibeam therapy is still in its infancy. Progressing towards clinical usage, pMBT research should overcome the technical and biomedical limitations of the current irradiation test stages and animal models. A new pMBT facility at the 68 MeV cyclotron of the Helmholtz Zentrum Berlin is currently being built. A small animal radiation research platform (SARRP) will be mounted in the beamline. It will be used for the positioning of small animals or in vitro samples, x-ray irradiation, onboard CT-imaging, and treatment planning. However, since the late 1950s, the 3R Principle (Replace, Reduce, Refine animal experiments) is defined as a guideline for scientific work dealing with animal models. To follow this principle and reduce or even replace the number of animal experiments, this work discusses the development of biological cancer models which will be used as a preliminary stage of animal models at this pMBT facility. We show possible 3D cell culture models. Additionally, we show evaluation methods using live cell phase contrast as well as fluorescence imaging methods. Further, preliminary results of a tumor model irradiated with x-rays are shown.

### P 023 - Radiopathological aspects of pulmonary proton minibeam radiation therapy

#### Annaïg Bertho^1^, Ramon Ortiz^2^, Julie Espenon^2^, Cristèle Gilbert^2^, Marjorie Juchaux^2^, Annalisa Patriarca^3^, Frédéric Pouzoulet^4^, Ludovic De Marzi^3^, Yolanda Prezado^2^

##### ^1^Institut Curie - CNRS, U1021/UMR 3347, Orsay, France ^2^Institut Curie - CNRS, U1021/umr3347, Orsay, France ^3^Institut Curie, Hospital, Orsay, France ^4^Institut Curie, Experimental radiation therapy department, Orsay, France

Proton minibeam radiation therapy (pMBRT) has shown a remarkable reduction in neurotoxicity [Lamirault et al., 2020] and equivalent or superior tumor control [Prezado et al., 2018; Bertho et al., 2021] over conventional proton therapy (PT). While the majority of pMBRT studies focused on brain or skin irradiations [Sammer et al., 2020; Girts et al., 2015; Bertho et al., 2021], this work reports on the first evaluation of the pulmonary response to pMBRT. Pulmonary irradiations are challenging as cardiorespiratory motion might blur the spatial fractionation of the dose. We compared the radiopathological consequences of pulmonary pMBRT versus conventional PT. Pulmonary irradiations, delivering a mean dose of 17Gy in both modalities, were performed in C57BL/6 mice. The development of radiation-induced pulmonary fibrosis was monitored by cone-beam computed tomography (CBCT). CBCT images revealed a significant increase in lung density following conventional PT, corresponding to the development of radiation-induced lung fibrosis, which ultimately impacted the survival of the animals (6/8 reached the endpoints). The increase in lung tissue density observed in the CBCT images of the pMBRT group was only mild 6 months post-irradiation (Figure1). All the animals in this group survived until the end of the study without clinical symptoms. Histopathological analysis will characterize the response of the lung parenchyma and cellular actors involved in the development of radiation-induced fibrosis. These preliminary results suggest that pMBRT minimizes the development of radiation-induced lung fibrosis: the gain of normal tissue tolerance is also present in lung irradiation. This opens the door for pMBRT in moving targets.

### P 024 - The effect of ultra-High Dose-Rate Carbon ion irradiation to cell invasion on breast tumor cells

#### Karin Oniwa^1^, Kazumasa Minami^1^, Masashi Yagi^2^, Shinichi Shimizu^2^, Masahiko Koizumi^1^, Kazuhiko Ogawa^1^, Tatsuaki Kanai^3^

##### ^1^Osaka University Graduate School of Medicine, Radiation Oncology, Suita, Japan ^2^Osaka University Graduate School of Medicine, Department of Carbon Ion Radiotherapy, Suita, Japan ^3^Osaka heavy Ion Therapy Center, Physics, Osaka, Japan

Currently, the normal tissue sparing and local tumor control at ultra-High Dose-Rate (uHDR) irradiation have been reported with particle and photon. Our group is interested in the effect of radiation to metastatic potentials on irradiated cells. We focused on the comparison of cell invasive capabilities between uHDR and conventional irradiated cells with carbon ion beam on breast cancer cell lines. Triple negative human breast cancer cell line MDA-MB-231 and estrogen positive human breast tumor cell line MCF-7 were used. The cells irradiated at uHDR (> 90 Gy/sec) and conventional dose rate (CDR) (1.16 Gy/sec) with carbon ion beam. Matrigel invasion assays were conducted using living cells after 24 hours from irradiation. At uHDR irradiation of 1.6 Gy compared to non-irradiation (0 Gy), the invasion cells were significantly reduced both cell lines. The inhibited ratios of invasive capabilities with uHDR on MDA-MB-231 and MCF-7 were 73.3% and 77.7%, respectively. The inhibited ratios of invasive capabilities with CDR on MDA-MB-231 and MCF-7 were 3.94% and 20.1%, respectively. We confirmed the cellar invasive potential in human breast cancer cell lines by uHDR and CDR irradiation with carbon ion beams. Our results suggested there was a significantly difference in the effect to cell invasive capability between uHDR and CDR. Remarkably, even if low dose can inhibit the one of the metastatic potentials on breast cancer cells with uHDR. We are studying the molecular mechanism in this phenomenon and would like to discuss in this presentation.

### P 025 - Investigating the DNA damaging effects of ultra-high vs conventional dose rates via proton irradiation of plasmids

#### Abigail Hemming^1^, Karen Kirkby^1^, Michael Merchant^1^, Nicholas Henthorn^1^, John Warmenhoven^1^

##### ^1^The University of Manchester, PRECISE group within the faculty of biology medicine and health, Manchester, United Kingdom

The aim of this study is to use the plasmid DNA nicking assay approach as a reductionist technique to investigate the differential DNA damaging effects of proton irradiation at ultra-high and conventional dose rates. PBR322 plasmid samples, diluted to a concentration of 100 ng/μl, were irradiated with doses of 0, 5, 15, 30, 45 and 60 Gy at both conventional (0.03 Gy/s) and ultra-high (74.15 Gy/s) dose rates using 245 MeV protons. Irradiated and control samples were then analysed through agarose gel electrophoresis and DNA damage yields were quantified using a published fitting method by Mcmahon et al. The results of this experiment showed that the samples irradiated at the ultra-high dose rate contained notably fewer single strand breaks than the conventionally irradiated samples. However, no substantial difference in the number of double strand breaks created was seen between the two dose rates. A plasmid system is a viable and valuable option to assess DNA damage, with adequate sensitivity to detect differential DNA damage. Our work probes the effect of rultra-high dose rates in terms of DNA damage, removing cofounding factors found in in vitro and in vivo work. Following on from this we plan to investigate the effect of including hydroxyl radical scavengers such as TRIS and DMSO within our samples as well as more biologically relevant scavengers including thiols e.g. glutathione. We will also explore the effect of differing oxygen concentrations on the DNA damage yields at both dose rates.

### P 026 - FLASH maintains anti-leukemic cytotoxicity of proton radiation

#### Mithun Sarangdhar^1^, Eric OBrien^1^, Caroline Theile^1^, Brett VanCauwenbergh^1^, Michael Lamba^2^, Dan Ionascu^2^, Mayur Sarangdhar^1,3^, Stefanno Nunez^1,2^, Anthony Mascia^1,2^, John Perentesis^1^

##### ^1^Cincinnati Children’s Hospital Medical Center, Cancer and Blood Diseases Institute CBDI - Oncology, Cincinnati- OH 45229, USA ^2^College of Medicine- University of Cincinnati, Department of Radiation Oncology, Cincinnati- OH 45267, USA ^3^Cincinnati Children’s Hospital Medical Center, Division of Biomedical Informatics, Cincinnati- OH 45229, USA

**Background**: Radiation remains the most potent modality for the treatment of chemotherapy drug-resistant leukemias. However, damage to tissues in the radiation field limits use. FLASH may widen the therapeutic window with improvement of the normal tissue complication probability (NTCP). Radiation therapies are part of standard of care approaches for the treatment of newly diagnosed or drug-resistant lymphomas, leukemic central nervous system disease and chloromas. Early reports with electron FLASH have suggested potential genetic determinants of sensitivity or resistance. We studied the relative antileukemic efficacy of proton FLASH vs conventional dose-rate proton (CONV) vs X-ray, and to identify potential sensitivity/resistance mechanisms.

**Methods and Findings**: We investigated the *in-vitro* dose-response of proton FLASH versus CONV protons and X-rays on a genetically characterized panel of acute myeloid leukemia (AML) and B-lymphoblastic leukemia (B-ALL) representing 13 World Health Organization subtypes. We calculated normalized AUC/exposure cytotoxicity values with doses of 0 (control), and 5-, 8-, and 16-Gy. AML and B-ALL cell lines responded to all 3 forms of radiation in a dose-dependent manner. The average relative biological effectiveness (RBE) of proton compared to X-ray was 1.11. We observed genetic signatures of resistance/sensitivity to X-ray and proton radiation (Figure 1). There was no statistically significant difference in the average cytotoxicity of proton FLASH versus proton CONV across the subtypes. Mechanistic studies utilizing metabolomic profiling and gene expression will be presented.

**Summary**: FLASH proton radiotherapy maintains effective and potent cytotoxicity compared to conventional proton and x-ray. Notable proton sensitivity was identified in chemotherapy drug-resistant KMT2A-rearranged leukemias.

### P 027 - A quick circuit of management and assessment of proton therapy indications in public centers of the Valencian Region

#### Antonio Jose Conde-Moreno^1^, Adela Cañete^2^, Erika Collado^3^, Carlos Ferrer^4^, Jose Gomez-Codina^5^, Sagrario Hernando^6^, Maria Tasso^7^, Gabriel Vazquez^8^, Jose Jaime Verdu^9^, Jose Perez-Calatayud^10^, Eduardo Ferrer^11^

##### ^1^Hospital Universitario y Politécnico La Fe, Radiation Oncology, Valencia, Spain ^2^Hospital Universitario y Politécnico La Fe, Pedriatic Oncology, Valencia, Spain ^3^Hospital Universitario y Politécnico La Fe, Radiation Oncology, Valencia, Spain ^4^Consorcio Hospitalario Provincial de Castellon, Radiation Oncology, Castellon, Spain ^5^Hospital Universitario y Politécnico La Fe, Medical Oncology, Valencia, Spain ^6^Conselleria de Sanitat Universal i Salut Pública, Servicio de Planificacion de Programas y Servicios Sanitarios, Valencia, Spain ^7^Hospital General Universitario de Alicante, Pediatric Oncology, Alicante, Spain ^8^Hospital Universitario San Juan, Radiation Oncology, Alicante, Spain ^9^Hospital Clínico Universitario de Valencia, Pediatric Oncology, Valencia, Spain ^10^Hospital Universitario y Politécnico La Fe, Medical Physics-Radiation Oncology, Valencia, Spain ^11^Hospital Clinico Universitario, Radiation Oncology, Valencia, Spain

**Objective**: The scientific societies and the Spanish Ministry of Health have established indications for the use of proton therapy in our country. Establishing an efficient referral procedure is a fundamental need.

**Material and Methods**: In the Valencian Region a data safe circuit and a multidisciplinary, multi-centric Evaluation Committee integrated by Radiation Oncologists, Medical and Pediatric Oncologists, Medical Physicist and government staff has been established to evaluate candidates to proton therapy. After the indication assessment within the local tumor boards of each referring center, requests are sent by the local Radiation Oncologist to the Central Services of the Department of Health of our region. This Department up-load the request to a central corporative virtual platform (CONCERTS) for assessment by the Evaluating Committee. The Clinical Documentation and Admission Service of each Department of Health is notified in the same day so that the patient can be evaluated at the destination center. Decisions are communicated via corporative email by each member of the Evaluation Committee. The whole process comply with the current data protection law.

**Results**: Between January and December 2022, 47 patients were evaluated (24 paediatric and 23 adult), of whom 45 met clinical indications for proton therapy. The multidisciplinary assessment process and issuance of reports did not exceed 48 hours.

**Conclusions**: Clear communication of the indications and the request process, to the previous multidisciplinary assessment of the same in the Tumor Committees, and secure circuit through a virtual communication platform, allows avoiding delays and optimizing the objectives of proton treatment.

### P 028 - A study on dosimetric impact and tumor control probability of proton versus intensity-modulated radiotherapy for breast cancer regional nodal irradiation

#### Tien Yee Amy Chang^1^, Bin Yang^2^, Wing Lun Alan Mui^3^, Sze Wing Miranda Poon^3^, Wai Wang Tomie Lam^2^, Wing Ki Winky Fung^3^, Siu Ki Ben Yu^2^, George Chiu^3^, Koon Ming Michael Kam^1^, Chun Chung Yau^1^, William Wong^4^

##### ^1^Hong Kong Sanatorium & Hospital, Comprehensive Oncology Centre, Hong kong, Hong Kong ^2^Hong Kong Sanatorium & Hospital, Medical Physics Department, Hong kong, Hong Kong ^3^Hong Kong Sanatorium & Hospital, Department of Radiotherapy, Hong kong, Hong Kong ^4^Mayo Clinic Arizona, Department of Radiation Oncology, Arizona, USA

**Purpose**: For patients receiving adjuvant irradiation to regional lymph nodes (RNI) for high-risk breast cancer, volumetric modulated arc therapy (VMAT) or helical tomotherapy (HT) may provide improved coverage of internal mammary chain (IMC) and sparing of normal tissues compared to 3D-conformal radiation therapy (3DCRT). Intensity modulated proton therapy (IMPT) potentially further reduce heart and lung doses. This study aims to compare dosimetry and tumor control parameters of RNI in various modalities.

**Methods**: Twenty patients were included. Clinical target volumes (CTVs) include chest wall, whole or reconstructed breast, supraclavicular fossa (SCF), IMC, and level III axillary chain. Target volumes were treated with 40.05Gy in 15 fractions, and re-optimized with partial arcs VMAT, HT and IMPT. Doses for optimal target coverage and organs at risk (OARs) were compared, and tumor control probability (TCP) and normal tissue complication probability (NTCP) were measured.

**Results**: VMAT, HT and IMPT achieved at least 90% prescribed doses to target except 3DCRT plans (V38Gy 94.63%, 94.3%, 95.5% and 86.02% respectively; V36Gy 97.62%, 97.1%, 97.17% and 93.19% respectively; p<0.0001). IMPT achieved significant reduction in mean heart dose, and contralateral breast and lung sparing. Mean dose to heart were 4.25Gy (VMAT), 4.44Gy (HT) and 1.28Gy (IMPT; p<0.01). TCP for 3DCRT is inferior (29.57%) compared with VMAT, HT and IMPT (99.82%, 99.80%, 95.82%, respectively, p< 0.001). NCTP to ipsilateral lung was higher for 3DCRT (10.58%) and VMAT (5.79%) and lower for HT and IMPT (4.58% and 4.12% respectively, p<0.0001).

**Conclusions**: IMPT achieves the most significant heart, lung, and contralateral breast sparing with favorable tumor control parameters.

### P 029 - Proton therapy: A new standard care for pregnant cancer patients?

#### Menke Weessies^1^, Murillo Bellezzo^1^, Britt Hupkens^1^, Frank Verhaegen^1^, Gloria Vilches-Freixas^1^

##### ^1^Maastro, Radiation Oncology, Maastricht, Netherlands

**Introduction**: Cancer during pregnancy is fortunately uncommon. However, the incidence is increasing due to the rising average age of pregnancy. The interest in treating pregnant patients with protons is growing due to the reduced out-of-field dose in proton pencil beam scanning (PBS) compared to photon radiotherapy (1-4). Especially, the estimation of the neutron-dominated out-of-field radiation remains a challenge. This study aims to develop a platform to determine for which pregnant patients PBS will yield sufficient benefit above photons.

**Methods and Materials**: We developed an experimental setup consisting of the Rando–Alderson anthropomorphic phantom where some slabs were substituted by PMMA slabs with holes to allocate bubble detectors and films at different distances and depths. In the abdominal area, additional slabs were to mimic 20, 25 and 30 weeks of gestational age belly (Fig1). To measure the out-of-field neutron dose we used bubble detectors. Proton treatment plans were created in RayStation 11A. A Monte Carlo (MC) simulation platform for the prediction of out-of-field doses to the fetus was developed.

**Results**: To have a reliable neutron dose estimate, the whole treatment room has to be included in the MC simulations. For a breast cancer plan at a distance between 7.5-15cm corresponding to 30-20 weeks of gestational age, the fetus will receive ∼0.002% of the target dose, from which >85% originates from neutrons.

**Conclusion**: We developed an experimental and MC platform with a modified anthropomorphic phantom that could be used to quantify the eventual benefit of PBS for a wide range of indications.

### P 030 - Advanced Proton therapy approaches: A multicenter high-quality data registry

#### Daniela Alterio^1^, Maria Giulia Vincini^1^, Sara Gandini^2^, Giulia Peruzzotti^3^, Mattia Zaffaroni^1^, Luca Bergamaschi^1^, Barbara-Alicja Jereczek-Fossa^1,4^, Roberto Orecchia^5^

##### ^1^IEO European Institute of Oncology, Division of Radiation Oncology, Milan, Italy ^2^IEO European Institute of Oncology, Molecular and Pharmaco-Epidemiology Unit- Department of Experimental Oncology, Milan, Italy ^3^IEO European Institute of Oncology, Data Management Unit, Milan, Italy ^4^IEO European Institute of Oncology- University of Milan, Division of Radiation Oncology- Department of Oncology and Hemato-oncology, Milan, Italy ^5^IEO European Institute of Oncology, Scientific Directorate, Milan, Italy

Paucity and low evidence level data on protontherapy (PT) represent one of the main issues for the establishment of solid indications in the PT setting. Aim of the present registry is to provide a tool for systematic, prospective, harmonized, and multidimensional high-quality data collection to promote knowledge in the field of PT with a particular focus on the use of hypofractionation. All patients with any type of oncologic disease (benign and malignant disease) who will be eligible for PT at the European Institute of Oncology (IEO), will be included in the present registry. Three levels of data collection will be implemented: Level 1 clinical research (patients outcome and toxicity, quality of life, and cost/effectiveness analysis); Level 2 radiological research (radiomic and dosomic analysis, as well as biological modeling); Level 3 Biological and Translational research (biological biomarkers and genomic data analysis). A summary of collected data and possible research lines is provided in Figure 1. Endpoints and outcome measures of hypofractionation schedules will be evaluated in terms of either Treatment Efficacy (tumor response rate, time to progression/percentages of survivors/median survival, clinical, biological, and radiological biomarkers changes, identified as surrogate endpoints of cancer survival/response to treatment) and Toxicity. The study protocol has been approved by the IEO ethical committee (IEO 1885). Other than patients treated at IEO, additional PT facilities (equipped with IBA Proteus®ONE or Proteus®PLUS technologies) are planned to join the registry data collection. Moreover, the registry will be also fully integrated into international PT data collection networks.

### P 031 - Audit of motion management strategies in Pencil Beam Scanning Proton Beam Therapy for moving tumors: PROMO prospective registry

#### Anupamma Reddy^1^, Srinivas Chilukuri^1^, Ashwathy Mathews^1^, Sham Sundar^1^, Sapna Nangia^1^, Ashok Reddy Karra^1^, Kartikeshwar Patro Ch^1^, Manikandan arjunan^1^, Dayananda Sharma^1^, Rakesh Jalali^1^, Minnal Ms^1^, Sanjib Gayan^1^

##### ^1^Apollo Proton Cancer Centre, Radiation Oncology, Chennai, India

**Purpose**: To audit motion management strategies(MMS) used in patients with moving tumors treated with pencil beam scanning proton therapy(PBS-PBT) as part of a prospective registry.

**Methods**: Fifty-one consecutive patients with moving tumors underwent planning CT and 4DCT scans acquired using surface tracking. Plans were generated using robust optimization to ITV/iGTV for 3-5mm setup error, 3.5-4% range uncertainty and were evaluated for 4D robustness. Patients were treated using daily CBCT and continuous surface tracking during treatment delivery. Quality-assurance(QA) CTs were acquired to assess need for adaptive re-planning.

**Results**: Patient, tumor characteristics and motion mitigation strategies are summarized in Figure-1 and Table-1. 24 patients(47%) and 27 patients(53%) were treated in breath-hold(BH) and free-breathing(FB) respectively and 5 patients(10%) were treated with compression-belt(CB). ITVs were generated in 80% of patients. Two-thirds had MFO plans, and their frequency did not vary between FB and BH. 59%(16 patients) of FB plans and 13%(3 patients) of BH plans were treated with volumetric repainting. Adaptive re-planning was performed in 21 patients(41.2%) and 33% of them required it more than once. 5 patients(10%) required change in MMS during treatment owing to patient/setup related causes. Mean of the individual median treatment times for BH and FB was 30±5min(11-85) and 32±10(11-89) respectively. All except one patient completed their planned dose of treatment and none of the patients had acute grade 3 toxicities.

**Conclusion**: MMS workflow was successfully implemented for moving tumors using daily CBCTs and surface guidance with acceptable treatment times. Need for adaptive re-planning is considerably high in these patients.

### P 032 - Pediatric treatments in the upright orientation

#### Shane Ikner^1^, Jemma Nunn^2^, Ross Thierney^3^, Tracy Underwood^4^, Stephen Towe^4^, Rock Mackie^5^, Niek Schreuder^6^

##### ^1^Leo Cancer care, Therapy, Middleton, USA ^2^Leo Cancer Care, Therapy, Smallfield- Horley, United Kingdom ^3^Leo Cancer Care, Engineering, Smallfield- Horley, United Kingdom ^4^Leo Cancer care, Medical Physics, Smallfield- Horley, United Kingdom ^5^Leo Cancer Care, Medical Physics, Middleton, USA ^6^LEO Cancer Care, Medical Physics, Knoxville, USA

Leo Cancer Care (see www.leocancercare.com) is developing technologies that will enable positioning patients in an upright orientation for imaging and radiation therapy. The clinical benefits of upright imaging and radiotherapy treatments are numerous and are well documented [1, 2, 3]. The first evidence of the important clinical benefits using the system to position real patients in a test setting was recently documented [4]. The question that are often raised is whether pediatric patients can be treated in the upright orientation. This presentation will address the feasibility of treating pediatric patients in the upright position looking at three categories i.e. (1) pediatric patients that are tall enough to fit the adult patient profile per the system design specifications, (2) smaller pediatric patients that does not require anesthesia and (3) pediatric patients that require anesthesia. We will discuss design solutions based on numerous pediatric positioning exercises we conducted using the LEO upright patient positioner. We will also discuss the feasibility of administering anesthesia to patients in the upright orientation.

### P 033 - Patients’ needs in proton therapy: A survey among 10 european facilities

#### Giovanni Carlo Mazzola^1^, Bergamaschi Luca^1^, Cristiana Pedone^2^, Maria Giulia Vincini^1^, Matteo Pepa^3^, Mattia Zaffaroni^1^, Stefania Volpe^1^, Barbara Rombi^4^, Jerome Doyen^5^, Piero Fossati^6^, Karin Haustermans^7^, Morten Hoyer^8^, Hans Langendijk^9^, Raul Matutue^10^, Ester Orlandi^3^, Hyllevi Rylander^11^, Esther Troost^12^, Roberto Orecchia^1^, Daniela Alterio^1^, Barbara Alicja Jereczek-Fossa^1^

##### ^1^European Institute of Oncology, Radiation Oncology, Milan, Italy ^2^University of Milan, Oncology and Emato-Oncology, Milan, Italy ^3^Centro Nazionale di Adroterapia Oncologica CNAO, Radiationtherapy, Pavia, Italy ^4^Trento Protoncenter, Radiationtherapy, Trento, Italy ^5^Centre Antoine-Lacassagne, Radiationtherapy, Nice, France ^6^MedAustron Ion Therapy Center, Radiationtherapy, Wien, Austria ^7^Karin Karin Haustermans, Radiationtherapy, Leuven, Belgium ^8^Danish Centre for Particle Therapy, Radiationtherapy, Aarhus, Denmark ^9^University Medical Center Groningen, Radiationtherapy, Groningen, Netherlands ^10^Centro de Protonterapia Quironsalud, Radiationtherapy, Madrid, Spain ^11^Skandionkliniken proton facility, Radiationtherapy, Uppsala, Sweden ^12^University Hospital Carl Gustav Carus, Radiationtherapy, Dresden, Germany

**Aim**: Proton Therapy (PT) facilities are still in limited number worldwide, limiting the access to treatment for patients. Aim of the present survey was to assess the support provided to patients undergoing PT across Europe.

**Methods**: A questionnaire was distributed to ten European PT centres. The questionnaire consisted of 62 questions divided into six sections: i) personal data; ii) information on clinical activity; iii) fractionation, concurrent systemic treatments and technical aspects; iv) indication to PT and reimbursement policies; v) economic and/or logistic support to patients vi) possible limitation of access to PT. A qualitative analysis was performed.

**Results**: Nine centres treating from 100 to 500 patients per year participated in the survey. Paediatric patients account for 10-30% for 7 centres. Most frequent adult tumours treated are brain tumours, sarcomas and head and neck carcinomas. Mean treatment duration is 3 weeks. Health National System (HNS) provides reimbursement for PT treatment in 80% of cases as well as economic, logistic and meal supports to patients in 70%, 20% and 40% of centres. PT facilities offer economic and/or logistic support to patients in 90% of the cases while parents of paediatric patients are supported in one-third of the centres. Seventy percent of respondents agree that geographic challenges may limit patient’s access to proton facilities and 60% believe that additional support should be given.

**Conclusion**: Considering the differences among European countries, further efforts should be made to support patients’ economic and logistic issues in access to PT care.

### P 034 - Fetal dose assessment comparing proton PBS to photon 3DCRT and VMAT in a pregnant patient with brain tumor

#### Robabeh Rahimi Darabad^1^, Michael Taylor^1^, Grayden MacLennan^1^, Lienard Chang^1^, Peng Wang^1^, Chawla Ashish^1^, Fan James^1^, Kuan Ling Gwen Chen^1^

##### ^1^Inova Schar Cancer Institute, Radiation Oncology, Fairfax, USA

At our center, a 33-year-old female who was 24 weeks pregnant and had prior RT to brain in 2020 presented with recurrent astrocytoma. Adjuvant radiation was indicated. Proton therapy is normally preferred for reirradiations; however there are hesitations in the use of protons for pregnant patients (due to neutron risk to the fetus). We did pre-treatment study and compared photon (3DCRT and VMAT) and proton PBS, Picture 1-“PreTx measurements”. Planning techniques were employed to reduce scatter and leakage dose for photon treatment and neutron dose for proton treatment. Patient specific radiation shielding was built for photon, but no shielding was needed for proton, besides shielding for imaging dose. Measurements were done using Wendii and track etch detectors, sensitive to photons and neutrons (thermal and fast). Proton therapy was demonstrated to be superior for our pregnant patient compared to photon therapy, since the out-of-field dose was reduced, in addition to the optimum dose distribution achieved in the target region, Picture 2-“PreTx measurements results”. The patient successfully completed the full course of proton treatment. She subsequently delivered a healthy baby at full term with no complications. Future considerations will include the evaluation of patients with tumors closer to fetus and/or patients with attached electronic devices with PBS proton treatment. Proton treatment, specially a PBS system, can practically be proven to be superior for all such cases, therefore we believe that traditional hesitation to the use of neutron-generated particles may need to be revisited to ensure optimal individual care to our patient population.

### P 035 - Cost-effectiveness of proton beam therapy versus conventional radiation therapy for unresectable pancreatic cancer

#### Yuichi Hiroshima^1,2^, Kondo Masahide^3^, Takuya Sawada^1^, Shu-ling Hoshi^3^, Reiko Okubo^3^, Takashi Iizumi^1^, Haruko Numajiri^1^, Toshiyuki Okumura^1^, Hideyuki Sakurai^1^

##### ^1^University of Tsukuba, Department of Radiation Oncology & Proton Medical Research Center- Faculty of Medicine, Tsukuba, Japan ^2^Ibaraki Prefectural Central Hospital- Ibaraki Cancer Center, Radiation Oncology, Kasama, Japan ^3^University of Tsukuba, Department of Health Care Policy and Health Economics- Faculty of Medicine, Tsukuba, Japan

**Purpose**: Pancreatic cancer is one of the most common malignant tumors and has a poor prognosis. Recently, an increasing number of reports have used proton beam therapy (PBT) to treat unresectable, locally advanced pancreatic cancer (LAPC). However, the cost-effectiveness of PBT for pancreatic cancer has not been evaluated. This study aimed to determine the cost-effectiveness of PBT for LAPC as a replacement for conventional photon radiotherapy (RT).

**Material and Methods**: We estimated the incremental cost-effectiveness ratio (ICER) of PBT as a replacement for RT, using clinical evidence in the literature and expense complemented by expert opinions. We used a decision tree and an economic and Markov model to illustrate the disease courses followed by LAPC patients. Effectiveness was estimated as quality-adjusted life years (QALY) using utility weights for the health state. Social insurance fees were calculated as the costs. The stability of the ICER against the assumptions made was appraised using sensitivity analyses.

**Results**: The effectiveness of PBT and RT was 1.67610615 and 0.97181271 QALY, respectively. The ICER was estimated to be ¥5,376,915 (US$46,756) per QALY. According to the suggested threshold for anti-cancer therapy from the Japanese authority of ¥7,500,000 (US$65,217) per QALY gain, such a replacement would be considered cost-effective. The one-way and probabilistic sensitivity analyses demonstrated stability of the base-case ICER.

**Conclusion**: PBT, as a replacement for RT, is cost-effective and justifiable as an efficient use of finite healthcare resources. Making it a standard treatment option and available to every patient in Japan is socially acceptable from the perspective of health economics.

### P 036 - Quantitative evaluation of using HD shielding modules to address the problem of the decommissioning of proton therapy facilities

#### El Hassane Bentefour^1^, Deeoak Samuel^2^, Robert Farrell^3^

##### ^1^Veritas Medical Solutions, Physics and Radiation Shielding, Harleysville, USA ^2^Central University of Karnataka, Department of Physics, Karnataka, India ^3^Veritas Medical Solutions-, Radiation shielding, Harleysville, USA

The expected life cycle of proton therapy systems may be estimated around 30 years. After this period, the facility decommissioning may need to be addressed. The decommissioning of a facility concerns the accelerator and the related equipment as well as the accelerator vault. After beam extraction from the accelerator, the full energy beam is transported to the treatment room. Beam losses occur along the beam transport line due to the interactions of the protons with the different transport line elements and in the patient during the treatment. These beam interactions generate secondary neutrons which can cause material activation within the facility’s shielding walls and equipment. The material activation originates form a series of nuclear reactions that produce radioactive isotopes with half-lives ranging from hours to many years. The long-lived isotopes, such as 152Eu, 133Ba,134Cs, 60Co and 22Na, are particularly problematic for the decommissioning of the facility because in addition to their long half-life, they require very low Clearance Index (CI) to qualify as non-nuclear waste. In this work, we review the shielding capability of Verishield, an engineered high density shielding module, against fast and thermal neutrons in comparison to regular concrete. Then, we explore the extent of the long-term neutron activation problem for various formulas of these Verishield modules with different content of cement. Last, we discuss and compare the cost effectiveness of building and decommissioning a proton therapy facility when using respectively Verishield and concrete.

### P 037 - Efficient use of treatment room

#### Sebastian Makocki^1^, Julia Thiele^1^, Stefan Menkel^1^, Esther G. C. Troost^1,2,3,4,5^, Christian Richter^1,2,3,4^

##### ^1^Department of Radiotherapy and Radiation Oncology, Faculty of Medicine and University Hospital Carl Gustav Carus- Technische Universität Dresden, Dresden, Germany ^2^OncoRay – National Center for Radiation Research in Oncology, Faculty of Medicine and University Hospital Carl Gustav Carus- Technische Universität Dresden- Helmholtz-Zentrum Dresden - Rossendorf, Dresden, Germany ^3^Helmholtz-Zentrum Dresden - Rossendorf, Institute of Radiooncology – OncoRay, Dresden, Germany ^4^German Cancer Consortium DKTK- Partner Site Dresden, and German Cancer Research Center DKFZ, Heidelberg, Germany ^5^National Center for Tumor Diseases NCT- Partner Site Dresden- Germany: German Cancer Research Center DKFZ- Heidelberg- Germany, Faculty of Medicine and University Hospital Carl Gustav Carus- Technische Universität Dresden- Dresden- Germany- and- Helmholtz Association/Helmholtz-Zentrum Dresden - Rossendorf HZDR, Dresden, Germany

**Background**: In recent years, we have observed a continuous increase in clinics offering particle therapy, allowing a substantial number of patients to benefit from the advantages of this therapy. The capacity of particle therapy is noticeably limited in comparison to conventional radiotherapy; however, efficient use of the existing treatment rooms permits treatment for the utmost number of patients.

**Methods**: PTCOG patient statistics for 2020 were used to calculate treated patients per room and year for 90 institutions.

**Results**: The range of treated patients was 12 – 567 patients per room and year, with a median of 133. With 242 treated patients, our centre holds the 10^th^ place in the evaluation and places 3^rd^ among academic centres. Some of the centres with significantly lower patient numbers have recently become operational - the number of patients should increase in the upcoming years.

**Discussion**: Our clinic has been treating patients since 2014 and was able to significantly increase the number of patients treated ever since. Of note, 95% of the patients are treated within clinical studies, which often require complex techniques or additional treatment effort. Several time-saving steps have been implemented. The anaesthesia for paediatric patients is initiated outside of the treatment room and monitored with a mobile unit during the irradiation. Installation of a virtual hand pendant saved a considerable amount of time in quality assurance (QA). Using self-designed phantoms allows for simultaneous measurements of various beam parameters and keeps the morning QA efficient.

### P 038 - Clinical validation of an automated system for cranial locations contouring based on magnetic resonance imaging (MRI): A single institution experience

#### Marta Montero Feijoo^1^, Juan Antonio Vera Sánchez^2^, Stephanie Bolle^1^, Morena Sallabanda Hajro^1^, Ana de Pablo Gonzalez^1^, Fernando Cerron Campoo^2^, Juan María Pérez Moreno^2^, Juan Castro Novais^2^, Raul Matute Martin^1^, Raymond Miralbell Izard^1^, Norina Predescu^3^, Jarkko Niemela^4^

##### ^1^Centro de Protonterapia Quironsalud, Radiation Oncology, Madrid, Spain ^2^Centro de Protonterapia Quironsalud, Medical Physicist, Madrid, Spain ^3^MVision AI, Product Clinical Quality Manager, Helsinki, Finland ^4^MVision AI, Chief Product Officer, Helsinki, Finland

The aim of this work is providing clinical information on an automated system for contouring cranial locations based on MRI. Cranial malignancies are one of the most frequent indications for protontherapy. Precise delineation is mandatory to preserve them if feasible. We have collaborated in the development of the cranial model for automatic contouring using artificial intelligence (AI). A set of 68 patients with brain or head and neck MRI and radiotherapy CT simulation scans with treatment structure sets have been provided. Prior to submission, each structure set has been reviewed. After MVision AI tested their existing model on provided MRI, 43 scans with the poorest performance, was used for manual annotations according to the provided clinical segmentations following EPTN contouring guidelines. All approved annotations were added to the pre-existing datasets for model retraining and improvement. Following training the model, clinical validations are being performed through clinical judgement by independent MD experts. These validations consist of independent evaluation by three MD experts who score the overall performance of the system and measure individual deviations when detected. On first clinical cases, dosimetric comparison between the expert delineation and the automated solution will be performed to adequately address the clinical dosimetric impact. The expected results of this solution are the reduction of time during treatment preparation, the reduction of inter-observer variability in the contouring process and the homogenisation of the delineation of cranial structures in future clinical trials. Our first results based on clinical judgement show promising results in achieving the desired outcomes.

### P 039 - Equity of access to proton beam therapy in England: A national NHS analysis

#### Simona Gaito^1,2,3^, Anna France^3^, Marianne Aznar^2^, Adrian Crellin^4^, Karen Kirkby^1,2^, Shermaine Pan^1^, Gareth Price^2^, Peter Sitch^3^, Gillian Whitfield^1,2^, Ed Smith^1,2,3^

##### ^1^The Christie NHS FT, Proton Beam Therapy Centre, Manchester, United Kingdom ^2^University of Manchester, Division of Clinical Cancer Science- School of Medical Sciences, Manchester, United Kingdom ^3^The Christie NHS FT, Proton Clinical Outcomes Unit, Manchester, United Kingdom ^4^NHS England, Lead of NHSe Proton Beam Therapy service, Manchester, United Kingdom

**Introduction**: Policies to improve population health have often focused on equitable access to health services. While new technologies have an enormous potential in improving health outcomes, they may not always be equally accessible across diverse geographical areas and socio-economic backgrounds. Aim of this **study is to evaluate equity of access to PBT throughout the country and how this has changed since the national PBT service inception**.

**Methods**: Incidence data were provided by the National Disease Registration Service (NDRS). The incidence data referred to the time period 2013-2019. The first national NHS PBT centre started accepting referrals in October 2018, therefore this time period was split in pre-NHS PBT (1/1/13-30/9/18) and post-NHS PBT (1/10/18-31/12/19). Demographics and clinical characteristics of patients referred for PBT were extracted from the national NHS PBT registry. The ratio between the referred and newly diagnosed patients is the Proton Utilisation Proportion (PUP).

**Results**: The breakdown of incidence and referral statistics is given in Table 1 for the pre- and post-NHS PBT periods. Figure 1 shows the PUP pre- and post-NHS PBT and the percentage increase post-NHS PBT by region. Of note, post-NHS PBT, 99% of the patients aged 0-15 with these 7 common indications for PBT were successfully treated with PBT.

**Conclusions**: Promoting equality of access to cutting-edge radiotherapy technologies is at the heart of NHS England’s values. The PUP has increased since the opening of a National PBT service in England. Further analysis will follow to examine whether socio-economic or geographical barriers exist within each region.

### P 040 - Treatment planning analytics for single room proton facility

#### Rex Cardan^1^, James Snider^2^, Jim Kraus^1^, Joseph Harms^1^, Adam Kole^1^, Richard Popple^1^, John Fiveash^1^

##### ^1^University of Alabama at Birmingham, Radiation Oncology, Birmingham, USA ^2^South Florida Proton, Radiation Oncology, Delray, USA

**Introduction**: The business model for proton therapy centers depends strongly on accurate and realistic metrics for planning, evaluation and delivery. The purpose of this work is to provide planning workload data from a single room proton center at an academic institution.

**Methods**: Patient plans from a new single room proton center operating 16 hours daily were tracked using a custom web application that was developed to model the flow of patients from simulation to treatment. Data was captured between 18-33 months after first patient. Time points were recorded when planning was started, when plans were ready for evaluation by physics and when plans were approved for treatment by the physician. Planning tasks were distributed among dosimetrists without regard to treatment site. For analysis, plans were categorized by treatment site and dosimetrist, and timespans were recorded as total hours including non-working hours.

**Results**: Over 15 months, 359 plans were analyzed across 4 dosimetrists over 10 distinct treatment sites including head and neck (43.4%), brain (14.5%) prostate (11.7%), chest wall (7.5%), and breast (5.3%). The average ± SD total planning time for each of these was 113.2 ± 143.6 hrs, 92.6 ± 137.2 hrs, 69.2 ± 106.1 hrs, 166.3 ± 149.9 hrs, and 280.6 ± 339.4 hrs respectively. The dosimetrist median planning times were 101.7 hrs (N=54), 34.28 hrs (141), 71.0 hrs (N=84) and 103.9 hrs (N=65).

**Conclusion**: The data collected detailed workload statistics which might be useful for modeling future proton centers.

### P 041 - Intensity-modulated proton radiotherapy spares musculoskeletal structures in regional nodal irradiation for breast cancer: A dosimetric comparison study

#### Jessica Burlile^1^, Kevin Brom^2^, Heather Gunn^3^, Jon Gustafson^2^, Cassandra Sonnicksen^4^, Satomi Shiraishi^2^, Rachel Jimenez^5^, Krishan Jethwa^1^, Kimberly Corbin^1^

##### ^1^Mayo Clinic, Department of Radiation Oncology, Rochester, USA ^2^Mayo Clinic, Department of Medical Physics, Rochester, USA ^3^Mayo Clinic, Qualitative Health Sciences, Rochester, USA ^4^Mayo Clinic, Department of Radiation Oncology- Dosimetry, Rochester, USA ^5^Massachusetts General Hospital, Department of Radiation Oncology, Boston, USA

**Background**: Restricted shoulder mobility after multi-modality therapy for breast cancer is common and may in part be due to radiation exposure to musculoskeletal structures. Utilizing more conformal intensity modulated radiotherapy (IMRT) en lieu of 3D-conformal radiotherapy may be associated with better functional outcomes, and intensity-modulated proton therapy (IMPT) could provide additional benefit. We performed a dosimetric comparison of IMPT and IMRT regional nodal irradiation (RNI) plans to quantify this difference, and hypothesized that IMPT would result in significant dose reduction to muscles of shoulder abduction and flexion.

**Methods**: Before examining any radiation plans or dose distribution, 14 patients with IMRT and IMPT plans were identified for contouring. Twenty musculoskeletal structures were contoured after consultation with a physical therapist specializing in breast cancer. Physical dose statistics were extracted using PROKnow DS and were converted to the equivalent dose in 2-Gy fractions (EQD2) assuming an alpha-beta ratio of 4. Dosimetry was compared using paired t-tests. The Bonferroni correction was applied to adjust for multiplicity (alpha=0.0003125).

**Results**: Dosimetric comparisons are displayed in Table 1. The mean dose delivered with IMPT was statistically significantly less for every structure except for the sternocleidomastoid. Relative dose reduction approached 100% for some structures. “Anterior” structures had fewer significant differences in dose compared to “posterior” structures, especially for the D5% (high dose) metric. Figure 1 shows a representative IMRT and IMPT plan.

**Conclusion**: Structures implicated in shoulder dysfunction after RNI can be spared with the use of IMPT. Further research is needed to correlate musculoskeletal structure dose-volume associates with patient-reported function.

### P 042 - Research on light flash during proton beam therapy and photon radiotherapy: A multicenter prospective study

#### Yoshiko Oshiro^1^, Masashi Mizumoto^2^, Sumiya Taisuke^2^, Takashi Iizumi^2^, Haruko Numajiri^2^, kei Nakai^2^, Hideyuki Sakurai^2^

##### ^1^Tsukuba University and Tsukuba Medical Center, Radiation Oncology, Tsukuba, Japan ^2^Tsukuba University, Radiation Oncology, Tsukuba, Japan

**Purpose**: To evaluate characteristics of light flashes during proton and photon radiotherapy.

**Methods and Materials**: A prospective observational study was performed in adult patients received proton and photon therapy at two institutes. During a radiotherapy, the presence, color, darkness, intensity, and duration of the color was evaluated using a checklist.

**Results**: A total of 650 courses for 621 patients were evaluated. 416 and 205 patients received photon and proton radiotherapy, and 88 (16%) felt light during the treatment. According to multivariate analysis, higher retina dose and younger age (p < 0.001) were significantly associated with presence of light flash, but there was no significant difference between proton and photon radiotherapy. Light flashes were frequently sensed for whom the retina was irradiated (56% (54/97) vs. 7% (33/524)). The median duration of light flash was 10 seconds. The color was varied white, blue, purple, yellow, red, white, and others, and numbers of patients who sensed these colors were 16, 52, 15, 15, 9, and 8, respectively (multiple selections possible). The blue and white light was frequently sensed in brain and head and neck radiotherapy, whereas, red, purple and yellow light was frequently sensed in radiotherapy to the body trunk.

**Conclusions**: The light flash is considered as so-called Cherenkov light, but the reason of color variation and light sense among the patients whose retina was not irradiated was unknown. The retina dose and a younger age were significantly associated with the frequency of light flashes during proton radiotherapy as well as photon radiotherapy.

### P 043 - Renal growth changes after proton beam therapy for pediatric tumor

#### Masashi Mizumoto^1^, Yinuo Li^1^, Yoshiko Oshiro^1^, Haduki Nitta^1^, Takashi Saito^1^, Takashi Iizumi^1^, Hirokazu Makishima^1^, Haruko Numajiri^1^, Kazushi Maruo^2^, Hideyuki Sakurai^1^

##### ^1^University of Tsukuba, Radiation Oncology, Tsukuba, Japan ^2^University of Tsukuba, Biostatistics, Tsukuba, Japan

**Purpose**: To analyze renal late effects after proton therapy (PT) for pediatric tumors.

**Method**: A retrospective study was performed in 11 pediatric patients (< 8 years) who received pelvic PBT between 2013 and 2018, and the treatment field included one kidney. The growth rate (GR: %/year) of both irradiated kidney volume (KV) and contralateral non-irradiated KV was calculated as follows: (KV at the follow up – KV at the treatment planning) ×12/follow up period (months)). Also, we calculated relative atrophy rate of irradiated kidneys as follows: [(KV*_irradiated_* at the treatment planning × (1 + GR_*contralateral*_)- KV_*irradiated*_ at the last follow up)]/[KV_*irradiated*_ at treatment planning × (1 + GR_*contralateral*_)]. Dose-volume relationship was also analyzed.

**Result**: The median follow-up was 24 months (range, 11-57 months). Kidney function was preserved in all patients. The median GR of irradiated kidneys and control contralateral kidneys were −8.55% (−47.52 to +15.51%) and +9.53% (−2.13 to +38.78%), respectively. The median relative atrophy rate relative to control kidneys was −16.42% (−52.21 to −4.53%). Kidneys irradiated with maximum doses of 20, 30, 40 and 50 GyE had volume reductions of 1.09%, 1.24%, 2.34% and 8.2% per irradiated volume, respectively. Volume reduction was much greater in patients aged 4-7 years than in those aged 2-3 years.

**Conclusion**: These results suggest that kidneys exposed to PBT in treatment of pediatric tumor show continuous atrophy in follow-up. The degree of atrophy is increased with a higher radiation dose, greater irradiated volume, and higher age.

### P 044 - Short-term of post-irradiation ear complications in patients of nasopharyngeal carcinoma treated with intensity-modulated proton beam therapy versus volume-modulated arc therapy

#### Kuang-Hsu Lien^1,2^, Tai-Yu Chen^1^, Kai-Ping Chang^1^, Joseph Tung-Chieh Chang^3^

##### ^1^Chang Gung Memorial Hospital- Linkou branch, Otolaryngology - Head & Neck Surgery, Taoyuan, Taiwan Province of China ^2^Chang Gung University, Graduate Institute of Clinical Medical Sciences- College of Medicine, Taoyuan, Taiwan Province of China ^3^Chang Gung Memorial Hospital- Linkou branch, Department of Radiation Oncology, Taoyuan, Taiwan Province of China

**Background**: We aimed to investigate the short-term of post-irradiation ear complications in patients of nasopharyngeal carcinoma(NPC) treated with intensity-modulated proton beam therapy (IMPT) versus volume-modulated arc therapy (VMAT).

**Methods**: We enrolled 14 NPC patients receiving IMPT and 41 NPC patients receiving VMAT between August 2020 to October 2021. All patients received pure tone audiometry (PTA), word-recognition scores (WRS), Tinnitus Handicap Inventory (THI) scores and meticulous clinical examinations by otologists when diagnosed with NPC and finished radiotherapy treatment for six months. The exclusion criteria consisted of (1) any head and neck cancer history (2) histories of ear irradiation or surgery, or (3) known hearing problems. We compared the PTA, WRS, THI scores, incidence of post-irradiation middle ear effusion, middle ear ventilation tube insertion and ear drum perforation between both groups.

**Results**: Before treatments, there were no differences between IMPT and VMAT in all indices. After finishing treatments for 6 months, the IMPT group showed lower THI scores than VMAT group (*p*<0.001). Otherwise, there were no significant differences between both groups in other indices. The subgroup analysis revealed that VMAT group had poorer performance than IMPT group in WRS in T3 stage (*p*=0.046). In AJCC stage II and III, VMAT group showed deteriorated average hearing levels (*p*=0.021) and high-tone hearing levels (*p*=0.008) after treatments.

**Conclusions**: Our study indicates that IMPT had less post-irradiation tinnitus and subtly better hearing performance during short-term follow-up. However, more patients and longer follow-up periods should be evaluated in the future.

### P 045 - Development of a simple model for skin toxicity prediction

#### Fernando Cerron^1^, Marta Montero^2^, de Pablo Ana^2^, Juan Maria Perez^1^, Juan Antonio Vera^1^, Juan Castro^1^, Raul Matute^2^, Raymond Miralbell^3^, Alejandro Mazal^4^

##### ^1^Centro de Protonterapia Quironsalud, Medical Physicist, Madrid, Spain ^2^Centro de Protonterapia Quironsalud, Radiation oncology, Madrid, Spain ^3^Instituto tecnologico Teknon, Chief of radiation oncology, Barcelona, Spain ^4^Centro de Protonterapia Quironsalud, Technical director of the center and chief of medical physicist department, Madrid, Spain

Skin toxicity is one of the known issues associated with head and neck tumors that are treated with radiation therapy. Due to the physics of the radiation-matter interaction, proton radiation therapy is known to deliver higher dose to the skin. This fact makes the quantification of the skin dose crucial to understand the relation of radiation received and skin toxicity. The prediction of the toxicities associated with a dose of radiation is fundamental to mitigate and treat the secondary effects of proton radiation. To try to anticipate potential toxicities, we have developed a model that correlate the volume of skin irradiated with a specified dose and skin toxicity grade. We analysed 22 H&N patients, and for each of them, we computed the volume of skin (assuming a thickness of 3 mm) irradiated with more than 50Gy and more than 60Gy (figure 1). We found a correlation of the volume of skin irradiated with more than 50Gy or 60Gy and grade 2-3 toxicity (see figure 2). On the other hand, more data must be collected for this model to be reliable, but it may be potentially useful for predicting skin toxicity.

### P 046 - Dosimetry comparison of incidental irradiation to the low axilla in whole breast irradiation using IMRT photons and IMPT protons therapy

#### Lu Cao^1^, Jia-Qi Huang^1^, Chao Li^1^, Mei Chen^1^, Yi-Bin Zhang^1^, Jia-Yi Chen^1^

##### ^1^Ruijin Hospital- Shanghai Jiaotong University School of Medicine, Department of Radiation Oncology, Shanghai, China

**Purpose**: It has been hypothesized that incidental dose to low axilla contributes to regional control in patients receiving whole breast irradiation (WBI). We aimed to compare axillary dose between intensity-modulated radiotherapy (IMRT) and intensity-modulated proton radiotherapy (IMPT).

**Methods**: Dosimetric comparisons between IMRT and IMPT were conducted in 10 patients treated with WBI without regional node irradiation(RNI). The prescribed dose was 40.05 Gy (RBE)/15Fx. Axillary lymph nodes levels I, II (ALN I, ALN II) and rotter’s lymph nodes (RN) were contoured and evaluated.

**Results**: The dose to the axilla was generally low for IMPT, with mean dose to ALN I, ALN II, and RN of 821.5, 223.0, and 2103.87 cGy (RBE), respectively. The mean V90% and V50% of ALN I was higher for IMRT (11.3 cm^3^, 22.2% of structure volume, p < 0.001; 36.8 cm^3^, 65.1%, p < 0.001) compared to IMPT (1.1 cm^3^, 1.9%; 7.7cm^3^, 14.7%). Similar finding was found in other low axilla. Mean dose to total ALN caudal to axillary vein was 857 cGy (RBE) in IMPT compared to 2262.1 cGy in IMRT (p < 0.001). Within all substructures of axilla, mean dose to RN was the highest, but it remains lower in IMPT than in IMRT [2103.87 cGy (RBE) versus 3618.7 cGy, p < 0.001].

**Conclusion**: WBI treated with IMPT leads to a significant reduction of axilla dose compared to IMRT. In clinical scenarios like Z0011 or likewise requiring incidental irradiation to the low axilla without additional RNI, contouring should be modified based on individual risk.

### P 047 - Impact of bladder volume on dosimetry of CTV and OARs in localized prostate cancer treated with proton therapy

#### Huan Li^1^, Dan-Ni Wang^1^, Lu Cao^1^, Wei-Xiang Qi^1^, Jia-Yi Chen^1^

##### ^1^Ruijin Hospital- Shanghai Jiaotong University School of Medicine, Department of Radiation Oncology, Shanghai, China

**Purpose**: To analyze the impact of bladder volume on dosimetry of clinical target volume (CTV) of prostate and organ at risk (OARs) in localized prostate cancer treated with intensity-modulated proton radiation therapy (IMPT).

**Materials and Methods**: A total of 25 patients and 30 sets of IMPT plans from the registration clinical trial for China’s first proton therapy system (SAPT-PS-01) were included in this analysis. Prescripted dose to CTV of prostate was 74-76 Gy (RBE) in 37-38 fractions. The bladder volume was measured in 30 sets of planning CT and 170 sets of weekly CT imagings during the treatment course in therapeutic position.

**Results**: The median V100%, and V95% of CTV were 99.49% and 100%, respectively. In the planning CT, the median bladder volume was 161.83 (IQR: 131-264) ml. Bladder volume was significantly negatively correlated with bladder mean dose and V40, V50, V60, V70 (b=−6.173, −0.018, −0.044, −0.063, −0.080, all P<0.01) and negatively correlated with mean dose and V50 of small intestine (b=−0.185, −0.062, both P<0.001). There was no significant impact of bladder volume on dosimetry of CTV and rectum in IMPT plans. During the treatment course, decrease in bladder volume by more than 20% was significantly associated with an increase in V40-70 of bladder, while weekly dynamic change of bladder volume had no significant impact on dosimetry of CTV.

**Conclusions**: The bladder volume was negatively correlated with the dosimetry of bladder and small intestine. It is recommended to keep the bladder volume 80% or above in planning CT during daily treatment.

### P 048 - Radiation-induced lymphopenia: Incidence and prevalence in proton therapy

#### Sarah Al-Hamami^1^, Jiří Kubeš^1^, Andrlik Michal^2^, Matěj Navrátil^2^, Vladimír Vondráček^2^

##### ^1^Proton Therapy Center, Radiation Oncology, Prague, Czech Republic ^2^Proton Therapy Center, Physics, Prague, Czech Republic

**Purpose**: Radiotherapy exhibits an interesting duality – it can stimulate an anti-tumour immune response, and it can also be immunosuppressive, primarily by inducing lymphopenia. A sufficient degree of immunosuppression may negate the immune response and ultimately lead to a worse clinical outcome. Our aim was to determine the incidence, prevalence, and grade of lymphopenia in various types of tumours treated with protons.

**Methods**: One hundred one (101) patients treated at a single center with diagnoses including head and neck, lymphoma, esophageal, lung, pancreatic and prostate tumours were selected. Blood counts were collected before, during (weekly), and after treatment, and analysed along with clinical status and dosimetric data.

**Results**: Across all diagnoses, 66% of patients developed some degree of lymphopenia. 10% developed grade 1 lymphopenia, 33% and 22% progressed to grade 2 and 3, and 2% reached grade 4. Total lymphocyte count (TLC) <1,000 cells/mm3 prevailed throughout the entire treatment course in 13% of patients, and for over half the course in 45%. Incidence was 0% in prostate without lymph node irradiation and 15% with lymph nodes reached G3 + 4. We noted a peak in TLC after the first fraction in 42% of patients. Incidence and prevalence generally correlated with an increase in PTV and total body integral dose.

**Conclusion**: This data, particularly in combination with a dosimetric data assessment, can be valuable in determining which factors have the greatest impact on TLC. Understanding the full complexity of radiation-induced lymphopenia could be the key to maximizing the synergistic effect of particle therapy and immunotherapy.

### P 049 - Outcomes for patients with spinal treatments extending over 5 or more vertebrae using proton beam radiotherapy

#### Teresa Dinizulu^1^, Shermaine Pan^1^, Gillian Whitfield^1^, Rovel Colaco^1^

##### ^1^The Christie NHS Foundation Trust, Clinical Oncology, Manchester, United Kingdom

**Aim**: We wanted to investigate any neurological dysfunction in patients treated with proton beam radiotherapy extending over 5 or more vertebrae

**Methods**: Looking at the case series of 6 patients treated for either ependymomas, pilocystic astroctyomas or spinal meningiomas we detailed the neurological function of these patients at baseline versus at most recent follow-up to determine if there was any loss in neurological status. We documented the dose delivered, mean CTV high, dose to spinal cord (max, 1cc & 5cc), number of vertebrae covered, and whether progression was seen at follow-up.

**Results**: This series of patients were treated at the Christie proton centre in Manchester from 2018 to 2021 for various pathologies including ependymomas, pilocystic astrocytomas, and spinal meningiomas. They ranged in age from 12 to 26 years. The longest treatment length extended over 19 vertebrae; from brainstem to T12, and the shortest was over 5 vertebrae; T2-T6. The mean number of vertebrae covered was 11. The max dose, 1cc & 5cc dose to the spinal cord remained well below dose constraints for that organ. Neurological function did not deteriorate for any of the patients treated. One patient had stable spinal lordosis 3 years post treatment.

**Discussion**: This small series analysis shows that with proton beam therapy it is possible to safely treat over multiple vertebral levels without leading to neurological dysfunction in those treated.

### P 050 - Comparison of craniospinal irradiation with proton beams by irradiation method and initial experience for pediatric patients

#### Nobuyoshi Fukumitsu^1^, Hikaru Kubota^1^, Masayuki Mima^1^, Yusuke Demizu^1^, Takeshi Suzuki^2^, Daiichiro Hasegawa^3^, Yoshiyuki Kosaka^3^, Atsufumi Kawamura^4^, Toshinori Soejima^1^

##### ^1^Kobe proton center, Radiation oncology, Kobe, Japan ^2^Kobe proton center, Anesthesiology, Kobe, Japan ^3^Kobe Children’s Hospital, Hematology and Oncology, Kobe, Japan ^4^Kobe Children’s Hospital, Neurosurgery, Kobe, Japan

**Purpose**: To compare craniospinal irradiation (CSI) of proton beam therapy (PBT) by irradiation methods and investigate the initial effect.

**Methods and Materials**: Twenty-four pediatric patients (age: 1–24 years) who received proton CSI were examined. Treatment method was passive scattered PBT (PSPT) in 8 patients and intensity modulated PBT (IMPT) in 16 patients. The whole vertebral body (WVB) technique was used for the 13 patients whose age was younger than 10 and vertebral body sparing (VBS) technique was used for the remaining 11 patients aged 10 and above. Follow up period was 17–44 (median 27) months. Doses of organs at risk, clinical target volume (CTV) and clinical data were examined.

**Results**: The maximum dose of the lens in the IMPT was lower than PSPT (p=0.008). The mean dose of thyroid gland, lungs, esophagus and kidneys were lower in the patients treated by VBS than MVB technique (p<0.001, all). The minimum CTV dose of IMPT was higher than PSPT (p=0.01). Inhomogeneity index of IMPT was lower than PSPT (p=0.004). Blood cell counts were mildly decreased overall and white blood cell and red blood cell counts were reduced in WVB technique (p=0.02 and 0.04). Seven of 8 patients who were treated with PSPT and all 16 patients treated with IMPT have been still alive as of Dec 2022.

**Conclusions**: IMPT can decrease the dose of lens than PSPT. VBS technique can decrease the doses of neck-chest-abdomen organs. The CTV coverage of IMPT are superior to PSPT. Hematological toxicity is mild.

### P 051 - Hypo-fractionated proton therapy as a treatment of high-risk head and neck non-melanoma skin cancers (N-MSC)

#### Dinu Stefan^1^, Laurine Bonnor^2^, Michel De Pontville^3^, Dorothée Lebhertz^2^, Marie Lecornu^1^, Cédric Loiseau^2^, Cyril Moignier^2^, Andreea Stefan^3^, Anthony Vela^2^, Jacques Balosso^1^

##### ^1^Centre François Baclesse, Radiotherapy, Caen, France ^2^Centre François Baclesse, Medical physics, Caen, France ^3^Centre François Baclesse, Dermatology, Caen, France

N-MSC is the most common tumors in the elderly, often located within the head-and-neck region. The prognosis of tumors larger than 40mm, with peri-neural and/or deep infiltration is less favorable. When surgery is not possible, radiotherapy, mostly based on photon/electron beams is an alternative. However these modalities are not adapted for superficial skin tumors given the “build-up” effect. Customized bolus are, thus, needed, but remain imperfect. Indeed, for head-and-neck N-MSC, bolus application can be poorly reproducible. Proton-therapy could be the technical of choice, because of its dose-deposition with no “build-up” effect and better tissue-sparing. From November 2021, 27 patients received hypo-fractionated proton-therapy for N-MSC with adjuvant (18patients) or exclusive intent (9cases). The reproducibility was good after robust evaluation and no bolus has been needed. An hypo-fractionated regimen of 3 or 6GyRBE per fraction, was used. The Relative Biological Efficiency of protons was 1,1. All patients presented a grade 1 radio dermatitis which was treated with topic applications. Late grade 3-4 late effects, were recorded in 2 cases (skin flap necrosis). An objective complete tumor response was observed in all cases treated with exclusive intention. No relapse has been diagnosed in these patients so far. Proton therapy is a promising treatment for high risk head-and-neck N-MSC. An hypo-fractionated schedule was used in order to reduce the treatment costs. More clinical data are needed in order to optimize the hypofractionated radiation regimens. This appears to be the first cohort of N-MSC patients treated by proton-therapy.

### P 052 - Dosimetric correlation of acute radiation dermatitis in breast cancer patients undergoing pencil beam proton therapy

#### Eng-Yen Huang^1,2,3^, Meng-Wei Ho^1^

##### ^1^Kaohsiung Chang Gung Memorial Hospital, Radiation Oncology, Kaohsiung, Taiwan Province of China ^2^College of Medicine- National Sun Yat-sen University, School of Medicine, Kaohsiung, Taiwan Province of China ^3^Chang Gung University, School of Traditional Chinese Medicine, Taoyuan, Taiwan Province of China

**Purpose**: To evaluate the correlation of skin dosimetry and acute radiation dermatitis in patients undergoing proton beam therapy for breast cancer.

**Materials and Methods**: From October 2018 to December 2022, 29 patients with breast cancer were reviewed. The dose of radiotherapy was 40.5 Gy (RBE)/15 fractions (n=26), 43.5 Gy (RBE)/15 fractions (n=2), or 50 Gy (RBE)/25 fractions (n=1) for whole breast or chest wall without or with regional lymph nodes. Pencil beam proton therapy (Sumitomo Heavy Industries) was delivered. Skin doses (from surface to 3mm depth) were evaluation as maximum dose, 1cc, and 10cc dose. Electron beam boost 8.7-10 Gy/3-4 fractions was given in 18 patients. Acute radiation dermatitis was evaluated according to common toxicity criteria (CTC). Receiver operating characteristic (ROC) curve and area under curve (AUC) was used to choose optimal cutoff dose for Grade 2 or greater dermatitis.

**Results**: All patients developed radiation dermatitis. Grade 2 dermatitis was noted in 4 (36.4%) and 10 (55.6%) patients without and with electron boost (*p*=0.316), respectively. No Grade 3 or 4 dermatitis was noted. ROC curve revealed that CTV volume (AUC=0.911, *p*=0.004), D10cc (AUC=0.811, *p*=0.029), D1cc (AUC=0.844, *p*=0.016) and Dmax (AUC=0.789, *p*=0.042) were significant factors of Grade 2 dermatitis. Logistic regression revealed CTV volume>300 mL (*p*=0.007) and D1cc > 42 Gy (RBE) (*p*=0.030) were independent factors of Grade 2 dermatitis.

**Conclusion**: Proton beam therapy may achieve acceptable incidence of Grade 2 dermatitis. D1cc < 42 Gy can be a constraint to reduce incidence of Grade 2 dermatitis.

### P 053 - The role of LET and RBE in the occurrence of sacral insufficiency fractures in CIRT for sacral chordoma

#### Giuseppe Magro^1^, Laura Giberti^2^, Silvia Molinelli^1^, Angelica Ghirelli^1^, Nadia Facchinetti^1^, Agnieszka Chalaszczyk^1^, Salvatore Gallo^2^, Mario Ciocca^1^, Maria Rosaria Fiore^1^, Ester Orlandi^1^

##### ^1^CNAO Foundation - National Center for Oncological Hadrontherapy, Clinical Department, Pavia, Italy ^2^University of Milan, Physics Department, Milan, Italy

**Purpose**: To assess the correlation of radiation-induced sacral insufficiency fractures (SIFs) associated with carbon-ion radiation therapy (CIRT) with RBE models (RBE) and dose-averaged LET (LET*_D_*) maps.

**Material and Methods**: We retrospectively analyzed 78 patients, consecutively treated between 2013 and 2021, with radical CIRT. The prescribed RBE-weighted dose (*D*_RBE_) ranged from 70.4 to 76.8 Gy(RBE), in 16 fractions, according to the local-effect-model (LEM). The median follow-up was 38 months (range 2-111). For patients reporting SIF at follow-up, the fracture was contoured on the corresponding MR. For the sacral bone, the correlation between SIF occurrence and DRBE, calculated with both LEM and microdosimetric-kinetic-model (MKM), and LETD was assessed with the Mann-Whitney test.

**Results**: SIF was diagnosed in 44% of patients after a median follow-up of 18.5 months. The fracture occurred in areas irradiated with intermediate doses and >90% patients experienced fractures in the sacral wings (Figure-1). For MKM, the dose received by >20-25% of the sacrum volume (D20-25%) and the fraction of volume receiving >20-25 Gy(RBE) (V20-25Gy(RBE)) were significantly higher in the fractured cohort and influenced the occurrence of toxicity over time (Figure-2). For LETD, statistically significant differences were found in LET95% and V20-25keV/μm. No significant relationships among SIF occurrence, LEM parameters and patient age were found. Females had higher risk of fracture.

**Conclusion**: SIF occurred mainly when larger portions of sacrum were irradiated. Highest doses also contributed to the appearance of the fracture (MKM-D20-25%). Further analyses should be performed to develop a patient-specific risk assessment and minimize severe clinical complications.

### P 054 - Development of occupational status and financial toxicity in patients with meningioma before and after proton therapy

#### Birgit Flechl^1^, Milana Achtaewa^1^, Lisa Konrath^1^, Eugen B. Hug^1^, Piero Fossati^1,2^, Maciej Pelak^1^, Petra Georg^3^, Carola Lütgendorf-Caucig^1^

##### ^1^EBG MedAustron GmbH, Medical Department, Wiener Neustadt, Austria ^2^Karl Landsteiner University of Health Sciences, Medical Department, Krems, Austria ^3^University Hospital Krems, Radiation therapy, Krems, Austria

**Objectives**: Patients (pts) with meningioma are commonly treated with proton therapy (PT) with high control rates. However, potential loss of employment or reduced working hours may lead to financial worries. We investigated subjective disease burden in pts with meningioma with regards to income, financial difficulties and inability to resume work before and after PT.

**Methods**: Data from prospectively assessed questionnaires (EORTC QLQ-C30) including subjective financial difficulties scaling from 1-4 (1=“not at all” to 4=”very much”) was collected. Observed time points were prior to start of PT (t1) and incrementally thereafter (up to 48ms;t2-t8). Net household income was ranked into 5 groups from <1300€->5000€and employment status was collected.

**Results**: One hundred seventy-one (171) pts (39m/131f) aged 20-88 years (mean 55.1) with meningioma were included with a mean observation time of 29.6ms. Thirty-seven (37) pts (35.9%) reported inability to work before PT. Over observation, it decreased to 33.8% at 12ms, 27.3% at 24ms, 15.4% at 36ms, 13.6% at 48ms. Thirty-four (34, or 33%) were employed prior to their diagnosis (14part-time, 18full-time). While rates of full-time employment initially decreased, it steadily increased from 1yr FU (t3-t8: 28.7%-59.1%). Before PT, 69 pts (40%) reported financial difficulties. Although rates decreased initially they increased significantly at 6ms (36.9%; *p*<*0.001*) and consistently decreased at all subsequent time points up to 20.6% at 48ms reflecting the lower burden of financial toxicity over time.

**Conclusion**: At a mean observation time of >2yrs, we prospectively analyzed the potential impact of proton therapy in patients treated for meningioma. Patients reported an overall increase in employment rate and significant improvement in their financial situation over time.

### P 055 - Less plan adaptations with 3 beam proton radiotherapy in esophageal cancer patients

#### Petra Klinker^1^, Erik Korevaar^1^, Pietro Pisciotta^1^, Margriet Dieters^1^, Charlotte Ijsbrandy^1^, Jannet Beukema^1^, Johannes langendijk^1^, Christina Muijs^1^

##### ^1^University Medical Centre Groningen, Radiation Oncology, Groningen, Netherlands

**Purpose**: In 2020 we started with model base selection (MBS) of esophageal cancer (EC) patients for Intensity Modulated Proton Therapy (IMPT). The aim of this study was to evaluate the impact on plan adaptations of: time (representing a potential learning curve), and other potential relevant factors, such as breathing motion and beam set up.

**Material and Methods**: EC patients were selected for IMPT using the model-based approach. Additionally, target and diaphragm motion had to be <15 mm and <25 mm, respectively. Initially, robustly optimized IMPT plans were created using a 2 beam approach. After 6 months, a 3 beam set up was gradually introduced as new standard to improve robustness. Weekly repeat CTs were performed to evaluate target coverage. If target coverage was regarded insufficient by the radiation oncologist, plan adaptations were obtained.

**Results**: Since April 2020, 135 out of 161 patients (84%) were treated with IMPT (see Table 1). Plan adaptations were required in 39 (28.9%) patients. Using a 2 beam IMPT plan, plan adaptations were performed in 50% of the patients, while in patients with a 3 beam strategy this was 23.9% (p<0.05). In multivariable logistic regression analysis, plan adaptation was significantly associated with beam set up (Table 2). No significant association was found with time after introduction or breathing amplitude of target or diaphragm.

**Conclusion**: The change in beam set up from 2 to 3 beams in IMPT for EC resulted in less patients with plan adaptations. Plan adaptation was not significantly associated with time or breathing motion.

### P 056 - Proton therapy in locally aggressive pituitary macroadenomas: First clinical results

#### Birgit Flechl^1^, Eugen Hug^1^, Astrid Fraller^2^, Antonio Carlino^1^, Maciej Pelak^1^, Christoph Hajdusich^1^, Petra Georg^3^, Piero Fossati^1,4^, Carola Lütgendorf-Caucig^1^

##### ^1^EBG MedAustron GmbH, Medical Department, Wiener Neustadt, Austria ^2^Landesklinikum Wiener Neustadt, Radiation therapy, Wiener Neustadt, Austria ^3^University Hospital Krems, Radiation therapy, Krems, Austria ^4^Karl Landsteiner University of Health Sciences, Medical Department, Krems, Austria

Pituitary macroadenomas are generally benign diseases. A subgroup presents a therapeutic challenge due to skull base invasion, close proximity to the optic structures, or recurrent growth. We report on patients treated with fractionated proton therapy (PT) at MedAustron therapy center. 08/2017-12/2022, 21 patients were treated. Median age was 52a (30-77a). 67% (n=14) had 1 surgery before PT, 9.5% (n=2) 4 surgeries and 24% (n=5) no surgeries. 76% (n=16) were benign tumors (69%, n=11 null cell adenomas; 31%, n=5 hormone-active), 25% (n=5) higher grade histology (Ki67 ≥10%). All tumors were initially in contact with optical system. 62% (n=13) had pre-existing optical deficits, 71% (n=15) partial endocrine dysfunctions. Ophthalmological and endocrinological examinations were carried out before PT and in the follow-up (F/U). Tumor response was assessed by MRI, toxicity by CTCAE V4.0. CTV1 included tumor and pre-op extent (anatomically adapted), with doses >54Gy, a CTV2 (=GTV) was also defined, PTV margin was 3mm. Median dose was 54GyRBE (54-66Gy) in 27-33 fractions; 5 patients with higher grade histology and/or extensive skull base infiltration received 54-66GyRBE (median dose 60GyRBE). Optical structures were immediately adjacent to or part of the CTV. Median D1% on optical structures was 54GyRBE (49-60GyRBE). With a median F/U of 28months (max. 62months), local control was 100%. No acute or early-late toxicity ≥G3 was observed. Pre-existing visual deficits did not change significantly and no new ones appeared. Initial data show that in patients with locally aggressive pituitary macroadenomas, PT is a good therapeutic option in terms of local control with preservation of visual functions.

### P 057 - Clinical outcomes and patient-reported functional status after proton therapy for intracranial meningioma in children: A single-center retrospective cohort experience

#### Marta García Marqueta^1^, Reinhardt Krcek^2^, Míriam Vázquez Varela^1^, Ulrike Kliebsch^1^, Dominic Leiser^1^, Nicola Bizzocchi^1^, Nadia Sauder^1^, Alessia Pica^1^, Damien C. Weber^1^

##### ^1^Paul Scherrer Institut, Center for Proton Therapy, Villigen, Switzerland ^2^Inselspital, Universitätsklinik für Radio-Onkologie, Bern, Switzerland

**Purpose**: To report the experience in the treatment of pediatric intracranial meningioma with pencil beam scanning proton therapy (PBS-PT) and to assess their functionality after treatment.

**Patient and Methods**: Out of a total cohort of 207 intracranial meningioma patients treated with PBS-PT, 10 (5%) were children treated between 1999 and 2022. Median age was 13.9 years (range, 3.2-17.2). Seven (70%) children were treated as primary treatment (postoperative PT, n=5; exclusive PT, n=2) and all presented with skull base lesions. Acute and late toxicities were registered according to Common Terminology Criteria for Adverse Events. Educational, functional, and social aspects were assessed through our in-house developed follow-up questionnaires. Median follow-up time was 71.1 months (range, 1.6-249.7).

**Results**: Five (50%) children had local progression 32.4 months (range, 17.7 -55.4) after PBS-PT, of which four were considered in-field. One patient died of T-cell lymphoma. Four-year local tumor control and overall survival were 51.43% and 100.0%, respectively. No patient developed grade ≥ 3 late toxicities. One child developed cataract that required surgery. During the first year after PT, one child required educational support, one needed to attend a special school, one had social problems and three required assistance for daily basic activities (DBA). Three years after PT, one child required educational support and another one assistance for DBA.

**Conclusions**: The outcome of children with intracranial meningioma after PBS-PT is in line with other series reporting the results of radiotherapy delivered to these patients. This therapy provides favourable functional status profiles with no high-grade adverse radiation-induced events.

### P 058 - Short-term efficacy and toxicity of a registration clinical trial for China’s first proton therapy system: A preliminary report

#### Wei-Xiang Qi^1^, Lu Cao^1^, Cheng Xu^1^, Huan Li^1^, Yunsheng Gao^1^, Zhiling Chen^2^, Zhentang Zhao^2^, Haitao Chen^3^, Guang Ning^4^, Jia-Yi Chen^1^

##### ^1^Ruijin Hospital- Shanghai Jiao Tong University School of Medicine, Radiation oncology, Shanghai, China ^2^Shanghai Advanced Research Institute- Chinese Academy of Sciences, na, Shanghai, China ^3^Ruijin Hospital-Shanghai Jiao Tong University School of Medicine, Thoracic surgery, Shanghai, China ^4^Shanghai Institute of Endocrine and Metabolic Diseases- Ruijin Hospital- Shanghai Jiao Tong University School of Medicine, Endocrine and Metabolic Diseases, Shanghai, China

**Objective**: SAPT-PS-01 was the first proton therapy system based on synchrotron produced by Shanghai APACTRON Particle Equipment Co., Ltd. This registration clinical trial was conducted as part of the registration procedure from National Medical Products Administration to verify its efficacy and safety in clinical implementation.

**Materials and Methods**: This was a single-center and single-arm objective performance criteria trial, in which all participants were allocated to proton irradiation. The primary endpoints were 3-months local tumor control probability (TCP) and acute treatment-related adverse events (TRAEs).

**Results**: Between Nov 2021 and Jun 2022, a total of 47 participants were treated with IMPT. Among them, 39 patients treated with a fixed beam treatment system and 8 patients with a 180° rotating beam treatment system. Of these patients, 12 participants presented with head and neck cancer, 4 cases with thoracic/abdominal tumors, 4 cases with spinal tumors and 27 cases with prostate cancer. After completion of 3-months follow-up, the local TCP was 100%. Of the 22 non-prostate tumor cases, 4 cases (18.2%) archived partial response, and 18 cases with stable disease. For the 27 prostate carcinomas, biological-recurrence free survival was 100% (figure 1). Proton irradiation was well tolerated. 32 cases (68.1%) developed grade 1 TRAEs and 4 cases (8.5%) with grade 2 TRAEs. No grade 3 or higher TRAEs occurred.

**Conclusion**: The preliminary report of this registration clinical trial confirms that the proton therapy system SAPT-PS-01 is safe and effective. Long-term follow-up is still needed to determine the late toxicity and long-term TCP.

### P 059 - Evaluation of robustness in proton therapy of high-risk prostate patients included in national clinical trial

#### Sofie Tilbæk^1^, Stine Elleberg Petersen^1^, Anne Vestergaard^1^, Lilliana Stolarczyk^1^, Heidi S. Rønde^1^, Tanja Johansen^1,2^, Lise Bentzen^3^, Jimmi Søndergaard^4^, Morten Høyer^1^, Ludvig Muren^1^

##### ^1^Aarhus University Hospital, Danish Centre for Particle Therapy, Aarhus N, Denmark ^2^Rigshospitalet, Department of Oncology, Copenhagen, Denmark ^3^University of Southern Denmark, Department of Oncology- Vejle Hospital, Vejle, Denmark ^4^Aalborg University Hospital, Department of Oncology, Aalborg, Denmark

**Introduction**: Inter-fractional anatomical changes may degrade treatment quality in proton therapy of high-risk prostate cancer. Pre-treatment robust evaluation (PRE) of treatment plans takes into account isocenter shifts and range uncertainties. The aim of this study was to perform an offline during-treatment robust evaluation (DRE) using weekly control-CT scans (cCTs), and compare this to the PRE.

**Materials and Methods**: Treatment plans and 7-8 cCTs from each of the first three pilot patients of the randomized trial PROstate PROTON Trial 1 (NCT05350475) were evaluated. Treatment plans were optimized following protocol guidelines with 78 Gy to the primary target (CTVp; prostate and involved seminal vesicles) and 56 Gy to the elective target (CTVe; pelvic lymph nodes) in 39 daily fractions. CTVp was expanded into an internal target volume (iCTV) to account for deformations and displacements. Treatment plans were recalculated on the cCTs and dose/volume measures corresponding to clinical constraints were checked to see if the DRE was within the predicted range from the PRE.

**Results**: Of the 22 cCTs, 20 showed CTVp measures from the DRE inside the range of the PRE (Fig. 1); this was also the case for most of the CTVe measures. The DRE constraint measures for the rectum, bladder and bowel were outside the PRE range in 0, 4 and 14 cCTs, respectively.

**Conclusion**: All treatment plans and most of the cCTs recalculations showed acceptable target coverage and acceptable doses to organs at risk. Treatment plan robustness will be assessed prospectively for the remaining pilot patients of the trial.

### P 060 - Early clinical and patient-reported outcomes following definitive proton therapy for patients with sacral chordoma

#### Michelle Li^1,2^, Maeve Keys^1^, Daniel Saunders^1^, James Wylie^1^, Corinne Faivre-Finn^3^, James Price^1^, Ed Smith^1^

##### ^1^The Christie NHS Foundation Trust, Proton Beam Therapy Centre, Manchester, United Kingdom ^2^Peter MacCallum Cancer Centre, Radiation Therapy, Melbourne, Australia ^3^The Christie NHS Foundation Trust and The University of Manchester, Department of Radiotherapy Related Research, Manchester, United Kingdom

**Purpose**: There is limited data on the toxicities and outcomes of using definitive intensity modulated proton therapy (IMPT) for sacral chordomas. This study evaluated the acute toxicities, patient reported outcomes and early clinical outcomes of patients with sacral chordoma treated with definitive IMPT in a single institution.

**Methods**: Records of patients between January 2019 and October 2020 were retrospectively reviewed. Patient treatment details, toxicity data and disease status at each follow-up were recorded. The toxicities were scored prospectively using CTCAEv5.0. Electronic patient reported outcome measures (ePROMs) including EuroQol five-dimensional, five-level questionnaire (EQ-5D-5L) were completed.

**Results**: Ten patients were identified. The median age was 61 years (41-81). All patients received 75.6CGE. Median follow-up time was 14 months (range,0-25). Nine patients were alive at time of analysis. Four patients had acute grade 3 skin toxicity that subsequently improved. Other common acute toxicities were grade 1/2 pain, constipation and fatigue. No acute grade 4/5 toxicities were reported. After PBT, one patient developed a rectal fistula and two patients had pelvic insufficiency fractures. Two patients had a radiological partial response, six patients had stable disease, and one patient recurred outside the radiation field. Four patients completed ePROMs at baseline. 3/4 patients had improved self-rated health status at completion of treatment. At 3 weeks follow-up, the EQ5D-5L profile improved in two patients, unchanged for one patient and worsened in another patient.

**Conclusion**: Definitive PBT for sacral chordoma is well tolerated and provides good early local control, however a larger cohort with longer follow-up is required.

### P 061 - Proton therapy for bone and soft tissue tumors: An audit of initial 116 consecutive patients treated over 3 years

#### Ramakrishna Kamath^1^, Srinivas Chilukuri^1^, Sham Sundar^1^, Nagarjuna Burela^1^, Vishnu Ramanujan^2^, Radhakrishnan Satheesan^3^, Ramya Uppuluri^4^, Revathi Raj^4^, Sujith Mullapally^5^, Rakesh Jalali^1^

##### ^1^Apollo proton cancer centre, Radiation Oncology, Chennai, India ^2^Apollo proton cancer centre, Orthopedic Oncology, Chennai, India ^3^Apollo proton cancer centre, Pediatric Surgery, Chennai, India ^4^Apollo proton cancer centre, Pediatric Hemato-Oncology, Chennai, India ^5^Apollo proton cancer centre, Medical Oncology, Chennai, India

**Purpose**: Proton beam therapy (PBT) is an emerging modality used in the management of bone and soft tissue(BST) tumors. We report an audit of BST tumors treated with image-guided intensity-modulated PBT at our center in the first 3 years of its operation.

**Method**: 116 consecutive patients treated with PBT at our center for BST tumors were retrospectively analyzed. Patient, tumor, treatment-related characteristics and early outcomes were analyzed. All treatments were based on a multidisciplinary tumor board.

**Results**: Patient characteristics are mentioned in table 1. At presentation, 24% had recurrent disease, 15% received prior radiation and 34% underwent more than 1 surgery. 48% received definitive treatment, while 27% received adjuvant and 1 patient received pre-operative treatment. Median high-risk clinical tumor volume (CTV) was 100.57cc (1.43–1995.94) and median low-risk CTV was 418.58cc (13.26–4841.58). The median PBT dose was 70 CGE (41.4–72.6) over 32 fractions (20-35). The median GTV D98 of spinal tumors was 64.61 CGE (61.76 – 69.41 CGE). 12% of patients had metal implants and received a Proton-Tomotherapy combination. 31% received systemic therapy of which 9.1% of patients received concurrent treatments. At a median follow-up of 12 months(5–38months), 94% had locally controlled disease, 83% are progression-free, 11% had systemic progression and 6% had local progression. 11 patients(9.5%) had acute grade 3 toxicities of which 7 were dermatological.

**Conclusion**: Based on our early experience, with carefully selected patients of BST tumors, PBT seems to be safe and effective. Patients are being accrued into a prospective database to analyze long-term outcomes.

### P 062 - Proton beam therapy for paediatric renal tumours using a targeted flank delineation approach

#### Mohamed Khalid Abutaleb^1^, Matthew Lowe^2^, Frances Charlwood^2^, Daniel Saunders^1^, Ed Smith^1^, Shermaine Pan^1^

##### ^1^The Christie NHS Foundation trust, Clinical Oncology department- The Proton Beam Therapy Centre, Manchester, United Kingdom ^2^The Christie NHS Foundation trust, Christie Medical Physics and Engineering- Proton Beam Therapy Centre, Manchester, United Kingdom

**Introduction/Background**: Advanced radiotherapy techniques such as arc therapy and proton beam therapy (PBT) to the flank for primary renal tumours have improved conformality and reduced dose to organs at risk (OARs). We report a single centre experience of flank treatment with pencil beam scanning PBT.

**Patients and Methods**: All paediatric patients (<16 years), who received PBT to the flank between December 2018 and November 2022 were retrospectively identified (n=5). Patients were planned using Eclipse v16.1.

**Results**: Five patients with median age of 47 months (16-59), all with left-sided tumours were included. Patient characteristics and dosimetric information are in Table 1. All patients had a targeted flank delineation approach according to SIOP-2001 with a radiation dose of 10.8–25.2Gy(RBE). Patients were treated supine with 3 to 6 posterolateral fields using multifield optimisation. With a median follow-up of 11 months (2-15), local control is 100%. Doses to contralateral kidney were extremely low in all cases. Mean spleen dose was limited to <10Gy in one patient without target coverage compromise after local implementation of national guidance on splenic dose constraint. Pancreas mean dose was close to 10Gy for 2/5 patients despite no predefined dose constraint before planning. All patients experienced predominantly Grade 1 acute toxicities of Fatigue, abdominal pain and diarrhoea. The only grade 2 toxicity was constipation in 1 patient. No patients had >Grade 2 acute toxicities.

**Conclusion**: We present the first UK experience of flank PBT for primary renal tumours. Treatment outcome thus far shows good local control with potential to minimise doses to OARs.

### P 063 - Proton beam therapy for high-risk neuroblastoma patients: A brief review of clinical effectiveness

#### Sung-Hsin Kuo^1^, Chun-Wei Wang^1^, Christine NS Chong^2^, Hsiu-Hao Chang^3^, Kun-Huei Yeh^4^, Ann-Lii Cheng^5^, James Chih-Hsin Yang^5^

##### ^1^National Taiwan University Hospital and and National Taiwan University Cancer Center, Department of Oncology, Taipei, Taiwan Province of China ^2^YongLin Healthcare Foundation, YongLin Healthcare Foundation, Taipei, Taiwan Province of China ^3^National Taiwan University Hospital, Departments of Pediatrics, Taipei, Taiwan Province of China ^4^National Taiwan University Hospital, Department of Oncology, Taipei, Taiwan Province of China ^5^National Taiwan University Cancer Center, Department of Medical Oncology, Taipei, Taiwan Province of China

**Background**: We previously showed that high-risk neuroblastoma patients who received radiotherapy (RT) to the primary site using helical tomotherapy (median dose of 21.6 Gy) had a favorable local control and survival benefit. Our institute will be proud to offer neuroblastoma patients access to advanced, multidisciplinary cancer care (including proton beam therapy (PBT), ProBeam® proton therapy system) in the last quarter of this year. The aim of the present systematic review is to evaluate the efficacy and safety of PBT, as part of a modern multimodality paradigm, for high-risk neuroblastoma patients.

**Methods**: We provide a brief overview of selected literature on the relative biological effectiveness of dose, double scattered proton or pencil beam scanning, local control, disease-free survival (DFS), overall survival (OS), and toxicity of PBT for the primary site or metastatic site of patients with neuroblastoma who receive systemic chemotherapy followed by tumor excision, and high-dose chemotherapy and autologous stem-cell transplantation.

**Results**: For high-risk neuroblastoma patients, the most dose for the primary site or metastatic site was 21.6 Gy, whereas the most dose for gross residual lesion was 36 Gy. Overall, PBT provides a better local control rate compared with photon beam therapy, ranging from 97% to 100%, a 3-year DFS ranging from 77.0% to 81.8%, and a 3-year OS ranging from 89.0% to 90.9%, and it results in rare grade 2 toxicities of the late gastrointestinal tract and hematological toxicities (including renal toxicity).

**Conclusions**: PBT provides excellent local control and clinical outcome with rare acute and late adverse effects for high-risk neuroblastoma patients.

### P 064 - Outcomes of patients treated in the UK Proton Overseas Programme with proton beam therapy

#### Simona Gaito^1^, Eun Ji Hwang^1^, Anna France^2^, Adrian Crellin^3^, Marianne Aznar^4^, Gillian Whitfield^1^, Beate Timmermann^5^, Daniel J. Indelicato^6^, Adam Holtzman^6^, Ed Smith^1^

##### ^1^The Christie NHS FT, Proton Beam Therapy Centre, Manchester, United Kingdom ^2^The Christie NHS FT, Proton Clinical Outcomes Unit, Manchester, United Kingdom ^3^NHS England, Lead of NHSe Proton Beam Therapy service, Manchester, United Kingdom ^4^University of Manchester, Division of Clinical Cancer Science- School of Medical Sciences, Manchester, United Kingdom ^5^University Hospital Essen, Department of Particle Therapy, Essen, Germany ^6^University of Florida, Department of Radiation Oncology, Jacksonville, USA

**Aim**: In 2008, the UK National Health Service (NHS) started the Proton Overseas Programme (POP), to provide access for Proton Beam Therapy (PBT) abroad for selected tumour diagnoses, whilst two national centres were being planned. Clinical outcomes for patients treated within the POP are reported.

**Materials and Methods**: Since the start of the POP, an agreement between NHSEngland and UK referring centres ensured PBT outcomes data collection into a prospective national patient database, including overall survival (OS), local tumour control (LC) and late toxicity data (LT). Clinical and treatment-related data were extracted from this database. Grade (G) ≥3 LT are reported following CTCAE (Common Terminology Criteria for Adverse Events) v 4.0, occurring later than 90 days since completion of PBT. The patient cohort was divided by diagnoses into Central Nervous System (CNS) and Non-CNS subgroups.

**Results**: Between 2008 and September 2020, 1325 patients were treated within the POP. OS data were available for 1310 patients and Local Control data for 1184 patients. Toxicity analysis was carried out on 1138 patients, with patients excluded due to short follow-up (<90 days) and/or inadequate toxicity data available. Overall survival and Local control data are presented in Table 1. G3 toxicities were the most common and shown in Figure 1. In the CNS subgroup, 8 G4 and 4 G5 toxicities were also reported.

**Conclusions**: The results of this study indicate safety of PBT for both CNS and non-CNS malignancies, which reflects sound patient selection and treatment quality. The rate of late effects compares favourably with published cohorts.

### P 065 - ^11^C-Methionine and ^18^F-3,4-Dihydroxyphenylalanine-PET-CT for target boost delineation in brain tumors

#### Jacobo Palma Delgado^1^, Jose Javier Aristu Mendioroz^1^, Adriana Ayestaran Aldaz^1^, Francisco Javier Serrano Andreu^1^, Rosa María Meiriño Seoane^1^, Felix Mauricio Cambeiro Vázquez^1^, Pedro Aguilar Aguilar Redondo^2^, Alberto Viñals Muñoz^2^, José Pablo Cabello García^2^, Felipe Ángel Calvo Manuel^1^

##### ^1^Clínica Universidad de Navarra, Radiation Oncology, Madrid, Spain ^2^Clínica Universidad de Navarra, Physics, Madrid, Spain

**Objectives**: ^11^C-Methionine Positron Emission Tomography (MET-PET) and ^18^F-3,4-Dihydroxyphenylalanine Positron Emission Tomography (DOPA-PET) may contribute to a more precisely delineation of the target volume, in particular a metabolic-guided definition a intratumoral boost. The use of PET planning images can contribute to improve outcomes of brain tumors.

**Methods**: Between 10/2020 and 11/2022 28 patients diagnosed of primary or recurrent CNS tumors, with a median age of 48 years (range 25-74) were treated with Proton Therapy (PT). CT, MR and MET-PET/DOPA-PET images were obtained for radiation planning. The GTV-boost was generated using the MET-PET/DOPA-PET metabolically active regions (Figure 1) and the CTV boost were designed from the GTV-boost plus a 5mm of margin. The CTV no Boost were delineated using MR T2 FLAIR/T1Gad sequences.

**Results**: Eleven (39.3%) were glioblastoma, 10 (35.7%) AA, 7 (25%) others. Ten patients were reirradiated. All patients showed areas of hypermetabolism within the neoplasia range and showed a differential distribution of the trace to guide boost delineation. The median prescribed dose was 60Gy RBE (35-60) and the median number of fractions was 25 (10-28). Seven patients relapsed, with a median progression free survival of 6 months (1-24). Twenty-four patients are alive, with a median over survival since end of the treatment of 5 months (0-24).

**Conclusion**: The use of PET in CNS tumors allow boosting selected hypermetabolic tissue, in effort to improve outcomes. It is necessary a longer follow up and a large number of patients to assure the impact in overall survival.

### P 066 - Implication of posterior neck lymph node irradiation inclusion for comprehensive breast treatments with intensity modulated proton therapy

#### Marcio Fagundes^1^, Jen Yu^1^, Dan Chen^1^, Joseph Panoff^1^, Maria Amelia Rodrigues^1^, Zachary Fellows^1^, Lorrie Legrand^1^, Andrew Wroe^1^, Alonso Gutierrez^1^

##### ^1^Miami Cancer Institute, Department of Radiation Oncology, Miami, USA

**Purpose**: The posterolateral supraclavicular region known as posterior neck (PN) has been shown to be at risk for nodal metastases in node positive breast cancer patients. We evaluated the dosimetric impact on organs at-risk (OAR) with and without inclusion of the (PN) in intensity modulated proton therapy (IMPT).

**Methods**: Twelve patients received left-sided post-mastectomy comprehensive IMPT between 4/2020 and 5/2022 to 50.4Gy in 28 fractions targeting the chest-wall, axilla, supraclavicular and internal mammary with inclusion of PN nodes. Two en-face beams were employed with robustness evaluation to ensure targets received at least 95% of the prescription dose. Retrospectively, plans were re-optimized excluding PN with the same planning configurations and goals (Figure 1). Dosimetric values for OARs were compared with and without PN irradiation. Wilcoxon signed-rank test was conducted to evaluate the statistical significance of the differences.

**Results**: Table 1 summarizes the comparison of OAR dose values for patients treated with IMPT. Mean heart, lung V5, V20 and brachial plexus D1cc doses (W/PN vs WO/PN) were: 0.87 vs 0.87Gy, 45.3 vs 46.7%, 18.1 vs 18.4% and 51.6 vs 51.8Gy, respectively (p=NS). Overall, mean humeral head dose was higher with PN, 9.9Gy vs 5.2Gy (p<0.001), however both values are at or lower than historical photon controls. For three patients treated without humeral head avoidance, mean humeral head dose was 17Gy vs 7.5Gy for those with avoidance, demonstrating that increased beam modulation is able to reduce dose.

**Conclusion**: Inclusion of PN did not increase dose to heart, lung or brachial plexus.

### P 067 - Linphopenia pattern during photon vs. proton craniospinal irradiation for meduloblastoma

#### Javier Serrano^1^, Javier Aristu^1^, Jacobo Palma^1^, Elena Panizo^1^, Alvaro Lassaleta^1^, Borja Aguilar^1^, Rosa Meiriño^1^, Mauricio Cambeiro^1^, Carmen Gonzalez^2^, Felipe A. Calvo^1^

##### ^1^Clinica Universidad de Navarra. Campus Madrid., Radiation Oncology, Madrid, Spain ^2^Gregorio Marañón University Hospital, Radiation Oncology, Madrid, Spain

**Objective**: Analyse variations in lymphocyte counts in medulloblastoma pediatric patients (p) using proton or photon craniospinal irradiation (CSI).

**Methods**: Two consecutive pediatric cohorts from two institutions were prospectively registered and retrospectively analysed. All p were treated with conformal-3D photon radiotherapy (3DCRT) or pencil-beam scanning protontherapy (PBSPT) under identical protocol. Patients received between 23.4-39.6 Gy CSI (median=36Gy) and 54-59.4 Gy boost (median=55.8Gy) using daily 1.8 Gy fractions. Complete blood test including lymphocyte values were obtained pre, during and after CSI completion. Platelets were transfused if <30.000 and GCSF if <1.000 neutrophil count.

**Results**: Thirty-four children were included for analysis, 17 treated with 3DCRT and 17 with PBSPT. Relevant clinical characteristics were similarly distributed (age, gender, risk-group, metastatic-stage, chemotherapy, postsurgical residue, duration and CSI-boost dose). No differences were observed in G-CSF requirements (median 8 vs 12 doses). A comparative case-study of lymphocyte lethal doses (V3Gy) that received the following structures using protons vs photons showed: thoracic aorta (36.11 vs 44.55 cc), heart (20.72 vs 293.55 cc), liver (28.75 vs 612.66 cc) and total integral dose (5902.95 vs. 12488.71 cc), respectively. Complete cohort evaluation is under analysis. Minimum leucocytes and neutrophils count between photon and proton groups were 1.083 vs 1.383 per mm3 and 636 vs 756 per mm3, respectively. Statistically significant difference was observed in mean (327 vs 461 per mm3; p=0.005) and minimum lymphocytes count (105 vs 170 per mm3; p=0.013) in favour of proton group.

**Conclusion**: Proton PBSPT CSI significantly protects from lymphopenia in medulloblastoma pediatric patients compared to photon 3DCRT.

### P 068 - Definitive and postoperative IMPT for oral cancers: The APCC experience

#### Sapna Nangia^1^, Manthala Padannayil Noufal^1^, Utpal Gaikwad^2^, Nagarjuna Burela^1^, Anupamma Reddy^1^, Anusha t^1^, Sham Sundar^1^, Srinivas Chilukuri^1^, Dayananda Shamurailatpam Sharma^1^, Rakesh Jalali^1^

##### ^1^Apollo Proton Cancer Centre, Proton Therapy/Radiation Oncology Centre, Chennai, India ^2^Krupamayi Hospital, Radiation Oncology, Auranagabad, India

Reduction in toxicity and improvement in treatment outcomes of oral cancer (OC) remain unmet needs. Proton therapy (PT) allows significant reduction of the dose received by organs-at-risk and the possibility of improved outcomes. We report toxicity and clinical outcomes of definitive and postoperative PT for OCs. Twenty-seven consecutive OC patients underwent image-guided-intensity-modulated-proton-therapy (IMPT) for inoperable cancers in 7, and as postoperative adjuvant in 20 patients, respectively, from May 2019 to Dec 2022. Their characteristics were as follows: gender: M: F-20:7, age: 23-89 years, median 52 years, stage: T3-T4-14, N2-N3-16, disease subsite: anterior tongue-13, bucco-alveolar-complex-11, palate- 2, and floor-of-the mouth-1 patient, respectively. Eight patients received 70 Gy to HRCTV, 12, 64-66Gy and 7, 60 Gy, in standard fractionation, using dose-painting. Ten of 13 patients treated with concurrent systemic therapy received at least 5 doses. Grade 3 acute mucositis was observed in 55%, limited to the HRCTV and grade 3 skin reactions in 26%. Notably, weight loss was <5% in 63% and <10% in 96%. Four of 7 patients aged ≥65yrs and 3 of 20 patients <65 yrs old required feeding-tube insertion. Treatment interruption was ≤ 2 days in 7 (26%) of the 10 (37%) patients who required a gap. At a median follow-up of 19 months, the 1-yr and 2-yr LRFS and DFS are 91.4% and 80%, respectively. The OS at one year is 88%, and at 2 yrs, 78%. We conclude that in a cohort comprising OC patients treated for inoperable disease and following surgery, PT demonstrates excellent tolerance and outcomes.

### P 069 - Efficacy and safety of protontherapy in high-grade meningiomas

#### Katarzyna Holub^1,2^, Thibault Passeri^3^, Loic Feuvret^4^, Sylvie Helfre^1^, Farid Goudjil^1^, Isabelle Pasquie^1^, Remi Dendale^1^, Sébastien Froelich^3^, Valentin Calugaru^1^, Hamid Mammar^1^

##### ^1^Institut Curie- Paris Saclay University, Department of Radiation Oncology, Paris, France ^2^Univeristy of Barcelona, Oncology, Barcelona, Spain ^3^Hôpital Lariboisière, Department of Neurosurgery, Paris, France ^4^Hôpital La Pitié Salpêtrière, Department of Radiation Oncology, Paris, France

**Purpose**: To evaluate the efficacy and the safety of the high-dose conformational Protontherapy (PT) in high-grade meningiomas (HGM).

**Method**: One hundred twenty three consecutive patients with HGM WHO grade 2 (G2, n=101) and grade 3 (G3, n=22) treated with Protontherapy +/−photon beam component at the Institute Curie – Orsay Protontherapy Centre between Nov 1996-Sep 2022. The total median dose was 68.0 Gy RBE in 38 fractions with Proton component alone (n=91; G2=76, G3=15) or Protons + photon component (n=32; G2=25, G3=7). The median age at PT was 60.6 years old (range 18-79.6), 74 females and 49 males. Localization was supratentorial in 80 patients (65.0%), skull base in 33 (26.8%), and infratentorial in 10 (8.1%). Gross tumor resection was reported in 29 cases (23.6%), subtotal in 91 (22.8%), while 3 patients were not treated with surgery; 41 patients underwent multiples (2-4) surgeries. Local control rate (LC), time to local progression (TLP), overall survival (OS), meningioma-related survival (MRS) and 2-, 5- and 9-year survival rates were evaluated using Kaplan-Meier method. Post-PT clinical toxicity was graded according to the Common Terminology Criteria for Adverse Events (CTCAE v 5.0).

**Results**: During follow-up after PT (median 47.6 months), local progression was reported in 38 patients (LC rate 69.1%, in G2=74.3%, in G3=45.4%) and 35 patients died (including 21 meningioma-related deaths). Median TLP was 32.7 months (IC 95% 1.9-164.5) and OS was 163 months (IC 95% 111.4-214.6). Late toxicities were reported in 77 patients (≤ Grade2).

**Conclusions**: PT alone or PT-photons were effective and well-tolerated treatment in HGM.

### P 070 - Proton therapy for skull-base chordomas and chondrosarcomas: Clinical experience from the Maria Sklodowska-Curie National Research Institute of Oncology in Krakow

#### Tomasz Skóra^1^, Dominika Wojton-Dziewońska^1^, Kamil Kisielewicz^2^, Elżbieta Pluta^1^, Anna Patla^1^, Eleonora Góra^2^, Konrad Urbanek^1^, Renata Kopeć^3^, Garbacz Magdalena^3^, Jadwiga Nowak-Sadzikowska^1^

##### ^1^Maria Sklodowska-Curie National Research Institute of Oncology, Department of Radiotherapy, Kraków, Poland ^2^Maria Sklodowska-Curie National Research Institute of Oncology, Department of Medical Physics, Kraków, Poland ^3^Institute of Nuclear Physics Polish Academy of Sciences, Bronowice Cyclotron Center, Kraków, Poland

**Purpose**: The objective of this study is to present our results in patients with skull-base chordomas and chondrosarcomas treated at our Institute with proton beam therapy.

**Methods and Materials**: Between November 2016 and December 2020, 75 patients (median age, 51 years; range, 22-77 years) with skull-base tumors were treated with proton pencil beam scanning. Seventy three patients underwent upfront surgery. Forty-nine (65.3%) patients were diagnosed with chordoma. The median prescribed dose for chordoma and chondrosarcoma was 74GyRBE and 70 GyRBE, respectively. Survival rates were calculated. Treatment tolerance was assessed using CTCAE (version 5.0) grading system.

**Results**: The median follow-up was 51.7 months (range, 2.8-74.1 months). Nine (12%) patients died. The deaths were tumor-related in 5 (6.7%) patients. At the time of analysis 11 (14.7%) patients experienced treatment failure (local – 7 patients, distant – 4 patients). The local failure-free survival rates at 2 and 3 years were 94.5% and 89.7%, respectively. The corresponding overall survival rates were 94.6% and 88.7% for both time cutoffs. The late high-grade (G3) treatment toxicity included 5 patients with hearing impaired and 1 patient with brain necrosis.

**Conclusion**: Proton therapy with pencil beam scanning seems to be effective treatment for skull-base chordomas and chondrosarcomas. Despite high dose administration, the late toxicity is acceptable.

### P 071 - Simultaneous integrated boost in dominant intraprostatic lesion in moderate- and ultra-hypofractionated PET PSMA-MRI guided proton therapy prostate cancer

#### Mauricio Cambeiro^1^, Javier Aristu^1^, Javier Serrano^1^, Jacobo Palma^1^, Rosa Meiriño^1^, Bernardino Miñana^2^, Borja Aguilar^1^, Felipe Calvo^1^

##### ^1^Clinica Universidad de Navarra, Radiation Oncology, Madrid, Spain ^2^Clinica Universidad de Navarra, Urology, Madrid, Spain

**Purpose**: To evaluate the feasibility of dominant intraprostatic lesions (DILs) SIB boost in the context of moderate hypofractionated Intensity modulated proton therapy (IMPT) prostate cancer (PC).

**Methods and Materials**: Patients with organ confined PC underwent fiducial Carbone Marker and SpaceOAR prior CT scan and mpMRI planning radiotherapy in RayStation Planning System (RaySearch). CTV prostate and DILs were defined using T2-weighted dynamic contrast-enhanced and diffusion-weighted. Prescription dose on CTV Prostate was 60 Gy in 20 (RBE factor 1.1) Eq2Gy α/β 2 (75.0 Gy). Prescription dose (PD) in CTV DILs was 110% of the CTV (Prostate) prescription. Strict Rectal and Bladder constraints were predefined D1 rectal (0.01cc) <105% DP (60 Gy); V40.5<50%; V48.7<35%; V52.7 < 30%. D1 Bladder (0.01cc) <105% DP (60 Gy) V56.8<5%; V48.7<25%. Treatment was delivered in Hitachi Synchrotron Accelerator with MFO Pencil Beam Scanning technique. IGRT megavoltage cone beam was performed before each treatment.

**Results**: Ten patients were treated. All patients received more than 95% of PD on CTV prostate and CTV DIL; median D95% CTV Prostate was 58 Gy and median D95% CTV DIL was 62.7 Gy. All patients met predefined rectal and bladder constraints. No acute G2 or higher rectal or urinary toxicity were registered.

**Conclusions**: Radical Hypofractionated IMPT PC with Boosting DILs and the fulfillment of strict rectal and bladder constraints is feasible and well tolerated with IMPT MFO Pencil Beam Scanning technique. Follow-up to assess the clinical impact of dosimetric data is needed.

### P 072 - Outcomes for patients treated with post-mastectomy breast reconstruction and adjuvant proton therapy

#### Julie Bradley^1^, Eric Brooks^2^, Raymond Mailhot Vega^2^, Oluwadamilola Oladeru^3^, John Murray^4^, Christopher Morris^3^, Emma VIviers^2^, Teena Burchianti^2^, Nancy Mendenhall^2^

##### ^1^University of Florida Health Proton Therapy Institute, Radiation Oncology, Jacksonville, USA ^2^University of Florida, Radiation Oncology, Jacksonville- FL, USA ^3^University of Florida, Radiation Oncology, Gainesville- FL, USA ^4^University of Florida, Surgery, Jacksonville- FL, USA

**Purpose**: We report clinical outcomes for patients treated with proton therapy following mastectomy with breast reconstruction.

**Methods**: From a prospective database, we identified patients with breast cancer treated with mastectomy with immediate or delayed breast reconstruction and adjuvant proton therapy between 2012 and 2021. Clinical and dosimetric data were extracted from the electronic medical record. The cumulative incidence method assessed reconstruction failure rate.

**Results**: Sixty (60) patients were identified (with 61 treated reconstructed breasts or chest walls). Median age at the time of radiation was 48 years (range, 25-79). Immediate expander reconstruction was performed in 47, immediate implant in 6, immediate autologous with expander in 2 and delayed autologous in 6. Median total radiation dose was 60GyRBE (range, 50-73), delivered with pencil beam scanning in 40 and double scatter in 21 reconstructed breasts/chest walls. Median follow-up was 3.0 years (range, 0.8-10.0). Three-year locoregional control was 100% (1 local failure at 3.7 years). The 3-year cumulative incidence of patients with post-radiation reconstruction failure was 13% (95%CI: 6%-23%). Median time from completion of radiation to reconstruction failure was 0.7 years (range, 0.2-1.5). Reconstruction failure occurred due to infection (n=5), implant exposure (n=2), and flap necrosis (n=1). 41 (67%) of reconstructed breasts underwent planned post-radiation reconstruction surgery, most commonly expander to implant exchange. Nineteen (31%) of reconstructed breasts also underwent unplanned reconstructive surgeries, primarily to improve symmetry.

**Conclusion**: Post-mastectomy proton therapy for patients with breast reconstruction results in excellent locoregional control, with rates of unplanned reconstructive surgeries and reconstruction failures similar to photon series.

### P 073 - Proton Beam Therapy (PBT) in adult patients with spinal/sacral chordoma: A single institution experience

#### Martha Wilson^1^, Mark Reed^1^, Ed Smith^1^, Anna France^2^, James Wylie^1^, Daniel Saunders^1^, William Croxford^1^, Maeve Keys^1^

##### ^1^The Christie NHS Foundation Trust, Proton Beam Therapy, Manchester, United Kingdom ^2^The Christie NHS Foundation Trust, Proton Clinical Outcomes Unit, Manchester, United Kingdom

**Introduction**: Since December 2018, patients with spinal/sacral chordoma have been treated at the Christie Hospital PBT Centre. We describe prospectively gathered demographics, outcomes and toxicity data.

**Method**: Follow up data was identified for 59 patients treated from December 2018 – August 2022. Data was collected from Christie clinical records, and ARIA treatment planning system at baseline, 6 weeks, 6 months, and annually post treatment. CTCAE V4 and RTOG skin toxicity grading were used.

**Results**: Median age at treatment was 58 years (28-82years). Gender split was 32% Female 68% Male. Histology included 1 de-differentiated chordoma. Anatomical sites treated: C-spine 8 (14%), T-spine 6 (10%), L-spine 11 (19%) and sacrococcygeal 34 (58%). Median dose prescribed was 75.6 Gy/CGE (range 50.4-75.6Gy/CGE). 24 (41%) patients received definitive PBT, 35 (59%) patients received post-operative PBT (2 pre- and post-op). Median follow-up was 12 months (0-38 months). 11 (19%) patients have relapsed: 2 locally, 6 distant and 3 both local and distant. 3 (5%) patients had died at analysis. Of those who relapsed locally 4 were treated definitively with large tumours. 2 patients had grade 3 toxicities recorded at baseline. There were 6 late grade 3 toxicities reported. No grade 4 or 5 toxicities reported.

**Conclusion**: PBT has good local control and acceptable toxicity in this cohort. Longer follow up with a larger number of pts. is needed to establish OS, PFS and whether there is wider prevalence of higher-grade long-term toxicity

### P 074 - Proton craniospinal irradiation for patients with solid tumor leptomeningeal metastasis: Suggested planning approaches for prior irradiation patients

#### Zachary Fellows^1^, Andrew Wroe^1,2^, Rupesh Kotecha^1,2^, Matthew Hall^1,2^, Minesh Mehta^1,2^, Alonso Gutierrez^1,2^

##### ^1^Miami Cancer Institute, Radiation Oncology, Miami, USA ^2^Herbert Wertheim College of Medicine- Florida International University, Radiation Oncology, Miami, USA

**Objective**: Proton craniospinal irradiation (CSI) for patients with leptomeningeal disease (LMD) often require consideration of prior radiotherapy (prior-RT). We evaluated two treatment planning approaches for patients with overlapping prior spinal irradiation in various clinical scenarios.

**Methodology**: For standard LMD CSI cases, prescription dose administered is 30Gy in 10fx to the brain (two posterior oblique fields) and spinal axis (gradient matched PA fields). For spine prior-RT patients, two planning techniques were investigated that reduced dose in the overlap region, namely dose-painting and sequential cone-down. The goal of both techniques is to deliver 20Gy EQD2 while limiting the cumulative spine dose to 60Gy EQD2 using an alpha/beta of 3.

**Results**: For dose-painting approach, prior-RT is considered upfront in the optimization to decrease dose to these regions. Although more complex, this results in a single plan treated in 10fx. If areas of prior-RT extend superior to C4 or inferior to L4, the dose-painting option is not feasible given field size limitations and need to maintain a 10cm gradient. Moreover, when multiple sites of irradiation exist along the spine, it is difficult to employ the dose-painting approach. A sequential cone-down technique irradiates the whole craniospinal axis to 18Gy in 6fx, followed by boost to 30Gy total dose to the target not previously irradiated. This sequential approach requires additional computational resources but enables a treatment approach for patients not being able to be treated via dose-painting.

**Conclusion**: This study describes two planning techniques that can be utilized for proton CSI patients with LMD and prior-RT.

### P 075 - Reirradiation of recurrent head and neck tumors with protontherapy: A single institution experience

#### Marta Montero Feijoo^1^, Morena Sallabanda Hajro^1^, Fernando Cerron Campoo^2^, Juan María Pérez Moreno^2^, Juan Castro Novais^2^, Juan Antonio Vera Sánchez^2^, Ana de Pablo Gonzalez^1^, Raúl Matute Martin^1^, Alejandro Mazal^2^, Raymond Miralbell Izard^1^

##### ^1^Centro de Protonterapia Quironsalud, Radiation Oncology, Madrid, Spain ^2^Centro de Protonterapia Quironsalud, Medical Physicist, Madrid, Spain

The aim of this study is to describe a single-institution experience regarding local control and tolerance of protontherapy reirradiation in a cohort of patients with recurrent head and neck tumors following different treatment schedules depending on location, histology and prior OARs doses. Between January/2021-December/2022, 9 patients diagnosed with recurrent head and neck tumors previously irradiated with radical dose usually combined with chemotherapy, were re-irradiated with protontherapy in our institution. The estimated mean time between irradiations was 8.5 years (range 10 months-30 years). Among these patients, 4 were treated in a normofractionation scheme (30-35 fractions of 2GyRBE/fraction), 4 with moderate hypofractionation (14-15 fractions of 4GyRBE/fraction) and one of them under extreme hypofractionation (5 fractions of 8GyRBE/fraction). Treatment was delivered with a Proteus®ONE IBA using Pencil Beam Scanning. After a median follow-up of 9.45 months (range 21-3 months) 7 patients (66.7%) experienced complete response, one (11.1%) showed local control with distant progression 6 months after the end of treatment, one (11.1%) died due to tumor progression 7 months after the end of the treatment and the other patient (11.1%) died due to post-treatment infectious complications. Tolerance during treatment was acceptable, with grade II-III mucositis, grade II dysphagia and grade II epithelitis. One patient suffered an esophageal perforation solved with conservative treatment. In conclusion, despite the risk of severe toxicity described for head and neck reirradiation, protontherapy is an effective strategy with a low rate of life-threatening complications. Hypofractionated schemes seems to be cost-effective with equivalent results in terms of local control compared to normofractionated treatment.

### P 076 - Evidence-based review on the effectiveness of proton re-irradiation for pelvic malignancies

#### Tien Yee Amy Chang^1^, Bin Yang^2^, Anand Aman^3^, Ming Chun Darren Poon^1^, Jonathan Ashman^3^

##### ^1^Hong Kong Sanatorium & Hospital, Comprehensive Oncology Centre, Hong kong, Hong Kong ^2^Hong Kong Sanatorium & Hospital, Medical Physics Department, Hong Kong, Hong Kong ^3^Mayo Clinic Arizona, Department of Radiation Oncology, Phoenix, USA

**Background**: Pelvic malignancies after prior local irradiation can develop recurrence, and pelvic re-irradiation caused major toxicities. The role of proton re-irradiation and potential benefit is reviewed in this study.

**Methods**: Medline and PubMed are used for literature search on patients with gynecological, gastrointestinal, and prostate cancers who received proton re-irradiation. Studies since 2011 and sample size of at least ten patients were included. Clinical outcome including acute and late toxicities, 1-year local control, overall and progression-free survival are reviewed.

**Results**: Three case series were included, showing gynecological and rectal cancers were the most common primary pelvic malignancies receiving proton re-irradiation. Acute and late grade 3 and above toxicity were low (3.8-10.7% and 0-13.3% respectively). Local control was acceptable (67 – 90%) and 1-year overall survival was satisfactory (62-81.8%). There is dosimetric advantages of sparing to bone marrow, femur, bladder and rectum comparing proton versus photon in one study.

**Conclusions**: Proton re-irradiation has been shown to be well tolerated with satisfactory survival. Further investigation on the use of multi-modality treatment settings should be explored to improve clinical outcome.

### P 077 - Comparative evaluation of IMPT and VMAT for brachial plexus sparing in repeat regional nodal irradiation for recurrent non-metastatic breast cancer

#### J. Isabelle Choi^1,2^, Beryl McCormick^2^, Jana Fox^3^, Peter Park^1^, Mark Millar^1^, Katherine Walker^2^, Chih Chun Tung^1^, Peter Florio^2^, Chin-Cheng Chen^1^, Oren Cahlon^4^

##### ^1^New York Proton Center, Radiation Oncology, New York, USA ^2^Memorial Sloan Kettering Cancer Center, Department of Radiation Oncology, New York, USA ^3^Montefiore Medical Cancer, Department of Radiation Oncology, New York, USA ^4^NYU Langone, Department of Radiation Oncology, New York, USA

**Purpose**: Repeat regional nodal irradiation (RNI) for recurrent non-metastatic breast cancer presents a treatment challenge due to cumulative brachial plexus (BP) dose tolerance. We hypothesize that pencil beam scanning proton therapy (PBS-PT) will better optimize tumor coverage and BP sparing relative to photon volumetric-modulated arc therapy (VMAT).

**Methods and Materials**: In an IRB-approved comparative planning study, consecutive patients with non-metastatic ipsilateral recurrent breast cancer treated with prior photon RT and then received PBS-PT reirradiation (reRT) with at least partial BP overlap were identified. VMAT and IMPT plans were created using standard TV including breast/chest wall, axilla I-III, IMN, and supraclavicular region (SCV). Prescription dose was 50.4 Gy in 28 fractions, with a standard maximum BP dose constraint of <25 Gy.

**Results**: Coverage of the three levels of the axilla and supraclavicular region differed significantly at dose levels of V55%, V65%, V75%, V85%, and V95% between the VMAT and IMPT plans, with increased dose coverage in the IMPT plans. (Table 1) Only V55% of axilla level I coverage did not reach a p-value of <0.05 (p=0.056) in favor of IMPT. Coverage of regions through which the BP does not typically traverse, including the chest wall/breast and IMN chain, were not significantly different between VMAT and IMPT.

**Conclusion**: In this cohort, PBS-PT improved BP sparing and TV coverage versus VMAT. Clinical correlation with DVH parameters will be of value to better define cumulative BP dose constraints in the setting of reRT.

### P 078 - Dosimetric planning evaluation of single stereotactic body proton therapy versus stereotactic body radiation for bony metastases

#### Kitwadee Saksornchai^1^, Napapat Amornwichet^2^, Kanjana Shotelersuk^2^

##### ^1^Chulalongkorn University- King Chulalongkorn Memorial hospital The Thai Red Cross society, Faculty of Medicine- Division of Radiation Oncology- Department of Radiology, Bangkok, Thailand ^2^Chulalongkorn University- King Chulalongkorn Memorial hospital The Thai Red Cross society, Faculty of Medicine- Division of Radiation Oncology- Department of Radiology, Bangkok, Thailand

**Purpose**: There is limited data on stereotactic body proton therapy (SBPT) in bone metastatic disease. We would like to compare the dosimetric study of a single fraction comparing SBPT and stereotactic body radiotherapy (SBRT).

**Material and Methods**: Eight (8) spine and 3 non-spine lesions who had previously completed photon therapy at our institution were replanned with SBRT and SBPT. The prescription dose was a single dose of 18 Gy. The spinal and non-spine treatment target volumes were contoured according to RTOG 0631 and BR001 protocol, respectively. The SBPT plans were created with two posterior oblique fields (gantry angle ranging from 130° to 150° and 210° to 230°). For robustness considerations, proton planning was performed with a 3-mm setup uncertainty and a 3.5% range uncertainty. The treatment plan is acceptable as long as 95% of the target volume receives the prescribed dose.

**Results**: The median treated volumes were 45.5 cc (45.5–61.1). In terms of target coverage, there was no significant difference between SPBT and SBRT. The Dmax of the CTV was higher in the SBRT group (20.1(SBRT) vs 22.4 Gy (RBE) (SBPT), p-value 0.045). For organs at risk dose, there is no statistically significant difference between SBPT and SBRT planning in parameters of spinal cord, partial cord, bladder, and rectum. SBPT plans were significantly better than SBRT plans in the heart, lungs, kidneys and bowel dosimetric values.

**Conclusion**: SBPT improves some of the organs at risk dosimetric metrics while remaining comparable in target coverage. Further clinical studies are needed to prove the feasibility of using single dose of SBPT.

### P 079 - Proton stereotactic radiosurgery (PSRS) using pencil beam scanning (PBS) combined with collimating aperture

#### Chen-Yu CHOU^1^, Chien-Yi Yeh^1^, Shen-Hao Lee^1^, Hsiao-Chieh Huang^1^

##### ^1^Linkou Chang Gung Memorial Hospital, Department of Radiation Oncology, Taoyuan City, Taiwan Province of China

**Purpose**: Proton stereotactic radiosurgery (PSRS) is a proton treatment approach that allows providing a shaper penumbra dose fall off and better surrounding tissue sparing. The PBS delivery technique combined with a collimated aperture can achieve this goal. This study evaluates the dose result of PSRS and traditional IMPT.

**Materials and Methods**: We have designed and set up a homemade aperture system for proton treatment, especially with small targets. There are two choroid melanoma and three arteriovenous malformations (AVM) patients that were enrolled in this study. For each patient, two treatment plans were executed, with and without using the collimated aperture. All plans were optimized on the CTV with the same beam arrangement and the same robust optimization (2 mm setup errors, 3.5% range uncertainties). The heterogeneity index (HI) and conformal index (CI) were used to evaluate plan quality and dose distribution outcomes. R50 index (50%-dose volume divided by the target volume) was also used for analyzing the low-dose volume changes.

**Results**: There are no significant differences in plan results between PSRS and traditional IMPT approaches. Both treatment approaches provide similar plan quality. However, dose conformality and low-dose volume significantly improve if implementing collimated apertures.

**Conclusion**: This study result shows the aperture system can provide benefits to the proton cases with small targets.

### P 080 - Late toxicity profile in ultrahypofractionated proton radiation therapy in prostate cancer: A single-center experience of the first two years

#### Nikita Kataev^1^, Nikolay Vorobyov^1^, Alexei Mikhaylov^1^, Natalia Berezina^2^, Mikhail Cherkashin^2^, Kirill Suprun^2^

##### ^1^Medical Institute n.a. Berezin Sergey, Radiation Oncology, Saint Petersburg, Russian Federation ^2^Medical Institute n.a. Berezin Sergey, Administrative Department, Saint Petersburg, Russian Federation

**Purpose**: To analyze late toxicity profile in patients with prostate cancer treated with intensity-modulated proton therapy(IMPT) with an ultrahypofractionated schedule in the first two years in our center.

**Materials and Methods**: Between 02.2019 and 02.2021, 73 patients were treated using IMPT with a median total dose of 36.83Gy SD=0.41Gy in 5 fractions (36-37.5Gy). The mean age at the time of treatment was 63.9 SD=8.4(33-82) years, and the median prostate-specific antigen (PSA) value was 9.05 μg/L(2.83-32.00μg/L). A total of 25(34.2%) patients had low-risk, 37 patients(50.7%) intermediate-risk, and 11(15.1%) patients high-risk cancer. WHO/ISUP grade group was 1 in 48(66.7%) patients, 2 in 21(28.7%) patients, 3 in 3(4.1%) and 4 in 1(0.5%) patient. Of 73 patients, 17 were lost from follow-up and thus were excluded from the analysis. Data for 56 patients was collected by patient reports and analyzed with a median follow-up time of 22(2-46) months. In addition, 9(12.3%) patients underwent neoadjuvant androgen deprivation therapy(ADT) and no patients had adjuvant ADT. Prostate cancer radiotherapy late toxicity questionnaire results were obtained by phone.

**Results**: The median treatment time was 5.3 days(5-8days). Late toxicity (CTCAEv.5.0) was as follows: gastrointestinal(GI): grade 1, 10 patients(17.9%), grade 2, 3 patients(5.3%), and no patients reported grade 3 or higher GI toxicity; genitourinary(GU): grade 1, 8 patients(14.3%), grade 2, 6 patients(10.7%), and no patients reported grade 3 or higher GU toxicity. De novo erectile disfunction(ED) rate was reported in 8 patients (14.3%).

**Conclusion**: In our study, the use of ultrahypofractionated IMPT for prostate cancer did not result in significant GU, GI toxicity, and ED.

### P 081 - Feasibility study of using intensity modulated proton therapy for head and neck stereotactic radiation therapy

#### Fahed Alsanea^1^, Jack Phan^2^, Falk Poenisch^1^, Thomas J. Whitaker^1^, Tylor Williamson^3^, Rachel Hunter^3^, Xiaodong Zhang^1^, He Wang^1^

##### ^1^UT MD Anderson Cancer Center, Radiation Physics, Houston, USA ^2^UT MD Anderson Cancer Center, Radiation Oncology, Houston, USA ^3^UT MD Anderson Cancer Center, Radiation Oncology Physics Support, Houston, USA

**Purpose**: Stereotactic body radiation therapy (SBRT) is a treatment technique utilized for patients with head and neck tumors due to proximity of target volumes to numerous critical organs. We investigate the feasibility of proton therapy SBRT.

**Methods**: We selected 18 patients with head and neck tumors previously treated with photon SBRT using volumetric-modulated arc radiation therapy (VMAT) at MD Anderson Cancer Center. Intensity-modulated proton therapy (IMPT) plans were created in Raystation using Monte Carlo dose calculation algorithm. The IMPT plans used 3-4 fields with robust optimization (±2.5% range, and 2mm setup uncertainties) to cover the same CTVs as in VMAT plans. Target coverage and doses to critical organs of the IMPT plans were compared to the photon VMAT plans.

**Results**: The 18 patients (3 skull base, 7 neck, 3 larynx and 5 oropharynx patients) had primary tumors with mean volume 18.4 cm^3^ (range: 0.82 – 76.2 cm^3^). The SBRT dose was 27 – 45 Gy in 3-5 fractions. IMPT plans achieved comparable primary CTV coverage (97.6 ± 1.6% vs. VMAT 96.3 ± 1.6%) and CTV uniformity (D1/D99 = 1.08 ± 0.11 vs. VMAT 1.09 ± 0.08). Lower IMPT doses were observed to critical organs that were >5mm away from targets. Comparing to VMAT plans, the volume in patient body covered by 20Gy dropped up to 37% in IMPT plans.

**Conclusion**: There is a dosimetric advantage of proton therapy SBRT over photon SBRT. Further studies are needed to utilize this clinical benefit including management of anatomic change uncertainties through adaptive planning.

### P 082 - Extreme hypofractionation in skull base chordomas and chondrosarcomas: First experience with proton therapy

#### Morena Sallabanda^1^, Juan Antonio Vera^2^, Juan María Pérez^2^, Marta Montero^1^, Ana De Pablo^1^, María Isabel Garrido^1^, Raúl Matute^1^, Raymond Miralbell^1^, Fernando Cerrón^2^, Juan Castro^2^, Alejandro Mazal^2^

##### ^1^Centro de Protonterapia Quironsalud, Radiation Oncology, Madrid, Spain ^2^Centro de Protonterapia Quironsalud, Medical Physics, Madrid, Spain

**Purpose**: To describe acute toxicity and dosimetric parameters of the first 10 patients with chordomas or condrosarcomas of the skull base treated with five-fraction protontherapy.

**Materials and Methods**: Ten patients with chordoma or chondrosarcoma of the skull base were included in a prospective protocol of hypofractionated protontherapy between June and December 2022. The inclusion criteria were: Age > 18 yo., Karnofsky Performance Status (KPS) ≥ 70%, absence of metastases, clinical target volume (CTV) up to 50 cc and compliance with dose restriction to organs at risk. Treatment was delivered with a Proteus®ONE IBA cyclotron using Pencil Beam Scanning. IMPT plans were obtained employing 4 to 6 beams. In 8 patients, apertures were used to reduce lateral penumbra.

**Results**: Ten patients (6 males and 4 females) with age 29 - 79 yo., were included. Five patients with classic chordomas and five patients with grade I-II condrosarcomas were treated with a total dose of 37.5 Gy and 35 Gy, respectively, in 5 daily fractions. CTV range was 10-50 cc. Median CTV coverage was V95%=D95%. Tolerance during treatment was excellent with grade I asthenia and headache in 7 patients and grade I nausea in 3 patients. Median clinical follow-up was 4 months (1-7 months). Five patients presented alopecia, but the overall acute tolerance was remarkable.

**Conclusions**: Five-fraction protontherapy for the treatment of skull base chordomas and condrosarcomas is dosimetrically feasible and well tolerated with very low acute toxicity for selected patients. Longer follow-up is necessary to further validate treatment tolerance and local control.

### P 083 - Dose-escalated proton therapy with concurrent and adjuvant immunotherapy for chordoma: Early results and toxicities from a single institution case series

#### Keitrina Mair^1^, Yazmin Odia^2^, Minesh Mehta^1^, Muni Rubens^3^, Katherine Von Werne^1^, Carlos Rios^1^, Ranjini Tolakanahalli^1^, Andrew Wroe^1^, Alonso N. Gutiérrez^1^, Matthew Hall^1^

##### ^1^Miami Cancer Institute- Baptist Health South Florida, Radiation Oncology, Miami, USA ^2^Miami Cancer Institute- Baptist Health South Florida, NeuroOncology, Miami, USA ^3^Miami Cancer Institute- Baptist Health South Florida, Biostatistics, Miami, USA

**Purpose**: To report on outcomes and toxicities following dose-escalated proton therapy (PT) with concurrent and adjuvant immunotherapy in chordoma patients at a single institution.

**Methods**: Data on chordoma patients treated with immunotherapy and either PT or combined proton/intensity-modulated radiotherapy (PT/IMRT) from 2017-2022 were retrospectively reviewed. Radiotherapy and immunotherapy toxicities were scored using CTCAE v4.0.

**Results**: Seven chordoma patients (2 skull base, 3 mobile spine, 2 sacrum) received immunotherapy and PT and met the eligibility criteria. Median age was 51 years (range: 35-79). Three (43%) received PT alone while four received PT/IMRT due to metal hardware placed during surgery. Median dose was 68.4 GyRBE (range: 66-73.8). Six patients (86%) received pembrolizumab (200 mg IV q3weeks) and one (14%) nivolumab. Six (86%) received concurrent and adjuvant immunotherapy with radiotherapy, while one only received adjuvant immunotherapy. With a median follow-up of 18 months, six patients remain alive and five are disease-free. One patient developed a local-only failure and another developed distant-only progression. Concurrent immunotherapy/radiotherapy were well-tolerated. No patients experienced grade 3+ toxicity during or after radiotherapy; grade 2 toxicities were limited to mucositis/esophagitis in two (29%) and skin desquamation in three patients (43%). During adjuvant immunotherapy, two patients discontinued treatment after 5-6 cycles due to disease progression; two others had delays due to Grade 2 thyroiditis and colitis, respectively, that resolved without corticosteroids. To date, four patients have completed >6 months of immunotherapy.

**Conclusions**: Concurrent and adjuvant immunotherapy for chordoma were well-tolerated in this small series with no Grade 3 toxicities to date.

### P 084 - New trends in enhancing the biological efficacy and safety of proton therapy: Georgian experience

#### Archil Chirakadze^1^, Nodar Mitagvaria^2^, Giorgi Chubinidze^1^, Nugzar Dvali^1^, Teimuraz Chichua^1^, Zakaria Buachidze^3^, Akaki Gigineishvili^4^, David Jishiashvi^5^, Mariam Abuladze^6^, Irakli Nadiradze^7^

##### ^1^Caucasus International University, Scientific Centre for the Synthesis and Research of Chemotherapeutic Drugs, Tbilisi, Georgia ^2^Caucasus InternationalUniversity, Scientific Centre for the Synthesis and Research of Chemotherapeutic Drugs, Tbilisi, Georgia ^3^Georgian Technical University, Archil Eliashvili Institute of Control Systems, Tbilisi, Georgia ^4^Georgian Technical University, Department of Engineering Physics, Tbilisi, Georgia ^5^Georgian Technical University, V. Chavhhanidze Institute of Cynernetics, Tbilisi, Georgia ^6^Kutaisi International University, Hadron Therapy Center, Kutaisi, Georgia ^7^Georgian Technical Uiiversity, Department of Physical Engineering, Tbilisi, Georgia

The concept of highly localized combined multicomponent cancer therapy in its advanced form has been formed very recently. Its sufficiently substantiated principles were first set forth in papers of the Japanese and Georgian scientists in 2017 and 2018. Our research summarizes the most significant results and findings of a five-year multidisciplinary study aimed at improving the biological efficacy and safety of one of the most modern methods of cancer treatment, namely - proton therapy. To overcome the most principle limitations of the efficacy and safety of proton therapy (quite high doses of radiation; the spread of proton radiation into healthy tissues; leakage of radiation; secondary neutron radiation; Prompt gamma-quants; Inaccuracy in target positioning or displacement., novel solutions in combining chemotherapy, Curie temperature controlled magnetic hyperthermia, photodynamic therapy and treatment with reactive oxygen species (ROS); development and effective use of temperature, irradiation and pH controlled anticancer nanomaterials, nano-based fluids and targeted delivery systems; effective use of additional proton-boron and neutron-boron nuclear reactions with aim to increase the share of double strand breaks (DSBs) in DNA and reduce the penetration of primary and secondary radiation in healthy tissues; effective use of various DNA reparative synthesis inhibitors, ionophores, DMSO and various organic solvents; Sparing methods of animal testing according to the 4R principles, etc., have been analysed, developed and applied. Promising result and findings have been achieved and reported.

### P 085 - Development and testing of nanoparticles for treatment of cancer cells by curie temperature controlled magnetic hyperthermia

#### Archil Chirakadze^1^, Nodar Mitagvaria^1^, Giorgi Chubinidze^1^, Nugzar Dvali^1^, Teimuraz Chichua^1^, Zakaria Buachidze^2^, Akaki Gigineishvili^3^, David Jishiashvi^4^, Mariam Abuladze^5^, Alexanderi Jishiashvili^6^

##### ^1^Caucasus International University, Scientific Centre for the Synthesis and Research of Chemotherapeutic Drugs, Tbilisi, Georgia ^2^Georgian Technical University, Archil Eliashvili Institute of Control Systems, Tbilisi, Georgia ^3^Georgian Technical University, Department of Engineering Physics, Tbilisi, Georgia ^4^Georgian Technical University, V. Chavhhanidze Institute of Cynernetics, Tbilisi, Georgia ^5^Kutaisi International University, Hadron Therapy Center, Kutaisi, Georgia ^6^Georgian Technical University, Department of Physical Engineering, Tbilisi, Georgia

Reviewing of about 500 scientific publications on cancer statistics, cancer incidence and mortality projections, the effectiveness of cancer therapy, financial performance of leading pharmaceutical companies and medical institutions, analysis of the market research reports, etc., explicitly indicates the continuous increase in demand for the more effective and convenient, less invasive and less expensive combined modalities for cancer treatment, at least up to 2035, not only not only for adults but also for children and adolescents. A vast amount of nanoparticles has been developed and proposed for the local hyperthermia of cancer during the last decades, but only a few of them correspond to the mandatory requirements of having therapeutic range Curie temperature (TC = 41-450 C), high-rate crystallinity and “strong” magnetic properties, strictly controlled homogeneity and dispersion of the nanoparticles, good biocompatibility and harmless decomposition products. Among them are the nickel-copper (Ni-Cu) and silver doped lanthanum manganite (AgxLa1-xMnO3) nanoparticles. The developed research showed that the materials obtained at lower than usual temperatures using microwave enhanced synthesizes and annealing can be successfully used for local hyperthermia revealing high crystallinity, high magnetic properties (appropriate and easily tuneable Curie temperature, saturation magnetization, remnant magnetization and coercivity) and appropriate specific absorption rate (SAR). Behavioural toxicity testing of developed nanoparticles was enhanced by blood oxygen saturation measurements using non-invasive oximetry in white rats. Both of the developed nanomaterials revealed a lower toxicity level than the commercially available Fe2O3 nanoparticles.

### P 086 - Microwave synthesis, characterization and testing of acute toxicity of boron nitride nanoparticles by monitoring of behavioural and physiological parameters

#### Archil Chirakadze^1^, Nodar Mitagvaria^1^, Giorgi Chubinidze^1^, Nugzar Dvali^1^, Teimuraz Chichua^1^, Zakaria Buachidze^2^, Akaki Gigineishvili^3^, David Jishiashvi^4^, Mariam Abuladze^5^, Alexander Jishiashvili^6^, Nana Khuskivadze^1^

##### ^1^Caucasus International University, Scientific Centre for the Synthesis and Research of Chemotherapeutic Drugs, Tbilisi, Georgia ^2^Georgian Technical University, Archil Eliashvili Institute of Control Systems, Tbilisi, Georgia ^3^Georgian Technical University, Department of Engineering Physics, Tbilisi, Georgia ^4^Georgian Technical University, V.Chavhanidze Institute of Cynernetics, Tbilisi, Georgia ^5^Kutaisi International University, Hadron Therapy Center, Tbilisi, Georgia ^6^Georgian Technical University, Department of Physical Engineering, Tbilisi, Georgia

Hexagonal boron nitride nanoparticles, nanosheets and nanotubes (BNNPs) are even more promising materials for biomedical application than carbon nanotubes (CNTs) and nanoparticles (CNPs) due to their negligible cytotoxicity. Nanostructured hexagonal boron nitride (h-BN) is a highly prospective material for the tumour localized boron capture therapy characterized by high biocompatibility, and significant efforts have been made to reduce the conventional synthesis and annealing temperature, improve the crystalline structure, as well as for more detailed study of the microwave enhanced synthesis and microwave properties of the hexagonal boron nitride and its compositions, encapsulating capacity and luminescence properties. The reported research yielded in development and testing of two distinctive microwave enhanced comparatively low-temperature methods of synthesis of the hexagonal boron nitride nanoparticles and nanosheets with reduced distortion of the crystal lattice, and an improved method of general toxicity testing of the developed nanomaterials utilizing continuous observation of behavioural effects in white rats in combination with blood oxygen saturation, systolic blood pressure and body temperature measurements in full agreement with the 4R principles of animal welfare in scientific research. The obtained results allow us to expect that the developed materials can be a good basis for developing highly effective modalities for anticancer (in combination with chemotherapy, hyperthermia and radiotherapy) and antiviral (in combination with chemotherapy and hyperthermia) treatment. The developed samples have significantly less disordered (turbostratic) crystal lattice than the materials treated at maximum temperatures of 4500С and ≈ 9000С.

### P 087 - Biomarker correlates of MC-ROR1771, a prospective trial evaluating stereotactic body proton radiotherapy with nivolumab for metastatic head and neck cancer

#### Daniel Ebner^1^, Scott Lester^1^, Mauricio Gamez^1^, David Routman^1^, Kathryn Price^2^, Haidong Dong^3^, Sean Park^1^, Ashish Chintakuntlawar^2^, Michelle Neben-Wittich^1^, Lisa McGee^4^, Yolanda Garces^1^, Samir Patel^4^, Robert Foote^1^, Jaden Evans^5^

##### ^1^Mayo Clinic, Radiation Oncology, Rochester, USA ^2^Mayo Clinic, Medical Oncology, Rochester, USA ^3^Mayo Clinic, Urology and Immunology, Rochester, USA ^4^Mayo Clinic, Radiation Oncology, Phoenix, USA ^5^Intermountain Healthcare, Radiation Oncology, Murray, USA

**Purpose**: Platinum-refractory recurrent head and neck squamous cell carcinoma (PR-HNSCC) patients have a median overall survival (mOS) of 6 months (CheckMate 141). The multisite prospective MC-ROR1771 trial evaluated stereotactic body proton therapy (SBPT) addition to nivolumab in this population, demonstrating mOS of 12.5 months and progression free survival (PFS) of 4.5 months. A planned biochemical marker/T-cell subpopulation analysis is presented here.

**Methods**: Patients with metastatic PR-HNSCC were eligible. Two cycles of nivolumab were followed by SBPT to 1-2 target lesion(s), with maintenance nivolumab thereafter. Variation in potential predictive biomarkers drawn during treatment were evaluated.

**Results**: Thirteen (13) patients received SBPT; one withdrew, with 3 laryngeal, 1 nasopharyngeal, and 8 oropharyngeal primaries. Regarding chemotherapy, 4 received one, 3 two, and 5 patients three cycles. 11 received first line chemoradiation; 1 received prior chemotherapy. SBPT ranged from 35-50 Gy(RBE1.1). T-cell population response was observed particularly following SBPT between days 15 and 30 post-nivolumab initiation. Bim+/CD8+CD11aHi was unchanged. Most patients experienced a decrease in %PD-1+/CD8+CD11aHi and TCM-CD8+CCR7+CD45RA- cells with nivolumab, with rebound after SBPT. The inverse was seen in TEM-CD8+CCR7-CD45RA- and TEF-CD8+CCR7-CD45RA+ cells. Further analysis is ongoing.

**Conclusion**: MC-ROR1771 demonstrated favorable OS/PFS versus CheckMate 141; biomarker analysis has demonstrated significant shifts in T-cell populations with systemic response noted both to initiation of nivolumab as well as following SBPT. Prospective evaluation of SBPT and immunotherapy appears warranted.

### P 088 - Clinical breast delineation versus HU-based mammary glandular tissue delineation for breast sparing in mediastinal Hodgkin lymphoma proton therapy

#### Pierre Loap^1^, Goudjil Farid^2^, Kirova Youlia^2^

##### ^1^Institut Curie, Department of Radiation Oncology, Saint-Cloud, France ^2^Institut Curie, Department of Radiation Oncology, Paris, France

**Purpose**: The aim of this study was to compare clinical breast and Hounsfield-unit (HU)-based glandular tissue volumes in terms of delineation and dosimetric parameters when planning proton therapy for mediastinal Hodgkin lymphoma.

**Methods**: The study population consisted in ten consecutive mediastinal Hodgkin lymphoma patients treated in a single center with involved-site pencil beam scanning proton therapy in an adjuvant setting to a total dose of 30Gy. The clinical breast, delineated based on anatomic landmarks and wire, was used for treatment optimization (clinical OAR) with a maximum tolerated mean dose was 4Gy, in accordance with the ILROG consensus. The mammary glandular tissue was retrospectively automatically delineated based on tissue density discrimination using minimal HU values of -80 (glandular OAR, figure 1). The mean and maximum doses to the clinical and glandular OAR were retrieved and compared, as well as their volume.

**Results**: Compared with the clinical OAR, the glandular OAR was characterized by a ten-time smaller average mean dose (p<0.01) and a twice smaller average maximum dose (p<0.01). The clinical OAR was significantly larger than the glandular OAR (p<0.01) (table 1).

**Conclusion**: When planning mediastinal Hodgkin lymphoma proton therapy, a clinical delineation woud allow a more conservative breast sparing than a HU-based glandular tissue delineation, which is more prone to radiation exposure underestimation.

### P 089 - Comparison of estimated late toxicities between IMRT and IMPT when treating prostate cancer with whole pelvis radiation postoperatively

#### Emile Gogineni^1^, Hao Chen^2^, Abhinav Reddy^2^, Curtiland Deville^2^

##### ^1^The Ohio State University, Radiation Oncology, Columbus, USA ^2^John Hopkins University, Radiation Oncology and Molecular Radiation Sciences, Baltimore, USA

**Purpose**: To compare estimated rates of late gastrointestinal (GI) and genitourinary (GU) toxicity between modern scanning beam intensity-modulated proton therapy (IMPT) and photon radiation therapy (IMRT) when treating prostate cancer with whole pelvis radiation postoperatively.

**Methods**: IMPT and volumetric modulated arc therapy (VMAT) IMRT plans were created for 10 consecutive patients receiving postoperative radiation for prostate cancer. The prescription was 50 Gy radiobiologic equivalent (GyE) (using proton radiobiological effective doses of 1.1) to the elective pelvic nodal regions and 70 GyE to the prostate bed, all in 2 GyE per fraction. The paired 2-sided Wilcoxon signed-rank test was used to compare differences in GI and GU toxicity risk using published multivariable normal tissue complication probability (NTCP) models.

**Results**: IMPT and IMRT plans provided similar target coverage. Doses to organs at risk (OARs) were significantly lower with IMPT than IMRT for bladder (V5-V65), bowel (V5-V45), and rectum (V5-V70). IMPT plans provided significantly lower mean doses than IMRT for all OARs. Estimated late toxicities were similar for GU endpoints but significantly lower with IMPT than IMRT for all GI endpoints, including grade 2 stool frequency (0.1 vs. 2.5%, p=.02), fecal incontinence (0.1 vs 1.0%, p<.00), and rectal bleeding (0.1 vs. 0.2%, p=.03).

**Conclusion**: IMPT provided significantly lower doses to all OARs in comparison with IMRT. This translated to significantly lower risks of estimated late toxicity for all GI endpoints with IMPT. Results from ongoing prospective trials will likely provide more definitive answers on any clinical benefits with the use of proton irradiation.

### P 090 - Clinical evidence of variable RBE for non-small cell lung cancer (NSCLC) patients treated with passive scattering proton therapy (PSPT)

#### Yulun He^1^, Antony Adair^2^, Guillaume Cazoulat^1^, Pablo Yepes^2^, Carol Wu^3^, Peter Balter^2^, Julianne Pollard-Larkin^2^, Zhongxing Liao^4^, Radhe Mohan^2^, Kristy Brock^1^

##### ^1^The University of Texas MD Anderson Cancer Center, Imaging Physics, Houston, USA ^2^The University of Texas MD Anderson Cancer Center, Radiation Physics, Houston, USA ^3^The University of Texas MD Anderson Cancer Center, Diagnostic Imaging, Houston, USA ^4^The University of Texas MD Anderson Cancer Center, Radiation Oncology, Houston, USA

**Purpose**: To derive proton variable RBE (RBE_var_) from radiographic changes and validate with pneumonitis predictions using dose distributions weighted by RBE=1.1 vs RBE_var_.

**Materials and Methods**: Clinical data for NSCLC patients previously treated with IMRT (N=51; dose: D_x_) or PSPT (N=67; dose, dose-averaged LET: D_p_, LET_d_) to 60-74Gy(RBE=1.1 for PSPT) on a prospective randomized trial was acquired. Individually, a ∼4-month post-treatment CT was deformed to the planning image to compute the voxel-level image density change (IDC) in the normal ipsilateral lung as clinical evidence of response. RBE values were empirically measured as D_x_/D_p_ corresponding to the same IDC levels. Subsequently, these RBE values were used to fit a linear-quadratic model (McNamara, α/β=3Gy). For each PSPT patient, NTCP scores for grade >=2 pneumonitis (Lyman model with TD_50_=34.8Gy, m=0.22, and n=0.5) using dose distributions weighted by RBE=1.1 and the modelled RBE_var_ were compared.

**Results**: The figure shows the voxel-level dose-IDC relationships, from which the RBE_var_ was modelled (Table). The fitted model parameters were p_0_=8.56, p_1_=14.24, p_2_=2.44, and p_3_=−0.34 (pseudo-R^2^=0.98). When comparing the average NTCP scores for patients with vs without pneumonitis, RBE_var_-based NTCP scores increased 3% as compared to 1% for RBE=1.1. The patient with the largest NTCP increase from using RBE=1.1 to RBE_var_ (95%) received average LET_d_ of 15.67keV/µm in the total lung and developed grade 3 pneumonitis.

**Conclusion**: Evidence of variable RBE was demonstrated in voxel-based radiographic changes as a metric of response. The data suggests the potential of variable RBE to better predict pneumonitis.

### P 091 - Optimizing complementary therapies to avoid anesthesia for AYA patients receiving proton therapy

#### Ravish Kapoor^1^, Karen Moody^2^, Brandon Gunn^3^, Susan McGovern^3^

##### ^1^University of Texas M.D. Anderson Cancer Center, Anesthesiology and Perioperative Medicine, Houston, USA ^2^University of Texas M.D. Anderson Cancer Center, Pediatrics, Houston, USA ^3^University of Texas M.D. Anderson Cancer Center, Radiation Oncology, Houston, USA

**Background**: For adolescent and young adult (AYA) patients, the risks of anesthesia are increased compared to younger (< 12 years old) patients. Additionally, as a free-standing facility, resources to manage anesthesia-related complications at our proton center are limited compared to centers physically attached to comprehensive hospitals. Consequently, the use of anesthesia for patients 12 years of age and older at the proton center needs to be critically considered. Given the iterative and prolonged nature of proton therapy, the need for complementary approaches to minimize the use of anesthesia is critical.

**Methods**: To help providers identify alternative strategies to the use of anesthesia, our center recently developed an algorithm for maximizing the use of non-anesthesia interventions in patients 12 years old and older. These are designed to address the most common referral reasons for anesthesia in AYA patients, pain and anxiety. Through early referral to our Pediatric Supportive Care group, pharmacologic and non-pharmacologic methods for managing pain and anxiety can be tailored to the patient’s unique needs. Additionally, age-appropriate support from Child Life Specialists can help patients through all aspects of the treatment process.

**Results and Conclusion**: For AYA patients preparing for proton therapy, pain and anxiety often trigger requests for anesthesia. Through an algorithm-based optimization of non-anesthesia techniques for addressing these issues, including early involvement of Pediatric Supportive Care specialists, patient-specific strategies can be developed that allow for the delivery of proton therapy without anesthesia.

### P 092 - Immunomodulation by partial tumor irradiation using protons and carbon-ions: Clinical exploitation of bystander and abscopal effects

#### Slavisa Tubin^1^, Piero Fossati^2^, Antonio Carlino^3^, Markus Stock^3^, Eugen Hug^4^

##### ^1^EBG MedAustron GmbH, Medical Department, Wiener Neustadt, Austria ^2^Medaustron- Center for Ion Therapy, Radiation Oncology, W. Neustadt, Austria ^3^Medaustron- Center for Ion Therapy, Medical Physics, W. Neustadt, Austria ^4^Medaustron- Center for Ion Therapy, Radiation Oncology, W. Neustadt, Austria

**Background**: A novel immunomodulatory approach for PArtial Tumor irradiation of HYpoxic segment (PATHY) sparing the Peritumoral Immune Microenvironment (PIM) was developed to add to direct radiation tumor cell killing also the component of immune-mediated killing. The hypothesis is that for effective immunomodulation, the entire tumor volume may not need to be irradiated but only a partial volume to initiate the immune cycle in radiation-spared PIM. The aim was to assess the impact of PATHY on tumor downsizing, tumor control and survival among patients affected by recurrent unresectable bulky tumors.

**Materials and Methods**: Fifteen (15) consecutive patients were treated between March 20 and October 22. According to the Palliative Prognostic Index, all patients had a life expectancy of <3months. 63% of patients had sarcoma while 37% other unresectable bulky tumors. The prescription dose was 30, 36 or 45 Gy RBE in 3 fractions.

**Results**: Median follow up was 9.2 months. Overall survival and progression-free survival at last follow up were 64% and 46%, respectively. Median patient survival was 7.1 month (estimated was < 3 months). Local tumor control was 46% following the single PATHY course. Bystander effect was induced in 73% of patients. Average GTV volume reduction was 60%. In 3 out of 5 metastatic patients (60%) abscopal effects were reported.

**Conclusions**: PATHY was effective, safe and well tolerated treatment. It resulted in improvement in symptoms and quality of life of highly complex patients without associated treatment related toxicity. This approach showed high immunogenic and neoadjuvant potential that potentially can improve radiotherapy therapeutic ratio.

### P 093 - Can we treat prostate cancer patients without an endorectal balloon using spot scanning proton therapy?

#### Mei Chen^1^, Yan Yuanlin^1^, Zhou Hui^1^, Jiang Xuming^1^, Li Huan^1^, Cao Lu^1^, Xu Cheng^1^, Chen Jiayi^1^

##### ^1^Ruijin Hospital- Shanghai Jiao Tong University School of Medicine, Department of Radiation Oncology, Shanghai, China

**Purpose**: This study aimed to investigate whether it is feasible to treat prostate cancer patients without an endorectal balloon for stabilizing the prostate in spot scanning proton therapy (SSPT).

**Materials and Methods**: Fifteen intermediate-risk prostate cancer patients were included. The prescribed dose was 60 Gy(RBE)/30fx to CTV1 and 76 Gy(RBE)/38fx to CTV2. Treatment plans were robustly optimized in RayStation 10B accounting for 5 mm setup uncertainties and 3.5% range uncertainty. Each patient was administered glycerol suppositories for emptying the rectum 30 minutes before daily treatment and had a verification CT every week. Deformable registration was carried out between the verification CT and the planning CT. The interfraction motion was quantified as the shift of the target’s centroid relative to the planning CT. Variations in the dose to 95% (D95) and 5% (D5) of the target were evaluated.

**Results**: The maximum displacements for CTV1 (6.5 mm) and CTV2 (6 mm) were observed in the anterior-posterior direction. The maximum shift of the target in the right-left and inferior-superior directions were within 3.1 mm and 4.5 mm, respectively. Compared to the planning dose, the delivered dose of CTV1 and CTV2 varied within 1% concerning D95 and D5. The maximum deviation in target coverage was observed in the first week for CTV1 D95 (-0.8%) and the final week for CTV2 D95 (0.4%).

**Conclusion**: By emptying the rectum, the interfraction motion of the prostate was within 6 mm and the delivered dose deviated by within 1%. It is feasible to treat prostate cancer patients without an endorectal balloon using SSPT.

### P 094 - Does proton arc therapy increase the risk of secondary malignancy in patients treated for breast cancer?

#### Perry Johnson^1^, Maria Mamalui^1^

##### ^1^UF Health Proton Therapy Institute, Radiation Oncology, Jacksonville, USA

**Purpose**: Compared to IMPT, proton arc therapy (PAT) is expected to improve dose conformality, delivery efficiency, and provide a more favorable LET distribution. As a tradeoff, the low-dose bath is spread out over larger volumes which could impact the likelihood of developing a radiation-induced malignancy. The goal of this study was to evaluate this comparative risk using available models, specifically for breast radiotherapy.

**Methods**: PAT and IMPT treatment plans were created for five representative patients using an alpha build of RayStation 12B. Risk estimates were based on the organ-equivalent dose (OED) concept using Schneider’s site-specific, mechanistic dose-response models for carcinoma induction. Organs included the ipsilateral, contralateral, and whole lung, contralateral breast, thyroid, and esophagus. Lifetime Attributable Risk (LAR_70_) was calculated using an age of exposure of 45, a 5-year latent period for cancer induction, and a maximum age of 70.

**Results**: In each case, OED for the thyroid and esophagus was ≤0.5Gy with negligible LAR_70_. OED_avg_ for the contralateral breast was slightly higher when using PAT (0.33Gy vs 0.24Gy), however, the difference in LAR_70_ was minor (0.09% vs 0.07%). OED_avg_ for the ipsilateral lung was lower when using PAT (1.21Gy vs 1.61Gy) with a similar difference in LAR_70_ (1.22% vs 1.61%).

**Conclusion**: In PAT of the breast, dose is extended laterally but is more conformal along the chest wall. The associated dose reduction in the lung is estimated to decrease the risk of secondary cancer induction in this organ by 0.4% with negligible change to the risk profile in other organs.

### P 095 - Application of a multipurpose Proton Arc Therapy treatment planning algorithm to an abdominal phantom and retrospective ependymoma case

#### Sam. Burford-Eyre^1^, Michael Merchant^2^, Peter Sitch^3^, Ranald Mackay^2,3^, Adam Aitkenhead^2,3^, Robert Appleby^1^

##### ^1^University of Manchester, Physics and Astronomy, Manchester, United Kingdom ^2^University of Manchester, Division of Cancer Sciences- Faculty of Biology- Medicine and Health, Manchester, United Kingdom ^3^The Christie NHS Foundation Trust, Christie Medical Physics and Engineering, Manchester, United Kingdom

A versatile Proton Arc Therapy (PAT) planning algorithm was developed using the matRad research treatment planning system, supporting single and multi-field optimisation (SFO/MFO); single and dual arc delivery; and robust optimisation. IMPT and PAT plans were generated using CTs from an abdominal phantom and retrospective ependymoma patient. SFO and MFO were applied separately to single arc plans. Dual arc delivery was simulated by distributing the predetermined optimal energies across one clockwise and anti-clockwise arc. For all plans, appropriate nominal and worst-case dose and LET metrics were recorded. For both cases, most worse-case CTV and OAR dose and LET metrics of the default MFO single arc plans were comparable to their IMPT counterpart. In the ependymoma case, PAT provided further spinal cord sparing with = [44.53,47.26Gy] and = [5.67, 7.33keVμm-1] for PAT and IMPT respectively. In the abdominal phantom, SFO and dual arc delivery both separately improved CTV coverage relative to the default PAT plan with = [99.58%, 99.65%, 98.96%] respectively. In contrast, SFO and dual arc deliver caused CTV coverage to fall below clinical thresholds for the more complex ependymoma case, with = [95.17%, 97.41%, 98.02%] for SFO, dual arc delivery and the default PAT plan respectively. SFO and dual arc delivery caused no significant difference to in all ROIs. Further work using more patient datasets across multiple treatment sites is required to understand the dosimetric and biological benefits of PAT relative to IMPT.

### P 096 - Can proton planning be more like photon planning?

#### Rock Mackie^1^, Niek Schreuder^2^

##### ^1^Leo Cancer Car, NA, Verona, USA ^2^Leo Cancer Care, Research, Knoxville, USA

The workhorse technology for photon beam radiotherapy is co-planar, intensity-modulated multi-field therapy delivered typically by 6 MV, beams. Not only high-quality treatment planning standardized but image-guidance workflow is routinized making delivery more efficient. Coplanar proton radiotherapy is today used in the vast majority of treatments. Vertex fields are used in some few-field brain radiotherapy cases because it offers a way to spread the entrance dose but arc therapy with effectively many beam directions also spreads the entrance dose (and exit dose - for which proton radiotherapy has a distinct physical advantage). Adding more coplanar entrance directions will almost always accomplish spreading the entrance dose so that is less than photon rotational therapy for tumors. Not changing the couch angle greatly improves workflow and eliminates concerns about the patient moving when the couch is moved. For proton radiotherapy for tumors that are significantly closer to one side of the patient than the opposite side, the proton beams can be arranged to come through the closer side to minimize the integral dose to normal tissue and indeed to increase the volume of normal tissue that has almost no deposited dose. If non-coplanar delivery is rare and coplanar planning and delivery methodologies can be implemented which enables sufficient avoidance of normal tissue then the vast majority of patients can be treated with coplanar beams which do not require a rotational beam gantry if the patient can be setup and maintained stably in the upright position and a fixed horizontal beam used.

### P 097 - Novel approach to three-dimensional image guidance to deliver prostate stereotactic body proton therapy in a fixed beam room

#### Shaakir Hasan^1^, Francis Yu^1^, Chin-Cheng Chen^1^, Daniel Gorovets^2^, Stanislav Lazarev^3^, Madhur Garg^4^, Keyur Mehta^4^, Arpit M. Chhabra^1^, J. Isabelle Choi^1^, Charles B. Simone II.^1^

##### ^1^New York Proton Center, Radiation Oncology, New York, USA ^2^Memorial Sloan Kettering Cancer Institute, Radiation Oncology, New York, USA ^3^Mount Sinai, Radiation Oncology, New York, USA ^4^Montefiore Medical Center, Radiation Oncology, New York, USA

**Introduction**: Localized prostate cancer is typically treated with two opposed lateral proton beams in a fixed-beam room (FBR). However, FBRs lack cone-beam computed tomography (CBCT) to optimally evaluate rectal and bladder filling, thereby raising safety concerns of stereotactic body proton therapy (SBPT) in FBRs. We, therefore, performed out-of-room evaluation CTs (eCT) with a simulation CT scanner right before each prostate SBPT treatment in a FBR and present preliminary clinical and dosimetric data herein.

**Methods**: Consecutive patients with rectal diameter <3cm at simulation were assessed. All patients received an enema before each fraction and had fiducials and a rectal spacer. Patients received an initial ultrasound bladder volume scan followed by eCT to evaluate rectal/bladder filling, and if approved by physician would then be transferred to a FBR where beams were aligned to fiducials. Dosimetric and differences between CT-sim and eCT were calculated.

**Results**: Seven patients with 35 pre-treatment eCTs were analyzed. The median time between eCT and FBR treatment start was 19 minutes. In 2/35 fractions, patients were rescanned due to distended rectums, requiring rectal evacuation. The mean differences between eCT and CT-sim for FBR patients are per the table: All patients used prophylactic Flomax. There were no instances of acute grade 2+ GI/GU adverse events, and mean difference in IPSS score 1-month and 3 months post-treatment relative to baseline were +5 and -1, respectively.

**Conclusion**: In well selected patients, pre-treatment eCT can fulfill the role of CBCT to evaluate rectal and bladder filling and safely enable prostate SBPT in a FBR.

### P 098 - A proton craniospinal irradiation (CSI) planning procedure

#### Elena Antolin^1^, Borja Aguilar^1^, Pablo Cabello^1^, Alberto Viñals^1^, Roser Fayos-Sola^1^, Miguel Garcia^1^, Carlos Ramon^1^, Juan Diego Azcona^1^, Jose Miguel Delgado^1^, Diego Pedrero^1^

##### ^1^Clínica Universidad de Navarra, Medical Physics and Radiation Protection, Madrid, Spain

**Materials and Method**: The treatment planning system (TPS) used is RayStation (RaySearch Laboratories) with Monte Carlo Dose Calculation Algorithm. The Proton Therapy System modeled is a ProBeat-CR (Hitachi). It is proven that Proton Therapy may deliver low entry doses and almost no exit dose. Here we explain the standard scheme that we use to get systematically good results for CSI: A minimum of 2 isocenters separated by a maximum of 30 cm.

- Isocenter 1: It is placed on a vertebra next to the larynx. Therefore, the CBCT will better control the correct matching of the neck. We program three fields with different gantry angles: Iso1_G180°, Iso1_G90°, Iso1_G270°.
- Isocenter 2 (a third one would follow the same pattern): It will only differ in the craniocaudal coordinate towards the feet, and will only have a field (Iso2_G180°)

The Clinical Target Volume (CTV) will be split in 8 Optimization Target Volumes (OTVs) which do not overlap:

- OTV_Brain, OTV_neck, junctions 1-5, OTV_Spine (With another isocenter we will have other 6 volumes: 5 more junctions and an OTV_Spine2)

Links field-target:

- Iso1_G90, Iso1_G270 aims to OTV_Brain
- Iso1_G180 aims to OTV_Brain, OTV_neck and junctions 1-5.
- Iso2_G180 aims to junctions 1-5 and OTV_Spine.

**Results**: A highly conformal and uniform dosimetry is obtained, while avoiding OARs and considering 3 mm and 3% patient position and density uncertainties respectively, to make it robust.

**Conclusions**: To obtain a safe plan from the beginning, it is worth investing time, placing the isocenters, splitting contours, and conscientiously defining objectives and constraints in the optimization step.

### P 099 - Successful PBT treatment for agitated elderly with esophageal cancer under BIS-guided intubation-free anesthesia

#### Kuan-Ching Huang^1^, Bing-Shen Huang^1^, Jen-Yu Cheng^1^, Meng-Wei Ho^1^, Eng-Yen Huang^1^, Shao-Chun Wu^2^, Yu-Ming Wang^1^

##### ^1^Kaohsiung Chang Gung Memorial Hospital and Chang Gung University College of Medicine, Department of Radiation Oncology & Proton and Radiation Therapy Center, Kaohsiung 833, Taiwan Province of China ^2^Kaohsiung Chang Gung Memorial Hospital and Chang Gung University College of Medicine, Department of Anesthesiology, Kaohsiung 833, Taiwan Province of China

An 80-year-old woman with questionable dementia presented with cervical esophageal squamous cell carcinoma and retro-aortic LN metastasis, cT4bN2M1G2, visited our clinic. After MDT, definitive chemoradiation was recommended thus local radiotherapy was arranged. The prescribed dose was 50 Gy (RBE) in 25 fractions to the gross tumor and LNs, mediastinum, and bilateral SCF. During her first-time simulation procedure, difficulties in fixation and breathing coordination was noted due to the fluctuation of her dementia symptoms. The respiratory signals captured by the Anzai system were not in concordance and the procedure was failed. After discussion with our anesthesiologist, intravenous general anesthesia (IVGA) under bispectral index (BIS) guidance to maintain her optimal sedation level was recommended. The suppression time of BIS shown she undoubtfully had weak, aged, and easily vulnerable to general anesthetics. Under BIS guidance, she underwent simulation, 4DCT image acquisition, and daily PBT treatment course as the BIS was kept at 40-60 after she lost her consciousness and limb movements without the requirement of intubation. The respiratory signals were with high concordance under her voluntary breathing and there were no involuntary limb movements noticed. To the best of our knowledge, this is the first experience of BIS-guided IVGA for patients with difficulties following PBT instruction. The respiratory coordination was highly reproducible throughout the treatment course. BIS-guided IVGA could be considered and is now an option at our center for patients with clinical indications.

### P 100 - A PBS system for ocular melanoma

#### Jatinder Saini^1^, Jonathan Chen^1^, Andrew Stacey^2^, Lia Halasz^1^, Charles Bloch^1^

##### ^1^Fred Hutchinson Cancer Center/University of Washington, Radiation Oncology, Seattle, USA ^2^Department of Ophthalmology/University of Washington, Ocular Oncology, Seattle, USA

Ocular treatments have mostly been confined to dedicated beamlines with superior beam properties. Enabling the newer general-purpose spot scanning beamlines to treat ocular could increase access and usher in modern technologies such as full 3-D imaging (CT and MRI) for treatment planning and dose calculation, Monte Carlo algorithms, and LET/RBE evaluation. We have developed a complete solution to treat ocular targets at the Fred Hutchinson Proton Therapy Center using the general-purpose IBA pencil beam scanning beamline and mainly commercially available tools. A gaze setup and monitoring system were designed to aid in eye immobilization during CT and treatment delivery. The gaze angle is determined before the CT based on diagnostic MR information, and the optimum angle depends on the tumor size, location, and projected beam arrangement. Patients are implanted with three tantalum markers before the CT simulation that are used for daily IGRT. For contouring, 3D (CT and MRI) and Fundus images are used for target delineation. Dose calculations will be performed on full patient CT using one or more beams and the RayStation MC algorithm. As this is a high-energy beamline, a range shifter is mounted 50cm upstream from the isocenter to obtain lateral penumbras similar to dedicated beamlines. Patient-specific apertures are inserted into the beam path to allow for a sharp lateral penumbra of 1.5 to 2.5 mm. We will give an overview of gaze management, target contouring, treatment planning, and dose calculation.

### P 101 - Particle Therapy Co-Operative Group Genitourinary Subcommittee consensus statement on the use of proton therapy for the management of testicular seminoma

#### Seungtaek Choi^1^, Charles Simone^2^, Iain Macewan^3^, Huan Giap^4^, Shaakir Hasan^2^, Daniela Branco^5^, Kosj Yamoah^6^, Nancy Mendenhall^7^

##### ^1^M.D. Anderson Cancer Center, Radiation Oncology, Houston, USA ^2^New York Proton Center, Radiation Oncology, New York, USA ^3^University of California San Diego, Radiation Oncology, San Diego, USA ^4^Medical University of South Carolina, Radiation Oncology, Charleston, USA ^5^University of California San Diego, Radiation Physics, San Diego, USA ^6^Moffitt Cancer Center, Radiation Oncology, Tampa, USA ^7^University of Florida, Radiation Oncology, Jacksonville, USA

**Introduction**: Proton therapy offers significant dosimetric advantages over photon therapy in the treatment of testicular seminoma patients. However, due to decreased availability and increased cost of this treatment compared to photons, the use of proton therapy has been limited. The goal of this consensus statement by the PTCOG GU Subcommittee is to familiarize practitioners with proton therapy so that seminoma patients who would benefit would be offered that treatment more readily.

**Results**: Several publications have demonstrated benefits of protons over photons. Efstathiou et al and Simone et al showed decreased radiation dose to the normal tissues with protons, with both hypothesizing a lower risk of secondary cancers with protons. A comparison plan between intensity-modulated proton therapy (IMPT), intensity-modulated radiation therapy (IMRT) with photons, and 3D conformal photon therapy is shown in Figure 1. Based on these data, and through consensus voting, we recommend that patients who meet criteria listed in Table 1 be strongly considered for proton therapy.

**Conclusion**: Published data show that proton therapy is safe and effective in the treatment of testicular seminoma. Furthermore, proton therapy reduces the excess radiation dose to normal tissues, which should lead to decreased risks for complications. Therefore, proton therapy may not only improve clinical outcomes, but it may also offer a more cost-effective treatment over the lifetime of a patient.

### P 102 - Dosimetric and NTCP analyses for selecting parotid gland cancers patients to proton beam therapy

#### Anna Maria Camarda^1^, Maria Giulia Vincini^2^, Stefania Russo^1^, Stefania Comi^2^, Francesca Emiro^2^, Alessia Bazani^1^, Rossana Ingargiola^1^, Barbara Alicja Jereczek^2^, Ester Orlandi^1^, Daniela Alterio^2^

##### ^1^National Center for Oncological Hadrontherapy CNAO, Radiation Oncology Department, Pavia, Italy ^2^IEO-European Institute of Oncology- IRCCS, Division of Radiation Oncology, Milan, Italy

**Purpose/Objective**: To perform a dosimetric and a Normal Tissue Complication Probability (NTCP) analyses between Intensity Modulated Proton Therapy (IMPT) and Volumetric Modulated Arc Therapy (VMAT) in a cohort of patients (pts) with parotid gland cancers (PGCs) in a post-operative or radical setting.

**Materials and Methods**: From May 2011 to September 2021, 37 PGCs pts treated at two Institutions were eligible. Inclusions criteria were as follows: pts aged ≥ 18 years old, diagnosis of PGCs candidate for PORT or definitive RT, presence of written informed consent for the use of anonymous data for research purposes. Target coverage was defined as D95 > 98%. Six NTCP models were selected. NTCP profiles were calculated for each patient using an internally-developed Python script in RayStation TPS. Average NTCP profiles were tested for significance with a two-sided Wilcoxon signed-rank test.

**Results**: Seventy-four (74) plans were generated. A lower Dmean to the majority of OARs was obtained with IMPT vs VMAT with statistically significance (p < .05). Dosimetric results and average NTCP profile are shown in Table 1 and Figure 1. Ten (27%) pts had a ΔNTCP_x-p_ >10% for hearing loss and/or tinnitus: among them, 7 qualified for both endpoints, 2 pts only for hearing loss, and 1 for tinnitus.

**Conclusions**: In the current study, IMPT showed a dosimetric advantage for most of all OARs over VMAT. Nearly one-third of pts resulted eligible for PT and they are the most likely to benefit in terms of prevention of hearing loss and tinnitus.

### P 103 - Single-isocenter multi-target (SIMT) stereotactic radiosurgery (SRS) with particle arcs: Proof-of-concept using protons, helium and carbon ions

#### Stewart Mein^1,2^, Lennart Volz^3^, Thomas Tessonnier^1^, Peilin Liu^4^, Andrea Mairani^1^, Christian Graeff^3^, Xuanfeng Ding^4^, Taoran Li^2^

##### ^1^Heidelberg Ion-beam Therapy Center HIT, Biophysics in Particle Therapy BioPT, Heidelberg, Germany ^2^Perelman School of Medicine of the University of Pennsylvania, Department of Radiation Oncology, Philadelphia, USA ^3^GSI Helmholtzzentrum für Schwerionenforschung, Biophysics, Darmstadt, Germany ^4^Beaumont Health System, Department of Radiation Oncology, Royal Oak, USA

**Purpose**: Single-isocenter multi-target (SIMT) stereotactic radiosurgery (SRS) is the state-of-the-art standard of care for patients with multiple brain metastases (MBM). Recent developments in particle arc therapy demonstrate the potential for high-precision, efficient arc delivery using light and heavy ions. Here, particle-arc based multi-target SRS for treating MBM is presented.

**Methods**: Planning and delivery strategies for spot-scanning proton/hadron arc (SPArc and SHArc) were developed within a multi-institutional collaboration for non-coplanar SRS with particle arcs using protons, helium and carbon ions. Dedicated spot/energy/arc selection procedures were developed using clinical/research systems (RayStation/TRiP/FRoG) for multi-arc treatments. Photon VMAT SRS (HA-VMAT) treatments were generated using the HyperArc platform (Varian) for cases exhibiting 3-12 brain lesions and prescription of 15-18Gy in a single fraction. HA-VMAT plans were optimized with PTV=GTV+1mm. SPArc/SHArc plans were optimized applying 2.5%/1mm robustness criteria for range/positioning uncertainty.

**Results**: All particle arc plans were highly conformal to the PTV with sharp fall-off and a reduced low-dose bath compared to HA-VMAT (Figures 1&2). V_2Gy_ and V_4Gy_ were similar or reduced for ion arc compared to HA-VMAT. SHArc plans exhibited similar or improved brain volume metrics depending on number of targets, tissue-type and RBE model, with substantial increases in GTV-LET (e.g., ∼>100keV/µm for ^12^C). Estimated delivery times ranged from ∼3-45 minutes depending on number of lesions and sub-arcs.

**Conclusions**: SIMT-SRS using particle arc therapy shows promise in improving target conformality, reducing low-dose volumes in brain (<2-4Gy) and/or increasing LET levels for combating radio-resistant tumors. Further development is underway, e.g. efficient optimization and delivery for cases with >5 targets.

### P 104 - Initial clinical experience with proton reirradiation: A optimized therapeutic option

#### Jose Javier Aristu Mendioroz^1^, Rosa Meirino^1^, Javier Serrano^1^, Mauricio Cambeiro^1^, Jacobo Palma^1^, Diego Pedrero^2^, José Miguel Delgado^1^, Carlota Guisbert^1^, Felipe Calvo^1^

##### ^1^Clinica Universidad de Navarra, Radiation Oncology, Madrid, Spain ^2^Clinica Universidad de Navarra, Radiophysics, Madrid, Spain

**Objectives and purpose**: Proton therapy (PT) emerges as a therapeutic alternative to deliver radical radiation doses minimizing the risk of toxicity in previously irradiated structures. The early experience based on post-selected patient referral evaluation in a PT Unit is analyzed.

**Material and Methods**: Between 04/2020-03/2022, 94 patients (med 58-yo) have been treated with proton-reirradiation (PT-rRT). Most patients were referred from Spanish regions (80%) and the remaining were international origin. The distribution by tumor location was as follows: 28 (30%) CNS, 18 (19%) head and neck, 16 (17%) sarcoma, 9 (10%) GI tumors, 7 (7%) prostate and 16 (17%) miscellaneous. The median time elapsed between the first RT and P-rRT was 29 months (3m-470m) and 60 Gy (18Gy-72Gy) were the median initial RT-dose.

**Results**: The median CTV volume, P-rRT prescribed dose, number of fractions and dose fractionation were 70cc (2.5cc-3769cc), 54 Gy (18Gy-72Gy), 20 (5-35) and 3Gy (1.5Gy-7Gy). The total EQ2 administered (initial RT+P-rRT) for a/b-3 y a/b-10 were 57.2 Gy and 54.7, respectively. Twenty-six out 52 patients experienced symptoms relief. Fifteen (16%) patients had acute toxicity grade 3 or higher. With a MFU of 13.4 months (2m-24.8m), grade-3 or higher late toxicity was registered in 15 patients (16%). 6-12m OS have been 91%-84%, respectively.

**Conclusions**: Recurrent/second tumors arising on previously irradiated oligo-symptomatic patients is an increasingly common clinical scenario in radiotherapy practice. PT-reRT emerges as an optimized treatment recommendation for providing durable tumor control and potential definitive cancer control in selected patients without significant excess of severe toxicity.

### P 105 - Feasibility of Dynamic Proton Arc Lattice therapy: Dose and LETd comparison vs. conventional IMPT

#### Maria Mamalui^1^, Perry Johnson^1^

##### ^1^University of Florida Health Proton Therapy Institute, Therapy Medical Physics, Jacksonville, USA

**Purpose**: Spatially fractionated radiation therapy (SFRT, both 3D Lattice and GRID) has been shown to have such therapeutic advantages as significant tumor reduction with a single dose (≥15Gy) followed by conventional fractionation and high pain relief rate while maintaining superior OAR sparing. The aim of this work is exploring possible additional features/advantages from implementing proton lattice SFRT via Dynamic Proton Arc Therapy (DPAT) pencil beam scanning.

**Methods**: For several representative patient cases of bulky chordomas/sarcomas, 12 treatment plans were generated: IMPT and DPAT plans for lattice CTV targets (LCTV) with center-to-center (CTC) distances 1.5-3cm between spherical vertices; clinical/delivered IMPT plan and DPAT plan to prescribed CTV^RX^. All plans were created using Raystation^TM^. Physical dose (peak-to-valley-dose-ratio/PVDR) and LETd metrics were compared between modalities. Achievable PVDRs and non-optimized (i.e., occurring by virtue of spatial fractionation only) LETd were compared vs target geometry (varying CTCs).

**Results**: For CTV^RX^, DPAT improved LETd^max^ from 2.6 (IMPT) to 4 (DPAT) kV/μm (averaged over all studied cases). For lattice SFRT targets, IMPT has slightly over-performed in average LETd (by 0.2 kV/μm) with nearly identical LETd^max^ achieved via DPAT and IMPT. Much greater difference was observed in achievable PVDRs: with varying LCTV CTCs, average 3DPVDRs ranged [1.8-10.4] in DPAT plans while IMPT resulted in 3DPVDRs [1.2-7.2].

**Conclusion**: DPAT results in higher achievable peak-to-valley-dose-ratios vs IMPT regardless of specific target CTC. DPAT enhances LETd within CTV^RX^ and LCTVs vs non-SFRT IMPT clinical plans. However, this enhancement is marginally smaller than LETd improvement produced by a non-LET-optimized IMPT plans.

### P 106 - Robust plan-of-the-day IMPT strategy for locally advanced cervical cancer

#### Eva Negenman^1,2^, Sander Kuipers^1,2^, Jérémy Godart^1,2^, Anouk Corbeau^3^, Andras Zolnay^1^, Stephanie de Boer^3^, Remi Nout^1^, Mischa Hoogeman^1,2^

##### ^1^Erasmus MC Cancer Institute - University Medical Center Rotterdam, Department of Radiotherapy, Rotterdam, Netherlands ^2^HollandPTC, Department of Medical Physics & Informatics, Delft, Netherlands ^3^Leiden University Medical Center, Department of Radiation Oncology, Leiden, Netherlands

**Purpose**: Treating locally advanced cervical cancer (LACC) with IMPT is challenging due to anatomic variations within the pelvis. Appropriate robustness and margin settings for a plan-of-the-day strategy for LACC IMPT are currently unknown. The aim of this study is to propose such settings, by evaluating the accumulated target dose for simulated treatment courses.

**Methods**: Fourteen patients treated for LACC were included in this study. With an empty and full-bladder planning-CT, ITVs were created. A library of 1-4 plans was created. ITV margins were determined to encompass the low-risk CTV (CTV-LR) in 90% of repeat-CTs, acquired weekly during treatment. IMPT plans were created with 5 mm set-up and 3% range robustness. Ten treatments per patient were simulated by recalculating the chosen plan-of-the-day on four repeat-CTs, with additional simulated treatment uncertainties. Target coverage was evaluated on CTV-LR and elective CTV (CTV-E).

**Results**: Anisotropic margin of 1-1;7-5;3-3mm in LR;AP;CC directions were found adequate to encompass CTV-LR with ITV. The treatment simulations showed that V95%>42.75Gy for 99% of the treatments for the CTV-LR and 92% for the CTV-E (see Fig.1). The most common regions with dose <42.75Gy were identified in the CTV-E against the vertebrae (3/14 patients), in the cervix posteriorly (3/14), and in the uterus anteriorly (3/14) (see Fig.2).

**Conclusion**: The margin and robustness settings for the plan-of-the-day strategy was robust to motion, geometric uncertainties, and range uncertainties. Both targets met the dose criterion in more than 90% of the patients, indicating opportunities to further optimize the balance between adequate target coverage and normal tissue exposure.

### P 107 - Dosimetric analysis of the proton therapy adaptation in Krakow

#### Marzena Rydygier^1^, Anna Spaleniak^1^, Magdalena Garbacz^1^, Dawid Krzempek^1^, Gabriela Foltynska^1^, Eleonora Gora^2^, Kamil Kisielewicz^2^, Tomasz Skora^2^, Antoni Rucinski^1^, Renata Kopec^1^

##### ^1^Institute of Nuclear Physics Polish Academy of Sciences- PL-31342 Krakow- Poland, Cyclotron Centre Bronowice, Krakow, Poland ^2^National Oncology Institute- National Research Institute, Krakow Branch, Krakow, Poland

In proton therapy random treatment, uncertainties are frequently addressed by applying tumor volume safety margins or robust optimization. Further unforeseeable changes in the patient anatomy occur due to, e.g., tumor shrinkage or sinus filling. In this study the findings of adaptive radiation therapy (ART) of perisinusoidal and brain areas are presented. At the Cyclotron Centre Bronowice (CCB), in cooperation with the National Oncology Institute, control CT scans are typically taken weekly and are further used for ART if necessary. ∼10% of patients treated in Krakow between 2020 and 2022 underwent ART, while most of the tumors were located in the head-and-neck area (∼92.5%). Seven patients with perisinusoidal and brain tumors have been selected for dosimetric analysis and divided into two groups according to ART motivation: (i) treatment-related reasons such as changes in PTV; (ii) reasons unrelated to radiotherapy such as mucosal filling of the sinuses or patient’s anatomy changes with preservation of PTV volume. The adaptive treatment plans were forward-calculated on the reference treatment planning CT. The OAR DVH parameters were compared between adapted and non-adapted plans to demonstrate the dosimetric benefit from adaptations performed in Krakow. Decreased dose exposure of OARs was observed more often (aprox. 63%) during ART using new CT scans (group ii) than new PTV contours only (aprox. 35%; group i). Nevertheless, there was no substantial relationship between ART and the dose in OARs. The patient database will be updated in the future.

### P 108 - RBE variability in proton therapy: Collecting evidence in clinically observed side effects

#### Martina Palkowitsch^1,2^, Fabian Hennings^1,2^, Mechthild Krause^1,2,3,4,5^, Jona Bensberg^6^, Christian Hahn^1,5,6^, Feline Heinzelmann^7,8,9^, Christian Bäumer^6,7,8,9^, Armin Lühr^6^, Beate Timmermann^7,8,9^, Steffen Löck^1,3,4,5^

##### ^1^OncoRay – National Center for Radiation Research in Oncology, Faculty of Medicine and University Hospital Carl Gustav Carus - Technische Universität Dresden - Helmholtz-Zentrum Dresden – Rossendorf, Dresden, Germany ^2^Helmholtz-Zentrum Dresden - Rossendorf, Institute of Radiooncology – OncoRay, Dresden, Germany ^3^National Center for Tumor Diseases NCT - Partner Site Dresden - Germany: German Cancer Research Center DKFZ, Heidelberg - Germany - Faculty of Medicine and University Hospital Carl Gustav Carus - Technische Universität Dresden - Dresden - Germany - and Helmholtz Association/Helmholtz-Zentrum Dresden - Rossendorf HZDR, Dresden, Germany ^4^German Cancer Consortium DKTK - partner site Dresden, and German Cancer Research Center DKFZ, Heidelberg, Germany ^5^Department of Radiotherapy and Radiation Oncology, Faculty of Medicine and University Hospital Carl Gustav Carus - Technische Universität Dresden, Dresden, Germany ^6^TU Dortmund University, Department of Physics, Dortmund, Germany ^7^West German Proton Therapy Center Essen WPE, University Hospital Essen, Essen, Germany ^8^German Cancer Consortium DKTK - partner site Essen, and German Cancer Research Center DKFZ, Heidelberg, Germany ^9^Clinic for Particle Therapy, University Hospital Essen, Essen, Germany

**Introduction**: Uncertainties in RBE hamper exploiting the full potential of proton beam therapy (PBT). We recalibrated normal tissue complication probability (NTCP) models developed for a constant RBE=1.1 considering a variable RBE and evaluated model performance.

**Material and Methods**: Seventy-six (76) patients with primary brain tumors treated with PBT were analyzed. Dose distributions were computed based on a constant RBE of 1.1 (D_constRBE_) and a variable RBE obtained from the Wedenberg et al. model (D_varRBE_) using different α/β values. Dose-volume parameters were inserted into 10 published NTCP models that were developed for PBT and a constant RBE=1.1. These NTCP models were recalibrated to physician-rated patient toxicity data based on D_constRBE_ and D_varRBE_, resulting in different models (NTCP_constRBE_ and NTCP_varRBE_, respectively). Model performance was evaluated by the area under the curve (AUC).

**Results**: On average, dose-volume parameters considering a variable RBE were higher than those based on a constant RBE=1.1, resulting in higher toxicity estimates from the original NTCP models (Figure 1). After recalibration (Table 1), NTCP models based on large OARs (skin, brain-CTV) showed minor differences (<2%) in outcome prediction between NTCP_constRBE_ and NTCP_varRBE_. Larger AUC differences (6-15%) were observed for models based on small OARs (cochlea, brainstem) or maximum dose. The impact of adjusting α/β of the respective OAR was small (<5%).

**Conclusion**: For some side effects based on either maximum dose or small OAR volumes, we obtained better performance for models using D_varRBE_ instead of D_constRBE_. This implies that NTCP models based on D_constRBE_ may not be optimal and require a re-development using D_varRBE_-parameters.

### P 109 - Variable dose-weighted linear-energy-transfer factor of radiobiological doses for the intraprostatic-tumor pencil-beam-scanning proton therapy

#### Zhong Su^1^, Curtis Bryant^2^, Mark Artz^2^, Wen Hsi^1^

##### ^1^University of Arkansas for Medical Sciences, Radiation Oncology, Little Rock, USA ^2^University of Florida Health Proton Therapy Institute, Radiation Oncology, Jacksonville, USA

**Purpose**: To compare the radiobiological-equivalent (RBE) doses between dose-weighted Linear-Energy-Transfer (LET_d_) and constant 1.1 factors over 12 patients having the focal intraprostatic-tumor IPT within prostate.

**Methods**: The simultaneous-integrated-boost (SIB) plan using the constant 1.1 factor with pencil-beam-scanning protons was constructed with Cobalt-equivalent CGE 85.8 Gy to IPT and 78.0 CGE to prostate. The optimization of each SIB plan achieved the dosimetric goals on targets and organ-at-risks as the standard treatment of 78.0 Gy. We utilized a linear-slope model; 1+K*LET_d_ with K=0.055, to recalculate the RBE doses using the spot-patterns of optimized plan with constant RBE. We utilized a sigmoid function to extract quantitative values for presenting the trend of LET_d_ volume histogram (LVH) of IPT and prostate extracted from RayStation system.

**Results**: The mean of LET_d_ with its standard-deviation StDeV (keV/A) for IPT are 2.32/0.18 and 2.47/0.18, while for prostate are 2.20/0.12 and 2.32/0.19 for initial and boost plans, respectively. Because of the small <0.02 StDeV for both IPT and prostate, the doses of variable RBE were similar as the dose of constant RBE.

**Conclusions**: Because only few percentages increasing and small deviation of overall dose distributions between the variable and constant RBE, the tumor control between variable and constant RBE doses are similar. However, large mean LET_d_ value for bladder, rectum, and femoral heads are up to 3.0 KeV with a tail of LETd value up to 5.0-6. KeV. A quantitative analysis of the complex LVH distribution need to be further conducted for evaluating the normal tissue complication probability.

### P 110 - Proteomics to assess the impact of LET on early biological response in the clinic

#### Wolfgang Sauerwein^1,2^, Mitsuko Masutani^3^, Andrea Wittig^4^, PierLuigi Mauri^5^

##### ^1^University Hospital Essen, NCTeam- Department of Radiation Oncology, Essen, Germany ^2^Deutsche Gesellschaft für Bor-Neurtoneneinfangtherapie DGBNCT, Board of Directors, Essen, Germany ^3^Nagasaki University Graduate School, Department of Molecular and Genomic Biomedicine- CBMM, Nagasaki, Japan ^4^University Hospital Jena, Klinik Für Strahelntherapie und Radioonkologie, Jena, Germany ^5^Consiglio Nazionale delle Ricerche, Istituto di Tecnologie Biomediche CNR-ITB, Segrate MI, Italy

**Purpose**: It is known that high and low LET radiation have different effects on the modulation of immunogenic responses. Unfortunately, clinical observations are not yet available to a sufficient extent to target this in the clinic. Clinical studies with a tumor response as an endpoint are expensive and require long follow-up periods. Proteomics offers another approach targeting early molecular effects.

**Method**: Non-invasive samples, such as urine and plasma, will be collected prior and after radiotherapy to investigate useful molecular profiles in relation to the disease and to evaluate differential effects of high and low LET at the molecular level. Such data will allow more accurate hypotheses to be developed regarding the differing effects of high and low LET and their potential clinical application.

**Results and Discussion**: At our best knowledge, such clinical trials have never been performed in hadrontherapy. But our group has used proteomics to demonstrate the specificity and efficacy of targeted nanoparticles as a new theranostic agent (Rainone et al., Int. J. Nanomed, 2021), the effects of gene therapy on children affected by WAS (Sereni et al., JACI 2019), and it was possible to predict responders to mAb therapy in severe asthma subjects (Mauri et al, Imm.Lett. 2014). PTCOG is the best platform to find partners for such a study using protons and carbon ions in standard treatment protocols. The results will help to better understand the differential effects of high and low LET radiation to guide both treatment protocols and the selection of appropriate patients for treatment.

### P 111 - Fast, GPU-accelerated Monte Carlo LET spectra scorers for proton treatment planning and optimisation

#### Jan Gajewski^1^, Angelo Schiavi^2^, Paulina Stasica^1^, Angelica De Gregorio^2^, Dawid Krzempek^1^, Marzena Rydygier^1^, Damian Borys^3^, Renata Kopeć^1^, Antoni Rucinski^1^

##### ^1^Institute of Nuclear Physics Polish Academy of Sciences, Medical Physics Department, Kraków, Poland ^2^Sapienza Università di Roma, Dipartimento di Scienze di Base e Applicate per l’Ingegneria, Rome, Italy ^3^Silesian University of Technology, Department of Systems Biology and Engineering, Gliwice, Poland

Proton radiotherapy is recognized as an effective treatment method of tumours allowing to spare normal tissues and minimize side effects. While a variable radiation quality is currently disregarded in proton treatment planning, new approaches employ dose-averaged proton Linear Energy Transfer (LETd) to quantify this effect. However, since no correlation was observed between therapy side effects and average LETd distributions in recent studies, it is hypothesized that LETd is not a sufficient descriptor of radiation quality in a given point of the mixed radiation field, and the LET spectra, consisting of high- and low-LET components should be considered. To include LET spectra in treatment planning and optimization, fast MC methods for the LET spectra scoring are needed. The LET spectra scorers have been implemented in the fast, GPU-accelerated MC FRED [1], developed at the Sapienza University in Rome, Italy. Along with the extensively validated beam model [2], the methods allow computation of the LET spectra in each voxel for patient treatment plans. We present the first results of the LET spectra scorer in voxelized geometry, validated against corresponding GATE/Geant4 MC calculations. Figure 1 presents the LET spectra at given points of the mixed radiation field produced by a single proton pencil beam in water. The methodology presented in this work is the first step to improve the precision of modelling of physical and biological effectiveness during proton radiotherapy and for development of LET spectra-based treatment planning and optimization methods.

### P 112 - LET-optimized planning in proton therapy: Can we minimize the risk of the radiation necrosis with TPS and Monte Carlo calculations?

#### Magdalena Garbacz^1^, Jan Gajewski^1^, Eleonora Gora^2^, Kamil Kisielewicz^2^, Dawid Krzempek^1^, Marzena Rydygier^1^, Tomasz Skora^2^, Renata Kopec^1^

##### ^1^Institute of Nuclear Physics Polish Academy of Sciences, Cyclotron Centre Bronowice, Krakow, Poland ^2^National Oncology Institute - National Research Institute, Krakow Branch, Krakow, Poland

Proton therapy (PT) provides the ability to treat tumors with high-radiation doses and preserve dose limits to critical organs located around the target volume. However, exceeding the dose constraints is not the only concern when planning treatment with charged particles. Studies in recent years show that not only the dose distribution (DD) but also the linear energy transfer (LET) can affect the risk of brain necrosis. The LET or dose-weighted LET (LETd) distribution is valuable information, which might affect the strategy of beam arrangement during treatment planning. To provide this information, the Monte Carlo (MC) methods can be used to simulate the irradiation prior to the treatment. In this work we will present the outcome of an implemented workflow combining the treatment planning system (TPS) Eclipse 16.1 and GPU accelerated MC code FRED. The treatment plans of patients receiving PT and with diagnosed brain changes will be reoptimized using LETd distributions from MC simulations in order to reduce the LETd values in areas with diagnosed necrosis. Fig1 shows LETd and DD for LET-optimized (Plan1) and the clinical plan (Plan2). Tab1 summarizes the LETd values for both plans. LETd was on average 12% lower in brain changes for LET-optimized plan. The results show that the proposed method can be used to avoid the high LETd and risk of brain changes for systems which are not equipped with LET-optimization software.

### P 113 - Analysis and reduction of the uncertainty in clonogenic assays to allow modeling the RBE as a function of the LET

#### Natalia Chamorro Claver^1^, Pedro Arce^1^, Juan Ignacio Lagares^1^, Marta Ibañez^1^, Miguel Angel Morcillo^1^, Juan Diego Azcona^2^, Marta Oteo^1^, Borja Aguilar^2^, José Manuel Pérez^1^

##### ^1^CIEMAT Center of Energy- Environment and Technology Research, Medical Applications of Ionizing Radiation Unit, Madrid, Spain ^2^Clinica Universidad de Navarra, Radiophysics and radiological protection, Madrid, Spain

Published data from clonogenic assays to study the dependence of RBE on LET show high variability among them. Uncertainty in the final result is not reported or explained in detail in many of the reviewed articles. It is therefore difficult to model phenomenological RBE as a function of dose, LET and α/β and therefore their potential application in clinical treatments. We propose a tool that estimates the RBE measurement uncertainty considering the contribution of each factor which also serves to optimize the experimental setup. This analysis shows that, while the biological uncertainties of the SF measurements have the biggest contribution in the final α/β uncertainty, the contribution of dose uncertainties can be also very high. Therefore, it is crucial to minimize the uncertainty of dose deposition, which is usually not done. To reduce the dose uncertainty, the PDD of the proton is measured with a method that allows to reduce the common uncertainty of 100-200µm to 20µm. That means a reduction of the uncertainty in α and β due to dose from 0.692Gy^−1^ to 0.0322Gy^−1^ and from 3.23Gy^−2^ to 0.0102Gy^−2^, for highest LETs. Other parameters such as the number of measurements or the maximum dose (minimum SF measured) can also reduce the final uncertainty around 10-20%. On the other hand, using an optimal beam spatial distribution can reduce the dose deposition uncertainty to less than 3%. In conclusion, we have designed a tool to automatically compute uncertainty in clonogenic assays and used it to reduce the uncertainty of our measurements.

### P 114 - Single proton LET characterization with the Timepix detector and artificial intelligence for advanced proton therapy treatment planning

#### Antoni Rucinski^1^, Paulina Stasica^1^, Hanh Nguyen^2^, Carlos Granja^3^, Lukas Marek^3^, Cristina Oancea^3^, Marzena Rydygier^1^, Keith Schubert^2^, Reinhard Schulte^4^, Jan Gajewski^1^

##### ^1^The Henryk Niewodniczanski Institute of Nuclear Physics Polish Academy of Sciences, Cyclotron Centre Bronowice, Kraków, Poland ^2^Baylor University, Department of Electrical and Computer Engineering, Waco, USA ^3^ADVACAM s.r.o., Research and Development Department, Prague, Czech Republic ^4^Loma Linda University, Division of Biomedical Engineering Sciences, Loma Linda, USA

Clinical proton beams produce mixed radiation fields consisting of particles ranging from low- to high-linear energy transfer (LET), the latter of which yields an increased radiobiological effect. Prediction of LET of primary and secondary particles at a given point of the radiation field in a patient can be performed by Monte Carlo simulations but is difficult to verify experimentally. The single particle tracking with the semiconductor pixel detector Timepix and the developed convolutional neural network allowed us to measure the deposited energy and resolve the type of each particle comprising the mixed radiation field produced by a therapeutic proton beam in a water phantom (fig.1). Protons’ LET spectra and dose-averaged LET (LETd) values were computed and compared with GATE/Geant4 simulations. An accuracy of over 95% was achieved for proton recognition. Measured protons’ LET spectra and LETd are compared with the results of simulations (fig.2). We measure a broad spectrum of LET values ranging from a fraction of keV/μm to about 10 keV/μm for most performed measurements. The introduced method can be applied to validate any treatment planning system that includes LET-based optimization. Its commercial accessibility makes it easy to be translated into a clinical routine in any proton therapy facility to support treatment planning and quality assurance.

### P 115 - Progress and developments with TURBO: A system enabling fast energy switching for rapid beam delivery

#### Jacinta S. L. Yap^1^, Adam Steinberg^1,2,3^, Adam Yeo^4^, Hannah Norman^1,2,3^, Robert Appleby^2,3^, Suzie Sheehy^1,5^

##### ^1^University of Melbourne, Physics, Melbourne, Australia ^2^University of Manchester, Faculty of Science and Engineering, Manchester, United Kingdom ^3^Cockcroft Institute, Accelerator Physics, Warrington, United Kingdom ^4^Peter MacCallum Cancer Centre, Medical Physics, Melbourne, Australia ^5^Australian Nuclear Science and Technology Organisation ANSTO, Centre for Accelerator Science, Sydney, Australia

The prevalence of charged particle therapy (CPT) has grown significantly in recent years yet prohibitive facility costs and technological limitations still impact access, availability, and utility. The beam delivery system (BDS) determines several aspects of the beam parameters and the capability to provide high quality treatments. There are several challenges related to the costs, complexities, and constraints with existing systems; new and emerging methodologies such as FLASH, Arc, multi-ion therapies require improvements with current beam delivery technologies. A limiting constraint is related to the energy layer switching time (ELST) as the BDS must adjust when delivering across layers of different beam energies: this can account for a large amount of the beam delivery time, impacting treatment efficacy and efficiency. The ‘Technology for Ultra Rapid Beam Operation’ (TURBO) project is a novel BDS in development at the University of Melbourne, utilising novel Fixed Field Alternating Gradient optics to increase the energy acceptance to allow multiple beam energies to be transported given the same fixed fields, thereby reducing the ELST and enabling rapid beam delivery across the tumour depth. We present recent progress with ongoing development toward the realisation of a proof-of-concept demonstrator adapted for low energy protons. Design and simulation modelling work are shown for various components of the system alongside experimental measurements. Treatment focused studies investigate the feasibility and capabilities of a scaled-up system. Further plans overview the potential and clinical benefits of TURBO as a fixed-beamline BDS with fast energy switching for CPT.

### P 116 - Development of next-generation proton therapy system

#### Shu Nakajima^1^, Daizo Amano^1^, Nagaaki Kamiguchi^1^, Takuya Miyashita^1^, Kenzo Sasai^1^

##### ^1^Sumitomo Heavy Industries- Ltd, Industrial Equipment Division, Shinagawa-ku, Japan

Increasing the number of patients eligible for proton therapy has been a challenge in terms of clinical usefulness and business viability. Sumitomo Heavy Industries (SHI) has developed a next-generation proton therapy system to enhance the utility of proton therapy and improve its business feasibility. In particular, to improve its clinical usefulness for respiratory moving organs, SHI attempted to realize high-dose and short-time irradiation by using a new accelerator and ultra-high-speed scanning. To shorten the irradiation time, the maximum scanning speed in the lateral directions was increased to 100 m/s, five times faster than the conventional model. The energy switching time was reduced to 100 ms, 1/3 of that of the conventional model. To increase the dose rate, a superconducting cyclotron was newly developed and the proton beam current was increased to 1000 nA, 3.3 times that of the conventional model. In 2022, a proton irradiation test was conducted at SHI’s factory (Ehime, Japan) using a test model of the next-generation proton therapy system. Key performance characteristics of the next-generation system, such as energy switching time, scanning speed, and beam positioning accuracy, were demonstrated, as well as the shortening of irradiation time when simulating an actual target. With the realization of high-dose and short-time irradiation, irradiation can be completed within 6 seconds for large targets such as large hepatocellular carcinomas and within 2 seconds for the lungs, with only one breath-hold. This technology is expected to improve treatment accuracy, shorten treatment time, and reduce the burden on technicians and patients.

### P 117 - Achieving higher delivery efficiency using a large momentum accepttance superconducting gantry

#### Bin Qin^1^, Xu Liu^1^, Yicheng Liao^1^, Wei Wang^1^, Aote Chen^1^, Zhaoxia Yuben^1^, Qushan Chen^1^, Dong Li^1^

##### ^1^Huazhong University of Science and Technology, School of Electrical and Electronic Engineering, Wuhan, China

**Background**: Superconducting (SC) gantry can be applied to proton therapy to significantly reduce both the footprint and overall weight of the gantry system. However, relative lower ramping limit of the SC magnetic field becomes an bottle-neck for energy change and efficient beam delivery.

**Purpose**: To design a large momentum accepttance (LMA) beam optics can overcome the requirement for fast magnetic field regulation for matching various proton beam energy. Meanwhile, LMA features have potential to achieve fast beam delivery with high beam transmission.

**Methods**: Alternating-gradient Canted Cosine-Theta (AGCCT) superconducting magnets are used for bending section with local dispersion suppression. Generic algorithm is applied to optimize both high order optics and realistic magnetic fields configuration. TOPAS is used to simulate beam energy degrading process to evaluate beam transmission.

**Results**: +/−10% momentum acceptance of the gantry beamline can be achieved with a relative small magnet aperture for AG-CCT. It is expected to cover more than 40% clinical cases by using fixed magnetic fields during treatment. With this feature, reduction of spots and energy layers can signicantly reduce the overall treatment time. Volumtric scanning is under study by expanding the spots from 2D to 3D.

### P 118 - A new spot-scanning beam delivery method based on the large momentum acceptance beamline in proton therapy

#### Wei Wang^1^, Xu Liu^1^, Yicheng Liao^1^, Benzhaoxia Yu^1^, Zhiyong Yang^2^, Bin Qin^1^

##### ^1^Huazhong University of Science and Technology, State Key Laboratory of Advanced Electromagnetic Engineering and Technology- School of Electrical and Electronic Engineering, Wuhan, China ^2^Huazhong University of Science and Technology, Cancer Center- Union Hospital- Tongji Medical College, Wuhan, China

Proton therapy has become a significant treatment option for many tumors. The large momentum acceptance beamline based on the super-conducting magnet technique has become an important direction for the miniaturization of proton therapy facilities. Besides greatly reducing the size and weight of facilities, it can also transport a large energy spread beam, which can reduce the beam loss and the number of needed energy layers during irradiation. However, the large energy spread beam increases the lateral and distal penumbra. Therefore, to solve the problem, we propose a new spot-scanning beam delivery method based on the large-momentum acceptance beamline using the superconducting magnets technique. A variable aperture collimator and movable energy collimator are introduced in the beamline, in order to provide two different beams. When scanning the peripheral part of the tumor, the small aperture collimator and the energy collimator are used to provide smaller lateral and longitudinal size beams, whereas using larger beams provided by using the large aperture collimator to irradiate the inner part of the tumor. The new beam delivery method is verified theoretically and compared with the traditional spot-scanning delivery method. The results show that the new delivery method not only achieves miniaturization but also has a smaller lateral and distal penumbra, as well as better dose uniformity. Additionally, it needs fewer energy layers and spots to cover the target, thus improving the delivery efficiency.

### P 119 - Research on the impact of the MRI machine’s fringe magnetic field on skin dose during proton therapy

#### Lei Zhang^1^, Ming Wang^1^, GuoDong Li^1^

##### ^1^Chengdu university of technology, College of Nuclear Technology and Automation Engineering, Chengdu, China

When MRI technology is used in image-guided proton therapy, a non-uniform magnetic field (up to 1.3 T) is introduced between the treatment head and the patient’s body. Now more than previously, the magnetic field has an impact on the secondary particle transport mechanism in the beam. Under the effect of a proton beam, the widely used range-shifter material polymethylmethacrylate (PMMA) generates a high number of secondary particles, the bulk of which are electrons. These electrons, which are mostly concentrated in skin tissue, are extremely susceptible to magnetic field rotation. This study intends to examine the effects of the isocenter, range shifter, and fringe magnetic fields on skin tissue injury. First, a thorough analysis of the impact of the fringe magnetic field on skin dose deposition using TOPAS’s physical model of the SC200 superconducting proton therapy device was conducted. The fringe magnetic field’s effect on secondary particles in the beam before the water tank’s entrance is being investigated. The findings indicate that the fringe magnetic field mostly impacts the isocenter electron flow (approximately 89.13%) and reduces the likelihood of skin damage from 200 MeV, 150 MeV, and 100 MeV proton beams by 1.0%, 0.95%, and 9.20%, respectively. When the range shifter is nearer the isocenter, this phenomenon is more pronounced. The fringe magnetic field’s border currently aligns with the model’s center. The entrance to the water model will be located within a homogenous magnetic field in the next stage, which will consider a scenario more like the actual treatment scenario.

### P 120 - Development of ripple filter using multi-layered metal mesh

#### Sodai Tanaka^1^, Taku Inaniwa^1^, Shunya Matsuba^1^

##### ^1^National Institutes for Quantum Science and Technology, Institute for Quantum Medical Science, Chiba, Japan

Plates with a fine ridge and grooves structure have been used as a ripple filter (RiFi) in carbon-ion therapy. However, the Bragg peak width broadened by a RiFi strongly depends on the precision of microfabrication, and surface dose inhomogeneity was caused by the structure. We developed a RiFi using multi-layered metal mesh (mRiFi), which can easily be made to order, and the dependencies of the mRiFi parameters such as wire diameter, wire spacing, wire material, and number of the layers were evaluated. The mRiFi was made by stacking 25 aluminum meshes with a diameter of 0.29 mm and a wire-to-wire spacing of 1.27 mm at random positions and angles (figure 1). Due to the randomness of its structure, the water equivalent thickness varies at different beam path, resulting in a beam energy distribution. The microstructure prevents the lateral surface dose inhomogeneity, and microfabrication technique is not necessary, leading to low cost. As experimental results, the Bragg peak was successfully broadened in the depth dose distribution of the beam modulated with the mRiFi (figure 2). The acquired distribution agreed with the distribution obtained by convolving the depth dose distribution of the pristine beam with the normal distribution with σ=1.5 mm. In Monte Carlo simulation, the Bragg peak width of the modulated beam agreed with the analytical calculations. We developed the mRiFi consisting of the multi-layered metal mesh, and clarified the dependencies of the mRiFi parameters.

### P 121 - An overview on an innovative proton therapy solution

#### Luigi Picardi^1^, Raffaele Andrea Prisco^1^, Giovanni Rutigliani^1^, Roberto Orecchia^1^, Lidia Strigari^2^

##### ^1^Linearbeam srl, Particle Accelerator, Ruvo di Puglia, Italy ^2^IRCCS Azienda Ospedaliero-Universitaria di Bologna, UOC Fisica Sanitaria - Dipartimento Malattie oncologiche ed ematologiche, Bologna, Italy

**Introduction**: Proton therapy is a complex but consolidated technology. The current positive rate of diffusion favours, technological developments aimed at both a reduction in term of costs and performance. Interesting developments are underway in the use of linear accelerators for proton production, conceptually similar to the accelerators used in conventional radiotherapy.

**Method**: The system consists of a Linear Accelerator for protons composed of an injector and a series of Radio Frequency modules that release the proton beam along a horizontal line. The timing and intensity of the pulses, accompanied by a low emittance, offers the possibility to delivery an optimal dose in terms of precision following even on moving organs.

**Results**: The Linearbeam proeject is a low-cost, integrated device designed to provide proton treatments to patients by delivering a predetermined dose of radiation to a predetermined three-dimensional treatment target volume, thereby protecting the patient and hospital staff from unnecessary radiation exposure and other risks.

**Conclusion**: The system is innovative since the combination of a linac capable of producing a pulsed beam that largely exceed the therapy needs with a gantryless delivery system makes the facility specifically intended for a very fast 4D proton therapy treatment and reduces the overall plant costs with almost the same target in number of patients of a facility equipped with a gantry. In the test site in Ruvo di Puglia (Ba), Italy, the accelerator characterization is in progress, as well as the robotic arms and their control. The treatment planning system specific for this apparatus is under CE mark.

### P 122 - Beam delivery and patient alignment: Perspective of the innovative normandy hadrontherapy (NHA) C400ions multi-ion system

#### Virgile Letellier^1^, Stefan Schmidt^2^, Olivier Cosson^1^, Paul-Francois Doubliez^3^, Philippe Velten^1^, Dirk Dölling^3^, Marc De Leenheer^1^

##### ^1^Normandy Hadrontherapy, Physics, Caen, France ^2^Particle Therapy Consultant, Physics, Overath, Germany ^3^Ion Beam Applications, Physics, Louvain-la-Neuve, Belgium

The C400IONS system is the first multi-ion particle therapy system based on a cyclotron world-wide. The first treatment delivery is foreseen in Caen, France in 2026. This work provides major beam delivery and patient alignment specifications and design evaluations that will be integrated in the first installation. The C400IONS is an innovative system developed on a 400 MeV/u isochronous cyclotron, it can generate carbon ions, protons and helium ions. The beam delivery and treatment room design of this multi-ion treatment system is based on the IBA Proteus®PLUS equipment. The C400IONS fixed horizontal beamlines use pencil beam scanning technology and provide, e.g., planar imaging, a 6D robotic patient positioning system coupled with either a couch or a chair, and options to use patient or treatment specific accessories like a ripple filter. Nozzle and chair have been designed for optimal clinical perspectives for adult and pediatric treatments. The Beam Delivery System will provide scan fields from 20x20cm^2^ for carbon ions up to 30x30cm^2^ for protons. Beams will be available for treatment with ranges between 4 and 32 g/cm^2^ for protons and helium ions and between 4 and 27 g/cm^2^ for carbon ions. The nozzle shape and bottom clearance have been optimized for a chair designed for brain and head and neck treatment with several backrest heights and rotation settings enabling a large treatment volume. This work describes design investigations by NHa and its partners on nozzle and patient positioning components, supporting a reliable, comprehensive, optimized and evolutive multi-ion therapy system.

### P 123 - Heavy ion radiotherapy system with advanced scanning magnet and gantry in Yonsei University Health System

#### Tomoya Shimada^1^, Kosuke Nakanishi^1^, Masaki Asano^1^, Yoshifumi Nagamoto^1^, Chae-Seon Hong^2^, Min Cheol Han^2^, Changhwan Kim^2^, Jin Sung Kim^2^

##### ^1^Toshiba Energy Systems and Solutions Corporation, Particle Radiotherapy Business Project Engineering Department, Kawasaki, Japan ^2^Yonsei University Health System, Department of Radiation Oncology- Yonsei University College of Medicine, Seoul, Korea Republic of

This presentation describes the new heavy ion radiotherapy system installed in Yonsei University Health System in Korea. It consists of one fixed beam treatment room and two compact superconducting gantry treatment rooms. It is the world’s first heavy ion radiotherapy facility with multiple gantries with advanced 3D scanning system. The construction and installation have been finished in the facility during last 2 years. Clinical commissioning in the horizontal fixed beam port and the beginning of treatments are scheduled in 2023. Toshiba’s advanced scanning irradiation system is installed in each irradiation port and our fast 3D scanning system can be downsized the total footprint of whole facility. The pencil beam scanning technique and respiratory gating system enable to distribute high conformity of dose distribution at tumor targets. Our system also provides a high-speed 3D scanning method in which the fast 2D scanning is performed slice by slice, and the fast multi-energy extraction is made to control a depth of each slice. No use of range shifters will contribute to reduction of scattering effect and secondary reactions to patients as well. Toshiba has also developed a compact gantry system with the rotating angle of 360 degrees by utilizing our superconducting technologies and the downsized scanning system. It allows various and superior treatment plans using a carbon-ion beam with the flexibility as same as that of the proton and x-ray radiotherapy. Toshiba will provide the rotating gantry system as a standard configuration of heavy-ion radiotherapy to give comfort to patients and medical staff.

### P 124 - Development of the extended collimator for accelerator-based boron neutron capture therapy

#### Hiroaki Kumada^1^, Yinuo Li^2^, Kenta Takada^3^, Kei Nakai^1^, Susumu Tanaka^1^, Takashi Sugimura^4^, Masaharu Sato^4^, Akira Matsumura^5^, Takeji Sakae^1^, Hideyuki Sakurai^1^

##### ^1^University of Tsukuba, Faculty of Medicine, Tsukuba, Japan ^2^University of Tsukuba Graduate School, Comprehensive Human Sciences, Tsukuba, Japan ^3^Gunma Prefectural College of Health Sciences, Department of Radiological Technology, Maebashi, Japan ^4^High Energy Accelerator Research Organization, Accelerator Laboratory, Tsukuba, Japan ^5^Ibaraki Prefectural University of Health Sciences, President, Ami- Inashiki, Japan

iBNCT project has produced “iBNCT001”, a demonstration device of a linac-based treatment device for boron neutron capture therapy (BNCT). iBNCT001 generates neutrons by irradiating an 8 MeV proton beam to a beryllium target. We have developed extended collimators in addition to normal collimators. A feature of the extended collimator is that the surface of the beam aperture protrudes 100 mm from the surrounding wall. Protruding of the beam surface particularly helps to avoid interference between the patient’s shoulder and the wall in the irradiation of a head and neck cancer patient. To confirm the performance of the neutron beam released from the extended collimator, several measurements with a water phantom have been performed. Fig.1 shows a picture taken of an experiment that was performed to measure the thermal neutron flux distributions inside the phantom set at the irradiation position with the extended collimator. Fig. 2 shows the thermal neutron flux distributions on the central axis in the water phantom for each collimator. The maximum thermal neutron flux in the phantom was approximately 8.0 × 10^8^ (cm^−2^s^−1^). The intensity of the extended collimator was reduced by approximately 40% at each depth, relative to the normal beam collimator. However, both distribution profiles were in close agreement. These results demonstrate that the extended collimator can be applied to the treatment though irradiation time increases to approximately 55 min.

### P 125 - Broadened bragg peak shapes for breath-hold treatment: A comparative study

#### Isabella Colizzi^1,2^, Vivek Maradia^1,2^, Damien Charles Weber^1,3,4^, Antony John Lomax^1,2^, David Meer^1^, Serena Psoroulas^1^

##### ^1^Paul Scherrer Institut, Center for Proton Therapy, Villigen, Switzerland ^2^ETH, Department of Physics, Zurich, Switzerland ^3^Bern University Hospital- University of Bern- Inselspital, Department of Radiation Oncology, Bern, Switzerland ^4^University, Hospital, Zurich, Switzerland

The need to reduce treatment time for treating moving targets and the interest in FLASH have attracted attention to ridge filters (RF) as modulated devices that can expand the Bragg peak (BP) shape. Since there are several examples of RFs, each generating different broadening in the Bragg peak in the literature, we have studied three distinct Bragg peak forms and determined which one produces the best spot (Bragg peak) and energy reduction while maintaining target coverage in two breath-hold patient scenarios. The RFs were designed using an analytical model and fine-tuned with TOPAS-MC simulations, also used to generate depth dose curves. Figure 1 shows the three designed RFs and their corresponding depth dose curves. Two patient cases were investigated, a liver and a lung, each with multiple organs at risk (OAR). Dose distributions were optimized to provide the same target coverage for each RF (Fig. 2). DVHs (fig 2) show that RF3 delivers more dose to the OAR than other RFs due to the larger distal fall-off. Additionally, RF3 offers the greatest reduction in the number of spots, while RF3 and RF2 show similar results in terms of energy layer reduction (Fig. 2). RF1 delivers the lowest dose to OAR but has the worst spot and energy reduction. We conclude that “gaussian-like” RFs offer the best balance between spot/energy layer reduction and OAR sparing for the investigated cases. These results provide insight for future studies with RFs to investigate the feasibility of efficiently treatment delivery of moving targets with breath-hold.

### P 126 - Integration of beam monitoring and range verification systems for advanced carbon ion beam delivery

#### Simona Giordanengo^1^, Elisa Fiorina^1^, Felix Mas Milian^1,2^, Mohammed Abujami^1,3^, Emanuele Data^1,3^, Veronica Ferrero^1^, Sahar Ranjbar^1,3^, Francesco Pennazio^1^, Marco Pullia^4^, Roberto Sacchi^1,3^

##### ^1^Instituto Nazionale di Fisica Nucleare INFN, Section of Torino, Torino, Italy ^2^Universidade Estadual de Santa Cruz, Departamento de Ciencias Exactas e Tecnológicas, Ilhéus, Brazil ^3^Università degli Studi di Torino, Dipartimento di Fisica, Torino, Italy ^4^Centro Nazionale Adroterapia Oncologica CNAO, Research Division, Pavia, Italy

**Introduction**: We propose an innovative beam delivery system for carbon ions integrating new beam monitors and range verification tools. The new system exploits thin silicon detectors[1], segmented in strips, able to detect single ions at clinical rates and to directly measure their delivery time.

**Matherials and Methods**: A new technology based on 4D particle tracking is in progress within the INFN-SIG project for faster beam monitoring with unprecedented sensitivity to beam parameters (fluence, position, shape and energy). Additionally, the timing information is expected to boost the performances of the existing in-vivo range verification systems [2], allowing for better background rejection in the identification of fast decaying positron emitters and for exploiting the gamma timing technique.

**Results**: Preliminary tests were carried out correlating in time the signal of an 8-strips thin silicon detector, providing the crossing time with 50 ps resolution, with the signal of prompt gamma detectors. As an example, Figure 1 shows the coincidence between the measured signal of a single primary proton (blue peak) and of the secondary photon (yellow curve) starting with a few nanoseconds of delay. Similar results are expected with carbon ions considering the large signals from single ions and the excellent time resolution measured with 60 µm thick sensors as shown in Figures 2_Left and 2_Right.

**Conclusions**: We prove the feasibility of an advanced beam delivery system for particle therapy based on the integration of fast beam monitors with in-vivo measurements by the upgraded range verification system.

### P 127 - Delivery benefits and dosimetric implications of proton PBS with novel continuous scanning

#### Xiaoying Liang^1^, Chris Beltran^1^, Chunbo Liu^2^, jiajian shen^3^, Heng Li^4^, Keith M. Furutani^1^

##### ^1^Mayo Clinic - Florida, Rad Onc, Jacksonville, USA ^2^The First Affiliated Hospital of Zhengzhou University, Rad Onc, Zhengzhou, China ^3^Mayo Clinic - Arizona, Rad Onc, Phoenix, USA ^4^Johns Hopkins Medicine- Baltimore, Rad Onc, Baltimore, USA

**Purpose**: To investigate delivery benefits and dosimetric implications of dose-driven continuous scanning (DDCS) vs discrete spot scanning (DSS) for PBS systems.

**Methods**: Eight treatment plans in prostate, HN, liver, and lung, with both conventional fractionation and hypofractionation were included in the study. The beam delivery benefit and dose deviation such as gamma analysis were simulated as a function of stop ratio for DDCS. The results were compared with those of DSS.

**Results**: DDCS considerably improved the irradiation duty factor (range, 11%-41%) compared to DSS (range, 4%-14%), within which, hypofractionation schemes had greater improvement than conventional fractionation. With decreasing stop ratio constraints, the DDCS BDT reduction was greater, but dose deviation also increased. With stop ratio constraints of 2, 1, 0.5, and 0, DDCS BDT reduction reached to 6%, 10%, 12%, and 15%, respectively. The 3%/2-mm gamma passing rate was greater than 99% with stop ratio constraints of 2 and 1, and greater than 95% with a stop ratio of 0.5. When the stop ratio constraint was removed, 3 of the 8 treatment plans had a 3%/2-mm gamma passing rate below 95%. The PTV DVH RMSE was within 1%, 2% and 3% for stop ratio of 2, 1 and 0.5, respectively. When there is no stop ratio constraint, the PTV DVH RMSE reached 5%.

**Conclusion**: The irradiation duty factor was considerably improved with DDCS. Smaller stop ratio constraints led to shorter BDTs, but with the cost of larger dose deviations.

### P 128 - Investigation of high-speed and large-field scanning magnets for small gantries

#### Shinji Nomura^1^, Nagaaki Kamiguchi^1^, Takehisa Tsurudome^1^

##### ^1^Sumitomo Heavy Industries- Ltd., Technology Research Center, Yokosuka, Japan

The miniaturization of the gantry is important to make the proton therapy facilities smaller. However, the downsizing of the gantry, i.e., the reduction of SAD (Source to Axis Distance), has a significant influence on the scanning speed and irradiation field. We investigated the scanning magnet with the fastest scanning speed and the largest irradiation field in the industry for a smaller-than-standard gantry. Based on 2D and 3D magnetic field analysis, the magnet geometry, number of coil turns, inductance, and power supply performance were optimized. The benchmark scanning speed and irradiation field were 100 mm/ms and 300 mm × 400 mm, respectively. The upstream and downstream magnets were for scanning in the vertical and horizontal directions, respectively. For the upstream magnet, the magnetomotive force was reduced by increasing the pole length. On the other hand, a large irradiation field was achieved by increasing the magnetomotive force. The downstream magnet was affected by the deflection of the beam in the upstream magnet, resulting in a larger current requirement. This increased the current sweep rate and reduced the scanning speed. It was found that both the irradiation field and the scanning speed can be increased by modifying the shape of the magnet.

### P 129 - Advanced Beam delivery technology for heavy ion radiotherapy system

#### Kazuhito Tomita^1^, Shoichi Yagi^1^, Akihiro Osanai^1^, Atsushi miyamoto^1^, Shinya Matsuda^1^

##### ^1^Toshiba Energy Systems & Solutions Corporation, Particle Radiotherapy Business Project Engineering Dept., Horikawa-cho- Saiwai-ku- Kawasaki, Japan

Toshiba, in collaboration with NIRS/QST, has been developing advanced beam delivery technologies. One of the features of latest Toshiba beam delivery technology is high-speed 3D scanning system which is eliminated the range shifters due to 600 levels of the energy changing. Each slice is scanned at fast speed using a scanning electromagnet with expanding shape like a cone and typical slice switching time (energy changing time) is 200ms/2mm range change at the high energy level. This technology enables efficient and accurate irradiation. Furthermore, in 2016, Toshiba achieved a reduction in size and weight of the rotating gantry for QST compared with the conventional one by applying superconducting electromagnets instead of resistive magnet. Currently, we developed even compact rotating gantry, the length is about 9m, which is equivalent to the rotating gantry applied to proton radiotherapy. While these technologies are contributing to the advanced treatment and the expansion of the scope of application, the equipment and systems are becoming more complicated. Therefore, we are working on shorten the construction and beam adjustment period and save labor by devising the construction method and automating the operation system. These technologies are incorporated in several heavy ion therapy facilities as NIRS/QST, Kanagawa Cancer Center, Yamagata University, and Yonsei University witch the beginning of treatments are scheduled in 2023.

### P 130 - Tungsten carbide as an alternative aperture material to brass in proton beam therapy

#### Alexandre Santos^1^, Scott Penfold^1^

##### ^1^South Australian Health and Medical Research Institute, Australian Bragg Centre for Proton Therapy and Research, Adelaide, Australia

With the widespread utilisation of pencil beam scanning (PBS) techniques, the need for apertures has reduced. However, in a limited number of cases the improved lateral penumbra provided by apertures may be beneficial in providing dose sparing to organs at risk. Traditionally, brass has been utilised for making apertures in proton therapy. However, the cost associated with brass apertures can be considerable. In this study, an alternative material is investigated, that is tungsten carbide. Tungsten carbide apertures have been routinely utilised for electron beam cutouts and can be provided at a fraction of the cost of that of brass. In this work, TOPAS monte-carlo has been utilised to characterize the properties of tungsten carbide and compare those to brass. The key characteristics investigated are the neutron production, scatter effects and activation of the aperture. As expected, tungsten carbide irradiated with 235 MeV protons produces significantly more neutrons than brass. Additionally, due to the activation of longer lived radioisotopes the cooling rate of tungsten carbide is slower than that of brass. These results were simulated with a solid piece of material and hence represent a worst case. In the scenario of PBS where only a thin section of the aperture material will be irradiated, tungsten carbide shows promise to be an alternative to brass.

### P 131 - Development of a 2D-ripple filter for the particle therapy facility MedAustron, optimized for non iso-centric treatment

#### Patrick Karner^1^, Martin Bräuer^2^, Andreas F. Resch^1^, Alessio Elia^1^, Loïc Grevillot^1^, Markus Stock^1,3^

##### ^1^EBG MedAustron GmbH, Medical Physics, Wiener Neustadt, Austria ^2^Siemens Healthcare GmbH, Clinical Application & Physics, Erlangen, Germany ^3^Karl Landsteiner University of Health Sciences, Medical Physics, Wiener Neustadt, Austria

This work describes the process of developing and manufacturing a 2D ripple filter (RiFi) for non-isocentric treatment at MedAustron. The RiFi design is intended to accurately deliver and reduce delivery times of a spread-out Bragg peak (SOBP). This is achieved by broadening the width of the pristine Bragg peak (BP), which leads to a reduction in the number of energy layers. For the development, Gate/Geant4 Monte Carlo depth dose profiles were simulated, where the initial energy spread of the beam model (created with RiFi) was optimized to fit open beam measurements. From these data, a geometry independent RiFi pin-shape was determined that would produce a homogeneous SOBP with a dose deviation of less than 1% using initial carbon beam energies with range steps of 3 mm in water (see Figure 1). The raw pin-shape was corrected with the WET of the 3D-printed material and adapted to the footprint of the element structure size of the RiFi with λ=1 mm (see Figure 2), which is based on the width of the Gaussian angular distribution of the MedAustron carbon beam to guarantee a non-isocentric irradiation 20 mm after the exit window. The 2D-RiFi was manufactured using a PolyJet 3D-printer (Stratasys, USA-Israel), where pyramid shaped pins were rotated 45° to the footprint of the ripple structure size (see Figure 2). This design choice offers the advantage of a self-stabilizing structure, and eliminates the need for a whole manufacturing base-structure for the RiFi, thus avoiding an unnecessary range shift for the modulated beam.

### P 132 - Repurposing a LINAC vault for proton therapy: A shielding review

#### Mark Leuschner^1^, Niek Schreuder^2^

##### ^1^Granite Radiological Consultants- LLC, Shielding Design, Peterborough, USA ^2^LEO Cancer Care, Medical Physics, Knoxville, USA

One of the main hurdles to bring proton therapy to more patients is the total project costs of a typical proton therapy center comprising of the equipment costs, the building costs, working capital and interest. The large sizes of the proton rotating gantries make installing a proton therapy system in an existing radiation therapy building impossible hence disallowing re-using existing shielded vaults and clinical spaces. Leo cancer care developed an upright imaging and patient positioning system that allows for imaging and treating cancer patients in a seated or semi standing position obviating the need for large rotating gantries. Combining the Leo system with a proton beam delivery nozzle constitutes an extremely compact proton therapy solution that easily fits in most existing LINAC rooms. However, a source for the accelerated protons is still needed. This can be achieved in two ways – (1) by installing a very compact proton accelerator and beam delivery nozzle in the same room i.e. the accelerator delivers the protons directly to the beam delivery nozzle or (2) by installing the proton accelerator in another room and bringing the accelerated protons to the beam delivery nozzle via a beam transport line protruding the LINAC bunker from the outside. We examined the shielding requirements for both these scenarios using validated Monte Carlo methods. We will present the minimum shielding barrier thicknesses required for both these scenarios and show that exiting LINAC rooms can easily be modified to accommodate such compact proton systems.

### P 133 - Upgrade programme of the Douglas cyclotron, Clatterbridge Cancer Centre

#### Peter Corlett^1^, Linda Mortimer^1^, Stephen Elmer^1^, John Gordon^2^, Jernej Podobnik^3^

##### ^1^Clatterbridge Cancer Centre, Physics, Bebington, United Kingdom ^2^Pyramid Technical Consultants, European Office, Henfield, United Kingdom ^3^Cosylab, Medical devices, Ljubljana, Slovenia

The Douglas cyclotron was commissioned in 1984 to provide fast neutron therapy for randomised patient trials of radio-resistant tumours of the pelvis and head and neck. Since 1989, a proton therapy beamline has been used for the treatment of cancers of the eye. Up to this point, the cyclotron has been diligently maintained in its original condition, with limited modifications. Due to the extreme age of the cyclotron, many components and sub-systems have exceeded their design life span, and are beginning to fail. Manufacturer support cannot be relied upon as most components are no longer manufactured and are obsolete. In order to extend the u seful life of the facility by 5 to 10 years, a programme of targeted upgrades is underway to replace key components which are deemed to present a risk of failure with modern, supported, state of the art alternatives. These upgrades range from basic engineering – replacing aging water hoses, to large complex upgrades such as complete replacement of the accelerator control system and replacing the dose control system. The current status of the upgrade programme is presented, along with future plans.

### P 134 - Performance, functionality, and future upgrades of the Christie proton research beamline for radiobiology

#### John-William Warmenhoven^1^, Nicholas Henthorn^1^, Samuel Ingram^1^, Samuel Manger^1^, Samuel Burford-Eyre^1^, Jack Aylward^1^, Michael Merchant^1^, Michael Taylor^1^, Ranald Mackay^2^, Karen Kirkby^1^

##### ^1^University of Manchester, Division of Cancer Sciences, Manchester, United Kingdom ^2^The Christie NHS Foundation Trust, Medical Physics and Engineering, Manchester, United Kingdom

The University of Manchester/Christie NHS FT proton research room (Figure 1) has been operational since late 2019 occupying the 4th gantry room of the proton therapy centre and is served by the same accelerator as the clinical rooms. This room offers researchers access to clinically-relevant-energy proton irradiation for in vitro samples and is the only dedicated proton research facility of its kind in the UK. The research room also has a dedicated biology end-station that provides automated irradiation of samples in environmental conditions controlled for temperature, humidity, and gas mixture. The end-station and fully equipped bio-prep room enables us to provide high throughput irradiation of biological samples in ideal conditions for in vitro experiments. Additionally, funding has been secured to expand the capabilities of the room by building a second pre-clinical beamline. This new beamline will be split from the main beamline by a dipole magnet and provide pencil beam scanning of a 1 mm spot over a 30x30 mm area at dose rates exceeding 40 Gy/s, both in transmission and Bragg-peak modes. Here, we provide details of the measured capabilities to highlight what is available to researchers, some examples of experimental achievements, and descriptions of future capabilities.

### P 135 - 3D range-modulators: How to improve the complex geometry implementation in FLUKA with a faster, but simpler, calculation approach

#### Warisara Charuchinda^1,2^, Felix Horst^1^, Yuri Simeonov^3^, Narumon Suwonjandee^2^, Burin Asavapibhop^2,4^, Marco Durante^1^, Uli Weber^1^

##### ^1^GSI Helmholtzzentrum für Schwerionenforschung, Biophysics Division, Darmstadt, Germany ^2^Chulalongkorn University, Department of Physics, Bangkok, Thailand ^3^Institut für Medizinische Physik und Strahlenschutz, Technische Hochschule Mittelhessen, Giessen, Germany ^4^The Southeast Asian Ministers of Education Organization Regional Centre for STEM Education, STEM Resources and Capacity Building, Bangkok, Thailand

The hybrid delivery of raster scanning applied with a passive device called a “3D range-modulator (3DRM)” is one of the promising beam application techniques for FLASH therapy with protons or heavy ions [1]. Because of its compact design as a combination of a range-compensator and a 3D ridge filter, the 3DRMs modality allows a highly conformal dose and modulated Spread-Out-Bragg-Peak to be created in the target volume within milliseconds. Being an established and reliable tool for particle therapy research, the FLUKA Monte Carlo particle transport code has been selected to support the development process of 3DRMs, e.g., investigating and validating the modulating properties of the prototypes and the particle fluence distribution behind them, as well as being utilized in planning and designing the experiments with 3DRMs. Despite the fact that FLUKA is a user-friendly, fully integrated physics package, implementing such complex structures as 3DRMs (figure 1) using only the standard FLUKA combinatorial geometry is not readily possible. Fortunately, FLUKA provides users with an alternative, self-customized user routine called USRMED.f for tasks requiring complex geometries. A new FORTRAN routine within USRMED.f was specifically developed for 2DRMs and 3DRMs. In this presentation, the fast algorithm of the routine will be disclosed and described thoroughly, step by step. This calculation was successfully implemented in FLUKA with the exact results exhibited in figure 2, and could greatly reduce the amount of simulation time.

### P 136 - Monte Carlo simulation model of the SIRMIO beamline and experimental validation in clinical facilities

#### Ze Huang^1^, Jonathan Bortfeldt^1^, Neeraj Kurichiyanil^1,2^, Katrin Schnürle^1^, Franz S. Englbrecht^1^, Thomas Rösch^1^, Sairos Safai^3^, Mateusz Sitarz^4^, Katia Parodi^1^, Marco Pinto^1^

##### ^1^Ludwig-Maximilians-Universität München, Department of Medical Physics Faculty of Physics, Garching b. München, Germany ^2^GSI Helmholtzzentrum für Schwerionenforschung GmbH, Department of Vacuum Systems, Darmstadt, Germany ^3^Paul Scherrer Institute, Center for Proton Therapy, Villigen, Switzerland ^4^Aarhus University Hospital, Danish Center for Particle Therapy, Aarhus, Denmark

The project SIRMIO aims at a portable research system for precision small-animal proton irradiation. This talk will introduce the simulation model for the SIRMIO beamline, consisting of quadrupole triplet as well as energy degradation and collimation systems, and its first experiment validation. The simulation model serves three purposes: 1) better understanding of the system, 2) further testing of new features (eg. FLASH), and 3) providing phase space input for the treatment planning system (TPS). The field maps of the magnetic triplet were created with magnet measurements and Radia software magnet simulation results. The energy degrader used in the beamline was characterized dosimetrically. The beamline model implemented in the Monte Carlo code G4beamline (based on Geant4) was benchmarked against experimental data at two clinical treatment facilities, PSI and DCPT, in various beam energies and beamline set-ups. Fig.1 shows beam profiles measured by an optical-readout Micromegas detector and a gafchromic film in one of the proof-of-concept tests of the SIRMIO beamline, and simulation results under the same condition. The simulation model assisted us to define degrader and collimator settings, predict and understand experimental results, and refine beamline set-up from experimental results. The simulation model also provides phase space data necessary for SIRMIO TPS optimisations. This work is supported by the EU grant 725539 (SIRMIO), and beamtimes were provided within INSPIRE TNA (EU grant 730983) and HITRIplus (EU grant 101008548). We also acknowledge support from the entire SIRMIO team and collaborators.

### P 137 - Patient-specific beam delivery times in a synchrotron-based proton pencil beam (PBS) scanning system

#### Javier Burguete^1^, Pedro Borja Aguilar^2^, Marina García-Cardosa^1^, Juan Diego Azcona^2^

##### ^1^University of Navarra, Physics and Applied Mathematics, Pamplona, Spain ^2^Clínica Universidad of Navarra, Radiofísica y Protección Radiológica, Madrid, Spain

**Purpose**: To characterize the beam delivery times for individual patients in a Hitachi synchrotron-based proton PBS system.

**Materials and Method**: The treatment plan stablishes global requirements for the dose delivery that can be modified on the real device. Our system is a ProBeat-CR (Hitachi) synchrotron that produces beams with 98 different energies, between 70.2 and 228.7 MeV. Values of relevant parameters such as dose rate (MU/s), spot, switch energy and spill change times, are specified by the manufacturer. The irradiation times are obtained using a digital signal from the PBS console that indicates when the beam extraction is on. The signal is recorded using an oscilloscope with a 100MHz filter and a 31kHz sampling rate. The whole treatment plan is recorded, from the start of the first field to the end of the last field. The retrieved data are compared to the treatment plan, to verify the field sequence, and the timing for the different energy levels, spots and spills. The MU rate is reconstructed from the measured spot time compared to the intended treatment time. The number of spots per spill and energy levels per spill are estimated.

**Results**: A preliminary analysis in a group of 10 patients reveals that all the parameters are within the manufacturer specifications. Using these data we can derive patient-specific temporal sequences to achieve time resolved dose calculations (blood circulation, breathing).

**Conclusions**: A tool to retrieve patient-specific beam delivery times is implemented. This tool provides with detailed information useful for algorithms that provide time-solved dose deposition.

### P 139 - Open issues in proton therapy applied to thoracic tumors treatments

#### Carlo Algranati^1^, Lidia Strigari^2^

##### ^1^APSS, Protonterapia, TRENTO, Italy ^2^Istituto di Ricovero e Cura a Carattere Scientifico IRCCS Azienda Ospedaliero-Universitaria di Bologna, Department of Medical Physics, Bologna, Italy

**Objectives**: Report the main recent technological improvements in proton therapy enabling the treatment of thoracic tumors and highlight the current challenges and prospects of these treatments, potentially helpful for improving the outcome and reducing their related toxicity.

**Methods**: A literature search was performed focusing on “proton therapy” AND ("thoracic"; OR “non-small-cell-lung-cancer” OR “small-cell-lung-cancer” OR “mesothelioma” OR “thymoma” OR “thymic malignancy” OR “esophageal cancer”), following the methodology lean to the work of Bogner&Menz on Expert Interviews already adopted in our previous work on imaging in proton therapy for thoracic tumors.

**Results**: Out of the 202 records, 24 were excluded as duplicates. 126 papers of 178 records were selected as inherent to the report’s purpose. Out of the 126 full-text articles assessed for eligibility, 100 full-text articles were grouped into the following categories: Planning, Motion, Delivery, Clinical outcome, Radiobiology, and Economics, and critically discussed according to the primary endpoint open issues and unsolved questions.

**Conclusions**: Most of the fundamental aspects concerning beam technique, planning, and delivery technologies of thoracic tumors are mainly solved, while others related to the recent introduction of modern PT accelerators with online imaging for setup and motion management are “almost solved.” Frontiers’ crucial aspects mainly concern the robustness and trustability of online adaptive, dose accumulation, and radiobiological models for predicting toxicity and secondary tumors based on reported clinical outcomes. In addition, cost-effectiveness evaluations represent open historical questions needing to reach adequate maturity.

### P 140 - Neutron Beam System for Accelerator-Based Boron Neutron Capture Therapy

#### Alex Dunaevsky ^1^

##### ^1^TAE Life Sciences, Beam Technology, Foothill Ranch, USA

Over the last decade, Accelerator-Based Boron Neutron Capture Therapy (AB-BNCT) has enjoyed exponential growth. Among a variety of accelerator systems used for generating neutron beams for AB-BNCT, electrostatic proton accelerators are proven to be simple, reliable, and cost-effective. Electrostatic proton accelerators with proton energies of about 2.3-2.5MeV generate neutrons on Lithium targets with an energy spectrum favorable for efficient moderation to the epithermal energies required for BNCT. The Neutron Beam System (NBS) offered by TAE Life Sciences (TLS) is based on the tandem architecture of electrostatic accelerators. Double action of the accelerating voltage and ion injector at the ground potential are among the technical advantages of the tandem accelerators. TLS NBS based on the tandem accelerator delivers both proton beam energy and proton current on the clinically relevant level. The system can support multiple treatment rooms. The first TLS NBS for clinical use is installed in Xiamen, China, as a part of NeuPex AB-BNCT System designed and developed by Neuboron Medical Group. At the present state, the first treatment room is fully commissioned and undergoing regulatory tests and pre-clinical trials. AlphaBeam, a turn-key AB-BNCT system offered by TLS, is currently on its way to installation in Padova, Italy at CNAO. The presentation provides a technical insight of the TLS NBS, as well as a quick overview of the AlphaBeam system.

### P 141 - Commissioning and quality assurance at East Japan Heavy Ion Center, Faculty of Medicine, Yamagata University

#### Takeo Iwai^1^, Hikaru Souda^1^, Takayuki Kanai^2^, Yuya Miyasaka^1^, Sung Hyun Lee^1^, Hongbo Chai^1^, Masashi Katsumata^3^, Azusa Sato^3^, Hiraku Sato^1^, Kenji Nemoto^1^

##### ^1^Faculty of Medicine- Yamagata University, East Japan Heavy Ion Center, Yamagata, Japan ^2^Tokyo Women’s Medical University, Department of Radiation Oncology, Tokyo, Japan ^3^Accelerator Engineering Corporation, Yamagata Office, Yamagata, Japan

The latest carbon-ion therapy facility in Japan called “East Japan Heavy Ion Center, Faculty of Medicine, Yamagata University” is now under operation. It is equipped with a horizontal port mainly for prostate cancer and a 360-degree rotating gantry port for other types of cancer. Our one outstanding feature is range shifter-less irradiation systems with 0.5 mm range step times 600 energy levels of accelerator output. It is challenging that we have to maintain all beam conditions including beam energy and gantry angle within tolerance levels we had set in our quality assurance program. Clinical commissioning of the horizontal port included installation of beam model to the treatment planning system (RayStation^TM^ 10A) based on the dose profile measurement, verification of volumetric dose distribution created by the TPS, calibration of MU and dosimeters, calibration between CT value and stopping power ratio, RBE intercomparison, baseline acquisition of periodic QA protocols, etc. It took several months and they are successfully done before treatment of prostate cancer. Then we completed clinical commissioning of the rotating gantry port. As the beam is basically kept to be compatible between the horizontal port and the rotating gantry port, some items above do not have to be done repeatedly for the rotating gantry port. However, many additional tasks including dependency on the gantry angles, commissioning for upward beam penetrating the couch top, motion management, etc. were done for the gantry. These clinical commissioning including methods, results and the latest status will be presented.

### P 142 - Monte-Carlo simulation-based patient-specific QA with machine log files for proton pencil beam line scanning therapy

#### Chanil Jeon^1^, Jinhyeop Lee^2^, Jungwook Shin^3^, Wonjoong Cheon^4^, Sunghwan Ahn^1^, Kwanghyun Jo^1^, Youngyih Han^1^

##### ^1^Samsung medical center, Department of radiation oncology, Seoul, Korea Republic of ^2^SAIHST- Sungkyunkwan University- South Korea, Department of Health Sciences and Technology, Seoul, Korea Republic of ^3^National Cancer Institute- National Institute of Health, Division of Cancer Epidemiology, Rockville, USA ^4^National Cancer Center, Proton Therapy Center, Goyang, Korea Republic of

**Purpose**: Intensity-modulated proton therapy (IMPT) is a state-of-the-art technique to deliver conformal doses to tumors while saving critical organs. However, IMPT has uncertainties in its intricate modulations. To guarantee accurate plan delivery to patients, patient-specific quality assurance (PSQA) is necessary. In this study, we developed an independent and accurate PSQA system with Monte-Carlo (MC) simulation utilizing machine log files for proton pencil beam line scanning therapy.

**Methods**: Proton pencil beam nozzle geometry in the MC simulation was constructed using TOPAS 3.5. The irradiated beam properties are acquired from the machine log files recorded from the monitor chamber of the nozzle. The spot positions and MU-related values in the logfile were converted to MC simulation parameters such as dipole magnet strength and proton count, respectively. The PSQA system automatically generated TOPAS simulation code with the converted information. The accuracy of the computed MC dose distribution was compared with the two-dimensional absolute dose distribution of the treatment planning system (TPS) and ionization chamber measurement for 56 clinical cases using gamma evaluation with a 2%/2mm criterion.

**Results**: In the gamma evaluation for absolute dose comparison, the average and standard deviation of passing rate for both the TPS and ionization chamber were above 96.5% and below 4%, respectively.

**Conclusion**: We developed a stable PSQA system for proton pencil beam line scanning and confirmed that the system’s accuracy is within a clinically acceptable range. We will expand our system’s functionality for FLASH radiotherapy. Acknowledgements: This work was supported by National Research Foundation of Korea grant funded by Korean government(MSIT). (No. 2021ME2E8A1048108)

### P 143 - Deep learning proton beam range estimation model for quality assurance based on two-dimensional scintillated light distributions in simulations

#### Youngmoon Goh^1^, Eunho Lee^2^, Byungchul Cho^1^, Jungwon Kwak^1^, Ui-seob Lee^1^, Si Yeol Song^1^, Jinhong Jung^1^

##### ^1^Ansa Medical Center, Department of Radiation Oncology, Seoul, Korea Republic of ^2^Yonsei Cancer Center, Department of Radiation Oncology, Seoul, Korea Republic of

**Purpose**: A deep learning method utilized to QA was used to predict the beam range and spread-out Bragg peak (SOBP) for two-dimensional (2D) map conversion from the scintillation light distribution (LD) into the dose distribution in a water phantom.

**Methods**: The 2D residual U-net modeling for deep learning was used to predict the 2D water dose map from a 2D scintillation LD map. Monte Carlo simulations for dataset preparation were performed with varying monoenergetic proton beam energies, field sizes, and beam axis shifts. The LD was reconstructed using photons backpropagated from the aperture as a virtual lens. The SOBP samples were constructed based on monoenergetic dose distributions. The training set, including the validation set, consisted of 8659 image pairs of LD and water dose maps. After training, dose map prediction was performed using a 300 image pair test set generated under random conditions. The pairs of simulated and predicted dose maps were analyzed by Bragg peak fitting and gamma index maps to evaluate the model prediction.

**Result**: The estimated beam range and SOBP width resolutions under varying beam conditions were 0.02 mm and 0.19 mm with deviations less than 0.1 mm and 0.8 mm respectively. The simulated and predicted distributions showed good agreement in the gamma analysis, except for rare cases with failed gamma indices in the proximal and field-marginal regions.

**Conclusion**: The deep learning conversion method using scintillation LDs in an optical camera system with a scintillator is feasible for estimating proton beam range and SOBP width with high accuracy.

### P 144 - Experimental validation of a gamma ray detection module for neutron capture enhanced particle therapy

#### Anita Caracciolo^1,2^, Marissa Kielly^3,4^, Andrew Chacon^4^, Davide Di Vita^1,2^, Marco Carminati^1,2^, Mitra Safavi-Naeini^4^, Carlo Fiorini^1,2^

##### ^1^Politecnico di Milano, Department of Electronics- Information and Bioengineering, Milan, Italy ^2^Istituto Nazionale di Fisica Nucleare INFN, Sezione di Milano, Milan, Italy ^3^University of Wollongong, Centre for Medical Radiation Physics, Wollongong, Australia ^4^ANSTO, Australian Nuclear Science and Technology Organization, Sydney, Australia

We present the experimental validation of a gamma ray detection module for Neutron Capture Enhanced Particle Therapy. NCEPT is an innovative radiotherapy technique that aims to increase the effectiveness of particle therapy with the targeting capability of boron neutron capture therapy (BNCT). As in BNCT, tumor cells are loaded with ^10^B compounds, but in NCEPT the irradiation is performed with protons or heavier ions, and the neutrons required for the ^10^B capture reactions are internally generated in the patient. The detection of gamma rays at 478 keV emitted by the boron neutron capture reactions can be used to real-time monitor the treatment accuracy, and in particular to assess the dose increase due to ^10^B(n, α)^7^Li reactions. We report here the performance of the BeNEdiCTE (Boron Neutron CapTurE) module, a gamma ray detector based on a 2 inches cylindrical LaBr3(Ce+Sr) scintillator crystal, optically coupled to a matrix of Silicon Photomultipliers (SiPMs). A phantom of PMMA material loaded with 5 borated plates (25x25x2 mm^3^ each) and 1 borated cube (1 cm^3^) was irradiated with helium and carbon ions at the HIMAC facility (Chiba, Japan), with a radiation dose rate of 0.033 Gy/min (Figure 1). The same measurements were repeated irradiating the phantom without the boron components, to estimate the difference in the 478 keV gamma ray emission. Some boron is present in the electronic material of the detector. Differences of around 37% and 45% have been found for helium and carbon ions irradiation respectively.

### P 145 - Determining the optimum measurement and analysis parameters for proton patient specific QA

#### Zubin Master^1^, Andrew Wibawa^1^, Calvin Wei Yang Koh^1^, Hong Qi Tan^1^, Kah Seng Lew^1^, Sung Yong Park^1^

##### ^1^National Cancer Centre Singapore, Department of Radiation Oncology, Singapore, Singapore

**Purpose**: Patient specific QA (PSQA) in proton therapy is a time-consuming task. Most centers use a 2D detector array to measure 1-3 dose planes, either within the target volume or upstream in the entrance region of the beam and compare the measured planar dose with the calculated dose from the TPS using gamma analysis. The aim off this study is to determine the optimum number and location(s) of measurement planes, and analysis criteria required to effectively detect errors in the proton plan delivery.

**Methods**: Five different types of plans were selected for this study. Errors of various amplitudes in the spot position and MU, mimicking the delivery error patterns of our proton system, were introduced into the proton plans to create ‘error plans’. The error plans were irradiated, and the measurement depths selected for PSQA were a single fixed depth at 2cm for all the plans, and 3 equally spaced depths within the SOBP formed by each beam (see figure 1). PSQA analysis was performed to determine how many measurement planes, at which depths and using what analysis criteria, were optimum for detecting errors in the plan delivery.

**Results and Conclusions**: One to two measurement planes are enough to detect delivery errors and the most suited depths are either in the proximal region of the SOBP or the entrance region of the beam path. Also, performing PSQA analysis with a 3mm/3% analysis criterion may not be sensitive enough to detect errors in proton beam delivery.

### P 146 - Development of fast and anti-recombination dose monitor for fast scan and FLASH

#### Nagaaki Kamiguchi^1^, Shu Nakajima^2^, Daizo Amano^2^, Takuya Miyashita^2^, Masanori Tachibana^2^, Takehisa Tsurudome^1^

##### ^1^Sumitomo Heavy Industries- Ltd., Technology Research Center, Yokosuka, Japan ^2^Sumitomo Heavy Industries- Ltd., Industrial Equipment Division, Tokyo, Japan

Sumitomo Heavy Industries, Ltd. has been developing fast irradiation technique. To perform fast irradiation, fast dose monitor systems are important. Our dose monitor systems have ever employed a kind of ionization chambers. When considering fast scan speed like 100 mm/ms, it is desirable that response time is less than 50 μs. In addition, consideration about recombination effect is significant for FLASH. The response time and recombination effect are determined from gas species, load voltage and electrode gap. To improve both parameters, reducing the electrode gap is easy way and multiple layer gap design can keep signal level. At this time, the monitor was designed as 2 kV was loaded to 2 mm gap and double sensitive gaps. In this configuration, response time showed 14.3 μs and 2% recombination effect corresponded to about 820 nA in case of 3 mm 1σ beam size. The performance test was conducted with actual beam test. The monitor system was connected to op-amp but the range of low beam current required large amplification gain so that time constant of amplifier circuit became dominant. As actual results, response time were 49.4 μs at high gain and 18 μs at low gain. Signal linearity was confirmed till 400 nA and recombination effect was less than 1%. In presenting development, the monitors for high current and fast scan were developed. By optimizing electrode gap and load voltage, response time achieved less than 50 μs from conventional dose rate to FLASH dose rate by single monitor.

### P 147 - Proton beam spot size and position measurement using a multi- strip ionization chamber

#### Ye Lin^1^, Shuaizhe Gu^2^, Liren Shen^3^, Ming Lv^1^, Qi Liu^2^, Manzhou Zhang^1^, Zhiling Chen^1^

##### ^1^Shanghai Advanced Research Institute- Chinese Academy of Sciences, Department of Particle Beam Applications, Shanghai, China ^2^Shanghai Institute of Applied Physics- Chinese Academy of Sciences, Department of Accelerator Applications, Shanghai, China ^3^Shanghai Advanced Research Institute- Chinese Academy of Sciences, Department of General Technology, Shanghai, China

Commissioning and quality assurance(QA) of pencil- beam scanning proton therapy system involves intensive measurements to calibrate the system and to have a comprehensive understanding of beam spot characteristics. A multi- strip ionization chamber is especially customized as a durable and fast instrument for the measurement of proton beam spot size and position, with a sensitive area of 420 mm*320 mm which covers the field size of the snout. In this study we investigate the feasibility of using a multi- strip ionization chamber to measure the proton beam spot size and position. The responses of the ionization chamber and Gafchromic EBT3 film to therapeutic proton beam were tested in air with various energies range from 73.5 MeV to 235 MeV. The beam spot size results agreed within 0.26 mm and beam spot position results agreed within 0.19 mm for all the measurements. We conclude that the instrument is a simple and practical tool for fast measurement of beam spot size and position.

### P 148 - Is the spot position tolerance set by proton therapy vendor adequate for a clinical plan?

#### Kah Seng Lew^1^, Hong Qi Tan^1^, Wei Yang- Calvin Koh^1^, Andrew Wibawa^1^, Zubin Master^1^, Sung Yong Park^1^

##### ^1^National Cancer Centre Singapore, Division of Radiation Oncology, Singapore, Singapore

Hitachi Probeat proton therapy system has an in-built spot position monitor(SPM) with a set tolerance of 1mm by Hitachi. During treatment delivery, only deviation beyond tolerance either in X or Y-direction will result in interlock. This study aims to investigate the impact of this deviation in actual clinical plan by simulating the worst-case scenario deviation through RayStation(RaySearch Laboratories, Stockholm, Sweden) treatment planning system(TPS). One clinical plan each from head and neck(H&N), breast and prostate is edited using scripting function within RayStation TPS. Each energy layer is shifted randomly in +/− X or Y-direction based on calculation of 1mm projection from SPM to isocenter. Dose is recalculated and repeated 5 times. Following clinical constraints from our centre shown in the table, changes in PTVs and OARs are reported. Comparing D98%, H&N is most robust to shifts with a median dose change of 0.1±0.2%. For Dmax and Dmin, breast is most robust to shifts with a median dose change of −0.05±0.9% and 0.2±1%. For OARs, breast is most robust when comparing across all DVHs with a median dose change of 0.9±0.5%, 0±0.5% and 1.3±0.5% for heart V1.5Gy, V7Gy and lung V8Gy respectively. Among the three, breast is least affected by shifts and this could be due to having the largest ratio between the shift and target volume. In conclusion, 1mm tolerance in Hitachi’s SPM is sufficient for large volumes but not for smaller ones. Attention has to be given as shifts translated to isocenter becomes larger especially for the scanning magnet that is further away.

### P 149 - Applications for a 2D scintillator for ion beam therapy

#### Yanyan Wen^1^, Nicki Schlegel^1^, Michael Moyers^1^

##### ^1^SPHIC, Medical Physics, Pudong, China

The limited availability of silver halide film during the last few years has made quality assurance (QA) and research and development (R&D) difficult. A two-dimensional scintillator (2DS) with reflecting mirror and CMOS detector was acquired and put into clinical service to replace film for some applications. The usable field size of the scintillator plate is 320 mm × 320 mm using 1200 × 1200 camera pixels yielding a pixel size of 0.267 mm square. A lightweight frame was built to allow one person to easily lift and install the 2DS on patient positioners for use with either horizontal or 45^°^ stationary beamlines. The frame allows the 2DS to be aligned by the same x ray localization system used for daily alignment of patients. Thus far, the primary QA tests performed with the scintillator include weekly spot size, position, and shape for proton and carbon ions and weekly range uniformity for carbon ions. For the spot QA, an integration mode is used for a single exposure involving multiple spot energies. For the range uniformity QA over a large field size, multiple frames are acquired as the spot scans across the field. The beam times for the proton spot, carbon ion spot, and carbon range uniformity tests are typically 36, 48, and 24 s respectively. Due to the sub-millimeter pixel size, the 2DS has also been used for an R&D project that requires measurements of lateral penumbra widths for eye treatments. Example data will be presented.

### P 150 - Verification of radiation isocenter of proton therapy beam using a plastic scintillator and a Raspberry Pi camera system

#### Ji Hye Han^1^, Kwanghyun JO^2^

##### ^1^Ewha Womans University, Department of Physics, Seoul, Korea Republic of ^2^Samsung Medical Center, Department of Radiation Oncology, Seoul, Korea Republic of

The measurement of the radiation isocenter is one of the important quality assurance items to guarantee the delivery accuracy of the treatment beam. In this study, a radiation isocenter verification system was developed using a plastic scintillator (Pi- 200, Mitsubishi Chemical Corporation, Tokyo, Japan, 300 × 300 × 0.9 mm^3^) and a Raspberry Pi (Raspberry Pi 4, Raspberry Pi Foundation, London, UK) system. The camera was a Raspberry pi camera V2 with a Sony IMX219 8-megapixel CMOS sensor. The scintillator and Raspberry Pi system with a camera were mounted on an in-house acrylic phantom. An automated procedure for star-shot analysis was presented and implemented as a Python-based GUI program (Tkinter). The procedure was as follows: first, the camera recorded video in avi format with 1640 × 1232 resolution and 30 frames per second. Second, still-shots were extracted from the images with the FFmpeg tool to obtain scintillation distribution images. The star-shot image was then analyzed using the open-source Python library Pylinac to evaluate both the calculated minimum circle radius and the radiation isocenter position. To validate the system, both the Gafchromic EBT3 film (Ashland, NJ, USA) and the system were measured. The minimum size of the radiation isocenter measured by EBT3 film was 0.09 mm and 0.058 mm by our system. The developed program calculates the radiation isocenter size with sufficient accuracy and provides a fast, convenient, and automated star-shot method for clinical proton therapy beams.

### P 151 - Development of multi-slit prompt-gamma camera for accurate proton beam range measurement

#### Youngmo Ku^1^, Chan Hyeong Kim^1^, Sehoon Choi^1^, Jaeho Cho^1^, Sehyun Jang^1^, Jong Hwi Jeong^2^, Sung Hun Kim^2^, Sungkoo Cho^3^

##### ^1^Hanyang University, Department of Nuclear Engineering, Seoul, Korea Republic of ^2^National Cancer Center, Proton Therapy Center, Goyang-si, Korea Republic of ^3^Samsung Medical Center, Department of Radiation Oncology, Seoul, Korea Republic of

Reducing range uncertainty in proton therapy can lead to a decrease in safety margin, thereby lowering healthy tissue dose and normal tissue complication probability. To reduce proton beam range uncertainty, a prompt gamma imaging-based in vivo range verification system, named multi-slit prompt-gamma camera, was developed in the present study. The developed camera consists of a parallel-slit tungsten collimator, a CsI(Tl) detector array, and a signal processing system (Figure 1). A multi-channel high-speed data acquisition system was also developed based on field-programmable gate arrays and analog-to-digital converters. In addition, a 6D automatic camera positioning trolley was developed for high-speed arrangement of the camera in treatment room. The performance of the camera was evaluated by measuring spot beam ranges in a fraction (1 Gy) of spot scanning treatment for a spherical target (diameter=5 cm, center depth=10 cm) in a PMMA phantom at National Cancer Center in Korea. To observe range measurement accuracy, global undershoot scenarios were introduced by placing a PMMA plate of five different thicknesses (0–10 mm). Figure 2 shows the difference between the measured and planned ranges of spots. The magnitude of undershoot was clearly observed for all spots on each energy layer (Figure 2a). The mean differences were in good agreement with introduced undershoots (< 0.3 mm error), and the precision in measurement was 0.6–1.1 mm, which is only ∼1% or less of the beam range (Figure 2b).

### P 152 - Quality Assurance process of a technical upgrade of a patient safety system

#### Pablo Fernandez Carmona^1^, Michael Eichin^1^

##### ^1^Paul Scherrer Institut, Center for Proton Therapy, Villigen, Switzerland

At the center for proton therapy at the Paul Scherrer Instute in Switzerland, we treat cancer patients with a cyclotron, two gantries and a fix beam line. In order to guarantee safe treatments there is a hardware Patient Safety System in place. This system, after 15 years in operation, had two problems that needed preventive action: 1) age of components and the corresponding obsolescence and lack of new spare parts, and 2) a bug that caused unnecessary emergency switch offs of the cyclotron, causing an accumulated damage and the need for extra maintenance and downtime to the patient program. The work presented here focuses on the two iterations of the plan-do-check-act cycle involved in the technical upgrade from a quality assurance perspective, which lasted four years. The first iteration was the upgrade of the safety system itself: identifying the requirements, planning the implementation and tests, assuring the performance and safety of the new system and its clinical commissioning. The second iteration involved the adaptation of user manuals, technical and safety documentation, personnel training, regular quality control tests and communication with the regulatory authorities. We seized the upgrade opportunity to review our QA manual for changing the periodicity, improving, removing, combining or introducing new tests to control our set-up as needed. In sum, we share our experience on the multiple aspects involved in a safety critical technical upgrade and how we consequently made sure that the documentation reflects the way we work, and is lived and not only used during commissioning.

### P 153 - Assessment of dosimetric characteristics leading to replanning of head and neck cancer patients in a clinical setting

#### Ihsan Bahij^1^, Ihsan Bahij^2^, Nadine Heidrun Vatterodt^3^, Nadine Heidrun Vatterodt^4^, Ulrik Vendelev Elstroem^3^, Stine Sofia Korreman^5^, Stine Sofia Korreman^6^, Stine Sofia Korreman^3^

##### ^1^Aarhus University Hospital, Danish Centre for Particle Therapy, Aarhus N, Denmark ^2^Aarhus University, Deparment of Health, Aarhus, Denmark ^3^Aarhus University Hospital, Danish Centre for Particle Therapy, Aarhus, Denmark ^4^Aarhus University, Department of Health, Aarhus, Denmark ^5^Aarhus University, Deparment of Clinical Medicine, Aarhus, Denmark ^6^Aarhus University Hospital, Department of Oncology, Aarhus N, Denmark

**Purpose**: We aimed to investigate dosimetric differences between patients for whom replanning was done compared to patients with no replanning, for the cohort of head and neck cancer (HNC) patients treated at our clinic.

**Materials and Methods**: This retrospective study included all HNC patients treated at one proton therapy center over a two-year period. The patients were divided into non-replanned (group1) and replanned (group2). In the clinic, assessment of need for replanning was based on weekly control CT (cCT) in which the original treatment plan was recalculated. We quantified the volumes receiving less than 95% of prescription dose within the CTVs (CTV1=68/66Gy, CTV2=60Gy, CTV3=50Gy), and minimum doses in these volumes recalculated in cCTs corresponding to replan decision time point. For group1 we evaluated on cCT from week 4. For patients who didn’t have an underdosed volume, we quantified the minimum dose in the whole CTV. Statistical significance was assessed using Wilcoxon Rank Sum Test.

**Results**: Fifty-eight (58) out of 87 patients had at least 1 replan; 40 were due to expected lack of dose coverage. The analysis showed no significant difference in minimum-dose for intermediate-risk CTV, but significant differences for low- and high-risk CTVs in favor of group1 (fig.1). Group1 showed significantly lower under-dosed volumes than group2. There was some overlap in the distributions of minimum-doses and underdosed volumes between group1 and group2 (fig.2).

**Conclusion**: Patients for whom replanning was performed in our clinic generally had larger underdosed volumes and lower minimum-doses than patients for whom no replanning was performed.

### P 154 - An analytical approach to predict 3D positron emitter distributions in carbon ion therapy

#### Tianxue Du^1^, Katia Parodi^1^, Marco Pinto^1^

##### ^1^Ludwig-Maximilians-Universität München, Department of Medical Physics, Munich, Germany

Carbon ion therapy offers ballistic precision but is more sensitive to range uncertainties than photon therapy. Therefore, range verification is important in quality assurance, and positron emission tomography (PET) is the most commonly used approach for this purpose. Since the PET signal isn’t directly proportional to the dose distribution, a comparison to a predicted positron emitter distribution (PED) is typically required for range verification. Parodi and Bortfeld [1] proposed an analytical method to predict PED from depth-dose distributions on proton therapy and Hofmann et al. [2] extended it to carbon ion therapy. However, they only investigated it for 1D cases and quasi-3D cases, which showed limitations in dealing with heterogeneities and integration with a planning system. Hence, their work was revised and improved, especially when handling heterogeneities and the positron emitters considered. A dedicated framework based on a pencil beam algorithm (PBA) was developed to construct 3D PEDs. The PBA includes the ray-casting model proposed by Schaffner et al [3] to properly model lateral inhomogeneities. The Geant4 Monte Carlo tool was used to build the dataset for this approach, and also for validation in in-silico studies using different homogeneous and inhomogeneous phantoms, and clinical data. The details about this approach will be disclosed and results from its validation will be presented. Tianxue Du acknowledges financial support from CSC.

### P 155 - Dose correction methods of EBT3 radiochromic film irradiated by mixed-LET carbon-ion beams

#### Xinguo Liu ^1^

##### ^1^Institute of Modern Physics- Chinese Academy of Sciences, Medical Physics Department- Biomedical Research Center, Lanzhou City, China

**Purpose**: To correct the doses measured by EBT3 radiochrmomic films irradiated by mixed LET carbon-ion beams, two dose correction methods were compared.

**Methods**: Carbon-ion beam of 260 MeV/u was used to obtain carbon-ion beams of six different dose-averaged LETs with energy degraders. Some films were irradiated by these carbon-ion beams respectively and a fitting formula was selected to fit the film dose response calibration curves. Another films were irradiated by two out of the six carbon-ion beams mixed with different proportions and the film dose response calibration curves were obtained with the same fitting formula. Relative Efficiency (RE) was used to quantify the under-response of EBT3 film with LET, and the doses of the films irradiated by mixed-LET carbon ions were corrected by the RE method. In addition, according to the relation between the fitting parameters of the dose response calibration curve and the dose proportions of different LETs, a new dose correction method, named fitting parameter method, was proposed and the doses of the films irradiated by mixed-LET irradiation were also corrected.

**Results**: The correction results of the two methods were compared with the irradiated doses, and it was shown that the dose deviations obtained by the fitting parameter method were less than 5%, which is better than the dose deviations obtained by the RE method less than 10%.

**Conclusion**: The fitting parameter method is a potential dose correction method for EBT3 films irradiated with mixed-LET carbon ions. However, the accuracy still needs to be improved further.

### P 156 - 3D-printed materials for end-to-end test phantoms in particle therapy

#### Jacob Brunner^1,2^, Markus Stock^2,3^, Barbara Knäusl^1,2^, Dietmar Georg^1^

##### ^1^Medical University Vienna, Department of Radiation Oncology, Vienna, Austria ^2^MedAustron Ion Therapy Center, Medical Physics, Wiener Neustadt, Austria ^3^Karl Landsteiner University of Health Sciences, Medical Physics, Wiener Neustadt, Austria

**Purpose/Objective**: End-to-end (E2E) tests in radiation oncology aim to mimic a complete patient workflow by employing a phantom suitable for imaging and dosimetry. This work aims to characterize 3D-printed and epoxy resin (ER)-based materials for manufacturing such a phantom for particle therapy with accurate CT numbers (CTN) and stopping power ratios (SPR).

**Material and Methods**: The measured SPR_meas_ of nine 3D-printed samples (5x5 cm^2^, thickness t = 1 cm) was determined in a 148.2 MeV proton beam at three different sample points (Table 1). CTN and predicted SPR_pred_ were retrieved from a CT (kVp = 120 V, slice thickness = 2 mm) scan of the CIRS CT calibration phantom equipped with 3D-printed plugs. Additionally, four ER-based samples made of 50% ER and different ratios of bone meal (BM) and polyethylene powder (PE) (1:0, 4:1, 1:1, 1:4 ratios BM:PE) were investigated.

**Results**: SPR_meas_ ranged from 0.91 to 1.52 with a measurement uncertainty ≤1% (Table 1). For six materials a difference <3% between SPR_pred_ and SPR_meas_ was found (Figure 1): NY-12, ABS, NY-FDM, PLA and two resin-based casts (1:0 BM:ER, 4:1 BM:ER). Due to trapped air resulting in a standard deviation of SPR_pred_ **≈**10%, 1:1 and 1:4 BM:PE samples were not considered.

**Conclusion**: Six tissue surrogates have been identified: ABS for soft tissue, Nylon-12 for breast/fatty tissue, PLA/FDM-Nylon for skin/cartilage and ER-based mixtures for low and medium density bone. Exploratory measurements with carbon ions confirmed the proton results. The identified materials will be used to produce E2E test phantom prototypes mimicking patient weight loss with various inserts for dosimetry.

### P 157 - A streamlined method for checking the output for all the beam energies available in a proton therapy synchrotron

#### Juan Diego Azcona Armendáriz^1^, Maria Fernandez Ramos^1^, Diego Pedrero^1^, Borja Aguilar^1^, Miguel Garcia Cutillas^1^, Roser Fayos-Sola^1^, Elena Antolin^1^, Jose Miguel Delgado^1^, Alberto Viñals^1^, Pablo Cabello^1^

##### ^1^Clinica Universidad de Navarra, Radiofisica y Proteccion Radiologica, Madrid, Spain

**Purpose**: To streamline the monthly quality assurance of a proton beam therapy system enabling a fast and reliable check of the output for all the beam energies available.

**Materials and Method**: Our system is model ProBeat-CR (Hitachi). The synchrotron produces pencil beams with 98 energies, between 70.2 and 228.7 MeV. A Bragg peak chamber, PTW Freiburg model 34070 was positioned with its surface at isocenter, at 2-cm depth on an acrylic phantom. The trigger in the water tank PTW MP3-PL activates the detector to measure when receiving the signal from the synchrotron at the extraction of a new spill. This measurement was combined with specific system “physics files” with a list of control points. A spill was used for every measurement of 10 monitor units delivered by a single spot to isocenter.

**Results**: The time needed to irradiate and collect the charge for all energies is about 10 minutes. The readings are corrected by pressure and temperature to relate to a “reference condition”. To demosntrate the method, we have measured the output three times (first as reference). The first comparison shows differences among all energies between 0.03 and −0.15%, with a mean difference of −0.06%. The second comparison showed differences between 0.93 and 0.61%, mean of 0.80%. This is consistent with and complements the results of TRS-398 SOBP checks using a subset of energies and a Farmer chamber.

**Conclusions**: A streamlined quality assurance procedure was developed which promises to be fast and accurate for performing monthly checks of the system output.

### P 158 - Measuring kV field size using a scintillator on proton beam therapy gantries

#### John Wong^1^, Alexander Grimwood^1^

##### ^1^University College London Hospitals NHS Foundation Trust, Radiotherapy Physics Department, London, United Kingdom

**Purpose**: IPEM Report 91 recommends any side of a kV imaging field should not exceed the detector field of view by > 1 cm when the source-to-detector distance (SDD) is 1 m. Field size measurements commonly use radiochromic film, but the 2.7 m SDD on UCLH ProBeam gantries renders this method insensitive. Our team devised an alternative measurement method using a Logos XRV-4000 scintillator.

**Method**: The XRV-4000 was aligned to the isocentre, and kV imaging was delivered with the source at 0°. Our team developed two image processing methods to define the kV field edges. Firstly, the gradient method found the second derivative of the image intensity profile and defined the kV edges by the largest gradient change. Secondly, the threshold method used Otsu’s Method to create a binary threshold mask and defined the kV edges accordingly. Three acquisitions were made for each measurement across all four gantries on both sources (A & B). Five measurements were also taken on Gantry 4 across different days to test consistency.

**Results**: Both methods produced consistent results on Gantry 4 with ±1 mm variability (Table 1). Other gantries produced similar agreements. Measurements on Gantry 4 across different days also demonstrated a similar consistency of ±1 mm. The threshold method on Gantry 4 (Source A) showed the A-side field edge exceeded a notional tolerance of +27.0 mm by 1.8 mm (Figure 1), which was small compared to the field’s penumbra.

**Conclusion**: The methodology described is a viable method to measure kV field size with a variability of ±1 mm.

### P 159 - Range verification for rapid QA using a scintillator and wedge

#### Alexander Grimwood^1^, Allison Toltz^1^, John Wong^1^, Alison Warry^1^

##### ^1^University College London Hospitals NHS Foundation Trust, Medical Physics, London, United Kingdom

**Introduction**: Proton range verification checks are integral to every treatment centre’s quality assurance programme. Scintillation-based devices are fast, lightweight and robust systems offering potential advantages over other range verification methods (i.e. water tanks or multi-leaf ionisation chambers). This study evaluated range measurements using a commercial scintillation device (Logos XRV-4000 Hawk) with a wedge-shaped buildup designed to capture Bragg Peaks across a range of depths (Logos LCW-300).

**Method**: Water equivalent proton range measurements were recorded for energies between 200 MeV – 100 MeV across four gantries at a Varian ProBeam centre. Results were compared to our treatment planning system’s beam model (TPS) and NIST reference data. Sensitivity to energy changes ≤1 MeV was quantified and an inter-gantry comparison was also conducted. Beam spot and Hawk acquisition parameters were optimised to maximise scintillation image quality whilst reducing irradiation dose and time.

**Results**: Range measurements were consistent, giving overall mean (SD) agreements of 0.0 (0.2) mm for NIST and 0.2 (0.3) mm for TPS (Figure 1). Sensitivity to systematic shifts in energy ≤1 MeV is illustrated in Figure 2 for a single gantry. Optimised spot spacing of 5 mm and weighting of 4 MU produced an acquisition time of 30 s for five energies for Hawk camera parameters of 4 frames/s and 10 dB gain.

**Conclusion**: Energy Range verification using a commercial scintillator and buildup wedge enabled consistent, sensitive and fast measurements with <0.5 mm uncertainty, making it well-suited to routine QA.

### P 160 - A fast quality assurance method for carbon ion radiotherapy using deep learning-based Monte Carlo denoising

#### Xinyang Zhang^1,2,3,4^, Hui Zhang^1,2,3,4^, Pengbo He^1,2,3,4^, Qiang Li^1,2,3,4^

##### ^1^Institute of Modern Physics- Chinese Academy of Sciences, Medical Physics, Lanzhou, China ^2^University of Chinese Academy of Sciences, Medical Physics, Beijing, China ^3^Key Laboratory of Basic Research on Heavy Ion Radiation Application in Medicine, Medical Physics, Lanzhou, China ^4^Key Laboratory of Heavy Ion Radiation Biology and Medicine of Chinese Academy of Sciences, Medical Physics, Lanzhou, China

**Purpose**: To evaluate the feasibility of fast quality assurance (QA) method for carbon ion radiotherapy using deep learning-based Monte Carlo (MC) denoising.

**Methods**: To accelerate the dose calculation, a CycleGAN model based on deep learning with the U-net model as generator was developed. The study was conducted on 30 head-and-neck cancer patients. The input MC dose distribution was derived from 1 × 10^6^ simulated carbon ions while the reference came from 1 × 10^8^. The root mean squared error (RMSE) and peak signal-to-noise ratio (PSNR) were computed to evaluate the similarity between the predicted and reference dose distributions. Three-dimensional gamma analyses (3mm, 3%) was also performed.

**Results**: After training, our model successfully denoised new MC dose maps. For the denoising approach, we found a significant improvement in the dose volume histogram (DVH) for the predicted images compared with the input images. The RMSE and PSNR for the predicted versus reference images were significantly better than those of the input versus reference images. Moreover, the average gamma pass-rates were >97.62% for the predicted versus reference images and the inference time of our model for a dose distribution was less than 10 s vs 20 hours (MC simulation using 1 × 10^8^ particles).

**Conclusion**: We propose an end-to-end deep network that can denoise Monte Carlo dose distributions. The network provides comparable qualitative and quantitative results as the MC dose distribution simulated with 1 × 10^8^ particles, offering a significant reduction in computation time.

### P 161 - A new audit tool for proton therapy: Proton head and neck evaluation (Prudence) phantom

#### Hannah Cook^1,2^, Nathan Niemann^3^, Callum Gillies^4^, Vasilis Rompokos^4^, Matthew Lowe^5^, Mohammad Hussein^1,2^, Catharine Clark^1,2,6,7^, Russell Thomas^2^, Andy Nisbet^1^, Gary Royle^1^, Hugo Palmans^2,8^, Ana Lourenço^1,2^

##### ^1^University College London, Medical Physics and Biomedical Engineering, London, United Kingdom ^2^National Physical Laboratory, Medical Radiation Science, Teddington, United Kingdom ^3^Barts Health NHS Trust, Clinical Physics Department, London, United Kingdom ^4^University College Hospital NHS Foundation Trust- Proton Beam Therapy Centre, Medical Physics Department, London, United Kingdom ^5^The Christie NHS Foundation Trust- Proton Beam Therapy Centre, Christie Medical Physics & Engineering, Manchester, United Kingdom ^6^Radiotherapy Trials Quality Assurance group RTTQA, Mount Vernon Cancer Centre, Northwood, United Kingdom ^7^University College Hospital NHS Foundation Trust, Radiotherapy Department, London, United Kingdom ^8^MedAustron Ion Therapy Centre, Medical Physics Group, Wiener Neustadt, Austria

Proton therapy has been proposed for the treatment of oropharyngeal cancer and the site is currently subject to a UK clinical trial (TORPEdO). This site can be challenging to treat due to a complex tumour target, numerous nearby organs at risk and a varying patient shape. Anatomical changes, such as weight loss, requiring treatment replanning are also common through the course of treatment. For such complex treatments, quality assurance measures such as end-to-end audits are essential to ensure quality and best practice for clinical trials and radiotherapy treatments. A phantom was designed to perform an end-to-end audit of the proton head and neck treatment process, including imaging, dose calculation, and treatment delivery. The phantom was manufactured from novel proton optimised tissue-equivalent materials and designed to use ionisation chambers, alanine pellets and radiochromic film to provide absolute and relative dose measurements within the phantom. A prototype weight-loss feature was also developed to assess the effects of weight change on patient plans. The phantom was tested as an end-to-end audit device at the two UK NHS high energy scanning proton centres. The study showed agreement between ionisation chamber and alanine to treatment planning calculations within 2% in the tumour region and film analysis showing >95% pass rate for 4%/3 mm global gamma analysis for both centres. The phantom was shown to be a useful tool to evaluate proton therapy deliveries and provides a realistic challenge for clinical centres as part of an end-to-end audit service.

### P 162 - Compact detector for coaxial monitoring of prompt gamma-rays for proton therapy range verification

#### Iris García Rivas^1^, Antonio Fernández Prieto^1^, Fernando Hueso-González^2^, Gabriela Llosá^2^, Pablo Vázquez Regueiro^1^

##### ^1^Galician Institute of High Energy Physics - University of Santiago de Compostela, Particle Physics, Santiago de Compostela, Spain ^2^Instituto de Física Corpuscular IFIC- CSIC-UV, Medical Physics Group, Valencia, Spain

One way of monitoring proton therapy treatments is by measuring the prompt gamma-rays generated as a by-product of the irradiation. A new compact measurement strategy is being developed: the Coaxial Prompt Gamma-ray Monitoring, where prompt gamma-rays are measured with a single fast scintillation detector (CeBr3) coupled to a PhotoMultiplier Tube positioned coaxially to the beam and behind the irradiated area. The principle of coaxial detection for range verification profits from the solid angle effect: the deeper the protons penetrate, the closer the distance between the generated prompt gamma-rays and the detector, and thus the higher the number of prompt gamma-rays detected. Also taking into account the radiation background produced during the irradiation, a count rate in the detector up to 10 million gamma-rays per second is expected, considering a proton beam kinetic energy of 125 MeV and a current at the target of 2 nA. A 1.5 inch diameter R13408-100 photomultiplier tube from Hamamatsu Photonics has been characterized with the help of controlled light sources and radioactive sources. After evaluating different custom-made voltage dividers, first experimental tests show that an active architecture is required in order to guarantee the system stability. Also a dead-time-free and trigger-less data acquisition system based on a fast ADC board (2.5GS/s) has been developed and tested. Furthermore we show the most recent developments regarding some other parts of the detector, as an interface board between the detector and the clinical acccelerator and an interface board between the detector and the ADC board.

### P 163 - A dedicated dual-head TOF-PET system for in-vivo quality control of beam delivery in proton therapy

#### Pedro Rato Mendes^1^, Pedro Arce^1^, Juan Ignacio Lagares^1^, Natalia Chamorro^1^, Oscar Vela^1^, Jesus Marín^1^, Borja Aguilar^2^, Leticia Irazola^2^, Juan Diego Azcona^2^

##### ^1^CIEMAT, Technology Department, Madrid, Spain ^2^Clínica Universidad de Navarra, Service of Radiation Physics and Radiation Protection, Madrid, Spain

We are developing a prototype dual-head time-of-flight (TOF)-PET system for in-vivo verification of beam delivery in proton therapy. Such a system will allow PET image acquisition shortly after irradiation, profiting from imaging of short-lived O-15 and reduced biological washout effects, whereas TOF-PET reduces image degradation due to incomplete acquisition of projection data from the planar detectors. Our proposed detector has a modular design with two parallel planar heads of approximately 20 cm by 20 cm active area, each consisting of 8 × 8 detector modules, composed of a LYSO:Ce array of 3.14 mm × 3.14 mm × 20 mm elements, optically coupled to SiPM arrays with TOF-enabling HRFlexToT ASIC readout [1]. The expected performance of the detector has been assessed via realistic Monte Carlo simulations of anonymized real patient treatments, by comparing the PET images from the original treatment with images obtained with artificially-modified proton ranges. Using a range estimation method developed by our group, we have concluded that the proposed PET system is capable of identifying range differences of the order of 1 mm with over 80% sensitivity and specificity, and 100% for range variations of 2 mm or larger [2-3]. We are presently developing a small field of view prototype with two planar heads of 10 cm by 10 cm to validate the proposed PET detector design.

### P 164 - A 3D-printed automated 1D water tank for range verification of low energy proton beams

#### Garett Roe^1^, Tomi Nano^2^, Kavita Mishra^2^, Sumi Sinha^2^, Jessica Scholey^2^

##### ^1^The University of California Davis, Physics, Davis, USA ^2^The University of California San Francisco, Radiation Oncology, San Francisco, USA

**Purpose**: To design and construct a 1D water tank capable of automated chamber positioning for measuring percent-depth-dose (PDD) curves in a compact ocular proton therapy-dedicated beamline where conventional QA tools are not practical and compare it to the current manual method in terms of data quality and time-cost savings.

**Methods**: The automated water tank was constructed using a custom graphical user interface (Python Tkinter toolkit), Arduino-driven microcontroller and stepper motor used to designate chamber position (0.5mm resolution), and 3D-printed housing (6x6cm^2^ wide, 7.5cm deep) with Markus chamber holder printed from polylactic acid (Ender-3 V2), shown in Figure 1. Measurements were made on the UCSF-UCD Crocker horizontal beamline (57MeV at isocenter) to determine tank window water-equivalent-thickness and compare pristine Bragg peak PDDs acquired using the proposed automated solution versus the manual method achieved by moving the chamber manually between exposures.

**Results**: The final automatic water tank prototype took 35 hours to fabricate and $37 in manufacturing costs. PDDs acquired using the automated 1D water tank agreed with manual measurements to within 0.5mm (R_90_ of 29mm vs. 29.5mm, respectively), shown in Figure 2. A typical PDD measurement took 10 minutes versus 1.6 hours for the automated versus manual methods, respectively, resulting in an estimated reduction of PDD QA beamtime of ∼90%.

**Conclusion**: PDDs acquired using the automated water tank were in close agreement (0.5mm, within measurement uncertainties) with ground-truth manual acquisition. Considerable beam time-related cost- and time-savings can be achieved by measuring PDDs using the proposed low-cost and open-source automated 3D-printed water tank.

### P 165 - Analysis of machine incident reports for a newly installed single-room gantry mounted active scanning proton therapy system

#### Ryan Price^1^, Sara St. James^1^, Adam Paxton^1^, Vikren Sarkar^1^, Christian Dial^1^, Frances Su^1^, Bill Salter^1^

##### ^1^University of Utah Huntsman Cancer Institute, Radiation Oncology, Salt Lake City, USA

**Purpose**: Single-room gantry-mounted active-scanning proton therapy systems are complex with non-trivial opportunity for downtime that can be disruptive to the clinical workflow. This work summarizes machine incidents and downtime experienced on one of these commercially available systems in the first 2 years of operation.

**Methods**: For 22 months of initial clinical operation, our facility recorded and categorized error logs associated with our MevionS250i. Details of errors and subsequent fixes were recorded whenever possible, and downtimes recorded. Events that resulted in clinical delays of at least 1 minute were recorded as downtime anddistributions of most common events were analyzed.

**Results**: Six common categories of downtime were identified. Adaptive coil positioner (ACP) related errors accounted for 6.0% of recorded events, outer gantry 6.4%, range-shifter 41.6%, adaptive aperture(AA) 11.5%, dosimetry circuit 2.7%, and slow discharge(SD) events (partial or complete magnet current loss) 1.0%. However, in percentages of total downtime ACP accounted for 10.5%(15.2hr), outer gantry 0.7%(1.1hr), range-shifter 11.4%(16.5hr), AA 23.9%(34.7hr), dosimetry circuit 5.4%(7.8hr), and SD 22.2%(32.3hr). Downtime also became less frequent over time, with 54.0% of downtime occurring in the first 3 months of clinical operation, and the relative amount of SD related downtime decreasing by half.

**Conclusions**: The proportion of errors was dominated by range-shifter related problems, however these did not reflect a proportional amount of downtime. While occurring much less frequently, problems with the AA or problems resulting in SD were more likely to cause large downtime events. Regardless, errors became much less common after the first few months of operation.

### P 166 - A Monte Carlo-based approach for secondary dose calculation for a gantry-mounted pencil beam scanning proton therapy system

#### Xinyuan Chen^1^, Arash Darafsheh^1^, Tianyu Zhao^1^

##### ^1^Washington University School of Medicine, Radiation Oncology, St. Louis, USA

**Purpose**: Currently, dose calculated in the treatment planning system (TPS) doesn’t allow users to modify the physics list or acquire information other than total dose. Therefore, this study is to develop an independent Monte Carlo simulation (MCS) tool on TOPAS for secondary dose calculation (SDC) for a gantry-mounted pencil beam scanning proton therapy machine and provide additional information such as neutron dose for pacemaker consultation and linear energy transfer (LET) supporting clinical decisions.

**Methods**: In the SDC, initial kinetic energy, energy spectrum, size, and divergence of the proton beam were determined by fitting measurements. Range shifter, block, and exiting window were simulated to include interactions of secondary particles. Patients’ CT simulation, as well as machine-specific Hounsfield unit (HU)-to-density calibration, were imported for dose calculation, including LET and neutron dose if requested by physicians. The SDC dose was compared with the TPS dose on the same dose grid in terms of absolute dose and gamma test passing rate. 369 patients with 696 fields were investigated and reported.

**Results**: Excellent agreement between the TPS dose and SDC dose were observed with a percentage dose difference of −0.12% (4.18%) and more than 95% of the fields having gamma 3%, 3mm passing rate greater than 95%. For a patient case included in this study, mean and maximum neutron dose to the pacemaker was 0.04 cGy/fraction and 1.29 cGy/fraction, respectively.

**Conclusion**: Independent MCS platform provides secondary dose verification as well as vital information such as neutron dose to a pacemaker and LET for proton therapy system.

### P 167 - Background study in a Compton camera for treatment monitoring

#### Marina Borja Lloret^1^, Luis Barrientos^1^, Fernando Hueso-González^1^, Javier Pérez-Curbelo^1^, Ana Ros^1^, Jorge Roser^1^, Rita Viegas^1^, Gabriela Llosá^1^

##### ^1^Instituto de Física Corpuscular UV-CSIC, Experimental, Paterna, Spain

Compton cameras are a prompt gamma imaging method proposed for in vivo hadron therapy monitoring. The work presented here is a detailed study of the background sources existing in a Compton camera based on the MACACO prototype developed by the IRIS group in IFIC (CSIC-UV). Simulations of two monolithic scintillator planes of LaBr_3_ have been carried out in GATE v8.2 with a proton beam delivered into a PMMA phantom. The background composition and image degradation have been evaluated at different proton beam energies and beam currents. In addition, various time coincidence windows (TCW) have been employed to collect events with the two planes working in coincidence. In Figure 1, spatial images are reconstructed for the proton simulations of 150 MeV at different beam intensities (rows) and TCW (columns). This figure shows that detectors with high time resolution are needed to minimize the amount of measured random coincidences and retrieve the fall-off position with better precision, specially at high beam intensities. The contrast-to-noise ratio (CNR) increased from 0.7 (TCW=50 ns) to 12.7 (TCW=500 ps) at a beam intensity of 10^10^ protons/s. Images are also degraded by photonic background and by coincidences produced by secondary radiations arriving from the phantom. For example, neutrons indirectly cause the 19.4% of coincidences at 10^9^ protons/s (Figure 2), and deposit energies similar to photons. Therefore, besides the improvements of the timing capabilities of MACACO, other background rejection methods are being explored to enhance the performance of the Compton camera.

### P 168 - A compact detector for eye therapy quality assurance at the Clatterbridge Cancer Centre

#### Simon Jolly^1^, Saad Shaikh^1^, Sonia Escribano-Rodriguez^1^, Pannell Fern^1^, Connor Godden^1^, Derek Attree^1^, Raffaella Radogna^2^, Linda Mortimer^3^, Peter Corlett^3^

##### ^1^University College London, Physics & Astronomy, London, United Kingdom ^2^University of Bari, Physics, Bari, Italy ^3^Clatterbridge Cancer Centre, National Centre for Eye Proton Therapy, Wirral, United Kingdom

To ensure optimal patient safety with Proton Beam Therapy (PBT), several beam properties are measured as part of Quality Assurance (QA), with the proton range in water being key a parameter. The Clatterbridge Cancer Centre (CCC) in Wirral, UK is a specialised facility for the treatment of eye cancers. CCC utilises a passively-scattered 60 MeV proton beam and is currently undergoing an overhaul of its control and QA systems. Measurements of the proton depth dose curve are carried out as part of the pre-treatment weekly QA but current methods are time-consuming and utilise a diode which degrades. A 60 MeV version of the Quality Assurance Range Calorimeter (QuARC) is currently under development at UCL to provide a compact and efficient solution for range QA measurements at the CCC. The detector utilises a series of optically isolated scintillator sheets where each sheet is coupled individually to a photodiode in order to sample the proton depth-light distribution. Fitting to an analytical depth-light model, the original depth-dose curve is reconstructed and the proton range is measured in real-time to sub-mm precision, without any optical artefacts. The model can be extended to fit SOBP depth-light data, wherein the original SOBP is recovered along with its distal range. Presented are measurements with a prototype compact QuARC of the 60 MeV pristine Bragg curve at the CCC as well as the SOBP. Experience with a dedicated web-based GUI, providing an easy-to-use, installation-free system for data display and detector control over local-area network is also discussed.

### P 169 - Are track dosimeters a good choice for neutron personal dosimetry?

#### Evangelina Martínez Francés^1^, Verónica Morán Velasco^1^, Teresa Cuenca Bandin^2^, Pedro Borja Aguilar Redondo^1^, Leticia Soria Ruiz-Ogarrio^1^, Josep María Martí Climent^1^

##### ^1^Clínica Universidad de Navarra, Medical Physics and Radiation Protection, Madrid, Spain ^2^Clínica Universidad de Navarra, Medical Physics and Radiation Protection, Pamplona, Spain

**Purpose**: To map neutron stray radiation around the target volume in scanning proton therapy.

**Materials and Methods**: Neutron ambient dose equivalent, H*(10), was measured at several positions inside treatment room (Figure 1) with two neutron extended rem-counters (WENDI-II, Thermo Scientific). Neutron personal dose equivalent, H_p_(10), was measured with track dosimeters (Neutrak, Landauer) at four positions (1-2, 4-5). Personal neutron dosimeters have a dose measurement range from 100 µSv to 250 mSv and an energy range from 0.25 eV to 40 MeV. A solid water phantom (60x30x30 cm^3^) was irradiated using a proton pencil beam with a 10 cm SOBP, maximum energy of 173.1 MeV, and a 10x10 cm^2^ field size. Dose delivered at isocenter was 48.75 Gy to achieve Neutrak lower limit of detection at measurement positions. For each measured point, relation between H*(*10*) and dose delivered at the isocenter was calculated.

**Results**: The highest H*(*10*), 197 µSv/Gy, was measured at position 7. The lowest H*(*10*) value, 4 µSv/Gy, was measured at position 2. At position four, *H_p_*(*10*) and H*(*10*) were similar, around 430 µSv, in contrast at position 2, *H_p_*(*10*) was null and H*(*10*) was ∼200 µSv. This discrepancy is explained because Neutrak dose measurement range.

**Conclusions**: Given the calculated neutron map and the response of Neutrak dosimeters, if a person is trapped in the treatment room during a clinical session, the Neutrak dosimeters would register a null dose. However, H*(*10*) could be estimated thanks to the neutron mapping within the room.

### P 170 - Dosimetric impact of spot size variation in proton treatment plan quality

#### David Reboredo Gil^1^, Adam Aitkenhead^1^, David Lines^1^, Matthew Lowe^1^, Peter Sitch^1^, Matthew Clarke^1^

##### ^1^The Christie NHS Foundation Trust, Christie Medical Physics and Engineering, Manchester, United Kingdom

**Background**: Deviations in proton spot size can potentially impact the quality of patient’s treatment, although the magnitude of this impact is not well quantified. The purpose of this study was to assess the dosimetric impact of spot size variation in various proton treatment plans.

**Method**: Changes in spot size of ±10% and ±20% from baseline values measured in air during beam model commissioning were evaluated. This represents the typical magnitude of the largest variations observed during routine quality assurance (QA) as well as a more extreme evaluation scenario. The resulting dose distributions were simulated in an in-house Monte Carlo system. A selection of complex clinical sites were evaluated including oropharynx, nasopharynx, base of skull and spine.

**Results**: For some cases, moderate impact to CTV coverage and changes in dose to serial critical organs at risk (OAR) adjacent to treatment volumes were observed. Parallel OAR mean doses did not seem to be significantly impacted. Though this study looked at the impact of spot size deviation without considering additional uncertainties like setup, range and anatomical changes on top of this, the magnitude of these changes were found to be small compared to these uncertainties.

**Conclusions**: Results from this study were used to review the current departmental spot size QA tolerances and to provide advice where QA results are above action levels. Current action levels (at 10%) were considered to be appropriate for clinical use.

### P 171 - Terapet’s quality assurance device for hadron therapy: First in-silico performance evaluation

#### Andrea Polesel^1^, Etiennette Auffray^1^, Ben Brunt^2^, Marcus Palm^2^, Michael Betz^2^, Robin Chappuis^2^, Luca Terradura^2^, Christina Vallgren^2^

##### ^1^CERN, ep-cmx, Meyrin, Switzerland ^2^Terapet, Sa, Geneva, Switzerland

Quality assurance is essential in the field of hadron therapy, in order to minimise the dose delivered to healthy tissue and to maximise that absorbed by the target. In this study we evaluate an in-situ, non-invasive quality assurance device developed by Terapet SA, for use with phantoms or head-sized anatomical targets. The detector consists of multiple scintillator-based modules, with its field of view extended by motion of the detector modules. Activity within the target is reconstructed in three dimensions using PET methods. Such a device would be helpful in various use cases, for example to detect changes in the delivered beam or beam-target alignment from any gantry angle or couch-top orientation (a constancy check), assessing the validity of Treatment Planning System modelling of a target, or to investigate the reproducibility of setup routines. A simulation campaign was carried out using the GEANT4 toolkit. The simulation was divided into multiple steps: first the interaction of protons was simulated in order to obtain information on the activation of the target, then positron-emitting isotopes were generated accordingly inside the target and the gamma rays traced to the detector where their interaction information was used for optical ray-tracing of the scintillation light produced and simulation of detector response. As a result of this work, we will present the detector sensitivity and reconstructed point source resolution, in addition to performance metrics specific to the proposed operating modes of the device.

### P 172 - Sensitivity to detector misalignment in carbon-ion treatment monitoring with charged nuclear fragments

#### Pamela Ochoa-Parra^1,2,3^, Luisa Schweins^1,2,3^, Laurent Kelleter^1,2,4^, Laura Ghesquiere-Dierickx^1,2,5^, Nelly Abbani^1,2,6^, Jürgen Debus^1,2,4,7,8^, Oliver Jäkel^1,2,4,7^, Maria Martišíková^1,2,4^

##### ^1^German Cancer Research Center DKFZ, Department of Medical Physics in Radiation Oncology, Heidelberg, Germany ^2^National Center for Research in Radiation Oncology NCRO, Heidelberg Institute for Radiation Oncology HIRO, Heidelberg, Germany ^3^University of Heidelberg, Department of Physics and Astronomy, Heidelberg, Germany ^4^NCT, National Center for Tumor diseases, Heidelberg, Germany ^5^University of Heidelberg, Medical faculty, Heidelberg, Germany ^6^University of Heidelberg, Medical faculty Mannheim, Heidelberg, Germany ^7^Heidelberg University Hospital, Heidelberg Ion Beam Therapy Center, Heidelberg, Germany ^8^Heidelberg University Hospital, Department of Radiation Oncology, Heidelberg, Germany

Carbon-ion radiotherapy is highly sensitive to treatment uncertainties such as the inter-fractional patient’s anatomical changes or detector misalignment. Our research group has been investigating in-vivo, and non-invasive monitoring methods based on tracking charged fragments emitted in nuclear interactions of carbon ions with patient tissue. Trackers based on silicon pixel detectors are used to measure tracks of emerging fragments. Previous studies of our group have demonstrated the capability of detecting and localizing air cavities in a homogeneous head-sized phantom. In this work, we investigated whether minor displacements of the detector could decrease the performance of the method in terms of detection and localization of anatomical changes. FLUKA Monte Carlo simulations were performed on a homogeneous head-sized phantom irradiated with a clinic-like treatment plan with an RBE-weighted dose of 1.5 Gy (RBE) using a horizontal beam. Seven mini-trackers each with a sensitive area of 4 cm2 were placed in accordance with the tracker system used in the In-Vivo Monitoring (InViMo) clinical study. Simulations were compared with data measured at the Heidelberg Ion Beam Therapy Center (Germany). It was found that the largest difference in the fragment track distribution was observed for displacements along the vertical axis, where shifts above 0.5 mm lead to p-values lower than the detectability threshold. The detectability and localization were found not to be affected by shifts along the horizontal and beam axes up to 2 mm, which exceeds the maximum detector position uncertainty. Based on these findings, offline detector misalignment corrections were implemented in the data post-processing.

### P 173 - Improvement of fragmentation-vertex reconstruction in carbon-ion treatment monitoring based on Monte Carlo simulations

#### Luisa Schweins^1,2,3^, Pamela Ochoa-Parra^1,2,3^, Laurent Kelleter^1,3,4^, Laura Ghèsquiere-Diérickx^1,3,5^, Nelly Abbani^1,3,6^, Jürgen Debus^1,3,4,7,8^, Oliver Jäkel^1,3,4,7^, Maria Martišíková^1,3,4^

##### ^1^German Cancer Research Centre DKFZ, Department of Medical Physics in Radiation Oncology, Heidelberg, Germany ^2^University of Heidelberg, Department of Physics and Astronomy, Heidelberg, Germany ^3^National Centre for Research in Radiation Oncology NCRO, Heidelberg Institute for Radiation Oncology HIRO, Heidelberg, Germany ^4^NCT, National Center for Tumor diseases, Heidelberg, Germany ^5^University of Heidelberg, Medical faculty, Heidelberg, Germany ^6^University of Heidelberg, Medical faculty Mannheim, Heidelberg, Germany ^7^Heidelberg University Hospital, Heidelberg Ion Beam Therapy Center, Heidelberg, Germany ^8^Heidelberg University Hospital, Department of Radiation Oncology, Heidelberg, Germany

Since carbon-ion radiotherapy is sensitive to treatment uncertainties such as morphological changes in the patient anatomy, it is desirable to have non-invasive in-vivo treatment monitoring systems. Our group has been developing a monitoring method based on the detection and tracking of charged nuclear fragments. A pair of pixelated semiconductor detectors (4 cm^2 sensitive area) are used as a mini-tracker to measure the tracks of the emerging fragments and to reconstruct the fragmentation point in the patient. To improve sensitivity limits of the method, the distance between the reconstructed fragment origin and ground-truth fragmentation vertex were studied using FLUKA Monte Carlo simulations. A homogeneous phantom was irradiated with a horizontal beam. The pencil-beam energy and position were varied within therapeutic ranges. The mini-tracker was rotated around the vertical axis between 20° and 40°. It was found that the distance between the reconstructed vertex and the ground-truth origin is dominated by the spatial resolution of the reconstruction along the beam axis, which increases with a larger observation angle. This leads to greater precision for increasing detector rotation angles and for fragmentation points that are further away from the detector. In contrary, the reconstruction of the vertical coordinate depends mainly on multiple coulomb scattering. The shorter the distance between the fragmentation vertex and the detector, the closer the ground-truth origin is to the reconstructed fragment track. Based on this knowledge, a correction procedure was developed increasing the reconstruction precision along the vertical axis by 10-25%.

### P 174 - Monte Carlo simulations and measurements of microdosimetric spectra with the Silicon MicroPlus probe in a 238.6 MeV/u carbon ion beam

#### Sandra Barna^1^, Cynthia Meouchi^2^, Andreas Franz Resch^3^, Giulio Magrin^3^, Dietmar Georg^1^, Hugo Palmans^3^

##### ^1^Medizinische Universität Wien, Universitätsklinik für Radioonkologie, Vienna, Austria ^2^Technische Universität Wien, Atominstitut, Wien, Austria ^3^MedAustron Ion Therapy Center, Medical Physics, Wr. Neustadt, Austria

**Introduction**: Monte Carlo (MC) simulations of microdosimetric spectra are available for different codes, detectors and particle types/energies. While good results can be achieved if the detector geometry is modelled in detail [1,2], a simpler approach can also result in very good agreement.

**Materials and Methods**: At five positions along a 238.6 MeV/u carbon ion pencil beam, spectra were collected using the Silicon MicroPlus probe. The measurements were collected along CAX as well as 10 mm off-center. The MC was performed with GATE/Geant4, using our validated beam model and a simplified detector geometry. Only the 400 sensitive volumes (diameter = 20µm, thickness = 10 µm, spaced 50 µm apart) were simulated, none of the housing/surrounding materials.

**Results**: The off-center measurements show a higher contribution of secondary particles, which we were able to reproduce in the MC (Figure 1). The agreement of the shape of the spectra is excellent for all positions along the Bragg peak. Especially in the fragmentation tail, where we were able to measure and simulate the boron edge with high accuracy.

**Discussion**: This simplified detector geometry could be implemented in future GATE installation checks, to perform a validation down to the microdosimetric scale. MC also offers a spectrum down to very low keV/µm values, which can be used to complete any experimentally obtained spectrum.

### P 175 - Investigation of PTW’s Octavius 1600 XDR with regard to field flatness analysis at the Heidelberg Ion Beam Therapy Center (HIT)

#### Julian Horn^1^, Benjamin Ackermann^1^, Stephan Brons^1^, Harald Latzel^1^, Oliver Jäkel^2^

##### ^1^Heidelberg Ion Beam Therapy Center at the Heidelberg University Hospital, Medical Physics, Heidelberg, Germany ^2^German Cancer Research Center- DKFZ, Division of Medical Physics in Radiation Oncology, Heidelberg, Germany

2D-Detectors play a significant role in particle therapy when it comes to acceptance testing and QA of beam parameters such as spot position and size, energy constancy and 2D-Flatness. The Octavius 1600 XDR, a dedicated 2D-IC-array for machine-QA in particle therapy by PTW Freiburg, was investigated towards its suitability for field flatness analysis at HIT. Among others, the detector was examined with regard to contrast and spatial resolution, i.e. the ability to detect signal variations dependent on both the signal intensity and the spatial size of the signal. Beforehand, the background signal stability, dose linearity and dose rate dependency of the detector had been successfully checked. In principle, the Octavius 1600 XDR can be used for field flatness analysis with restrictions concerning the detector’s measurement ranges: The measurement range for high dose rates does not match HIT’s accelerator settings resulting in noisy and therefore unusable data. Hence field flatness measurements are limited to the range for low dose rates (max. 67 mGy per 100 ms) and the QA plan design has to account for this, e.g. by limiting the dose applied per field. Anyhow, the detector has been successfully implemented in HIT’s machine QA program and is being used for spot size determination, energy constancy checks and field flatness analysis.

### P 177 - The transition to a novel Oncology Information (OIS) and Treatment Control System (TCS) in a busy dual particle facility

#### Piotr Andrzejewski^1^, Christopher Piribauer^1^, Markus Stock^1^, Johannes Hopfgartner^1^

##### ^1^MedAustron Ion Therapy Center, Medical Department, Wiener Neustadt, Austria

Due to technical advancements in the beam delivery system, a growing number of patients and expiring service and development of the currently used clinical software suite our facility faced the need to exchange the Oncology Information System (OIS) and Treatment Control System (TCS). As no off-the-shelf solution was suitable to interface our already existing systems, an implementation project was started in collaboration with one of the radiation oncology software vendors. The constantly ongoing developments to our custom build, heavy ion accelerator and ramp up of patients under treatment posed a challenging environment for such a project, heavily impacting every field and profession of the clinical department (over 100 users). For a standalone center without direct link to another hospital environment a stable, independent patient and clinical workflow management as well as a record and verify system are of pivotal importance. The pitstop-like, over-the-weekend transition to the novel clinical software suite was preceded with a close cooperation with the vendor to develop a comprehensive solution covering and improving all existing and planned treatment procedures. Followed by configuration, commissioning, acceptance and training it led to a successful implementation in the clinical environment. It was the first clinical go-live of the entire clinical software suite of that vendor consisting of that OIS, TCS and also treatment planning system (already previously in use but requiring an upgrade). This work includes the brief description of all above mentioned processes as well as a summary of how the new software configuration influenced the clinical workflow.

### P 178 - Summary of patient specific QA results for the first year of an IMPT center

#### Joshua Jardenil^1^, Robert Kaderka^1^, Mariluz De Ornelas^1^, Michael Butkus^1^

##### ^1^University of Miami, Radiation Oncology, Miami, USA

**Purpose**: An aggregation of patient-specific quality assurance (PSQA) gamma index rates from 7 months of a single-room proton center was collected to analyze trends in measurement fidelity.

**Methods**: Four hundred thirty (430) fields from 106 consecutive patients were analyzed using a 3%/3mm gamma criteria. Planar measurements were taken with a MatrixxPT ion chamber array at varying depths under RW3 solid water. Plan delivery was on a Varian ProBeam Compact delivery system with plans created in Eclipse Treatment Planning System. For all fields of one plan, a single depth was chosen near the area of maximum dose. Gamma rates were analyzed in subsets of measurement depth and treatment site.

**Results**: Of the 430 fields, 37 fields had measured gamma rates <95% with 8 of those < 90%. Figure 1 shows gamma rates as a function of measurement depth. An average gamma rate of 98.8%±2.8% was measured for all fields. For depths of 0 to 12 cm an average gamma rate of 99.1%±2.4% (n=422 fields) is seen compared to 94.5%±2.6% (n=8 fields) for depths greater than 12 cm. Figure 2 displays measured gamma rates by treatment site with average gamma rates ranging from 93.7% to 99.8%.

**Conclusion**: When using the 3%/3mm criterion we observed a trend towards reduced gamma rates for depths greater than 12 cm. Accordingly, treatment sites with larger depths trend towards lower gamma rates potentially requiring a replan.

### P 179 - A daily quality assurance test for volumetric repainting using magnetic field regulation

#### Eduardo Pons^1^, Andrew Wroe^1^, Alonso N. Gutiérrez^1^, Kristen McConnell^1^

##### ^1^Miami Cancer Institute, Radiation Oncology, Miami, USA

**Purpose**: A daily QA test was created to validate beam delivery accuracy and long-term stability of field regulation controlled volumetric repainting.

**Methods**: Field regulation utilizes a look-up table to account for magnetic field hysteresis, allowing for layer switching in both distal to proximal (DP) and proximal to distal (PD) directions without the need to power down the magnet. A daily QA test was generated to evaluate the proton system in this configuration. In one irradiation session, the test plan delivered a pre-defined spot map in the DP and PD directions. The IBA Lynx and Sphinx analyzed output, energy/spot characteristics, coincidence, and homogeneity. This work focused on examining the impact of layer delivery direction on distal/proximal depth, spot position/size, and homogeneity. To evaluate the QA test for a known error, a 2mm sheet of solid water was added in front of the Sphinx to induce a quantifiable range shift.

**Results**: Measurements were taken over 5 independent setups. The average deviations in spot position between delivery direction had a maximum of 0.3±0.19mm in X and 0.4±0.19mm in Y, while average deviations in spot size, range, and homogeneity were less than 0.1mm. With the 2mm sheet of solid water (2.06mm WET) added, distal and proximal depths were shifted by an average of 2.09mm and 2.10mm in the DP direction and 2.13mm and 2.18mm in the PD direction respectively.

**Conclusion**: Spot positions and range can be successfully detected within ±1 mm of baseline using field regulation. Long term stability data is currently being collected.

### P 180 - Acquisition of integral depth dose for the Varian ProBeam 360° pencil beam scanning proton therapy system

#### Yun Yang^1^, Lei Dong^2^, Virginia Lockamy^1^

##### ^1^Penn Medicine - Virtua Health Proton Center, Radiation Oncology, Voorhees, USA ^2^Penn Medicine, Radiation Oncology, Philadelphia, USA

**Purpose**: This study measured and characterized the integral depth dose (IDDs) of a Varian ProBeam 360° single gantry room proton therapy system for treatment planning system commissioning.

**Methods**: The ProBeam 360° system has six‐dimensional robotic patient support system, 360° rotating gantry, and two orthogonally onboard kV imaging panels (43cmx43cm) with CBCT capability. The superconducting cyclotron (AC226) has a maximum extraction energy of 226MeV and provides clinical energies from 69 to 218 MeV. IDDs were collected for energies between 70MeV and 218MeV with 10MeV intervals in both vertical (G0) and horizontal (G90) directions (Table1). IDDs were corrected for the entrance window water equivalent thickness (WET), including PTW Bragg peak chamber (Model 34070, 4.05mm), reference chamber (Model 34080, 2.10mm), and 3D water tank (model MP3-PL, 5.00mm). The distal range of 80% (R_80_), 20% (R_20_), and distal fall-off (DFO=R_20_–R_80_) were analyzed.

**Results**: Compared to both National Institute of Standards and Technology (NIST) and Varian specifications, the differences for R_80_ were within 0.4mm for IDDs measured at G0. Between IDDs measured at G0 and G90, the differences for R_80_ were also within 0.5mm. There was no difference for DFOs among IDDs measured at G0, IDDs measured at G90, and Varian specifications (Table1). The IDDs were normalized by outputs at 15mm WET and 1.1 RBE to commission Eclipse (v16.1) as shown in Figure1.

**Conclusions**: The IDDs were acquired for the new ProBeam 360° system with both vertical and horizontal scans. Results showed great agreements with NIST and Varian specifications and provided a reference for future commissioning.

### P 181 - Application of PHITS Monte Carlo code in ionization chamber dosimetry of high energy proton beams

#### Mark Ka Heng Chan^1^, Jörg Wulff^2^, Kilian Baumann^3^, Justin Malimban^1^, Stefan Both^1^

##### ^1^University Medical Center Groninge, Radiotherapy, Groningen, Netherlands ^2^West German Proton Therapy Center WPE and University Hospital Essen, Particle Therapy, Essen, Germany ^3^University Medical Center Giessen–Marburg and Marburg Ion–Beam Therapy Center, Radiotherapy and Radio–oncology, Marburg, Germany

**Background**: To investigated the potential of PHITS [1] Monte Carlo (MC) code in fQ calculation, the proton and chamber–specific part of the MC–calculated kQ.

**Methods**: PHITS was used to calculate fQ values of two bare cylindrical (CYL) and parallel–plate (PP) air cavities [Ref. 2] for inter–code comparisons. All simulations used a 150 MeV parallel (10 × 10cm2) mono–energetic proton source. fQ values of the PP NACP–02 were also calculated for 150, 200 and 250 MeV monoenergetic protons.

**Results**: Fig.1 show the fQ values of the CYL and PP cavities calculated by various MC codes. PHITS (INCL nuclear interaction (NI) model) deviated from PenH (with NI), FLUKA, Geant4/TOPAS–opt3 and –opt4 (Urban and WentzelVI models of electromagnetic interactions + BIC–NI) by −0.16, −0.42, −0.33, −0.68% for CYL and by −0.09, −0.44, −0.61, −0.88% for PP cavities [2], respectively. These de viations were within 2 standard deviations among various MC codes. Fig. 2 shows the fQ values of NACP–02. Deviations of PHITS from PenH without and with NI within the chamber geometry, FLUKA, and Geant4–opt4 using Bertini– and BIC–NI models were 1.44, −0.19, 0.73, −0.32, and 0.03% for 150 MeV monogenetic protons, −1.84, −0.88, 0.43, −0.08, and −0.28% for 200 MeV, and −1.33, −0.29, 1.67, 0.46, and 0.16% for 250 MeV, respectively.

**Conclusions**: PHITS yielded closest fQ values to PenH for the simplified IC geometries and closest to Geant4–opt4 + BIC for NACP–02. PHITS could be helpful to investigate this issue by 1) providing further fQ/kQ values and 2) checking on different NI models.

### P 182 - Commissioning of first proton therapy in NCCS: Range verification of single energy CT calibration in biological tissues

#### Wei Yang- Calvin Koh^1^, Hong Qi Tan^1^, Kah Seng Lew^1^, Andrew Wibawa^1^, Zubin Master^1^, Kit Loong Jeffrey Tuan^1^, Sung Yong Park^1^

##### ^1^National Cancer Centre Singapore, Radiation Oncology, Singapore, Singapore

To determine the HU to RSPR calibration curve accuracy using the single energy CT calibration method with a Hitachi Probeat Proton Therapy System and RayStation. In this study, lamb tissues were CT scanned with two energies (80 and 120 kVp) using GE Revolution CT scanner. The CT calibration was done using a stoichiometric method suggested by Schneider et al and two relative stopping power ratio (RSPR) curves were generated. The empty spaces in the container were packed with lamb meat and fully submerged in water. They were immediately irradiated, and the proton range is measured using a scintillator-based detector (Ranger-300, Logos Systems Int’l CA, USA) as shown in Figure 1. Irradiation plans of the monoenergetic beam (E_1_: 160.8 MeV and E_2_: 210.9 MeV) were generated using RayStation TPS for each RSPR curve where E_1_ traverse through individual tissues and E_2_ traverse through individual and the composition of all tissues. The WET of the tissues were then evaluated between the measurement and calculated value from TPS. The WET differences (calculated WET – measured WET) of different individual tissue showed a maximum of 1.6% for both energies and RSPR curves. In the scenario where the irradiation passes through all tissues, the maximum WET difference yields a -1.3%. as shown in Table 1. In conclusion, these results showed a value lower than the first proposed 3.5% margin of the total range for robust optimization by Goitein and this supports the use of 3.5% range uncertainty in our clinic as a conservative limit.

### P 183 - Proton range detection using a spherical resonance ionoacoustic wave emitted from a gold fiducial marker embedded in an agar phantom

#### Taeko Matsuura^1^, Shota Sueyasu^2^, Koki Kasamatsu^3^, Ye Chen^1^, Naoki Miyamoto^1^, Seishin Takao^1^, Yasutoshi Kuriyama^4^, Tomonori Uesugi^4^, Yoshihiro Ishi^4^

##### ^1^Hokkaido University, Faculty of Engineering, Sapporo, Japan ^2^Hokkaido University, Graduate School of Engineering, Sapporo, Japan ^3^Hokkaido University, Graduate School of Biomedical Science and Engineering, Sapporo, Japan ^4^Kyoto University, Institute for Integrated Radiation and Nuclear Science, Osaka, Japan

Ionoacoustic range measurement is a promising approach to reducing the range uncertainty in particle therapy. In the previous study, we demonstrated a range estimation method using spherical ionoacoustic waves with resonant frequency (SPIRE) emitted from a gold fiducial marker (GM) currently used for patient positioning. In this study, we further investigated this method by using a millimeter-sized optical hydrophone (OH) and an agar-based phantom. A 100-MeV proton beam with a 27-ns pulse width was produced using a fixed-field alternating gradient accelerator and irradiated to a GM in the agar phantom. The OH was embedded in a custom-made agar-based sensor head (SH) for stable measurement and coupled to the phantom. Five different-sized SHs (2 × 2×2, 2 × 2×3, 3 × 3×3, 3 × 3×6, 5 × 5×8 cm^3^) were tested to examine whether the SH size affects the measured beam range. Then, the SPIRE was measured by OH with 3 × 3×6-cm^3^ SH placed at several positions on the phantom. The detected beam range by OH was compared to that measured by a film. The observed variation of the estimated beam range among different-sized SHs was less than 0.3 mm. The absolute beam range measured at three (one) position(s) distal (lateral) to the Bragg peak were 0.8, 0.7, 1.9 (distal), and 0.7 (lateral) mm longer than that measured by the film. From these results, we conclude that the small-sized SH enables the stable SPIRE measurement using OH on the phantom surface. Future studies should investigate the precise positioning of the GM, which may be the main source of the range error.

### P 184 - Monte Carlo calculation of ion recombination correction factor in Carbon Ion Beams

#### Marina Orts Sanz^1^, Edmond Sterpin^1,2,3^, Kevin Souris^1,4^, Severine Rossomme^1,4^

##### ^1^UC Louvain Institute for Experimental and Clinical Research, Molecular Imaging Radiotherapy and Oncology, Brussels, Belgium ^2^KU Leuven, Department of Oncology Laboratory of Experimental Radiotherapy, Leuven, Belgium ^3^Particle Therapy Interuniversity Center Leuven, Particle, Leuven, Belgium ^4^Ion Beams Applications, s a, Louvain La Neuve, Belgium

Ion recombination correction factor is well known for standard modalities of radiotherapy, but for radiotherapy modalities that require high LET particle beams, not all beam conditions are considered. In the section of TRS-398 dedicated to ion beams, a method to determine initial recombination (intra-track recombination) is addressed, but recent studies show that volume recombination (inter-track recombination) is significant, hence cannot be neglected. For ensuring adequate accuracy in dosimetry, ion recombination correction factors should be close to unit, which may require specific experimental conditions and construction design of the ionization chamber (voltage and air gap size). Monte Carlo simulations could help to optimize such parameters, but they should be validated first in known conditions with experimental data available. We present here the validation of a Monte Carlo calculation of the ion recombination correction factor for plane parallel ionization chamber exposed to ion beams. The simulation tool has been validated against Jaffe’s theory for initial recombination, Boag’s theory for volume recombination and experimental data for a 62 MeV/n at 5.8 Gy/min and 115 MeV/n at 15 Gy/min carbon ion beam. The simulations were in accordance with the experimental data and theoretical values (relative difference <0.1% between 20V and 500V). The low dose rate data showed good agreement with the theoretical values of Jaffe theory, which is expected as initial recombination is predominant for high LET particles. Higher dose rate values show not only presence of initial recombination, but also volume recombination, which is expected, as volume recombination increases with the dose rate.

### P 185 - Radiobiology-microdosimetry combined experiments: Simultaneous irradiation under a clinical carbon beam

#### Gabriele Parisi^1^, Sandra Barna^2^, Giulio Magrin^3^, Cynthia Meouchi^4^, Francesco Romano^5^, Giuseppe Schettino^6^

##### ^1^University of Surrey, Department of Physics, Guildford, United Kingdom ^2^Medical University of Vienna, Department of Radiation Oncology, Vienna, Austria ^3^MedAustron Ion Therapy Center, Physics, Wiener Neustadt, Austria ^4^Technische Universität Wien, Radiation Physics, Vienna, Austria ^5^INFN - Istituto Nazionale di Fisica Nucleare, sezione di Catania, Catania, Italy ^6^NPL - National Physical Laboratory, Medical Radiation Science, Teddington, United Kingdom

Microdosimetry is a dosimetry technique measuring the energy deposited by radiation into cell-like volumes, taking into account the stochastic nature of radiation interaction. The interest in the application of microdosimetry to hadron therapy is growing with the improving performances of detectors and with the increasing experimental evidence of its benefits. For instance, the measured microdosimetry quantities can be directly linked to radiobiological response, allowing to estimate the Relative Biological Effectiveness (RBE) through dedicated radiobiological models. Combined radiobiological-microdosimetry measurements are needed to identify and specify key parameters (i.e., critical volume) and their relationship to specific radiobiological endpoints. These will be fundamental to improve and to validate existing radiobiological models. One of the main challenges of such experiments is to reproduce the same irradiation conditions for cells and microdosimeters due to the high sensitivity of microdosimetric measurements. An experimental method has been developed that allows to simultaneously irradiate cells and microdosimeters with a relative positioning accuracy of about 150 μm. The method ensures therefore that the same experimental and irradiation conditions are met. The system, showed in figure 1, aligns two water-proof diamond microdosimeters of different thickness (8 μm and 1 μm), a PTW Microdiamond dosimeter, and 3 cell samples. The system was used to assess the RBE of T98G cells (glioblastoma multiforme) along a clinical SOBP of Carbon ions at the MedAustron centre. The method and the results of the first radiobiology-microdosimetry combined experiment will be presented including a critical evaluation of the Microdosimetric Kinetic Model (MKM).

### P 186 - Dose response of normoxic N-vinylpyrrolidone-based polymer gel dosimeter for proton therapy

#### Hideyuki Takei^1^, Yusuke Watanabe^2^, Takuya Maeyama^3^, Shinya Mizukami^2^

##### ^1^National Institute for Quantum Science and Technology, Quantum Life and Medical Science Directorate, Chiba, Japan ^2^Kitasato University, School of Allied Health Sciences, Kanagawa, Japan ^3^Kitasato University, School of Science, Kanagawa, Japan

**Purpose**: Polymer gel dosimeters are three-dimensional dosimeters which can measure complex dose distribution with high spatial resolution. In this study, the response of the gel dosimeter to the absorbed dose was investigated in proton therapy.

**Materials and Methods**: A normoxic N-vinylpyrrolidone-based polymer gel dosimeter was irradiated with passively scattered proton beams. The beams had the 30-mm wide spread-out Bragg peak (SOBP) with the range of 163 mm. The dose range was 0-50 Gy at the center of SOBP. The irradiated gel samples were scanned with MRI scanner to obtain R2 maps. The dose-averaged linear energy transfer (LETd) was calculated with Monte Carlo simulation to evaluate the LETd dependence of the sensitivity.

**Results and Discussion**: The response of R2 to the absorbed dose was evaluated (Figure). The R2 was linear to the dose up to 40 Gy. The decrease in the dose sensitivity was observed at the distal side of the SOBP where the LETd increased. The R2 decreased by 18% at the LETd of ∼10 keV/μm at the distal end of the SOBP compared to that at 8.3 keV/μm at the proximal side. The decrease in the sensitivity was corrected with the ratio of the dose sensitivity at high LETd to that at plateau.

**Conclusion**: The response of the gel dosimeter to the absorbed dose was investigated. The R2 was linear to the dose up to 40 Gy. The gel dosimeter can be a useful three-dimensional dosimeter for proton therapy with the correction of LETd dependence.

### P 187 - Dosimetric characterisation of proton linear accelerator

#### Giovanni Rutigliani^1^, Luigi Picardi^1^, Grazia Laricchiuta^1^, Francesca Rosa Coniglio^1^

##### ^1^Linearbeam srl, Particle Accelerator, Ruvo di Puglia, Italy

**Introduction**: The prototype of the ERHA PL230 is designed and under commissioning by LinearBeam company in Ruvo di Puglia (Italy). It consists of a low frequency (428 MHz) 4 MeV injector by AccSys-Hitachi company, followed by a sequence of 3GHz accelerating modules, of SCDTL type up to 27 MeV and of CCL type up to 230 MeV. Actually the system is assembled and operating up to 160 MeV.

**Method**: For a complete characterization of the therapeutic beam several dosimeters were used e.g. ionization chambers, plastic scintillator, water and solid phantoms equipped with gafchromic films. Moreover an innovative acoustic methods have been used for precise energy determination and beam profile. Spread out bragg peaks at different energies have been measured with fully automatic and fast modulation of the beam energy due to each accelerating structure is powered with a dedicated modulator. Fast high dose delivery can be reached thanks to 100 Hz repetition rate with very short pulse width (<3us) and tens of microamperes beam current.

**Results**: The commissioning of the linear accelerator in terms of energy, stability and repeatability was followed by dosimetry experiments that validated the beam quality. All measurements confirm a very high quality beam suitable for a precise and fast 4D beam therapy.

**Conclusion**: This achievement demonstrates the feasibility of a proton therapy accelerator a ’fully linear’ that operates at high frequency and opens the way to a new class of machines for the treatment of cancer

### P 188 - Measurement of nuclear reaction cross sections of 10C by the annihilation γ-ray detection system nBOLPs

#### Masaki Kato^1^, Teiji Nishio^1^, Takamitsu Masuda^2^, Toshiyuki Toshito^3^

##### ^1^Osaka University, Health Science, Suita-shi, Japan ^2^National Institutes for Quantum science and Technology, Accelerator and Medical Physics, Chiba, Japan ^3^Nagoya City University West Medical Center, Department of proton therapy physics, Nagoya, Japan

**Purpose**: Proton beam therapy has high dose concentration to tumors, but can damage normal organs if the proton beam is misaligned. The radioactivity distribution of positron-emitting radionuclides can be visualized using the dual-plane annihilation gamma-ray detectors (nBOLPs) installed on the beamline. Currently, the number and accuracy of cross section data are not sufficient to accurately simulate the activity distribution. We are aiming to measure 12C(p,p2n)10C cross sections in the high-energy region, which will improve the **accuracy** of irradiation near the body surface.

**Methods**: We irradiated polyethylene targets with mono-energetic 100.2 MeV and 150.5 MeV protons, as shown in Fig.1. After proton irradiation, the activity distribution was measured for 30 minutes. This enabled us to obtain cross sections in the energy range of 0∼100 MeV and 65-150 MeV at a time. The number of incident protons was determined from the dose measured with a Bragg Peak Chamber (PTW Type 34070) and the Mont Carlo simulation (PHITS 3.26).

**Results**: The 10C cross section results are shown in Figure 2. At 65∼100 MeV, the two results did not completely agree; the cross section results up to around 50 MeV and around 150 MeV agreed with the previous study to the extent of error bars. This may be due to the effects of scattering of gamma rays and systematic errors in the detector.

**Conclusion**: We have roughly obtained 12C(p, p2n)10C reaction cross section data from 70MeV to 150MeV, which corresponds to the body surface in the treatment of shallow depth cancer.

### P 189 - Sensor design for ionoacoustics-based proton beam dose monitoring in small animals

#### Pratik Kumar Dash^1^, Marco Pinto^1^, Martin Gerlei^1^, Grigory Liubchenko^1^, Ze Huang^1^, Neeraj Kurichiyanil^1^, Jessica Maas^2^, Kirsten Lauber^2,3^, Katia Parodi^1^, Julie Lascaud^1^

##### ^1^Ludwig-Maximilians-Universität München, Department of Medical Physics, Garching, Germany ^2^University Hospital- LMU Munich, Department of Radiation Oncology, Munich, Germany ^3^German Cancer Consortium DKTK, Partner site Munich, Munich, Germany

**Introduction**: Ionoacoustics (IA)-based dosimetry is currently being investigated for high-precision monitoring of proton beams in the context of a small-animal proton irradiator (SIRMIO project). It relies upon thermoacoustic waves emitted from the region irradiated by pulsed proton beams. These waves can be used to reconstruct the spatial distribution of dose deposited by the protons and localize its Bragg peak (BP) via techniques such as Time-reversal-reconstruction (TRR). Presented here is a simulation study where the accuracy and precision of dose reconstruction of pre-clinical beams were assessed for different sensor arrangements and irradiation scenarios.

**Method**: Dose deposited by a clinical proton beam degraded to pre-clinical energies by the SIRMIO beamline was simulated in Geant4. Simulations of IA signals in homogeneous water, detection by a disk sensor array and TRR, were all performed via the k-Wave simulation engine. Errors in localizing the BP were evaluated for various 2D and 3D sensor designs. For the selected sensor design, errors were also evaluated for multiple beam positions within the array’s field-of-view, emulating different pencil beams delivered during a complete treatment plan to ensure dose uniformity within the tumor.

**Results and Conclusion**: The selected sensor design consists of 3 semi-circular arcs placed in parallel planes. BP can be localized with an accuracy of less than 1 mm for all beam positions. Work is ongoing on studying the performance of such a design in reconstructing the different pencil beams in a treatment plan delivered to a mouse. Support from ERC (grant number 725539) is gratefully acknowledged.

### P 190 - Evaluating environmental radiation dose to ensure radiation protection during the trial operation at National Taiwan University Proton Therapy Center

#### Liang-Hsin Chen^1^, Linag Hsiang-Kuang Tony^1^, Kuo Sung-Hsin^2^

##### ^1^National Taiwan University, Department of Biomedical Engineering, Taipei, Taiwan Province of China ^2^National Taiwan University Cancer Center, Department of Radiation Oncology, Taipei, Taiwan Province of China

**Background and Purpose**: Radiation protection of proton is an essential and important issue in proton therapy center establishment. During the trial operation at National Taiwan University Proton Therapy Center (NTUPTC), we performed the environmental radiation evaluation to ensure radiation protection.

**Materials and Methods**: We evaluated and monitored environmental neutron and photon dose rates in the areas (Figure 1) near main control room (MCR), cyclotron (CYC) and beam transportation system (BTS) during proton beam being extracted from CYC (Varian ProBeam System). The environmental radiation dose rate is required to be <10 μSv/hr according to Taiwan’s regulation. The proton beam currents range from 10 to 800 nA with maximum energy of 250 MeV. The environmental radiation dose rates were measured by 12 sets comprising one neutron detector (THERMO FHT 762) and two gamma detectors (ATOMTEX AT 1121).

**Results**: Totally, the dose rates were monitored at 12 locations and the neuron and photon dose rates were mostly <1 μSv/hr (Table 1). The neutron and photon dose rates at C2 location near the energy degrader were relatively high than those at the other locations but remained within the required level.

**Conclusion**: During the trial operation at NTUPTC, we confirmed that all the environmental neutron and photon dose rates fulfilled the requirement of Taiwan’s regulation. Our results verified the shielding ability of build construction of areas near MCR, CYC and BTS through evaluating environmental radiation dose rates, which ensures the radiation protection and safety of NTUPTC.

### P 191 - Single-shot thermoacoustic measurements during high dose rate delivery with a clinical synchrocyclotron

#### Sarah Patch^1^, Tianyu Zhao^2^, Yao Hao^2^

##### ^1^Acoustic Range Estimates, all, Chicago, USA ^2^Washington University in St Louis, Radiation Oncology, St Louis, USA

**Aim**: We investigate feasibility of real-time thermoacoustic range verification during high dose rate delivery by a Mevion Hyperscan S250i.

**Materials/Methods**: 10-20pC/pulse were delivered to a 6”x6”x3” Lexan block into which a transducer was embedded distal to the Bragg peak (Fig.1a-b). Nuclear emissions were detected by a large (0.5m) plastic scintillator+PMT assembly that provided a measure of beam current, Thermoacoustic emissions were detected without averaging. For each beam range, a library of signals was simulated using TRIM and kWave software, assuming a point receiver, (σ_x,σ_y)=(29.0,27.4)mm, and Lexan properties ρ=1.2, Γ=2 and ν_s=2.0mm/us, and convolved simulated pulses with I(t). Time shifts between the first compressional phase of measured and simulated pulses were computed by application of the Fourier-Shift theorem. Treating ranges of the simulated beamlets as a function of time shift and evaluating at δt=0 by interpolation yielded thermoacoustic range estimates.

**Results**: Broadband noise caused shot-to-shot variations. After bandpass filtering, however, DC levels were consistent and variations were primarily in amplitude. Thermoacoustic range estimates agree with modeled range to within 1.17+/-0.47 mm for a 121.7 MeV beam, and decreased with energy.

**Conclusions**: Single shot estimates were accurate enough to detect gross range errors. Averaging 8 shots provided millimeter accuracy, although incorrect assumptions of stopping power and/or soundspeed could have caused systematic errors. Experiments should be repeated in clinical mode with small diameter beamlets to determine utility of lateral transducer locations. Simulations should be improved by modeling transducer dimension and impulse response.

### P 192 - EBT3 radiochromic film calibration in a PBS protontherapy facility

#### Alberto Viñals^1^, José Miguel Delgado^1^, Roser Fayós-Solà^1^, Pedro Borja Aguilar^1^, Diego Pedrero^1^, José Pablo Cabello^1^, Miguel García^1^, Carlos Ramón^1^, Elena Antolín^1^, Juan Diego Azcona^1^

##### ^1^Clínica Universidad de Navarra, Radiation Physics, Madrid, Spain

**Purpose**: To describe a simple method to calibrate EBT3 radiochromic film (RCF) in order to perform treatment verification with Pencil Beam Scanning (PBS) proton beam irradiation.

**Materials and Method**: RCF response depends not only on absorbed dose but also on the Linear Energy Transfer (LET) of the beam due to quenching effect. To take this dependency into account it has been developed a two-step calibration such that the proton beam absorbed dose measured with RCF is obtained as: Dref·F(LET), where Dref is the RCF measured dose using a 6MV photon calibration curve and F(LET) is the LET dependent correction factor required due to quenching effect. To obtain F(LET), proton beam irradiations were carried out with our PBS system using a 10x10cm^2^ formed by 1840 beamlets of single energy. Dref was measured in 5 depths nearby the Bragg peak position using an acrylic phantom. F(LET) was calculated as the quotient between the dose measured with PTW Advanced-Markus chamber and Dref measured at the same depths; the results were linearly fitted against the Montecarlo dose-averaged LET values.

**Results**: Figure1 shows the correction factors F(LET) obtained. Linear fit yields an analytical function which allows to correct the RCF measured dose depending on the LET value. This methology was verified correcting PDD curves of 10x10cm^2^ squared fields of several energies measured with RCF and comparing with Montecarlo simulations as shown in figure 2.

**Conclusions**: A simple method of EBT3 calibration with LET correction is presented which allows yielding of depth-profiles in depth-modulated beams.

### P 193 - Relative dosimetry for conventional/FLASH proton therapy with novel silicon carbide detectors

#### Giuliana Milluzzo^1^, Massimo Camarda^2,3^, Marc-Jan Goethem^4^, Pietro Pisciotta^4^, Francesco Romano^1,4^

##### ^1^Istituto Nazionale di Fisica Nucleare, Catania División, Catania, Italy ^2^STLab, Srl, Catania, Italy ^3^SenSiC, DeliveryLab, Villigen, Switzerland ^4^University Medical Center Groningen‚ Particle Therapy Research Center PARTREC, Department of Radiotherapy, Groningen, Netherlands

The identification of the proper dosimetry devices for QA in FLASH-RT is a crucial step towards its translation in the clinical routine. In this framework, the INFN Catania Division and the ST-Lab company have developed novel prototypes of silicon carbide detectors (SiC) with different thicknesses (0.2-100 um) and active areas (1-10 mm^2^) including a novel type of “free standing” membranes, where the thick (<350 um) substrates are removed, resulting in an improved detector transparency. Such devices were proved to be suitable for FLASH beam monitoring and dosimetry, having a response in charge perfectly linear with the instantaneous dose-rate up to 1.2 MGy/s, as recently demonstrated using FLASH 9 MeV electron beams. Therefore, they are very promising also for FLASH proton beams. The SiC detectors with substrates were preliminary tested with the 180 MeV proton beams accelerated at conventional dose rates at the UMCG PARTREC facility in Groningen (NL). The proton depth distributions, acquired with the SiC and the Advanced Markus chamber, are reported in Figure 1. Although the comparison shows an increased peak-plateau ratio for SiC detector, the latter still maintains a good agreement for practical range measurements (222.3 mm and 222.9 mm respectively). However, further investigations will be performed to better understand the dose over-response in the proximity of the Bragg Peak, which might be ascribed to the LET-dependent “funneling” effect. In the future perspective, the ultra-thin membranes will be also soon tested with conventional and FLASH proton beams for both low perturbation real-time monitoring measurements and relative dosimetry.

### P 195 - Comparison of OAR sparing between IMRT and PBS plans based on Ann Arbor staging for Hodgkin and aggressive non-Hodgkin lymphoma

#### Keaton Reiners^1^, Emma Viviers^2^, Nataly Getman^2^, Kevin Kirby^2^, Jonathan Fakhry^3^, Fantine Giap^3^, Eric Brooks^3^, Nancy Mendenhall^3^, Raymond Mailhot Vega^3^

##### ^1^University of Florida College of Medicine, Medical Physics, Gainesville- FL, USA ^2^University of Florida Health Proton Therapy Institute, Radiation Oncology, Jacksonville- FL, USA ^3^University of Florida College of Medicine, Radiation Oncology, Gainesville- FL, USA

**Purpose/Objectives**: Photon IMRT and proton (PT) PBS radiotherapy (RT) are often used in combined modality therapy for patients with Hodgkin (HL) and aggressive Non-Hodgkin Lymphoma (aNHL). While PBS offers improved OAR sparing, PT is a limited resource. As such, it is important to identify cases in which PT has the greatest impact in order to provide providers and patients with the best possible information with which to make decisions. Accordingly, the purpose of this study was to assess the difference in OAR sparing between photon IMRT and PT-PBS based on disease location using the Ann Arbor staging system.

**Methods and Materials**: A cohort was established including all patients with HL or aNHL treated using either photon or proton RT at a single institution from 2007-2020. The current analysis is limited to patients with mediastinal disease. Two plans (IMRT and PBS) were developed for each patient using consistent planning techniques to a dose of 30 Gy. OAR differences were assessed based on disease location using the Ann Arbor staging system. Lower mediastinal disease referred to disease below the aortic valve.

**Results**: The dosimetric analysis reveals substantial OAR sparing in PBS plans for the heart, left ventricle, LAD, lungs, and breast (female patients only). Statistical comparisons were performed with the Wilcoxon signed-rank test.

**Conclusions**: PBS planning results in significant sparing to OARs for patients with mediastinal lymphomas, and the utilization of the Ann Arbor staging system allows for improved decision making when determining which patients will benefit the most from PT.

### P 196 - Performance evaluation experiment of prompt gamma imaging and positron emission tomography system for dose distribution verification in carbon ion therapy

#### Bo-Wi Cheon^1^, Hyun-Joon Choi^2^, Chul Hee Min^1^

##### ^1^Yonsei University, Department of Radiation Convergence Engineering, Wonju, Korea Republic of ^2^Wonju Severance Christian Hospital, Department of Radiation Oncology, Wonju, Korea Republic of

We suggested the integrated PG-PET-based imaging system to increase the prediction accuracy for patient dose distribution in carbon ion therapy. The purpose of this study is to experimentally evaluate the performance of the PG-PET system. The system is consisted of dual-head detector modules, which parallel-hole collimators and scintillator arrays at each detector module, respectively. The detector module includes 16 × 16 arrays of GAGG crystals and 3 × 3 mm^2^ SiPM pixels. The tungsten collimator has 300-mm thickness, 1.5-mm slab, and 6.5-mm hole width. The customized SiPMs were paired to the scintillator of each detector module to read out the optical signal of a total of 256 channels. For evaluation of each detector module, ^137^Cs and ^22^Na test sources were positioned detector face to measure gamma counts. The mean energy resolutions measured at 511, 662, and 1,275 keV peak energy of each test source were 12.7 3.3%, 11.4% 1.1%, and 5.2% 2.2%, respectively. The coincidence events also were evaluated in the 511 keV peak regions, the time resolution for the coincidence events was less than 4 ns. The higher the energy, the better the energy resolution was evaluated. Regarding the sensitivity variations for every scintillator pixel, the standard deviations of peak counts were 12%. We demonstrated the feasibility of the PG-PET system for photon detection by applying our own radiation signal processing algorithm. In the near future, we plan to do the experiment evaluating the dose distribution with therapeutic carbon beam for proof of principle.

### P 197 - Preliminary dosimetric validation of a small animal irradiation box for preclinical irradiation research in clinical proton beam centres

#### Ileana Silvestre Patallo^1^, Reem Ahmad^2^, Richard Coos^3^, Mohammad Hussein^1^, Alexander Grimwood^4^, Stéphane Lucas^3^, Maria Hawkins^2^, Colin Baker^4^, Hugo Palmans^5,6^, Andrew Nisbet^2^

##### ^1^National Physical Laboratory NPL, Medical Radiation Physics, Teddington, United Kingdom ^2^University College London UCL, Medical Physics and Biomedical Engineering, London, United Kingdom ^3^University of Namur, Nuclear reaction analysis research unit LARN, Namur, Belgium ^4^University College London Hospital UCLH, Proton Beam Therapy, London, United Kingdom ^5^National Physical Laboratory, Medical Radiation Science, Teddington, United Kingdom ^6^MedAustron Ion Therapy Center, Medical Physics Group, Wien, Austria

Within the proton community there is an unmet need for facilitating preclinical research in clinical facilities to enable clinical radiation quality dependent response calculations. Scientists from Namur University have designed and built a positioning system for irradiating small animals for preclinical and translational research (Figure 1). The device can be placed on the treatment couch in centres without dedicated research rooms. The device has been approved by the Namur University Animal Welfare and Hygiene Committee for animal safety and comfort under European Union Standards. In this work, a dosimetric characterization of the device was performed and applicability of a clinical radiation workflow (CT, contouring, plan design and transfer, positioning, target alignment and irradiation) to accurately irradiate small targets (5mm diameter) using Tantalum collimators was investigated. The RayStation MC algorithm was used to calculate absorbed dose to water at the location of the alanine, in bespoke built modular holder and mouse phantom for a single-spot plan with three energy layers optimized to cover the alanine pellet. Measurements were performed at UCLH Proton Beam Therapy centre. Reference dose to water measurements with a secondary standard Roos chamber agreed within 0.7% with alanine measurements (100 MeV proton beam). On average, the difference between dose calculated by TPS and the dose determined using alanine inside the irradiation device (holder and mouse phantom) was -3.7% (Table 1). No beam quality correction factors were applied to the alanine measurements. Preliminary analysis has demonstrated that clinical workflows can facilitate accurate conformal dose deliveries to small, collimated targets.

### P 198 - Study of radiosensitization by gold nanoparticles using photon and proton beams

#### Esther Loscertales^1,2^, Rosalía López^3^, Juan antonio Vera^4^, Alejandro Mazal^4^, Luis Mario Fraile^1,2^, Jose Manuel Udías^1,2^, Ana Espinosa^3,5^, Samuel España^1,2,6^

##### ^1^Universidad Complutense de Madrid, Departamento de Estructura de la Materia- Física Térmica y Electrónica, Madrid, Spain ^2^Instituto de Investigación del Hospital Clínico San Carlos IdISSC, Técnicas e Instrumentación Nuclear en Biomedicina, Madrid, Spain ^3^IMDEA Nanociencia, IMDEA Nanociencia, Madrid, Spain ^4^Centro de Protonterapia Quironsalud, Centro de Protonterapia Quironsalud, Madrid, Spain ^5^Instituto de Ciencia de Materiales de Madrid- ICMM-CSIC, Instituto de Ciencia de Materiales de Madrid- ICMM-CSIC, Madrid, Spain ^6^Centro Nacional de Investigaciones Cardiovasculares CNIC, Centro Nacional de Investigaciones Cardiovasculares CNIC, Madrid, Spain

**Introduction**: Gold nanoparticles (GNPs) have been proposed as radiosensitizers. In this study, we characterized the enhancement in the production of reactive oxygen species (ROS) by GNP when irradiated with photon and proton beams.

**Methods**: GNP with sizes from 1.9 to 20 nm and coated with citrate and thiolated PEG were irradiated using photon (Cs-137 gamma irradiator) and proton (Quironsalud Madrid) beams with doses ranging from 1 to 20 Gy. Samples were prepared at different concentrations in water solutions including fluorescent probes for ROS detection (3-CCA and SOSG for OH· and 1O2 radicals, respectively). The enhancement of ROS production was quantified by the enhancement factor (EF) defined as the ratio between the results obtained for GNP and water samples.

**Results and Conclusion**: Results obtained under photon irradiation shows high EF of OH· production using citrate-coated GNPs which increases for smaller nanoparticles. On the contrary, PEG-coated GNPs provide negligible enhancements. Our results agree with previous studies available in the literature. For the first time, we measured EF for 1O2 using SOSG probe although only PEG-coated GNPs were characterized as the probe gets absorbed in the surface of citrate-coated GNP preventing the possibility to obtain reliable results. Very high 1O2 EF were obtained for low concentration of GNPs while scavenging effect of GNPs is observed at higher concentrations. Preliminary results using protons show higher OH· EF on citrate-coated GNPs compared to photons. Further studies are needed to complete the characterization of ROS production with protons including in vitro studies.

### P 199 - 3D tracks of ionizations and excitations in a polymer gel dosimeter irradiated with a 60 MeV proton SOBP

#### Marek Maryanski^1^, Jakub Czubek^1^, Marta Marszewska^1^, Sylwia Szczepańska^2^, Marta Cichacka^2^, Marcin Wekwejt^3^, Tomasz Kowalski^4^, Jan Swakoń^4^, Edyta Michaś-Majewska^5^, Michał Gryziński^5^

##### ^1^Faculty of Applied Physics and Mathematics/Gdansk University of Technology, Institute of Nanotechnology and Materials Engineering, Gdansk- Poland, Poland ^2^Faculty of Applied Physics and Mathematics/Gdansk University of Technology, MS Student, Gdansk- Poland, Poland ^3^Faculty of Mechanical Engineering and Ship Technology/Gdansk University of Technology, Department of Biomaterials Technology, Gdansk, Poland ^4^Polish Academy of Sciences, Institute of Nuclear Physics, Kraków, Poland ^5^National Centre for Nuclear Research, Radiological Metrology and Biomedical Physics Division, Otwock-Świerk, Poland

**Background**: Feasibility of 3D mapping of both dose and LET in polymer gel dosimeters (PGD) by laser CT has recently been demonstrated. Here correlation between turbidity spectra and the microscopic structure of radiation-induced polymer clusters in the gel will be presented and the technology implementation prospects discussed.

**Methods**: The PGD was made of gelatin, water, and methacrylic acid monomer, with added proprietary modifiers. Gels were sealed in NMR test tubes and irradiated in a water phantom to doses from 2 to 20 Gy with a modulated 60 MeV proton beam with 29 mm range and 40 mm diameter, at IFJ PAN. A spectrophotometer was modified by adding a primary-beam collimator, a variable aperture of the photodetector and a 3D-printed sample holder. Turbidity spectra of gels were taken between 350 nm and 1100 nm. Digital optical micrographs of gel samples were taken on a microscope camera (Olympus, Japan). Polymer cluster sizes were analyzed using Image J freeware.

**Results and Conclusions**: Micrographs reveal straight-line tracks of primary, secondary and scattered protons and randomly distributed clusters polymerized by delta rays. Clusters are approximately spherical and have a narrow size distribution centered around 1 micrometer. Average near-neighbor distance exceeds 3 radii such that Mie-Debye’s theory can be applied to interpret turbidity spectra at different dose and LET levels. Cluster sizes increase with LET, thus supporting recently published predictions and data. Depending on LET and the wavelength of light, optical extinction spectra followed the Rayleigh-Gans and van de Hulst anomalous diffraction models of light scattering on non-absorbing spheres.

### P 200 - Dosimetric comparison of proton and carbon ion beams for ocular melanoma

#### Michael Moyers^1^, Ying Xing^1^, Jiyao Sun^1^, Xue Ming^1^, Hongliang Chen^1^, Zhuangming Shen^1^, Jie Li^1^, Rongcheng Han^1^

##### ^1^SPHIC, Medical Physics, Pudong, China

Although the local control rate of ocular melanoma tumors using anteriorly applied proton portals is extremely high, decreasing the dose delivered to normal tissues might result in a reduction of complications. This study investigates the dosimetric differences between proton and carbon ion portals. Treatment plans were generated for both phantoms and patients using the TIMPS^®^ treatment planning system that uses Monte Carlo based track-repeating dose calculations. Single portal plans were devised for protons using an aperture, carbon ions without an aperture, and carbon ions with an aperture. All plans used the modulated scanning and energy stacking beam delivery techniques. Biologically weighted dose distributions for proton plans were calculated used a constant 1.1 factor while carbon ion plans used the microdosimetric kinetic model. At both the surface and a depth corresponding to 80% of the maximum range, the lateral penumbras were largest for carbon ion plans without aperture, intermediate size for protons with aperture, and smallest for carbon ions with aperture. Carbon ion plans using apertures are similar to proton plans using apertures but with slightly reduced dose to structures lateral to the portal edges and slightly increased dose distal to the target. Further comparisons with a larger variety of patients should be studied to fully demonstrate advantages and disadvantages of each type of plan.

### P 201 - Scalp dose can be reduced in whole brain CSI intensity modulated proton therapy by removing superficial Bragg peaks

#### Witold Matysiak^1^, Sammie Hedrick^2^, Marieke C. Landeweerd^1^, Agata Bannink^1^, Mart A.A.M. Heesters^1^, Hiske L. van der Weide^1^, Charlotte L. Brouwer^1^, Johannes A. Langendijk^1^, Stefan Both^1^, John H. Maduro^1^

##### ^1^University Medical Center Groningen, Raditherapy, Groningen, Netherlands ^2^Thompson Proton Center, Radiotherapy, Knoxville, USA

**Objectives**: Due to the physical properties of protons, skin sparing in proton pencil beam scanning therapy for whole brain is challenging. It is noteworthy that a significant scalp dose results from protons exiting the target and still having enough residual range to reach the skin. Their contribution can be reduced by 1) using multiple beams directions and/or 2) editing the Bragg peaks located in the skin distal to the target, hereafter termed “scripted spot removal”. Careful evaluation of both techniques is required to ensure adequate target coverage robustness.

**Methods**: Two coplanar field directions: 260^0^ and 100^0^ were employed in 2-field SFUD plans while an extra posterior field was added to the 3-field SFUD and IMPT techniques. For each plan type two optimization strategies were applied: with and without the scripted spot removal. Plans for eight patients (6 pediatric, 2 adult) consecutively treated at our institution with prescription doses of 36Gy in 20 fractions were retrospectively generated this way. All plans were robustly evaluated against 3 mm translations/3% range uncertainty, and 2° rotations.

**Results**: Robustness evaluation shows that for each patient and plan type the target is adequately covered under uncertainties corresponding with our position verification protocol.

**Conclusions**: Three field directions (IMPT or SFUD optimized) combined with scripted superficial spots removal can be effectively used in adult patients to incrementally reduce intermediate skin dose while maintaining robust target coverage.

### P 202 - Determination of planning target volume according to changes in bladder and rectal around the prostate in the treatment of proton

#### Sun Young Kim ^1^

##### ^1^National Cancer Center- Republic of Korea, Department of Proton Therapy Center, Goyang-si- Gyeonggi-do, Korea Republic of

**Objective**: The purpose of this study was to evaluate changes of bladder volume and rectum area through statistical method and estimate treatment planning margin to be considered in treatment plan based on results.

**Background**: Pre-treatments of filling the bladder and inserting a balloon into the rectum are being performed to increase treatment efficiency of prostate fixation and reduce adverse effects of adjacent normal organ treatment.

**Materials and Methods**: A total of 100 patients participated in this study. After urinating and oral intake of water was 500 cc. One hour later, the volume of bladder was measured using ultrasonography. A balloon was inserted into the rectum and 100 cc of contrast medium diluted with saline. It is to obtain accurate rectal volume through imaging. The rectum area was measured using PACS. The bladder volume and rectum area were compared to that in the proton therapy treatment plan. Bland-Altman Plot analysis was performed on 100 cases to assess the degree of matching with the treatment plan values.

**Results**: The bladder volume showed that the matching limit ranged from −359.50 to +402.28. The mean and standard deviation was 21.39 ± 194.33 cc. The Rectum Area showed that the matching limit ranged from −1.58 to +1.25. The mean and standard deviation was −0.16 ± 0.72 cm^2^.

**Conclusion**: Based on the results, a treatment planning margin of at least 3 mm is required to adequately treat 95% of intra-prostate movements.

### P 203 - Carbon-ion radiotherapy in pancreatic cancer treatment at HIT: Where do we stand in comparison with the Japanese clinical experience?

#### Filipa Baltazar^1^, Thomas Tessioner^1,2,3,4,5^, Benedikt Kopp^2^, Thomas Haberer^2^, Jürgen Debus^1,2,3,4,5^, Jakob Liermann^2,6,7^, Andrea Mairani^1,2,4,5,8^

##### ^1^German Cancer Research Center DKFZ, Clinical Cooperation Unit Radiation Oncology, Heidelberg, Germany ^2^Heidelberg Ion-Beam Therapy Center HIT, Department of Radiation Oncology, Heidelberg, Germany ^3^German Cancer Consortium DKTK Core-Center Heidelberg, Clinical Cooperation Unit Radiation Oncology, Heidelberg, Germany ^4^Heidelberg University Hospital UKHD, Clinical Cooperation Unit Translational Radiation Oncology, Heidelberg, Germany ^5^National Center for Tumor Diseases NCT, Clinical Cooperation Unit Translational Radiation Oncology, Heidelberg, Germany ^6^Heidelberg University Hospital UKHD, Department of Radiation Oncology, Heidelberg, Germany ^7^Heidelberg Institute of Radiation Oncology HIRO, Department of Radiation Oncology, Heidelberg, Germany ^8^National Centre of Oncological Hadrontherapy CNAO, Medical Physics, Pavia, Italy

**Purpose**: The first European prospective phase II clinical trial investigating Carbon Ion Radiotherapy Treatment (CIRT) for pancreatic cancer treatment is taking place at the Heidelberg Ion Therapy Center (HIT). This work analyzes the dose constraints applied in the PACK-trial and compares it to the Japanese experience. Furthermore, the LETd distribution for all patients is evaluated and correlated with Local Control (LC).

**Methods**: The absorbed and RBE-weighted doses of 14 patients from the PACK cohort, as well as the LETd, were recalculated. For the RBE calculation, LEM-I and adapted MKM models were considered. By correlating both recomputed RBE-weighted distributions, a constraint to the gastrointestinal (GI) tract for plan optimization was derived from the Japanese clinical experience (NIRS).

**Results**: The results suggested that a more relaxed constraint to the GI tract may be considered without leading to increased toxicity. Furthermore, considering such NIRS-derived dose constraint for treatment plan optimization allows improvement of the tumor coverage, as shown in Figure 1. Regarding LETd, Figure 2 shows that the cohort’s LETd_98%_ in the GTV was lower than the minimum LETd which had been related to a better LC in 2019 by Hagiwara et al.

**Conclusion**: A lower LETd was observed in the GTV compared to the Japanese experience, due to differences in the beam configuration. However, the higher RBE-weighted dose in the tumor may be linked to the preservation of the tumor LC observed within the PACK-cohort to this date. Future analyses of other clinical endpoints are foreseen alongside the progression of the PACK-trial.

### P 204 - Field numbers and beam angles selection in Breath-Hold Intensity Modulated Proton Therapy (BH-IMPT) for Liver Stereotactic Body Proton Therapy (SBPT)

#### Mintra Keawsamur^1^, Kanokphorn Thonglert^1^, Petch Alisanant^1^, Napapat Amornwichet^1^, Chonlakiet Khorprasert^1^

##### ^1^HRH Princess Maha Chakri Sirindhorn proton center- King Chulalongkorn Memorial Hospital- Thai red cross society, Faculty of medicine- Chulalongkorn University, Bangkok, Thailand

The field numbers and beam angles selection are the specific requirements that accounted for the target coverage and organs at risk (OARs) sparing. This research aims to compare the dosimetric performance of Breath-Hold Intensity Modulated Proton Therapy (BH-IMPT) plans generated with eight plans of the different number of field and beam angle directions in various tumour locations for Liver SBRT patient. The plans involved one field, two fields with an angular separation of 30, 45, 60, 90 and 180-degree between beams, and three fields with an angular separation of 50 and 90-degree between beams. The robust optimization was implemented. The best plan was selected concerning conformity, mean dose to healthy liver, skin, ribs, and other OARs. For the tumour located in the peripheral area, the dose distribution of two fields with 60-degree separation was suggested since it provides the most sparing dose. For the two fields with an angular separation of 60-degree between beams are the best for sparing the normal liver, skin, and ribs when the tumour is in a middle area. The central tumour is often surrounded by the normal liver. A limited number of fields achieved low mean liver dose while maintaining the conformal dose to the GTV. The 0 and 180-degree beams produced the least mean liver dose. This research guided appropriate beam directions and angles selection for BH-IMPT for Liver SBPT concerning conformity and the least dose to liver, skin, and OARs.

### P 205 - Those fields could be delivered faster: RapidScan optimization of IMPT plans

#### Roni Hytonen^1^, Sara Rosas^1^, Pierre Lansonneur^1^, Roberto Cassetta^1^

##### ^1^Varian Medical Systems Finland, Proton Planning, Helsinki, Finland

**Background**: When optimizing radiotherapy plans, the aim is to cover the target with the prescribed dose while keeping the dose to healthy tissue as low as possible. The field-wise delivery time (DT_F_) is often of secondary concern, and is not directly considered during the plan optimization process, even though reducing the DT_F_ might prove beneficial e.g. in cases which require deep inspiration breath hold, or where the intrafraction motion becomes non-negligible due to large field sizes.

**Materials and Methods**: We have developed a new “RapidScan optimizer” that makes it possible to evaluate and control DT_F_ during the optimization, allowing the dosimetrist to explore the trade-offs between the dosimetric objectives and delivery time. We tested the new optimizer with four clinical plans (2 lung, 1 base of skull, and 1 liver). For each case, plans were optimized using the conventional NUPO 18.0 optimizer (Varian Medical Systems) and the RapidScan optimizer. Both plans were based on the same dosimetric objectives. The RapidScan optimizer was used to minimize DT_F_ while maintaining target coverage and plan robustness comparable to that of conventionally optimized plans.

**Results and Conclusion**: We were able to reduce the field-wise delivery times by 29–51% (mean reduction of 38±8%) while maintaining target homogeneity within ±0.5% (Figure 1). Our results indicate that many of the IMPT fields could potentially be delivered significantly faster without sacrificing target coverage or robustness by considering the delivery speed during the optimization.

### P 206 - Multi-field optimization for FLASH intensity modulated proton therapy using Bragg peaks

#### Pierre Lansonneur^1^, Miriam Krieger^2^, Anthony Magliari^3^, Michael Folkerts^3^

##### ^1^Varian Medical Systems, FLASH Planning, Le Plessis-Robinson, France ^2^Varian Medical Systems, FLASH program, Troisdorf, Germany ^3^Varian Medical Systems, FLASH program, Palo Alto, USA

**Background.** Currently FLASH Intensity Modulated Proton Therapy (FLASH-IMPT) has been shown possible using patient-specific 3D range modulators. However, state-of-the-art examples only rely on generating uniform Spread-Out Bragg Peaks (SOBP) to ensure the PTV coverage.

**Aims.** To improve both the quality and dose rates of FLASH-IMPT plans, a new Multi-Field Optimization (MFO) method was developed.

**Methods.** FLASH-IMPT plans were created for peripheral lung and brain metastasis cases. Guided by SBRT RTOG protocol [1], each lung plan was prescribed to deliver 34 Gy (respectively 18 Gy for brain metastasis following SRS RTOG protocol [2]) in one fraction to the PTV using 2 to 3 fields. The range modulator design and the spot list were optimized simultaneously using MFO. To ensure a high dose-rate, a minimum MU per spot was enforced to ensure an ultra-high dose rate delivery. Several dose metrics were evaluated and compared with and without minimum MU enforcement. The PBS dose rate distributions [3] were then calculated and compared for each plan.

**Results.** The fraction of irradiated volume (dose above 2 Gy) receiving at least 40 Gy/s reached up to 80% for Lungs (92% for the brain) for plans optimized with the minimum MU constraint (0% without minimum MU constraint). Each plan passed the RTOG protocol, and no differences were observed on the dose-volume histograms of plans optimized with and without minimum MU enforcement.

**Conclusion**. This optimization method will support the creation of high-quality plans for FLASH-IMPT.

### P 207 - Semiautomatic determination of tantalum clip locations on ocular MR-Images

#### Corné Haasjes^1,2^, Lisa Klaassen^1,2,3^, Khanh Vu^1^, Coen Rasch^3,4^, Jan-Willem Beenakker^1,2,3^

##### ^1^Leiden University Medical Center, Department of Ophthalmology, Leiden, Netherlands ^2^Leiden University Medical Center, Department of Radiology, Leiden, Netherlands ^3^Leiden University Medical Center, Department of Radiation Oncology, Leiden, Netherlands ^4^Holland Proton Therapy Center, n/a, Delft, Netherlands

**Introduction**: MRI is increasingly used in ocular oncology. The locations of surgically placed tantalum clips form an important input for ocular proton therapy. In this study, a method was developed and evaluated to semi-automatically determine the clip locations on ocular MR images.

**Methods**: MR-images were acquired using a 0.8mm isotropic 3DT1-weighted sequence [Jaarsma, Ophthalmol Retina 2022]. After an initial annotation by the user, the extent of the signal void was determined using the vitreous as a reference. Subsequently the clip center was obtained using a gaussian-smoothed distance-transform (Figure 1). The interobserver variability was assessed in 5 patients (20 clips). For 17 patients (68 clips) the clip-clip distances were compared to X-ray measurements.

**Results**: The interobserver difference was 0.15±0.21mm (mean±SD). 3/17 patients were excluded due to poor image quality. On average MRI showed 0.35mm (IQR = 0.01 – 0.77mm) smaller clip-clip distances than X-rays (Figure 2). Larger differences were observed for clips whose signal void overlapped with another clip (0.60mm vs 0.28mm). Overall, the method appears to have a systematic bias towards the sclera, which can be isointense to the clip-induced signal void (Figure 1B), likely causing the smaller clip-clip distances on MRI.

**Conclusion**: The developed pipeline can be used to localize clip centers with sub voxel-size accuracy and interobserver variability, but manual validation is required as signal voids from neighboring clips can confound the method.

### P 208 - The feasibility assessment of using daily two portals to replace traditional three portals in nasopharyngeal carcinoma

#### Hsuan Ting Wang^1^, Hsiao Chieh Huang^1^, Joseph Tung Chieh Chang^1^

##### ^1^Proton and Radiation Therapy Center, Linkou Chang Gung Memorial Hospital, Taoyuan City, Taiwan Province of China

**Purpose**: Nasopharyngeal carcinoma (NPC) is one of a rare head and neck cancer occurs in Asian. But the cost of proton therapy is tending to be a loading for the NPC patient. The feasibility of using daily two portals, besides one PA (posterior-anterior) beam, a left posterior oblique (LPO) or a right posterior oblique (RPO) beam, to replace traditional three portals for the purpose of reducing the treatment time and cost were considered to be a viable approach.

**Methods and Materials**: Ten patients with NPC in different stages were selected in the retrospective study. Three plans were made for all patients, including plan angle using LPO with PA, RPO with PA and LPO with RPO with PA. Two plans containing LPO with PA and RPO with PA were summed as a complete treatment planning. The summation treatment plans were used to compare with the traditional three portals treatment plan via dosimetric comparison.

**Results**: Evaluation of quality of treatment plans, including conformal index, homogeneity index, show no significant difference in both plans. However, R50 (ratio of 50% of prescription dose volume to the volume of clinical target volume), volume of receiving 3000cGy in brainstem and spinal cord show disadvantage in summation plan, but no difference in maximum dose.

**Conclusions**: Most dosimetric parameters of two kinds of treatment plan shows no obvious difference, but the intermediate to low dose region still be a benefit in three portals plan. The uncertainty of two portals plans also need to be concern in daily treatment.

### P 209 - The proton dosimetric comparison of using three and five portals in head and neck patients

#### Heng Ching Chang^1^, Hsiao Chieh Huang^1^, Joseph Tung Chieh Chang^1^

##### ^1^Chang-Gung Memorial Hospital, Department of Radiation Oncology, Taoyuan, Taiwan Province of China

**Purpose**: In proton therapy, two to three portals are usually used to treat tumors. For head and neck cancer, two posterior oblique (PO) beams or anterior oblique (AO) beams with one posterior-anterior (PA) are used clinically. In this study, traditional three portals plans PA with PO were compared to the five portals treatment plans, which combine daily PO with PA and AO with PA separately.

**Material and Methods**: Ten (10) nasopharyngeal carcinoma (NPC) patients were selected. The dose constraint of nearby organ at risk (OAR) including brain stem and spinal cord will be the first priority in the optimization process under two treatment scenario: (1) three portals with PO and PA (2) five portals plans which combined daily three portals PO with PA and AO with PA. The dosimetric results will be evaluated including OAR, R50 (ratio of 50% of prescription volume to the volume of CTV) and homogeneity index (HI).

**Results and Discussion**: HI and R50 of five portals plans shows significant improvement than the PO with PA plans in the statistical results. Maximum dose of brainstem and spinal cord also show advantage in five portal treatment plans. But mean dose of parotid gland is an obvious limitation in five portals plans.

**Conclusion**: Compared to three portal plans, five portal treatment plans offer better homogeneity and conformity, but cause parotid glands to receive more dose. In addition, the treatment time and cost also need to be considered.

### P 210 - Mapping proton stopping power: The influence of imaging parameters on proton range

#### Torbjörn Näsmark^1^, Jonas Andersson^1^

##### ^1^Umeå University, Department of Radiation Sciences- Radiation physics, Umeå, Sweden

**Purpose**: To evaluate the influence of imaging parameters for three DECT-based methods for mapping proton stopping power (SP): Näsmark and Andersson (N&A), Landry-Saito (L-S), and Siemens Healthineers application DirectSPR. The N&A method uses virtual monoenergetic images (VMI) as input, while L-S and DirectSPR use native kilovoltage images from a dual-source DECT scanner.

**Material and Methods**: Image data of a CIRS-062M phantom was acquired with two DECT scanners (GE Healthcare and Siemens Healthineers) using different dose levels, reconstruction kernels, and levels of iterative or deep learning-based noise reduction algorithms. All methods were compatible with the Siemens scanner, but only N&A was compatible with the GE scanner. A treatment planning system (RayStation, RaySearch Laboratories) was used to investigate the impact on proton range with different scan settings and methods to estimate proton SP.

**Results**: In general, all methods scored similar results and were mostly found to be robust to changes in imaging parameters. They all score well for soft tissue, while N&A struggles with inhaled lung, and L-S struggles with dense bone. For N&A, results were in general similar on both scanners, but significant differences were observed for lung tissue and bone in some cases. The L-S method displayed some issues with image noise, resulting in non-physical effective atomic numbers.

**Conclusions**: All methods demonstrated their potential to improve on the conventional method for estimating proton SP. The N&A method is compatible with any scanner capable of generating VMIs, e.g., all commercial DECT scanners, as well as emerging photon counting CT scanners.

### P 211 - Correction method for optical scaling of fundoscopy images for ocular proton beam therapy planning

#### Lennart Pors^1^, Noor P. Hoes^2^, T.H. Khanh Vu^2^, Nanda Horeweg^1^, Jan-Willem M. Beenakker^1,2,3^

##### ^1^Leiden University Medical Center, Radiation Oncology, Leiden, Netherlands ^2^Leiden University Medical Center, Ophthalmology, Leiden, Netherlands ^3^Leiden University Medical Center, Radiology, Leiden, Netherlands

**Objective**: In ocular proton therapy (OPT) planning, optical images of the fundus are used to define the tumor base and, in some centers, the location of the fovea and optic nerve. The scaling of these images, however, depends on the eye’s optics, which differs between subjects. In this study, we developed a method to correct for this scaling difference and assessed its impact in uveal melanoma patients.

**Methods**: A paraxial optical model of the eye and fundus camera (fig1A) was developed using ray-transfer matrix formalism, which can be personalized based on the patient’s biometry (e.g. corneal curvature, lens location and eye length). The model was validated using an in-house-developed optical eye phantom (21 different setups; refractive errors range -8D to +4D). The impact of ocular anatomy on image magnification was assessed and compared to the conventional method using a generic eye model (ISO 10940:2009). The method was evaluated in 18 OPT-patients.

**Results**: In the eye phantom, the model accurately predicted the magnification (absolute difference IQR 0.17%-1.05%, fig1A). Corneal curvature and eye length have the largest impact on magnification (up to 160% compared to average eye, p<0.001, fig1B). In patients, magnification differences between 0.95-1.23 were observed (fig 2A), corresponding to -0.26 to 0.80mm changes in macula–optic nerve distances (fig2B).

**Conclusion**: Ocular anatomy has significant effect on the scaling of fundus images, which can be accurately corrected by our model.

### P 212 - Robust margin reduction for lung cancer patients with large tumor movement

#### Vicki Taasti^1^, Anouk Vullings^1^, Janou Buck^1^, Maud van den Bosch^1^, Lieke in ’t Ven^1^, Stephanie Peeters^1^, Mirko Unipan^1^, Ilaria Rinaldi^1^

##### ^1^Department of Radiation Oncology MAASTRO, GROW – School for Oncology- Maastricht University Medical Centre+, Maastricht, Netherlands

**Background**: Until now, we have treated 83 lung cancer patients with a tumor movement >5mm with proton therapy using an internal target volume (ITV) approach where the ITV was expanded by 2mm in the robust optimization and evaluation. With this study we evaluated if the target margin could be reduced without compromising target coverage and plan robustness.

**Methods**: For 16 patients, clinically treated with an ITV expansion of 2mm (ITV_2mm), new plans were made with a 1mm expansion (ITV_1mm). All plan and optimization parameters were kept the same for the two plans, and robust optimization settings were 5mm setup and 3% range uncertainty for both. The treatment schedules included 25 or 30 fractions. The ITV_2mm- and ITV_1mm-plans were robustly re-evaluated on all weekly repeat-CTs, to analyze if the ITV_1mm-plans were equally robust to anatomical changes as the ITV_2mm-plans (Figure 1). The target coverage was evaluated, to assess if the dose constraints were still met on the repeat-CTs. Moreover, the organ-at-risk dose was evaluated.

**Results**: With the ITV_1mm-plan, all patients had a reduction in the mean dose to the heart. On average, the mean dose to the heart and the esophagus decreased by 0.4 Gy and 0.3 Gy, respectively. Only 2 patients needed an extra plan adaptation with the ITV_1mm-plan on a repeat-CT where no plan adaptation was needed with the ITV_2mm-plan (Figure 2).

**Conclusion**: The ITV_1mm-plans did not lead to considerably more plan adaptation than the ITV_2mm-plans. These results have led to the clinical introduction of the ITV_1mm-plans.

### P 213 - Examination of maximum initial proton energy for Stereotactic Ablative Body Radiotherapy (SABR) In prostate cancer

#### Chris Beltran^1^, Alvaro Perales^1^, Keith Furutani^1^

##### ^1^Mayo Clinic, Radiation Oncology, Jacksonville, USA

Intensity modulated proton beam (IMPT) has become a valid option to treat prostate cancer. However, the energy requisites needed to reach the tumour in depth has compromised its viability in terms of cost effectiveness. The purpose of this work is to show the goodness of treatment plans with different initial maximum energies. We have evaluated planning treatment volume (PTV) coverage and homogeneity as well as dose in organs at risk (OARs). Four plans were developed with maximum energies of 160, 180, 200 and 230 MeV respectively. 18 beams with an angle separation of 20 degrees covered the patient (Figure 1a). The dose prescription was 36.25 Gy over 5 fractions. Multifield optimization (MFO) using Eclipse treatment planning system (TPS) was employed to meet our dosimetric goals. Table 1 summarizes the dosimetric parameters assessed in our study. Dose-volume histograms (DVHs) comparison (Figure 1b) shows the plan with initial maximum energy of 160 MeV produces a dosimetric solution similar to the plan with an initial maximum energy of 230 MeV. We have demonstrated that a lower initial maximum energy of 160 MeV ensures a high tumor conformality keeping lower toxicities in OARs for prostate SABR.

### P 214 - Protontherapy for superficial tumors: Validation of the TPS dose calculation and comparison to high energy photon techniques

#### Alain Batalla^1^, Laurine Bonnor^1^, Pauline Dutheil^1^, Dorothée Lebhertz^1^, Laetitia Lechippey^1^, Marie Lecornu^2^, Cédric Loiseau^1^, Cyril Moignier^1^, Dinu Stefan^2^, Anthony Vela^1^

##### ^1^Centre François Baclesse, Medical physics department, Caen, France ^2^Centre François Baclesse, Radiotherapy department, Caen, France

Usually superficial tumors are treated with electron beams, low energy photon beams or high energy photon beams using bolus. In this work, we demonstrate the feasibility of superficial irradiation with a Proteus One. This latter allows intensity modulated proton therapy with a pencil beam scanning technique. Unlike photon with buildup effect at entrance, protons are particles capable of depositing dose at entrance surface. At nozzle exit, lowest energy available is 98.4 MeV, which correspond to a range of 7.5 cm in water, but a range shifter is used to place Bragg peaks at surface. Two complex superficial tumor locations that are nose and ear have been calculated and treated with protons. Dose measurements have been performed in order to validate the TPS dose calculation. Moreover, two conventional photon plans using thermoformed and 3D printed bolus have been calculated for comparison with proton plans. Results of these two clinical cases and the validation of the TPS dose calculation are presented. The advantages of protontherapy for superficial tumors are discussed. In particular, it is not possible to treat superficial tumors with high energy photon beams without a bolus. Whereas it is interesting for some indications to avoid bolus, because they are not always easy to manage: it must be in contact on the skin over the entire surface and positioning as to be the same between simulation and all treatment fractions. In conclusion, protontherapy is relevant to reduce dose delivery uncertainties for superficial tumors.

### P 215 - Evaluation of robustness and organ sparing potential of proton therapy for anal cancer

#### Kelvin Ng Wei Siang^1^, Maaike J. Berveling^1^, Witold Matysiak^1^, Jannet C. Beukema^1^, Johannes A. Langendijk^1^, Stefan Both^1^, Charlotte L. Brouwer^1^

##### ^1^University Medical Center Groningen, Department of Radiation Oncology, Groningen, Netherlands

**Purpose**: To evaluate the target coverage robustness and potential of intensity modulated proton therapy (IMPT) to spare organs at risk (OARs) against volumetric modulated arc therapy (VMAT) for anal cancer.

**Methods**: Seven anal cancer patients treated in our institution were included in the study. We considered a simultaneous integrated boost scheme of 25 fractions; 59.4Gy and 45.0Gy to the primary and elective targets; respectively. Two-arc VMAT and robustly optimized two field IMPT at gantry 0° and 180^°^ (Fig. 1) plans were evaluated against 5mm set-up and 3% range (for IMPT plans) uncertainties. The opposing IMPT fields only overlapped at a small junction zone (1cm) at the anterior CTVs. Target coverage (nominal and voxel-wise minimum) and OARs (nominal and voxel-wise maximum) doses were compared.

**Results**: Nominal dose and D2 of the target were acceptable (D98>95%, D2<107%). The voxel-wise minimum clinical target volume (CTV) coverage was overall adequate for both IMPT (D98 = 94.1%, [93.8–94.5%]) and VMAT (D98 = 93.3%, [90.7-94.8%]) techniques. The nominal mean OAR doses are shown in Fig. 2 with associated voxel-wise maximum dosimetric parameters (RTOG0529) in the corresponding table. The OARs doses were generally much lower for the IMPT plans compared to VMAT plans. Major differences were observed for the mean OARs doses and e.g., bone marrow V20: 30.3% [21.6-35.2%] for IMPT vs. 50.6% [39.5-56.6%] for VMAT; bowel bag V30: 318.2cm^3^ [140.5-511.4cm^3^] for IMPT vs. 419.2cm^3^ [197.4-712.5 cm^3^] for VMAT.

**Conclusions**: For anal cancer, robustly optimized IMPT offers robust target coverage and significant OAR sparring compared with VMAT.

### P 216 - Linear energy transfer of IMPT brain plans produced for different beamlines

#### Domenic Sievert^1^, Tianyu Zhao^1^, Tiezhi Zhang^1^, Stephanie Perkins^1^, Shahed Badiyan^1^, Thomas Mazur^1^

##### ^1^Washington University School of Medicine in St. Louis, Radiation Oncology, St. Louis, USA

IMPT plans were created to investigate differences in linear energy transfer (LET) in intensity-modulated proton therapy (IMPT) brain treatment plans produced by two proton platforms, P1 and P2, for 20 patients. P1 is synchrocyclotron-based and modulates energy via range-shifter plates, while P2 is cyclotron-based and incorporates slit-based energy selection with an option for mounting a range-shifter. Identical beam geometries and optimization objectives were used for both models, and P2 plans were created 1) without and 2) with range-shifter (P2RS). Dose and LET were calculated via Monte Carlo simulation with 2% statistical uncertainty. Mean dose-weighted LET (LETd) and LETd2% were calculated for each target volume and in adjacent 5 mm rinds extending 15 mm distally from the target in brain tissue (R1, R2, R3). Tests of significant differences in distributions between P1 and P2/P2RS were performed via two-sided Wilcoxon rank-sum tests. Significant differences for mean LETd were observed between P1 and P2 within R2 (p=0.004), whereas LETd2% significant differences were identified between P1 and P2 within R1 (p=0.012), R2 (p=0.0003), and R3 (p=0.0003). No significant differences were observed between P1 and P2RS. Median (inter-quartile range) LETd2% values in keV/μm in each rind were R1) 5.27(1.00) and 5.34(0.96), R2) 6.63(1.57) and 7.19(1.45) and R3) 7.48(1.74) and 8.31(1.57) for P1 and P2, respectively. Ongoing work is investigating variation in RBE-weighted dose between beamlines and treatment planning strategies to both 1) control LETd variation on each proton platform and 2) reduce hot spots in RBE-weighted dose.

### P 217 - Dosimetric comparison of two phase versus simultaneous integrated boost approaches for proton therapy of skull base chordoma and chondrosarcoma

#### Gillian Whitfield^1,2^, Peter Sitch^1^, Shermaine Pan^1^, Matthew Clarke^1^, Matthew Lowe^1,2^, Frances Charlwood^1^, Rovel Colaco^1^, Neil Burnet^1^

##### ^1^The Christie NHS Foundation Trust, Proton therapy, Manchester, United Kingdom ^2^University of Manchester, Manchester Academic Health Science Centre- The Christie NHS Foundation Trust, Manchester, United Kingdom

**Background and Aim**: To assess whether a Simultaneous Integrated Boost (SIB) technique improved dose to high risk target volumes compared to a 2-phase approach for skull base chordomas and chondrosarcomas.

**Methods**: For five patients, a clinical plan was produced with each technique. 2-phase plans prescribed 54Gy/30# to CTV_Low, then 19.8Gy/11# (chordoma) or 16.2Gy/9# (chondrosarcoma) to a smaller CTV_High. SIB plans prescribed 73.8Gy to CTV_High concurrent with 59.45Gy to CTV_Low in 41# for chordoma, and 70.2Gy to CTV_High with 58.5Gy to CTV_Low in 39# for chondrosarcoma; this provides at least equivalent BED for tumour alpha/beta of 2-10Gy for both dose levels. Both approaches limited brainstem D2%≤63Gy, D2% of a 2mm diameter brainstem “core” to 54Gy, and chiasm and each optic nerve to D2%≤60Gy and Dmean≤54Gy. For SIB plans, due to steep dose gradients, limits were applied to optic structures+1mm. For CTV_High and residual gross tumour we compared minimum and generalised equivalent uniform dose (gEUD), with an ‘a’ value of -15; both have been shown to correlate with local control. Additionally, mean target dose was compared along with D2% for optic structures, and chiasm Dmean.

**Results**: Comparison of both techniques is shown in Table 1 and Figure 1.

**Conclusions**: The SIB technique generally permitted higher target doses, with small estimated increases in tumour control probability (chordoma), matching clinical experience, especially near optics. It offers advantages in planning efficiency and uncertainty evaluation over the entire treatment course. By spreading dose to critical structures evenly over all fractions, injury risk should be lower.

### P 218 - Re-irradiation of rectal cancers and the ReRad II study: The importance of EQD2 correction for a meaningful dose accumulation

#### Heidi S. Rønde^1^, Jesper F. Kallehauge^1^, Christina G. Truelsen^1^, Karen-Lise G. Spindler^2^, Laurids Ø. Poulsen^3^, Birgitte M. Havelund^4^, Lene H. Iversen^5^, Bodil G. Pedersen^6^, Camilla J.S. Kronborg^1^

##### ^1^Aarhus University Hospital, Danish Centre for Particle Therapy, Aarhus, Denmark ^2^Aarhus University Hospital, Department of Experimental Clinical Oncology, Aarhus, Denmark ^3^Aalborg University Hospital, Department of Oncology, Aalborg, Denmark ^4^Vejle Hospital- University Hospital of Southern Denmark, Department of Oncology, Vejle, Denmark ^5^Aarhus University Hospital, Department of Surgery, Aarhus, Denmark ^6^Aarhus University Hospital, Department of Radiology, Aarhus, Denmark

**Purpose**: Proton therapy is highly eligible for re-irradiation – but correct dose summation is important for radiobiological evaluation of accumulated doses to Organs at Risk (OAR). Historically physical dose summation has been used, but with new software solutions it is possible to perform voxel-by-voxel fractionation correction and avoid both unintended overdosage to OAR or unwanted target coverage compromises. The aim was to determine the difference between corrected and un-corrected dose summation.

**Materials and Methods**: The initial twelve patients included in the ReRad II trial (dose escalated proton therapy re-irradiation for rectal cancer recurrences) were included. All treated with accelerated, hyperfractionated Intensity Modulated Proton Therapy (IMPT) 55-65Gy, (table 1). Re-irradiation IMPT plans were optimised in Eclipse (Varian Medical Systems). Both previous and re-irradiation plans were voxel-by-voxel Equivalent Dose in 2Gy Fractions (EQD2) corrected in Velocity (Varian Medical Systems). Subsequently both plans were accumulated in Eclipse as physical dose and EQD2-corrected dose, respectively. For the voxel-by-voxel EQD2-correction we used an α/β=3. We used rigid 6D-registration between previous and re-irradiation CTs, focusing on dose overlap regions.

**Results**: Difference (physical–EQD2) in accumulated bladder dose ranged from −16.5 Gy to 11.8 Gy for max and −5.5 Gy to 7.7 Gy for mean. Difference for bowel loops; max: −5.8 Gy to −11.8 Gy and mean: −5.7 to 14.0 Gy. See Figure 1.

**Conclusion**: We found large, patient dependent, deviations between physical and EQD2-corrected dose summation. Differences, if not corrected, could result in both unintended OAR dosage or unnecessary target compromises. This underlines the importance for 3-dimensional fractionation correction before dose summation.

### P 219 - Critical appraisal of paediatric embryonal cancers treated with image-guided intensity modulated proton therapy

#### Dayananda Shamurailatpam Sharma^1^, Noufal Mandala Padanthaiyil^1^, Ganapathy Krishnan^1^, Manikandan Arjunan^1^, Ashok Reddy^2^, Suhail Mahammood^1^, Sanjib Gayen^1^, Srinivas Chilukuri^2^, Rakesh Jalali^2^

##### ^1^Apollo Proton Cancer Center, Medical Physics, Chennai, India ^2^Apollo Proton Cancer Center, Radiation Oncology, Chennai, India

**Aim**: To undertake a comprehensive analysis of image-guided intensity modulated proton treatment (IG-IMPT) for craniospinal radiation (CSI).

**Materials and Methods**: An IG-IMPT database of 45 consecutive paediatric patients with CNS embryonal malignancies treated between January’2019 and April’2022 were critically appraised for demography, diagnosis, treatment planning strategy, and treatment delivery accuracy.

**Results**: Medulloblastoma(56%) predominated among the patients (median age, 7.5years; male:female=34:11), followed by recurrent ependymoma(19%), pinealoblastoma(5%), germ cell(5%), and others(15%). The median dose to CS and boost combined was 54GyRBE, while dose to PTV-CS (length:39.06-79.59cm) ranges from 21-35GyRBE. Median V95% to cribriform plate and optic nerves were 100% and 82.96%, keeping lens Dmax <3.9GyRBE. In skeletally immature patients(88.38%), anterior vertebral body (VB) was completely covered in 18.18% and under-dosed in 70.15%, with median Dmean of 10.11GyRBE to oesophagus. Lateral spine coverage was maintained on the edges of VB in 52.2% while it extends beyond in 48.8%. The median V98% for CTVs and V95% for PTVs of brain, spine, craniospinal were >97%, with an excellent conformity(0.89) and homogeneity(0.07) index for PTV-CS. All neurological OARs received median Dmax of 36-44GyRBE from combined CSI and boost regimens. Patient specific quality assurance results revealed that 545 (97.67%) planar dosage verification had gamma (3%@3mm) values >95%. The online patient set-up verification showed systematic and random error within 0.90mm and 1.71mm in translation, and 0.1° and 0.2° in rotation.

**Conclusion**: Change of practice pattern observed. The findings from the study add to the growing library of CSI practice and may serve as a reference for inter-institutional comparison.

### P 220 - Planning IOeRT FLASH treatments with a GPU-based Monte Carlo: The case of breast cancer

#### Gaia Franciosini^1,2^, Angelica De Gregorio^1,2^, Veronica De Liso^3^, Giuseppe Felici^3^, Annalisa Muscato^2,4^, Jake Pensavalle^3^, Alessio Sarti^2,5^, Angelo Schiavi^5^, Antonio Trigilio^1,2^, Vincenzo Patera^5^

##### ^1^University of Rome- Sapienza, Physics, Rome, Italy ^2^INFN, Section of Rome I, Rome, Italy ^3^S.I.T., Sordina IORT Technologies Spa, Aprilia, Italy ^4^Scuola post-laurea in Fisica Medica- University of Rome Sapienza, Scienze e biotecnologie- medico-chirurgiche, Rome, Italy ^5^University of Rome- Sapienza, Scienze di base e applicate per l’ingegneria, Rome, Italy

IOeRT is, as of today, one of the possibile testing ground for the FLASH effect evaluation. Even if breast cancer is widely treated using such technique with satisfactory results, one of its main limitations is a suboptimal irradiation of the target volume due to the absence of a dedicated treatment planning system. Aim of this contribution is to show how it is possible to, for the first time ever, plan and optimize an IOeRT treatment delivered at conventional and FLASH regime, using a fast GPU-based MC tool (FRED) and a new 3D real-time ultrasound intra-operative imaging acquisition, developed by the SIT company. The planning of an IOeRT breast cancer treatment has been studied using a real CT input of an anthropomorphic phantom with a silicone prosthetic breast application to mimic an ultrasound input. Once the input was properly setup, different treatment configurations, i.e. beam energy, protective disk and applicator size and position, have been explored. The Dose Volume Histograms have been obtained from the dose distributions computed by FRED for each treatment conditions and compared, allowing the selection of the best treatment configuration. The obtained results are promising, confirming that the developed tool is able to plan and optimize an IOeRT plan in few minutes, once the imaging is acquired, using a single GPU card and with an high level of accuracy. The impact of the FLASH effect has been also included, optimizing the biological dose, obtaining encouraging results in terms of superficial tissues sparing.

### P 221 - Automated robust optimization for IMPT: Introducing and comparing different approaches

#### Joana Dias^1^, Humberto Rocha^2^, Joana Neves^3^, Brígida Ferreira^4^

##### ^1^University of Coimbra, INESC Coimbra, Coimbra, Portugal ^2^University of Coimbra, Faculty of Economics, Coimbra, Portugal ^3^University of Coimbra, University of Coimbra, Coimbra, Portugal ^4^University of Lisbon, University of Lisbon, Lisbon, Portugal

Intensity-modulated proton therapy (IMPT) is a radiotherapy treatment modality that has proven to be able to properly irradiate tumors while preserving as much as possible normal tissues. However, it is extremely vulnerable to several different sources of uncertainties, which motivates the use of robust treatment planning optimization. We describe a new approach for robust automated IMPT treatment optimization, considering both positioning and range uncertainties, based on a set of auxiliary structures that are shifted versions of the clinical regions of interest (ROI: clinical target volume – CTV and organs-at-risk – OARs), and that are named clones. This approach is compared with the use of a pseudo-Planning Target Volume (PTV) and pseudo-planning OAR volumes built by the union of all the CTV and OAR clones, respectively (Figure 1). The proposed methodologies were tested using five post-operative prostate cancer cases. The quality of the calculated treatment plans was assessed by Monte Carlo simulation, taking into account treatment fractionation. The clones strategy with 6 mm deviations was able to guarantee the proper fulfilment of the dose prescription and, overall, led to a better sparing of the OARs, being evaluated as the best strategy considering the compromise between the evaluated features, i.e., dose distribution and robustness (Table 1). This strategy shows benefits when compared with the consideration of a single enlarged structure for each ROI. It is thus possible to include uncertainty explicitly into a totally automated treatment planning approach, not requiring trial-and-error or parameter tuning procedures and overcoming one of the major drawbacks of IMPT.

### P 222 - Geometrical accuracy of MRI for ocular proton beam therapy planning

#### Lisa Klaassen^1,2,3^, Patricia Cambraia Lopes^4^, Kees Spruijt^4^, Christal van de Steeg-Henzen^2,4^, Pauline Bakker^3,4^, Coen Rasch^3,4^, Berit Verbist^2,4^, Jan-Willem Beenakker^1,2,3^

##### ^1^Leiden University Medical Center, Ophthalmology, Leiden, Netherlands ^2^Leiden University Medical Center, Radiology, Leiden, Netherlands ^3^Leiden University Medical Center, Radiation Oncology, Leiden, Netherlands ^4^HollandPTC, HollandPTC, Delft, Netherlands

**Introduction**: Susceptibility artefacts caused by the nasal and oral cavities may result in geometric distortions in ocular MRI. In this study we developed a phantom and used it to assess the geometrical accuracy of a dedicated ocular MRI protocol for ocular proton therapy planning.

**Methods**: The phantom (Fig.1A-C) contained a saline solution, two 3D-printed polyamide grids (one with five tantalum clips), and several air-filled tubes, mimicking the oral and nasal cavities. The phantom was scanned at 3T MRI with a dedicated protocol with localized shimming and increased gradients, containing a gradient echo (GE) T1-weighted, spin-echo (SE) T1-and T2- weighted scans (0.8 mm isotropic acquisition voxel size) and with CT (0.3×0.3×0.5 mm^3^). Nine grid intersection landmarks (Fig.1D) and five clips (Fig.1E) were identified in a 3D viewer by two observers. Landmark-landmark and clip-clip distances on MRI were compared to CT as ground truth.

**Results**: Absolute differences between observers and imaging modalities were below the voxel size for distances up to 37 mm (Fig.2). Mean absolute differences between CT and MRI were below 0.20 mm for the landmarks and 0.45 mm for the clips and for all sequences these were not significantly different than the observer variation (p>0.3).

**Conclusion**: The small, <0.5 voxel, differences observed between CT and MRI can be attributed to inter-observer variation of clip and landmark identification. Thus, a dedicated ocular MRI protocol can produce geometrically accurate images of the eye and orbit.

### P 223 - Probabilistic approach to evaluate the robust margins used in proton treatment for brain tumours

#### Ali Alkhiat^1^, Marcus Fager^1^, Bruno Sorcini^1^, Erik Almhagen^1^

##### ^1^Karolinska University Hospital, Department of Medical Radiation Physics and Nuclear Medicine, Stockholm, Sweden

**Introduction**: At Karolinska University Hospital, Stockholm, Sweden, intracranial proton plans are currently optimized using robust optimization with a robust margin of 4 mm. The aim of this work was to evaluate if the robust margins used for these patients can be reduced.

**Material and Methods**: Patient setup uncertainties were estimated by offline-reviewing 20 patients. Uncertainties in the patient positioning system and beam delivery were estimated by analyzing quality assurance data from the Skandion Clinic, Uppsala, Sweden. Uncertainties due to intrafraction movement and delineation were taken from the literature. Treatment plans were reoptimized (Eclipse, Varian Medical Systems) using 3 mm robust margins, with all other optimization parameters preserved, for 20 patients. For each plan, 100 treatment scenarios were simulated. Every treatment scenario consisted of 30 fractions, and for each fraction and for each spot in the treatment plan, patient positioning system and beam delivery uncertainties were sampled and applied. For each treatment scenario, a delineation error of the CTV was sampled from a normal distribution, from which a modified CTV was created. As acceptance criteria, we required D98% > 95% of the prescribed dose for the modified CTV volume for at least 90% of all treatment scenarios.

**Results**: With 3 mm robust margins 99.2% and 100% of all treatments fulfilled the acceptance criteria for the modified and original CTV-volume, respectively.

**Conclusion**: The results satisfied the acceptance criteria, making it safe to reduce the robust margins to 3 mm. Future work is needed to investigate if the margins may be further reduced.

### P 224 - Probabilistic uncertainty analysis of the Dutch robustness evaluation and model-based IMPT patient-selection protocols: A multi-institutional study

#### Jesus Rojo-Santiago^1,2^, Steven J.M. Habraken^1,2^, Mirko Unipan^3^, Stefan Both^4^, Geert Bosmans^3^, Zoltán Perkó^5^, Erik Korevaar^4^, Mischa S. Hoogeman^1,2^

##### ^1^Erasmus MC Cancer Institute, Radiotherapy, Rotterdam, Netherlands ^2^HollandPTC, Medical Physics & Informatics, Delft, Netherlands ^3^Maastricht University Medical Center, Radiation Oncology, Maastricht, Netherlands ^4^University Medical Center Groningen, Radiation Oncology, Groningen, Netherlands ^5^Delft University of Technology, Radiation Science and Technology, Delft, Netherlands

**Purpose/Objective**: The Dutch Proton Therapy group has standardized a robustness evaluation protocol to ensure adequate CTV coverage and a model-based approach for IMPT patient-selection in terms of toxicity (NTCP). This work investigates consistency in CTV coverage and robustness of the patient-selection against treatment errors for IMPT H&N patients treated in the 3 Dutch centers.

**Methods**: Sixty clinical IMPT H&N plans were included, divided into subcohorts of 20 patients per center. Dose was prescribed to the voxel-wise minimum dose of 28 scenarios: VWmin-D_98%_≥L(%) (Figure 1), using different prescription levels (L) per center. Polynomial Chaos Expansion was applied to generate fast patient- and plan-specific models of voxel doses. It enabled the probabilistic evaluation of 100,000 simulated treatment courses per plan. Systematic (Σ=0.92mm) and random (σ=1.00mm) setup errors (1SD) were sampled from Gaussian distributions consistent with a margin M=2.5Σ+0.7σ=3mm. A systematic range error (1SD) of 1.5% was assumed from literature. A prior photon plan calibration suggests a V_95%_=99.8% probabilistically ensures adequate CTV coverage. To assess the protocol and patient-selection, per center population V_95%_ histograms and expected vs. nominal NTCP correlations were determined respectively.

**Results**: For center 1 and 2, the 90^th^-10^th^ percentile range of V_95%_ was above 99.8% (adequate CTV coverage). However, the V_95%_ values of center 3 were partly (CTV_70.00_) or completely (CTV_54.25_) below the goal (V_95%_=99.8%). Expected NTCPs from the uncertainty analysis agreed with the nominal values within 0.2%-point in all centers.

**Conclusion**: The Dutch robustness protocol showed inconsistent CTV coverages between centers. The nominal NTCP is robust, representing a good estimator of the expected NTCP during treatment.

### P 225 - Robust optimization of IMPT in skull base chordoma and chondrosarcoma: A single-institution clinical experience

#### Vesna Miladinovic^1,2^, Yvonne Klaver^1,2^, Stijn Krol^1,2^, Michiel Kroesen^2^, Berit Verbist^2,3^, Steven Habraken^1,2^, Wouter van Furth^4^, Ida Coremans^1,2^

##### ^1^Leiden University Medical Center, Department of Radiation Oncology, Leiden, Netherlands ^2^HollandPTC, Department of Radiation Oncology, Delft, Netherlands ^3^Leiden University Medical Center, Radiology department, Leiden, Netherlands ^4^Leiden University Medical Center, Department of Neurosurgery, Leiden, Netherlands

**Introduction**: Skull base chordomas and chondrosarcomas are rare, slowly growing malignant bone neoplasms. Despite low sensitivity to radiotherapy, proton therapy (PT) has successfully been used as adjunct to resection or as definitive treatment. In this study, we present the results of robustly optimized intensity modulated proton therapy (IMPT) of skull base chordoma and chondrosarcoma patients treated in HollandPTC, Delft, Netherlands.

**Methods**: Treatment plans of twelve patients (9 chordomas, 3 chondrosarcomas) treated with adjuvant IMPT between July 2019 and August 2021 in our institute were retrospectively reviewed. Treatment planning was performed with RayStation software (version 10B, RaySearch Laboratories, Stockholm, Sweden) using CT and 3.0T MR imaging examinations carried out in supine position in a thermoplastic mold. A cumulative dose of 70-74Gy (RBE) was administrated to the CTV with 3 or 4 beams, in 35-37 fractions with the Varian Probeam pencil beam scanning proton system (Varian Medical Systems, Palo Alto, CA, USA).

**Results**: Nominal coverage of 97.83% and voxel wise minimum dose coverage of 95.2% were noted for high dose CTV. Robust optimization was performed mainly for brainstem surface and core, and for spinal cord surface and core. (Table 1) Eleven patients had evaluation CT’s during the treatment from which nine patients had more than 3 evaluation CTs, one patient had 2, and one patient had 1 evaluation CT. Four plan adaptations were made in total. Early low-grade toxicities disappeared in the first 3 months post-PT.

**Conclusion**: Robustly optimized IMPT is clinically feasible as an adjuvant treatment in skull base chordoma and chondrosarcoma patients.

### P 226 - High-LET-dose based carbon ion treatment plan assessment: A novel evaluation concept

#### Mansure Schafasand^1,2^, Erik Traneus^3^, Lars Glimelius^3^, Andreas Franz Resch^1^, Ankita Nachankar^4,5^, Joanna Gora^1^, Dietmar Georg^2^, Markus Stock^1,6^, Antonio Carlino^1^, Piero Fossati^4,6^

##### ^1^MedAustron Ion Therapy Center, Medical Physics, Wiener Neustadt, Austria ^2^Medical University of Vienna, Radiation Oncology, Vienna, Austria ^3^RaySearch Laboratories AB, Light ions, Stockholm, Sweden ^4^MedAustron Ion Therapy Center, Radiation Oncology, Wiener Neustadt, Austria ^5^ACMIT, Medical Research and Development, Wiener Neustadt, Austria ^6^Karl Landsteiner University of Health Sciences, Radiation Oncology, Krems an der Donau, Austria

**Introduction**: Compared to the positive clinical outcomes achieved by carbon ion radiotherapy in treating small tumors, the reported results for larger tumors are inferior, indicating the necessity to better utilize the potential of these ion species, particularly their high linear energy transfer (LET) and relative biological effectiveness (RBE). “High-LET-dose” (hLD; the filtered physical dose based on a specified LET threshold) is a quantity that can act as a surrogate for dose-averaged LET and was used in this study to compare its distribution among different carbon ion treatment plans (cTP).

**Materials and Methods**: Six cTPs with a PTV<500 cc (small) and six cTPs with a PTV≥500 cc (large) were selected. The prescribed RBE-weighted dose was 73.6-76.8 Gy(RBE_LEMI_) over 16 fractions. The hLD distribution was scored for a LET threshold of 30 keV/µm (selected based on 18 different tested thresholds) and normalized to the underlying physical dose (nhLD). The nhLD distribution in the CTV was compared among the two cTP groups (large vs. small) by using nhLD volume histograms.

**Results**: The mean nhLD of the small cohort was always higher than the large one and significantly (p<0.05) different at most of the relative volumes (12%<RV_CTV_≤100%). The most significant difference (p<0.001) was observed at RV_CTV_=59% where the mean nhLD for the small and large group was 0.44±0.07 and 0.28±0.04, respectively (Figure1). nhLD_59%_≥(0.44-0.07) was considered as a new evaluation constraint.

**Conclusion**: Despite similar RBE-weighted doses, the nhLD distribution was found to be significantly lower in cTPs with large tumor compared to small ones. The findings can be used for future hLD-based optimization studies.

### P 227 - Quantification of beam range difference with the CT to stopping power ratio setting for carbon-ion radiotherapy

#### Soorim Han^1^, Changhwan Kim^1^, Yongdo Yun^2^, Jiwon Jang^3^, Eunho Lee^3^, Chae-Seon Hong^1^, Min Cheol Han^1^, Jin Sung Kim^1^

##### ^1^Yonsei University College of Medicine, Department of Radiation Oncology, Seoul, Korea Republic of ^2^Yonsei University College of Medicine, Medical Physics and Biomedical Engineering Lab MPBEL, Seoul, Korea Republic of ^3^Yonsei Cancer Center, Department of Radiation Oncology, Seoul, Korea Republic of

For carbon ion radiotherapy treatment planning, the beam range is calculated from the CT value and the corresponding stopping power ratio (SPR) and it is referred to as the CT-SPR table. Because the SPR varies with the beam energy and the materials of the target, it is necessary to consider the energy when calculating the SPR to calculate the beam range accurately in TPS. In this study, we obtained SPRs for several energies from MC simulation, calculated the beam ranges for each SPR table with TPS, and quantified the difference. The stopping power ratios for seven anatomical materials were simulated for four energies (100, 1200, 2400, and 4800 MeV) in GEANT4. After registering CT-SPR tables in TPS, we calculated the beam range in several materials with the four tables generated for each energy. In this test, RayStation11B was used. We compared the CT-SPR table generated at four different energies and the beam ranges calculated by TPS based on each CT-SPR table. The largest difference in range, more than 2 mm, was found when calculating 2400 MeV or higher energy beams with the 100 MeV SPR table. In this study, we quantitatively evaluated the difference in the range depending on the energy at which the CT-SPR was created with the SPR calculated from the MC calculation. The search for the optimal energy condition to calculate the accurate beam range is in progress. In the future, we will compare the energy with the recommended energy issued by Inaniwa et al.

### P 228 - Monte Carlo calculation for metallic and synthetic material modeling in proton therapy

#### Carlos Ramon Garcia^1^, Borja Aguilar^1^, Pedro Arce^2^, Alberto Viñals^1^, Pablo Cabello^1^, Jose Miguel Delgado^1^, Javier Serrano^3^, Pedro Rato^2^, Juan Ignacio Lagares^2^, Juan Diego Azcona^1^

##### ^1^Clínica Universidad de Navarra, Radiation Physics and Radiation Protection, Madrid, Spain ^2^CIEMAT Centro de Investigaciones Energéticas Medio Ambientales y Tecnológicas, Medical Applications Unit, Madrid, Spain ^3^Clínica Universidad de Navarra, Oncology, Madrid, Spain

**Purpose/Objective**: To ensure the accuracy of dose calculations in proton therapy in the presence of metallic prostheses and synthetic material.

**Material and Methods**: We have treated patients with prosthesis made on a basis of Titanium, Cobalt-Chrome, and with boluses made of ABS resin. Treatment planning was done in RayStation (RaySearch Laboratories AB). Modelling was done by compounds with different compositions, tweaking the mass density to match the actual material WET. We have calculated using a benchmarked Monte Carlo GAMOS/Geant4 code the proton scattering and nuclear cross sections for a sample of the actual compound vs. the model. Mass densities were those needed to reproduce the WET. Calculations were done for 7 energies between 75 and 225 MeV. In the case of the ABS, we tested and compared adipose, PlasticTE A-150, and RW3.

**Results**: Titanium prosthesis were accurately modeled as pure Titanium. Co-Cr prostheses were modeled as steel, properly reproducing its WET, as well as the proton scatter and the nuclear reactions. ABS resin was best modeled using RW3. RW3 and especially A-150 reproduced both very well the proton scattering, but nuclear cross sections for RW3 resembled much better than the other two compounds those from ABS.

**Conclusion**: A Monte Carlo comparison of the nuclear cross sections and proton scatter for the actual and modelled metallic and synthetic materials ensured the accuracy of RayStation dose calculations in the patients we have treated. Monte Carlo was very helpful to compare and deem which compound fits best the actual ABS resin.

### P 229 - Feasibility study of carbon ion radiotherapy with Simultaneous Integrated Boost (SIB) of head and neck tumors, chordomas and sarcomas

#### Marta Mumot^1^, Joanna Góra^1^, Antonio Carlino^1^, Markus Stock^1,2^, Ankita Nachankar^3,4^, Piero Fossati^3,5^

##### ^1^MedAustron, Medical Physics, Wiener Neustadt, Austria ^2^Karl Landsteiner University of Health Sciences, Medical Physics, Krems am der Donau, Austria ^3^MedAustron, Radiation Oncology, Wiener Neustadt, Austria ^4^ACMIT, Austrian Center for Medical Innovation and Technology, Wiener Neustadt, Austria ^5^Karl Landsteiner University of Health Sciences, Radiooncology, Krems am der Donau, Austria

Carbon ion radiotherapy (CIRT) for head and neck (H&N), Chordomas and Sarcomas is often delivered in 16 fractions with sequential. We investigated a SIB planning approach for this schedule. Prescribed dose for the low dose PTV was decided considering BED over a range of alfa/beta ratios (2-10 Gy). CIRT plans were re-optimized with SIB using RayStation11B TPS and LEM-I RBE-weighted dose optimization for 3 H&N cases, 2 pelvis chordomas and 3 sarcomas (1 of the head and 2 of the pelvis). We used the same dose constraints beam number and orientation as in the original sequential plan. Subsequently, plans were recomputed with the mMKM model and their LETd distributions were evaluated. Comparable coverage of the targets and similar organ sparing were achieved for both techniques, as expected SIB plans could achieve a more homogeneous LEM-I dose to low-dose target was observed. RBE recalculation resulted in larger difference between LEM-I and mMKM in the low dose target for SIB plans. mMKM CTV1 D95% was lower than nominal LEM-I by 12.7±1.9 versus 6.1±2.7 Gy RBE for SIB and sequential boost respectively. LETd evaluation did not show any significant differences. Both SIB and sequential boost approaches resulted in comparable OAR sparing, coverage of CTV2 and LET distributions. The large difference in RBE weighted dose for CTV1 between LEMI and mMKM model require careful further consideration before SIB is routinely used in clinical practice.

### P 230 - Experimental dosimetric validation of an open-source treatment planning system for IMPT dose delivery at a horizontal PBS research beamline

#### César Sepúlveda^1,2^, Benjamin Gebauer^3,4^, Aswin Hoffmann^3,4,5^, Armin Lühr^6^, Niklas Wahl^1,2^, Lucas Burigo^1,2^

##### ^1^German Cancer Research Center, Medical Physics in Radiation Oncology, Heidelberg, Germany ^2^National Center for Radiation Research in Oncology NCRO, Heidelberg Institute for Radiation Oncology HIRO, Heidelberg, Germany ^3^OncoRay—National Center for Radiation Research in Oncology, Faculty of Medicine and University Hospital Carl Gustav Carus- Technische Universität Dresden- Helmholtz-Zentrum Dresden—Rossendorf, Dresden, Germany ^4^Helmholtz-Zentrum Dresden—Rossendorf, Institute of Radiooncology—OncoRay, Dresden, Germany ^5^Faculty of Medicine and University Hospital Carl Gustav Carus- Technische Universität Dresden, Department of Radiotherapy and Radiation Oncology, Dresden, Germany ^6^TU Dortmund University, Medical Physics and Radiotherapy- Department of Physics, Dortmund, Germany

**Objectives**: This study investigates the ability of the open-source treatment planning system (TPS) matRad to generate accurate intensity-modulated proton therapy (IMPT) plans for a real-world horizontal proton pencil beam scanning (PBS) research beamline. Initial validation for IMPT dose delivery is addressed by comparing simulation data with measurements.

**Materials and Methods**: The PBS dose delivery was modelled with Monte Carlo (MC) simulations using TOPAS version 3.7. First, base data measurements were performed to develop a proton beam model (BM) for the OncoRay horizontal PBS research beamline. Measured data include spot sizes in air, depth-dose profiles and a monitor unit (MU) calibration. From BM data, matRad compatible pencil-beam kernels and metadata for IMPT treatment planning were created. For initial validation of dose delivery, simple 3D box plans were generated at three depths (target beam entrance at 10, 15 and 20cm) covering energies from 115 to 195MeV. Absolute and relative doses were measured using a Semiflex 31013 ionization chamber (PTW Dosimetry) and a scintillation detector (Lynx®, IBA Dosimetry), respectively.

**Results**: Simulations and dose measurements showed good agreement of relative dose profiles at depths of 14.75, 18.05 and 24.05cm for field sizes of 5 × 5, 10 × 10 and 15 × 15cm^2^. The overall absolute difference of measured and planned target doses was ∼1% (see Table 1).

**Conclusions**: Accurate MC-based BM was obtained and integrated into the TPS matRad. Absolute and relative dose measurements were in agreement with IMPT phantom plans. Validation will be continued with absolute and relative dosimetry of patient plans.

### P 231 - Training the future in HITRIplus project: A report on specialised course on heavy ion therapy research

#### Manjit Dosanjh^1,2^, Angelica Facoetti^3^, Maria Monica Necchi^3^, Nicholas Sammut^4^, Joseph Bateman^1^, Kristaps Palskis^2,5^, Taylor Rebecca^2,6^, Cameron Robertson^1^

##### ^1^University of Oxford, Department of Physics, Oxford, United Kingdom ^2^European Organization for Nuclear Research CERN, Accelerator Technology Sector-DO, Geneva, Switzerland ^3^CNAO Foundation, CNAO Foundation, Pavia, Italy ^4^University of Malta, Faculty of Information & Communication Technology, Msida, Malta ^5^Riga Technical University, Centre of High Energy Physics and Accelerator Technologies, Riga, Latvia ^6^Imperial College, Imperial College, London, United Kingdom

Heavy ion therapy is highly active topic including a vast of different interdisciplinary research fields. Despite this, many early-career researchers in associated fields have limited exposure to information outside of their domain. With the shortage of experienced personnel, detailed courses are essential for training and to bridge the divide between communities of young researchers in radiobiology, medical physics and technical sciences. A specialised course by HITRIplus was organised to meet these needs, aimed at researchers of different academic levels from a wide range of international and research backgrounds. To ensure the needs of the participants were understood, the organising committee included several early-career volunteers to help craft an engaging course inviting faculty of international experts to provide the relevant content. Main aspects of the course organizing process will be outlined, including the student application process and statistics, student selection criteria, choice of scientific topics covered in the course and inclusion of other relevant topics such as entrepreneurship and public speaking. The course took place in July virtually. The restricted number of course participants was offset by making the lectures available on YouTube immediately after each session. As each lecture had allocated time for interaction, excellent participation and student engagement with the faculty members was reached. The additional student sessions are also outlined and discussed. The course was successful in delivering a robust series of lectures, hands on sessions and student sections in a virtual environment. Participant feedback was highly positive and main statistics and results from course exit-poll are shown.

### P 232 - Impact of intra-fractional motion and setup uncertainties in ocular proton therapy

#### Daniel Björkman^1,2^, Riccardo Via^1^, Antony Lomax^1,2^, Guido Baroni^3^, Damien C. Weber^1,4,5^, Jan Hrbacek^1^

##### ^1^Paul Scherrer Institute, Center for Proton Therapy, Villigen, Switzerland ^2^ETH Zurich, Department of Physics, Zurich, Switzerland ^3^University Hospital Bern, Department of Radiation Oncology, Bern, Switzerland ^4^University Hospital Zurich, Department of Radiation Oncology, Zurich, Switzerland ^5^Inselspital- Bern University Hospital- University of Bern, Department of Radiation Oncology, Bern, Switzerland

Dosimetric effects of intra-fractional motion in ocular proton therapy have been investigated using an in-house developed treatment planning system (TPS). The novel TPS simulates dose and eye motion measured with an eye tracking system (ETS) to study the deterioration of the planned dose distribution. The treatment plan defined in the clinical TPS (Eyeplan) was recreated in the research TPS in order to validate consistency of results in static conditions. For a first example patient, dose was accumulated to the model of the treated eye by assesses the corresponding dose distribution at a time resolution of 1 second. Eye motion shown in figure Figure 1b resulted in cold regions of up to 10% of the prescription dose and dose increases of 17 percent outside the planned treatment field (Figure 2a). An instance of 5 seconds effectively broadened the penumbra in the isocentric plane by 0.278 mm to 2.374 mm (Figure 2b). Our research TPS offers the possibility to quantitatively evaluate the impact of intra-fractional motion on dose distributions. For a single patient, substantial changes to the treatment field have been demonstrated. This study will now be extended to a larger patient cohort.

### P 233 - Collision detection and prevention for proton therapy of head and neck cancer using ProBeam

#### Yifeng Yang^1^, Zhangmin Li^1^, Yajun Jia^1^, Zuofeng Li^1^, Yuanshui Zheng^1^

##### ^1^Guangzhou Concord Cancer Center, Radiation Oncology, Guangzhou, China

**Purpose**: The Snout of ProBeam proton therapy system is very large, making it vulnerable to collision with the patient body and treatment couch especially the ramp of the couch. However, the geometry of the Snout is not well modeled in Eclipse, the couch insert may be only partially inserted, and the treatment couch base is not considered in TPS, leading to potential collision hazards if not detected earlier. We developed a program to accurately calculate airgap and predict potential collisions for proton therapy.

**Method**: An in-house collision simulation software was developed using Python. The detailed geometry of snout head was simulated, and the structure of full couch insert and base were created and inserted for each patient in TPS. The CT images was extended so that a full treatment couch can be inserted into the exact position and added to body structure by registration of the immobilizaton device. Airgaps for real H&N patients were measured and compared to the predicted value. Furthermore, the thresholds for ProBeam to trigger collision interlock were also investigated and planned to be included in the software.

**Result**: The inhouse software was developed and in clinical use. The predicated airgap is verified to be within 3 mm of measurements.

**Conclusion**: The application we developed can be used to prevent potential collisions for proton therapy patients thus avoiding unnecessary plan change and other collision risk.

### P 234 - Reducing treatment fraction time by using two gantry angles with field-in-field beams for lung and breast patients

#### Mirko Unipan^1^, Vicki Trier Taasti^1^, Djoya Hattu^1^, Ilaria Rinaldi^1^, Marije Velders^1^, Femke Visser^1^, Judith Stoep- van der^1^, Judith Loon- van^1^, Karolien Verhoeven^1^, Gloria Vilches-Freixas^1^

##### ^1^Maastro Clinic, Proton therapy, Maastricht, Netherlands

**Purpose**: To increase treatment efficiency and patient comfort by reducing the fraction time, a treatment planning and delivery method was developed for lung and breast patients, whereby the number of gantry angles was reduced from typically 3 (up to 5) angles to 2 gantry angles using a field-in-field technique.

**Methods**: Twenty-two (22) lung and 9 breast proton patients previously treated at our proton facility were included in this study. During treatment the target coverage and organ-at-risk (OAR) dose were monitored on weekly repeat CTs. New treatment plans were made using 2 gantry angles, with 2 beams/angle using different spot spacing. These field-in-field plans were compared to the clinically delivered plans on the planning and weekly repeat CTs.

**Results**: For all patients the new field-in-field plan proved to be equivalent to the clinically delivered plan in terms of OAR dose sparing (Figure 1). For 21/22 lung patients and 9/9 breast patients, the field-in-field plan was as robust against anatomical changes as the clinical plan in terms of target coverage and OAR dose (Figure 2); only for one lung patient the target coverage on the repeat CTs for the field-in-field plan decreased under constraint, while the clinical plan met the constraint.

**Conclusion**: The field-in-field planning technique was clinically introduced at the beginning of 2022; coupled to an improved online imaging workflow, it led to a reduction in fraction time from 40 to 26 minutes on average for lung and from 44 to 32 minutes on average for breast patients, respectively.

### P 235 - Margin reduction while preserving adequate dose in axillary lymph node CTV in breast cancer IMPT

#### Jenneke Jacobs^1^, Sophie Bosma^1,2^, Emmanuelle Fleury^1,3^, Steven Habraken^1,3^, Mischa Hoogeman^1,3^, Rob Louwe^1^

##### ^1^Holland Proton Therapy Center, Radiotherapy, Delft, Netherlands ^2^The Netherlands Cancer Institute, Radiotherapy, Amsterdam, Netherlands ^3^Erasmus Medical Center, Radiotherapy, Rotterdam, Netherlands

**Purpose**: Three mm internal margins around axillary lymph nodes levels 1-2, and levels 3-4 are used during robust plan optimization for breast cancer IMPT to account for positioning uncertainty. Our aim is to investigate if these margins can be reduced or omitted for both CTVs.

**Material and Methods**: Four plans were created using 0, 1, 2 or 3mm internal margins around both CTVs. Robust optimization was carried out using 21 scenarios with 5mm setup and 3% range uncertainties. Pre-treatment CBCTs, acquired at fractions 1, 6 and 11 were used to assess the D98% in VWMin and D2% in VWMax of the delivered dose distributions. Both CTVs were re-delineated on the pre-treatment CBCTs (CTV_CBCT). The delineated volumes were transferred onto the planning-CT after rigid registration of these CBCTs with the planning-CT. The dose to the CTVs_CBCT for these fractions was assessed on the planning-CT using {3mm/3%} robust evaluation including 28 scenarios. The ipsilateral mean lung dose (MLD) was reported for nominal plans.

**Results**: Figure 1 displays the results for the internal margins for 10 patients. The median D98% of VWMin with 0, 1, 2 or 3mm margin was more than 96% for almost all cases. The median D2% of VWMax was less than 105% of the prescribed dose. The median MLD was reduced by 0.4Gy reducing the margin from 3 to 0mm.

**Conclusion**: Adequate dose to axillary lymph node levels 1 to 4 can be achieved without internal margins using {5mm/3%} robust optimization. The margin reduction has modest impact on the lung dose.

### P 236 - A novel method for FLASH radiotherapy: Combining FLASH with IMRT

#### João Lourenço^1,2^, Joana Leitão^1,3^, Niklas Wahl^4^, Filipa Baltazar^5^, José Marques^2^, João Seco^1^

##### ^1^DKFZ, E041 - Division of Biomedical Physics in Radiation Oncology, Heidelberg, Germany ^2^C2TN, Department of Nuclear Sciences and Engineering, Lisbon, Portugal ^3^LIP, Research Group Dosimetry, Lisbon, Portugal ^4^DKFZ, E0404 - Research Group Radiotherapy Optimization, Heidelberg, Germany ^5^DKFZ, E050 - Clinical Cooperation Unit Radiation Oncology, Heidelberg, Germany

This study proposes a novel strategy for multi-beam FLASH radiotherapy that combines FLASH delivered by proton beams with photon IMRT beams, allowing for a uniform coverage of the PTV. The FLASH effect has been observed for minimum doses of 6 Gy and minimum dose rates of 40 Gy/s. To achieve these conditions we used matRad, a treatment planning toolkit developed by DKFZ. We divided the PTV into different sections, which we called patch sections. For every patch section close to an OAR, a proton FLASH beam is used to deliver radiation dose. For the remaining PTV volume, photon IMRT beams are used to deposit the necessary dose (Fig.1). Furthermore, we also studied the robustness of our new multi-beam FLASH strategy to random and systematic uncertainties. We achieved both a minimum dose of 10 Gy and a minimum dose rate of 100 Gy/s in the OARs, for a FLASH beam intensity of 1*10^12 protons/s, thus allowing for their FLASH protection. We were also able to deliver an uniform dose distribution to the PTV (Fig.2), using the IMRT dose distribution patched with the FLASH dose distribution, such that: V_(100)>95%; D_98>0.95*(PD); D_2<1.07*(PD); CI>0.8 and HI<0.1. We also found that the dose delivered to the CTV was not affected by random and systematic uncertainties. We concluded that our strategy is suited for multi-beam FLASH radiotherapy, allowing for FLASH protection of the OARs without affecting PTV dose coverage. In addition, we showed that our method was robust to random and systematic uncertainties.

### P 237 - The usage of 3D printed individual compensator in proton radiation therapy for pediatric patient

#### Marzena Rydygier^1^, Marta Balamut^1^, Magdalena Gabracz^1^, Anna Spaleniak^1^, Dawid Krzempek^1^, Monika Lipa^1^, Kamil Kisielewicz^2^, Katarzyna Drosik-Rutowicz^2,3^, Tomasz Kajdrowicz^4^, Renata Kopec^1^

##### ^1^Institute of Nuclear Physics Polish Academy of Sciences- PL-31342 Krakow- Poland, Cyclotron Centre Bronowice, Krakow, Poland ^2^National Oncology Institute- National Research Institute, Krakow Branch, Krakow, Poland ^3^National Oncology Institute- National Research Institute, Gliwice Branch, Gliwice, Poland ^4^Institute of Nuclear Physics Polish Academy of Sciences- PL-31342 Krakow- Poland, Department of Radiation Research and Proton Radiotherapy, Krakow, Poland

Irradiation of shallowly located tumors with proton therapy requires the usage of range discriminators, which increase the lateral penumbras of the dose distribution. To limit the negative effect of beam dispersion on the conformity of proton plans, patient-specific compensators made in 3D printing technology can be used. In this work we will present the impact of such a compensator on dose distribution for pediatric patient irradiated at Cyclotron Center Bronowice (CCB) IFJ PAN in Krakow. Two plans were prepared for a 12-years old patient with rhabdomyosarcoma. First plan with applied standard Range Shifter (RS) (block of PMMA approx. 25 cm distant from patient surface) inserted into the beam line and second plan using individual 3D compensator attached to the patients mask (no RS in the beam path) (see fig.1). The 3D compensator was designed in a treatment planning system (Varian Eclipse 16.1) using Contouring Tool, then printed in 3D technology using PLA material. The PTV coverage, as well as the OAR doses have been evaluated (Tab. 1). The usage of dedicated 3D compensators requires additional work during the preparation of the irradiation process but allows to limit the transverse dispersion of the scanning pencil beam, allowing to significantly decrease the OARs dose exposure for shallowly located tumors.

### P 238 - Comparison of upright versus supine positioning in particle therapy of lung cancer: A 4D- dosimetric study

#### Maria Chiara Martire^1,2^, Lennart Volz^1^, Yinxiangzi Sheng^1^, Timo Steinsberger^1^, Marco Durante^1,3^, Mark Pankuch^4^, Christian Graeff^1,2^

##### ^1^GSI Helmholtzzentrum für Schwerionenforschung, Biophysik, Darmstadt, Germany ^2^Technical University, Electrical Engineering and Information Technology, Darmstadt, Germany ^3^Institute for Condensed Matter Physics, Technical University of Darmstadt, Darmstadt, Germany ^4^Northwestern Medicine Chicago Proton Center, Medical Physics, Warrenville, USA

Upright particle therapy gained renewed interest thanks to the development of commercial upright positioning and vertical CT systems. Aside from cost benefits, possible clinical advantages include increased lung volume and reduced respiratory motion. We present the first comparison between upright and supine particle therapy 4D dose calculation. For a cohort of lung cancer patients treated at the Chicago Proton Center under the PCG registry, 4DCTs acquired both in upright and supine position were available. To draw a resilient comparison, target VOI was propagated from the supine to the seated CT, and vice versa with Plastimatch. Hurdles presented by the massively different patient shape in the two positions were overcome by performing the registration in a sub-volume comprising only the lungs. Treatment plan optimization, both for carbon ions and protons, and 4D dose calculation were computed with the TRiP98 research TPS. Dose delivery with ideal rescanning, as well as the interplay effect with realistic beam delivery were considered. The plan quality was assessed through V_95,_ D_95_, V_107,_ HI and CN for the target, and V_25_ and V_20_ for heart and lung. Plan quality was comparable between the two positions. An example for one of the patients is shown in Figure 1 and Table 1. This patient showed slight plan quality improvement for the upright treatment. Comparable outcomes for the two positioning options enables to exploit the advantages of upright treatment, such as 360° angles available, reduced costs, and better patient comfort, without losing treatment quality.

### P 239 - Post-operative proton arc therapy for cervical cancer patients: Inter-fraction motion robustness and organ sparing potential

#### Elske M. Gort^1^, Jannet C. Beukema^1^, Marjan J. Spijkerman-Bergsma^1^, Stefan Both^1^, Johannes A. Langendijk^1^, Witold P. Matysiak^1^, Charlotte L. Brouwer^1^

##### ^1^University Medical Center Groningen, Department of Radiation Oncology, Groningen, Netherlands

**Purpose**: In cervical cancer radiotherapy patients are suffering from bone marrow and bowel toxicity. Proton arc therapy (PAT) can reduce the damage to organs at risk (OARs) because of its large degree of freedom in plan optimization due to the increased number of beams and energy layers (ELs) compared to two-field (2F) IMPT^1^. We investigated this PAT OAR sparing potential in post-operative cervical cancer radiotherapy compared to 2F-IMPT and VMAT, taking inter-fraction variability into account.

**Materials and Methods**: The planCT and five weekly repeated CTs (reCTs) with manually contoured OARs and target (vagina and lymph nodes) volumes were available for eight post-operative cervical cancer patients. PAT, 2F-IMPT and 2-arcs VMAT plans were created using 7-mm set-up and 3% range uncertainties. For PAT 360 ELs were distributed over 30 beams. 2-mm set-up and 3% range uncertainties were used for reCTs robustness evaluations. Accumulated doses were calculated using deformable image registrations between planCTs and reCTs. Nominal PAT OAR doses and voxel-wise minimum (vox min) accumulated D98 vagina and lymph node coverage >94% of prescribed dose 45 Gy_RBE, RBE=1.1_ were compared to VMAT and 2F-IMPT.

**Results**: Accumulated vox min D98 was >94% for all VMAT, 2F-IMPT and PAT treatments for lymph nodes (Fig.1A) and vagina (Fig.1B, except for 1 PAT treatment). PAT compared to 2F-IMPT and VMAT showed more robust sparing of OARs associated with bowel toxicity (bowel bag, Fig.2A) and bone marrow toxicity (bone marrow, Fig.2B).

**Conclusion**: PAT compared to 2F-IMPT and VMAT showed similar target coverage and great potential in bone marrow and bowel bag sparing.

### P 240 - Custom surface collimation IMPT in a patient with a deeply invasive conjunctival tumor: An in silico proof-of-concept planning study

#### Donald Cannon^1^, Vikren Sarkar^1^, Adam Paxton^1^, Sara St. James^1^

##### ^1^University of Utah Huntsman Cancer Institute, Radiation Oncology, Salt Lake City, USA

**Purpose/Objective**: Penumbra can limit normal tissue sparing in IMPT. Here we present an *in silico* study of the potential for a near-surface custom collimation device to reduce dose to OARs in a patient with a conjunctival tumor.

**Materials and Methods**: A model patient with a deeply invasive conjunctival tumor was selected for this study. The target and OARs (lens, cornea and eye) were contoured. In the treatment planning system (RayStation 11A-SP1), an approximately 3 cm thick structure was created to serve as a hemi-aperture conforming to the orbital surface with a 5 mm air gap and partially blocking the lens and cornea. This structure was variably set to a density of air, titanium, and brass. Dose calculation using the Monte Carlo algorithm was used to evaluate the different scenarios. The system modeled was the Mevion S250i with Hyperscan, employing static apertures and the dose was prescribed and normalized ensuring GTV D95=7000 CcGE.

**Results**: Surface collimation improved cornea dose with comparable GTV coverage (mean cornea dose reduced from 5473 CcGE to 4841-4921 CcGE, and cornea D95 from 2507 CcGE to 1313-1612 CcGE). Overscanning with spots beyond the edge of the surface collimator did not appreciably improve the dose fall-off.

**Conclusions**: A custom surface collimation device is shown to improve OAR sparing in a patient with a deeply invasive conjunctival tumor planned with IMPT. Further investigation is required to determine feasibility of clinical implementation and whether these findings hold over a broader range of clinical scenarios.

### P 241 - Non-Coplanar beams are not necessary for treating whole head and neck plans with proton beams

#### Byongyong Yi^1^, Jason Molitoris^1^, Jenna Jatczak^1^, Matthew witek^1^

##### ^1^University of Maryland School of Medicine, Radiation Oncology, Baltimore, USA

**Purpose**: Whole head and neck (H&N) proton plans often use non-coplanar beams to mitigate the dosimetric variations from daily setup or anatomical changes. This prohibits the use of CBCT in the patient setup and makes treatment time longer. This study is to evaluate the advantages of the non-coplanar beam plan (NBP) if any.

**Methods**: Ten H&N patients treated with 4 fields (2 coplanar and 2 non-coplanar beams) have been used. Coplanar beam plans (CBP), which have the same gantry angles as the clinical NBP but with no couch kicks, have been generated to compare the plan quality and the robustness. Thity-nine QACT images were used to compare the robustness of two planning strategies.

**Results**: Identical plan qualities to NBP in terms of the D95% of CTV’s and the mean of organs at risk (OAR) doses such as oral cavity, ipsilateral parotid glands, larynx, and spinal cords were achieved from CBP as it is shown in Fig 1. Slopes were 1.00±0.03 and R^2^ were >0.99 for both. Fig. 2 shows fractional changes in DVHs of CTVs of one of the cases. The patterns of D95% variations are almost identical between NBP and CBP. Those patterns of all 10 cases were the same within 1.5% and none of the cases showed >3%.

**Conclusion**: Ten cases tested in this study demonstrated the same plan qualities between CBP and NBP. NBP’s were not more robust to the fractional variations than those of CBP. Non-coplanar beams do not provide additional advantages in treating Whole H&N with proton beams.

### P 242 - Looking to the future: Moving an established ocular proton service from EyePlan to a commercially available 3D planning system

#### Robert Brass^1^, Katie Sanders^1^, Laura Howard^1^

##### ^1^The Clatterbridge Cancer Centre, Medical Physics, Bebington, United Kingdom

**Background**: Clatterbridge Cancer Centre has >30 years of experience in planning ocular proton treatments using EyePlan. There are few commercially available alternatives and EyePlan is still widely used around the world. However, the cessation of any future development or support brings the software to the end of its lifespan and CCC has been compelled to source an alternative solution. The chosen successor is RayOcular (RaySearch) which offers similar clinical capabilities to EyePlan and with the addition of CT/MRI based eye-model creation.

**Purpose**: To evaluate the functionality of RayOcular in comparison with EyePlan for planning clipped ocular treatments and specifically addressing how the software will integrate into the existing patient workflow – from simulation through to planning, QA and treatment.

**Method**: Planning study: 5 EyePlan plans and associated image datasets were used to recreate the equivalent treatment setup in RayOcular. Dose distributions were visually compared, along with reported clinical metrics. Service upgrade: 6 broad streams were identified for improvement: beam modelling, planning, treatment, pathway, QA and peripheral software.

**Results and Conclusions**: The transition to a new ocular planning system is part of a wider service upgrade and is a multi-faceted project requiring replacement of the associated QA and dosimetry software. Considering the planning aspect in isolation, comparisons suggest the resultant proton dose distributions remain consistent. The addition of CT brings greater certainty in the placement and registration of the clips. With increased complexity of the eye-model and high patient caseload, the increased planning time will require exploration of other efficiency savings.

### P 243 - 40 years of clinical practice in ocular proton therapy: From generic modelling to MReye-based planning

#### Emmanuelle Fleury^1,2^, Petra Trnková^2,3^, Kees Spruijt^1^, Serdar Yavuzyigitoglu^4^, Jenneke Jacobs^1^, Yvonne Klaver^1,5^, Pauline Bakker^1,5^, Jean-Philippe Pignol^6^, Marco van Vulpen^1^, Mischa Hoogeman^1,2^

##### ^1^HollandPTC, Radiation Oncology, Delft, Netherlands ^2^Erasmus Medical Center, Radiotherapy, Rotterdam, Netherlands ^3^Medical University of Vienna, Radiation Oncology, Vienna, Austria ^4^Erasmus Medical Center, Ophthalmology, Rotterdam, Netherlands ^5^Leiden University Medical Center, Radiotherapy, Leiden, Netherlands ^6^College of Medicine, Al Faisal University, Riyadh, Saudi Arabia

**Purpose/Objective**: Since 1983, proton therapy for uveal melanomas is based on a generic geometrical model for tumor and organs-at-risks. Clinical outcomes reported worldwide are all derived from this model. In this study, we aimed to investigate the feasibility of MReye-based plans using model-based treatment plan characteristics.

**Material and Methods**: Twenty-six patients from ErasmusMC treated at HollandPTC were retrospectively collected. All underwent a post-operative clips 1.5 or 3T MRI. Clinical treatment plans were generated in Varian-EOPP TPS. MReye-based plans were calculated with in-house developed dose algorithm applying the same gazing angle and aperture as in the model-based clinical plans. GTV and organs-at-risks were delineated using fused T1-weighted and T2-weighted MRI. A plan was deemed clinically acceptable if 95% of the GTV received at least 90% (54Gy) of the prescribed dose as per current practice. If this clinical goal was unsatisfied, an extra lateral margin (LM) was added, and a new plan calculated.

**Results**: A case example is presented in Fig1. Over all patients, the median EOPP-based GTV volume was 1.12cc while MReye-based GTV volume was 0.78cc (Fig2A). With the aperture in place, GTV-V54Gy coverage was reached in 50% of the patients with MReye-based plans. Median MReye-based GTV-V54Gy was 94.3%. Thirteen patients needed LM-1mm resulting in median GTV-V54Gy of 97.2% and two patients LM-2mm resulting in median GTV-V54Gy of 97.5% (Fig2B).

**Conclusion**: MReye-based planning resulted to be feasible and might significantly improve current practice, but the differences found show that a better understanding of the differences between the two techniques is important to ensure a safe transition.

### P 244 - Temporo-spatial dose heterogeneity optimization in proton arc therapy

#### Martin Soukup^1^, Kun-Yu Tsai^1^, Laurent Collignon^1^

##### ^1^Elekta, TPS Software, Stockholm, Sweden

**Objective**: To provide a method to obtain high dosimetric quality proton arc treatment plans benefiting from additional FLASH and GRID like conditions with no need for delivery changes.

**Methods**: An improved healthy organ sparing is assumed when delivering dose with very high dose rates (FLASH) and/or high spatial dose heterogeneity (e.g. GRID). Typically, these objectives are achieved by specific delivery methods (additional beam shaping hardware, ‘shoot-through’) that might lead to plan quality degradation and/or need for additional patient specific hardware. On the other hand, intensity modulated proton therapy approaches offer increased degrees of freedom that can be utilized for achieving extra plan goals. Treatment planning system Monaco supports both IMPT and proton arc planning including robustness and LET optimization goals. On top of that, in a research version of this TPS, we implemented another set of optimization goals to accomplish FLASH and GRID like conditions. In case of FLASH planning, we include an optimization goal that aims to restrict delivery of high dose in partial regions to single very short time. In case of GRID like planning, we include optimization goal aiming at maximizing spatial dose differences. Both functions can be used together or separately in selected structures and with existing plan prescription. To simplify the optimization problem, specific spot preselection can be applied as well.

**Results**: We obtain treatment plans that have comparable dosimetric quality with reference proton arc plans (Fig. 1) but improved properties regarding FLASH and GRID like conditions in selected region of healthy tissue (Fig. 2).

### P 245 - Fluence-aperture alternating optimization (FAAO) for HYPERSCAN adaptive aperture treatment planning

#### Jonathan Haefner^1^, Shuang Zhou^1^, Qinghao Chen^1^, Tianyu Zhao^1^, Tiezhi Zhang^1^

##### ^1^Washington University School of Medicine in St. Louis, Radiation Oncology, St. Louis, USA

**Purpose**: Mevion’s HYPERSCAN is equipped with a dynamic MLC which can potentially achieve superior plan quality to other proton machines without MLC. However, the treatment planning solution offered by Raystation TPS sets MLC positions based only on the target geometry, and does not optimize the MLC positions in order to achieve the best plan quality. We describe a novel fluence-based intensity modulated treatment planning technique (IMPT) to maximize the benefits of Mevion’s unique adaptive aperture (AA) design.

**Methods**: A proton beam spot at each energy layers is modeled as a composition of ideal pencil beams with Gaussian distributions in spatial and energy domains. Similar to IMRT planning, fluence-alternating aperture optimization (FAAO) starts with the optimization of a 3D proton fluence map, which is then converted to deliverable control points with MLC block shapes and spots. In the final step the spot weight is further optimized to recover the loss of plan quality in the conversion step.

**Results**: The energy spectrums and intensity profiles of each energy layer were obtained by decomposing spot dose distributions into the summation of ideal monoenergetic proton beams. Inverse optimization produced smooth 3D fluences with sharp gradients only at the boundary, which was converted to a deliverable plan that comprises spots and MLC positions at optimized energy levels.

**Conclusions**: Fluence-based optimization may achieve optimal treatment plans for complicated cases. Progress has been made towards implementing the FAAO algorithm. We have demonstrated high quality fluence optimization in multiple cases, with modest spot beam plan quality.

### P 246 - Auto plan scripting to improve quality, consistency, and reduce time for head and neck proton therapy treatment planning

#### Tyler Williamson ^1^

##### ^1^Tyler Williamson, Radiation Physics, Houston, USA

The aim of this project was to implement an auto-planning script to improve plan quality, consistency, and reduce the time it takes to create a proton therapy head and neck treatment plan. This was important, as transitioning to Raystation treatment planning system (TPS) provided a unique challenge and opportunity. The most recent 304 proton head and neck patients were collected. 152 for the manual plans in the previous TPS, and 152 for automated plans in Raystation. Time is measured in working days from physician contours complete to plan ready for physics pre-evaluation. The auto-plan script is able to help with these key areas: 1. contouring of planning structures; 2. adding a plan with standardized beam parameters; 3. adding optimization objectives, optimizing, and refining the treatment plan. The median time to create a treatment plan was reduced by 0.7 days (3 days to 2.3 days). This represents a percentage reduction of −23.53%. More plans were approved with no physician edits requested (auto-plan: 57% vs. manual plan: 43%). This is a percentage reduction of −28%. Treatment planning scripts can help standardize the process of creating a high-quality treatment plan. In addition, reduce the time, cost, and resources associated with developing a proton therapy treatment plan. Dosimetry can better utilize their time and efforts on more challenging cases. Rather than being slowed down by manual and repetitive tasks.

### P 247 - Aggressive spot reduction via L0 regularization for ultra-fast spot-scanning proton therapy

#### Junyi Fan^1^, Jian-Feng Cai^1^, Yuting Lin^2^, Gregory Gan^2^, Ronald Chen^2^, Hao Gao^2^

##### ^1^The Hong Kong University of Science and Technology, Mathematics, Hong Kong, Hong Kong ^2^University of Kansas Medical Center, Radiation Oncology, Kansas City, USA

**Purpose**: The treatment plan delivery efficiency for spot-scanning proton therapy depends on the number of proton spots to be delivered: smaller number of proton spots allows for larger minimum spot weight threshold to be imposed for all spots, which corresponds to higher dose rate. This work will develop an Aggressive Spot Reduction (ASR) method for ultra-fast spot-scanning proton therapy.

**Methods**: The ASR method utilizes L0-norm regularization to minimize the number of spots, in addition to the optimization of plan objectives. The minimum-monitor-unit (MMU) constraint is enforced with large minimum spot threshold for high-dose-rate plan delivery. Two optimization algorithms are proposed to solve ASR using Alternating Direction Method of Multipliers (ADMM) and Stochastic Coordinate Descent method (SCD) respectively.

**Results**: The ASR methods (“ASR-ADMM” and “ASR-SCD”) are validated in comparison with the MMU methods (“ADMM” and “SCD”: no L0 regularization) and a spot reduction (“SR”) method, under the scenario of large MMU threshold. The results showed that ASR-ADMM had the best plan quality in both target coverage and organ-at-risk (OAR) sparing. In contrast, ADMM and SR had substantially degraded target coverage, while all other methods had degraded OAR sparing.

**Conclusions**: The ASR methods using L0 regularization are developed and shown to outperform state-of-art methods (e.g., SR and SCD) for ultra-fast spot-scanning proton therapy.

### P 248 - Robust or not? High dose volumes within the prostate for proton treatments

#### Mariluz De Ornelas^1^, Robert Kaderka^1^, Matthew Abramowitz^1^, Michael Butkus^1^

##### ^1^University of Miami, Radiation Oncology, Miami, USA

**Purpose**: Dominant intraprostatic lesions (DIL), located within a larger prostate CTV are known to be the most common site of local recurrence for prostate cancer. Our clinical standard is to treat these lesions in a simultaneous integrated boost (SIB) approach without robustness on the DIL to minimize the maximum dose to nearby organs at risk. As a preliminary study, we retrospectively reviewed to see if robustness to the DIL would be indicative of higher tumor control probabilities (TCP) or normal tissue complication rates (NTCP).

**Methods**: Using five consecutive patients, treatment plans were remade using the same clinical optimization constraints but with robustness (5mm/3.5%) placed on the DIL (V95%=100%). TCP, NTCP (rectum and bladder), and complication-free TCP (CFTCP) rates were calculated for all nominal and uncertainty plans to evaluate potential differences in robust- or non-robust-DIL approaches.

**Results**: Of the 65 scenarios analyzed, 38 (65%) resulted in higher CFTCP from using robustness. The 27 with lower CFTCP,19 (70%) were in cases in which the DIL was less than 5mm away from the rectum and the larger high dose cloud from using robustness pushed dose into the rectum. Averaged across all scenarios mean DIL coverage was 1.7% higher in the robust cases.

**Conclusion**: More patients are needed; however, initial results indicate that robustness to DIL results in increasing CFTCP and increased coverage of DILs. However, DIL’s located within the geometric robustness setting will result in worse CFTCP due to increased dose to the rectum.

### P 249 - Proton vs. photon arc therapy for prostate cancer with hip prosthesis

#### Maryam Moteabbed^1^, Harald Paganetti^1^, Jason Efstathiou^1^

##### ^1^Massachusetts General Hospital, Radiation Oncology, Boston, USA

**Purpose**: We investigate the value of proton therapy for prostate cancer in the presence of unilateral hip prosthesis by comparing proton arc (PARC) and lateral-anterior-oblique (PLAO) beams with standard-of-care volumetric modulated arc therapy (VMAT) in terms of dosimetry and robustness.

**Methods**: Ten patients receiving weekly CT scans were selected from a randomized clinical trial. Treatment dose was 70 Gy to prostate and 50 Gy to proximal seminal vesicles, delivered over 28 fractions. Hydrogel spacers were used for rectum sparing. Hip prosthesis was simulated by density override of left femoral heads to 4.5 g/cc for Titanium. PARC, PLAO and VMAT plans were created, including robust optimization to range and setup uncertainties. The robustness to range and interfractional variations was evaluated by accumulating the dose recalculated on the weekly CTs.

**Results**: Target coverage was comparable among plans. Median of mean bladder dose for PARC was smaller than PLAO and VMAT by 1.2 and 5.8 Gy, respectively. Median of mean rectal dose for PARC was 0.7 Gy larger than PLAO and 10.7 Gy smaller than VMAT. Both proton plans completely avoided the prosthesis (maximum dose<1Gy), whereas VMAT delivered maximum dose of 12 Gy to this volume. PARC delivered significantly less dose to the intact femoral head, whereas VMAT delivered ∼6Gy less mean dose to the penile bulb than protons. Both proton plans were robust to range and interfractional changes.

**Conclusion**: PARC is promising for treatment of prostate cancer with hip prosthesis. Investigating the effect of linear energy transfer on the urethra is underway.

### P 250 - Open-source database to facilitate plan comparisons, clinical trial DICOM file organization, and knowledge base planning

#### Thomas Whitaker^1^, Alexandra Leone^1^, Clifton D. Fuller^2^, Amy C. Moreno^2^, Steven Frank^2^, Gary B. Gunn^2^, Karen Hoffman^2^, Laurence Court^1^, Narayan Sahoo^1^, Ron Zhu^1^

##### ^1^MD Anderson Cancer Center, Radiation Physics, Houston- Tx, USA ^2^MD Anderson Cancer Center, Radiation Oncology, Houston- Tx, USA

Collecting and organizing dosimetry data is a time-consuming process critical for projects based on plan comparisons, trial data, or knowledge-based planning techniques. We present a database system, utilizing MongoDB, designed to organize DICOM treatment planning data. The Flask Python library was used to create a web service workflow that imports DICOM files along with contextual data and organizes them into logical groups that can be queried according to the contextual tags. After the files are collected, the DICOMpyler-core library is used to extract DVH metrics from the RTDOSE and RTSTRUCT files for each structure in each group of files. The result is a database that can be queried for DVH metrics according to the contextual labels. Furthermore, we provide an enrichment path that allows DVH metrics to be extracted for additional contour sets for existing dose files or additional dose files for existing contour sets. We are careful to maintain the connection of the additional files to the primary group during the enrichment process. We also provide labels to differentiate the primary contours from any other sets. The enrichment path is a powerful tool for comparing different dose calculations, such as fixed RBE (1.1) compared to variable RBE models or comparing DVH results for clinical contours compared to AI-generated contours. Our system provides an effective method for storing and retrieving DICOM files and dosimetry data that are related yet multifaceted, allowing the data to be quickly organized and used confidently for multiple projects.

### P 251 - A comparison of ocular melanoma plans using an ocular nozzle with a reconfigured range shifter location for Pencil Beam Scanning

#### Hazel Wang^1^, Amanda Duggal^1^, Mark Pankuch^1^, William Hartsell^1^, Mingcheng Gao^1^

##### ^1^Northwestern Medicine, Radiation Oncology, Warrenville, USA

**Purpose**: Proton therapy for ocular melanoma is commonly treated using a dedicated Eyeline or Uniform Scanning(US). Most modern proton centers are equipped with only Pencil Beam Scanning(PBS) which fails to produce acceptable intraocular plans due to poor distal and lateral conformality. In this study the plan quality using aperture based PBS with a range shifter(RS) relocated upstream is evaluated.

**Methods**: Ten patients with different locations and sizes of intraocular tumors were planned with US using 2-3 conformal fields following our institution’s intraocular criteria. A virtual PBS machine was constructed with a 7.5cm WET RS positioned 50cm upstream from the aperture. PBS spot patterns were optimized using the same beam geometry to achieve identical tumor coverage as the US plans. PBS distribution was calculated using Monte Carlo. All air gaps were minimized as small as clinically achievable. PBS plans passed robustness criteria with all scenarios meeting CTVD95>95% with 2mm translational offsets and 3.5% range uncertainty.

**Results**:The average conformity index 95% and 50% reference isodose was better for PBS(1.43±0.22, 5.21±1.24) than US(1.72±0.56, 9.34±2.93) for all plans(p=0.025, p=0.000). The mean dose to anterior structures ciliary body, lacrimal gland, and D0.03cc lens were significantly lower than in the US plans(p<0.016). There were no significant improvements with PBS to the mean dose of the macula and optic disc(p>0.204) and to D0.03cc of the optic nerve, and retina(p>0.122).

**Conclusions**:PBS apertures with reconfigured RS and multiple beams is better for anterior OAR sparing and non-inferior to US planning of intraocular targets.

### P 252 - Comparison of prostate cancer proton therapy and analysis of single-field treatment feasibility

#### Yuanlin Yan^1^, Mei Chen^1^, Cheng Xu^1^, Jiayi Chen^1^

##### ^1^Ruijin Hospital- Shanghai Jiaotong University School of Medicion, Department of Radiation Oncology, Shanghai, China

**Purpose**: This study aimed to investigate whether it is feasible to treat prostate cancer patients with single-field as an alternative of two-field in spot scanning proton therapy.

**Materials and methods**: Eight spot scanning proton plans with single-field for intermediate-risk prostate cancer were compared to plans with two-field. The plans with single-field take turns left and right irradiation direction. The CTV1 was provided 60 Gy(RBE) at 2 Gy (RBE) and the CTV2 was provided 76 Gy (RBE) at 2 Gy (RBE). Treatment plans were robustly optimized in RayStation 10B accounting for 5 mm setup uncertainties and 3.5% range uncertainty.

**Results**: The average (±standard deviation) D99, D95, target average dose of CTV1 for single-field plans was higher 0.73% ± 0.28%, 0.73% ± 0.24%, 0.75% ± 0.22%, respectively, compared to two-field plans. The average (±standard deviation) D99, D95, target average dose of CTV2 for single-field plans was higher 0.76% ± 0.27%, 0.72% ± 0.2%, 0.76% ± 0.2%, respectively, compared to two-field plans. The average (±standard deviation) dose of 1cc absolute volume of rectum for single-field plans was higher 0.72% ± 0.28% than for two-field plans. The average (±standard deviation) dose of 1cc absolute volume of bladder for single-field plans was higher 0.82% ± 0.19% than for two-field plans.

**Conclusion**: This study demonstrates that there is no significant dosimetry difference between single-field and two-field for prostate cancer spot scanning proton radiotherapy. Thus, the prostate cancer spot scanning proton therapy with single-field can be utilized without rotating couch or gantry to shorten the treatment time.

### P 253 - Left whole breast irradiation with upright Spot-scanning Linac Proton Arc

#### Noora-Mari Pienimäki^1,2^, Wioletta Kozłowska^1,2^, Janine Bossé^2^, Jonathan Farr^1,2^, Anna Kolano^1,2^

##### ^1^Applications of Detectors and Accelerators to Medicine ADAM SA, Clinical Office, Meyrin, Switzerland ^2^Advanced Oncotherapy plc, Clinical Office, London, United Kingdom

**Introduction**: Whole breast radiotherapy (WBRT) is a popular treatment for early-stage breast cancer. However, left-side WBRT is associated with a high risk of cardiac morbidity. Spot-scanning Proton Arc (SPArc) therapy [1] can reduce dose delivered to organs at risk (OARs). While supine SPArc requires a complex gantry solution, an upright SPArc may use rotating chair. This study evaluates the feasibility of upright SPArc for left-side WBRT.

**Methods**: A digital anthropomorphic XCAT2 phantom was used as patient model [2]. Simulated breath-hold considered different expansion of the diaphragm and chest in supine and upright positions. Treatment plans created in RayStation 12A (RaySearch Laboratories AB, Sweden) used the LIGHT (Advanced Oncotherapy plc, UK) proton beam model, targeting 4256 cGy to 98% clinical target volume.

**Results**: Obtained plans (Fig1) used the same number of angles and comparable numbers of spots. The OARs received similar dose per volume for both positions. With robust evaluation (5 mm positioning and 3.5% density error), no difference in target coverage was observed in planning target volume, although OARs dose sparing was better in the upright position in all uncertainty cases (0.03% heart volume receive 40cGy in supine and 12 cGy in upright position). No significant differences in conformity or homogeneity indexes were observed (Fig2).

**Conclusion**: Upright SPArc for WBRT provides comparable treatment outcome to the supine SPArc, with better OARs sparing in robust evaluation. Additionally upright position may significantly.

### P 254 - Impact of sub-spots calculation parameter on proton PBS treatment planning

#### Jailan Alshaikhi^1^, Gary Royle^2^, Derek D’Souza^3^, Richard Amos^2^

##### ^1^Saudi Proton Therapy Center- King Fahad Medical City, Medical Physics Department, Riyadh, Saudi Arabia ^2^University College London, Department of Medical Physics and Biomedical Engineering, London, United Kingdom ^3^University College London Hospital, Department of Radiotherapy Physics, London, United Kingdom

**Aim**: To investigate plan quality and computation time as a function of XiO’s sub-spots calculation parameter.

**Introduction**: Several treatment planning systems (Pinnacle^3^, RayStation, XiO) use sub-spots in the dose calculation, improving accuracy in the presence of lateral heterogeneities. For both Pinnacle^3^ and RayStation, this parameter cannot be user-defined. In Pinnacle^3^ this parameter depends on in-air spot size and spot-spacing; calculation guaranteeing a minimum of 4 sub-pencils per 3-sigma. Additionally, Pinnacle^3^ computes 4-sigma, yielding at least 134 sub-spots per spot. For RayStation, the number of sub-spots, *n*_s_, is fixed at 19 per spot. In XiO, the formula: *n*_s_ = (2*n*+1)^2^. (1) defines the number of pencil beams per spot, where n (precision parameter) is user-defined and ranges from 0-5.

**Methods**: Plan quality and computation time were evaluated as a function of n for XiO (Elekta CMS Software, Maryland Heights, MO) v4.8.00 using a glioma case. Five IMPT plans were calculated using *n*=0,1,2,3,4 corresponding to 9,25,49,81 sub-spots, respectively.

**Results**: Parameters of plan quality as a function of n are shown in Figure 1. Total computation time increased as a function of n from 92 to 150 minutes.

**Conclusions**: It has been demonstrated that using multiple sub-spots in XiO improves plan quality, without additional improvement beyond 9 sub-spots. Our observations compare well with RayStation’s internally fixed 19 sub-spots. Pinnacle^3^ calculates at least 134 sub-spots which may extend calculation times.

### P 255 - A split-Bregman approach can solve beam orientation optimization with nonlinear RBE dose fidelity

#### Pavitra Ramesh^1^, Dan Ruan^1^, Ke Sheng^2^

##### ^1^University of California Los Angeles, Radiation Oncology, Los Angeles, USA ^2^University of California San Francisco, Radiation Oncology, San Francisco, USA

Direct biological dose optimization for proton therapy with relative biological effectiveness (RBE) weighting can improve biological effectiveness but is computationally challenging due to nonlinear RBE-dose dependence. The split optimization strategy presented decouples the calculation of McNamara RBE from our beam orientation optimization (BOO) algorithm. RBE-weighted BOO can be solved using a split-Bregman approach as follows: 1) the biological dose fidelity terms (block I) are separated from the beam-regulating group sparsity term (block II); 2) the two variables from blocks I and II approach each other with an equality constraint; 3) each block is iteratively solved by the FISTA algorithm, with periodic RBE re-calculation using the McNamara model for final dose weighting. This method (SB) was compared with LET-weighted BOO (LET) on three head and neck patients. CTV homogeneity index (HI), D95%, maximum RBE-dose, and OAR mean and maximum RBE-dose were evaluated along with worst-case statistics. Compared to LET, CTV [HI, Dmax, D95%] improved with SB on average by [0.09, 36.8%, −17.4%]. SB also improved [Dmean, Dmax] in OARs on average by [2.4, 9.4] GyRBE, respectively. As for robustness, SB improved the worst CTV [Dmax, D95%] by [37.5%, −4.4%] of prescription dose and reduced the worst OAR [Dmean, Dmax] on average by [1.7, 6.3] GyRBE but increased by [1.8, 2.3] GyRBE in larynx and constrictors. The splitting scheme provides an alternative solution for performing proton beam selection without compromising RBE modeling accuracy. We have shown that BOO performed with McNamara RBE is superior in dosimetry and robustness compared to LET-based methods.

### P 256 - Neutron dose equivalent increase from the use of range shifter in proton fields

#### Maite Romero-Expósito^1^, Malgorzata Liszka^1^, Athanasia Christou^1^, Iuliana Toma-Dasu^2^, Alexandru Dasu^1^

##### ^1^The Skandion Clinic, i, Uppsala, Sweden ^2^Stockholm University, Medical Radiation Physics, Stockholm, Sweden

Proton therapy of superficial targets often require the addition of a range shifter (RS) to bring the proton energies in the necessary range, at the cost of neutron production. These are a concern due to their high radiobiological effectiveness and the risk for second cancer induction. The purpose of the present work was to evaluate the neutron dose increase due to RS in patients treated with proton beam scanning (PBS). Two clinical plans, identical in terms of target coverage, were created in Eclipse treatment planning system (TPS) for an anthropomorphic phantom using one lateral field. One of the plans included the RS. MCNP code was used to simulate the plans and evaluate the neutron dose equivalent (*H_n_*). In the RS plan, neutrons produced inside the patient were assessed separately. Calculated proton doses in the target were within 1% from the TPS. Table 1 shows *H_n_* and maximum *H_n_* increase due to RS (Δ*H_n max_*) obtained at 1, 5, and 10 cm from field edge. In general, both quantities became lower as the distance to field edge rose. Variations for the same distance were due to position relative to the proton beam, being highest in locations behind the target. Table 1 shows that the relative contribution of the RS to H_n_ was greater when moving farther from the target. From 5 cm from field edge (around the 5% proton isodose), Δ*H_n_* was estimated to reach up 0.27 mSv/Gy. These indicate a low neutron contribution from the use of RS in PBS.

### P 257 - Robust beam selection based on water equivalent thickness analysis in passive scattering carbon-ion radiotherapy for pancreatic cancer

#### Zhou Yuan^1^, Makoto Sakai^2^, Tatsuya Ohno^2^, Yoshiki Kubota^2^, Yang Li^2^, Masahiko Okamoto^2^, Shintaro Shiba^1^, Shohei Okazaki^1^, Toshiaki Matsui^1^

##### ^1^Gunma university, Graduate School of Medicine, Gunma, Japan ^2^Gunma University, Heavy Ion Medical Center, Gunma, Japan

**Background**: Carbon-ion radiotherapy (CIRT) is one of the most advanced radiotherapy modalities. However, anatomical variations during the treatment may distort the carbon-ion beam, leading to dose degradation. This study aimed to select a robust-beam configurations (BC) by water equivalent thickness (WET) analysis in passive CIRT for pancreatic cancer.

**Method**: Overall, analysis was performed on eight pancreatic cancer patients using 110 computed tomography (CT) images and 600 dose distributions. The robustness in beam range was evaluated by calculating the WET variation every 5°using both planning CT and daily CT images, and two robust BCs for the rotating gantry and fixed port were selected. The planned, daily, and accumulated doses were then calculated and compared after bone matching (BM) and tumor matching (TM) between the planning CT and the daily CT. Dose-volume parameters for target and OARs were evaluated.

**Results**: Posterior oblique beams (120°–240°) in the supine position and anteroposterior beams (0° and 180°) in the prone position were the most robust to WET changes. The mean CTV V95% reductions with TM were −3.8% and −5.2% with the BC for gantry and the BC for fixed ports, respectively. The OAR dose was slightly increased, but robustness was ensured and did not reach a level where the risk of adverse events was a concern.

**Conclusions**: The robustness of dose distribution can be improved by BCs that are robust to ΔWET. Robust BC with TM appears to be effective in improving the accuracy of passive scattering CIRT for pancreatic cancer.

### P 258 - Volume dose evaluation of dose to medium vs. dose to water reporting mode of a commercial fast Monte Carlo method

#### Yi-Chun Tsai^1^, Chang-Shiun Lin^2^, Chun-Wei Wang^1^, Hsiang-Kung Liang^2^, Wan-Yu Chen^2^, Sung-Hsin Kuo^2^

##### ^1^National Taiwan University Hospital, Division of Radiation Oncology- Oncology, Taipei, Taiwan Province of China ^2^National Taiwan University Cancer Center, Radiation Oncology, Taipei, Taiwan Province of China

**Background**: In comparison to the proton convolution superposition algorithm (PCS), Acuros PT (ACU) is a Monte Carlo-like algorithm designed to improve the dose calculation accuracy and efficiency. It supports two reporting modes: dose-to-medium (Dm) and dose-to-water (Dw). This may result in a dilemma of reporting either Dm or Dw. This study aims to compare the dose difference between Dm and Dw of ACU and PCS algorithms in Eclipse on a virtual water phantom.

**Materials and Methods**: A 40-by-40-by-40 cm^3^ virtual phantom was created in Eclipse, a t-box installed on-site. Various plans consisting of different square field size (F, cm), nominal range (R, cm), and modulation-width (M), were calculated using PCS, ACU and reporting in Dm and Dw. The 100% dose of each plan was normalized to the central point of SOBP (mid-SOBP). Depth profiles along central axis and lateral profiles at the mid-SOBP were plotted. The penumbra, nominal range, and point-to-point dose difference were analyzed and plotted to compare the performance of the algorithms.

**Results and Conclusions**: Preliminary results showed the difference between Dm and Dw reporting modes were within 0.3 mm for both range and penumbra. In addition, the depth and lateral profiles were almost perfect matched to each other. In comparison to ACU (Dm), PSC tended to overestimate dose distribution approximate 5% at depths shallower than 5 cm for all cases (F*_3,10,20_*R*_30,12_*M*_4,10_*). Ongoing investigations are performed to further evaluate the difference in heterogeneous phantoms.

### P 259 - Balancing clinical and research needs of an in-house treatment planning system: Our first year experience with FIonA

#### David Oxley^1^, Francesca Albertini^1^, Rico Besson^1^, Alessandra Bolsi^1^, Jan Hrbacek^1^, Gabriel Meier^1^, Arturs Meijers^1^, André Nanz^1^, Carla Winterhalter^1^, Antony Lomax^1^

##### ^1^Paul Scherrer Institute, Center for Proton Therapy, Villigen, Switzerland

A handful of major vendors cater for the treatment planning needs of the global proton therapy community. While these commercial software grant excellent infrastructure for standard clinical practices, explorative research thrives under more flexible conditions and benefits from the possibility to undertake structural and source code changes. A dedicated platform is crucial to pursue this research and determine the feasibility of novel ideas. Such insight is invaluable in shaping the planning systems of tomorrow. The Paul Scherrer Institute has developed such a framework to facilitate treatments on its unique Gantry2. In 2021, the first patient was treated and since then this new treatment planning system – FIonA – has become an established clinical workhorse treating close to 200 planning series across just under 100 patients. The system accommodates the full clinical workflow offering numerous fast GPU-based optimization and analytical dose calculation algorithms. It is also equipped with integrated GPU-based Monte-Carlo capabilities. The platform incorporates technical standards like DICOM where possible. To maximize independence and extendibility, proprietary interfaces using e.g. XML or JSON are offered to surrounding systems. Meanwhile FIonA has been expanded to support daily adaptive re-optimizations of a treatment plan employing daily imaging data. It utilizes a maximally automated DICOM transfer workflow to process the input data of each daily fraction. A daily customized treatment plan can be delivered ∼ 5 minutes after daily CT acquisition with minimal user interaction. Highlights of a year’s clinical and research experience with a focus on daily adaptive therapy alongside other research interests shall be presented.

### P 260 - Dual-layer Computed Tomography based carbon ion treatment planning with matRad toolkit and dose verification using TOPAS Monte Carlo code

#### Euntaek Yoon^1,2^, Seongmoon Jung^2,3,4^, Jung-in Kim^2,3,4^, Chang Heon Choi^2,3,4^, Hyung Jin Choun^1,2^, Kyung Su Kim^3^, Jong Min Park^2,3,4,5^

##### ^1^Seoul National University, Interdisciplinary program in bioengineering, Seoul, Korea Republic of ^2^Seoul National University Hospital, Biomedical Research Institute, Seoul, Korea Republic of ^3^Seoul National University Hospital, Department of Oncology, Seoul, Korea Republic of ^4^Seoul National University Medical Research Center, Institute of Radiation Medicine, Seoul, Korea Republic of ^5^Seoul National University College of Medicine, Department of Radiation Oncology, Seoul, Korea Republic of

**Background and purpose**: In particle therapy, it is known that dual-energy computed tomography (DECT) can reduce uncertainty in range prediction. This study aims to establish the procedure to generate a carbon ion treatment plan based on dual-layer computed tomography (DLCT), which is a kind of DECT, with matRad toolkit and verify using TOPAS Monte Carlo code.

**Methods**: From the Philips IQon Spectral CT, which is the DLCT scanner, the relative electron density (ED) image and the effective atomic number (Zeff) image were acquired. Considering the parallel comparison pencil beam algorithm (PBA)-based dose calculation with Monte Carlo (MC) simulation, the elemental composition and mass density of each voxel were driven via tissue decomposition method using DECT.[1] Each pixel-specific material information was converted into relative stopping power (RSP) and optimized treatment plan was generated using matRad toolkit. Finally, MC dose calculation was performed with the generated plan and the material information of the image. The MC simulation was performed with TOPAS code and its in-house developed extension (Figure 1).

**Results**: The DLCT-based carbon ion treatment plan for abdominal cancer patient was successfully generated and the MC dose calculation was carried out (Figure 2). The results were compared using dose-volume histogram (DVH) parameters. The D95 for (plan/MC) was (3.19/3.17 Gy[RBE]) and D5 was (3.62/3.75 Gy[RBE]).

**Conclusion**: The DLCT-based carbon ion treatment plan generation and MC dose calculation procedure was established.

### P 261 - Study of the effects of different synchrotron parameters on treatment efficiency via rescanning with gating techniques

#### Xiufang Li^1^, Zhiling Chen^2^, Manzhou Zhang^2^

##### ^1^Shanghai Advanced Research Institute Chinese Academy of Sciences, Particle beam application Technology Department 3/5000, Shanghai, China ^2^Shanghai Advanced Research Institute- Chinese Academy of Sciences, Particle beam application Technology Department, Shanghai, China

**Aim**: The main advantages of synchrotron systems are adjustable energy and high beam utilization, in addition to the little impact on environmental radiation compared to the conventional passive scattering system. But the disadvantage of this system is that it takes a long time to change energy, usually 2∼3 seconds. For the treatment of conventional tumors, the beam delivery time of one field is 2∼3 minutes, basically meeting the requirements of clinical use. However, for the tumors with rescanning with gating techniques, treatment time will be significantly increased. We aim here to study the effects of the different synchrotron parameters on treatment efficiency rescanning with gating techniques.

**Methods**: Targets of different sizes and shapes are selected for our study, such as spheres, cubes, and clinical targets. Then, setting the different models of synchrotron systems, as follows. The beam delivery times for different targets under different models are calculated and compared.

**Results and Conclusions**: By comparing the beam delivery times of the above targets in different models, we can study the treatment efficiency and performance of synchrotron, providing direction for future

### P 262 - Nanodosimetric quantity-weighted dose optimization for ion-beam treatment planning

#### Jingfen Yang^1^, Qiang Li^1^

##### ^1^Institute of modern physics- Chinese Academy of Sciences, Institute of Medical Physics, Lan Zhou, China

To achieve accurate ion-beam treatment planning and reduce the biological uncertainty caused by the relative biological effectiveness (RBE) values, a treatment planning strategy based on the physical quantities of nanodosimetry is proposed. It has been well known that nanodosimetric quantities such as the cumulative probabilities are considered to be best correlated with the radiobiological effect. The statistical distributions of the ionization cluster size v probabilities P(v|Q) for photons and ion beams could be measured by nanodosimeter or calculated by Monte Carlo (MC) simulation, and thus the cumulative probabilities $F_{n}$ with v≥n could be calculated definitely. Then the nanodosimetric quantity-weighted dose (NQWD) could be deduced as follows: $\mathrm{NQWD}=D\times \frac{F_{n\_\mathrm{lon}}}{F_{n\_X}}$. According to the experience of photon therapy, the NQWD value for ion-beam treatment planning dedicated to a certain disease could be determined. What’s more, the existing algorithm for RBE-weighted dose (RWD) optimization could be readily applied to the NQWD-based optimization, and the treatment plans with uniform NQWD values in the target volumes are obtained. This method could not only make full use of the essential characteristics of ion-beam radiation quality, but also reduce the biological uncertainty existing in the RWD-based ion-beam treatment planning optimization. With the development of experimental nanodosimetry, nanodosimetric quantities could be measured directly, and the NQWD-based ion-beam treatment plans could be verified quantitively and rapidly. Therefore, precise ion-beam irradiation could be realized, thereby ensuring the safe and effective implementation of ion-beam irradiation. In addition, nanodosimetric quantities can also be applied to radiation protection, such as the reassessment of biological equivalent dose.

### P 263 - Clinical benefit of proton treatment planning based on dual-energy CT for neuro-oncological patients

#### Vicki Taasti^1^, Esther Decabooter^1^, Daniëlle Eekers^1^, Inge Compter^1^, Ilaria Rinaldi^1^, Marta Bogowicz^1^, Esther Kneepkens^1^, Gabriel Fonseca^1^, Mirko Unipan^1^, Wouter van Elmpt^1^

##### ^1^Department of Radiation Oncology MAASTRO, GROW – School for Oncology- Maastricht University Medical Centre+, Maastricht, Netherlands

**Background and Purpose**: Several studies have shown that dual-energy CT (DECT) can lead to improved accuracy for proton range estimation. This study investigated the clinical benefit of reduced range uncertainty, enabled by DECT, in robust optimization.

**Methods**: DECT scans for 27 neuro-oncological patients were included. Commercial software was applied to create stopping-power ratio (SPR) maps based on the DECT scan. Two plans were robustly optimized on the SPR map, keeping the beam and plan settings identical to the clinical plan. One plan was robustly optimized and evaluated with a range uncertainty of 3% (as used clinically; denoted 3%-plan); the second plan applied a range uncertainty of 2% (2%-plan). Both plans were optimized to be clinical acceptable and optimal. The dose-volume-histogram parameters were compared between the two plans. Two experienced neuro-radiation oncologists determined the relevant dose difference for each organ-at-risk (OAR). Moreover, the OAR toxicity levels were assessed.

**Results**: For 24/27 patients, a dose reduction >0.5/1 Gy (relevant dose difference depending on the OAR) was seen in one or more OARs for the 2%-plan; e.g. for brainstem D0.03cc in 10 patients, and for hippocampus D40% in 6 patients (Table 1). Furthermore, 12 patients had a reduction in toxicity level for at least one OAR, showing a clear benefit for the patient.

**Conclusion**: Robust optimization with reduced range uncertainty allows for reduction of OAR toxicity, providing a rationale for clinical implementation. Based on these results, we have clinically introduced DECT-based proton treatment planning with 2% range uncertainty for our neuro-oncological patients (Figure 1).

### P 264 - Development of an in-air profile assessment method for a commercial treatment planning system

#### Chang-Shiun Lin^1,2^, Liang-Chieh Lin^2^, Yi-Chun Tsai^3^, Chun-Wei Wang^3^, Hui-Yu Tsai^2^

##### ^1^National Taiwan University Cancer Center, Radiation Oncology, Taipei, Taiwan Province of China ^2^National Tsing Hua University, Institute of Nuclear Engineering and Science, Hsinchu, Taiwan Province of China ^3^National Taiwan University Hospital, Division of Radiation Oncology- Department of Oncology, Taipei, Taiwan Province of China

**Background**: To achieve correct and safe radiotherapy, accuracy of beam modeling for a treatment planning system (TPS) is important. In-air spot profile is one of the essential parameters for most TPSs, including Varian’s Eclipse. Intuitively, evaluating the modeling results directly on the same platform, that is Eclipse, is an efficient workflow. However, Eclipse has not supported the dose calculation in-air in the focus window of External-Beam-Planning. To directly assess the accuracy of modeling for in-air spot profiles on Eclipse, a method is proposed.

**Materials and Method**: This method is conducted the dose profile measurement in a virtual water phantom generated using Eclipse. The dose profile at 0.3 g/cm^2^ is considered to provide similar distribution measured in air (Figure1(c)). An evaluated source parameter set and Monte Carlo simulator were applied to evaluate the feasibility of proposed method. The impacts of scatted events, including backscatter (Figure1(b)) and combination of shallow region- and back-scatter (Figure1(c)), on the spot profile for mono-energetic proton beams were assessed using sigma (σ) and point-to-point dose difference.

**Results**: In general, the discrepancy of spot size between reference and the proposed method was less than 0.5% for the selected energies. In comparison to the reference configuration, the point-to-point percentage dose differences resulting from backscatter and the combination-scatter were overestimated with 1% and 5% (Figure2), respectively.

**Conclusions**: Preliminary results indicate that the method is feasible for proton beams with selected energies. To assess the response of energies for clinical applications and obtain an optimal reference depth, further investigations are necessary.

### P 265 - NanOx-based multiscale modeling of the biological dose in BNCT to estimate the impact of boron microdistributions

#### Maria Pedrosa Rivera^1,2^, Victor Levrague^1^, Mario Alcocer-Avila^3^, Rachel Delorme^1^, Etienne Testa^3^, Michaël Beuve^3^

##### ^1^LPSC, University Grenoble Alpes - CNRS/IN2P3 - Grenoble INP, Grenoble, France ^2^University of Granada, Department of atomic- molecular and nuclear physics, Granada, Spain ^3^IP2I, Université Claude Bernard Lyon 1- CNRS/IN2P3, Villeurbanne, France

BNCT is a promising therapy for bad prognosis tumors like glioblastoma or recurrent head and neck cancer. The benefit of BNCT relies on both the boron targeting into the tumor and the biological effectiveness of the low-energy ions following the neutron capture. Biological dose modeling is therefore required in order to optimize the treatment plans. Several formalisms have been presented over the years but they do not consider the microdistribution of boron that should strongly impact tumor control probability (TCP). The present study aims at quantifying this impact using multiscale modeling and consideration of biological effect due to each secondary particle instead of weighting factors. The scales range from nanometers, through the biophysical model NanOx, up to the patient for which the resolution is defined by the voxel size. NanOx has proven its accuracy to describe the cell response under several ion-type irradiations of interest for hadrontherapy. Our multiscale methodology can be decomposed in 4 stages: Modeling of multicellular geometry and various scenarios of intracellular boron distributions; for these scenarios, pre-calculations of biological coefficients with NanOx for several cell lines; in each voxel of the patient CT scan, calculations by Monte Carlo simulations of the dose deposited by gammas, the energy spectra of recoil protons and the concentration of neutron captures by boron; and calculation of 3D biological dose distributions and TCP by combining the outcomes from the two previous stages. Comparisons will be done with existing BNCT formalisms and various neutron spectra. Preliminary results will be presented for simplified BNCT irradiation conditions.

### P 266 - A hybrid deterministic/probabilistic approach for beam angle optimization in IMPT

#### Humberto Rocha^1^, Joana Dias^1^

##### ^1^University of Coimbra, Faculty of Economics, Coimbra, Portugal

Appropriate selection of proton beam directions in intensity-modulated proton therapy (IMPT) may have a profound impact on the quality of dose distributions, particularly due to the small number and depth-dose characteristics of beams used in IMPT. However, most of the time, the beams are fixed a priori, and no beam angle optimization (BAO) is performed. BAO is a highly non-convex optimization problem that can be interpreted as the optimization of an expensive multi-modal black-box function. Deterministic direct-search methods have been successfully used to address the BAO problem, but with the shortcoming of being computationally time-consuming procedures. For proton therapy, the number of degrees of freedom for BAO is increased compared with IMRT, e.g., due to the availability of different levels of energy, making the already difficult task of obtaining optimal beam irradiation directions in a clinically acceptable time even more challenging. Recently, numerical results have shown that introducing randomization into otherwise deterministic BAO frameworks leads to very competitive results in terms of computational time. However, these stochastic approximations do not provide consistent quality solutions, which is a serious drawback when thinking of applying these approaches to IMPT treatment planning. In this study we propose the use of a direct-search hybrid approach that is competitive in terms of computational times by using probabilistic search directions and competitive in terms of consistently obtaining high-quality solutions by using deterministic search directions. The approach performance is assessed considering a prostate and a head-and-neck cancer case.

### P 267 - Calculations and comparison of the proton therapy doses obtained with the Geant4 and matRAD for proton therapy

#### Mariam Abuladze^1^, Levan Kankadze^1^, Akaki Lomia^1^, Revaz Shanidze^1^

##### ^1^Kutaisi International University, Hadron Therapy Center, Kutaisi, Georgia

Proton therapy is a high-quality radiation therapy that uses a proton beam to irradiate cancer tissue. The advantage of this type of treatment is a highly conformal dose deposition due to the presence of the Bragg peak. GEANT4 is the well-developed software platform for the simulation of the passage of particles through matter using Monte Carlo methods. It can calculate delivered doses with high accuracy, by considering all the possible particle matter interactions which can occur during proton passage into tissue. The treatment planning systems (TPS) which are used for calculation of delivered doses in radiation therapy are usually based on semi-analytical methods and hence are less precise than Monte-Carlo technique. MatRad is well-known open-source TPS software for intensity-modulated photon, proton, and carbon ion therapy, which is actively used for educational and research purposes. We have calculated proton doses for the selected phantoms with the Geant4 software and matRAD-TPS. Results obtained in these calculations, as well their comparison are presented in this conference contribution.

### P 268 - Ant colony algorithm for the Monte Carlo simulation of proton transport

#### Daniel Puerta^1^, Marta Anguiano^1^, Salvador García-Pareja^2^, Antonio M. Lallena^1^

##### ^1^University of Granada, Department of Atomic- Molecular and Nuclear Physics, Granada, Spain ^2^Hospital Regional Universitario de Málaga, Unidad de Gestión Clínica de Radiofísica Hospitalaria, Málaga, Spain

Protontherapy has established itself as a viable alternative to traditional radiotherapy for certain tumor and patient profiles. As such, highly accurate methods are required to assure the quality of the treatment. Monte Carlo simulations are considered the gold standard in these scenarios. However, the long times of calculation needed to achieve a result with acceptable statistical uncertainties make their implementation in the clinic a complex task. Variance reduction techniques (splitting, Rusian roulette, range rejection,…) provide a means to reduce such uncertainties, producing faster accurate simulations. However, their application is usually depending on the specific problem under investigation. Ant colony algorithms allow overcoming this drawback by applying the different variance reduction techniques considered on the base of importance maps that are build up automatically for each problem, leading to a more efficient simulation [1]. Previous implementations of these methods in electron and photon transport with the Monte Carlo code PENELOPE have been proven to be highly successful, reaching reductions in computation times of a factor of about 100 in some cases. In this work, we study these techniques applied to proton transport in the Monte Carlo code PENH, the hadron extension for PENELOPE [2].

### P 269 - Study of Hadron Beam Fragmentation with the FOOT experiment

#### Giuliana Galati^1^, Vincenzo Boccia^2^

##### ^1^Università di Bari Aldo Moro, Physics, Bari, Italy ^2^Università di Napoli Federico II, Physics, Naples, Italy

Particle therapy uses protons and heavy ions for the treatment of deep-seated solid tumors, but the biological damage due to beam-induced tissue fragmentation is still an open issue. Indeed, heavy ions undergo nuclear interactions leading to the production of nuclear fragments with high Relative Biological Effectiveness (RBE), thus representing an important source of biological damage. Nuclear fragmentation is also significant for proton therapy, where highly ionizing target fragments deal damage to healthy cells, particularly in the plateaux region. Effects linked to target fragmentation are hard to evaluate and they are neglected in clinical practice. Accurate data on fragmentation cross sections are thus needed to improve the current RBE models and the clinical treatment plans. The FOOT (FragmentatiOn Of Target) experiment measures nuclear fragmentation cross sections in the 50-700 MeV/A beam energy range with about 5% uncertainty. Target nuclei (16O,12C) fragmentation induced by proton beams is studied via an inverse kinematic approach employing 16O, 12C beams impinging on graphite and polyethylene targets. Two complementary setups are used: the “emulsion configuration” measures the production of light charged nuclear fragments (Z≤3), while the “electronic configuration” focuses on the heavier (Z≥3) fragments. This talk discusses the goals and status of the FOOT experiment, and the first cross section fragmentation results obtained by the two experimental setups with 200MeV/A and 400MeV/A 16O beam on C, C2H4 and H. The figure shows, as an example, the total reaction cross section and the total production one for 200MeV/A 16O beam on C, C2H4 and H using the “emulsion configuration”.

### P 270 - Investigation of a Monte Carlo proton pencil beam scanning model to support the commissioning of treatment planning systems

#### Daniela Botnariuc^1,2^, Steven Court^3^, Ana Lourenco^1,2^, Gary Royle^1^, Andrew Gosling^3^, Mohammad Hussein^2^, Vasilis Rompokos^3^, Catarina Veiga^1^

##### ^1^University College London, Medical Physics and Biomedical Engineering, London, United Kingdom ^2^National Physical Laboratory, Metrology for Medical Physics Centre, Teddington, United Kingdom ^3^University College London Hospitals NHS Foundation Trust, Radiotherapy Physics Services, London, United Kingdom

**Purpose**: We demonstrate the potential of Monte Carlo (MC) to complement the time-consuming and resource-intensive measurements that comprise the validation of the treatment planning system (TPS) of new proton therapy facilities.

**Methods and Materials**: A beam model of a pencil beam scanning system (Varian ProBeam) was developed in GATE (v8.2) using integral depth-dose (IDDs) curves in water and spot profiles in air. We compared point doses of two commercial TPSs, Eclipse proton convolution superposition algorithm (v16.1, Varian Medical Systems) and RayStation MC (v12.0.100.0, RaySearch Laboratories), against measurements or GATE for an extensive set of plans typically assessed during commissioning. This included spread-out Bragg peaks (SOBPs) with varying range, width and range-shifters (RSs) (n=39) and patient-specific quality assurance (PSQA) plans of different anatomical sites (n=6).

**Results and Discussion**: The GATE beam model was validated against basic experimental data (Tab.1). The Pearson’s correlation coefficient for differences between the TPSs and measurements and the TPSs and GATE was 0.91 and 0.80 for the SOBP and PSQA plans, respectively (Fig.1). The strong correlation indicates that TPS doses may be confidently compared with MC as an alternative to measurements. The standard deviation of the differences between GATE and measurements (±0.5% for SOBP and ±0.8% for PSQA plans) may be a suitable tolerance to include when comparing TPS with MC to identify plans that fail the established passing criteria.

**Conclusion**: Early development of a MC beam model, validated with basic commissioning data, may reduce the number of measurements required for TPS commissioning and facilitate comprehensive investigation of the TPS limitations.

### P 271 - Dose reconstruction methods using secondary prompt-gamma radiation in proton therapy

#### Beatrice Foglia^1^, Chiara Gianoli^1^, Takamitsu Masuda^2^, Thomas Bortfeld^3^, Joost Verburg^3^, Katia Parodi^1^, Marco Pinto^1^

##### ^1^Ludwig-Maximilians-Universität LMU- Munich, Chair of Experimental Physics - Medical Physics, Garching b. München, Germany ^2^National Institutes for Quantum Science and Technology QST, Department of Accelerator and Medical Physics, Chiba, Japan ^3^Massachusetts General Hospital MGH, Department of Radiation Oncology, Boston, USA

Full clinical exploitation of the advantageous interaction properties of protons compared to photons for radiotherapy photons for radiotherapy is limited by high sensitivity to range uncertainties. Prompt-gammas (PG), emitted after nuclear interactions of protons with the tissue, can be exploited for in vivo range verification and dose reconstruction. Different algorithms were proposed in the literature to reconstruct the dose from secondary emission, mostly from positron emitter distributions. These include the analytical deconvolution method[1], the evolutionary algorithm[2][3] and the maximum-likelihood expectation-maximization (MLEM) algorithm[4][5], whose differences are mainly related to the objective function and its optimization. They are based on the forward filtering approach, developed by Parodi and Bortfeld[6] for PET monitoring, and then extended to PG radiation by Schumann et al.[2] and by Pinto et al.[7]. Within this work, these approaches were applied to PG distributions generated from simulations of protons interacting with homogeneous and inhomogeneous phantoms (fig.1), and to in-silico clinical data. The final purpose is the comparison of the evaluated dose reconstruction approaches in view of their applicability to real-time adaptive particle therapy. Accuracy, computation time and integration into clinical workflow will be considered. In addition to in-silico simulation studies, first measurements have been conducted with a water-filled head phantom (RTsafe P.C., Athens, Greece) (fig.2) irradiated at the Francis H. Burr Proton Therapy Center at MGH (Boston, US). PG data were acquired with an in-house PG spectroscopy detector prototype[8]. Results of the ongoing data analysis will be presented. This work is performed as part of the RAPTOR project, funded the EU’s Horizon 2020 MSCA,G.A.No.955956.

### P 272 - The fetal dose for carbon beam craniospinal irradiation during pregnancy was investigated by a Monte Carlo study

#### Ji Won Choi^1^, Yongdo Yun^2^, Se Hyung Lee^3^, Choonsik Lee^4^, Min Cheol Han^2^, Yeon Soo Yeom^1^, Jin Sung Kim^2^

##### ^1^Yonsei University, Radiation Convergence Engineering, Wonju, Korea Republic of ^2^Yonsei Cancer Center- Heavy Ion Therapy Research Institute- Yonsei University College of Medicine, Radiation Oncology, Seoul, Korea Republic of ^3^Hanyang University, Nuclear Engineering, Seoul, Korea Republic of ^4^National Cancer Institute, Division of Cancer Epidemiology and Genetics, Maryland, USA

We investigated fetal organ doses from carbon beam craniospinal irradiation (CSI) during pregnancy by performing Monte Carlo (MC) dose calculations. We employed the high-quality pregnant female phantom series developed by University of Florida (UF) for eight gestational ages (8, 10, 15, 20, 25, 30, 35, and 38 weeks). The phantoms were converted into DICOM-RT CT images and implemented in a treatment planning system (TPS) with the prescribed dose of 36 Gy (RBE). A carbon CSI plan created from TPS was used to perform a TOPAS MC dose calculations after commissioned to the carbon beam measurement data. Fetal organ absorbed doses for 28 organs considered radiosensitive were calculated and compared with those estimated for proton therapy in a previous study. The fetal doses were within a range from 21 mGy (gall bladder) – 30 mGy (brain) at 8 weeks, 5 mGy (oesophagus) – 23 mGy (bladder) at 15 weeks, and 6 mGy (lung) – 31 mGy (small intestine) at 25 weeks. The fetal doses tended to decrease with increasing the gestational ages, which can be understood considering that the fetal organ distances to the beam edge are generally longer at older ages. The fetal doses from carbon therapy, although all lower than 100 mGy, were greater than the values previously calculated from proton therapy by up to 9.3 times (prostate) at 25 weeks. Considering the significant attention to carbon therapy, as a first dedicated effort, the result would be clinically informative to estimate the fetal dose from carbon therapy during pregnancy.

### P 273 - Fully automatic treatment planning for intensity-modulated proton therapy using machine learning and heuristic optimisation

#### Renato Bellotti^1^, Jonas Willmann^1^, Damien Weber^2^, Tony Lomax^1^, Andreas Adelmann^3^, Jan Hrbacek^1^

##### ^1^Paul Scherrer Institute, Centre for Proton Therapy, Villigen PSI, Switzerland ^2^University Hospital of Zürich, Department of Radiation Oncology, Zürich, Switzerland ^3^Paul Scherrer Institut, Laboratory for Simulation and Modelling, Villigen PSI, Switzerland

Two new methods to automate the treatment planning process are presented. The first one is a machine learning model for beam arrangement selection. Afterwards, a heuristic iterative algorithm searches objectives for our in-house spot optimiser. The entire treatment planning pipeline is fully automatic and does not require any patient-specific input apart from the CT, planning target volume (PTV) and organ at risk (OAR) contours, prescribed dose to the PTV and OAR constraints. The automatic planning system was trained on 80 patients and for 19 test patients compared to reference plans that have been accepted for treatment in our institute. The average and standard deviation of the difference for the PTV V95% was 2 ± 4% of the prescribed dose, the difference in dose to OARs is shown in Fig. 1. The mean and standard deviation of the auto-planning time are 25 ± 38min (maximum 142min). An analysis of V95% of the target and mean and maximum dose values of OARs and normal tissue combined with a visual assessment of the dose distributions established that 9 of the automatically generated plans are acceptable for treatment without modifications. 7 plans are considered acceptable with minor modifications such as reduction of hotspots to a small number of voxels, and only 3 plans failed to meet our acceptance criteria. However, autoplanning could still save time because manual fine tuning is possible. The shortcomings mainly stem from the underlying spot optimiser, which will be improved in future work.

### P 274 - Assessment of Geant4 nuclear cross-sections of proton-induced reactions for therapy

#### Marie Vanstalle^1^, Nicolas Arbor^1^, Christian Finck^1^, Arshiya Sood^1^

##### ^1^Université de Strasbourg- CNRS- IPHC, Department of Subatomic Research, Strasbourg, France

The renewed interest for protontherapy drives the necessity of the improvement of the treatment planning tools used for an accurate delivered dose calculation. In protontherapy, TPS are usually based on analytical modelling of the dose, but an increasing interest in the use of Monte Carlo simulation codes in these TPS is emerging. One of the main advantages of these codes is to account for the nuclear reactions undergone by the incident beam. These nuclear reactions are responsible for the production of many secondary particles, which will potentially contribute to an additional dose in the patient, and therefore need to be correctly estimated. However, numerous studies demonstrated that commonly used Monte Carlo codes do not always correctly reproduce these nuclear reactions, which are handled by hadronic models. Therefore, it is essential to quantify the influence of these hadronic models on the secondary particles. This work aims to assess the precision of several hadronic models of the Geant4 simulation code in reproducing nuclear reactions, by comparing existing data extracted from published studies with results obtained by simulation. Figure 1 shows an example of comparison between experimental and simulated cross-sections of the ^12^C(p,p’γ_4.44_)^12^C reaction (producing a 4.44 MeV gamma-ray), with different models available in Geant4 (INCL, the Bertini cascade, BIC, QBBC and ParticleHP). In addition to specific reactions cross-sections, comparisons of differential cross-sections at energies of interest for protontherapy will also be presented. It will be demonstrated that important discrepancies up to a factor 10 can be observed between simulations and measurements.

### P 275 - Material decomposition based calibration of single source DECT scanners in proton therapy

#### David Viar-Hernandez^1^, Juan Antonio Vera-Sanchez^2^, Lucia Schmidt-Santiago^1^, Borja Rodriguez-Vila^1^, Juan Maria Perez-Moreno^2^, Juan Castro-Novais^2^, Fernando Cerron-Campoo^2^, Alejandro Mazal^2^, Norberto Malpica^1^, Angel Torrado-Carvajal^1^

##### ^1^Universidad Rey Juan Carlos, Medical Image Analysis and Biometry Laboratory, Móstoles, Spain ^2^Centro de Protonterapia Quironsalud, Servicio de Fisica Medica, Madrid, Spain

In proton therapy, dual energy CT (DECT) calibration methods may be employed to derive Z-effective (Zeff) and electron density (ED) maps from Hounsfield Units (HU) low and high images, to achieve subsequently the Relative Stopping Power (SPR) maps. However, when using single source DECT (ssDECT), these low and high images are not generated, as only decomposition maps (DM) and Virtual Monochromatic Images (VMI) are provided. In order to compute the SPR maps, we have developed a novel method for directly calculating ED and Zeff maps based on the DM pair water-iodine. The method is divided into three steps: X-ray tube calibration, ED calibration, and Zeff calibration. In the first step, the mass attenuation coefficient for water and iodine at different energies is obtained either theoretically using the energy spectra of the CT scanner and the XCOM. In the second step, the relationship between the DM maps and ED values is established by fitting a linear combination of water and iodine densities to the ED of the material under study. In the final step, the Zeff is calculated using the Jackson and Hawkes formulation and the linear attenuation coefficient. Our method was tested using a GE REVOLUTION CT scanner, a GAMMEX multi-energy CT phantom, and protocols for both single energy CT (SECT) and ssDECT. The resulting ED and Zeff maps were compared to the ground truth and showed similar performance to existing DECT calibration methods. However, certain differences were observed for lung tissues, indicating a need for further development.

### P 276 - A simulation framework of the preclinical proton irradiation workflow

#### Justin Malimban^1^, Felix Ludwig^1^, Danny Lathouwers^2^, Marius Staring^3^, Frank Verhaegen^4^, Sytze Brandenburg^1^

##### ^1^University Medical Center Groningen, Department of Radiation Oncology and Particle Therapy Research Center, Groningen, Netherlands ^2^Delft University of Technology, Department of Radiation Science & Technology, Delft, Netherlands ^3^Leiden University Medical Center, Department of Radiology, Leiden, Netherlands ^4^Maastricht Medical Center, Department of Radiation Oncology MAASTRO- GROW School for Oncology and Developmental Biology, Maastricht, Netherlands

The size of orthotopic tumors in small animals (typically a few mm) presents some challenges in preclinical proton dose delivery. For tumors situated deeper in the animal close to critical organs, determination of the actual dose distribution and conformity is challenging especially for Bragg peak irradiations. Therefore, it is important to optimize beam properties, verify CT HU-RSP calibration, and ensure the quality of dose distributions. In this work, we present a simulation framework that (1) allows generation of realistic X-ray -CBCT images, (2) facilitates CT HU calibration, and (3) performs proton dose calculations. A μ-CBCT model was developed using the fastCAT toolkit. Monte Carlo simulations were performed to generate the primary and scatter kernels, and imaging dose calibration appropriate for μ-CBCT scans. CTs were then generated for a mini Gammex phantom and the MOBY/ROBY digital rodent phantoms. The HU – SPR conversion is performed with the mini Gammex phantom. The resulting calibration parameters are then used to convert the CTs of the MOBY/ROBY phantoms to SPR maps. These are then used to calculate dose distributions in TOPAS for treatment plans created in matRad using realistic beams based on measured emittances and simulations of the beam transport with BDSIM. Since the composition of the MOBY/ROBY phantoms is known, a ground truth exists against which the accuracy of the calibration and dose distributions can be verified. This framework is used to optimize the irradiation setup and assess the quality of small animal irradiations.

### P 277 - Impact of temporal image resolution on the interplay effect in 4D Monte Carlo dose calculation for IMPT

#### Ivar Bengtsson^1,2^, Erik Engwall^3^, Albin Fredriksson^2^, Lars Glimelius^3^

##### ^1^KTH Royal Institute of Technology, Mathematics, Stockholm, Sweden ^2^RaySearch Laboratories AB, Research, Stockholm, Sweden ^3^RaySearch Laboratories AB, Development, Stockholm, Sweden

The interplay between the beam delivery time structure and the patient motion makes 4D dose calculation (4DDC) important when treating moving tumors with IMPT. In a conventional 4DDC, each spot is assigned to its nearest phase image in a 4DCT, based on a delivery log and a breathing signal. The doses computed on the phase images are then deformed to a reference phase and accumulated. An apparent source of error in 4DDC is the approximation applied in the nearest neighbor assignment of spots to phases, due to the coarseness of the temporal image resolution. We addressed this limitation by applying registration-based interpolation between phase images for increased temporal resolution of the 4DCT. Assuming linear motion between the phases, we deformed each phase image to its neighbor using the deformation vector field that aligns them, scaled by the desired time step. We then applied Monte Carlo-based 4DDC to both the original 4DCT (360 ms), and extended 4DCTs at increasingly fine temporal resolutions (180 – 26 ms). The method was evaluated on a NSCLC patient treated with three robustly optimized IMPT beams with spot delivery times of approximately 3 ms and energy switching times of 2 s. At the original resolution, the D98 and D2 of the CTV are 2% and 1.5%, respectively, away from the values to which they converge for resolutions ≥ 60 ms (Figure 1). Taking the 4DDC at 26 ms as reference, gamma (3%/3mm) pass rates of 100% in the CTV were achieved at ≥ 72ms.

### P 278 - Comparison of beamlet-based and beamlet-free spot weight optimization for treatment planning in proton therapy

#### Danah Pross^1^, Sophie Wuyckens^1^, Kevin Souris^2^, John A Lee^1^

##### ^1^UCLouvain, Molecular Imaging Radiotherapy and Oncology MIRO, Brussels, Belgium ^2^Ion Beam Applications SA, Ion Beam Applications SA, Louvain-la-Neuve, Belgium

**Introduction**: Treatment planning in proton therapy typically involves separate precalculation of beamlet dose distributions and subsequent optimization of their modulation factors. The dose precalculation requires performing multiple Monte Carlo simulations and storing the resulting dose distributions in memory. This creates a high demand for time and memory which hinders the implementation of efficient workflows, especially with respect to robust optimization and Arc proton therapy where large numbers of beamlets are required. The beamlet-free method seeks to address this issue by combining dose calculation and optimization into a single, efficient algorithm.

**Methods**: The beamlet-free algorithm iteratively simulates small particle batches to randomly chosen spots and evaluates their impact on the objective function, which is then used to update the spot probabilities. It was implemented in C with stochastic-like gradient descent and it is compared against a conventional beamlet-based optimizer relying on the Scipy code for LBFGS[1] (gradient descent modulated by an approximate Hessian). Comparisons are made on a brain case.

**Results**: The optimization parameters are listed in Table 1 and the results are depicted in Figure 1. The beamlet-free method achieved comparable plan quality for a reduction of computation time by 56% from 2004+-84 s to 884+-85 s. Peak memory could be reduced by 71% from 4.902 GB to 1.422 GB.

**Conclusion**: The beamlet-free method is capable of maintaining adequate plan quality while significantly reducing time and memory requirements.

### P 279 - Time and memory efficient deterministic proton dose calculations using the dynamical low-rank approximation

#### Pia Stammer^1,2^, Niklas Wahl^2^, Danny Lathouwers^3^, Jonas Kusch^4^

##### ^1^Karlsruhe Institute of Technology KIT, Steinbuch Centre for Computing SCC, Eggenstein-Leopoldshafen, Germany ^2^German Cancer Research Centre - DKFZ, Department of Medical Physics in Radiation Oncology, Heidelberg, Germany ^3^TU Delft, Department of Radiation Science & Technology, Delft, Netherlands ^4^University of Innsbruck, Numerical Analysis and Scientific Computing, Innsbruck, Austria

Dose calculations based on the solution of the linear Boltzmann transport equation are often inefficient due to the necessary fine numerical discretizations of the six-dimensional phase space. Considering the continuous slowing down approximation (CSDA), one may approach the solution treating energy as pseudo-time. Within this framework, we then propose a dynamical low-rank approximation (DLRA). The DLRA decomposes large solution matrices into smaller, truncated factors of rank r. These factors are evolved more efficiently through time. Both runtime and memory are reduced compared to conventional Boltzmann solvers: For rank r approximations and n spatial/m angular nodes, computational complexity reduces from O(nm) to O((n+m)r^2^) and memory from O(nm) to O(nr). We investigate the extent of this complexity reduction for protons using a pencil beam in a three-dimensional waterbox with simplified physics, i.e., using the Rutherford formula and neglecting nuclear scattering and range straggling. We observe that an extremely low rank of r=3 is sufficient for a good agreement with TOPAS MC without nuclear scattering (compared to m=37^2^ in a full solver). In this case the computation lasts 4:13min in a julia implementation using GPUs. A possible explanation for the low rank is that for protons advection is the dominant effect, followed by diffusion. Since both are individually low rank, this is sufficient throughout the computation. Future work will verify rank requirements with an extended physical model and incorporate range-straggling within the DLRA solver. Further, the potential for direct uncertainty propagation using a higher-dimensional low-rank approximation could be explored.

### P 280 - Radiation-induced cancer risk from neutrons in pediatric proton therapy and field angle dependence

#### Johannes Tjelta^1,2^, Kristian Smeland Ytre-Hauge^2^, Andreas Handeland^1,2^, Erlend Lyngholm^2^, Helge Henjum^2^, Camilla Hanquist Stokkevåg^1,2^

##### ^1^Haukeland university hospital, Department of Oncology and Medical Physics, Bergen, Norway ^2^University of Bergen, Department of Physics and Technology, Bergen, Norway

**Introduction**: Out-of-field doses in proton therapy are associated with adverse late effects in tissues distant from the target. Children with brain tumors are more susceptible to radiation-induced cancers and are frequently referred to pencil beam scanning proton therapy. The aim of this study was to investigate how field configuration affected neutron production and risk of radiation-induced cancer.

**Method**: A pediatric brain tumor prescribed 54 Gy(RBE) was recalculated in the FLUKA Monte Carlo code to obtain absorbed and equivalent neutron doses on a full body CT image extending to the pelvis. Three plan configurations with a variable treatment field from posterior (0°), 45° or vertex (90°), combined with two fixed lateral fields (80° and 280°) were simulated. Equivalent doses were based on the ICRP103, U.S.NRC, Baiocco and Kellerer models, while excess absolute risk (EAR) and lifetime attributable risk (LAR) were estimated from the BEIR VII report.

**Results**: Risk calculations were done for neutrons. Estimated LAR for the whole patient and complete treatment was 1.1% (ICRP103). Lung LAR was reduced threefold between field angles 90° and 0° (Table 1). Organs further away from the target volume had both lower EAR and LAR (Table 1, Figure 1) while the brain LAR≈0.1% was relatively unaffected by field angles. All LARs were similar across the weighting models (Table 1).

**Conclusion**: The impact of field configuration on the neutron contribution were substantial for organs distant from the target volume, and less influential for brain tissues nearby the tumor. Different biological weighting models resulted in minor variation across the estimated risks.

### P 281 - A silicon-based neutron detector for monitoring secondary neutrons during carbon-ions therapycarbon-ions therapy

#### Mohammad Alsulimane ^1^

##### ^1^University of Liverpool, Department of Physics, Liverpool, United Kingdom

There has been a rapid worldwide increase in particle therapy centres over the last 20 years, including Proton and Carbon beam usage as a radiation treatment beam rather than conventional radiotherapy. This increase is driven by particle therapy’s advantageous dose distribution compared to treatment with photons. While the prescribed dose is delivered to the tumour with minimal entrance and exit dose to the surrounding healthy structures, one of the concerns particular to particle therapy is the secondary radiation, neutrons in specific, that is created during treatment as a result of particle interactions with human tissue. In this project, we are using Geant4 to simulate the carbon-ions beam and its interactions with a silicon-based neutron detector that is fully submerged in a water phantom volume (20 × 20 × 40cm^3^) and located in the main path of the beam along the Z direction. The system design involves a silicon sensor coated with ^6^LiF film as a thermal neutron converter layer to produce the thermal neutron capture phenomena and measure the neutrons generated in the phantom. The thermal neutrons assess and monitored at different points inside the phantom during delivering the prescribed dose to the targeted cells of carbon-ions beams. The Geant4 simulation system will provide a preliminary study of the detector response for neutron signals detection, assessment and discriminate it from background mixed radiation in a carbon-ions therapy environment.

### P 282 - Robust evaluation of clinically treated PTV-based proton and carbon ion plans-the real-world data

#### Yinxiangzi Sheng^1^, Lennart Volz^1^, Maria Chiara Martire^1^, Weiwei Wang^2^, Jiayao Sun^2^, Xue Ming^2^, Marco Durante^1^, Christian Graeff^1^, Guangru Li^3^

##### ^1^GSI Helmholtzzentrum für Schwerionenforschung GmbH, Biophysics, Darmstadt, Germany ^2^Shanghai Proton and Heavy Ion Center, Department of Medical Physics, Shanghai, China ^3^Institute of Modern Physics, Biomedical center, Lanzhou, China

**Purpose**: Robust optimization has been developed and proved effective in mitigating dose uncertainties. However, robust optimization may not be clinically available, particularly for pencil beam scanning carbon ion therapy, due to limited treatment planning systems. This work provides a quantitative estimate of the real-world robustness of clinically treated PTV-based carbon and proton plans.

**Methods**: Thirty-one (31) plans from 27 lung and tracheobronchial cancer patients were included. A planning 4DCT and two weekly repeated 4DCTs were collected. Plans were generated using Syngo (V13b, SIEMENS) with PTV-based optimization. PTV was derived from iCTV with an expansion of 5mm laterally and 3.5%+3mm in the beam directions. Low-density areas of iGTV were assigned as muscle. Plans were optimized with Single (SFO) or Multiple Field Optimization (MFO) strategies. Robust evaluation in 21 scenarios was performed using TRiP4D on planning CT (5mm/3%) and two review CTs (2mm/3%). The CTV D95% and Lungs-iGTV Dmean were evaluated.

**Results**: For the worst case scenario of the planning CT, the CTV D95% decreased −8.6%±7.1% and −6.5%±5.7% over all carbon and proton plans. Target D95% reduction of −7.8%±6.0% and −9.5%±9.6% were observed over all IMPT and SFO plans. Both differences not significant (p>0.05). The reduction of CTV D95% were −6.6%±8.7% and −11.2%±12.3% for the review CTs, respectively.

**Conclusion**: Real-world data are inherently inferior to data produced in dosimetric comparison studies. Target coverage is compromised to protect OARs. The dosimetric robustness superiority of SFO over IMPT has not been observed. Adapting to anatomical changes over the treatment course remains obligatory.

### P 283 - Study of physical parameters relevant in reference dosimetry for hadrontherapy: Stopping power and w-values in water and air

#### Verónica Belén Tessaro^1^, Jeremías Olivieri^2^, Benoit Gervais^3^, Michael Beuve^4^, Mariel Elisa Galassi^5^

##### ^1^Facultad de Ciencias Exactas- Ingeniería y Agrimensura de la Universidad Nacional de Rosario UNR, Instituto de Física Rosario CONICET-UNR, Rosario, Argentina ^2^Facultad de Ciencias Exactas- Ingeniería y Agrimensura de la Universidad Nacional de Rosario, Escuela de Ciencias Exactas, Rosario, Argentina ^3^Centre de Recherche sur les Ions- les Matériaux et la Photonique- CIMAP-CIRIL-Ganil, CEA/CNRS/ENSICAEN/Université de Caen-Basse Normandie UCBN, Caen, France ^4^Université de Lyon 1- CNRS/IN2P3, Institut de Physique des 2 Infinis de Lyon, Lyon, France ^5^Facultad de Ciencias Exactas- Ingeniería y Agrimensura de la Universidad Nacional de Rosario UNR, Instituto de Física de Rosario CONICET-UNR, Rosario, Argentina

Precise knowledge of the absorbed dose in liquid water is required in reference dosimetry. The IAEA international protocol TRS-398 [1], provides a methodology to determine the absorbed dose in water using air-filled ionization chambers. To convert the dosimeter reading from air to water, some factors such as the water-to-air stopping power ratio and the air w-values are required. The study of these physical parameters is relevant since they are the ones that contribute the most to the uncertainties in physical dose determination [1]. In this work, we present the stopping power and w-values in water and air for proton and carbon ions, calculated by applying two methods: an analytical one based on the Continuous Slowing Down Approximation (CSDA) [2] and the Monte Carlo simulation MDM [3]. Due to the range of energies used in hadrontherapy, a relativistic approximation is applied to the kinetic energy of the particles. The post-collision contribution of Auger electron emission is also included. The results obtained with both methods have a very good concordance between them. The stopping power shows a very good agreement with the recommended data [4] when applying the relativistic correction (CSDA-AR) (see Figure). The calculated w-values strongly depend on the Auger emission contribution and the excitation cross sections used.

### P 284 - Development of a mobile small animal irradiation device for preclinical research on proton therapy clinical systems

#### Richard Coos^1^, Frederic Stichelbaut^2^, Malgorzata Liszka^3^, Sébastien Penninckx^1,4^, Alexandru Dasu^3,5^, Stéphane Lucas^1,2^

##### ^1^UNamur, Physics, Namur, Belgium ^2^IBA Group, IBA Group, Louvain-La-Neuve, Belgium ^3^The Skandion Clinic, The Skandion Clinic, Uppsala, Sweden ^4^Institut Jules Bordet, Medical Physics Department, Brussels, Belgium ^5^Uppsala University, Uppsala University, Uppsala, Sweden

Within the framework of preclinical research in proton therapy, the university of Namur is developing a device allowing the irradiation of murine systems to be located on the couch of a proton therapy clinical installation. This device, particularly well-suited to single room proton therapy system allows the simultaneous irradiation of 6 mice. It is characterized by: its ability to maintain mice under gazeous anesthesia, avoiding forced immobilization of the animals; the possibility to irradiate several mice simultaneously and not one by one as it is usually the case in research setups, reducing the experimental variability coming from the physics of the radiation; compatible with usual proton therapy protocols but also to perform FLASH study using pass-through or conformal modes; and compatible with clinical CBCT systems enabling in-room imaging and the integration in the associated TPS. In this lecture, we will present the last developments related to: tightness of the device with respect to animal excrements; long-term anesthesia without release of anesthetic gas into the clinical room; versatility of collimation systems to target tumors in supine and prone positions; homogeneous isoflurane (anaesthetic gas) distribution within mice population; use of Monte-Carlo simulation tools (MCNPX & Fispact-2) to evaluate the in-depth and lateral dose distribution and the associated experimental validations; and evaluation of activation of device post irradiation. The device was validated from a physical and animal handling point of view. Its use in a clinical room has been validated by the Commission for Hospital Hygiene and Infection Prevention of a Belgian hospital.

### P 285 - Irradiation of a synthetic bone doped with iodine for proton range verification

#### Sílvia Viñals^1^, Raúl Magro^1^, Miguel Manso^2^, José Antonio Briz^3^, Andrea Espinosa-Rodríguez^3^, Luis Mario Fraile^3^, Vicente García-Tavora^4^, Nil Mont^5^, Enrique Nácher^6^, Amanda Nerio^4^, Víctor Valladolid Onecha^3^, Max Pallàs^5^, Ariel Tarifeño^7^, Olof Tengblad^4^

##### ^1^Universidad Autónoma de Madrid, Centro de Micro-Análisis de Materiales CMAM, Madrid, Spain ^2^Universidad Autónoma de Madrid, Departamento de Física Aplicada, Madrid, Spain ^3^Universidad Complutense de Madrid, Grupo de física nuclear, Madrid, Spain ^4^Consejo Superior de Investigaciones Científicas CSIC, Instituto de Estructura de la Materia, Madrid, Spain ^5^Universitat Politècnica de Catalunya, Institut de Tècniques Energètiques, Cataluña, Spain ^6^Consejo Superior de Investigaciones Científicas CSIC, Institut de Física Corpuscular, Valencia, Spain ^7^Consejo Superior de Investigaciones Científicas CSIC, Institut de Física Corpuscular, Madrid, Spain

One of the main uncertainties associated with proton treatment is the deposited dose derived from the reach of protons in the tissue, increased moreover in the case of bones. One of the most attractive approaches to proton range verification is the use of biocompatible contrast agents that, once irradiated, emit different particles in response to the activation of the contrast agent. Iodine has a low energy threshold to open the activation channel, a large effective section (200 mbar at 10 MeV [1]) and, in addition, is routinely used as a contrast agent for various medical procedures. The induced nuclear reaction with protons is 127I(p,n)127mXe and decays by gamma deexcitation with two characteristic rays of 124.7 keV and 172.4 keV [2]. These gamma rays are emitted with a half-life short enough to assess activation during irradiation. In this work, the authors have synthesized and characterized four samples of iodinated hidroxyapatite at different concentrations in order to test the ability of 127I as a contrast agent for pT treatments in osteosarcoma. The concentration of iodine in each sample has been obtained through PIXE analysis and they contain, respectively, 0%, 0.8(1)%, 3.6(1)% and 6.0(1)%. From the preliminary results obtained so far, it can be stated that with an iodine concentration of 6%, the activation is clearly seen (Fig. 1). In the case of a concentration of iodine below 1%, the activation is still detectable (Fig. 2).

### P 286 - Calculation of beam quality correction factors for proton beams using Gate/Geant4

#### Guillaume Houyoux^1^, Nick Reynaert^1^, Séverine Rossomme^2^, Kevin Souris^2^, Sébastien Penninckx^1^

##### ^1^Institut Jules Bordet - Université Libre de Bruxelles, Medical Physics Department, Brussels, Belgium ^2^Ion Beam Application, Dosimetry, Louvain-La-Neuve, Belgium

**Introduction/Aim**: An effective way to calculate the beam quality correction factor k_Q of ionization chambers (IC) is to use Monte Carlo (MC) simulations where detailed modelling of the IC can be performed. The aim of this study is to compare the calculation of k_Q for mono-energetic proton beams by the MC code GATE/GEANT4 with other MC codes such as PENH, FLUKA and TOPAS/GEANT4.

**Materials and Methods**: We considered two plane-parallel IC IBA-PPC05 and IBA-PPC40. Their k_Q factors were determined by calculating the absorbed dose to water and the average absorbed dose to air in the sensitive volume of the IC. Both quantities were simulated for the Cobalt 60 photon spectrum and for mono-energetic proton beams with energies between 100 MeV to 250 MeV. We accepted a statistical uncertainty of 0.5% on the k_Q correction factor.

**Results**: For the IC PPC05, we obtain k_Q agreeing within 1% with the 3 other MC codes for each proton energy considered. For the IC PPC40, we obtain k_Q following the results determined with TOPAS/Geant4 and FLUKA within 1%. We observe that PENH behaves differently and tends to diverge as the energy increases, reaching a 2% deviation at 250 MeV. These results suggest the non-negligible role of nuclear interactions as they are differently implemented in the different codes.

**Projection**: Following this work, we aim to go further on the impact of nuclear interactions on the k_Q, studying heavier ion beams such as carbon ions.

### P 287 - Translating Nitrogen-12 real-time in vivo verification for proton therapy to the clinic using Monte Carlo simulation

#### Zahra Ahmadi Ganjeh^1^, Brian Zapien-Campos^1^, Stefan Both^1^, Peter Dendooven^1^

##### ^1^Particle Therapy Research Center PARTREC, University Medical Center Groningen, Groningen, Netherlands

The RIVER project at the Particle Therapy Research Center (PARTREC) is investigating imaging of the very short-lived positron-emitting nuclide N-12 (half-life of only 11 milliseconds), produced in the patient by the proton therapeutic beam, for real-time in vivo verification. The focus of the study is head and neck cancer which, typically, involves numerous healthy organs in close proximity to the tumor. The full clinical workflow is realistically mimicked using an anthropomorphic head phantom, making use of both experiments and Monte Carlo simulations. The simulation setup based on the experimental setup consisting of a CIRS 731-HN anthropomorphic head phantom and a PET scanner is shown in Fig. 1. A CT image of the head phantom is imported into a custom-modified research version of the RayStation treatment planning system. The PET image that results from irradiation is simulated using the GATE Monte Carlo simulation platform. Input to the simulation are the CT image of the phantom, the distribution of N-12 and other, longer-lived, positron emitters from RayStation, and the characteristics of our PET scanner. Experiments using homogeneous phantoms are used to validate the RayStation outcome. The performance for point sources was simulated and compared with measurements. The RIVER project aims to make a major step towards translating N-12 real-time in vivo verification for proton therapy to the clinic.

### P 288 - PETITION (PET for intensive care units and innovative protontherapy) scanner for proton therapy applications

#### Carla Winterhalter^1^, Keegan McNamara^1^, Shubhangi Makkar^1^, Günther Dissertori^2^, Christian Ritzer^2^, Erik Roelofs^3^, Antoni Rucinski^4^, Damien Charles Weber^1^, Catharina M.L. Zegers^3^, Antony Lomax^1^

##### ^1^Paul Scherrer Institut, Center for Proton Therapy, Villigen PSI, Switzerland ^2^ETH Zürich, Department of Physics, Zürich, Switzerland ^3^GROW School for Oncology Maastricht University Medical Centre+, Department of Radiation Oncology Maastro, Maastricht, Netherlands ^4^Polish Academy of Sciences, Institute of Nuclear Physics, Kraków, Poland

The PETITION scanner is a rotatable, open-ring, on-table PET scanner for proton therapy, which is currently being developed (figure 1). Two applications are foreseen (figure 2): Recording hypoxia information just before irradiation to potentially adapt the treatment plan (*hypoxia-based adaptive proton therapy*), and *online/offline range verification* during/after treatment. The feasibility and potential impact of *hypoxia adapted treatments* has been evaluated within a retrospective treatment planning study. For 7 patients, treatment plans have been created based on time-varying hypoxia data (taken twice during treatment, average boosting of dose to the hypoxic tumour volume 12%), showing that on average TCP increases by 6.9% (2.9%-11.0%) while NTCP decreases on average by 1.7%/4.8% (xerostomia/mucositis) compared to conventional photon plans. To evaluate the potential for *online/offline range verification*, activation calculations have been implemented in a GPU based Monte Carlo toolkit (average calculation time within the patient geometry 2.9 minutes) and a dedicated reconstruction toolkit has been developed, which takes into account a rotating scanner geometry. This allows to record and analyse short-lived isotopes during treatment (^12^N, 90% after first energy layer) for online range verification, and long-lived isotopes for offline range verification (^15^O, 55% after final energy layer). In summary, the PETITION scanner is a dedicated, on-table system for proton therapy. Treatment planning and simulation results indicate that hypoxia-based treatment plans might lead to a better treatment outcome, and that this scanner geometry might allow for both online and offline range verification. The scanner is currently being assembled and measurements are planned for January 2023.

### P 289 - Noise analysis in X-ray FPD image during proton beam irradiation for real-time tumor tracking

#### Toshiyuki Terunuma^1,2^, Shunsuke Moriya^1,2^, Mayu Osugi^3^, Masanori Takamiya^3^, Suzuka Asano^3^, Naoki Miyamoto^4^, Seishin Takao^4^, Takeji Sakae^1,2^

##### ^1^University of Tsukuba, Faculty of Medicine, Tsukuba, Japan ^2^University of Tsukuba Hospital, Proton Medical Research Center, Tsukuba, Japan ^3^Hitachi Ltd., Healthcare Business Division, Kashiwa, Japan ^4^Hokkaido University, Faculty of Engineering, Sapporo, Japan

**Purpose**: Analysis of noise caused by proton beam irradiation on X-ray flat panel detectors (FPD) is important because the noise may degrade the accuracy of real-time tumor tracking in X-ray fluoroscopic images. In particular, noise modeling and adding it to the training images in advance is essential for marker-less tumor tracking performed by deep learning. The purpose of this study is to model the noise during proton beam scanning irradiation.

**Methods**: A phantom test was performed to analyze the noise, i.e., the high-brightness pixel values, in X-ray images acquired by an FPD during proton beam irradiation. For comparison, X-ray fluoroscopic images were acquired under three conditions: 1) during proton beam scanning irradiation, 2) at intervals of proton beam scanning irradiation, and 3) X-ray imaging only.

**Results**: The amount of noise due to proton beam irradiation was less than 1% of the total pixels in the X-ray fluoroscopic images. Histogram analysis of pixel values revealed an exponentially decreasing relationship between the noise brightness and the number of pixels. These results allow us to model the noise during proton beam irradiation as a random event.

**Conclusion**: Noise can be modeled as a random event with the measured exponentially decreasing relationship. This model can be applied to reproduce noise in deep-learning training images, which will lead to robust real-time tumor tracking.

### P 290 - Development of SGRT and IGRT phantom using Silicone and gypsum for a highly accurate patient positioning in radiation therapy

#### Jeongho Kim^1^, JeeHoon Park^1^, Tea Gyu Kim^1^, Byung Do Park^1^

##### ^1^Samsung Chanwon Hospital- Sungkunkwan University School of Medicine, Department of Radition Oncology, Chanwon, Korea Republic of

In recent years, the SGRT method has been developed and used to improve the accuracy of radiotherapy. This is also important in particle therapy. In this research, we developed a SGRT and IGRT phantom and evaluated its characteristics. This phantom is composed of silicone and gypsum. Soft tissue was mimicked with silicone (HU 150) and bone with gypsum (HU 600-1000) (Fig. 1). For the fabricated phantom, treatment planning was performed using the Eclipse planning system after obtaining CT images with the philips CT. The verification of phantom was performed using TrueBeam STx and the Exactrac dynamic. The phantom were positioned using SGRT system. In addition, after patient positioning, the patient was shifted (lateral, longitudinal, vertical) from 1 to 3 cm at 1 cm intervals. CBCT images and OBI (on board image) were obtained and compared with Planning CT after patient positioning and shift positioning (Fig. 2). As a result, the mean difference between CBCT images and Planning CT was 0.4 (0.1-0.9, lateral), 0.7 (0.5-0.9, longitudinal), and 0.3 (0.2-0.5, vertical) mm, and the average difference between OBI and Planning CT was 0.3 (0.1-0.6, lateral), 0.4 (0.1-0.6, longitudinal), 0.5 (0.4-0.6, vertical) mm. In conclusion, we confirmed that the difference in all directions is less than 1 mm, and confirmed that the phantom developed in this study can be used for simultaneous verification of the SGRT system and the IGRT system. In addition, it has a texture similar to that of the human body, and is thought to be useful for training workers.

### P 291 - Dose distribution of Magnetic Resonance-integrated Proton Therapy in space before and after correction

#### Guodong Li^1^, Ming Wang^1^, Lei Zhang^1^

##### ^1^Chengdu university of technology, College of Nuclear Technology and Automation Engineering, Chegndu, China

Magnetic Resonance-integrated Proton Therapy(MRiPT) beam delivery technology can improve the dose delivery precision, but the disturbance of proton beam transport by magnetic field will change the dose distribution in the body. Therefore, the beam trajectory correction in magnetic field and the optimize the body dose distribution are of great importance for the MRiPT study. In this study, TOPAS and matRad software systems were used to analyze the disturbance of dose distribution in the body under non-uniform magnetic field. Firstly, a beam trajectory deflection model of proton beam in inhomogeneous magnetic field and inhomogeneous medium is established, and then a beam trajectory correction algorithm is developed and verified. The spatial radiation dose distribution in the phantom and liver tumor model before and after correction was analyzed. The results showed that in the phantom, the passage rate of γ in the target area was 85.1% (The reference plan pass rate was 92.1%) after correction at 3%/3mm standard, and the total dose in the non-target area increased by 0.12% compared with the reference plan. In the liver tumor model, the passage rate of target γ was 80.8% (89.3% under the reference plan) after correction at 3%/3mm, and the total dose in the non-target area increased by 0.38%. The results show that the beam trajectory correction algorithm can optimize the target dose distribution under magnetic field, but the γ passing rate needs to be improved. The results also show that the irradiation dose in the non-target area increases when the magnetic field is present.

### P 292 - Evaluation of noise robustness in patient-specific deep learning using orthogonal X-ray fluoroscopic images for markerless tumor tracking

#### Fumiaki Komatsu^1,2^, Toshiyuki Terumuma^3^, Shunsuke Moriya^3^, Takeji Sakae^3^

##### ^1^University of Tsukuba Hospital, Proton Medical Research Center, Tsukuba, Japan ^2^Doctoral Program in Medical Sciences Degree Programs in Comprehensive Human Sciences Graduate School of Comprehensive Human Sciences, Division of Medical Physics, Tskuba, Japan ^3^Faculty of Medicine, University of Tsukuba, Tskuba, Japan

**Purpose**: We reported patient-specific deep learning (DL), which trains digitally reconstructed radiographs (DRRs) and identifies target positions and shapes on kilovolt (kV) x-ray fluoroscopic (XF) images for real-time markerless tumor tracking radiotherapy. Because this method uses XF, the additional dose must be as low as reasonably achievable. However, the robustness of this method under low dose conditions, i.e., noise robustness, has not been fully investigated. The purpose of this study is to improve the conventional convolutional neural network (CNN) model and demonstrate its noise robustness in clinical XF simulations.

**Methods**: Our previous method requires two independent CNN models for each orthogonal XF image, i.e., one model accepts one channel of image input (1XF-CNN). In contrast, the newly proposed CNN model can accept two channels of image input (2XF-CNN). With this model improvement, we can expect that the proposed 2XF-CNN is more robust to noise because the pair of orthogonal XF images contains twice as much information as a single XF image. Robustness to noise was confirmed using clinical XFs with artificially injected noise.

**Results**: The tumor tracking accuracy obtained with the conventional 1XF-CNN model showed a breakdown as the noise intensity of the XF images increased. In contrast, the proposed 2XF-CNN model was shown to be able to track tumor contours despite the increase in noise.

**Conclusion**: We confirmed that the proposed 2XF-CNN demonstrated more robustness to noise in clinical XFs than 1XF-CNN. Proposed 2XF-CNN has the potential to provide robust real-time markerless tumor tracking radiotherapy.

### P 293 - Patterns of practice of image-guided particle therapy for extremities: A site-specific multi-institutional survey of the European Particle Therapy Network

#### Lorenzo Placidi^1^, Juliette Thariat^2^, Aswin Hoffmann^3^, Alessandra Bolsi^4^

##### ^1^Fondazione Policlinico Universitario Agosrino Gemelli IRCCS, Medical Physics Department, Rome, Italy ^2^Center Baclesse- ARCHADE, Radiation Oncology, Caen, France ^3^University Hospital Carl Gustav Carus, Proton Therapy Center, Dresden, Germany ^4^Paul Scherrer Institute, Center for Proton Therapy, Villigen, Switzerland

Particle therapy is clinically employed for extremities treatment but a shared and common consensus on the image-guided particle therapy (IGPT) methods is still not available. The aim of this european multi-institutional survey is to assess the patterns of practice of IGPT for extremities. Eight centres provided feedback on the extremities survey, five of which treat patients. Most of the centres are using SECT for treatment planning, and only very few routinely are using DECT. When anatomical changes are detected and replanning is required, this will be based on a repeated CT acquisition, with the same nominal planning CT scanner and protocol settings. MR imaging is used mainly for delineation purposes: in half of the cases, MR acquisition is performed with specific sequences for radiation therapy and with the extremities in the treatment position, minimising uncertainties in the registration. Daily image guidance is mostly based on 2D X-rays vs DRR match based on bone anatomy. Surface imaging is rarely used as well as CBCT imaging modality. Intra-fraction monitoring using post-treatment control images is rarely performed. Treatment of extremities, despite being performed in multiple centres, does not involve large patient population, which is normally limited to max 10-20 patients per centre. Specific literature is scarce on this topic, therefore most of the centres base their IGPT workflow on their own experience. The centres highlight the need of a more streamlined IGPT process, including solutions for images automatic registration and surface imaging. Standardization of IGPT clinical practice requires guidelines, as suggested by survey participants.

### P 294 - Investigation of uncertainty maps to assess deep learning-based synthetic CT quality for adaptive proton therapy

#### Arthur Galapon^1^, Adrian Thummerer^1^, Johannes Langendijk^1^, Dirk Wagenaar^1^, Stefan Both^1^

##### ^1^University Medical Center Groningen, Radiotherapy, Groningen, Netherlands

**Purpose**: To investigate if uncertainty maps (UM) based on Monte Carlo dropout (MCD) can be utilized to assess the accuracy of MRI-based sCTs in the absence of ground truth CT images.

**Materials and Methods**: A UNET-based DCNN model was trained using 101 registered CT and MR images (71 training, 10 validation, 20 test). sCT images and UMs were generated by repeated inference with activated dropout layers. A final sCT was obtained by averaging all inferred sCTs. The UM was obtained from the variance of inferences. The resulting UMs were compared to various metrics, including the HU error, range error, WET maps, dose difference, and gamma maps. A 2D UM and HU error map was generated by projection along the 90^°^-angle to facilitate a comparison with range error and WET maps. To evaluate against the dose distribution, the UMs were projected along the clinical beam angles. For the dosimetric evaluation, a mask was applied to only include voxels along the beam paths. Pearson’s correlation coefficient was calculated between UM and all metrics to quantify correlation.

**Results**: Figure 1a shows a generated UM, projected error map, proton range difference, and WET difference. Figure-1b shows the UM map compared to the dose difference and gamma map. Figure-2 shows the correlation coefficient between UM and the various metrics evaluated. On average, HU error shows the highest correlation with r=0.90 ± 0.03.

**Conclusion**: The observed correlations show the potential to use UMs as a QA tool for the HU-accuracy of DL-based sCTs. Dose-based metrics showed a lower correlation with uncertainty maps.

### P 295 - Patterns of practice of image-guided particle therapy for brain tumors: A site-specific multi-institutional survey of the European Particle Therapy Network

#### Iuliana Toma-Dasu^1^, Dante Amelio^2^, Alexandru Dasu^3^, Alessandra Bolsi^4^, Aswin Hoffmann^5^

##### ^1^Karolinska Institutet, Medical Radiation Physics, Stockholm, Sweden ^2^Proton Therapy Center, Azienda Provinciale per i Servizi Sanitari Trento, Trento, Italy ^3^Skandion Clinic, n/a, Uppsala, Sweden ^4^Paul Scherrer Institute, Center for Proton Therapy, Villigen, Switzerland ^5^University Hospital Carl Gustav Carus, Proton Therapy Center, Dresden, Germany

The well-known advantages of particle therapy for brain tumours rely on accurate image guidance in all treatment phases to mitigate range and positioning uncertainties, which might affect the accuracy of the delivered dose. The patterns of practice of Image Guidance Particle Therapy (IGPT) for brain tumours have been addressed in this European multi-institutional survey. The results include the responses of six treating centres, which have based their IGPT workflow both on published data and on their specific experience and available tools. SECT imaging is mostly used for planning CT acquisitions, whilst DECT is used in two institutes. All centres use MR imaging (and RT-specific pulse sequences) for tumour delineation purposes; in most cases the MR acquisitions are not performed in treatment position and they are rigidly registered with the planning CT scans. Some centres repeat MR image acquisitions during the treatment course, mainly to assess treatment response and anatomical changes. PET acquisitions are performed in the treatment preparation phase, and rigidly registered with the planning CT scan. Daily pre-treatment IGPT is generally based on 2D X-rays images matched with DRR, whilst 3D images (CBCT) are acquired in very few centres on regular intervals (either daily or weekly). In half of the centres specific IGPT protocols are defined for paediatric patients with the goal of reducing dose from image guidance. All centres indicate that IGPT for brain tumours would benefit from improvements in the tools and software for image guidance and offline review, to further increase treatment efficiency and accuracy.

### P 296 - 3D imaging of inter-fraction morphological variations using secondary fragments in 12C ion therapy

#### Giacomo Traini^1^, Angelica De Gregorio^2^, Micol De Simoni^3^, Gaia Franciosini^2^, Yunsheng Dong^4^, Riccardo Mirabelli^2^, Annalisa Muscato^5^, Antonio Trigilio^2^, Viviana Vitolo^6^, Alessio Sarti^7^

##### ^1^INFN - Section of Rome, -, Rome, Italy ^2^Sapienza Università di Roma, Physics, Rome, Italy ^3^Ludwig Maximilians Universität München LMU Munich, Département of Medical Physics, Munich, Germany ^4^INFN, Section of Milan, Milan, Italy ^5^Sapienza Università di Roma, Scuola di specializzazione in fisica medica-, rome, Italy ^6^CNAO, Centro Nazionale di Adroterapia Oncologica, Pavia, Italy ^7^Sapienza Università di Roma, Dipartimento di Scienze di Base e applicate per l’Ingegneria SBAI, Rome, Italy

One of the challenges currently faced when planning Carbon Ion Radio Therapy treatments is the proper accounting for possible morphological changes occurring in the patient body during the treatment delivery. To avoid that the ballistic precision of carbon ions in targeting the tumour volume could be spoiled by a morphological change, causing an underdosage of the target volume and reducing the treatment efficacy, safety margins are implemented at the planning stage. Re-evaluation CTs are generally performed in the middle of the treatment, to check possible dose distribution variations both in the target or in the surrounding organs, to re-optimize, if needed, the plan. The Dose Profiler is a device designed to operate as an online treatment verification system, capable of reconstructing the 3D emission maps of charged particles produced by the interaction of the ion beams with the patient tissues. We developed a method to identify morphological variations based on the comparison of the reconstructed maps collected in different fractions by means of a gamma test. Such tools aim to indicate the need of a re-evaluation CT for shallow morphological changes that could significantly alter the absorbed dose distribution and reduce the safety factors implemented during the planning. The proposed method is under test at the CNAO by the INSIDE collaboration in the context of a clinical trial (ClinicalTrials.gov Identifier: NCT03662373) in which 20 patients will be enrolled. The in-vivo performance of the technique will be presented and the results discussed in the context of CIRT online monitoring and planning.

### P 297 - Patterns of practice of image-guided particle-therapy for prostate cancer: A site-specific multi-institutional survey of the European Particle Therapy Network

#### Markus Stock^1^, Christian Reschl^2^, Alessandra Bolsi^3^, Aswin Hoffmann^4^

##### ^1^MedAustron Ion Therapy Centre, Medical Physics, Wiener Neustadt, Austria ^2^MedAustron Ion Therapy Centre, Radiation Therapy Technology, Wiener Neustadt, Austria ^3^Paul Scherrer Institute, Medical Physics, Villigen, Switzerland ^4^University Hospital Carl Gustav Carus, Proton Therapy Centre, Dresden, Germany

On behalf of EPTN, a survey on image-guided particle-therapy (IGPT) was sent to European particle therapy centres to understand current practice parameters of IGPT for prostate cancer. Response data from 8 treating centres was collected and showed that number of patients treated with particle-therapy for prostate is low but increasing, with the majority of centres treating less than 50 patients per year. 50% of centres use thermoplastic masks for immobilization. Most centres treat patients in supine position with knee support and rectum and/or bladder stabilization procedures. Endorectal balloons are routinely or optionally used by 50% of centres, whereas rectal spacers are optionally used by a majority of centres. For treatment planning, still SECT is in routine use in all centres. 66% of centres use routinely or optional some kind of metal artifact correction algorithms All centres use MRI for contouring and rigid MRI-to-CT registration is standardly performed. In 50% of the centres the MRI scan is acquired in treatment position. PET imaging is mainly an option for primarily staging and target volume delineation. For patient positioning, the daily setup procedure is performed predominantly inside the treatment room. All centres acquire CT control scans during the course of treatment to evaluate the need for re-planning, which is routinely based on CT or optionally on CBCT imaging. 2D IGPT is routinely used and performed before each treatment fraction and mainly matched on fiducial markers if available. 3D IGPT is seldomly used but increasing. Post-treatment images are not routinely acquired in most centres.

### P 298 - Stability testing of a commercially available us-tracking system in a spot scanning proton delivery environment

#### Martin Szegedi^1^, Vikren Sarkar^1^, Sara St. James^1^, Adam Paxton^1^, Ryan Price^1^, Christian Dial^1^, Jessica Huang^1^, Hui Zhao^1^, Bill J. Salter^1^

##### ^1^University of Utah, Radiation Oncology, Salt Lake City, USA

Pencil beam scanned (PBS) proton therapy is sensitive to positioning variation as well as target motion. Optical surface tracking has been used as a surrogate to position and monitor patient movement. A more direct non-invasive approach can be Ultrasound-Image Guidance (USIG), enabling tracking of an actual target volume. To understand the feasibility of USIG use in a proton-vault, we have placed a commercially available USIG system into a single-room PBS-vault. Equipment in such a vault does generally not receive direct proton radiation damage, however secondary neutrons will interact with equipment. The USIG system was exposed for 2 years to the neutron scatter of the PBS-vault as well as neutron radiation from phantom prostate treatments. We recorded the amount of n-radiation generated by the PBS system with the USIG off and during phantom treatments with personal gamma/neutron dosimetry monitoring devices on three critical components of the system, namely the US-probe-crystal, the optical tracking component and the computer-based tracking components. We have not observed image-distortion or tracking accuracy degradation since the exposure of the USIG system to the proton vault environment. Comparison of images between phantom treatments or during tracking periods were without differences. We confirm that the USIG system can be routinely used in a a single room proton vault where neutrons are present to conduct positioning and tracking, with no observable image degradation from the interaction with the neutron radiation. Future use on patients will allow for a systematic clinical evaluation of positioning and tracking abilities in prostate proton patients.

### P 299 - A novel personalized adaptive patient pre-alignment strategy to mitigate systematic positioning inaccuracies before setup verification in image-guided pediatric particle therapy

#### Matteo Pepa^1^, Andrea Pella^1^, Giulia Sellaro^1^, Alfredo Mirandola^2^, Angelica Ghirelli^3^, Sabina Vennarini^4^, Sara Imparato^3^, Mario Ciocca^2^, Ester Orlandi^3^, Guido Baroni^1,5^

##### ^1^CNAO National Center for Oncological Hadrontherapy, Bioengineering Unit- Clinical Department, Pavia, Italy ^2^CNAO National Center for Oncological Hadrontherapy, Medical Physics Unit - Clinical Department, Pavia, Italy ^3^CNAO National Center for Oncological Hadrontherapy, Clinical Department, Pavia, Italy ^4^Fondazione IRCCS Istituto Nazionale dei Tumori, Pediatric Radiotherapy Unit, Milan, Italy ^5^Politecnico di Milano, Department of Electronics- Information and Bioengineering, Milan, Italy

**Purpose**: Purpose of this retrospective study was to propose a novel personalised adaptive pediatric patient pre-alignment strategy that exploits information of previous treatment fractions to identify and mitigate systematic positioning inaccuracies in image-guided particle therapy.

**Methods**: Correction vectors (V) guiding a 6-DOF treatment couch resulting from rigid registration between daily in-room X-ray images and digital reconstructed radiographs from simulation CT were collected. For each fraction, an adjusted correction vector (V’) was computed as the difference between the actual one (V) and the algebraic average of the previous ones – simulating patient pre-alignment before imaging accounting for information from previous fractions. The Euclidean norm of each V’ was computed and normalized with respect to that of the corresponding V to derive N (Fig. 1). For each fraction, an average value across all patients was considered.

**Results**: Thirty-two pediatric patients (particle: 31 protons, 1 carbon ions; district: 22 head, 8 thorax/pelvis/limbs) and 853 fractions were considered. Pre-correcting all the coordinate values led to a 35% average reduction (min 20%, max 40%) in the magnitude of the correction vectors, considering the first 27 fractions (average value in this cohort of patients) (Fig. 2, down). If considering patients singularly, there were 29/853 outlier cases (3.4%) with 1 < N < 2 and 4/853 (0.5%) with N > 2 (Fig. 2, up).

**Conclusions**: Results demonstrate the validity of such strategy to ameliorate patient setup during CT planning phase and initial patient positioning. Further investigations to identify patients most likely to benefit from this approach are needed.

### P 300 - The small animal imaging and irradiation device SmART+ IB is being established for preclinical image-guided proton therapy experiments

#### Moritz Schneider^1,2^, Joshua Schilz^1,2^, Felix Horst^2,3^, Sebastian Gantz^2,3^, Elisabeth Bodenstein^2,3^, Michael Schürer^2,4^, Elke Beyreuther^1,2^

##### ^1^Helmholtz-Zentrum Dresden-Rossendorf, Institute of Radiation Physics, Dresden, Germany ^2^OncoRay, National Center for Radiation Research in Oncology - Faculty of Medicine and University Hospital Carl Gustav Carus - Technische Universität Dresden - Helmholtz-Zentrum Dresden-Rossendorf, Dresden, Germany ^3^Helmholtz-Zentrum Dresden-Rossendorf, Institute of Radiooncology - OncoRay, Dresden, Germany ^4^National Center for Tumor Diseases Dresden NCT/UCC, Germany: German Cancer Research Center DKFZ- Heidelberg- Germany- Faculty of Medicine and University Hospital Carl Gustav Carus- Technische Universität Dresden- Dresden- Germany- Helmholtz-Zentrum Dresden-Rossendorf HZDR- Dresden- Germany, Dresden, Germany

**Introduction**: Proton therapy is most suitable for brain tumor entities since there is no dose beyond the tumor tissue. Although patient treatment is already established, there is still a need for preclinical in vivo experiments on proton RBE and regional sensitivities. This research could benefit from an irradiation setup capable of photon and proton irradiation, which is why the small animal irradiation device SmART+ IB was installed and prepared for image-guided experiments.

**Material, Methods, and Results**: The existing setup for mouse brain irradiation (doi: 10.3389/fonc.2022.982417) is integrated in the SmART+ IB, with scattered dose contributions to the flat panel detector and electronics minimised by optimising positioning using Monte Carlo simulations (FLUKA). In preparation for proton and photon treatment planning, various CT imaging parameters of the SmART+ IB were evaluated regarding contrast-to-noise ratio at low imaging doses. For all CT configurations and mouse phantoms tested, reasonable imaging qualities at low doses of a few tens of mGy were obtained. Acceptable range uncertainties of <0.51 mm for stoichiometric and empirical Hounsfield look-up tables were determined. For photon irradiation, dose rates, beam profiles and correction factors for all field configurations were implemented in the treatment planning software (µRayStation 8B). For proton experiments, the corresponding values were measured and the beam model is in preparation. A protocol for reproducible alignment of proton beam and X-ray isocenter was developed.

**Conclusion**: A small animal image-guided setup for proton and photon irradiation is being established to enable comparative animal experiments in the future.

### P 301 - Deep learning for patient-specific calibration of X-ray CT into RSP based on sparse ion radiographies

#### Ines Butz^1^, Marko Zlatic^1^, Héctor Andrade Loarca^2^, Gitta Kutyniok^2^, Katia Parodi^1,3^, Chiara Gianoli^1^

##### ^1^Ludwigs-Maximilians-Universität Munich, Chair of Experimental Physics - Medical Physics, Garching bei München, Germany ^2^Ludwigs-Maximilians-Universität Munich, Bavarian AI Chair for Mathematical Foundations of Artificial Intelligence - Mathematics, München, Germany ^3^German Cancer Consortium DKTK, Partner Site Munich, München, Germany

Treatment planning in particle therapy is typically performed on X-ray Computed Tomography (CT), which undergoes a semi-empirical calibration to ion stopping power relative to water (RSP), resulting in a calibrated CT (*CTcal*) with typical inaccuracies up to 3%. Ion radiography directly measures the integral RSP along the traversed ion path. Based on analytical optimization, the calibration curve can be adjusted by comparing the forward-projection of the *CTcal* with the ion radiographies. However, intrinsic inconsistencies in the forward-projection model can inhibit the elimination of the calibration inaccuracies. To overcome these limitations, a data-driven approach based on a deep convolutional neural network is proposed to infer an improved CT image (*CTinf*) based on a limited number of ion radiographies. The projection to image domain transform is explicitly implemented (Lee at al., IEEE 2022). Back-projected ion radiographies from two projection angles are fed into the network (Ye et al., IEEE 2018) alongside the *CTcal* and compared to the ground truth image in a double-branch architecture, with each branch consisting of 10 convolutional layers (64 channels, 3×3×3 kernel, batch normalization) (see Figure 1). The network is trained/tested on 128/28 slices from clinical-like data generated from CT scans of four head-and-neck patients. First results show a reduction of the RMS error between the *CTinf* and the ground truth, compared to the previously introduced error of the *CTcal*. Refinement of the network architecture and tuning of hyperparameters are ongoing. Authors acknowledge the DFG-funded project ‘HIGH-ART’ and support from Dr. Meyer and Profs. Belka and El Naqa.

### P 302 - Imaging of radioactive and stable oxygen ion beams with a high-resolution DOI in-beam PET scanner

#### Giulio Lovatti^1^, Munetaka Nitta^1^, Mohamad Safari^1^, Daria Boscolo^2^, Ulrich Weber^2^, Emma Haettner^3^, Peter Thirolf^1^, Christoph Scheidenberger^2,4^, Marco Durante^2^, Katia Parodi^1^

##### ^1^Ludwig-Maximilans-Universität, Medical Physics, Munich, Germany ^2^Helmholtzzentrum für Schwerionenforschung, Biophysics, Darmstadt, Germany ^3^Helmholtzzentrum für Schwerionenforschung, Fragment Separator, Darmstadt, Germany ^4^Justus-Liebig University Giessen, Physics, Giessen, Germany

In the framework of the ERC-project Biomedical Applications of Radioactive ion Beams (BARB), an experimental campaign on in-beam positron-emission-tomography (PET) with ^15^O and ^16^O ion beams (Durante, 2020) was conducted at the medical-cave of GSI. This experiment represents the first test of a novel PET-detector design with a reduced number (32) of PET-detector-blocks and 4 Compton-camera arms (Binder, 2022), integrated into a spherical geometry (figure 1). The detector was developed within the LMU project Small Animal Proton Irradiator for Research in Molecular Image-guided Radiation-oncology (SIRMIO). The single PET-detector blocks, developed in collaboration with the National Institutes for Quantum Science and Technology (QST), are composed of 3-layer depth-of-interaction (DOI) scintillators of pixelated (0.9 mm) LYSO (Nitta, 2022). Different phantoms made of varius arrangements of PMMA, PE and air-gaps were used as targets. A ^15^O beam produced and separated with the fragment separator (FRS) in an achromatic mode was implanted in the phantom. For the Spread-out-Bragg-peak (SOBP) a 3D-range-modualator was used (figure 2). The Geant4 and Medium-Energy Gamma-ray Astronomy library (MEGAlib) were used for simulations and the images were reconstructed with a Maximum-Likelihood-Expectation-Maximization iterative algorithm. Comparison of measurements with simulations and ongoing improvements of the PET system will be discussed. This experiment along with the radioactive carbon ion beam campaign (Boscolo, 2021) lays the foundation for the investigation of a novel hybrid-detector for the next phase of the BARB project. We acknowledge the support of ERC Grants8832425 (BARB) and 725539 (SIRMIO), as well as BaCaTeC. We thank the BARB and SIRMIO teams.

### P 303 - High contrast in-room image generation strategy based on non-local total variation denoising for improved image guidance in carbon ion radiotherapy

#### Ho Lee^1^, Min Cheol Han^1^, Chae-Seon Hong^1^, Hojin Kim^1^, Dong Wook Kim^1^, Jin Sung Kim^1^

##### ^1^Yonsei Cancer Center, Radiation Oncology, Seoul, Korea Republic of

The aim of this study is to demonstrate the feasibility of using a non-local total variation (NLTV) denoiser to improve structural delineation in two X-ray in-room images mounted on carbon ion radiotherapy (CIRT) system. The NLTV denoiser is applied to enlarge the difference between striking features and unwanted noise by calculating similarities between non-local patches versus a reference patch. It is directly applied to X-ray in-room images acquired with a projection angle of ±35 degree in CIRT system. The NLTV objective function is minimized based on the steepest gradient descent optimization to augment the difference between a real structure and noise, cleaning noisy pixels without significant loss of the fine structure and details that remain in the X-ray images. The patch of the Gaussian kernel is defined to be 5 × 5 with unit variance and the non-local search area is 21 × 21 with unit variance. The spatially encoded factor is also applied to reduce the weighted averaging effect in high gradient regions to maintain the contrast. It was evaluated using actual measurement data acquired from CIRT system. The noise power spectrum (NPS) peak was on average lower with NLTV denoiser than without denoiser. The peak spatial frequency values of NPS for NLTV denoiser shifted to lower spatial frequencies in comparison to no denoiser. In addition, the NLTV appeared to preserve the whole spatial resolution while reducing noise magnitude. These quantitative results indicate that the NLTV makes more stable and robust for X-ray in-room imaging system.

### P 304 - Experimental characterisation and comparison of two Si-based compact setups for proton imaging of small animals for image-guided proton irradiation

#### Guyue Hu^1^, Angelica Noto^1^, Katrin Schnürle^1^, Matthias Würl^1^, Franz Siegfried Englbrecht^1^, Johannes Gebhard^1^, Jonathan Bortfeldt^1^, Mateusz Sitarz^2^, Per Poulsen^2^, Katia Parodi^1^

##### ^1^LMU Munich, Department of Medical Physics, Garching, Germany ^2^Danish Center for Particle Therapy, Department of Clinical Medicine, Aarhus, Denmark

Within the project SIRMIO, which aims at developing a portable prototype system for precision image-guided small-animal proton irradiation, we are developing advanced image guidance solutions using approaches of different complexity for proton radiographic and tomographic transmission imaging. In this work, we compare two approaches relying on commercial compact planar Si-based pixellated detectors, TimePix3 or Lassena, which provide spatially resolved detection of individual (TimePix3) or integral (Lassena) proton energy deposition. Using a calibration phantom containing tissue-equivalent inserts of well-characterised relative stopping power values, radiographs were obtained at the Danish Center for Particle Therapy in Aarhus, Denmark. The results will compare the achievable spatial resolution and accuracy of water equivalent thickness retrieval in the radiographic mode for the two different detector systems at different imaging doses. Furthermore, ongoing tomographic reconstruction using proton radiographs obtained with the TimePix3 system for the above-mentioned calibration phantom and a dedicated 3D-printed mouse-like phantom will be presented. This work is supported by EU through the grant agreements 725539 (SIRMIO), 730983 (INSPIRE), and 101008548 (HITRIPlus). The authors would like to thank Michael Allum, Tim Edwards, Jonathan Jacobs Headspith, and David Reynolds from Nordson for their support with the Lassena detector. The authors would also like to thank Cristina Oncea and Carlos Granja for their support with the TimePix3 detector.

### P 305 - In-vivo Dose Estimation tool from PET measurements (IDE-PET) for proton range verification

#### Victor Valladolid Onecha^1,2^, Fernando Arias-Valcayo^1,2^, Andrea Espinosa-Rodriguez^1,2^, Paula Ibañez^1,2^, Samuel España^1,2^, Daniel Sanchez-Parcerisa^1,2^, Luis Mario Fraile^1,2^, Jose Manuel Udias^1,2^

##### ^1^Complutense University of Madrid UCM-, Faculty of Physical Science- Nuclear Physics Group and IPARCOS- EMFTEL- CEI Moncloa, Madrid, Spain ^2^Instituto de Investigación del Hospital Clínico San Carlos, IdISSC, Madrid, Spain

Online proton range verifications techniques are necessary to identify deviations and correct them during the treatment delivery. However, the deposited dose must be estimated from the activity distribution in real-time for its clinical implementation. In the case of in-beam PET, this must be done in a few seconds. In this work, we propose a novel tool (IDE-PET) able to obtain dose in real-time from PET measurements. It includes everything necessary to perform this process, both the PET reconstruction and the dose estimation from it. For PET reconstruction, the IDE-PET tool uses a GPU-based MLEM algorithm [1] to reconstruct 3D images in less than a second, enabling on-the-fly monitoring of the PET activity. For dose estimation, we have implemented the Dictionary based software capable of estimating deposited dose from PET activation measurements[2]. By combining these two tools, the IDE-PET is capable of displaying the deposited dose and the proton range deviation online, with only a few seconds of delay. We have tested the IDE-PET over a theoretical scenario where a plastic phantom is irradiated, and the signal measured with an in-house developed in-beam PET. PET activity and deposited dose are displayed with a delay of only 3.5 seconds. Based on these promising results, the same scenario will be reproduced in a clinical proton center.

### P 306 - Influence of morphological changes outside of the primary beam path on carbon-ion radiotherapy monitoring with charged nuclear fragments

#### Rebekka Kirchgässner^1,2,3^, Laurent Kelleter^1,3,4^, Gernot Echner^1,3^, Oliver Jäkel^1,3,4,5^, Jürgen Debus^1,3,4,5,6^, Mária Martišíková^1,3,4^

##### ^1^German Cancer Research Center DKFZ, Division of Medical Physics in Radiation Oncology, Heidelberg, Germany ^2^Karlsruhe Institute of Technology KIT, Department of Physics, Karlsruhe, Germany ^3^National Center for Radiation Research in Oncology NCRO, Heidelberg Institute for Radiation Oncology HIRO, Heidelberg, Germany ^4^NCT, National Center for Tumor Diseases, Heidelberg, Germany ^5^Heidelberg University Hospital, Heidelberg Ion Beam Therapy Center HIT, Heidelberg, Germany ^6^Heidelberg University Hospital, Radiation Oncology and Radiotherapy, Heidelberg, Germany

Carbon-ion radiotherapy is particularly sensitive to beam range uncertainties that can be caused by morphological changes in the patient anatomy. Therefore, the development of treatment monitoring methods is of utmost importance. When interacting with tissue, carbon ions undergo nuclear fragmentation, producing secondary particles which can leave the patient’s body. In this work, charged nuclear fragments are tracked by a silicon pixel tracker with the aim of detecting interfractional changes using the reconstructed fragmentation-vertex distributions. The detection of air cavities in the path of the primary carbon-ion beam in a homogeneous head-sized phantom has been demonstrated before. While morphological changes in the fragment path outside of the beam path have no effect on the delivered dose distribution, they influence the absorption and scattering of the fragments. Therefore, they might decrease the detectability of changes in the primary ion path. This makes the differentiation between changes inside and outside of the treated area crucial for the clinical applicability of this monitoring method. In therapy-like measurements at the Heidelberg Ion Beam Therapy Center, as sketched in the figure below, it was found that the signatures of both types of changes differ significantly. This work demonstrates that the carbon-ion treatment monitoring using charged nuclear fragments has the potential to identify the changes outside of the treated area and thus minimise their influence on the detectability of the therapy-relevant changes of interest. Figure: Measurement setup for investigating the influence of morphological changes outside of the beam path on carbon-ion radiotherapy monitoring with charged nuclear fragments.

### P 307 - PET-based mid-treatment dose escalation of proton therapy in head and neck cancer

#### Guillermo Garrido Hernandez^1^, Helge Henjum^2^, René Winter^1^, Signe Danielsen^3^, Mirjam Alsaker^4^, Kristian Ytre-Hauge^2^, Kathrine Redalen^1^

##### ^1^Norwegian University of Science and Technology, Department of Physis, Trondheim, Norway ^2^University of Bergen, Department of Physics and Technology, Bergen, Norway ^3^St. Olav’s Hospital, Department of Oncology, Trondheim, Norway ^4^St. Olav’s Hospital, Department of Radiotherapy- Cancer Clinic, Trondheim, Norway

**Purpose/Objective**: Image-driven dose escalation to tumor sub-volumes has been proposed to improve treatment outcome in head and neck cancer (HNC). In our study we used 18F-FDG-PET data acquired before treatment (baseline) and two weeks into treatment (interim) to extract boost target volumes (BTV). We then assessed the feasibility of adapting proton therapy plans by BTV-guided dose escalation two weeks into treatment.

**Materials and Methods**: We compared three different methods to segment BTVs from planning CT and 18F-FDG-PET/MR images on nine HNC patients in treatment position; 1) just-enough-interaction semiautomated method, 2) standardized uptake value (SUV) threshold of 2.5, 3) 40% SUVmax threshold. Proton therapy plans with and without BTVs (via method 1) were made in Eclipse (Varian Medical Systems) on the baseline and interim images.

**Results**: Segmentation method 3 showed large variability, while the other methods showed a consistent reduction in BTV volumes from baseline to interim images (Figure 1). Method 1 required fewer manual corrections. Adaptation based on BTV revealed that dose escalation of proton therapy plans to the BTV is feasible without increasing the dose to organs at risk (OARs) (Figure 2).

**Conclusions**: Segmentation methods 1 and 2 showed the expected reduction in BTV on interim FDG-PET scans. Adaptation of dose escalation proton therapy based on interim FDG-PET may give more precise treatment to radioresistant sub-regions of the tumor without significant increase in OAR doses. Further studies in a larger cohort are required to determine the full potential for mid-treatment BTV-guided dose escalation of proton therapy in HNC.

### P 308 - Application of a neural network for prompt gamma-ray based proton range verification: Better than an analytical algorithm?

#### Michael Taylor^1^, Samuel Manger^1^, Samuel Ingram^2^, Ranald MacKay^3^, Karen Kirkby^1^

##### ^1^The University of Manchester, Division of Cancer Sciences, Manchester, United Kingdom ^2^The University of Manchester and Christie NHS Foundation Trust, Division of Cancer Sciences, Manchester, United Kingdom ^3^The Christie NHS Foundation Trust, Medical Physics and Engineering, Manchester, United Kingdom

The use of machine learning (ML) algorithms in healthcare research as seen a dramatic increase in recent years driven, in part, by the quality of both real and synthetically derived data. In particular, particle therapy research has explored the efficacy of using machine learning in areas such as treatment planning and outcome prediction (tumour control and toxicities). In-vivo range verification is arguably one of the greatest challenges facing clinical particle therapy and is an area that could potentially benefit from the application of machine learning. An investigation into the applicability of using a Feedforward neural network to determine proton range using input data from a prompt gamma-ray detection device has been conducted. The network takes digitised gamma-ray detector energy, time and position signals as inputs and aims to determine the position of the maximum gamma-ray intensity position which correlates with the Bragg peak. The speed and accuracy of the neural network has been compared to that of a previously validated analytical algorithm which has been shown, using synthetically generated data, to determine the position of the Bragg peak from a 180 MeV proton beam in water with an uncertainty of around 4%. An overview of the ML model along with its speed and accuracy compared to an analytical algorithm for determining proton range will be presented.

### P 309 - Super Definition CBCT (SD CBCT) reconstruction framework for adaptive particle therapy

#### Justin Chunjoo Park^1^, Xiaoying Liang^1^, Bo Lu^1^, Alessio Parisi^1^, Janet Denbeigh^1^, Sridhar Yaddanpudi^1^, Keith Furutani^1^, Chris Beltran^1^

##### ^1^Mayo Clinic Florida, Radiation Oncology, Jacksonville, USA

**Purpose**: To develop a fully customized, ultra-high definition end-to-end CBCT reconstruction framework that provides superior quality compared to conventional CBCT and enables online adaptive particle therapy.

**Methods**: SD CBCT reconstruction framework comprises of 5 steps: 1) Bow-tie filter correction, 2) novel realtime scatter reduction, 3) conventional FDK reconstruction with maximum resolution (0.5mm × 0.5mm × 0.5mm, 512 × 512 × 300), 4) a novel model based filtered iterative reconstruction that preserves detail while penalizing noise in the uniform HU domain, and 5) HU value conversion based on phantom calibration. To evaluate the performance of our SD CBCT, CatphanTM physical phantom and a clinical head-and-neck patient case were used for analysis. The results were compared with current CBCT generated by commercially available software clinically used in our clinic. (Varian Truebeam^TM^, Palo Alto, CA).

**Results**: Examination of our SD CBCT showed that high-quality, high-resolution CBCT images can be reconstructed. Moreover, in comparison with commercially available software, the image quality of the SDCBCT algorithm is shown to be superior. (See Figure 1). Dosimetric deviation of identical plan of Catphan phantom from CT scanner vs proposed method was within 0.5%. (See Figure 2) With full parallelization of our algorithm with GPU, 512 × 512 × 300 images can be reconstructed within 3 minutes.

**Conclusion**: This work demonstrates the potential for providing high-quality CBCT for online adaptive particle therapy. More rigorous investigation and analysis are currently being performed with proton TPS as well as CBCTs from various proton therapy systems.

### P 310 - Dosimetric evaluation of MRI-derived pseudo-CT for MR-only proton therapy planning in prostate cancer

#### Kajsa Fridstrøm^1^, Sophie Nord^1^, Sigrun Saur Almberg^2^, Signe Danielsen^2^, Kathrine Redalen^1^

##### ^1^Norwegian University of Science and Technology, Department of Physics, Trondheim, Norway ^2^St. Olavs hospital, Cancer Clinic, Trondheim, Norway

**Background**: For prostate cancer, guidelines suggest the use of MRI to delineate target volumes due to superior soft tissue contrast compared to CT. Although CT is still the primary imaging modality for both photon and proton dose calculations, studies have shown that MRI-based radiotherapy planning (MR-only) using pseudo-CTs generated from MR is feasible. Our aim was to evaluate proton dose calculation accuracy on MR-based pseudo-CT for prostate cancer and compare the results to photons.

**Methods**: Both MRI and CT were acquired in treatment position from 8 prostate cancer patients treated with photons. Pseudo-CTs were generated using the MRI planner software (Spectronics, Sweden). After co-registration, dosimetric accuracy was evaluated by generation of photon and proton plans on the original planning CT, and recalculating the plans on the pseudo-CTs using RayStation (RaySearch). All plans were made with a total dose of 60 Gy (20 fractions of 3 Gy).

**Results**: The mean dose difference between plans made with pseudo-CT and original planning CT for target volumes and OARs for photons and protons are shown in Figure 1. The mean dose difference to both target volumes and OARs were well below 1 Gy, although the differences where slightly larger for protons. The external contour showed the largest dose difference, arising as a consequence of the co-registration. The largest mean dose difference in the target volumes was 0.65 +/− 0.12 Gy for photons and 0.37 +/− 0.81 Gy for protons.

**Conclusion**: Dosimetric analysis of prostate cancer patients revealed that MR-only proton planning is feasible using pseudo-CT.

### P 311 - Patient-specific 3D CT images reconstruction from 2D kV images via vision transformer-based deep-learning

#### Yuzhen Ding^1^, Jason Holmes^1^, Baoxin Li^2^, Carlos Vargas^1^, Sujay Vora^1^, William Wong^1^, Mirek Fatyga^1^, Robert Foote^3^, Samir Patel^1^, Wei Liu^1^, Martin Bues^1^, Daniel Robertson^1^

##### ^1^Mayo Clinic, Radiation Oncology, Phoenix, USA ^2^Arizona State University, School of Computing and Augmented Intelligence, Tempe, USA ^3^Mayo Clinic, Radiation Oncology, Rochester, USA

**Purpose**: In proton treatment, patient alignment often relies on 2D orthogonal kV images as no 3D on-the-bed-imaging capability is available in some proton machines. But the visibility of the tumor in kV images is limited, thus leading to large patient setup uncertainties. Therefore, it is necessary to reconstruct the 3D CT images given kV images.

**Methods**: An asymmetric autoencoder-like network built with vision-transformer blocks was developed. The data was collected from 1 head and neck patient: 2 orthogonal kV images (10241024 voxels), 1 3D CT (512512512) acquired from the in-room CT-on-rails before kVs were taken and 2 digitally-reconstructed-radiograph (DRR) images (512512) based on the CT. We resampled kV images every 8 voxels and DRR and CT every 4 voxels, thus formed a dataset consisting of 262,144 samples, in which the images have a dimension of 128 for each direction. kV and DRR images were used for training, the CT was used as ground-truth (gCT) and mean-absolute-error (MAE) was used as the loss function. Independent kV images were used in testing. The image quality of the synthetic CT (sCT) was evaluated using MAE and per-voxel-absolute-CT-number-difference volume histogram (CDVH).

**Results**: The model achieved a MAE of <40HU and the CDVH showed that <5% of the voxels had a per-voxel-absolute-CT-number-difference larger than 185HU (Figure.1). Figure.2 compared a typical CT slice between the *gCT* and *sCT*, which agreed with each other well.

**Conclusion**: A patient-specific vision-transformer-based network was developed and shown to be accurate and efficient to reconstruct 3D CT images from kV images.

### P 312 - Focus stacking: A new way for improved spatial resolution and 3D feature detection with particle radiography

#### Lennart Volz^1^, Marco Durante^1^, Christian Graeff^1^, Charles-Antoine Collins-Fekete^2^

##### ^1^GSI Helmholtz Center for Heavy Ion Research GmbH, Biophysics, Darmstadt, Germany ^2^University College London, Medical Physics and Biomedical Engineering, London, United Kingdom

We demonstrate a novel focus stacking technique to improve spatial resolution of single-event particle radiography (pRad), and explore its potential for 3D feature detection. Focus stacking, common in optical photography and microscopy, is a technique to combine multiple images with different focal depths into a super-resolution image. Each pixel in the final image is chosen from the image with the largest gradient at that pixel’s location. pRad data can be reconstructed at different depths in the patient utilizing an estimate of each particle’s trajectory (distance-driven binning; DDB). For a given feature, the resolution is maximal in the image reconstructed at the approximately same depth. This enables to apply focus stacking on a series of DDB images from a single pRad acquisition (Figure 1). This also provides information on the features’ depths. We tested the method with Geant4 simulated pRads of a water phantom (20cm thick) with bone cube inserts (1x1x1ccm) and an XCAT digital lung patient. For proton radiography of the cube phantom (Figure 2a/b), focus stacking achieved a 50% median resolution improvement compared to a state-of-the-art reconstruction. For the XCAT, resolution visually improved (Figure 2c/d) without loss in accuracy. For helium radiography, the central cube could be located with an accuracy comparable to the cube’s edge length. Focus stacking utilizes the inherent 3D information encoded in pRad through the particle’s scattering, overcoming current spatial resolution limits and enabling 3D feature localization. pRads can be acquired within few seconds with available systems and no modifications are required, making focus stacking a powerful technique.

### P 313 - Contrast agents for in-vivo range verification in proton therapy

#### Luis Fraile^1,2^, Samuel España^1,2^, Andrea Espinosa-Rodríguez^1,2^, Víctor Valladolid Onecha^1,2^, Daniel Sánchez Parcerisa^1,2^, Miguel García Díez^1,2^, Paula Ibáñez^1,2^, Víctor Martínez-Nouvilas^1,2^, Víctor Sánchez-Tembleque^1,2^, José Manuel Udías^1,2^, Carolina Gutiérrez-Neira^3^, Sílvia Viñals^3^, Miguel Ángel Morcillo^4^, Marta Ibáñez-Moragues^4^, María José García Borge^5^, Enrique Nácher^6^, Paloma Bragado^2,7^, Álvaro Gutiérrez-Uzquiza^2,7^, Almudena Porras^2,7^, Alejandro Mazal^8^, Juan Antonio Vera-Sánchez^8^

##### ^1^Universidad Complutense de Madrid, Grupo de Física Nuclear & IPARCOS, Madrid, Spain ^2^Instituto de Investigación del Hospital Clínico San Carlos, IdISSC, Madrid, Spain ^3^Universidad Autónoma de Madrid, Centro de Microanálisis de Materiales CMAM, Madrid, Spain ^4^CIEMAT, Unidad de aplicaciones médicas de las radiaciones ionizantes, Madrid, Spain ^5^CSIC, Instituto de Estructura de la Materia, Madrid, Spain ^6^CSIC/Universidad de Valencia, Instituto de Física Corpuscular, Madrid, Spain ^7^Universidad Complutense de Madrid, Departamento de Bioquímica y Biología Molecular, Madrid, Spain ^8^Quironsalud, Centro de Protonterapia, Madrid, Spain

The use of contrast agents is common place in medical imaging to enhance specificity and image quality. In the context of online range verification in proton therapy, we have proposed employing activable contrast media to improve range verification using PET and prompt-gamma (PG) imaging. Contrary to radiotracers produced by proton reactions on naturally-occurring isotopes [Zhu2011, PMB56], other external isotopes with a high proton-induced reaction cross-section and a low production threshold can be administered. They have the advantage of being able to yield significant radioactivity in a volume close to the proton Bragg peak, which can be subsequently identified by gamma detection or imaging techniques. In the framework of the PRONTO-CM project we have explored several candidates for contrast media from the point of view of the reaction mechanisms and production yields, biocompatibility, and the prospects for their detection as PET and PG products in protontherapy. We will describe the possibilities offered by iodine, a contrast agent already approved for a number of medical applications, which leads to the production of the ^127m^Xe, suitable for online range verification [Espinosa21, RPC185]. We will also address the use of ^18^O-enriched water (18-W). Experiments on a chicken-embryo chorioallantoic membrane tumor model of head and neck cancer reveals the production and retention of ^18^F within the last millimeter of the proton range inside the tumor, which makes the measurement of the proton range possible using offline PET imaging [España2022, SciRep12]. The method also enables the localization of the activation via the detection of prompt gammas.

### P 314 - An optimized dual-energy CT simulation protocol for planning and IGRT for proton therapy of ocular melanoma

#### Tomi Nano^1^, Jessica Scholey^1^, Sinha Sumi^1^, Kavita Mishra^1^

##### ^1^University of California- San Francisco, Radiation Oncology, San Francisco, USA

**Introduction**: Proton therapy of ocular lesions conventionally uses 2D imaging simulation for treatment planning and image-guidance (IGRT), however, 3D CT simulation is more common in radiotherapy. The purpose of this study is to develop a CT simulation protocol that will enable treatment planning and IGRT for proton ocular treatment with current clinical systems (EyePlan).

**Methods**: An anthropomorphic head phantom containing tantalum clips was used to optimize a dual-energy CT (DECT) protocol with metal artifact reduction. Conventional 2D x-ray images were compared with digitally reconstructed radiographs (DRRs) and clip positions were measured. EyePlan comparison plans were created using clip measurements from x-rays and DRRs. We compared organ-at-risk (OAR) V50%/V90% of the macular, optic disc, ciliary body, and optic nerve while maintaining PTV V100%-to-98%.

**Results**: An optimized DECT protocol acquired with a Siemens Syngo Monoenergetic 170keV with 1mm slices shows reduced streaking artifacts and high contrast around clips compared to an equivalent 120kV CT protocol (Figure 1A). Although DRRs have lower resolution than x-ray images (Figure 1B), the clip center-of-mass can be easily identified in both x-rays and DRRs. The clip measurement difference between x-ray and DRR images was 0.57±0.36mm (Figure 2) and the average OAR dose difference was 1.9±0.8% respectively.

**Conclusions**: An optimized DECT simulation protocol provides DRRs capable of accurately measuring clip position that is clinically acceptable for ocular proton treatments. In addition to providing DRRs for the current EyePlan clinical workflow, 3D imaging with DECT is translatable to 3D planning with future systems such as RayStation’s ocular proton planning.

### P 315 - Integrated mode proton radiography using 2D lateral projections for adaptive radiotherapy

#### Mikael Simard^1^, Daniel Robertson^2^, Ryan Fullarton^1^, Sam Beddar^3^, Charles-Antoine Collins-Fekete^1^

##### ^1^University College London, Medical Physics & Biomedical Engineering, London, United Kingdom ^2^Mayo Clinic Arizona, Division of Medical Physics- Department of Radiation Oncology, Phoenix, USA ^3^The University of Texas MD Anderson Cancer Center, Department of Radiation Physics, Houston, USA

The acquisition of proton radiographs (pRads) using the proton source is an imaging solution of interest for adaptive proton therapy, as it is co-planar to the treatment beam and provides highly accurate water equivalent thickness (WET) maps. A clinically compatible option is integrated mode proton imaging, which acquires integrated signals from individual pencil beams. This study reports the image quality of a fast, low-cost pRad device combining a volumetric scintillator and CCD cameras. pRads were acquired at Mayo Clinic capturing 2x2D lateral views of the 3D energy deposition inside the scintillator (Fig 1a). WET maps were reconstructed using a novel reconstruction framework performing a weighted reprojection of multiple candidate Bragg peaks. Data was acquired in a 13x13 cm^2^ field of view using clinical settings (135 MeV, 3 mm beam spacing). The Las Vegas phantom (contrast), slanted edge (resolution), tissue-substitute inserts (WET accuracy), and a paediatric head phantom (189 MeV) were scanned. The proposed reconstruction is compared with a conventional integrated mode system using a range telescope (1D lateral). Clinical image quality for the paediatric phantom is reported in fig-1d. Compared to 1D lateral, our method yields a 30% increase in resolution (fig-1b) and a 27% increase in contrast (Fig-1c). High WET accuracy (mean absolute error of 0.4 mm, fig-1e) is obtained. Sub-second imaging is also achievable with a 6 mm beam sampling. This work illustrates that pRads can be obtained with clinical beam settings. Applications are expected for patient positioning, identifying the time of offline replanning, and motion management.

### P 316 - Dosimetry comparison between voluntary breath hold and free breathing in proton pencil beam scanning treatment

#### Chien-Cheng Sung^1^, Meng-wei Ho^2^, Chen-shou Chui^1^, Pei-jiuan Juang^1^, Pei-Yi Lee^1^, Yu-Ming Wang^1^

##### ^1^Chang Gung Memorial Hospital, Proton and Radiation Therapy Center, Kaohsiung, Taiwan Province of China ^2^Chang Gung Memorial Hospital, Proton and Radiation Therapy Center, Linco, Taiwan Province of China

**Background or Introduction**: Two different breathing techniques (voluntary breath hold and free breathing) were investigated for their differences in proton pencil beam delivery results. For both techniques,the ANZAI respiratory gating system was used to trigger the proton beam according to the breathing cycle and the gating window. We wish to assess and simulate the delivered dose distributions of these breathing modes in proton radiation therapy.

**Material and Methods**: Ten pencil beam scanning patterns were used to irradiate a PTW Octavius detector 1500-XDR which was mounted on a CIRS motion platform. The platform was programmed to simulate both voluntary breath hold and free breathing motions. The platform motion was monitored by the ANZAI respiratory gating system which then triggered the proton beam. The dose distributions measured by the PTW Octavius detector for both breathing motions were compared with the gamma index, using a 3%/3mm criteria. We also simulated the doses interplay effect in monte carlo simulation.

**Results and Discussion**: The SI-moving platform can be used in CIRS Phantom to measure the dose distributions. Using voluntary breath hold(VB) mode for respiratory-gated technique in proton radiotherapy can reduce the treatment time and increase the proton beam efficiency. According to the gamma index, voluntary breath hold(VB) mode can reduce the interplay effect in pencil beam scanning beam.

### P 317 - Plan adaptation in a particle therapy: Retrospective analysis on one-year treatments at the Italian National Center for Oncological Hadrontherapy

#### Alessandro Vai^1^, Elena Livia Chilug^2^, Silvia Molinelli^1^, Amelia Barcellini^3^, Giuseppe Magro^1^, Alfredo Mirandola^1^, Eleonora Rossi^1^, Stefania Russo^1^, Ester Orlandi^3^, Mario Ciocca^1^

##### ^1^CNAO Foundation, Medical Physics, Pavia, Italy ^2^Horia Hulubei National Institute, Physics and Nuclear Engineering IFIN-HH, Magurele-Ilfov, Romania ^3^CNAO Foundation, Radiotherapy Department, Pavia, Italy

**Purpose/Objective**: To analyze retrospectively our plan adaptation strategy based on re-evaluative imaging and robust optimization.

**Material and Methods**: A single-center analysis was carried out on CT-based PT treatments (protons and carbon ions) delivered from July21 to June22. Plans were optimized with Raystation TPS (v.8b) with site-specific uncertainties parameters (patient position: 2-8 mm, range: 2%-5%). At least 1 re-evaluative image dataset was acquired during treatment course, whose inspection drives the choice for plan adaptation. Possible correlations between more than 20 parameters (patient data, tumor histology and location, treatment schedule timeline, particle, nominal plan parameters (e.g. beam geometry, robustness) and rate of replanning were investigated.

**Results**: Four hundred eighty-five (485) pts received a CT-based treatment plan and n=340 (70.1%) had at least 1 re-evaluative CT. N=87(17.9%) were re-optimized once and n= 20 (5.9%) 2 times or more. We registered the following rate of replanning respectively for brain, H&N, abdomen, gyneco/pelvis tumor sites: 2.7%/55.7%/27.2%/13.8%. Plans were re-optimized in minimal part for clinical reason and setup variations (7.8%) while mostly for target coverage variations (78.4%). Relative target D95 and D1 median (IR) difference to nominal values were respectively -4.1%(6.3%) and 2.4%(3.1%). For OARs, largest variations were observed for near-maximum dose for pelvis (rectum median ΔD1=9.8Gy(RBE)) and mean dose for H&N (larynx median ΔDmean =16.2Gy(RBE)). Tumor location was the only parameter significantly related to the rate of replanning (p<0.05).

**Conclusion**: A retrospective analysis of 1 year of treatment plans showed that our approach of robust planning combined with site-specific re-evaluative CT is able to detect sub-critical dose variations for both target coverage and OARs.

### P 318 - Motion management with respiration guidance gating system (RG2S) verified firstly on the heavy-ion therapy facility in Lanzhou

#### Pengbo He^1^, Qiang Li^1^, Guangru Li^1^, Xinyang Zhang^1^, Jun Zhang^1^, Jian Wang^1^

##### ^1^Institute of Modern Physics- Chinese Academy of Sciences, Department of Medical Physics, Lanzhou, China

In order to treat the moving targets under the synchrotron-based pulsed heavy-ion beam delivery, a novel respiration guidance gating system (RG^2^S) was developed to synchronize the patterns between the patients’ respiration and synchrotron magnetic excitation curve (MEC). The respiratory synchronized irradiation was realized by adding a time interval between the adjacent MECs, which was calculated based on the even sequence of synchrotron and the period of a patient specific breathing guidance curve, as shown in Figure 1(a). A short breath-hold time was added to the end exhalation phase that was coincident with the beam extraction flattop. In this way, each beam pulse can be fully utilized while the target is in a relative static state during irradiation. The functionality and effectiveness of the RG^2^S system were verified firstly on the Heavy Ion Medicine Machine (HIMM) in Lanzhou, China. A programmable portable moving platform was fixed in the treatment room (Figure 1(c)) that could move following the volunteers’ breath, where the volunteers conducted the breathing guidance tests outside the treatment room as shown in Figure 1(b). A treatment planning for a cubic target (6 cm × 6 cm × 6 cm, 2Gy) was designed using raster scanning beam delivery mode with RayStation (RaySearch Laboratories AB, Stockholm, Sweden). As shown in Figure 1(d-g), the dose distribution uniformity was increased by a factor of 2.7 compared to the conventional gating method, while the treatment efficiency was increased 2.3 times. Thus, the RG^2^S system greatly improved the performance of the heavy-ion treatment facility.

### P 319 - Prompt Gamma Time Imaging technique for proton range control during particle therapy

#### Adélie André^1^, Saba Ansari^1^, Yannick Boursier^2^, Joël Hérault^3^, Christophe Hoarau^1^, Marie-Laure Gallin-Martel^1^, Daniel Maneval^3^, Christian Morel^2^, Giovanni Tripodo^4^, Sara Marcatili^1^

##### ^1^Université Grenoble Alpes, CNRS- Grenoble INP- LPSC-IN2P3 UMR 5821, Grenoble, France ^2^Aix-Marseille Univ, CNRS/in2p3- CPPM, Marseille, France ^3^Centre, Antoine Lacassagne, Nice, France ^4^Dipartimento di Fisica e Chimica “E. Segrè”, Università degli Studi di Palermo- via delle Scienze, Palermo, Italy

We propose a new monitoring approach for Particle Therapy called Prompt Gamma Time Imaging (PGTI). The purpose of this approach is to measure the proton range in-vivo with the aim to adapt the treatment during the irradiation in case of unforeseen anatomical changes or mispositioning. The technique relies on the measurement of particle Time Of Flight (TOF) followed by the reconstruction of the PG vertex distribution in the space domain. This original reconstruction allows combining the response of several detectors at different angular positions, providing a 3D PG distribution and a uniform detection sensitivity in the whole field of view. The use of multiple detectors with no collimation also ensure the high detection efficiency required for real-time monitoring. A dedicated PG detector is currently under development to measure the proton plus PG TOF. It is composed of a diamond-based beam monitor read in time coincidence with a multi-channel gamma detector (TIARA) composed of 30 Cherenkov-based detection modules (Figure 1). The system spatial resolution correlates to the system Coincidence Time Resolution (CTR) which is of 109 ps σ for our current prototype. Our previous simulation work (Jacquet et al. PMB 2020) demonstrated that a 1 mm sensitivity (at 2σ) is achievable with a CTR of 100 ps σ and 3000 PG acquired (∼10^8^ incident protons). More recently, using a heteogeneous phantom, we measured a range shift of 4 mm at 2σ with only 600 PGs. We will present the PGTI concept and the detector performances obtained at clinical proton facilities.

### P 320 - Proton therapy for lung cancer patients: Three years clinical experience

#### Vicki Taasti^1^, Esther Kneepkens^1^, Djoya Hattu^1^, Marije Velders^1^, Jolein Mannens^1^, Judith van der Stoep^1^, Judith van Loon^1^, Dirk De Ruysscher^1^, Wouter van Elmpt^1^, Ilaria Rinaldi^1^

##### ^1^Department of Radiation Oncology MAASTRO, GROW – School for Oncology- Maastricht University Medical Centre+, Maastricht, Netherlands

**Purpose**: To report on three years of proton therapy for lung cancer patients, selected based on normal-tissue-complication-probability (NTCP) plan comparison following the Dutch model-based approach in our proton facility with a Mevion Hyperscan S250i system.

**Methods**: For patients with tumor movement <5 mm a clinical target volume (CTV) approach was used, while for tumor movement >5 mm (up to 20 mm) an internal target volume (ITV) approach was applied (Figure 1). All plans were robustly optimized (5 mm setup, 3% range uncertainty) in RayStation based on a 4DCT. Patients were treated in free-breathing. Here we describe our clinical workflow experience.

**Results**: In total, we have treated 243 lung cancer patients, where 70 patients had a tumor movement >5 mm. Fractionation schedules included 20-30 fractions (mainly 25×2.4 Gy/fx or 30×2 Gy/fx). The mean doses to heart, lung, and esophagus varied greatly, depending on tumor position (Figure 2), but compared to the photon plans the mean dose was on average 2.5 Gy lower for heart and 0.9 Gy lower for lungs. All plans had a clinical acceptable target coverage (V95%>95% in the robust voxel-wise minimum dose distribution). Plan adaptations were performed for 26% of patients, mainly triggered by target under-dosage, often caused by tumor regression. Several projects have been performed to increase efficiency, reducing treatment planning and delivery times. The median delivery time was reduced from 30 to 23 minutes.

**Conclusion**: The current workflow enables treating a large number of lung cancer patients, even those with large tumor movements.

### P 321 - Proton and photon breath-hold in radical radiotherapy for lung cancer: Exploration of its benefits

#### Esther Kneepkens^1^, Judith van der Stoep^1^, Daisy Emans^1^, Marije Velders^1^, Anouk Vullings^1^, Janou Buck^1^, Vicki Trier Taasti^1^, Djoya Hattu^1^, Dirk de Ruysscher^1^, Stephanie Peeters^1^

##### ^1^GROW-School for Oncology and Developmental Biology- Maastricht University Medical Centre, Department of Radiotherapy MAASTRO Proton Therapy, Maastricht, Netherlands

**Objective**: Inspirational breath-hold (IBH) during radiotherapy for lung cancers may be beneficial to reduce dose to normal tissues because of larger lung volumes or margin reduction compared to free breathing (FB). In this study, we compared proton and photon FB and IBH plans.

**Material and Methods**: For nineteen non-small-cell lung cancer patients, four treatment plans (30 × 2 Gy), were compared: photon and proton plans in FB and IBH.For the photon plans, CTV-PTV margins were 5 mm for the nodes (CTVn), and an amplitude-dependent margin for the primary tumor (CTVp). For the proton plans, if the amplitude was <5 mm, a 3 mm margin around CTVp, and 0 mm around CTVn were used. Otherwise, an internal target volume (ITV) + 2 mm margin was used. Robust optimization settings were 5 mm setup and 3% range uncertainty. Photon plans were made using 2 half-arcs, and proton plans had 2-3 beam directions. Organ-at-risk (OAR) dose differences and normal tissue complication probabilities (NTCP) were evaluated.

**Results**: Dose and volume differences are reported in Table 1/Figure 2A/2B; NTCPs in Figure 2C/2D. No differences were seen in OAR dose sparing or NTCP decrease in the IBH plans for patients with >5mm versus <5 mm amplitude (Figure 2).

**Conclusion**: IBH did not generally improve relevant NTCPs for either proton or photon plans. However, for number of patients, a clinically relevant decrease in grade 2 radiation pneumonitis NTCP was observed. Patient or plan specific parameters predicting if a patient would benefit overall from IBH, require more investigation.

### P 322 - AI-assisted proton radiography interpretation for fast detection and classification of treatment deviations

#### Giuliano Perotti Bernardini^1^, Gabriel Guterres Marmitt^1^, Carmen Seller Oria^1^, Arthur Galapon^1^, Peter van Ooijen^1^, Johannes Langendijk^1^, Stefan Both^1^

##### ^1^University Medical Center Groningen, Department of Radiation Oncology, Groningen, Netherlands

**Purpose**: The effectiveness of dose delivery in proton therapy can be compromised by various sources of range uncertainty. Proton radiography (PR) provides a method to detect these treatment deviations. We propose a deep learning tool to assist with evaluation of PRs in the fast pace clinical environment.

**Materials and Methods**: Thirty-two (32) HN cancer patients were employed and subjected to artificial changes scripted in RayStation for patient setup errors (SE), ±2 to ±4 mm, CT calibration curve errors (CC) such as fat tissue ±3 to ±5%, soft tissue ±3 to ±5%, and bone ±7 to ±11%, and anatomical changes (AC) ± 2 to ± 12 mm mimicking weight changes. PRs were simulated using ray-tracing method for a 26 × 26 cm^2^ field, 210 MeV beam energy and 270° gantry angle. 14503/13653 range shift maps (RSM) images between reference and impaired PRs were calculated (Figure 1) with and respectively without background errors (SE =1mm, CC ≤ 2%). Based on these images (10114 training, 1378 validation, 3011 evaluation) a Convolutional Neural Network (CNN) model was generated. EfficientNet-B1 was selected for multi-label classification of SE, CC, AC errors usually encountered in clinical practice. Accuracy, Precision, Recall, and F1-score performance metrics were evaluated.

**Results**: Figure 2 shows the CNN training results and the metrics with and without background errors, respectively: Accuracy: 97%/82%, Precision: 99%/93%, Recall:99%/95%, F1-score: 99%/93%. It took 6 s to interpret a PR.

**Conclusion**: The AI-based tool can identify SE, CC and AE sources of range errors and assist with PR interpretations in the clinic.

### P 323 - First application of the Prompt Gamma-Ray Timing method for proton treatment verification on an anthropomorphic head phantom under clinical irradiation

#### Krystsina Makarevich^1,2^, Katja E. Römer^3^, Sonja M. Schellhammer^1,2^, Joseph A. B. Turko^3^, Andreas Wagner^3^, Toni Kögler^1,2^

##### ^1^Helmholtz-Zentrum Dresden-Rossendorf, Institute of Radiooncology – OncoRay, Dresden, Germany ^2^Technische Universität Dresden and Helmholtz-Zentrum Dresden-Rossendorf, OncoRay – National Center for Radiation Research in Oncology/Faculty of Medicine and University Hospital Carl Gustav Carus, Dresden, Germany ^3^Helmholtz-Zentrum Dresden-Rossendorf, Institute of Radiation Physics, Dresden, Germany

The Prompt Gamma-Ray (PG) Timing technique (PGT) is a promising candidate for online proton treatment verification as it is small, light-weight, and gantry-integrable, and it introduces no additional dose to patients. We report on the first evaluation of the PGT system under clinically relevant conditions. To this end, the CIRS Proton Therapy Dosimetry Head phantom was irradiated with clinically realistic glioblastoma treatment plans. Time-of-flight distributions of PG were acquired with inorganic scintillators of different sizes (Ø1″×1″, Ø2″×1″, Ø2″×2″). Fast dedicated plug-on spectrometers with throughput rates up to 1 × 10^6^ s^−1^ and integrated pile-up rejection were used to enable high-resolution time and energy spectroscopy. While maximizing the detection efficiency, the Ø2″-detectors show the expected reduced time resolution and worse gain stability compared to the Ø1″×1″ crystals. Doubling the acceptance of stabilized voltage dividers from 4 to 8 TeV/s improves the gain stability and significantly reduces the gain drift caused by extreme load changes during pencil beam scanning. With active pile-up rejection, the overall count rate was reduced from 700 × 10^3^ s^−1^ to a maximum of 450 × 10^3^ s^−1^. The limited number of processable PG with less than 100 events per treatment spot per detector was identified as the main factor inhibiting clinical applications. These findings imply that further development of the PGT setup is necessary for a successful translation into clinical applications. We propose strategies for such a development, including the use of larger crystals, or higher segmentation, small ring collimators, and spot aggregation, and report on the first results acquired by applying these methods.

### P 324 - Anatomy-preserving virtual CT generation for proton online adaptive therapy

#### Suryakant Kaushik^1,2^, Albin Fredriksson^1^, Iuliana Toma-Dasu^2,3^, Jakob Ödén^1^

##### ^1^RaySearch Laboratories, Research and Development, Stockholm, Sweden ^2^Stockholm University, Medical Radiation Physics- Department of Physics, Stockholm, Sweden ^3^Karolinska Institutet, Medical Radiation Physics, Stockholm, Sweden

**Purpose**: CT images generated from CBTCs are used for replanning daily in proton adaptive workflows. Corrected CBCTs (cCBCTs) better preserve the anatomy in comparison to virtual CTs (vCTs) but might be affected by artifacts and noise. This study proposes a method to generate an anatomy-preserving virtual CT (APvCT) for comparison with conventional cCBCT and vCT methods in the context of online adaptive proton radiotherapy.

**Material and Methods**: RayStation v12A was used for four low-risk prostate cancer patients to generate various daily CTs using three methods: (1) cCBCT: Analytical CBCT correction and conversion using planning CT (pCT) as a reference and CBCT-CT joint histogram. (2) vCT: pCT deformably registered to CBCT without controlling region of interests (cROIs). (3) APvCT: Bladder, rectum and femoral heads as cROIs were generated on cCBCT using deep-learning segmentation with manual corrections and rigidly registered to CBCT. The CBCT and the pCT were then registered utilizing anatomically constrained deformations driven by boundary conditions on cROIs. Further, a robustly optimized proton plan was created on APvCT and subsequently re-calculated on cCBCT and vCT for dosimetric analysis.

**Results**: Figure 1 displays one example of the cROIs overlayed on the daily CTs showing that APvCT improved the anatomy preservation compared to vCT, and the HU, density and stopping power ratio distributions compared to cCBCT. The detailed comparison of various parameters for different ROIs and isodoses is shown in Figure 2.

**Conclusion**: An APvCT can be accurately and efficiently generated for use in proton online adaptive therapy based on automatic contouring on cCBCT.

### P 325 - Innovative positioning system for gantryless treatment room in proton therapy

#### Faiza Bourhaleb^1^, Roberto Orecchia^2^, Raffaele Andrea Prisco^2^, Massimiliano Tabasso^2^, Claudia Pardi^1^, Vincenzo Forlese^2^, Grazia Krizia Masciavè^2^

##### ^1^I-SEE srl, Particle Therapy, Torino, Italy ^2^Linearbeam srl, Particle Accelerator, Ruvo di Puglia, Italy

**Introduction**: Proton therapy has provided much evidence of quality and accuracy in treatment planning. Proton beam scanning technology allows physicians to precisely manipulate and direct the beam to conform the dose to the tumour. The current main problem with proton therapy is the underlying technology and the enormous costs compared to conventional radiotherapy. To take full advantage of this relatively new treatment technique, we have defined a new configuration that can reduce costs. In fact, we propose a comprehensive study on the use of a fixed source instead of a gantry.

**Methods**: Two robotic arms are used as positioning system. Each arm has six degrees of freedom. The first one is handling the couch while the second one is endowed with an imaging system that uses a Cone Beam CT installed on a C-arc ring. The positioning system is controlled and interfaced with a dedicated software translating and converting complex motions to equivalent once in conventional treatments.

**Results**: Full Monte Carlo investigations have been performed for all configurations. We see then advantages of combining such solutions in offering new smart way of fully exploiting the hardware in our disposal nowadays. This solution is well integrated within the whole treatment workflow

**Conclusions**: This work has explored the feasibility and the advantages in the use of a gantryless configuration combined with a robotic positioning system focusing on outcomes in treatment planning. According to the results obtained, costs and benefits are reported together with additional non-standard field setups that propose planning improvements

### P 326 - 4D robust evaluation of proton PBS treatments for respiration management

#### J. Pablo Cabello Garcia^1^, J. Diego Azcona Armendáriz^1^, P. Borja Aguilar Redondo^1^, Alberto Viñals Muñoz^1^, Roser Fayos-Solà Capilla^1^, Miguel García Cutillas^1^, Carlos Ramón García^1^, Diego Pedrero de Aristizábal^1^, Elena Antolín San Martín^1^, J. Miguel Delgado Rodriguez^1^

##### ^1^Clinica Universidad de Navarra, Radiofísica y Protección Radiológica, Madrid, Spain

**Purpose**: To describe the workflow to optimize and evaluate treatments with organ motion with pencil beam scanning (PBS) proton therapy.

**Materials and Method**: The GTV was contoured in every phase of a 4DCT and the ITV was created combining all of them. The CTV was created adding a patient-dependent clinical margin. The average intensity projection was used for optimization. Back incidences were used, minimizing the effect of breathing. Dosimetries were robustly optimized with (5mm, 5%) (spatial, range) uncertainty. Plans were calculated in all phases of the 4DCT. All the phase CTs were deformably registered to the average projection. The registrations were used to accumulate the dose and, then, compare the metrics (D95%) between planned and accumulated doses. A simple approach was used to combine range and spatial uncertainties with the respiration effect in the accumulated distribution. For the spatial robustness, the CTV was expanded 4mm (quadratic sum of DIR and setup uncertainties). To combine range and respiration; a CTVproximal and CTVdistal were defined by displacing the CTV distally and proximally along the beams’ direction. Metrics were evaluated in these volumes. Interplay can be mitigated with repainting. A CBCT was performed in every session. Five patients were treated: 2 lungs, 2 pancreas and 1 liver.

**Results**: The optimizations were performed until good coverage and OAR metrics in the whole analysis were achieved. The tables shows coverage indicators in the five cases.

**Conclusions**: The results shows that PBS treatments with dynamic organs involved can be delivered in a safe way.

### P 327 - Inter- and intrafractional 4D dose accumulation for evaluating ΔNTCP robustness in lung cancer

#### Andreas Smolders^1,2^, Adriaan Hengeveld^3^, Stefan Both^3^, Robin Wijsman^3^, Johannes Langendijk^3^, Damien Charles Weber^1,4,5^, Tony Lomax^1,2^, Francesca Albertini^1^, Gabriel Guterres Marmitt^3^

##### ^1^Paul Scherrer Institute, Center for Proton Therapy, Villigen, Switzerland ^2^ETH Zurich, Department of Physics, Zurich, Switzerland ^3^University Medical Center Groningen, Department of Radiation Oncology, Groningen, Netherlands ^4^Inselspital- Bern University Hospital, Department of Radiation Oncology, Bern, Switzerland ^5^University Hospital Zurich, Department of Radiation Oncology, Zurich, Switzerland

**Background and purpose**: The Dutch model-based approach selects patients for proton therapy only if a predefined reduction in normal tissue complication probability (NTCP) with respect to photon therapy is achieved. This study investigates if this decision, necessarily made based on the treatment plan, holds through the course of treatment for lung cancer patients, as the NTCP is affected by delivery inaccuracies caused by breathing motion such as tissue density changes and the interplay effect.

**Materials and Methods**: For 20 lung cancer patients, the delivered proton therapy dose was retrospectively reconstructed on weekly 4DCTs using log-file-based dose reconstruction. These phase-resolved doses were accumulated inter- and intrafractionally using deformable image registration (DIR). The biologically equivalent dose was used to account for dose variations between fractions. Since this depends on the uncertain α/β ratio and additionally varies with the choice of DIR algorithm, the analysis was repeated for 5 α/β ratios ranging from 0 to ∞ and 5 DIR algorithms (Raystation ANACONDA, Plastimatch b-spline and demons, OpenRegGUI Morphons and Cosylab).

**Results**: The expected benefit of proton therapy was confirmed in 97% of all studied cases, despite regular differences of up to 2 percent point (p.p) NTCP between the delivered and planned treatments (Fig. 1). The choice of DIR algorithm affected NTCP up to 1.6 p.p., an order of magnitude higher than the effect of α/β ratio (Fig. 2).

**Conclusion**: The plan-based ΔNTCP evaluation was found robust overall (97%) despite of variations up to 2 p.p. due to delivery inaccuracies.

### P 328 - Monte Carlo feasibility study of PET driven synthetic CT imaging for disease monitoring in proton therapy

#### Martina Moglioni^1,2^, Pietro Carra^1,2^, Davide Bersani^3^, Maria Giuseppina Bisogni^1,2^, Elisa Fiorina^4^, Aafke Christine Kraan^2^, Matteo Morrocchi^1,2^, Francesco Pennazio^4^, Alessandra Retico^2^, Giancarlo Sportelli^1,2^

##### ^1^University of Pisa, Physics, Pisa, Italy ^2^INFN Pisa, Physics, Pisa, Italy ^3^University of Turin, Physics, Turin, Italy ^4^INFN Turin, Physics, Turin, Italy

Proton therapy (PT) is a well-established treatment option for many tumor types and anatomical sites, allowing for better dose conformity and excellent sparing of healthy tissue. However, it is sensitive to anatomical variations in the patient, that can have a negative impact on the efficacy of the treatment plan. In patients where anatomical changes are expected to occur, a control CT is generally prescribed during the treatment course to check the patient morphology. Positron Emission Tomography (PET) is a non-invasive technique used for in-vivo dose monitoring in PT that can help in the decision making for replanning. The interpretation of the PET monitoring data is still a subject of research since PET does not offer a direct representation of the disease progress as a CT does. Our aim is to contribute to overcome this issue by using Neural Networks (NNs), which can produce synthetic CT images starting from the planning CT and the inter-fractional PET monitored data. We studied the feasibility of synthetic CT production with simulations. An example of our annihilation data is given in Fig. 1, which shows the spatial coordinates of the ^+^-emitting isotopes. A Visual Transformer NN was built and trained on five different patients, using the annihilation maps and the planning CT. The output produced the expected intra-fractional synthetic control CTs. The leave-one-out procedure of evaluation has shown that synthetic CTs predicted by our model agreed well with the expected control CT (Fig. 2). This work can be a highly valuable tool in adaptive PT.

### P 329 - Correlation between external surrogate signal and internal motion according to respiratory irregularity, and its impact on respiratory-gated carbon ion radiotherapy

#### Changhwan Kim^1^, Jiwon Jang^2^, Eunho Lee^2^, Seyjoon Park^2^, Soorim Han^1^, Min Cheol Han^1^, Chae-Seon Hong^1^, Jin Sung Kim^1^

##### ^1^Yonsei Cancer Center- Yonsei University College of Medicine, Department of Radiation Oncology, Seoul, Korea Republic of ^2^Yonsei Cancer Center, Department of Radiation Oncology, Seoul, Korea Republic of

In this study, we quantitatively evaluated how motional uncertainties by irregular respiration affect the correlation between external surrogate signal and actual organ motion and investigated its influence on respiratory-gated carbon ion radiotherapy. The representative respiratory signal of the patient was modeled from the one’s external surrogate signal, and the resulting model was established as a reference signal. Using this representative signal as the ground truth, the average value of absolute error was defined as the respiratory irregularity. Based on this quantitative index, the patients were divided into regular and irregular respiration group, and the correlation with internal organ motion acquired from 4DCT was analyzed accordingly. Pearson correlation coefficient and SSIM were respectively calculated for this analysis. As a result, it was confirmed that the correlation with internal motion was lower in the group with irregular breathing than in the regular breathing group. Moreover, it was also determined that the respiratory irregularity may lead to deviations on respiratory bin up to 15% either phase or amplitude level; This may cause ±5∼10% dose hot and cold spots in the worst case. Through these results, it was checked that the patient’s irregular breathing could give adverse effect to respiratory-gated carbon ion radiotherapy. To alleviate this, it would be desirable to use such respiratory guidance and feedback system that can reduce the respiratory irregularity. In addition, phase-controlled rescanning scheme in conjunction with fluoroscopic imaging should be considered to accurately treat even in the presence of such irregularity.

### P 330 - Stopping power estimation from prompt gamma timing data: A new approach to particle therapy verification and quality assurance

#### Julius Friedemann Werner^1^, Veronica Ferrero^2^, Marco Aglietta^2^, Andreas Bolke^1^, Piergiorgio Cerello^2^, Elisa Fiorina^2^, Jona Kasprzak^1^, Anna Vignati^3^, Francesco Pennazio^2^, Magdalena Rafecas^1^

##### ^1^Universität zu Lübeck, Institute of Medical Engineering, Lübeck, Germany ^2^Istituto Nazionale di Fisica Nucleare, Sezione di Torino, Turin, Italy ^3^Università degli Studi di Torino, Department of Physics, Turin, Italy

Electronic stopping power (ESP) is a crucial parameter for particle therapy planning and execution, since it determines the Bragg peak position. Currently, the ESP distribution in the patient is determined from (dual energy) x-ray CT or proton CT. None of these options are available during the irradiation. We are developing new techniques to estimate the ESP from prompt gamma timing (PGT) measurements, i.e., the time difference between primary particle entry and prompt gamma (PG) detection. PGT measurements hold information about the spatiotemporal emission distribution of PGs, which is closely linked to the particle’s motion. By reconstructing the spatiotemporal PG emission distribution and fitting motion models, the ESP can be estimated. Monte Carlo simulations with a homogeneous PMMA phantom show promising results, with an estimation error between 4% and 16% for the ESP corresponding to 0.6±3.1 mm particle range errors for proton energies ranging from 110 MeV to 219 MeV. Next steps include improving the models and algorithms and their extension to non-homogeneous targets. The proposed technique is very promising, since it allows for a direct comparison of the expected and measured stopping power distribution compared to more indirect measures like range estimates or secondary particle distributions. The ESP could be used to calculate the dose of the fraction, updating the treatment plan for the next fraction, or to confirm the conversion models from x-ray CT to stopping power ratio as part of quality assurance.

### P 331 - Log-file based 4D dose tracking for carbon ions

#### Franciska Lebbink^1,2^, Lars Glimelius^3^, Daniel Simon Colomar^3^, Erik Engwall^3^, Dietmar Georg^2^, Markus Stock^1,4^, Barbara Knäusl^1,2^

##### ^1^MedAustron Ion Therapy Centre, Medical Physics, Wiener Neustadt, Austria ^2^Medical University of Vienna, Radiation Oncology, Vienna, Austria ^3^RaySearch Laboratories AB, Physics, Stockholm, Sweden ^4^Karl Landsteiner University, Medical Physics, Wiener Neustadt, Austria

4D dose prediction provides insight into the effect of patient’s breathing and beam-delivery dynamic on the dose distribution, which is especially important for particle therapy with its high motion sensitivity [1]. This work aims to validate carbon 4D dose tracking (4DDT) employing time-resolved measurements. A carbon SOBP treatment plan (5Gy(RBE); RayStation v7.99, PBv4.4) was delivered 5 times to the static and moving (tumor motion 6 and 20mm) ARDOS breathing phantom. The dose was measured with 5 pinpoint (PP) chambers in the target and penumbra region [1]. 4DDT (RSv12.0) recomputed the static dose distribution on 6-10 4DCT phases employing delivery log-file information followed by dose accumulation on the planning CT using deformable image registration. 4DDT could observe a dose distortion caused by motion (Figure1) up to 7.6% (PP1-3) in the SOBP and 13.7% in the distal fall-off (PP5). The measurements for PP1 and 3 agreed with 4DDT within 4% (Table1). PP4 represents an OAR in the penumbra region where the measured dose was slightly higher. The individual measurements of PP2 differed by 0.01, 0.24, 0.16, 0.08 and 0.45Gy from 4DDT. For PP5 the dose deviation of the 20mm motion agreed within 5% with 4DDT, while some inconsistencies were found for smaller motions which might be caused by measurement and 4DCT based motion uncertainties in the high dose-gradient region. 4DDT showed feasible for predicting carbon dose distributions for moving targets but detailed investigations related to dose-averaged LET and non-linear RBE will be necessary before clinical implementation.

### P 332 - Implementation and experience of respiratory gating treatment for a proton therapy system

#### Yuanshui Zheng^1^, Yifeng Yang^1^, Shuyuan Zhang^1^, Taize Yuan^1^, Zuofeng Li^1^, Shen Fu^1^

##### ^1^Guangzhou Concord Cancer Center, Radiation Oncology, Guangzhou, China

**Purpose**: To present the implementation of gating treatment for a PBS proton treatment unit and our experience on its clinical use for lung cancer treatment.

**Methods**: The RGSC system (Varian, Palo Alto, CA, US) was used for 4D CT simulation. RPM (Varian) was used for motion monitoring and gating in the ProBeam proton treatment room (Varian). The gating delay was measured during commissioning. The respiratory signal obtained from the RPM was compared to that from RGSC. An end to end study with a dynamic thorax motion phantom (Sun Nuclear, Norfolk, VA, US) and real patient respiratory signal was conducted to validate the dose delivery of gating treatment on moving targets. 4D CT was evaluated to determine gating window for the lung cancer patient. Gating treatment and repainting technique were used for lung cancer patients.

**Results**: The gating delay was 211.6 ms for beam on and 101.96 ms beam off. The respiratory signal magnitude and breathing period were in agreement between RPM and RGSC. As RPM and RGSC were of different size and shape, careful RPM placement was needed to match the RGSC signal. End to end study with the motion phantom indicated acceptable agreement between TPS calculation and film dose measurement. Initial experience with a lung cancer patient was positive with manageable treatment time. The more detailed effect of time delay on dose for a motion phantom and/or patients is to be investigated.

**Conclusions**: Respiratory gating treatment was implemented for a ProBeam proton system and used clinically in our institution.

### P 333 - Pseudo CT synthesis from CBCT using deep learning In an adaptive proton therapy context

#### Juan Manuel Molina Maza^1^, David Viar-Hernandez^1^, Hugo Alcalde-Benito^1^, Pedro Miguel Martínez Gironés^1^, Borja Rodríguez-Vila^1^, Juan María Pérez-Moreno^2^, Juan Antonio Vera-Sanchez^2^, Alejandro Mazal^2^, Norberto Malpica^1^, Angel Torrado-Carvajal^1^

##### ^1^Universidad Rey Juan Carlos, Medical Image Analysis and Biometry Laboratory, Móstoles, Spain ^2^Centro de Protonterapia Quironsalud, Servicio de Física Médica, Madrid, Spain

Given the required accuracy in protontherapy, Cone-Beam CT (CBCT) scans are necessary in a high proportion of cases for daily positioning and replanning. However, their lower image quality hinders their use for dose calculation and precise patient positioning in adaptive proton therapy treatments. To address this problem, a Convolutional Neural Network (CNN) is proposed to synthesize a CT image from a CBCT scan. Fifteen (15) head and neck CBCT-CT pairs (10 females) with a time difference of less than a week between acquisitions were retrospectively obtained from clinical studies. Data were allocated into training (60%), validation (20%) and test set (20%). CBCT-CT pairs were rigidly registered using RayStation Software, and then divided into 32x32x32 patches due to computational limitations. The CNN implemented is an Unet autoencoder with several residual blocks, each of them applying batch normalization and followed by a Relu activation function. Downsampling and upsampling operations in the network are carried out by strided and transposed convolutions, respectively, and skip connections link layers of equal resolution between the encoding and decoding phase. The Adam optimizer was used for updating the model parameters using the mean absolute error as loss function. Both qualitative and quantitative analysis reveal promising results as synthesized CTs seem to show sufficient quality for replanning proton therapy treatments. Qualitative results showed significant visual agreement between both images. Image similarity metrics, Voxel-by-Voxel Correlation Plot and Bland-Altman Plot all showed that synthetic CTs quantitatively agreed with the ground truth.

### P 334 - Institution-specific motion management methods and analyses for forthcoming thoracic lymphoma treatments with proton pencil beam scanning - Preliminary results

#### Gabriela Foltyńska^1^, Weronika Dziechciarz^1^, Magdalena Garbacz^1^, Dominika Kędzierska-Pardel^1^, Tomasz Mikołajski^1^, Monika Radolińska^1^, Karolina Sobkowicz^1^, Urszula Sowa^1^, Tomasz Skóra^2^, Renata Kopec^1^

##### ^1^Institute of Nuclear Physics Polish Academy of Sciences, Cyclotron Center Bronowice, Kraków, Poland ^2^The Maria Sklodowska-Curie National Research Institute of Oncology, Kraków Branch, Kraków, Poland

According to the recommendations and guidelines of the Particle Therapy Co-Operative Group (PTCOG) Thoracic and Lymphoma Subcommittee, any institution planning to treat moving tumors in the thoracic region using pencil beam scanning (PBS) proton therapy, should develop their own criteria for motion management strategies. Those criteria should be based on the available options and limitations of the treatment planning system, as well as monitoring and delivery equipment. The list of clinical indications for proton radiotherapy in Poland extends continuously- soon to be supplemented with malignancies of moving organs. Due to this fact, we have begun to test feasibility of using available techniques and tools (T&Ts) to effectively and safely implement the treatment of moving targets. Among those T&Ts we concentrate on: 4DCT- motion analysis with respiratory phantom, CT calibration consideration, surface guidance with Vision RT system, patient immobilization devices and procedures; treatment planning- beam arrangement analysis, optimization technique (single- or multi-field), 3D robust optimization with Varian Eclipse NUPO optimizer; and treatment delivery- IBA cyclotron-based PBS with dedicated beam model, repainting strategies and surface guidance/gating during treatment. The gantry room with phantom setup and surface imaging system at proton therapy center in Poland is presented in Figure 1. Preliminary results from performed tests and analyses will be presented and widely discussed.

### P 335 - Evaluation of Hitachi four-dimensional cone beam computed tomography (4D-CBCT) at different virtual gantry rotation speeds

#### Sridhar Yaddanapudi^1^, Chunjoo Park^1^, Bo Lu^1^, Xiaoying Liang^1^, Ronald Zhu^2^, Chris Beltran^1^, Keith Furutani^1^, Thomas Whitaker^2^

##### ^1^Mayo Clinic, Radiation Oncology, Jacksonville, USA ^2^UT MD Anderson Cancer Center, Radiation Physics, Houston, USA

Hokkaido Cancer Center has demonstrated four-dimensional cone beam computed tomography (4D-CBCT) for the Hitachi compact proton gantry. Mayo Clinic Florida and MD Anderson Cancer Center are both Hitachi conventional large gantry pencil beam scanned proton therapy systems. This study evaluates the 4D-CBCT image quality of the Hitachi dual-axis CBCT image-guided system. Respiratory correlated 4D-CBCT images are acquired for a large range of bpm including 10 and 15bpm for the conventional one revolution per minute (6 and 4 sec period respectively), with the CIRS Dynamic Thorax Phantom moving 5.0 mm and 15.0 mm in the superior-inferior direction. In addition, a reference CBCT with no motion applied to the phantom is acquired for multiple gantry rotations. A virtual gantry rotation speed is then generated from the multiple repeated acquisitions using multiples of the period since a change in period is equivalent to a change in gantry rotation speed. The 4D-CBCT projections are binned into ten phases for the different virtual gantry rotation speeds in the respiratory cycle using the Amsterdam Shroud method and validated using the CIRS Dynamic Thorax Phantom motion information. All the CBCT projection data is reconstructed by applying the Feldkamp-Davis-Kress (FDK) method including each of the ten phases. Image quality metrics are presented as a function of virtual gantry rotation speed, amplitude, and nominal phantom motion period. $I=I_{0}*sin^{4}\left(\frac{\pi *t}{motion\;period}+phase\;advance\right)\;t:\left\{0,\ldots ,60\right\}secs$

### P 336 - Validating Eclipse capability of modelling 3D range-modulators for FLASH-IMPT

#### Yuri Simeonov^1^, Miriam Krieger^2^, Michael Folkerts^3^, Gerard Paquet^3^, Petar Penchev^1^, Klemens Zink^1,4^, Uli Weber^5^, Christoph Schuy^5^

##### ^1^Technische Hochschule Mittelhessen THM University of Applied Sciences, Life Science Engineering LSE, Gießen, Germany ^2^Varian Medical Systems Particle Therapy GmbH, Flash, Troisdorf, Germany ^3^Varian Medical Systems, Flash, California, USA ^4^Marburg Ion Beam Therapy Center, Radiation therapy, Marburg, Germany ^5^GSI Helmholtzzentrum für Schwerionenforschung, Biophysics division, Darmstadt, Germany

**Purpose**: A 3D-range-modulator (3D-RM) combined with a high-intensity proton-beam can enable FLASH irradiation. However, detailed Monte Carlo modeling and direct optimization of 3D-RM pin-shapes can be prohibitively resource intensive. The creation of a modulator-pin geometry from an analytical IMPT framework could be an efficient approach for potential clinical routine application. This work investigates the implementation of a 3D-RM modelling-capability in Eclipse. Both the IMPT-plan and its corresponding 3D-RM were exported from Eclipse and simulated with FLUKA.

**Methods**: The dose in Eclipse was modelled analytically using the maximum beam-energy at the nozzle and variable-thickness range-shifters (“RaShi” method). Thus, any treatment plan consists of multiple water-equivalent-thickness (WET)/scan-spot layers, which facilitate the use of already existing IMPT-planning-techniques. A 3D-RM was developed by converting the spot-weights and WET-layers from a RaShi-plan, optimized for spherical target of 5cm diameter, to step-shaped pins. Two MC-simulations were then performed using 250MeV proton-beam and 17cm PMMA pre-absorber: RaShi simulation: the scan-spot map for each thickness-layer was exported from the DICOM-RTPLAN. Additionally, FLUKA was customized to modulate the range-shifter-thickness on the fly in a single simulation, and 3D-RM simulation: the cumulative raster plan was calculated and used to irradiate the modulator.

**Results**: There is excellent agreement between the dose distributions from the original RaShi-plan and the 3D-RM, as shown in Fig.1. The Gamma-Index passing rate (1%/1mm, local) was 99.8%.

**Conclusion**: It is possible to reproduce the complex energy spectrum and 3D dose distribution from an IMPT-plan by using one single energy and a 3D-RM. This capability has been successfully implemented and validated in Eclipse.

### P 337 - A TLD array for dosimetry in ultra-high dose rate narrow proton beams

#### Silvia Motta^1^, Jeppe Christensen^1^, Michele Togno^2^, Robert Schäfer^2^, Sairos Safai^2^, Tony Lomax^2^, Eduardo Yukihara^1^

##### ^1^Paul Scherrer Institute, Department of Radiation Safety and Security, Villigen PSI, Switzerland ^2^Paul Scherrer Institute, Center for Proton Therapy, Villigen PSI, Switzerland

Given the challenges of dosimetry in ultra-high dose rate beams and the interest for FLASH radiotherapy in proton beams, we propose LiF:Mg,Ti thermoluminescence detectors (TLDs) for dosimetry of ultra-high dose rate proton beams. This study investigates the use of a matrix of TLDs to reconstruct the narrow proton beam, as well as possible dose rate effects of LiF:Mg,Ti. Arrays of 5 × 5 LiF:Mg,Ti cubes (1.0 mm side) were irradiated in the entrance region of a 250 MeV single-spot proton beam. Average dose rates in the range (1 - 4500) Gy/s were investigated. Volume averaging effects resulting from the narrow spot size at the highest dose rate (Gaussian beam sigma < 2 mm) were estimated and corrected with a beam reconstruction procedure. A Faraday cup, a diamond detector, and radiochromic films were used for reference dosimetry. The lateral beam spread from the beam reconstruction procedure agrees with the measurements with radiochromic films within 4%. No indication of dose rate effects in LiF:Mg,Ti was observed within 5% from the reference detectors at all dose rates. The dose rate independence of LiF:Mg,Ti makes them suitable for dosimetry in ultra-high dose rate proton beams. The detector small size can support in-vivo dosimetry of biological experiments for FLASH radiotherapy. Moreover, the use of an array of detectors and a procedure of beam reconstruction can be applied to determine the beam profile of narrow proton beams.

### P 338 - Experimental demonstration of 360nA FLASH proton beam current via synchrocyclotron using IBA Proteus®ONE

#### Nicolas Gerard^1^, Jarrick Nys^1^, Lucian Hotoiu^1^, Maximilien Melissas^1^, Nicolas Bruyr^1^, Vincent Engelen^1^, Jerome Mandrillon^1^, Hao Gao^2^, Yuting Lin^2^

##### ^1^Ion Beam Application, Innovation and Development, Louvain-La-Neuve, Belgium ^2^University of Kansas Medical Center, Department of Radiation Oncology, Kansas City, USA

To obtain a FLASH dose rate > 40Gy/s in a large field, a high proton beam current is required. In 2019, the superconducting synchrocyclotron (S2C2) of the IBA ProteusOne clinical system delivered 70nA at isocenter [1]. In this work, the beam current has been substantially increased to 360nA. Firstly, the beamline transmission efficiency has been increased to 60% at the highest energy (228MeV) through optimization of the beamline optics and S2C2 parameters. This modification was implemented in the Clinical ProteusOne Proton machine at University of Kansas Medical Center. We were able to measure a current of 200nA with a spot sigma of 5 mm at isocenter. Secondly, the S2C2 output has been significantly increased by hardware upgrades. This upgrade was implemented in the factory but can be easily deployed in clinical system. A beam current up to 600nA was measured on Faraday cup at S2C2 exit. If this is implemented at a clinical system, it will translate in a beam current of 360nA at isocenter. Future improvements of the accelerator are still under investigation. We then investigated the feasibility of applying the dose current to a large field using the www.openFLASH.software. The dose rate achievable with these currents in large fields was computed using the open source TPS. Dose rate > 40Gy/s can be delivered by ConformalFLASH in a 7×10cm field.

### P 339 - Development of pixel-type real time monitoring system for flash beam

#### Youngjae Jang^1,2^, Tae-keun Yang^1^, SeongHee Park^2^, HongSuk Jang^1^, JeongHwan Kim^1^, Moo-Jae Han^1^, Gyu-Seok Cho^1^, KumBae Kim^1^, Geun-Beom Kim^1^, Sang Hyoun Choi^1^

##### ^1^Korea Institute of Radiological & Medical Sciences, Research Team of Radiological Physics & Engineering, Nowon-gu, Korea Republic of ^2^Korea University, Department of Accelerator Science, Sejong, Korea Republic of

**Introduction**: Flash radiation therapy is a state-of-the-art treatment technology that maximizes the treatment effect by delivering a high dose of radiation within a very short time within one second. In order to accurately measure ultra-high dose rate radiation, a system capable of measuring in real time while minimizing signal loss is required. In this study, we developed a pixel-type monitoring detector and DAQ system that can measure the dose and geometry accuracy of flash radiation in real time, and evaluated its usefulness in a proton beam.

**Materials and Methods**: The pixel-type detector developed in this study is consists of 256 pixels, and the maximum size of the measuring beam is 6.4cm × 6.4cm. The proton beam used in the experiment was 45MeV/10∼70nA and 226MeV/1.8nA. We collected the beam profile and data using DAQ system during beam monitoring.

**Results and Discussion**: The center position of the proton beam identified was confirmed to be within 0.3cm from the center of the detector and the dispersion value was 2.0. By comparing the beam profile monitored in real time with the dose value irradiated on the film, it was confirmed that the ultra-high dose rate proton beam could be measured.

**Conclusion**: In this study, the profile of the beam measured in real time was compared and analyzed with the film. It was confirmed that the developed detector can accurately measure the position and intensity of the proton beam. Based on these results, a beam profile measurement for the ultra-high dose rate electron beam will be carried out.

### P 340 - Preclinical proton minibeam radiotherapy facility for small animal irradiation

#### Aikaterini Rousseti^1^, Georgios Kourkafas^2^, Jessica Neubauer^1^, Michael Mayerhofer^1^, Andrea Denker^2^, Jürgen Bundesmann^2^, Günther Dollinger^1^, Judith Reindl^1^

##### ^1^Universität der Bundeswehr München, Institute for Applied Physics and Measurement Technology- Department for Aerospace Engineering, Neubiberg, Germany ^2^Helmholtz-Zentrum Berlin, Protons for Therapy, Berlin, Germany

Numerous in vivo studies have demonstrated that combining protons and spatial fractionation for cancer treatment, can lead to better sparing of healthy tissues. We present beamline simulations for a preclinical proton minibeam radiation therapy (pMBT) facility at the cyclotron of the Helmholtz-Zentrum Berlin (HZB). The intention is small animals irradiation using focused 68 MeV (approx. 4 cm in water) proton minibeams with a sigma of 50 μm, at center-to-center (ctc) distance as small as 1 mm of adjacent minibeams and a high peak-to-valley dose ratio (PVDR). The minibeams will be delivered in scanning mode and with a beam current of 1 nA. After the beam extraction from the cyclotron, a first degrader determines the maximum energy of the beam. Dipole magnets and quadrupole triplets transport the beam to the treatment room while multiple slits properly form the transverse beam profiles. A high magnetic field gradient triplet lens forms the small minibeams in front of the target station and, scanning magnets are used for a raster scan application at the target area. A second degrader, positioned close before the focusing spot and the target, further reduces the energy, creating a spread-out Bragg peak (SOBP). A small animal radiation research platform (SARRP) will be used for imaging and positioning of the target. These results will contribute to the construction of a preclinical pMBT facility giving the opportunity for systematical research in the field of proton minibeam radiotherapy.

### P 341 - Improved nozzle design for FLASH irradiation of small samples using a double scattering system and a 3D printed ridge filter

#### Jens Heufelder^1^, Andreas Weber^1^, Alina Dittwald^2^, Jürgen Bundesmann^2^, Timo Fanselow^2^, Georgios Kourkafas^2^, Andrea Denker^2^

##### ^1^Charité - Universitätsmedizin Berlin, BerlinProtonen am HZB, Berlin, Germany ^2^Helmholtz-Zentrum Berlin, Protons for Therapy, Berlin, Germany

Based on our technical experience for 68 MeV proton FLASH irradiations of single mice eyes [1], we improved the layout of our nozzle to allow shorter irradiation times, larger irradiation fields, and deeper penetration depths. Now both injectors are equipped with an electrostatic kicker in front of the cyclotron in addition to the fast mechanical beam stoppers used in the treatment of ocular tumors. The FPGA-control-system was modified for driving the kicker. A voltage of about 800 V is used to deflect the protons from their pass into the cyclotron within µs. The single scattering setup was turned into a double scattering system (figure). A 40 µm tantalum foil acts as first scatterer. The second consists of polyethylene and tantalum. Instead of a modulator wheel a 3D printed ridge filter made from M2S-HT90 is used. The changes allow irradiation of samples with 20 mm diameter using a spread out Bragg peak with full modulation and 26.5 mm range in water (figure). FLASH pulses down to 2 ms with a dose reproducibility of better than 2% and dose rates up to 900 Gy/s are possible, using a bunched injection into the cyclotron. Switching between clinical dose rates and FLASH dose rates takes about 15 min. The described setup was used for the irradiation of sarcoma and fibroblasts organoids with a dose rate of 80 Gy/s. Acknowledgements: HZB cyclotron crew (proton beam), U. Zuther (workshop), C. Albinus (3D printing)

### P 342 - FLASH-RT dose threshold effect estimation for particle beams by modelling solvate electron production dependency on particle LET 3D distribution

#### Kristaps Palskis^1,2^, Toms Torims^2^, Maurizio Vretenar^1^, Mariusz Sapinski^3^, Joao Seco^4^

##### ^1^European Organization for Nuclear Research CERN, ATS-Directorate Office ATS-DO, Geneva, Switzerland ^2^Riga Technical University, Centre of High Energy Physics and Accelerator Technologies, Riga, Latvia ^3^Paul Scherrer Institute, Beam instrumentation group SY/BI, Villigen, Switzerland ^4^German Cancer Research Center DKFZ, BioMedical Physics in Radiation Oncology E041, Heidelberg, Germany

**Background and Aims**: While there is a growing clinical interest and emerging technological developments for FLASH-RT with different ionizing particle beams, the majority of relevant research has been done with the use of electron, photon or proton beams. Investigations of achievable FLASH effect with heavy ions are therefore needed for understanding the clinical potential of particle beams for FLASH-RT.

**Methods**: The study investigates the dose threshold effect of FLASH-RT with proton and heavy ion beams, by modelling the achievable FLASH effect through the production yields of solvated electrons (e^−^_aq_) and spatial differences of production yields due to LET dependency. Solvated electrons have been chosen as surrogate for indication of FLASH effect based on their reactivity with cellular oxygen, therefore standing as possible mediators of oxygen dependency of FLASH-RT. Pristine pencil beam Bragg peak 3D dose distributions of various ion energies were simulated with GEANT4 and biological spread-out Bragg peaks (SOBP’s) were calculated from these sets for different field sizes and treatment depths. Various heavy particle types vere investigated - protons and ions of ^3^He, ^4^He, ^6^Li, ^7^Li, ^9^Be, ^10^B, ^12^C, ^14^N, ^16^O and ^20^Ne. Mixed beam linear energy transfer (LET) 3D distributions were calculated and were used for estimation of solvated electron production yields.

**Results**: FLASH-RT dose threshold levels for different heavy ion beams are estimated, indicating impact of SOBP geometry and field size on this threshold. Dose threshold effect spatial difference maps are indicated due to solvated electron production yield dependency on LET.

### P 343 - Proton multi-beam FLASH radiotherapy: Combining FLASH and IMPT

#### Joana Leitão^1,2,3^, Juan María Pérez^4^, João Lourenço^1,2,5^, Patrícia Gonçalves^2,3^, Alejandro Mazal^4^, João Seco^1,6^

##### ^1^German Cancer Research Center DKFZ, Biomedical Physics in Radiation Oncology, Heidelberg, Germany ^2^Instituto Superior Técnico, Física, Lisboa, Portugal ^3^Laboratório de Instrumentação e Física Experimental de Partículas LIP, Dosimetria, Lisboa, Portugal ^4^Centro de Protonterapia Quirónsalud, Física- Pozuelo De Alarcón, Madrid, Spain ^5^Centro de Ciências e Tecnologias Nucleares C^2^TN, Department of Nuclear Sciences and Engineering, Lisboa, Portugal ^6^Univeristy of Heidelberg, Physics and Astronomy, Heidelberg, Germany

We propose a novel strategy for multi-beam FLASH-PT, combining FLASH Bragg Peak-in-target beams and IMPT, that could be applied in deep-seated tumors or in complicated treatment sites. We defined “the FLASH effect” to occur in healthy tissue if irradiated with a minimum dose of 10 Gy and a minimum dose rate of 100 Gy/s. We used matRad, a treatment planning tool kit developed at DKFZ. We defined a target within a phantom surrounded by either one or two OARs in different positions (see Figure 1), plus a reference OAR which is spared. A passive scattered beam complying with the FLASH conditions is used for the target region close to an OAR. The remaining target volume is irradiated with 2 to 7 optimized proton IMPT beams to achieve the correct tumor dose coverage. Figure 1 shows the dose distribution with the best coverage and homogeneity for each geometry tested. All plans achieved adequate target coverage, V(Prescribed Dose) > 0.98, and target uniformity, HI < 0.07, while complying with the FLASH conditions in the directly irradiated OARs (in black in Figure 1). For the robustness analysis, we found that the dose delivered to the CTV was not affected by systematic or random shifts. Future work entails applying our method to different clinical sites and comparing the results with the clinical standard.

### P 344 - Is the new proton therapy facility ERHA PL230 a candidate accelerator for delivering Ultra-high dose rate?

#### Lidia Strigari^1^, Roberto Orecchia^2^, Raffaele Andrea Prisco^2^, Giovanni Rutigliani^2^, Luigi Picardi^2^

##### ^1^IRCCS Azienda Ospedaliero-Universitaria di Bologna, UOC Fisica Sanitaria - Dipartimento Malattie oncologiche ed ematologiche, Bologna, Italy ^2^Linearbeam srl, Particle Accelerator, Ruvo di Puglia, Italy

Ultra-high dose rate (UHDR) radiotherapy is an intriguing modality primarily performed using electron and proton beams. Increasing evidence from in vivo pre-clinical studies is found about a remarkably better sparing of healthy tissues without decreasing the tumor control of UHDR (> 40 Gy/s). However, no proton accelerators are designed ad hoc for delivering UHDR PT. To evaluate the ability to deliver UHDR proton therapy using our linear proton accelerator, we calculated the instantaneous dose rate values and the main dosimetric characteristics of our new facility. The ERHA PL230 is a new proton therapy facility designed and under construction by LinearBeam in Ruvo di Puglia (Italy). It consists of an injector followed by a sequence of accelerating modules, SCDTL up to 27 MeV and CCL up to 230 MeV. The system is completed by a short magnetic beamline and a delivery line equipped with scanning magnets in order to perform a full 4D irradiation of target. The expected ERHA PL230 pulse dose rates are larger than 100 kGy/s in the Bragg Peak region for each single voxel (e.g. at 30 uA pulse current, 3 microsecond pulses, 3 Gy/pulse @ 160 MeV, at the Bragg peak averaged within 1 mm^3^). Our preliminary results at the test site confirm the calculated characteristics, and the agreement between the calculated and measured EHRA dosimetric parameters, as well as possible further upgrades will be the subject of the presentation. Our facility, in commissioning phase, presents several dosimetric features that make it a candidate for delivering UHDR

### P 345 - Prototype passive scattering system for FLASH irradiation of biological samples at the synchrocyclotron room of a clinical facility

#### Adrian Zazpe^1^, Juan Antonio Vera^2^, Alejandro Mazal^2^, Samuel España^1^, Luis Mario Fraile^1^, Jose Manuel Udias^1^, Daniel Sanchez-Parcerisa^1^

##### ^1^Universidad Complutense de Madrid, Nuclear Physics Group & IPARCOS, Madrid, Spain ^2^Quironsalud Proton Therapy Center, Medical Physics, Madrid, Spain

While the dose rate achievable in the treatment room of a clinical protontherapy facility is, at present, below the FLASH threshold (usually 40Gy/s), we present a prototype irradiation station for small samples that can be installed along the transport line, upstream of the range modulator, in a clinical synchrocyclotron room (Fig1a). Previous measurements with radiochromic film have shown an achievable dose rate in shoot-through mode (using 230-MeV protons) of 2.2 kGy/s in an area of 5 mm^2^ (80% isodose) with the nude beam. Our system, composed of 3D-printed elements and lead collimators (Fig1b), was designed to (1) produce a homogeneous field in microwells used for *in-vitro* experiments, and (2) be set up and aligned in seconds, given the potentially high activation of the room. Using TOPAS simulations, we optimized the collimator design to produce a field as homogeneous as possible, given the limited irradiation volume, and the uncertainty in the (unfocused) beam position (up to 3 mm). With our prototype design, the 80% isodose area was increased to 26 mm^2^ while maintaining a dose rate above 800 Gy/s (Fig 2a), using the experimental dose distribution (Fig 2b) as an input for the simulation after correcting for beam divergence, which was measured in a separate experiment. With the proposed prototype irradiation system, FLASH irradiations of biological samples can take place at the IBA proteusOne facility at Quironsalud Madrid.

### P 346 - Radiation shielding evaluation for an existing proton center for FLASH radiotherapy

#### Tony Wong^1^, Matt Ruth^1^, Leland Palmer^2^, Francois Vander Stappen^3^, Jatinder Saini^1^, Marco Schwarz^1^

##### ^1^Fred Hutchison Cancer Center - Proton Therapy, Medical Physics, Seattle, USA ^2^IBA, IBA-Seattle, Seattle, USA ^3^IBA, IBA-Philadelphia, Philadelphia, USA

**Purpose**: FLASH radiotherapy (FLASH-RT) has continuously attracted vast interest in radiation oncology with the potential to widen the therapeutic window of normal tissue complication probability and tumor control probability. Our proton facility has four treatment rooms with a common beam line, and one room (the Gantry room) can deliver FLASH-RT. This study reports our preliminary investigation of radiation shielding for FLASH-RT.

**Method**: FLASH-RT requires a minimum dose rate of 40Gy/s and delivery in a sub-second range. We estimated the required cyclotron beam current for a transmission FLASH-RT of a 10 cm^2^ field at 230 MeV will be 700 nA. To account for the response time of the wide-energy neutron detector (WENDI-II), we reduced the beam current to 100 nA for our radiation shielding measurements. The measurements were performed at fourteen locations surrounding the cyclotron vault and the Gantry room with a 25×30×30 cm^3^ solid water at the isocenter at 0 gantry angle.

**Results**: With a conservative workload estimation of treating five FLASH-RT patients per hour, three beams per patient, two seconds per beam, eight hours per day, and fifty-two weeks per year, the annual occupational or public doses at the fourteen locations were well within the annual dose limits and the time-averaged dose rate limit. The maximum occupational dose of 1.3 mSv/year (2.6% of the maximum limit) was at the isocenter of the fixed-beam room adjacent to the cyclotron vault.

**Conclusion**: The radiation shielding evaluation indicates that our proton center designed for conventional proton treatment, is adequate for FLASH-RT.

### P 347 - Beam monitors for a compact medical synchrotron based ultra-high dose-rate scanned carbon-ion beam contributing to further development of FLASH irradiation

#### Masashi Yagi^1^, Shinichi Shimizu^1^, Noriaki Hamatani^2^, Toshiro Tsubouchi^2^, Masaaki Takashina^2^, Takuya Nomura^3^, Takuto Miyoshi^4^, Masumi Umezawa^3^, Kazuhiko Ogawa^5^, Kanai Tatsuaki^2^

##### ^1^Osaka University Graduate School of Medicine, Department of Carbon Ion Radiotherapy, Suita, Japan ^2^Osaka Heavy Ion Therapy Center, Department of Medical Physics, Osaka, Japan ^3^Hitachi Ltd., Healthcare Business Division, Kashiwa, Japan ^4^Hitachi Ltd., Research & Development Group, Hitachi, Japan ^5^Osaka University Graduate School of Medicine, Department of Radiation Oncology, Suita, Japan

**Background**: An ultra-high dose-rate (uHDR; FLASH) dedicated beam monitors are needed to control the uHDR beam due to a different range of measured beam current compared to the normal dose-rate.

**Aims**: This study aimed to develop beam monitors for an uHDR scanned carbon-ion irradiation with a compact-type medical synchrotron.

**Methods**: Large plane parallel ionization chambers (ICs) with the active area of 120 mm×120 mm×3 mm and dedicated electrical circuit are newly developed (Fig.1(a)). The characteristics of the electrical circuit were measured with a function generator. The medical synchrotron extracted ≥1.0 × 10^9^ carbon-ions at 208.3 MeV/u within a maximum 100 ms. The carbon-ion beam was scanned once to create a field within the extraction time. The dosimetric characteristics of the ICs were evaluated. Advanced Markus chamber and Gafchromic film were used to measure the absolute dose and the field size respectively.

**Results**: Output frequency and input current for the electrical circuit were nicely linear (Fig.1(b)). The FLASH irradiation utilizing beam scanning was confirmed with a dose of 9.7 ± 0.08 Gy (homogeneity of ±2.0%) for the beam in a volume of at least 16 mm × 16 mm in a square field and a corresponding dose-rate of 106.5 Gy/s (± 5.5 Gy/s) (Fig.1(c, d)). The dose was changed to 1.6, and 5.4 Gy for the dose-rate and the field size.

**Conclusion**: The developed dedicated beam monitors can control uHDR scanned carbon-ion beams with the compact-type medical synchrotron keeping the FLASH dose-rate >40 Gy/s at different dose levels in a useful field size for research.

### P 348 - Proton minibeam radiation therapy for cardiac radioablation: A proof of concept

#### Maria Giorgi^1,2^, Pierre Loap^3^, Jorge Miguel Sampaio^1,2^, Youlia Kirova^3^, Yolanda Prezado^4^

##### ^1^LIP - Laboratório de Instrumentação e Física Experimental de Partículas, Dosimetria, Lisboa, Portugal ^2^Faculdade de Ciências da Universidade de Lisboa, Departamento de Física, Lisboa, Portugal ^3^Institut Curie, Department of Radiation Oncology, Paris, France ^4^Institut Curie, Signalisation Radiobiologie et Cancer, Orsay, France

**Purpose**: Proton minibeam radiation therapy (pMBRT) is a new radiotherapy technique which reduces normal tissue damage thanks to the spatial fractionation of the dose. Single-fraction proton therapy has been proposed to treat refractory ventricular tachycardia. This study aims to evaluate the potential sparing of the organs at risk (OARs) and cardiac substructures using pMBRT in cardiac radioablation treatments by means of Monte Carlo simulations (TOPAS version 3.7).

**Material and Methods**: The contours for a ventricular tachycardia case were drawn in CT images of an anonymized patient by a radiation oncologist. The simulated clinical target volume was located on the left ventricular free wall and has a volume of 17.1 cm3. A prescribed dose of 25 Gy(RBE) in one fraction was considered. Two different plans for pMBRT were evaluated: (i) two fields of minibeams spaced by 3 mm to get a quasi-homogeneous dose in the target and (ii) one array of minibeams spaced by 9 mm leading to a heterogeneous dose. pMBRT dose distributions were compared to pencil beam scanning (PBS) dose distributions evaluated by ECLIPSE treatment planning system. Comparison included tumor coverage and mean dose to OARs.

**Results**: In both plans, pMBRT provide a similar or lower mean dose to the cardiac substructures than conventional proton therapy and a significantly reduced penumbra.

**Conclusions**: The promising results obtained in this theoretical study aim to further evaluate the use of pMBRT for cardiac radioablation.

### P 349 - FLASH Whole-brain mice irradiations at PSI: Irradiation setup

#### Serena Psoroulas^1^, Robert Schaefer^1^, Isabella Colizzi^1^, Michele Togno^1^, Vivek Maradia^1^, Martin Grossmann^1^, Tony Lomax^1,2^, Damien Charles Weber^1,3,4^, Sairos Safai^1^, David Meer^1^

##### ^1^Paul Scherrer Institut, Centre for Proton Therapy, Villigen PSI, Switzerland ^2^ETH, Physics, Zurich, Switzerland ^3^University of Bern, University Hospital Bern, Bern, Switzerland ^4^University Hospital Zurich, Radiation Oncology, Zurich, Switzerland

How dose rate should be calculated for implementing the FLASH effect in the clinic is an open question. Experimental data show that the average dose rate resp. an irradiation time of hundreds of milliseconds is required to trigger the FLASH effect, but the correlation between delivery parameters makes it hard to single out their relative impact. For translation to larger treatment volumes, the role of the local dose rate needs to be understood. At PSI Gantry 1, we conducted FLASH experiments in scattering conditions, using both transmission and Bragg peak mode. Our scattering setup consists of polystyrene slabs, placed at the end of the beamline as required by the beam size. Depending on penumbra or field size required, we utilize copper collimators. This setup is optimized for both transmission and Bragg peak irradiations, using a custom 3-D printed ridge filter in case an SOBP larger than a few millimeters is required. Our transmission scattering setup reached an average dose rate of 115 Gy/s in mice irradiations. With our SOBP scattering setup, we achieved an average dose rate higher than 500 Gy/s. We can vary further the dose-rate to design experiments aimed at distinguishing the impact of different delivery parameters, such as pauses of variable length (which can be introduced by our flexible in-house control system) or using spot scanning and spot placement to achieve different local dose-rate maps, still under development. Both features will be used in the next round of biological experiments and potentially treatments of pets with spontaneous tumors.

### P 350 - Quantities for reporting proton mini-beam radiotherapy

#### Fardous Reaz^1^, Mateusz Sitarz^2^, Niels Bassler^2^, Erik Traneus^3^

##### ^1^Aarhus University, Clinical Medicine, Aarhus, Denmark ^2^Aarhus University Hospital, Danish Centre for Particle Therapy DCPT, Aarhus, Denmark ^3^RaySearch Laboratories AB, Research and Development, Stockholm, Sweden

Proton minibeam therapy (pMBRT) can possibly be a way to further enhance the therapeutic window. A multi-slit collimator (MSC) is the most convenient solution to produce a mini-beam dose profile. An optimum configuration of an MSC is essential for biological validation and quantification experiments of pMBRT. However, the quantification and reporting of pMBRT fields are not adequately defined, occasionally leading to ambiguities. Using Monte Carlo simulations, we investigate quantities that characterize pMBRT of an actual proton beam model to obtain more explicit definitions applicable in a realistic setup. The center-to-center (CTC) distance is typically reported based on the MSC geometry. However, the actual CTC obtained from a dose distribution varies in a realistic beam, depending on the position in the field. We also define the geometrical throughput (gTP) as the ratio of the aperture area to the field-size. Throughput, in general, is a meaningful quantity that scales with neutron yield, beam delivery efficiency, and collimator activation. However, we discriminate here to the actual throughput (aTP), which may be very different from gTP since aTP depends on the beam model, and MSC design, as we show in Figure 1. The accuracy of peak and valley dose calculation depends on the scoring voxel width along the spatial fractionated direction. 10% to 40% width of the assessed volume segment can provide a representative value of doses with a reasonable error, but larger voxels will underestimate the peak dose or vice versa for the valley dose.

### P 351 - A prototype secondary standard calorimeter for dosimetry of conventional and ultra-high dose rate protons

#### Jack Aylward^1,2^, Graham Bass^3^, Michael Taylor^1,4^, Ranald MacKay^1,2^, Karen Kirkby^1,4^, Russell Thomas^3,5^

##### ^1^The University of Manchester, Division of Cancer Sciences- Faculty of Biology- Medicine and Health, Manchester, United Kingdom ^2^Christie NHS Foundation Trust, Christie Medical Physics and Engineering, Manchester, United Kingdom ^3^National Physical Laboratory- Hampton Road- Teddington- Middlesex- UK, Medical Radiation Physics, Teddington, United Kingdom ^4^Christie NHS Foundation Trust, Christie, Manchester, United Kingdom ^5^University of Surrey, Faculty of Engineering and Physical Sciences, Guildford, United Kingdom

Ionisation chambers are commonly used as secondary standard detectors, however these are less suited for ultra-high dose rates (UHDR), or FLASH dosimetry, because of collection inefficiency due to ion recombination. Calorimetry shows promise for UHDR: it is suited for irradiations of high dose of short duration to minimise heat loss from the detector core during the measurement. A low-cost secondary standard calorimeter has recently been developed at the UK’s National Physical Laboratory (NPL) and has been tested in x-ray and electron beams, including at UHDR electrons [1]. This work tests the feasibility of the calorimeter, and characterises the response, in conventional dose rate and UHDR Pencil Beam Scanning (PBS) protons. A 245 MeV PBS gaussian proton beam (σ_x_ = 4.6 mm, σ_y_ = 6.6 mm) was used to characterise the calorimeter at a reference depth of 2 cm in water. The linearity of dose response was assessed up to 60 Gy. Dose rate dependence was assessed up to 110 Gy/s corresponding to a maximum nozzle current of 56.4 nA. Repeatability was assessed for a 10 × 10 cm scanned field. Repeatability was measured to be 0.67% and 1.93% at conventional and UHDR respectively (one standard deviation). Conclusion: The suitability of a low-cost calorimeter as a secondary standard detector of conventional and UHDR protons is presented.

### P 352 - Implementation of proton mini-beam radiation therapy (pMBRT) using a clinical compact proton system

#### Daniel Johnson^1^, Yolanda Prezado^2^, Gregory Gan^1^, Weijie Zhang^1^, Wangyao Li^1^, Hao Gao^1^, Yuting Lin^1^

##### ^1^University of Kansas Medical Center, Radiation Oncology, Kansas City, USA ^2^Institut Curie, Signalisation radiobiologie et cancer, Orsay, France

**Purpose**: To implement a proton mini-beam radiation therapy (pMBRT) system to facilitate the clinical translation of pMBRT, we have tested the feasibility of implementing a multi-slit collimator using the beam structure and the beam data specific to KU’s IBA Proteus®ONE compact gantry proton system.

**Methods**: The proton beam is produced using pencil beam scanning technique. The brass multi-slit collimator is designed through comprehensive Monte Carlo simulations [1], using the beam structure and the beam data specific to our IBA Proteus®ONE proton system, with 0.4mm width per slit, 4 mm center-to-center distance, and 0.1 degree divergence tailored to the IBA Proteus®ONE beam divergence. The collimator can be mounted to the system via the snout accessory as seen in the Figure 1B. We implement a unique design where we kept the outer fitting to the snout, and can insert various design of collimators, as shown in Figure 1C.

**Results**: A uniform 150MeV proton beam was scanned over a 10cm × 10cm field with 0.25cm spot spacing, and the relative dose was measured using Galchromic films. A proof of principle measurements were demonstrated in Figure 2 implementing the multi-slit collimator in a clinical compact gantry proton system. The dose delivered to the surface is about 8 Gy and delivery time of one minute. Future work includes quantitative analysis of peak valley dose ratio at various measurement depths and its comparison with Monte Carlo simulations.

### P 353 - A novel dose-rate optimization method to maximize ultra-high dose-rate coverage of organs-at-risk without compromising dosimetry metrics in proton PBS FLASH-RT

#### Xingyi Zhao^1^, Sheng Huang^2^, Haibo Lin^3^, J. Isabelle Choi^3^, Charles B. Simone II^3^, Kun Zhu^1^, Xueqing Yan^1^, Minglei Kang^3^

##### ^1^Peking University, State Key Laboratory of Nuclear Physics and Technology, Beijing, China ^2^Tianjin Medical University Cancer Institute & Hospital, Radiation Oncology, Tianjing, China ^3^New York Proton Center, Radiation Oncology, New York, USA

**Purpose**: To present the implementation of a novel genetic algorithm(GA) method for PBS spot delivery sequence optimization to achieve FLASH dose rate optimization under relatively low cyclotron beam currents.

**Materials and Methods**: Dose and dose rate were optimized sequentially. Firstly, a multi-field FLASH plan was developed to meet all the dosimetric goals. Then, a novel GA method was implemented into the in-house treatment platform to maximize the dose rate by exploring the best spot delivery sequence. A phantom with a C-shape target and 10 consecutive lung patients were planned using 45GyRBE in 3 fractions transmission FLASH-RT to evaluate the effectiveness. Time averaged-dose-rate(ADR, including spot and scanning time), FLASH dose-rate volume histogram(DRVH), and dose rate matric V_40GyRBE/s_ were investigated to assess the efficacy of dose rate optimization quantitatively.

**Results**: Using a relatively low MU/spot of 150 and nozzle beam current of 65nA, the V_40GyRBE/s_ of the core was increased from 0 to 60%(Fig1) in the phantom study. In the lung patients, the major OARs, including lung-GTV, spinal cord, heart, and esophagus, FLASH V_40GyRBE/s_ were improved from 44.4%, 45.8%, 45.8%, 49.4% to 63.5%, 82.9%, 61.4%, and 87.5%, respectively (p-values all<0.001, except heart p-value of 0.207). The averaged V_40GyRBE/s_ for the target and OARs was increased from 43.6% to 75.6%, showing a significant dose rate increase for a clinical cyclotron proton system(Fig2).

**Conclusion**: The phantom and patient results demonstrated that this novel GA effectively improves dose rate coverage for OARs, which can potentially allow for OAR toxicities reductions through the FLASH effect.

### P 354 - Proton pencil beam scanning FLASH radiotherapy dose rates: Numerical analysis in vivo

#### Seyyedeh Azar Oliaei Motlagh^1^, Michele Kim^1^, Ioannis I. Verginadis^1^, Beryl Bell^2^, Francois V. Stappen^2^, Wei Zou^1^, Costas Koumenis^1^, Lei Dong^1^, Eric S. Diffenderfer^1^

##### ^1^University of Pennsylvania, Department of Radiation Oncology, Philadelphia, USA ^2^IBA, Ion Beam Applications S.A., Louvain-la-Neuve, Belgium

**Purpose**: To simulate the delivered dose to a group of mice in proton pencil beam scanning (PBS) FLASH modality in a clinical gantry room and to calculate different volume histograms of dose averaged dose rate (DADR) and PBS dose rate using the recorded machine log files.

**Methods**: A group of 19 mice was given a 14Gy radiation dose through the whole abdomen using 5×5 pencil beam spots spaced at 6mm in the IBA Proteus Plus gantry treatment room. Using a 2×2 cm^2^ collimator, a sharp field penumbra was achieved. For PBS FLASH irradiation, the beam current was 800nA. Based on calculations, 67.4 percent of the planned target volume (PTV) received a 14Gy dose with PBS dose rates of 246-248Gy/s and DADR of 1069-1102Gy/s. The PBS dose rate is derived by considering only a substantial percentage of the voxel dose and the time needed to deliver that dose (Marlen et al., 2022). The dose rate weighted by the dose fraction of each spot to a voxel averaged across all spots is referred to as the DADR. (Marlen et al., 2022).

**Results**: The mean dose delivered with the FLASH beam at PTV was 14.0Gy with mean dose rates of 125.6-127.3Gy/s, mean DADRs of 1105.4-1139.0Gy/s, and mean PBS dose rates of 253.4-256.6Gy/s. The total time of exposure was 115.5-117ms.

**Conclusion**: Our findings suggested that the target was effectively irradiated in the clinical gantry room, opening the door for FLASH radiation to be used safely and effectively in clinical settings.

### P 355 - Experimentally validating a cost-effective Bragg-peak tracking technique for FLASH radiotherapy

#### Shouyi Wei^1^, Haibo Lin^1^, J. Isabelle Choi^1^, Charles B. Simone II^1^, Minglei Kang^1^

##### ^1^New York Proton Center, Radiation Oncology, New York, USA

**Purpose**: To experimentally validate a cost-effective Bragg-peak tracking technique that can be applied for more affordable FLASH radiotherapy(RT).

**Method and Materials**: Energy degrader, selection, and focusing systems were eliminated to preserve high beam transmission efficiency for ultra-high dose rate FLASH-RT. Bragg-peak distal-tracking can be achieved using a universal range shifter(URS) and beam-specific range compensators (RC). PBS spot weights and placement were optimized to generate intensity-modulated proton(IMPT)-equivalent plan quality by in-house TPS. A homogeneous solidwater phantom with a cylindrical target and an anthropometric phantom with a “brain tumor” were prescribed to 10Gy/fraction for plan optimization. RCs were designed and manufactured by a 3D printer(Figure1). The nozzle current was scaled down to 10nA to avoid the saturation of MatriXX PT for dose measurement. A high-spatiotemporal resolution 2D strip-chamber array was used to measure the spot delivery time structure under FLASH beam currents. 3D dose rates were reconstructed using the dose and time measured by in-house tools.

**Results**: The 3D-printed RCs can accurately adapt the Bragg-peak to the target distally. The measured 2D dose is consistent with calculated values, with gamma passing rates>99% in solidwater phantom and >98% in anthropometric phantom using 2mm/2% thresholds. The point dose difference between the TPS and measurement is <2%. The strip ion chamber array can reliably measure the spot time, benchmarked with machine logfiles, for 3D dose rate reconstruction under FLASH beam currents(Figure2).

**Conclusion**: The experimental validation demonstrates the dosimetric accuracy and robustness of this novel delivery method. The Bragg-peak tracking method can potentially be applied for FLASH-RT.

### P 356 - LIGHT proton linac multi-energy conformal FLASH minibeam as an alternative to photon SRS treatments

#### Anna Maria Kolano^1,2^, Wioletta Kozlowska^1,2^, Tara Gray^3^, Chieh-Wen Liu^3^, Ping Xia^3^, Jonathan Farr^1,2^

##### ^1^Applications of Detectors and Accelerators to Medicine SA, Clinical Office, Meyrin, Switzerland ^2^Advanced Oncotherapy plc, Clinical Office, London, United Kingdom ^3^Cleveland Clinic Foundation, Radiation Oncology, Cleveland, USA

**Purpose**: To investigate Ultra-High Dose Rate (UHDR) minibeams produced by the LIGHT (Advanced Oncotherapy plc, UK) proton linac (PL), as an alternative to photon Stereotactic Radiosurgery (SRS) for multiple brain metastasis treatment.

**Methods**: PLs have output dose rates invariant on energy and fast spot and energy switching of 5 ms, allowing for multi-energy FLASH target irradiation within 0.5s. Additionally, PLs can generate minibeams (∼1/3 nominal spot size) - a good alternative to photon SRS.

Using LIGHT minibeam parameters, treatment plans (single uniform field per target) were created. The FLASH-optimised plans were retrospectively compared to photon plans regarding target coverage, dose to Organs at Risk (OARs), and Dose-Rate average (DR_av_).

**Results**: Minibeam plans reached UHDRs of 120 Gy/s, with target size-dependent DR_av_ (Figure 1). Reduced spot counts maximised the FLASH effect (FE). FLASH minibeam plan quality was superior to the photon SRS, with comparable conformity and lower OAR doses (Table 1). D2 brain dose was reduced up to 50% compared to photons (0 Gy for other OARs). Allowing higher dose inside the tumour, spot reduction, and spot charge optimisation minimised local increase in brain dose at D2/1.

**Conclusions**: Clinically relevant PL conformal minibeam plans, applied to brain metastasis, offer equal or better conformity than photon SRS, with additional benefit of the FE. In studied cases, the UHDR averaged over all brain targets was around 60 Gy/s for FLASH minibeams. Target conformity was comparable to nominal photon plans, with significantly reduced OAR doses. Submillimetre PL beams are potential candidates for conformal FLASH therapy.

